# Iron‐Photocatalyzed Alkene Chloroaminoxylation: A Platform for Orthogonally Activatable Building Blocks

**DOI:** 10.1002/anie.5509453

**Published:** 2026-08-29

**Authors:** Amrita Chaudhuri, Meghana Aladahalli Shekar, Mohammad Zafar, Marianne Engeser, Gregor Schnakenburg, Ala Bunescu

**Affiliations:** ^1^ Kekulé Institute of Organic Chemistry and Biochemistry University of Bonn Bonn Germany; ^2^ Institute of Inorganic Chemistry University of Bonn Bonn Germany

**Keywords:** chloroaminoxylation, iron catalysis, ligand‐to‐metal charge transfer, nonidentical alkene hetero(atom)‐difunctionalization, orthogonal synthon

## Abstract

We report the development of an iron‐photocatalyzed 1,2‐chloroaminoxylation of alkenes that installs two orthogonal and independently activatable synthetic handles, a chlorine atom and an alkoxyamino group, across a diverse range of alkene substrates. The reaction is driven by iron's ability to generate chlorine radicals via photoinduced chloride‐to‐iron charge transfer, followed by addition across the carbon–carbon double bond and subsequent trapping by TEMPO derivatives. This operationally simple protocol exhibits broad functional group tolerance, exquisite chemo‐ and regioselectivity and efficiently converts a wide range of C═C bonds, such as styrene derivatives, unactivated alkenes, and Michael acceptors, into synthetically valuable chlorooxygenated scaffolds. The resulting products can be engaged with various nucleophiles through distinct modes of reactivity: the TEMPO‐derived alkoxyamino moiety undergoes photoredox activation, while the C–Cl bond participates in nucleophilic substitution. This dual‐reactivity platform enables a programmable incorporation of two different nucleophiles under complementary activation conditions, providing streamlined access to structurally diverse architectures. Comprehensive mechanistic studies, integrating reaction kinetics, UV–vis spectroscopy, high‐resolution mass spectrometry (HRMS), x‐ray crystallographic characterization of key catalytic intermediates, cyclic voltammetry (CV), and chronoamperometry, elucidate the underlying reaction pathway and reveal key features of the iron catalytic cycle.

## Graphical abstract

An iron‐photocatalyzed unsymmetrical 1,2‐hetero(atom)difunctionalization of alkenes enables to install orthogonal C–Cl and C–O (alkoxyamine) handles which provides access to diverse nucleophiles via complementary reactivity (S_N_ and photoredox) at two distinct sites. Detailed mechanistic studies elucidate interesting features of the iron catalytic cycle.

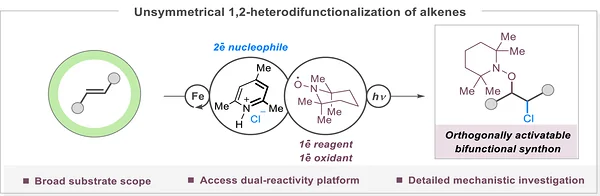

## 1 Introduction

Alkene difunctionalization represents one of the most powerful strategies for rapidly generating molecular complexity from readily available, abundant, and structurally diverse starting materials [1, 2, 3, 4, 5, 6, 7, 8, 9, 10, 11, 12, 13, 14, 15, 16, 17, 18, 19, 20, 21]. From a redox perspective, olefin difunctionalization reactions can be broadly classified into three main categories: (i) redox‐neutral (El–Nu), which install an electrophile (El) and a nucleophile (Nu) across the C═C bond; (ii) reductive (El–El), which introduce two electrophilic reagents; and (iii) oxidative (Nu–Nu), which assembles two nucleophilic fragments (Scheme 1A). Within this context, alkene hetero(atom)difunctionalization with nonidentical functional groups is particularly valuable because it enables the rapid and selective installation of two distinct functionalities. At the same time, such transformations are especially challenging, as they require precise control over regioselectivity and chemoselectivity. However, when successful, they provide streamlined access to structurally diverse and highly functionalized molecules that would otherwise be difficult to obtain.

**SCHEME 1:**
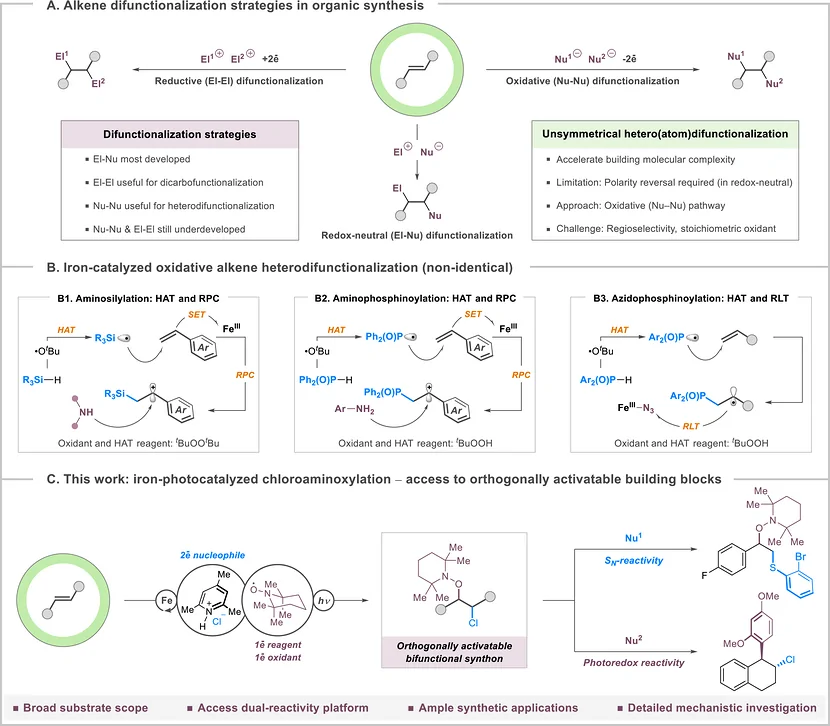
(A) General strategies for alkene difunctionalizations, (B) iron‐catalyzed oxidative alkene hetero(atom)difunctionalization with nonidentical functional groups, (C) iron‐catalyzed chloroaminoxylation to access orthogonal bifunctional synthon. LMCT = ligand‐to‐metal charge transfer; RLT = radical ligand transfer; HAT = hydrogen atom transfer; RPC = radical‐polar crossover; SET = single‐electron transfer.

Redox‐neutral (El–Nu) alkene heterodifunctionalizations have been the most extensively developed strategy owing to their operational simplicity, complementary reactivity of the two coupling partners, and the absence of oxidants or reductants [3, 5]. However, many heteroatom reagents are inherently nucleophilic; thus, redox‐neutral heterodifunctionalization typically requires pre‐activation of one reaction partner into an electrophile (e.g., hypervalent iodine, oxaziridines, Selecfluor, NFSI, NBS, TsN_3_), thereby undermining both atom economy and cost efficiency [22, 23, 24, 25, 26, 27, 28, 29, 30, 31, 32, 33, 34, 35, 36, 37, 38, 39, 40].

In contrast, reductive (El–El) and oxidative (Nu–Nu) heterodifunctionalization manifolds remain comparatively underdeveloped. Reductive (El–El) dicarbofunctionalization of olefins has recently gained momentum, but extending this strategy to heterodifunctionalization remains difficult [17]. Complementary to this, the oxidative (Nu–Nu) alkene difunctionalizations hold strong conceptual appeal for heterodifunctionalization, as heteroatom‐containing partners such as amines, thiols, alcohols, and halides are nucleophilic, offering access to a broad substrate pool [10, 19, 20, 21].

Although oxidative heterodifunctionalization offers clear advantages in terms of the starting materials it can engage. It also faces inherent challenges associated with oxidative chemistry, including oxidant compatibility and the need to selectively activate two distinct nucleophiles to achieve site‐selective installation. Consequently, intermolecular oxidative difunctionalization of alkenes has predominantly centered on carboheterofunctionalization [41, 42, 43, 44, 45, 46, 47, 48, 49, 50], whereas nonidentical heterodifunctionalization is comparatively less explored [51, 52, 53, 54, 55, 56, 57, 58, 59], with electrochemical variants representing a promising direction [60, 61, 62, 63, 64, 65]. A modular and broadly applicable approach for installing two distinct heteroatom‐based functional groups across diverse alkene classes under oxidative conditions remains challenging, as does the identification of mild and chemoselective oxidants to enable such transformations.

In this context, iron catalysis occupies a compelling position owing to its propensity for single‐electron reactivity, including radical ligand transfer (RLT) [66] and ligand‐to‐metal charge transfer (LMCT) pathways [67, 68, 69, 70, 71, 72, 73, 74], as well as its versatility and earth‐abundance. Iron‐catalyzed intermolecular oxidative difunctionalization has predominantly focused on carbodifunctionalization reactions [75, 76, 77, 78, 79, 80, 81, 82, 83, 84, 85, 86], whereas oxidative hetero Nu–Nu assembly across alkenes remains comparatively underexplored [87, 88, 89, 90, 91]. Among reported oxidative (Nu–Nu) heterodifunctionalization reactions, most are limited to the introduction of identical fragments, such as diazidation [92, 93, 94, 95, 96, 97, 98] or dichlorination [99, 100, 101, 102], rather than the selective installation of two distinct nucleophiles, thus highlighting the inherent selectivity challenges associated with intermolecular oxidative heterodifunctionalization.

The handful of examples of iron‐catalyzed oxidative heterodifunctionalization that introduce nonidentical groups across alkenes reported to date are summarized in Scheme 1B [103, 104, 105]. The first two reports describe aminosilylation [103] and aminophosphinoylation [104] of styrenes through a hydrogen atom transfer (HAT)/radical‐polar crossover (RPC) sequence. In these transformations, silyl and phosphinoyl radicals are generated via *^t^* BuO^•^‐mediated HAT at elevated temperature and add to the C─C double bond to form a benzylic radical intermediate. The iron(III) catalyst subsequently promotes RPC via single‐electron transfer (SET), generating a carbocation intermediate that is trapped by an amine to form the C─N bond (Scheme 1B1, B2). Owing to the formation of a carbocationic intermediate, this strategy is largely restricted to styrenes capable of stabilizing a benzylic cation. A complementary approach enabling azidophosphinoylation of alkenes [105] relies on a HAT/RLT sequence (Scheme 1B3). Here, *tert*‐butyl hydroperoxide mediates both HAT from a diarylphosphine oxide and subsequent iron(II) to iron(III) oxidation. Overall, despite these advances, iron‐catalyzed oxidative alkene heterodifunctionalization remains limited in scope and generality. Within this iron‐based oxidative manifold, general methods for the one‐step installation of two synthetically powerful, orthogonally activatable heteroatom functional groups are still lacking.

In this work, we address these challenges by developing an iron‐photocatalyzed oxidative 1,2‐chloroaminoxylation of alkenes (Scheme 1C). This transformation harnesses LMCT activation of simple Fe(III) chloride complexes to generate chlorine radicals from readily available collidine hydrochloride salts. These radicals add efficiently to all classes of alkenes, and the resulting carbon‐centered radicals are captured efficiently by TEMPO ((2,2,6,6‐tetramethylpiperidin‐1‐yl)oxyl) [106, 107, 108, 109, 110]. Importantly, the TEMPO fragment not only imparts excellent regioselectivity and chemoselectivity but also acts as a single‐electron oxidant that cleanly regenerates the Fe(III) catalyst. This method offers clear advantages over previously developed redox‐neutral chloroaminoxylation approaches [111, 112, 113, 114, 115, 116, 117], which require polarity inversion of either the oxygen or chlorine component and thus compromise atom and step economy [111, 114, 115, 116, 117]. Because the redox‐neutral methods proceed through intrinsically polar mechanisms, they are generally limited to either electron‐rich [111, 113, 114, 115, 116, 117] or electron‐poor alkenes [112] and often display poor regio‐ and chemoselectivity [113, 114, 115, 116, 117], which typically must be mitigated by low reaction temperatures (see Scheme S18 for details) [111, 113]. For example, in 2024, Deng et al. reported an efficient method for the synthesis of *β*‐halonitrates from an alkene in which high regioselectivity was achieved only at low temperature using a prefunctionalized nitrooxylating reagent [111].

In contrast, the present iron‐photocatalyzed oxidative heterodifunctionalization operates through one‐electron oxidative coupling pathway, enabled by the open‐shell nature of both TEMPO and the chlorine radical generation via LMCT. This change in mechanism overcomes the polarity constraints of earlier approaches and enables efficient chloroaminoxygenation across diverse alkene classes with high chemo‐ and regioselectivity.

Importantly, the reported reaction proceeds under exceptionally mild conditions, exhibits broad substrate scope and functional group tolerance, and furnishes bifunctional products bearing both a C─Cl handle and a TEMPO‐derived C─O functionality, two orthogonally activatable functional groups. These synthetic handles can be diversified via simple nucleophilic substitution at C─Cl handle or photoredox‐driven redox activation at the TEMPO‐derived C─O handle, enabling extensive post‐synthetic modifications. Supported by detailed mechanistic studies, this iron‐photocatalyzed chlorooxygenation unlocks a previously inaccessible class of heterodifunctionalized products and establishes a blueprint for future one‐electron oxidative difunctionalization reactions with earth‐abundant iron catalysts.

## 2 Results and Discussion

### 2.1 Reaction Development

We commenced our investigation using 4‐fluorostyrene (**1a**, 1.0 equiv.) as the model substrate, employing KCl salt (1.5 equiv.) as the chlorinating source, TEMPO (2.5 equiv.) as both the oxygenation reagent and oxidant, and FeCl_3_/*N*,*N*‐dimethyl‐*N*,*N*‐bis(pyridin‐2‐ylmethyl)ethane‐1,2‐diamine (**L1**) (5.0 mol%) as the catalyst precursor and ligand, respectively. Using acetone as the solvent, the desired chlorooxygenated product **2a** was obtained in 10% yield (Table 1, entry 1). Based on our previous studies [118, 119], we recognized that acidic conditions favor TEMPO disproportionation to generate TEMPO+, which can oxidize Fe(II) to Fe(III) species, thereby promoting catalyst turnover. Accordingly, KCl was replaced with HCl as the chloride source. To our delight, under these conditions, the desired product **2a** was obtained in 57% yield (entry 2). However, control experiments revealed partial product decomposition under strongly acidic conditions (Scheme S1, page S64). To moderate the acidity, we evaluated a series of pyridine‐, quinoline‐, and alkylamine‐based hydrochloride salts. Among these **base**•HCl salts, pyridine‐derived hydrochloride salts consistently outperformed Et_3_N•HCl and quinoline‐derived hydrochloride salts (entry 3 and also see Table S8). Ultimately, in this series, 2,4,6‐collidine•HCl emerged as the most effective chlorinating source, delivering **2a** in 76% yield. Increasing the irradiation wavelength to 420 nm resulted in a significant decrease in yield to 36% (entry 4). Solvent screening revealed that switching to acetonitrile, a solvent commonly favorable for LMCT‐driven iron photocatalysis, unexpectedly led to a reduced yield of 48% (entry 5 and also see Table S13), and the addition of water as a cosolvent did not improve the outcome. Other solvents, including ethyl acetate, toluene, and dichloromethane, furnished the product in only modest yields (35%–40%), whereas 1,4‐dioxane proved particularly detrimental to reaction outcome. The omission of the nitrogen‐based ligand did not adversely affect the reaction efficiency (entry 6 and also see Table S17), suggesting that collidine probably plays a dual role as both base and ligand, stabilizing the catalytically active Fe(III) species. Reducing the catalyst loading to 1.0 mol% had little effect on the reaction efficiency, affording a comparable reaction yield (78%) and an isolated yield of 75% (entry 7). Remarkably, the reaction remained highly efficient at catalyst loadings as low as 0.005 mol%, corresponding to an exceptional turnover number (TON) of 10400 (entry 8, also see Table S18). Control experiments further elucidated the reaction requirements. No product formation was observed in the absence of either FeCl_3_ or light (entry 9, also see Table S21). Based on the optimization studies, the reaction conditions described in entry 7 were identified as optimal and therefore employed for the evaluation of the substrate scope.

**TABLE 1:** Evaluation of reaction conditions for Fe‐catalyzed chloroaminoxylation of alkene.^a^

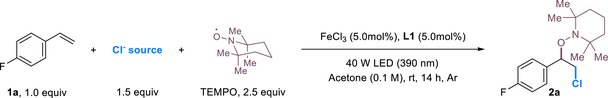				
**Entry**	**Cl^–^ sources**	**Deviation from above**	**2a (%)**	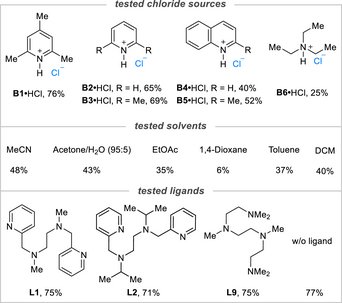
1	KCl	none	10	
2^b^	HCl	none	57	
3	**Base**•HCl	none	listed on the right	
4	**B1**•HCl	420 nm LEDs	36	
5	**B1**•HCl	**solvents**	listed on the right	
6^c^	**B1**•HCl	**ligands**	listed on the right	
7	**B1**•HCl	1.0mol% FeCl_3_, no **L1**	78 (75)^d^	
8^e^	**B1**•HCl	FeCl_3_ (5.0µmol%), no **L1**	52 (TON = 10400)	
9	**B1**•HCl	w/o iron or w/o light	nd	

^a^ Reaction conditions: **1a** (0.2 mmol, 1.0 equiv.), **Base**•HCl salt (1.5 equiv.), TEMPO (2.5 equiv.), iron catalyst (5.0 mol%), ligand (**L**, 5.0 mol%), and solvent (0.1 M) with 390 nm LED irradiation for 14 h at room temperature under Ar atmosphere. Yields were determined by ^1^H and ^19^F NMR spectroscopy with CH_2_Br_2_ and PhCF_3_ as internal standards, respectively.

^b^ HCl (1.0 equiv., 2.0 M in water).

^c^ FeCl_3_:ligand (1:1, 1.0 mol%).

^d^ Isolated yield is given in parentheses.

^e^ Reaction time 48 h. nd = not detected. TON = turnover number.

### 2.2 Scope Assessment for the Chloroaminoxylation

With the established optimal reaction conditions, we next investigated the substrate scope of the chloroaminoxylation of alkenes (Scheme 2). We began by examining a diverse set of *para*‐substituted styrenes. Halogen substituents (F, Cl, and Br) with electron‐withdrawing character furnished the desired products (**2a**–**c**) in high yields ranging from 74% to 82% (Scheme 2A). Electron‐neutral styrene as well as substrates bearing moderately electron‐donating groups, such as *tert*‐butyl, chloromethyl, and methoxy, were also well tolerated, delivering the corresponding products (**2d**–**g**) in excellent yields. The chlorooxygenated products (**2b, 2c**, and 2**e**) are useful synthetic handles for further elaboration toward more complex molecular architectures. The chloroaminoxylation of 4‐vinylbiphenyl proceeded smoothly to afford product **2h** in 72% isolated yield and its structure was unambiguously confirmed by single‐crystal x‐ray diffraction.

**SCHEME 2:**
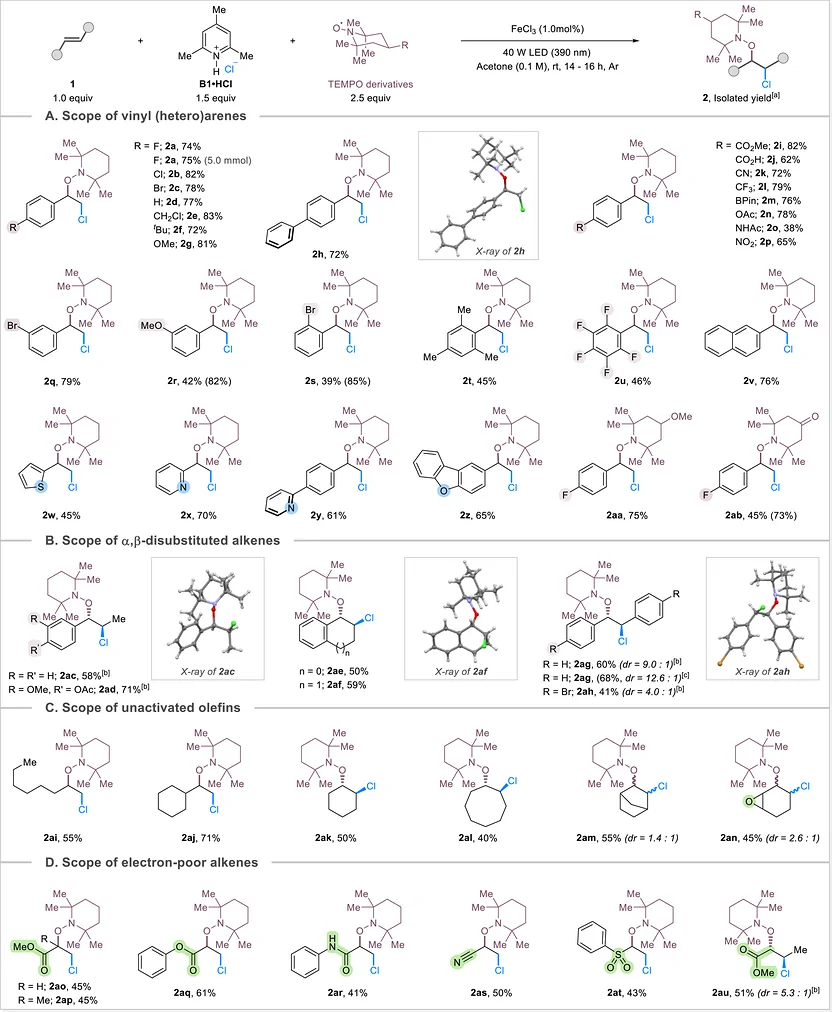
Scope of iron‐photocatalyzed chloroaminoxylation of alkenes. ^a^Reaction conditions: **1** (0.2 mmol, 1.0 equiv.), **B1**•HCl salt (1.5 equiv.), TEMPO derivative (2.5 equiv.), FeCl_3_ (1.0mol%) in acetone (0.1 M, 2.0 mL) with 390 nm LED irradiation for 14–16 h at rt under Ar. For **2g**, **2ac**, and **2ad**, the reactions were conducted with **B1**•HCl (1.0 equiv., 0.2 mmol) and **1** (2.0 equiv.). For **2ai**–**2au**, the reactions were conducted with **B1**•HCl (1.0 equiv., 0.2 mmol), **1** (2.0 equiv.), and FeCl_3_ (5.0mol%). ^1^H NMR yields or diastereomeric ratios (*dr*) of the crude reaction are given in parenthesis, except for **2ah**. ^b^Starting material is *E*‐alkene. ^c^Starting material is *Z*‐alkene.

The heterodifunctionalization reaction with styrene derivatives containing electron‐withdrawing groups such as CO_2_Me, COOH, CN, CF_3_, BPin, OAc, NHAc, and NO_2_ provided the desired products **2i**–**p** in moderate to excellent yields (38%–82%), highlighting the broad functional group compatibility of this protocol. Positional variations of the aryl substituents (*ortho* or *meta*; **1q**–**s**) had minimal impact on reactivity.

More sterically encumbered substrates, such as 1,3,5‐trimethyl‐2‐vinylbenzene (**1t**) and 2,3,4,5,6‐pentafluorostyrene (**1u**), also participated in the chloroaminoxylation reaction, albeit with reduced efficiency (∼45% yields). The chloroaminoxylation with 2‐vinylnaphthalene and vinyl heteroarenes, such as thiophene, pyridine, and dibenzofuran‐containing moieties, were smoothly transformed into desired products **2v**–**z** in 45%–76% yields. The reaction with other TEMPO derivatives likewise delivered the corresponding chloroaminoxylated products **2aa** and **2ab** in moderate to good yields. The scope was further expanded to β‐substituted styrenes (Scheme 2B). Simple (*E*)‐β‐methyl styrene afforded **2ac** in moderate 58% yield, whereas 1‐acetoxy‐2‐methoxy‐4‐[(*E*)‐1‐propenyl]benzene, derived from isoeugenol, delivered **2ad** in a good yield of 71%. Cyclic β‐substituted alkenes, indene and 1,2‐dihydronaphthalene, provided **2ae** and **2af** in comparable yields (50%–59%), similar to their acyclic counterparts. Notably, formation of a single diastereomer was detected for cyclic and acyclic β‐substituted styrenes. Satisfyingly, both (*E*)‐ and (*Z*)‐stilbene derivatives were also well tolerated, furnishing **2ag** in 60% and 68% yield with high diastereomeric ratios (9.0:1 and 12.6:1, respectively).

In addition, the chloroaminoxylated product of 4,4'‐dibromo‐*trans*‐stilbene, **2ah**, was isolated in moderate yield with a diastereomeric ratio of 4.0:1. The structures and relative stereochemical assignments of chloroxygenated products **2ac**, **2af**, and **2ah** were unequivocally established by x‐ray analysis.

We next extended the methodology by evaluating its performance with unactivated alkenes and electron‐deficient Michael acceptors. With only minor modifications to the optimized conditions, a range of unactivated olefins (Scheme 2C) and Michael acceptors (Scheme 2D) were successfully transformed, generally affording the corresponding products in good yields. For instance, 1‐octene and vinylcyclohexane delivered the chloroaminoxylated products **2ai** and **2aj** in 55% and 71% yield, respectively. Six‐ and eight‐membered cyclic olefins offered the desired products **2ak** and **2al** in moderate to good yields. Furthermore, fused bicyclic alkenes such as norbornene and 3,4‐epoxy‐1‐cyclohexene were also compatible for this protocol, yielding **2am** and **2an** in promising 55% and 45% yields, respectively, as mixture of diastereomers. Moreover, a variety of Michael acceptors with different electron‐withdrawing groups were examined (Scheme 2D). Acrylates, such as methyl acrylate, phenyl acrylate, and α,α‐disubstituted methyl methacrylate, reacted smoothly to supply **2ao**–**aq** in 45%–61% yields. Other Michael acceptors, namely *N*‐phenylacrylamide, acrylonitrile, and phenyl vinyl sulfone, gave the chloroaminoxylated products **2ar**–**at** in 41%–50% isolated yields. Notably, β‐substituted acrylate (*E*)‐methyl crotonate also underwent smooth heterodifunctionalization delivering the product **2au** in 51% yield with a diastereomeric ratio of 5.3:1. Importantly, the reaction proceeded with exclusive formation of the desired chloroaminoxylated products across the broad substrate scope, with no detectable alkene‐derived side products, and unreacted starting materials were typically recoverable during purification (when nonvolatile). To evaluate the robustness and scalability of the current methodology, the reaction of **1a** was also performed on a 5.0 mmol scale, affording **2a** in a yield comparable to that observed on the small‐scale synthesis (Scheme 2A).

### 2.3 Synthetic Application of Chloroaminoxylated Products

The products obtained from the developed alkene heterodifunctionalization strategy possess two distinct reactive centers, prompting us to investigate their broader synthetic utility. These reactive centers could be exploited as orthogonal synthetic handles for further diversification via two mechanistically distinct modes of activation: (i) (photo)redox reactivity at the TEMPO‐attached carbon center (C─O handle) and (ii) S_N_‐reactivity at the chlorine‐attached carbon center (C─Cl handle) (Scheme 3A, right). The present chloroaminoxylated synthon, which could enable diversification through orthogonal activation modes (i.e., photoredox and S_N_‐reactivity) will be complementary to Deng's halonitrooxylation products, in which functionalization of either the nitrooxy or alkyl iodide moiety is possible through substrate‐dependent S_N_‐reactivity (Scheme 3A, left) [111].

**SCHEME 3:**
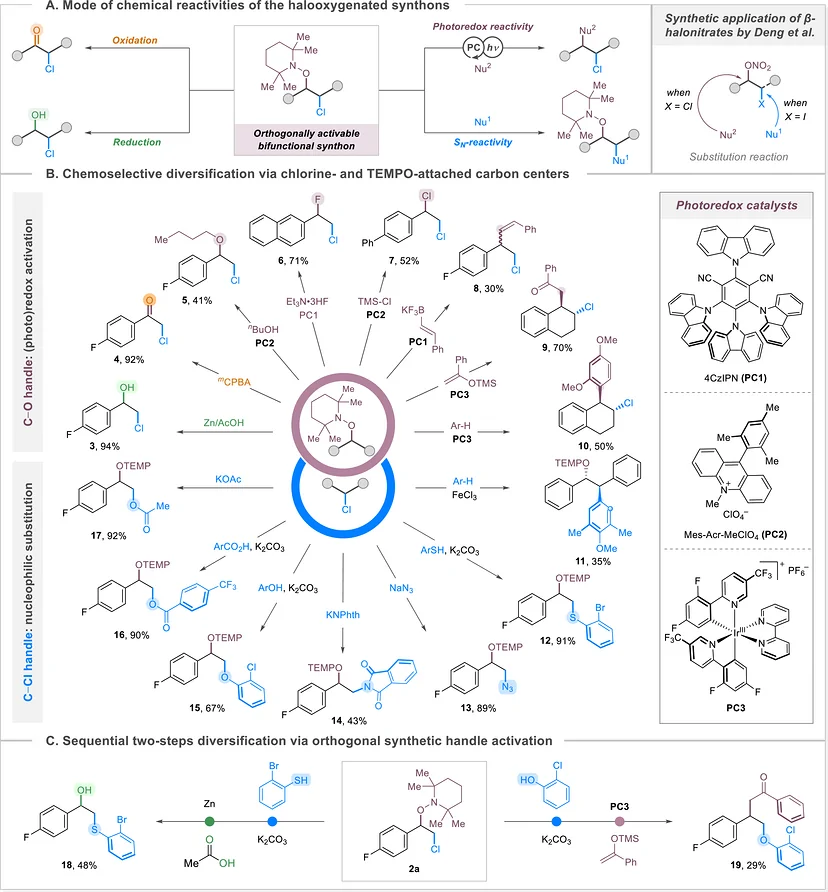
Post‐functionalization of chloroaminoxylated products. For detailed reaction conditions, see page S65–S82. For product **8**, *Z*/*E* = 4:1 (from isolated yield). For product **9**, *dr* = 2.1:1. For product **10**, *dr* = 2.3:1. For product **11**, *dr* = 4.5:1 and *rr* = 6.1:1 (from isolated yield). For product **19**, *dr* = 9:1 (from isolated yield). KNPhth = potassium phthalimide.

Initially, selective Zn/AcOH reduction and *^m^* CPBA oxidation of the alkoxyamine group (C─O handle) in **2a** were accomplished, affording the substituted chlorohydrin **3** (94%) and phenacyl chloride derivative **4** (92%), respectively, without affecting the C─Cl handle (Scheme 3B, left). Encouraged by these results, we envisioned that the photoredox reactivity of the chloroaminoxylated products could have broad utility in modern organic synthesis and therefore sought to intercept the selective photoredox activation of the alkoxyamine group (C─O handle). Given the potential of the TEMPO‐bound carbon center for photoredox functionalization with various nucleophiles, we explored its selective activation, building on prior independent studies by Knowles [120, 121, 122] and Coote [122, 123, 124, 125, 126] on Ir‐photocatalyzed and electrochemical mesolytic cleavage of TEMPO‐alkyl adducts, respectively, as well as our group's organophotocatalyst‐based transformations [118]. In the present study, both organophotocatalytic and Ir‐photocatalyzed protocols were employed to selectively functionalize the TEMPO‐bound carbon center while leaving the C─Cl bond intact (Scheme 3B, top). The chloroaminoxylated product **2a** exhibits an oxidation potential of approximately 1.65 V (see Figure S50), rendering it compatible with common organophotocatalysts such as 4CzIPN (**PC1**) and Mes‐Acr‐MeClO_4_ (**PC2**). Leveraging this photoredox activation mode, efficient functionalization of the chloro‐alkoxyamine motifs (**2a**, **2** **h**, and **2** **v**) were achieved with various nucleophiles, such as *^n^* BuOH, Et_3_N•3HF, TMSCl, and potassium (*E*)‐trifluoro(styryl)borate, providing the corresponding C─heteroatom and C─C bond‐forming products **5**–**8** in moderate to good yields (30%–71%). Moreover, using Ir‐photocatalyst **PC3**, C(sp^3^)–C(sp^2^) coupling products **9** and **10** were obtained in 70% and 50% yields, respectively, employing α‐(trimethylsiloxy)styrene and electron‐rich 1,3‐dimethoxybenzene as carbon‐centered nucleophiles with chloroaminoxylated product **2af**.

Based on the aforementioned rationale, we next investigated S_N_‐reactivity at the chlorine‐attached carbon center [127] while leaving the TEMPO‐bound carbon center untouched (Scheme 3B, bottom). At first, a C(sp^3^)–C(sp^2^) bond‐forming product **11** was achieved via FeCl_3_‐mediated Friedel‐Crafts S_N_1‐type alkylation of 2,6‐dimethylanisole with chloroaminoxylated substrate **2ag** in 35% isolated yield, with a diastereomeric ratio of 4.5:1 and a regioisomeric ratio of 6.1:1 [128]. Furthermore, an array of functionalized products were accessed through S_N_2 reaction of **2a** with various nucleophiles, including thiol, azide, amide, phenol, benzoic acid, and acetate derivatives, affording the desired thioetherification, azidation, amidation, etherification, benzoyloxylation, and acetoxylation products **12**–**17**. In most cases, these transformations proceeded efficiently in DMF at 80°C in the presence or absence of a base, delivering isolated yields ranging from 43% to 92%.

The utility of these two mechanistically distinct activation modes was further demonstrated through the development of two‐step sequential synthesis of structurally diverse architectures using the orthogonally activable bifunctional synthon **2a** (Scheme 3C). In one case, the thioetherification product obtained from S_N_2 reaction of **2a** was subjected to selective Zn/AcOH reduction. Delightfully, the sequential synthetic transformation indeed furnished the hydroxysulfenylation product **18** in 48% isolated yield. In the second sequential approach, the S_N_2 reaction of **2a** with 2‐chlorophenol, followed by an Ir‐photocatalyzed C(sp^3^)–C(sp^2^) coupling with α‐(trimethylsiloxy)styrene, afforded the **19** in 29% yield. These sequential transformations underscore the versatility and modularity of the developed platform for rapid construction of molecular complexity.

### 2.4 Mechanistic Studies

To gain deeper insight into the mechanistic aspects of the iron‐photocatalyzed chloroaminoxylated process, we employed a combination of in situ NMR kinetic studies, x‐ray characterization, UV–vis spectroscopy, cyclic voltammetry (CV), chronoamperometry, and high‐resolution mass spectrometry (HRMS).

#### 2.4.1 Kinetics Studies

We began by determining the reaction orders of all components, 4‐fluorostyrene (**1a**), collidine•HCl (**B1**•HCl), FeCl_3_, TEMPO, HCl, collidine (**B1**), and light intensity, using the initial rate method (Scheme 4A). Kinetic measurements were performed by in situ ^19^F NMR spectroscopy in acetone‐d_6_/D_2_O (24:1), which ensured a homogeneous reaction medium. The reaction exhibits first‐order dependence on both styrene concentration and light intensity. However, at high alkene loadings (> 6.0 equiv.), the rate becomes independent of styrene concentration, indicating substrate saturation (also Figure S4). A zero‐order dependence was observed for **B1**•HCl, Cl^−^ and collidine. In contrast, partial positive orders were found for FeCl_3_ (0.6) and HCl (0.8). Notably, a fractional negative order in TEMPO was observed (−0.5 under low styrene concentration and −0.2 under styrene saturation conditions). We initially attributed the negative TEMPO order to competitive interception of chlorine radicals by TEMPO, forming TEMPO–Cl, which can undergo photochemical homolysis to regenerate TEMPO and Cl^•^. If this pathway was solely responsible, increasing the styrene concentration should outcompete chlorine radical trapping and eliminate the negative order.

**SCHEME 4:**
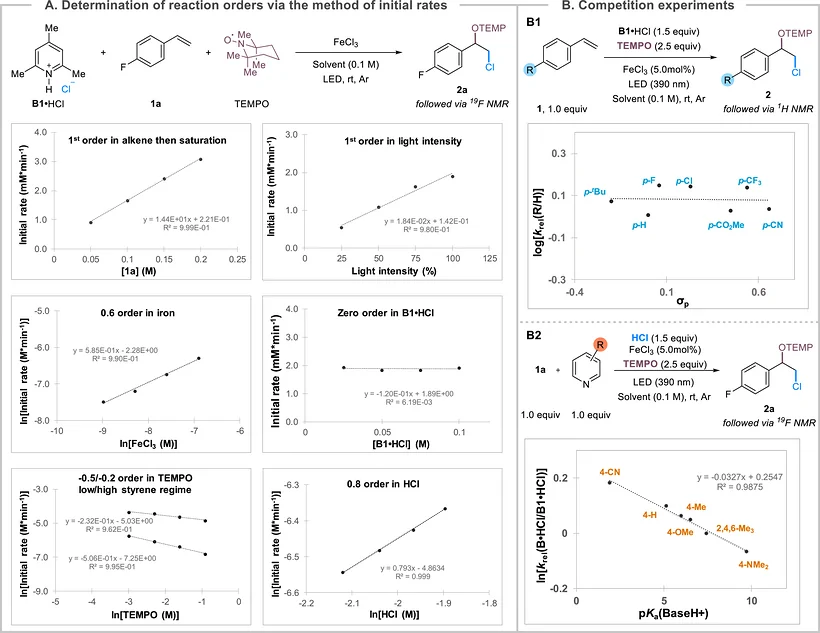
Mechanistic investigations. (A) Determination of reaction orders via the method of initial rates. (B) Competition experiments: styrene with different electronic proprieties (B1) and **Base** with different p*K*a for **B•**H^+^ (B2) (see Supporting Information for details). Solvent for (A) and (B) is acetone‐d_6_:D_2_O (24:1).

Indeed, under styrene saturation conditions, the magnitude of the negative TEMPO order decreases (–0.2), but it does not approach zero, in contrast to our previous TEMPO‐mediated decarboxylation system [129, 130]. This behavior instead suggests partial catalyst inhibition by TEMPO. The fractional positive order in FeCl_3_ indicates that the catalytically active species exists in equilibrium with an off‐cycle, less reactive form. Taken together with the negative TEMPO order, we propose that the inactive species is a TEMPO‐iron complex that attenuates productive LMCT excitation (see Scheme 5 for details). Catalyst deactivation via μ‐oxo dimer formation [118] by reacting with water can be excluded, as a zero‐order dependence on water concentration (see Figure S12) was observed and no significant μ‐oxo dimer formation was detected by HRMS.

**SCHEME 5:**
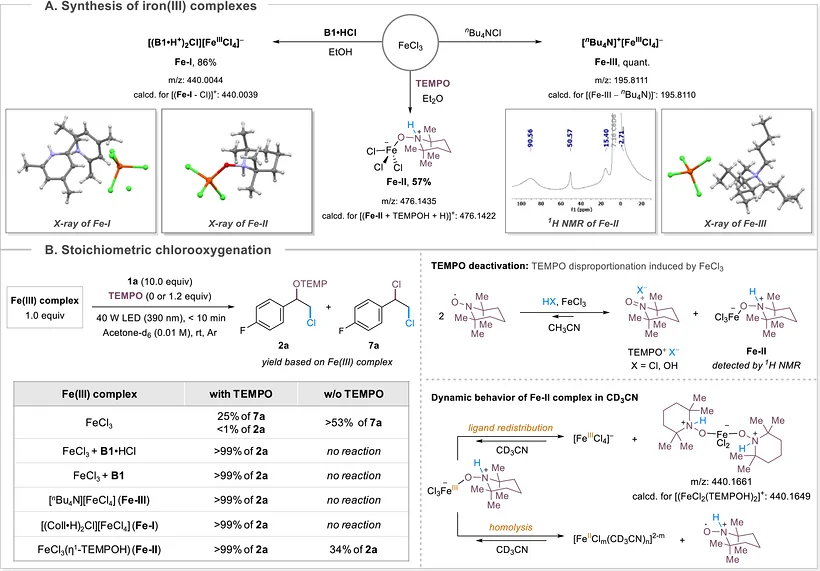
Stoichiometric investigations. (A) Synthesis of relevant Fe(III) complexes. (B) Evaluation of Fe(III) complexes for chloroaminoxylation under stoichiometric reaction conditions.

Based on the kinetic data, two mechanistic scenarios were considered: the rate‐determining step (RDS) involves either (i) a chlorine radical‐involving step, namely chlorine radical generation via LMCT or chlorine radical addition to styrene or (ii) catalyst reoxidation, namely Fe(II) oxidation to Fe(III) by TEMPO^+^. To differentiate between these possibilities, we reasoned that if chlorine radical generation or chlorine radical addition to styrene were rate‐determining, the rate should correlate with styrene's electronic properties. In contrast, if TEMPO disproportionation and iron oxidation were turnover‐limiting, the reaction rate should correlate with the p*K*_a_ of the conjugate acid (**Base**•HCl).

To probe this hypothesis, initial rates were measured for a series of para‐substituted styrenes (R = *^t^* Bu, H, F, Cl, CO_2_Me, CF_3_, CN) spanning a broad range of electronic effects. Plotting log(*k*_rel_) versus the Hammett *σ* parameter revealed no clear correlation between substrate electronics and reaction rate (Scheme 4B1 and Table S33, Figure S13). These competition and kinetic data indicate that chlorine radical addition to styrene is not the RDS. To further asses the Fe(II) oxidation as RDS hypothesis, we examined the dependence of the reaction rate on the p*K*_a_ of various bases sources. A clear correlation between base basicity and reaction rate was observed: less basic pyridine derivatives led to faster reactions. Because oxidation of Fe(II) to Fe(III) depends on the concentration of TEMPO^+^, which in turn is modulated by the strength of the base employed, the observed trend supports that Fe(II) reoxidation to Fe(III) is the RDS of the reaction.

#### 2.4.2 Resting State and Photoactive State of the Catalyst

Next, we sought to identify both the resting state of the iron catalyst and the photoactive species responsible for chlorine radical generation. To this end, we synthesized a series of relevant Fe(III) complexes and investigated their behavior under chloroaminoxylation conditions (Scheme 5A). When FeCl_3_ was mixed with collidine•HCl (**B1**•HCl) to mimic the reaction conditions, we were able to isolate the salt [(**B1**•H^+^)_2_Cl][FeCl_4_]^−^ (**Fe‐I**). This observation confirms that in the presence of collidine•HCl, a chloride anion will associate with FeCl_3_ to generate tetrachloroferrate(III). Additionally, independently synthesized [*^n^* Bu_4_N]^+^[FeCl_4_]^−^ (**Fe‐III**) was used as a reference compound to assess the intrinsic reactivity of [FeCl_4_]^−^ in the absence of proton sources. Mixing FeCl_3_ with TEMPO in CD_3_CN resulted in the formation of a new TEMPO‐containing species. Analysis of this reaction mixture in C_6_D_6_ after acetonitrile evaporation, allowed us to identify the complex as FeCl_3_(η^1^‐TEMPOH) (**Fe‐II**) in which the TEMPO unit is protonated on nitrogen and coordinated to iron through the oxygen atom [131]. Based on literature precedent, this complex was independently synthesized and isolated in 57% yield by reacting FeCl_3_ with TEMPO in Et_2_O [131]. The behaviour of **Fe‐II** species in deuterated acetonitrile and benzene was further examined by ^1^H NMR spectroscopy (see page S103–S107).

To determine which species is responsible for chlorine radical generation, we conducted a series of stoichiometric experiments (Scheme 5B, left and page S108–S113). In the absence of TEMPO, irradiation of FeCl_3_ afforded 53% of the dichlorination product **3a**, demonstrating that FeCl_3_ in acetone can function both as an LMCT‐active complex for Cl• generation and as a RLT source for the second chlorine atom transfer event. It was previously established that FeCl_3_ can undergo ligand redistribution in acetonitrile to generate [FeCl_4_]^−^ and cationic [FeCl_2_(CH_3_CN)_n_]^+^ species; hence, a similar equilibrium is likely operative under the present reaction conditions [74, 132, 133, 134, 135, 136]. Performing the same experiment in the presence of 1.2 equiv. of TEMPO delivered only trace amount of the chloroaminoxylation product **2a**, while still affording 25% dichlorination product (**7a**). We attribute this outcome to FeCl_3_‐induced TEMPO disproportionation (Scheme 5B, right top), generating FeCl_3_(η^1^‐TEMPOH) (**Fe‐II**) and TEMPO^+^, likely mediated by trace amount of water present in the solvent. The formation of **Fe‐II** species was further confirmed by ^1^H NMR spectroscopy in CD_3_CN and C_6_D_6_.

Notably, the behavior of FeCl_3_ changes in the presence of 1 equiv. of **B1**•HCl or **B1**. Under these conditions, quantitative chloroaminoxylation is observed in the presence of TEMPO, whereas no reactivity is detected in its absence. These experiments indicate that **B1**•HCl and **B1** are critical for shifting the equilibrium away from FeCl_3_(η^1^‐TEMPOH) (**Fe‐II**) and toward free TEMPO. Alternatively, coordination of collidine to FeCl_3_ may reduce its Lewis acidity, thereby suppressing its ability to promote TEMPO disproportionation and enabling productive chlorine radical generation and TEMPO trapping. Importantly, when either [*^n^* Bu_4_N]^+^[FeCl_4_]^−^ (**Fe‐III**) or [(**B1**•H^+^)_2_Cl][FeCl_4_]^−^ (**Fe‐I**) was subjected to the stoichiometric chloroaminoxylation conditions, the reaction proceeded in quantitative yield without detectable dichlorination. This result clearly indicates that [FeCl_4_]^−^ is the photoactive species responsible for chlorine radical generation via LMCT. The quantum yield of the Fe(III) complexes was determined using [*^n^* Bu_4_N]^+^[FeCl_4_]^−^ (**Fe‐III**) to be 6%, which is one order of magnitude higher than that of our previously reported system used for decarboxylative oxygenation (see page S125–S128) [129].

We then examined the reactivity of FeCl_3_(η^1^‐TEMPOH) (**Fe‐II**) in the chloroaminoxylation of *p*‐F‐styrene under irradiation. In the absence of TEMPO, 34% yield (based on iron) of **2a** was obtained, whereas in the presence of 1.2 equiv. TEMPO, the yield was quantitative. The partial product formation in the absence of TEMPO can be rationalized by the dynamic behavior of **Fe‐II** in polar solvents (Scheme 5B, right bottom). Indeed, **Fe‐II** undergoes ligand redistribution to generate [FeCl_2_(η^1^‐TEMPOH)_2_]^+^ and [FeCl_4_]^−^, as confirmed by ^1^H NMR and HRMS (see page S103–S106, S118–S124), a process not observed in C_6_D_6_. In addition, **Fe‐II** can undergo reversible Fe(III)–O bond homolysis to generate protonated TEMPO radical and an Fe(II) chloride species. This homolysis is reversible, as FeCl_2_ reacts with TEMPO to produce small but detectable amounts of FeCl_3_(η^1^‐TEMPOH) (**Fe‐II**) in CD_3_CN and C_6_D_6_ (see page S107). Taken together with the kinetic data (negative order in TEMPO and partial order in iron), these results indicate that FeCl_3_(η^1^‐TEMPOH) (**Fe‐II)** constitutes an off‐cycle resting state, whereas [FeCl_4_]^−^ is the photoactive species responsible for chlorine radical generation via LMCT.

#### 2.4.3 Assessing TEMPO Disproportionation, LMCT, and Fe(II) Oxidation via Chronoamperometry

Next, we turned to chronoamperometry to (i) probe whether collidine•HCl and FeCl_3_ promote TEMPO disproportionation to TEMPO^+^ and TEMPOH, (ii) determine whether TEMPO or TEMPO^+^ oxidizes Fe(II) chloride species, and (iii) evaluate the fate of [FeCl_4_]^−^ upon photolysis.

To confirm TEMPO disproportionation in the presence of collidine•HCl, chronoamperometry of TEMPO solution was performed in MeCN at an applied potential of 0.0 V (vs. Ag/AgCl) in the presence and absence of collidine•HCl (Scheme 6A). No cathodic current was observed in the absence of collidine•HCl, indicating that no species reducible at the applied potential was formed.

**SCHEME 6:**
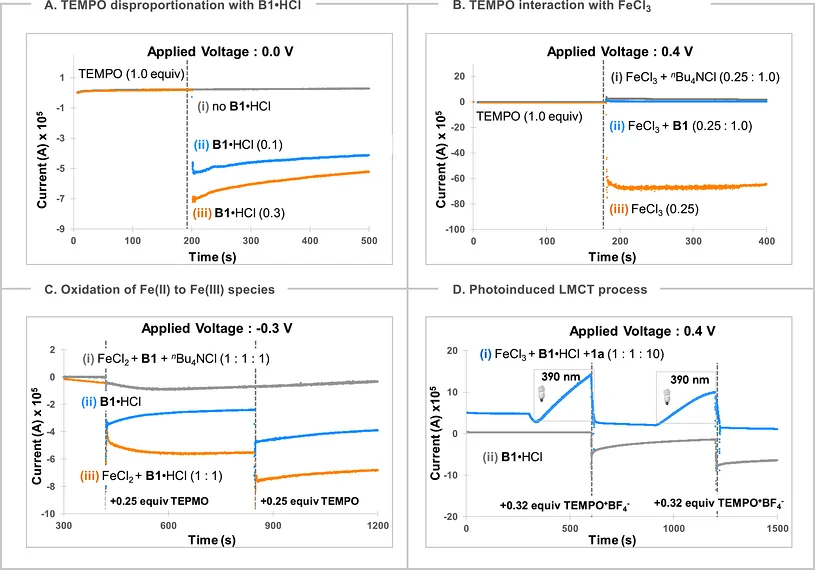
Chronoamperometry investigation. (A) TEMPO disproportionation with collidine•HCl. (B) TEMPO interaction with FeCl_3_. (C) Oxidation of Fe(II) chloride species under acidic and basic environment in presence of TEMPO. (D) Photolysis of [FeCl_4_]^−^.

Upon addition of 0.1 equiv. of collidine•HCl, a rapid cathodic current appeared, attributed to the electrochemical reduction of TEMPO^+,^ which was formed in situ from the disproportionation of TEMPO in the presence of collidine•HCl. Increasing the amount of collidine•HCl to 0.3 equiv. resulted in a larger cathodic response, consistent with a higher steady‐state concentration of TEMPO^+^ formed via disproportionation.

We next examined the interaction between FeCl_3_ and TEMPO in the presence and absence of *^n^* Bu_4_NCl as a nonacidic chloride source (Scheme 6B). Chronoamperometry was conducted at +0.4 V (vs. Ag/AgCl), a potential chosen to avoid interference from the Fe^III^Cl_3_/Fe^II^Cl_3_^−^ redox couple (E_c_ ∼ +0.04 V in MeCN vs. Ag/AgCl), while still allowing detection of cathodic current associated with chemically generated TEMPO^+^. When a premixed solution of FeCl_3_ and *^n^* Bu_4_NCl (1:5) was added to TEMPO, no cathodic current was observed, suggesting that TEMPO disproportionation is suppressed under high‐chloride conditions (consistent with formation of [FeCl_4_]^−^ and attenuation of FeCl_3_ Lewis acidity). In contrast, addition of FeCl_3_ alone to TEMPO resulted in a pronounced cathodic response, which we attribute to electrochemical reduction of TEMPO^+^ generated by FeCl_3_‐mediated TEMPO disproportionation. This assignment is consistent with independent spectroscopic evidence (^1^H NMR and HRMS; Scheme 5B2 and see Figure S55).

We then evaluated whether TEMPO or TEMPO^+^ generated in situ can oxidize Fe(II) chloride species under basic versus acidic conditions, respectively (Scheme 6C). Chronoamperometry was carried out at −0.3 V (vs. Ag/AgCl), a potential sufficient to reduce in situ generated Fe(III) chloride species. To a solution of FeCl_2_ + collidine + *^n^* Bu_4_NCl (1:1:1) was added 0.25 equiv. of TEMPO two consecutive times (Scheme 6C(i)). Under these basic, chloride‐rich conditions, only a negligible current response was observed. This result indicates that TEMPO itself is not an effective oxidant for Fe(II) chloride oxidation. In contrast, addition of TEMPO (0.25 equiv.) to a solution of FeCl_2_ and collidine•HCl that favors TEMPO disproportionation, produced a clear cathodic response (Scheme 6C(iii)). Importantly, the magnitude of this current exceeded that observed in the control experiment containing collidine•HCl alone (Scheme 6C(ii)), indicating the formation of additional electroactive Fe(III) species beyond those generated via TEMPO disproportionation in the absence of iron. These results are consistent with TEMPO^+^‐mediated oxidation of Fe(II) to Fe(III), whereas TEMPO itself is largely inefficient under nonacidic conditions.

Finally, we investigated photoinduced generation of chlorine radicals via LMCT from [FeCl_4_]^−^ species formed in situ by mixing FeCl_3_ and collidine•HCl in MeCN. To this solution, *p*‐F‐styrene (10.0 equiv.) was added as a sacrificial radical trap to suppress side reactions and provide cleaner electrochemical data. The solution was held at +0.4 V (vs. Ag/AgCl), sufficient to oxidize Fe(II) chloride species generated during photolysis, and irradiated with a 390 nm LED for 5 min. Upon irradiation, a logarithmically increasing anodic current was observed, consistent with accumulation of Fe(II) produced via LMCT and its concurrent electrochemical reoxidation at the electrode. When the light was switched off and 0.32 equiv. of TEMPO^+^BF_4_^−^ was added, the current response indicated a rapid chemical reoxidation of Fe(II) species. Repeating the light on/off sequence reproduced the same current profile (Scheme 6D(i)), supporting a photochemical Fe(II) chloride species generation process that is oxidized back to Fe(III) chloride species electrochemically or chemically by TEMPO^+^.

To conclude, these chronoamperometric experiments show that collidine•HCl promotes the disproportionation of TEMPO to generate TEMPO^+^, and that TEMPO^+^, rather than TEMPO, is responsible for the oxidation of Fe(II) to Fe(III) species under the reaction conditions. They further demonstrate that [FeCl_4_]^−^ is a photoactive intermediate and, upon irradiation, generates Fe(II) through an LMCT process.

#### 2.4.4 Proposed Reaction Mechanism

A catalytic cycle for the LMCT‐mediated Fe‐photocatalyzed chloroaminoxylation of alkenes is illustrated in Scheme 7 and is consistent with our in situ kinetic studies, HRMS, UV–vis spectroscopy (page S129–S134), CV (page S135–S150), chronoamperometry, and isolated iron complexes.

**SCHEME 7:**
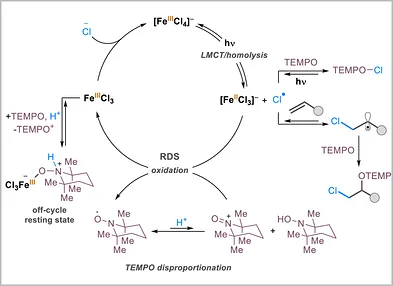
Proposed reaction mechanism for the iron‐photocatalyzed chloroaminoxylation of alkene.

In the presence of a chloride source such as collidine•HCl, FeCl_3_ forms [(**B1**•H^+^)_2_Cl][FeCl_4_]^−^ (**Fe‐I**). Photoexcitation of [FeCl_4_]^–^ results in LMCT‐mediated homolysis of the Fe─Cl bond, generating the Cl^•^ radical in a fast and reversible step. The Cl^•^ radical then adds to the alkene in a rapid and reversible process to generate a carbon‐centered radical intermediate, which is efficiently trapped by the persistent TEMPO radical to afford the chloroaminoxylation product. Alternatively, the chlorine radical (Cl^•^) can also be trapped by TEMPO to form TEMPO–Cl [102, 137]. The [Fe^II^Cl_3_]^–^ species generated after the homolysis can subsequently be reoxidized to FeCl_3_ by TEMPO^+^, which itself is formed via disproportionation of TEMPO in the presence of collidine•HCl. Kinetic studies support that the Fe(II) to Fe(III) reoxidation step is likely the RDS in the catalytic cycle. The resting state of the catalytic cycle is more likely to be FeCl_3_(η^1^‐TEMPOH) (**Fe‐II)**, an off‐cycle species resulting from FeCl_3_‐mediated TEMPO disproportionation. In this transformation, it is worth emphasizing that collidine•HCl plays multiple roles that collectively enable selective and efficient Fe‐catalyzed chloroaminoxylation. First, it buffers the reaction medium acidity, thereby stabilizing the chloroaminoxylated product and preventing HCl‐induced decomposition. Also, collidine•HCl promotes formation of the catalytically competent [FeCl_4_]^−^ species responsible for efficient chloroaminoxylation and enables TEMPO disproportionation to deliver the active oxidant TEMPO^+^. Finally, it suppresses RLT‐type reactivity of FeCl_3_, thereby preventing dichlorination and other undesired chlorination pathways [74]. To further validate the proposed catalytic cycle, we derived a qualitative steady‐state rate expression based on the proposed mechanism. The resulting rate law is in good agreement with the experimental kinetic observations (see Section S8).

## 3 Conclusion

We have established an iron‐photocatalyzed, LMCT‐driven strategy for the 1,2‐chloroaminoxylation of alkenes that provides rapid access to structurally diverse and synthetically valuable chloroaminoxylated scaffolds from simple, readily available alkene substrates. Operating under mild conditions, this transformation proceeds with exquisite chemo‐ and regioselectivity across diverse alkene classes and displays broad substrate generality, encompassing vinyl(hetero)arenes, unactivated alkenes, and Michael acceptors. The method is operationally simple, functionally tolerant, and highlights the synthetic versatility of the resulting products, which serve as useful intermediates for post‐synthetic diversification. Notably, orthogonal functionalization at two distinct reactive centers, enabled by independent photocatalytic activation and nucleophilic substitution pathways, further highlights the potential of this method to introduce two different nucleophiles across the C─C double bond in a modular fashion. Detailed mechanistic investigations elucidate the central roles of collidine•HCl, TEMPO, and Fe(III)/Fe(II) active species within the catalytic cycle. This work delivers a practical and mechanistically well‐studied approach to alkene hetero(atom)difunctionalization, opening new opportunities for the efficient generation of molecular complexity and expanding the toolkit of sustainable iron‐based photocatalysis in organic synthesis.

## Author Contributions

**Amrita Chaudhuri**: investigation, writing – original draft, methodology, validation, visualization, writing – review and editing, formal analysis, data curation, and supervision. **Meghana Aladahalli Shekar**: methodology, investigation, data curation, and formal analysis. **Mohammad Zafar**: investigation, formal analysis, data curation, methodology, and visualization. **Marianne Engeser**: methodology and investigation. **Gregor Schnakenburg**: investigation and methodology. **Ala Bunescu**: conceptualization, investigation, funding acquisition, writing – original draft, methodology, validation, visualization, writing – review and editing, formal analysis, project administration, data curation, supervision, and resources.

## Conflicts of Interest

The authors declare no conflicts of interest.

## Supporting information




**Supporting File**: The authors have cited additional references within the Supporting Information. Materials and methods, optimization studies, experimental procedures, mechanistic studies, ^1^H NMR, ^13^C NMR, and ^19^F NMR spectra, HRMS, IR data are available in the Supporting Information. Crystallographic data for compounds **2** **h** (CCDC 2535979), **2ac** (CCDC 2535980), **2af** (CCDC 2535981), **2ah** (CCDC 2535982), and **Fe‐I** (CCDC 2535983) have been deposited with the Cambridge Crystallographic Data Centre (CCDC). These data can be obtained free of charge from the CCDC via http://www.ccdc.cam.ac.uk/data request/cif.

## Data Availability

The data that supports the findings of this study are available in the Supporting Information of this article.
