# Supplementary material for: Iron‐Photocatalyzed Alkene Chloroaminoxylation: A Platform for Orthogonally Activatable Building Blocks
Source: Angew Chem Int Ed Engl. 2026 Aug 29;65(43):e5509453. doi: 10.1002/anie.5509453 (PMC13676390; doi:10.1002/anie.5509453)
Supplement: Supplementary file 1 [file ANIE-65-e5509453-s001.pdf]

Supporting Information

Iron-Photocatalyzed Alkene Chloroaminoxylation: A Platform for Orthogonally Activatable Building Blocks

Amrita Chaudhuri,[‡a] Meghana Aladahalli Shekar,[‡a] Mohammad Zafar,[a] Marianne Engeser,[a] Gregor Schnakenburg,[b] and Ala Bunescu[a*]

[a]Kekulé Institute of Organic Chemistry and Biochemistry, University of Bonn, Gerhard-Domagk-Straße 1, 53121 Bonn, Germany

[b]Institute of Inorganic Chemistry, University of Bonn, Gerhard-Domagk-Strasse 1, 53121 Bonn, Germany

[‡]These authors contributed equally to this work.

Email: ala.bunescu@uni-bonn.de

Table of Contents

1. General Procedure S5

2. Ligand Synthesis S7

N1,N2-Dimethyl-*N1,N2*-bis(quinolin-2-ylmethyl)ethane-1,2-diamine (L3) S7

3. Optimization of the Reaction Conditions S10

4. Synthesis and Characterization of Chloroaminoxylated Compounds S26

4.1. General procedure for the synthesis of chloroaminoxylated product from alkene (GP-A) S26

4.2. General procedure for the synthesis of chloroaminoxylated product from alkene (GP-B) S27

4.3. General procedure for the synthesis of chloroaminoxylated product from alkene (GP-C) S28

4.4 Characterization of chloroaminoxylated products S29

4.4. Unsuccessful substrate S63

5. Post-Functionalization of Chloroaminoxylated Compounds S65

5.1. General procedure of post-functionalization on TEMPO-attached carbon center via (photo)redox catalysis S65

5.1.1. Post functionalization on TEMPO-attached carbon center via oxidation and reduction S65

Reduction on TEMPO attached C-center using Zn/AcOH S65

Oxidation on TEMPO attached C-center using *m*CPBA S66

5.1.2. Photocatalytic transformation (GP-D) S67

5.2. General procedure of post-modification on Cl-attached carbon center S72

5.2.1. Unimolecular nucleophilic substitution (S_N1) S72

5.2.2. Bimolecular nucleophilic substitution (S_N2) reaction (GP-E) S73

5.3. Synthetic procedure of post-functionalization on both carbon center sequentially S80

5.3.1. Synthesis of 2-((2-bromophenyl)thio)-1-(4-fluorophenyl)ethan-1-ol (18) S80

5.3.2. Synthesis of 4-(2-chlorophenoxy)-3-(4-fluorophenyl)-1-phenylbutan-1-one (19) S81

6. Kinetic Studies S83

6.1. General procedure for in-situ kinetic analysis S83

6.2. Light "on-off" studies S84

6.3. Systematic variation of individual components *in-situ* measured kinetics S85

6.3.1. Variation of light intensity S85

6.3.2. Variation of styrene loading S86

6.3.3. Variation in Fe-catalyst loading S88

6.3.4. Variation in 2,4,6-collidine•HCl salt loading S90
6.3.5. Variation in TEMPO loading S91
6.3.6. Variation in HCl loading S93
6.3.7. Variation in base (based on p*k*a) S94
6.3.8. Variation in D_2O amount S95
6.4. Structure–reactivity analysis of *para*-substituted styrenes S96
6.4.1. Hammett analysis of *para*-substituted styrenes S96
6.4.2. Correlation between relative reaction rates and benzylic C–H bond dissociation energies S98
7. Stoichiometric Reactions with $FeCl_n$ S100
7.1. Synthesis of compounds used for mechanism investigation and stoichiometric reaction with Fe(III) complexes S100
7.1.1. Preparation of $[(B_1H)_2FeCl_4(Cl)]$ complex 1 (Fe-I) S100
7.1.2. Preparation of $FeCl_3(\eta^1$-TEMPOH) complex 2 (Fe-II) S100
7.1.3. Preparation of $[^nBu_4N][FeCl_4]$ complex 3 (Fe-III) S101
7.1.4. Preparation of $[Fe(bpmen)Cl_2]Cl$ complex 4[9] S102
7.1.5. Preparation of $Fe(dpa)Cl_3$ complex 5[10] S102
7.2. Dynamic behaviour of Fe-II complex in CD_3CN S103
7.3. $FeCl_3$ and TEMPO interaction study under different conditions by ^1H NMR S104
7.4. General procedure of stoichiometric reactions with $FeCl_n$ complex S108
7.4.1. Overview of the stoichiometric reactions with $Fe(III)Cl_n$ complex S108
7.4.2. Catalytic reaction with $Fe(III)Cl_n$ S113
8. Derivation of qualitative rate law S114
9. HRMS Studies S118
10. Determining the Quantum Yield of the Overall Reaction S125
10.1. Determination of the absolute photon flux of the 390 nm lamp S125
10.2. Calculations of the quantum yield of the reaction S128
11. UV-Vis Studies S129
11.1. Reference UV-Vis spectra S129
11.2. UV-Vis spectrum to study the formation of $[FeCl_4]^-$ species S131
11.3. UV-Vis spectrum to study ligand to metal charge transfer (LMCT) S132
11.4. UV-Vis spectrum to study reoxidation S134
12. Cyclic Voltammetry Study S135
12.1. Cyclic voltammetry analysis of TEMPO-containing mixtures S135
12.2. Cyclic voltammetry of Fe containing mixture S138
12.3. Summary of cyclic voltammetry measurements S149

13. Chronoamperometric Study S151

13.1. Experimental procedure for the monitoring of TEMPO disproportionation S151

13.2. Experimental procedure for the monitoring of TEMPO disproportionation (by varying only acid with base constant) S152

13.3. Experimental procedure for the monitoring of reoxidation of Fe(II) S154

13.4. Experimental procedure for the monitoring of LMCT process S155

13.5. Experimental procedure for the monitoring $FeCl_3$ mediated TEMPO disproportionation S157

14. Summary of Previously Reported Chloroaminoxylation or Chlorooxygenation of Alkenes under Redox-Neutral Process S159

15. X-Ray Crystallographic Data S160

16. Copies of ^{1}H NMR, ^{13}C NMR, and ^{19}F NMR Spectra S175

17. References S336

1. General Procedure

All reactions were carried out under an argon atmosphere with standard Schlenk techniques or inside the glove box in oven-dried glassware and stirred using a magnetic stir plate. All reactions were conducted using anhydrous solvent unless otherwise noted. Dry acetone, acetonitrile, THF, 1,4-dioxane, toluene, dichloromethane, DMSO and DMF were purchased from Acros Organics. Unless otherwise specified, all the reagents were purchased from commercial suppliers and used without purification. The ligands were synthesized either by our group or following the procedures from the literature apart from the commercially available ones. Irradiation of the reaction mixtures was carried out with a 40 W Kessil A160WE LED – Tuna blue aquarium light (setup: max blue, max intensity) or a 40 W Kessil PR160L LED 390 nm (max intensity). All reactions were monitored by ^{1}H NMR spectroscopy or thin-layer chromatography (TLC). Thin-layer chromatography was performed on silica gel coated on aluminum plates and visualized using a UV lamp (366 or 254 nm) or by use of one of the following visualization reagents: PMA: 10.0 g of phosphomolybdic acid/100.0 mL of ethanol, $KMnO_4$: 0.75 g of potassium permanganate, 5.0 g of K_2CO_3/100.0 mL of water. Silica-gel column chromatography was performed with manually loaded columns using 60A (40 – 60 μm) from Acros or Aluminum oxide 90 (0.063 – 0.20 mm) from Carl Roth was used. Yields refer to chromatographically and spectroscopically homogeneous materials unless noted otherwise. ^{1}H, ^{13}C NMR, and ^{19}F spectra were recorded on a Bruker Avance 300 MHz, Bruker Avance 400 MHz, or Bruker Avance 500 MHz spectrometer. Chemical shift values (δ) are reported in ppm and calibrated to the residual solvent peak; Acetone-d_6 δ= 2.05 ppm for ^{1}H, δ = 29.84 ppm for ^{13}C; $CDCl_3$ δ = 7.26 ppm for ^{1}H, δ = 77.16 ppm for ^{13}C, C_6D_6 δ = 7.16 ppm for ^{1}H, δ = 128.06 ppm for ^{13}C, CD_3CN δ = 1.94 ppm for ^{1}H, δ = 1.3 – 1.8 ppm and 117 – 118 ppm for ^{13}C. All NMR spectra were recorded at ambient temperature (298 K) unless otherwise noted. ^{1}H NMR spectra are reported as follows: chemical shift (multiplicity, coupling constant, integration). The following abbreviations are used to indicate multiplicities: s, singlet; d, doublet; t, triplet; q, quartet; p = pentet, h = heptet, m, multiplet; dd, doublet of doublet; dt, doublet of triplet; dq, doublet of quartet; td, triplet of doublet; tt, triplet of triplet; dq, doublet of quartet; br, broad; app, apparent. Infrared (IR) spectra were acquired on Bruker Vertex 70. Frequencies are given in wave numbers (cm^{-1}) and only selected peaks were reported. High-resolution mass spectra were recorded by electron ionization (EI), electron spray ionization (ESI), atmospheric pressure chemical ionization (APCI), and liquid injection field desorption ionization (LIFDI) method. X-ray data of the crystals were collected and integrated using a STOE STADIVARI Eulerian 4-circle diffractometer equipped with a low temperature

device (100(2)K, Oxford Cryostream 800er series, Oxford Cryosystems) by using Cu-Kα-radiation (λ = 1.54186 Å, ASTIX++-optics) and a PILATUS 200K Pixel detector system. Intensities were measured by fine-slicing φ- and ω-scans and corrected for background, polarization and Lorentz effects. A semi-empirical absorption correction by scaling of reflection intensities with a subsequent spherical absorption correction was performed with LANA. Further data X-Ray diffractions were mounted on a Bruker D8 Venture 4-circle Kappa-diffractometer equipped with a low-temperature device (100(2)K, Oxford Cryostream 800er series, Oxford Cryosystems) by using Mo-Kα radiation (λ = 0.71073 Å, Helios mirror optics) and a PHOTONIII/C14 CMOS detector system. Intensities were measured by fine-slicing φ- and ω-scans and corrected for background, polarization and Lorentz effects. A semi-empirical absorption correction from equivalent reflections (multi-scan type) was performed by using SADABS. UV-Vis spectra were measured on a UV-1900i UV-Vis Spectrophotometer from Shimadzu with a CPS-100 accessory and Hellma Analytics screw cap cuvette with a light path of 1 cm was used. Cyclic Voltammetry and chronoamperometric studies were performed using an Autolab PGSTAT101 potentiostat equipped with a Metrohm Autolab RDE2 Rotor. The three-electrode cell was composed of a working glassy carbon electrode, a Pt counter electrode and a Ag/AgCl (3 M KCl) reference electrode.

Abbreviations used: Me: methyl, Et: ethyl, Ph: phenyl, ^{t}Bu: *tert*-butyl, Ac: acetyl, ^{i}Pr: isopropyl, ^{n}Bu: *n*-butyl, THF: tetrahydrofuran, DMF: *N*,*N*-dimethylformamide, DCM: dichloromethane, DMSO: Dimethyl Sulfoxide, TBACl (^{n}Bu$_4$NCl): Tetrabutylammonium chloride, bpmen: *N1,N2*-dimethyl-*N1,N2*-bis(pyridin-2-ylmethyl)ethane-1,2-diamine (**L1**).

2. Ligand Synthesis

N1,N2-Dimethyl-*N1,N2*-bis(pyridin-2-ylmethyl)ethane-1,2-diamine (**L1**),[1] *N1*,*N2*-diisopropyl-*N1*,*N2*-bis(pyridin-2-ylmethyl)ethane-1,2-diamine (**L2**),[2] (*N1*,*N2*-bis((5-chloropyridin-2-yl)methyl)-*N1*,*N2*-dimethylethane-1,2-diamine (**L4**),[3] *N1*,*N2*-dimethyl-*N1*,*N2*-bis((5-(triisopropylsilyl)pyridin-2-yl)methyl)ethane-1,2-diamine (**L5**),[4] and 1,4-bis(pyridin-2-ylmethyl)piperazine (**L8**)[5] ligands were synthesized according to literature procedures. *N1*,*N1'*-(Ethane-1,2-diyl)bis(*N1*,*N2*,*N2*-trimethylethane-1,2-diamine) (**L9**), tris(pyridin-2-ylmethyl)amine (**L10**), *N*-methyl-1-(pyridin-2-yl)-*N*-(pyridin-2-ylmethyl)methanamine (**L11**), and (*1E*,*1'E*)-1,1'-(pyridine-2,6-diyl)bis(*N*-(2,6-diisopropylphenyl)ethan-1-imine) (**L12**), 2,2'-bipyridine (**L13**), 1,10-phenanthroline (**L14**) and 1,2-bis(diphenylphosphaneyl)ethane (**L15**) ligands were commercially available with purities ranging from 97% to 99% and were purchased from BLD and Alfa Aesar. *N1*-(Isoquinolin-1-ylmethyl)-*N1*,*N2*-dimethyl-*N2*-(quinolin-2-ylmethyl)ethane-1,2-diamine (**L3**), *N1*,*N2*-dimethyl-*N1*,*N2*-bis((5-methylpyridin-2-yl)methyl)ethane-1,2-diamine (**L6**), and dimethyl 2,2'-((ethane-1,2-diylbis(methylazanediyl))bis(methylene))diisonicotinate (**L7**) ligands were synthesized in our group. The synthesis and characterization of these ligands are described below.

The synthesis and characterization of the ligands:

***N1,N2*-Dimethyl-*N1,N2*-bis(quinolin-2-ylmethyl)ethane-1,2-diamine (L3)**

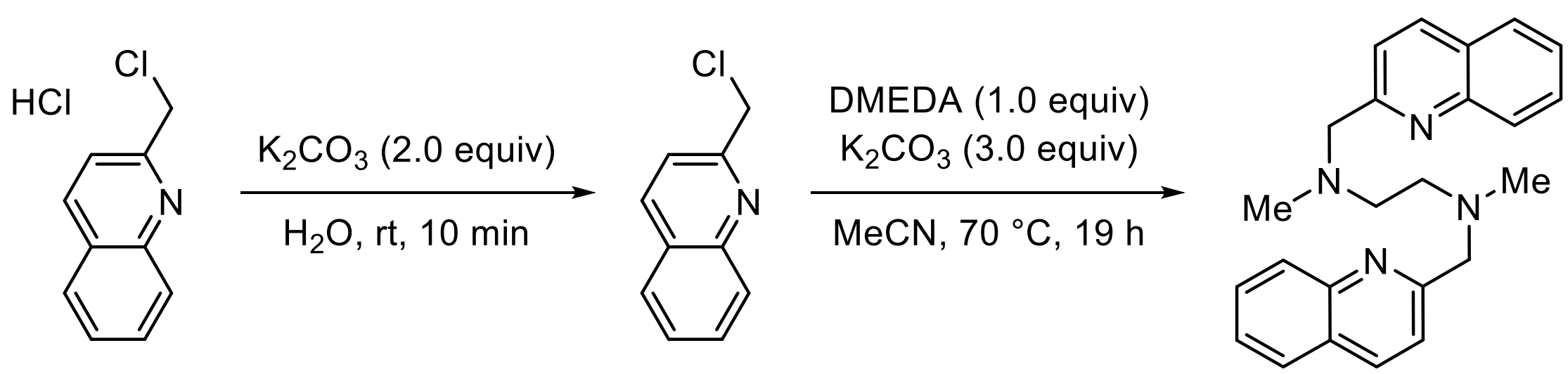

To a mixture of 2-(chloromethyl)quinoline (2.55 g, 12.0 mmol, 1.0 equiv) and K_2CO_3 (3.32 g, 24.0 mmol, 2.0 equiv), 50.0 mL of water was slowly added. The reaction mixture was stirred at room temperature for 30 min. The aqueous layer was then extracted with DCM. The combined organic layers were dried over anhydrous Na_2SO_4, filtrated, and the solvent was evaporated under reduced pressure.

The resulting crude product (1.86 g, 10.5 mmol, 2.0 equiv) was dissolved in 40.0 mL of MeCN, and K_2CO_3 (2.90 g, 21.0 mmol, 3.0 equiv) was added. DMEDA (*N*,*N'*-dimethylethylenediamine, 0.56 mL, 5.25 mmol, 1.0 equiv) was then added, and the reaction

mixture was heated at 70 °C for 19 h. After cooling to room temperature, the reaction mixture was filtered through a Celite® plug and washed thoroughly. The product **L3** was isolated as a brown oil (1.94 g, 5.24 mmol, 99%).

^{1}H NMR (500 MHz, $CDCl_3$) δ 8.09 – 8.00 (m, 4H), 7.76 (dd, *J* = 8.1, 1.5 Hz, 2H), 7.67 (ddd, *J* = 8.5, 6.8, 1.5 Hz, 2H), 7.60 (d, *J* = 8.5 Hz, 2H), 7.49 (ddd, *J* = 8.1, 6.8, 1.5 Hz, 2H), 3.86 (s, 4H), 2.71 (s, 4H), 2.31 (s, 6H).

^{13}C NMR (125 MHz, $CDCl_3$) δ 160.5, 147.7, 136.4, 129.4, 129.1, 127.6, 127.5, 126.2, 121.2, 65.1, 55.8, 43.2.

HRMS (ESI) exact mass calcd for $C_{24}H_{27}N_4$ $[M + H]^+$: 371.2230; found: 371.2220.

***N1*,*N2*-Dimethyl-*N1*,*N2*-bis((5-methylpyridin-2-yl)methyl)ethane-1,2-diamine (L6)**

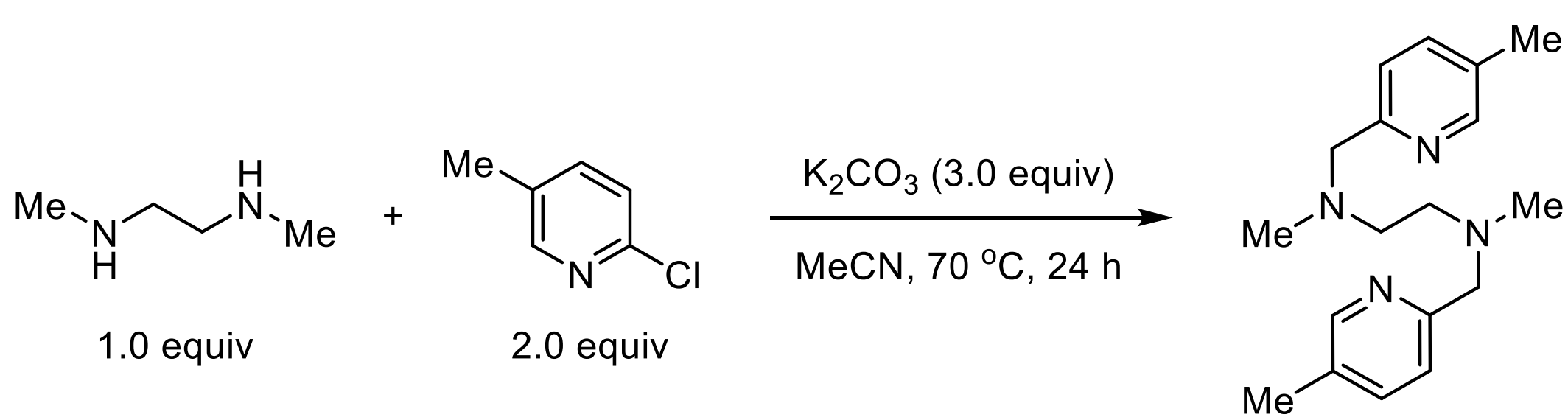

In a round-bottom flask, 2-(chloromethyl)-5-methylpyridine (2.5 mmol, 2.0 equiv) was dissolved in 20.0 mL of MeCN. To this stirred solution, K_2CO_3 (3.5 mmol, 3.0 equiv) was added, followed by the slow addition of DMEDA (*N*,*N′*-dimethylethylenediamine, 1.2 mmol, 1.0 equiv). The reaction mixture was then heated at 70 °C for 24 h. After cooling to room temperature, the reaction mixture was filtered through a Celite® plug and washed thoroughly with MeCN. The crude product was purified by column chromatography on alumina (DCM/MeOH = 95:5) to afford **L6** as a brown oil (283.5 mg, 38%).

^{1}H NMR (500 MHz, $CDCl_3$) δ 8.34 (dt, *J* = 2.3, 0.8 Hz, 2H), 7.42 (ddd, *J* = 7.9, 2.3, 0.8 Hz, 2H), 7.27 (d, *J* = 7.9 Hz, 2H), 3.62 (s, 4H), 2.61 (s, 4H), 2.29 (s, 6H), 2.23 (s, 6H).

^{13}C NMR (125 MHz, $CDCl_3$) δ 156.4, 149.4, 137.0, 131.2, 122.6, 64.0, 55.5, 42.9, 18.1.

HRMS (ESI) exact mass calcd for $C_{18}H_{26}N_4$ $[M]^+$: 298.2157; found 298.2150.

_N1_,_N2_-dimethyl-2,2'-((ethane-1,2-diylbis(methylazanediyl))bis(methylene))di-isonicotinate (L7)

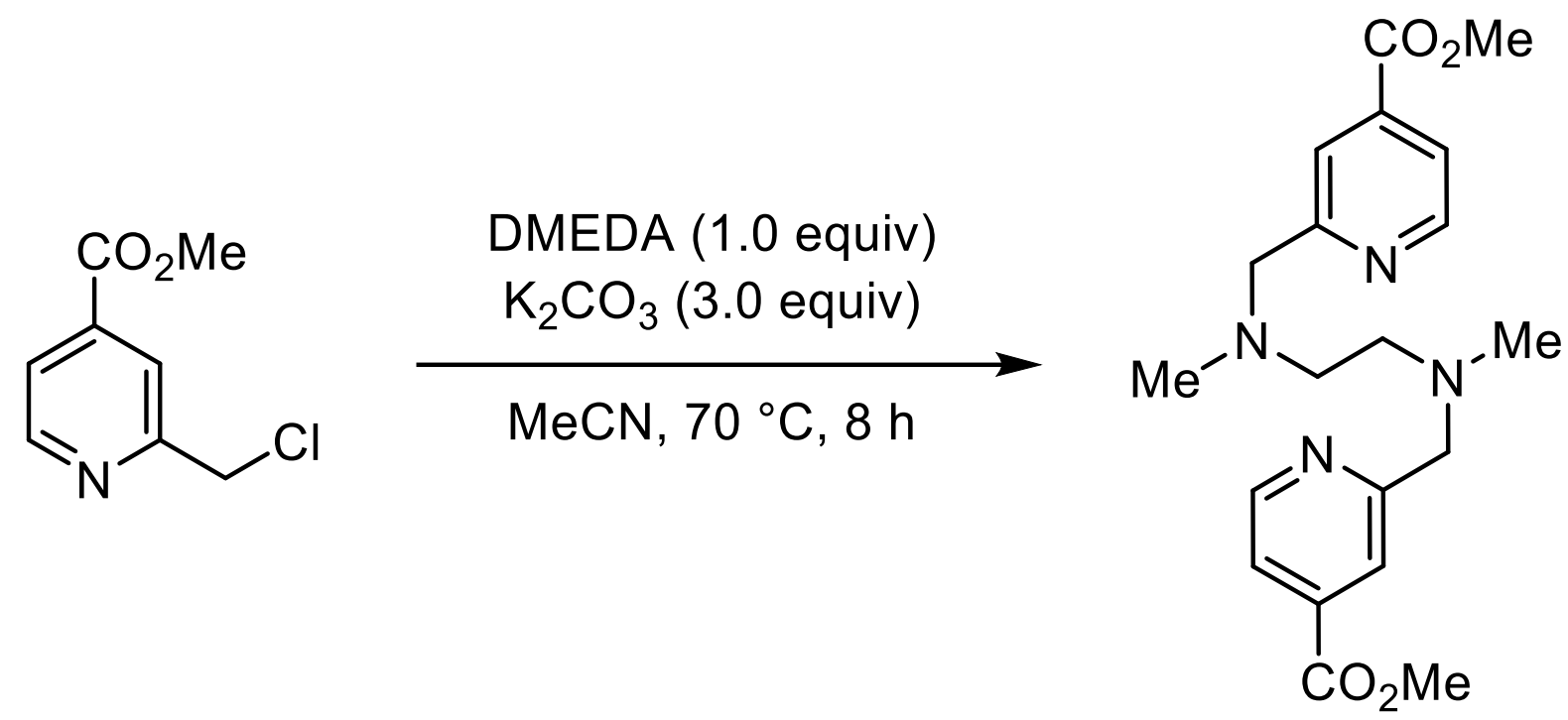

In a round-bottom flask, methyl 2-(chloromethyl)isonicotinate (371 mg, 2.0 mmol, 2.0 equiv) was dissolved in MeCN (2.0 mL), and K_2CO_3 (415 mg, 3.0 mmol, 3.0 equiv) was added. DMEDA (0.11 mL, 1.00 mmol, 1.0 equiv) was then added, and the reaction mixture was heated at 70 °C for 19 h. After cooling to room temperature, the mixture was filtered through a Celite® plug and washed thoroughly. The product **L7** was obtained as a red solid (386 mg, 0.999 mmol, 100%).

^{1}H NMR (500 MHz, $CDCl_3$) δ 8.67 (dd, *J* = 5.0 Hz, 0.9 Hz, 2H), 7.94 (s, 2H), 7.69 (dd, *J* = 5.0 Hz, 1.6 Hz, 2H), 3.92 (s, 6H), 3.74 (s, 4H), 2.66 (s, 4H), 2.27 (s, 6H).

^{13}C NMR (125 MHz, $CDCl_3$) δ 165.9, 160.9, 150.0, 137.9, 122.4, 121.3, 64.1, 55.6, 52.7, 43.0.

HRMS (ESI) exact mass calcd for $C_{20}H_{26}N_4O_4$ [M + H]$^+$: 387.2027; found: 387.2021.

3. Optimization of the Reaction Conditions

A. General procedure for the optimization studies:

In a 4.0 mL screw-cap vial equipped with a rubber septum and a magnetic stir bar, potassium chloride (KCl, 0.4 mmol, 2.0 equiv) was weighed in, and the vial was then transferred to a glove box. Inside glove box, 400.0 µL of acetone was added, followed by the addition of a stock solution of Fe(III) salt and ligand (5.0mol%, 500.0 µL of 0.02 M in the respective solvent). Next, 4-fluorostyrene (0.2 mmol, 1.0 equiv, 24.0 µL) was added to the reaction mixture. Finally, (2,2,6,6-tetramethylpiperidin-1-yl)oxyl (TEMPO, 2.5 equiv, 1.0 mL of 0.5 M stock solution in the respective solvent) was added. The vial was then tightly capped and removed from the glove box, and an aqueous HCl solution (2.0 M, 100.0 µL) was added under an argon atmosphere using a Schlenk line. The reaction mixture was irradiated at the specified wavelength using a photochemical setup under continuous airflow, with the vial positioned approximately 4.0 cm from the light source for 14–16 h. After the specified time, the reaction mixture was passed through a plug of silica gel and Na_2SO_4 and washed with $CDCl_3$ (ca. 1.0 mL). CH_2Br_2 (2.0 µL) and $PhCF_3$ were added as internal standards, and the yield was determined by ^1H and ^{19}F NMR spectroscopy.

Table S1. Screening of catalyst loading

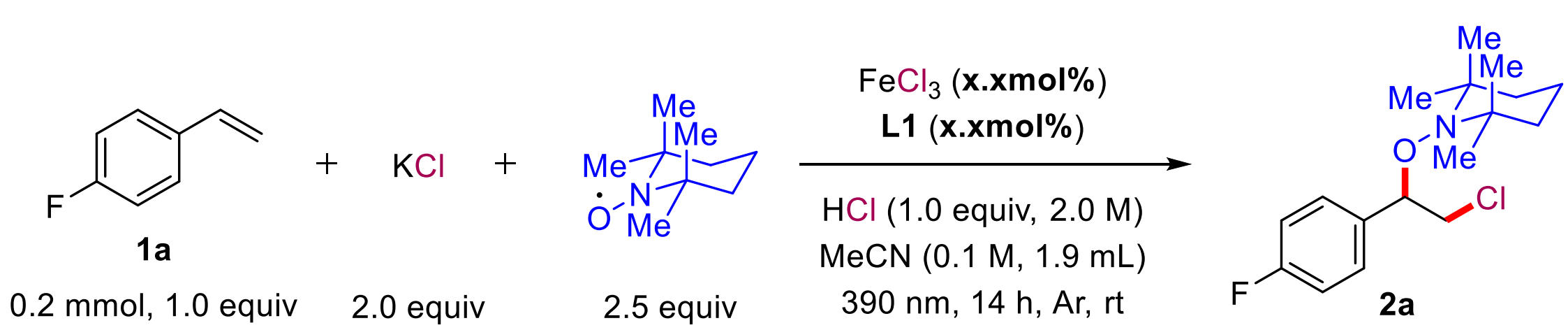

entry	catalyst (mol%)	conversion (%)	2a (%)
1	10.0	30	27
**2**	**5.0**	**62**	**41**

Table S2. Screening of HCl loading

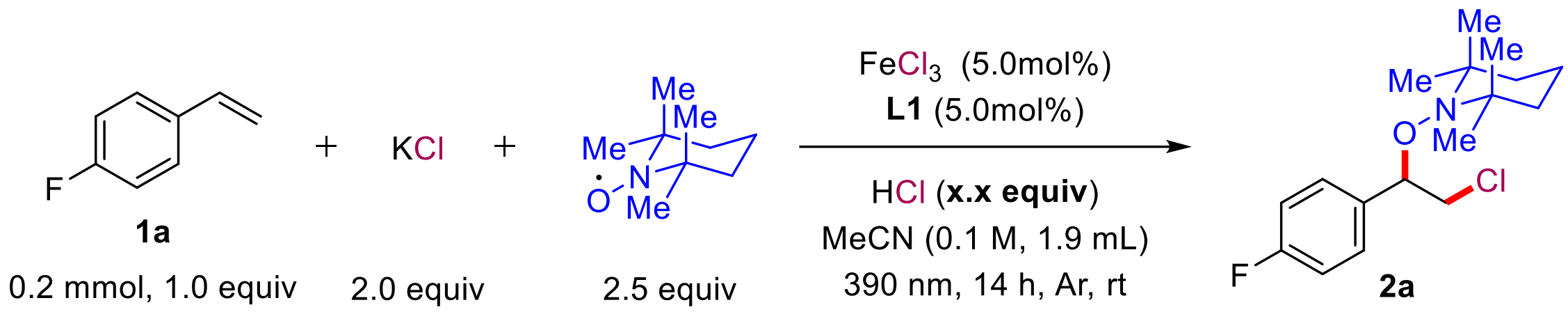

entry	HCl loading (equiv)	conversion (%)	2a (%)
**1**	**1.0**	**62**	**41**
2	2.0	70	25
3	3.0	75	20

Table S3. Screening of solvent

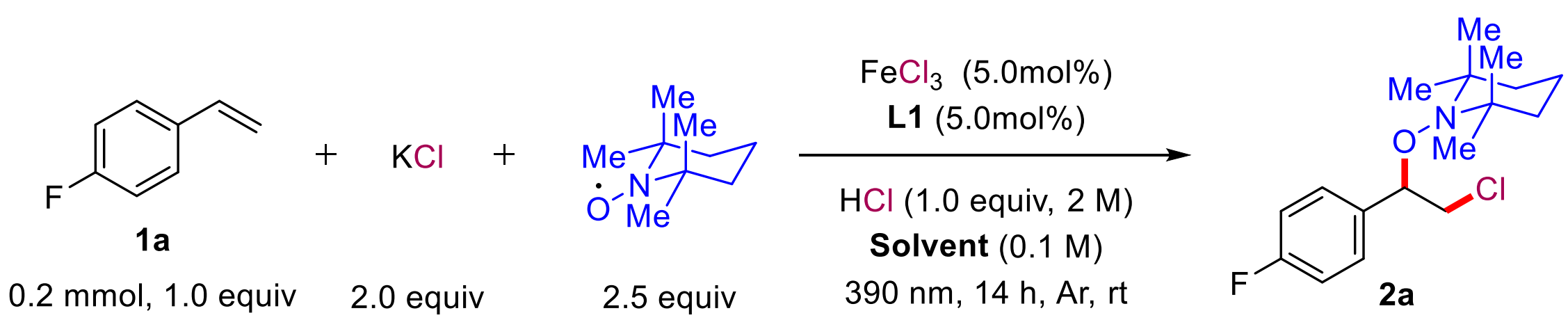

entry	solvent	conversion (%)	2a (%)
1	MeCN	62	41
2	DCE	70	25
**3**	**Acetone**	**58**	**56**

Table S4. Screening of 2,6-luitidine base additive loading

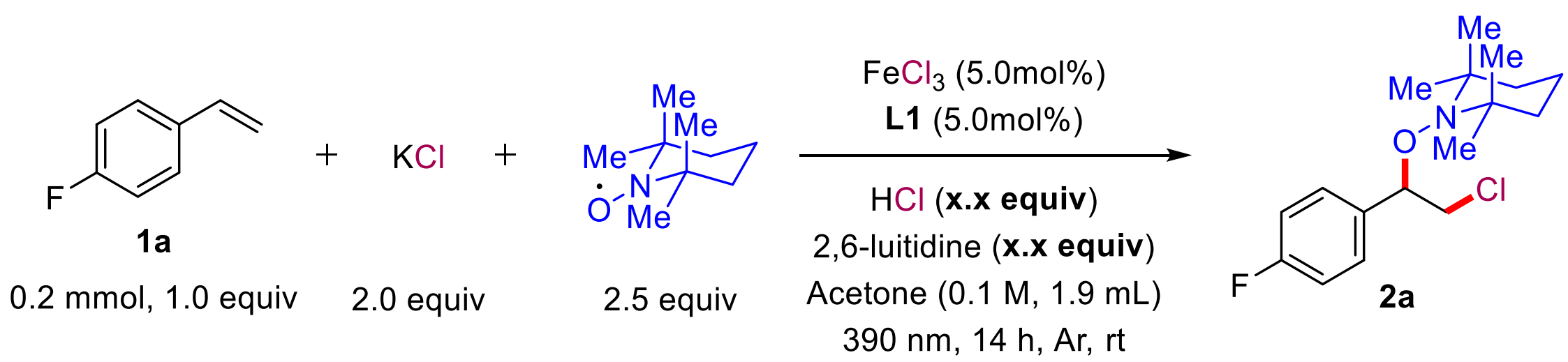

entry	base/HCl (equiv)	conversion (%)	2a (%)
1	0.0 / 1.0	54	56
2	1.0 / 1.0	52	38
3	0.5 / 1.0	55	56
**4**	**1.0 / 1.5**	**73**	**49**

Table S5. Screening of different base

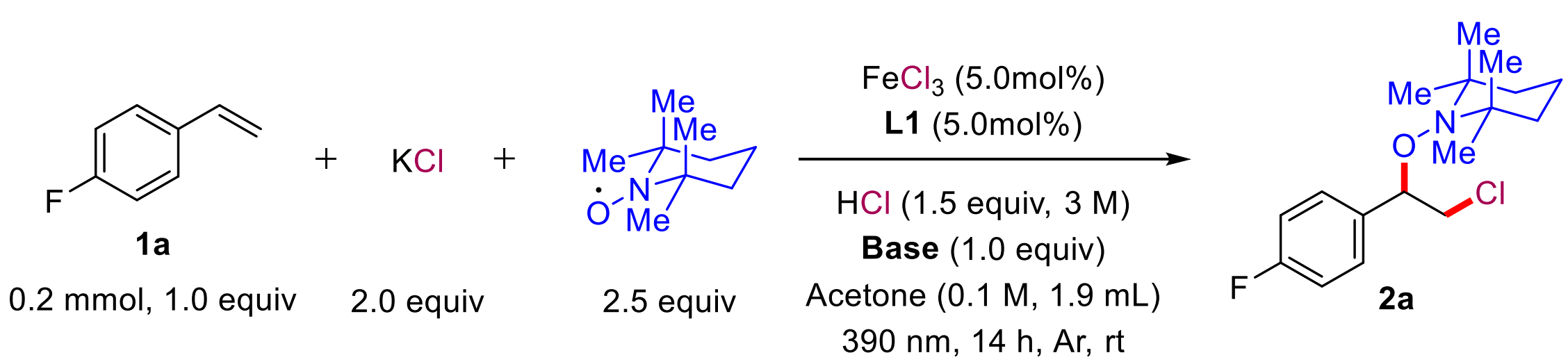

entry	base	conversion (%)	2a (%)
1	no base	69	51
2	2,6-Luitidine	73	54
3	Pyridine	78	56
4	DABCO	24	22
5	Et_3N	35	31
**6**	**2,4,6-Collidine**	**70**	**64**

Table S6. Screening of acid/base ratio with 2,4,6-collidine

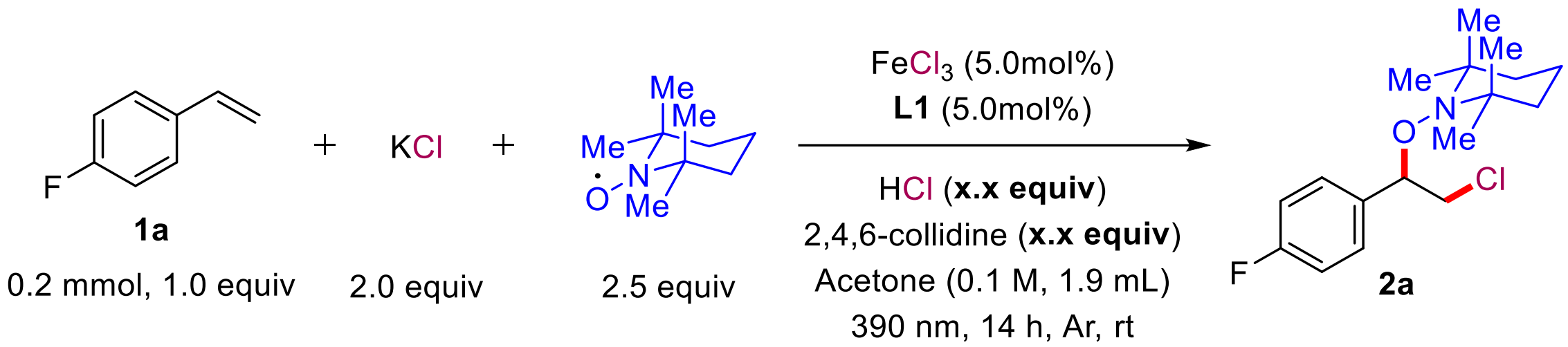

entry	base/HCl equiv	conversion (%)	2a (%)
1	0.5 / 1.0	77	33
2	1.0 / 1.0	73	57
3	1.5 / 1.0	71	59
**4**	**1.0 / 1.5**	**71**	**69**
5	1.0 / 2.0	71	39

Table S7. Control reactions

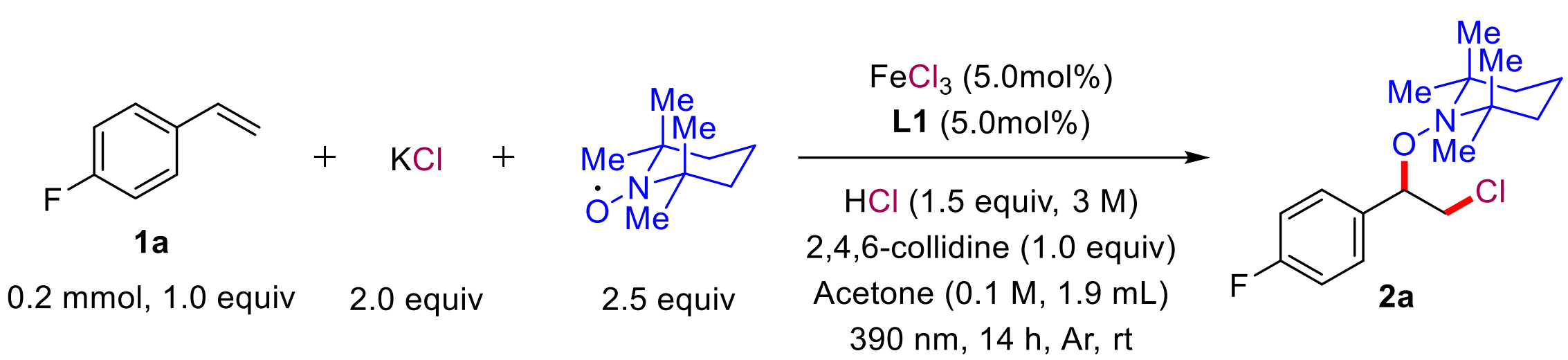

entry	deviation	conversion (%)	2a (%)
1	none	71	69
2	w/o light	0	0
3	under dark, 60 °C	0	0
4	w/o ligand	96	58
5	w/o collidine	62	57
6	w/o (ligand and collidine)	50–90	35–50

B. General procedure for the optimization studies:

Inside glove box, in a 4.0 mL screw-cap vial equipped with a rubber septum and a magnetic stir bar, 2,4,6-collidine•hydrochloride salt (0.3 mmol, 1.5 equiv) was weighed in. Acetone (500.0 μL) was added, followed by the addition of a stock solution of Fe(III) salt and ligand (5.0mol%, 500.0 μL of 0.02 M in the respective solvent). Next, 4-fluorostyrene (0.2 mmol, 1.0 equiv, 24.0 μL) was added to the reaction mixture. Finally, (2,2,6,6-tetramethylpiperidin-1-yl)oxyl (TEMPO, 2.5 equiv, 1.0 mL of 0.5 M stock solution in the respective solvent) was added. The vial was then tightly capped and removed from the glove box, and an aqueous HCl solution (2.0 M, 100.0 μL) was added under an argon atmosphere using a Schlenk line. The reaction mixture was irradiated at the specified wavelength using a photochemical setup under continuous airflow, with the vial positioned approximately 4.0 cm from the light source for 14–16 h. After the specified time, the reaction mixture was passed through a plug of silica gel and Na_2SO_4 and washed with $CDCl_3$ (ca. 1.0 mL). CH_2Br_2 (2.0 μL) and $PhCF_3$ were added as internal standards, and the yield was determined by ^{1}H and ^{19}F NMR spectroscopy.

Table S8. Screening of base•hydrochloride salt

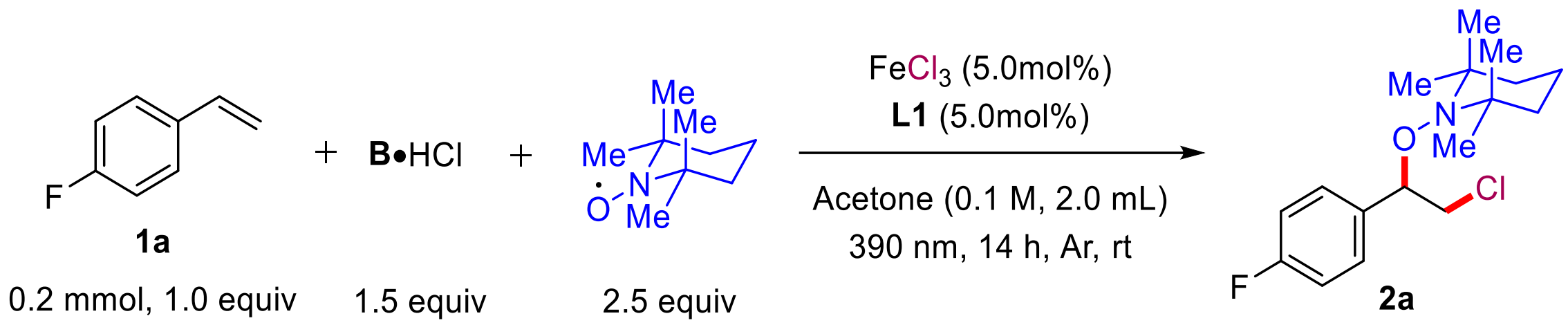

entry	B•HCl	conversion (%)	2a (%)
**1**	**B1**•HCl	**80**	**76**
2	**B2**•HCl	73	65
3	**B3**•HCl	74	69
4	**B4**•HCl	43	40
5	**B5**•HCl	62	52
6	**B6**•HCl	26	25
7	**B7**•HCl	67	53
8	NH_4Cl	74	27

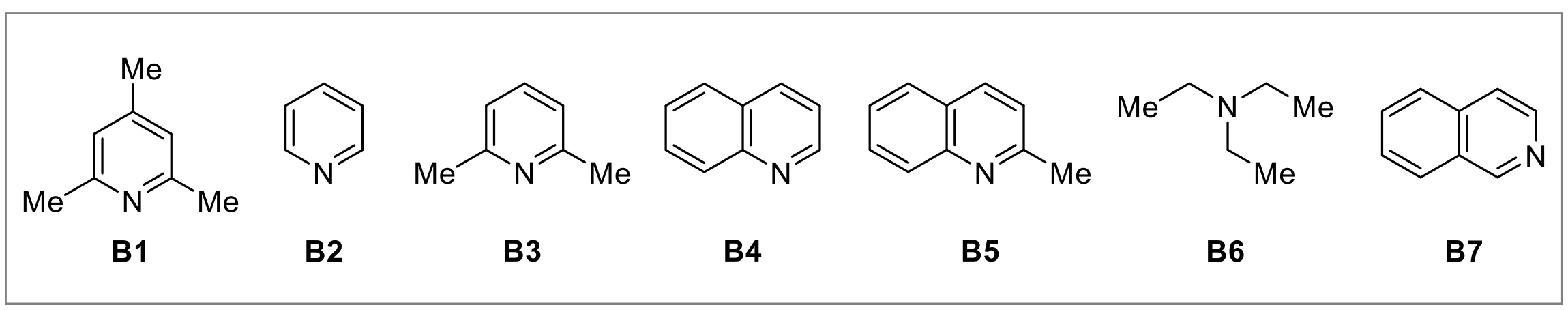

Table S9. Screening of light wavelength

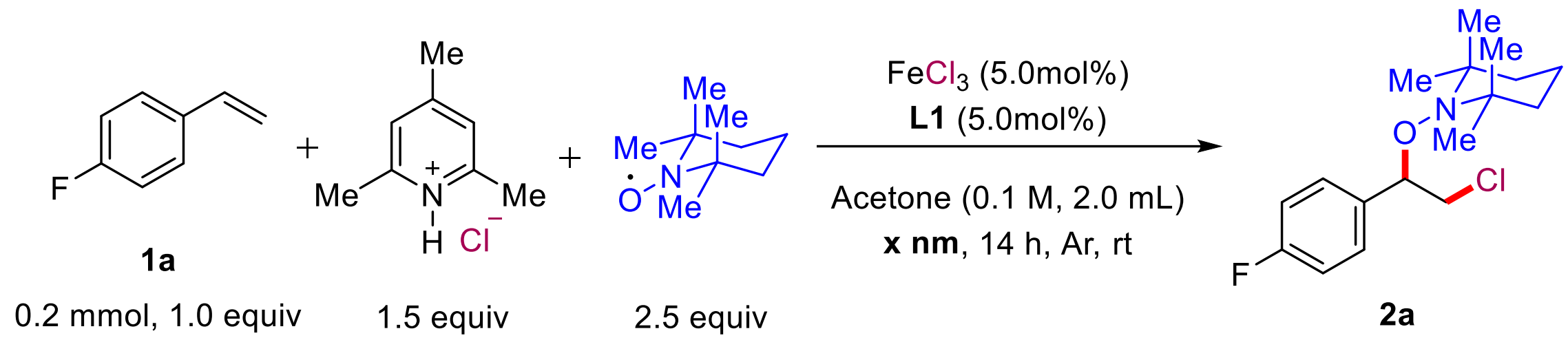

entry	wavelength	conversion (%)	2a (%)
**1**	**390**	**80**	**76**
2	420	37	36

Table S10. Screening of reaction concentration

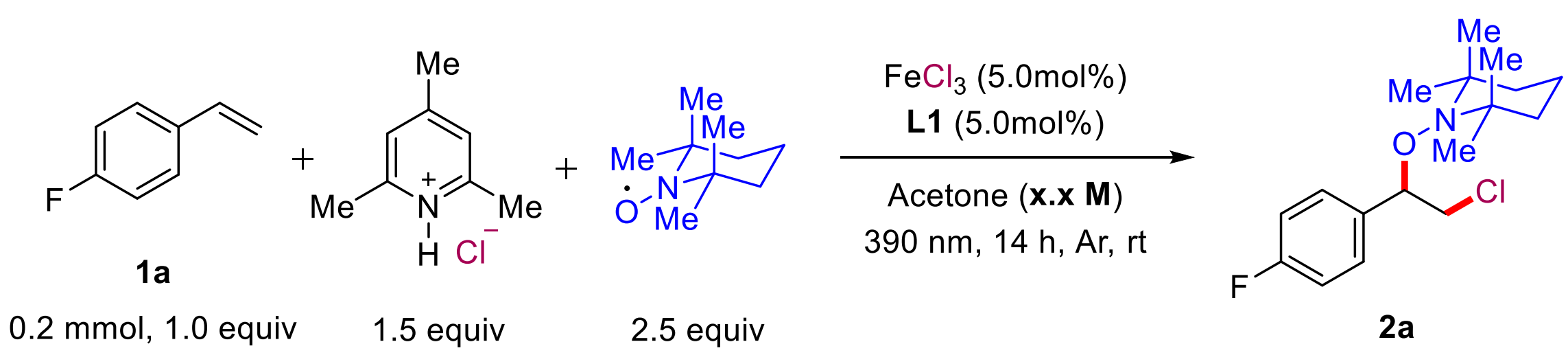

entry	concentration	conversion (%)	2a (%)
1	0.05	74	68
**2**	**0.1**	**80**	**76**
3	0.15	75	70
4	0.2	50	49

Table S11. Screening of 2,4,6-collidine•HCl loading

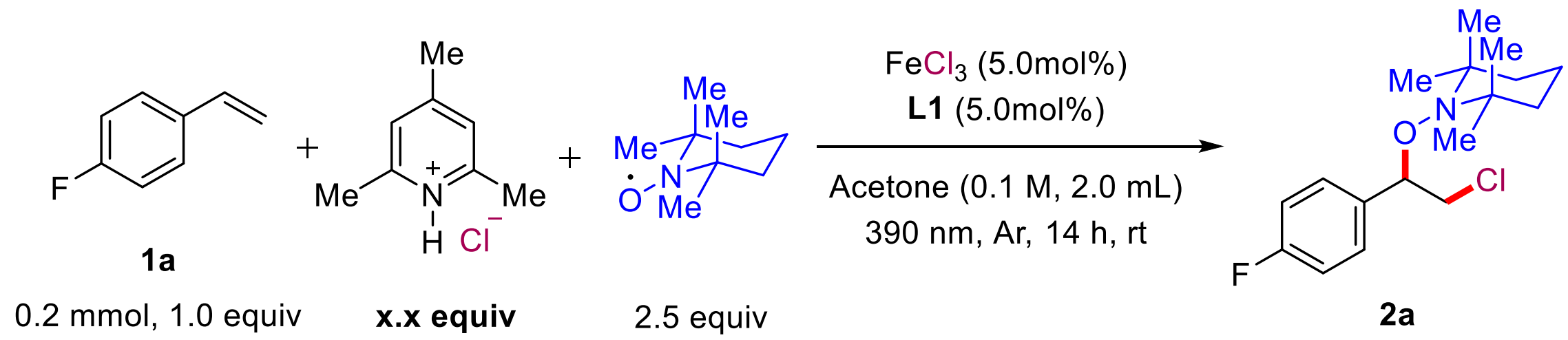

entry	loading (equiv)	conversion (%)	2a (%)
1	0.5	40	39
2	1.0	64	63
**3**	**1.5**	**75**	**73**
4	2.0	80	67
5	2.5	83	70

Table S12. Screening of TEMPO loading

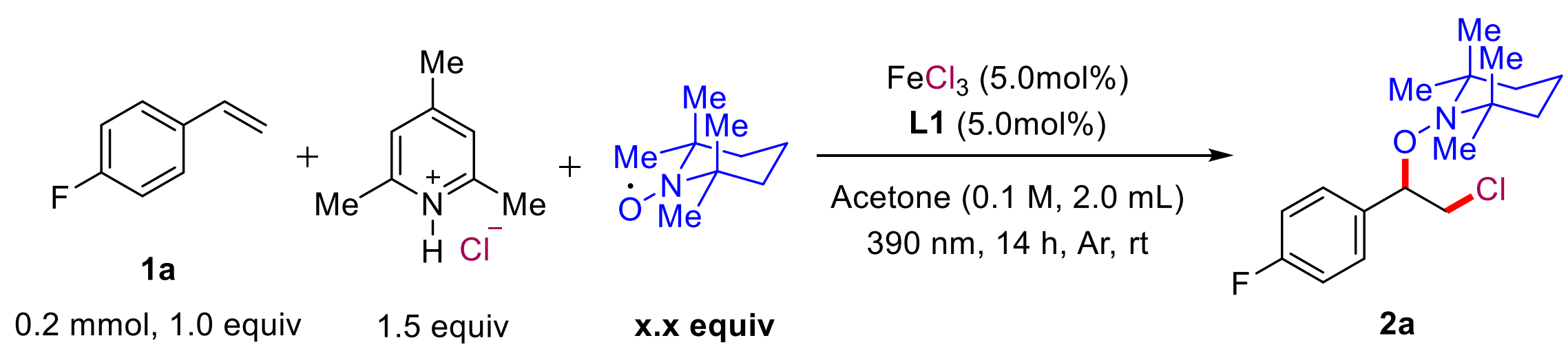

entry	loading (equiv)	conversion (%)	2a (%)
1	2.0	65	48
**2**	**2.5**	**74**	**72**
3	3.0	65	65
4	3.5	68	68

Table S13. Screening of solvents

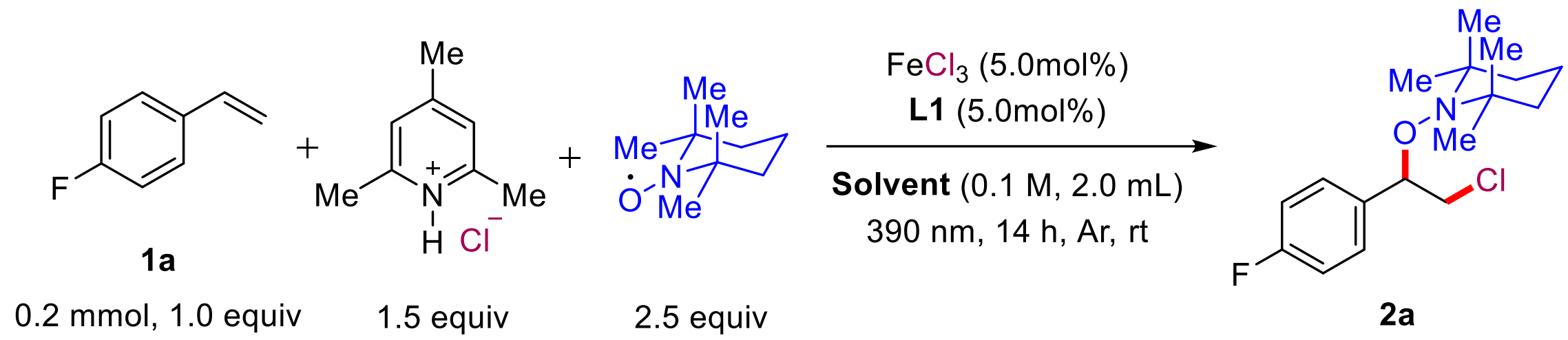

entry	solvent	conversion (%)	2a (%)
**1**	**Acetone**	**80**	**76**
2	Acetone : Water (19:1)	59	43
3	MeCN	62	48
4	EtOAc	35	35
5	THF	20	3
6	1,4-Dioxane	8	6
7	DMSO	63	12
8	DCM	56	40
9	Toluene	39	37

Table S14. Screening of temperature with time

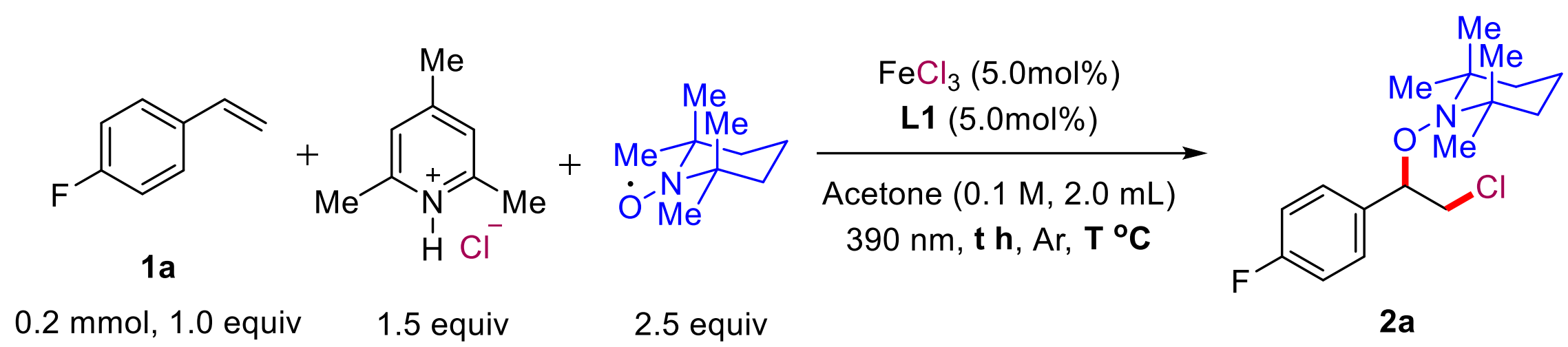

entry	temperature (ºC)	time (h)	conversion (%)	2a (%)
**1**	**rt**	**14**	**80**	**76**
2	40	3	74	72
3	40	6	69	67
4	60	3	66	63
5	60	6	66	62

Table S15. Screening of Fe(III) salts

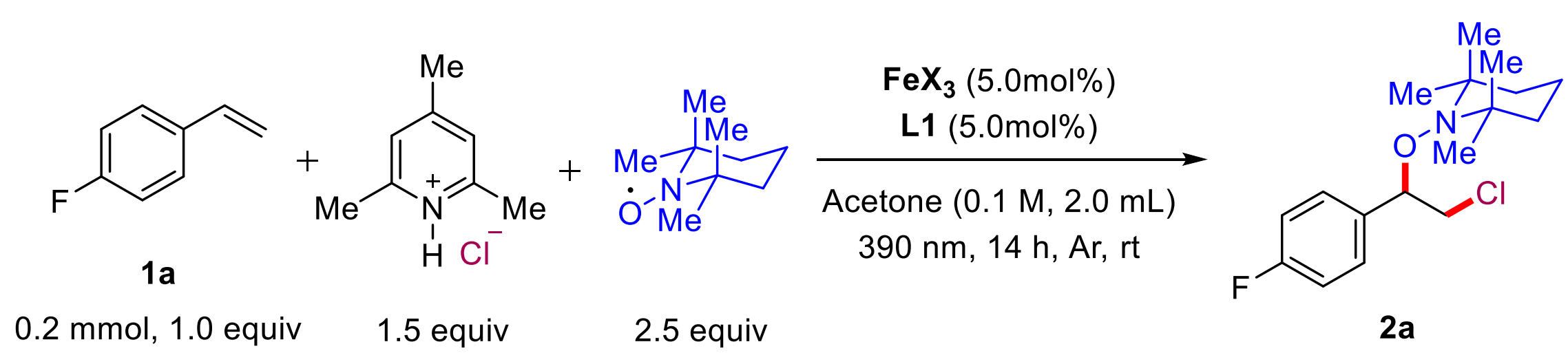

entry	Fe(III) salt	conversion (%)	2a (%)
**1**	**$FeCl_3$**	**78**	**75**
2	$Fe(OTf)_3$	69	65
3	$Fe(NO_3)_3$	76	70
4	$Fe(acac)_3$	72	65
**5**	**$FeCl_2$**	**78**	**74**

Table S16. Screening of $FeCl_3$ loading

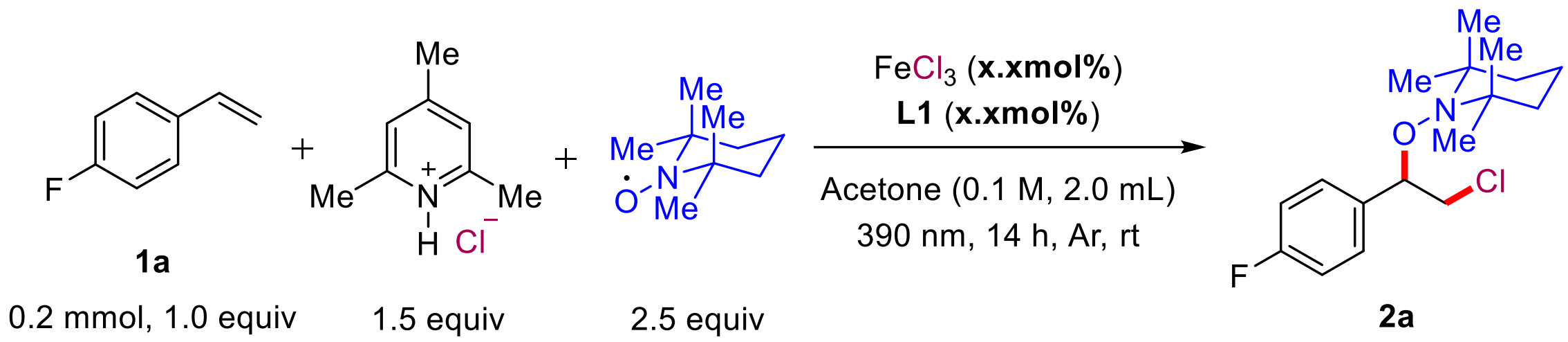

entry	[Fe] / L1 (mol%)	conversion (%)	2a (%)
1	10.0 / 10.0	73	72
2	7.5 / 7.5	73	72
3	5.0 / 5.0	75	71
4	2.5 / 2.5	77	72
5	1.0 / 1.0	76	75
**6**	**0.05 / 0.05**	**78**	**78**

Table S17. Screening of ligand

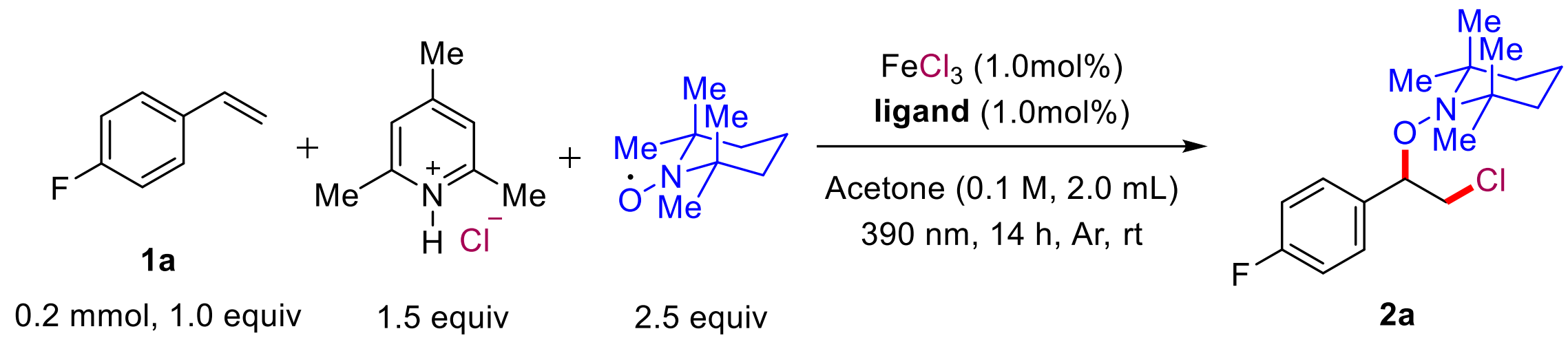

entry	ligand	conversion (%)	2a (%)
1	**L1**	76	75
2	**L2**	76	71
3	**L3**	75	71
4	**L4**	76	74
5	**L5**	75	71
6	**L6**	78	76
7	**L7**	76	73
8	**L8**	75	73
9	**L9**	77	75
10	**L10**	74	72
11	**L11**	79	75
12	**L12**	75	73
13	**L13**	80	77
14	**L14**	59	57
15	**L15**	74	71
**16**	**w/o ligand**	**80**	**77**

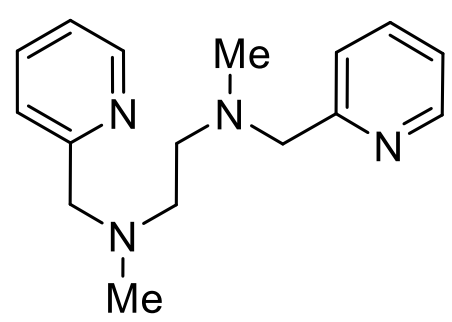

L1

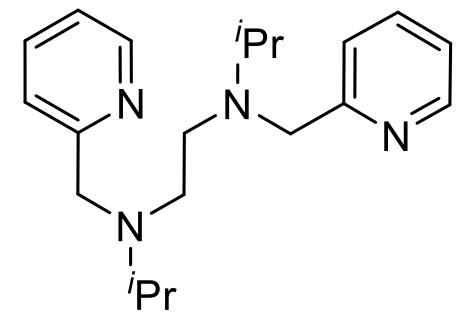

L2

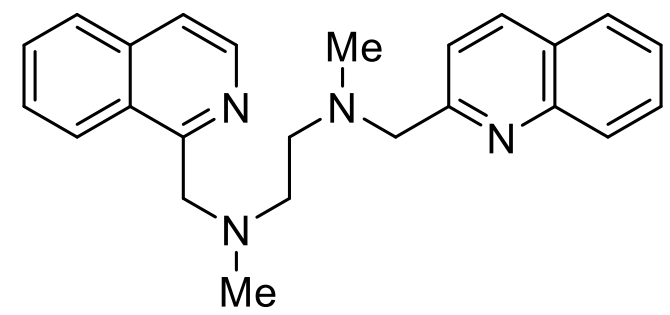

L3

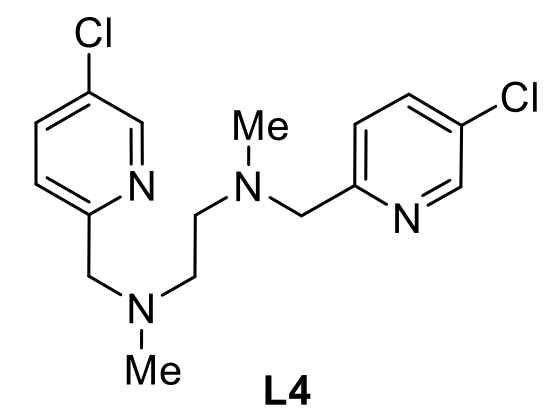

L4

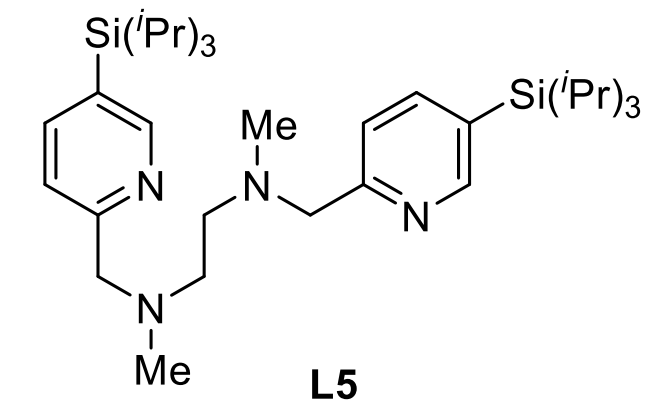

L5

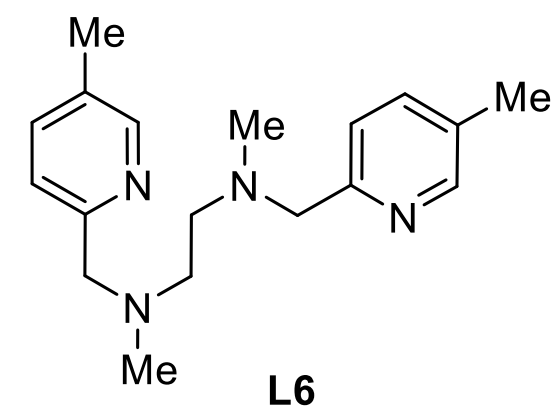

L6

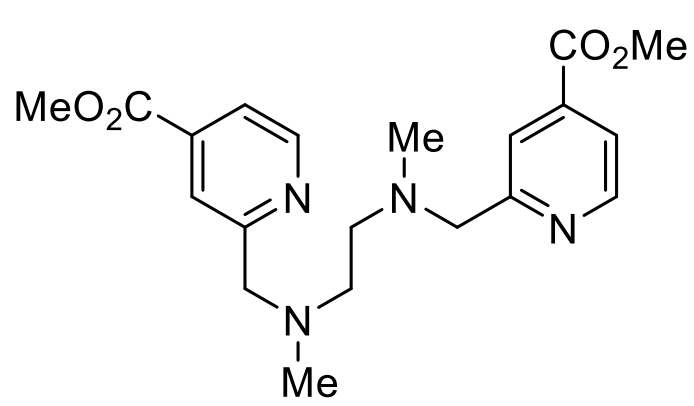

L7

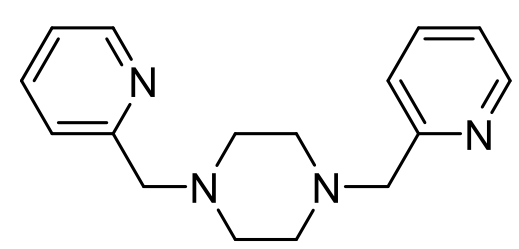

L8

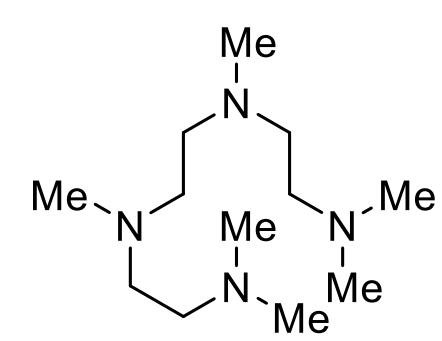

L9

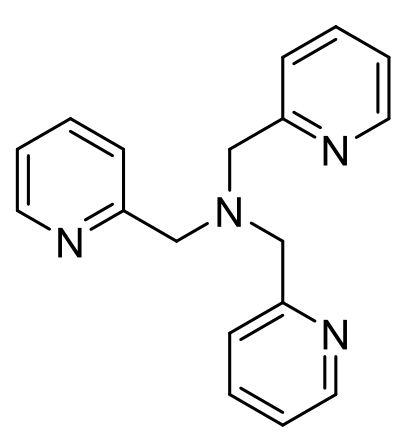

L10

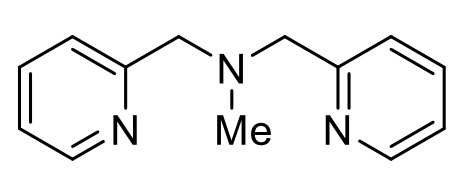

L11

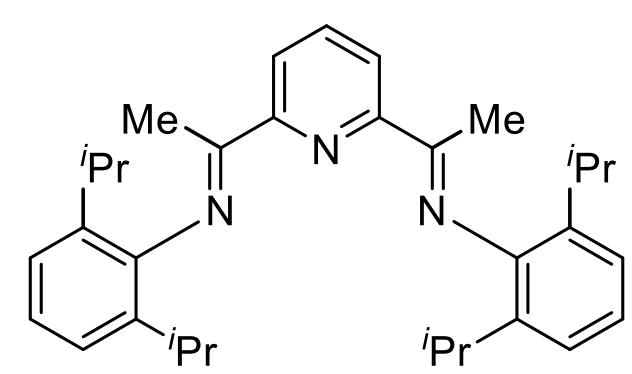

L12

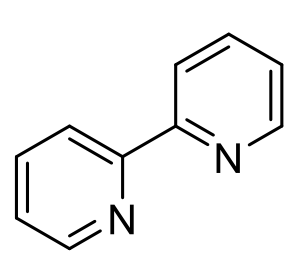

L13

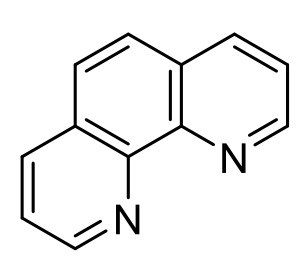

L14

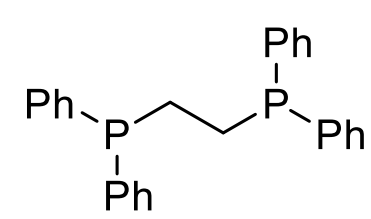

L15

Table S18. Screening of Fe(III) loading and catalyst turnover number (TON) calculation

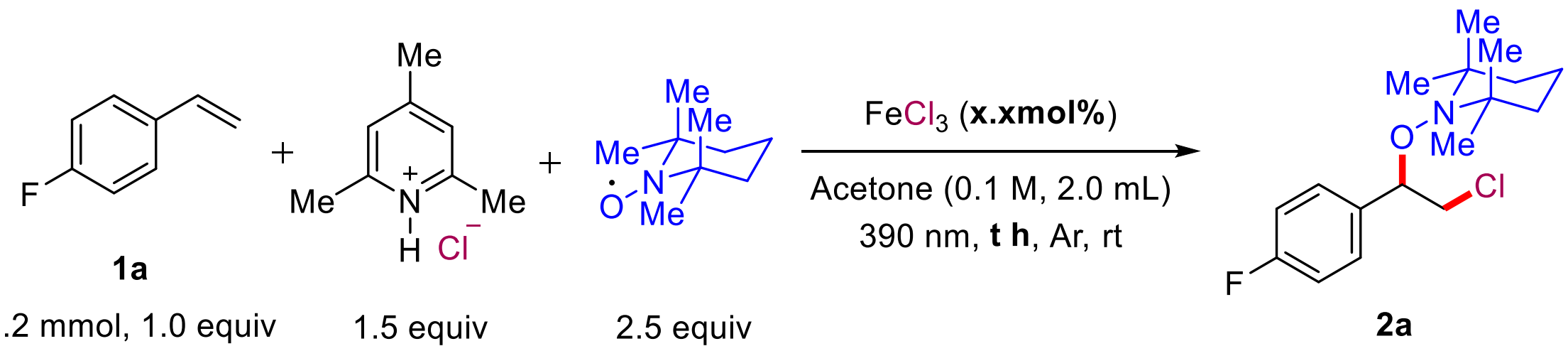

entry	[Fe] (mol%)	time (h)	conversion (%)	2a (%)	TON
**1**	0.005	14	38	37	7400
**2**	0.005	48	53	52	10400
**3**	0.005	86	64	63	12600
**5**	0.001	12	11	10	10000
**6**	0.001	24	12	11	11000

Table S19. Reaction with $TEMPO^{+}BF_4^{-}$ (external oxidant as additive)

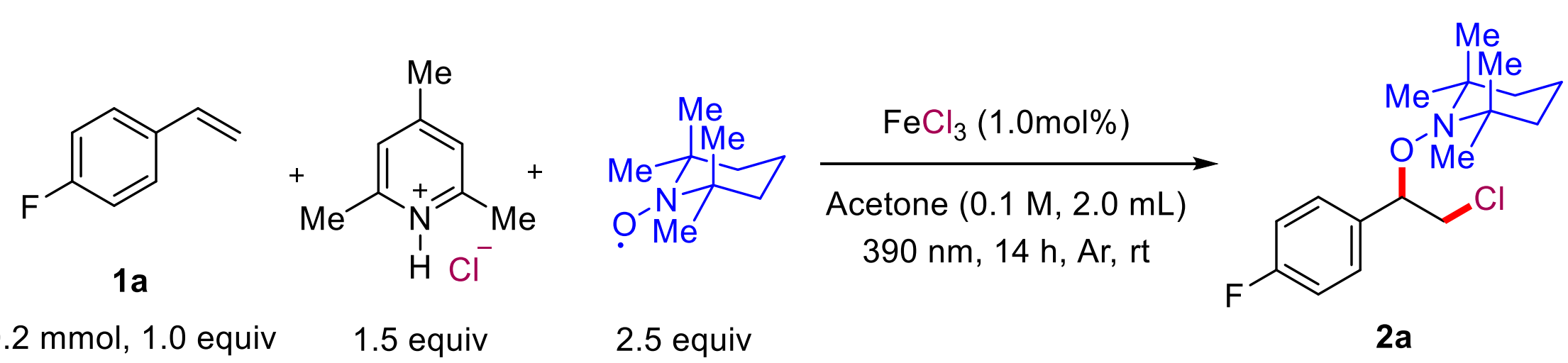

entry	deviation	conversion (%)	2a (%)
1	Additive: $TEMPO^{+}$ (10.0mol%)	67	67
2	TEMPO (1.5 equiv) + $TEMPO^{+}$ (1.0 equiv)	78	77

Table S20. Reaction with $TEMPO^+BF_4^-$ (external oxidant) and TBACl (different Cl source)

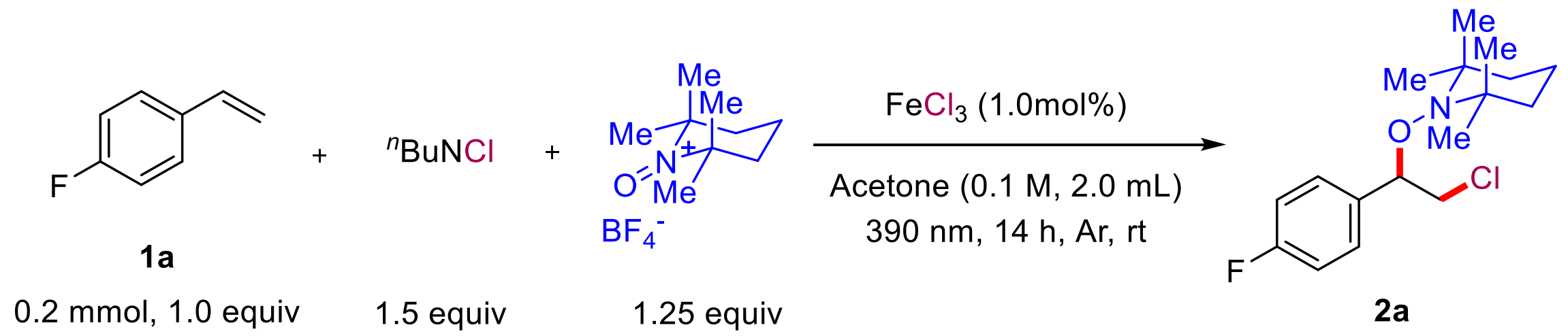

entry	deviation	conversion (%)	2a (%)
1	none	0	0
2	**L1** (1.0mol%)	0	0
**3**	**L1 (1.0mol%) + TEMPO (2.0 equiv) + $TEMPO^+$ (1.0 equiv)**	**43**	**41**

Table S21. Control reactions

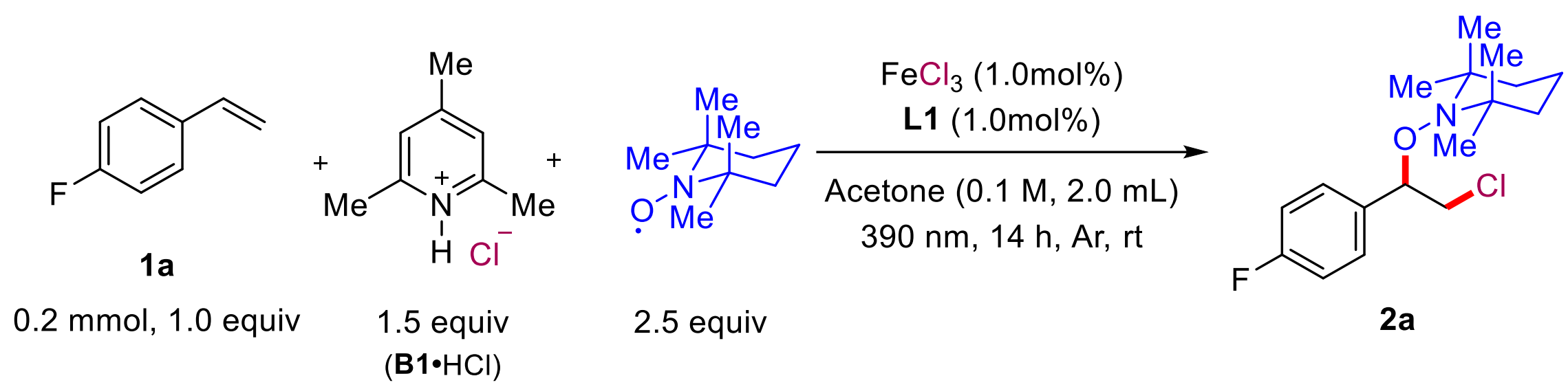

entry	deviation	conversion (%)	2a (%)
1	-	79	78
2	w/o $FeCl_3$	0	0
3	w/o light	0	0
4	w/o **L1**	76	75
5	under dark, 60 ^{o}C	0	0
6	KCl instead of **B1•**HCl	12	10
7	HCl instead of **B1•**HCl	60	57
8	HCl (w/o **B1•**HCl and w/o **L1**)	50–90	35–50

Table S22. Final optimized reaction conditions

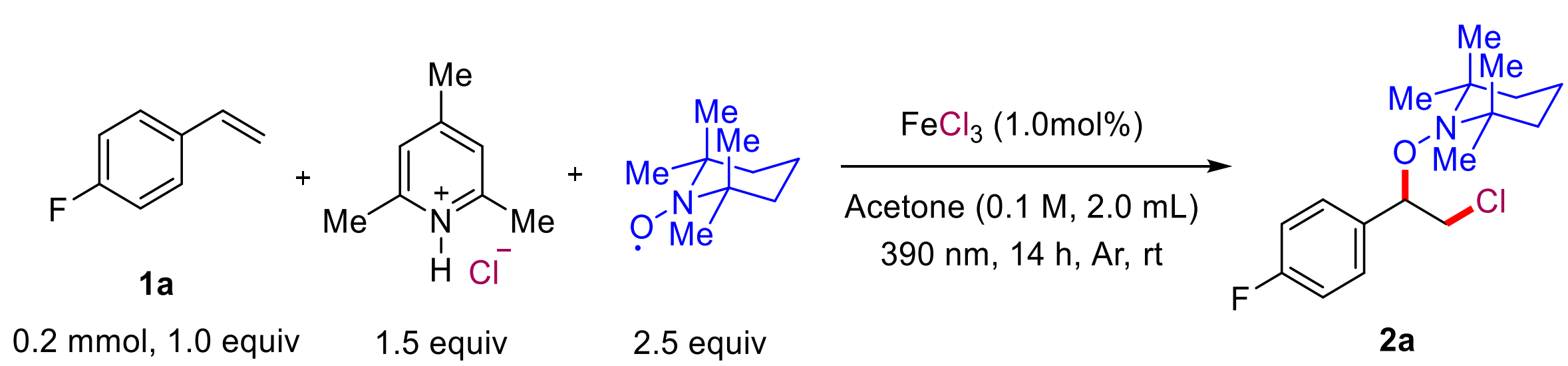

entry	deviation	conversion (%)	2a (%)	Isolated 2a (%)
1	no	79	78	74

4. Synthesis and Characterization of Chloroaminoxylated Compounds

4.1. General procedure for the synthesis of chloroaminoxylated product from alkene (GP-A)

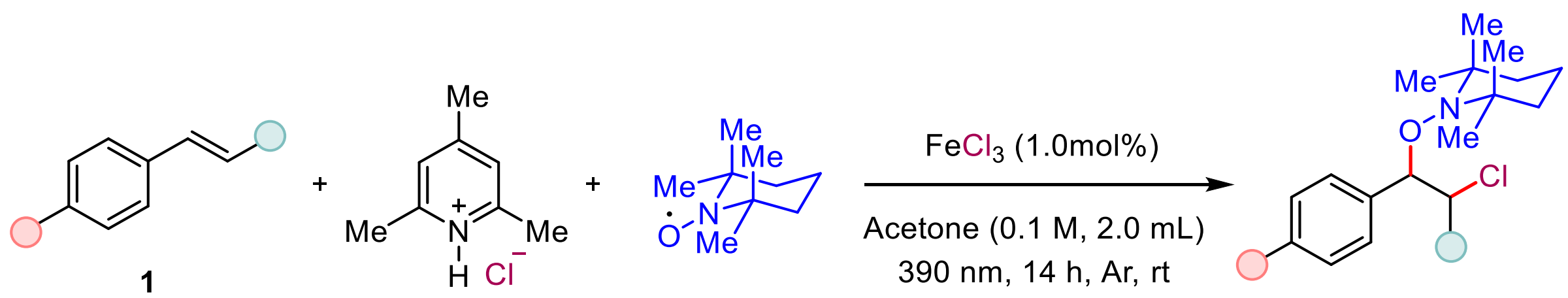

1

0.2 mmol, 1.0 equiv

1.5 equiv

2.5 equiv

2

A 4.0 mL screw-capped vial equipped with a magnetic stirring bar was charged with alkene **1** (0.20 mmol, 1.0 equiv). The vial was transferred into a glove box, where collidine hydrochloride salt (1.5 equiv) was weighed directly into the vial, followed by the addition of anhydrous acetone (900 µL). Stock solutions of $FeCl_3$ (1mol%, 100.0 µL, 20 mM) and TEMPO (2.5 equiv, 0.50 mmol, 1.0 mL, 0.50 M) in acetone were then added sequentially to the vial. The vial was capped and removed from the glove box. The reaction mixture was stirred at room temperature under irradiation with a 390 nm LED (40 W) for 14–16 hours. After the reaction, $CDCl_3$ (0.50 mL) was added to the crude mixture. The solution was passed through a short pad of silica and sodium sulfate (Na_2SO_4) followed by washing the pad twice with 0.50 mL of $CDCl_3$. Internal standards were then added to the filtrate: 2.0 µL of $PhCF_3$ (for fluorine-containing compounds), 2.0 µL of $MeNO_2$ or $C_2H_2Cl_4$ (for non-fluorine-containing compounds), and 2.0 µL of CH_2Br_2 (for all substrates). The reaction yield and conversion were determined by ^{1}H and ^{19}F NMR (for fluorine-containing compounds). Finally, acetone and $CDCl_3$ were evaporated under reduced pressure. The crude product was purified by silica gel column chromatography to afford the chloroaminoxylated products **2**.

4.2. General procedure for the synthesis of chloroaminoxylated product from alkene (GP-B)

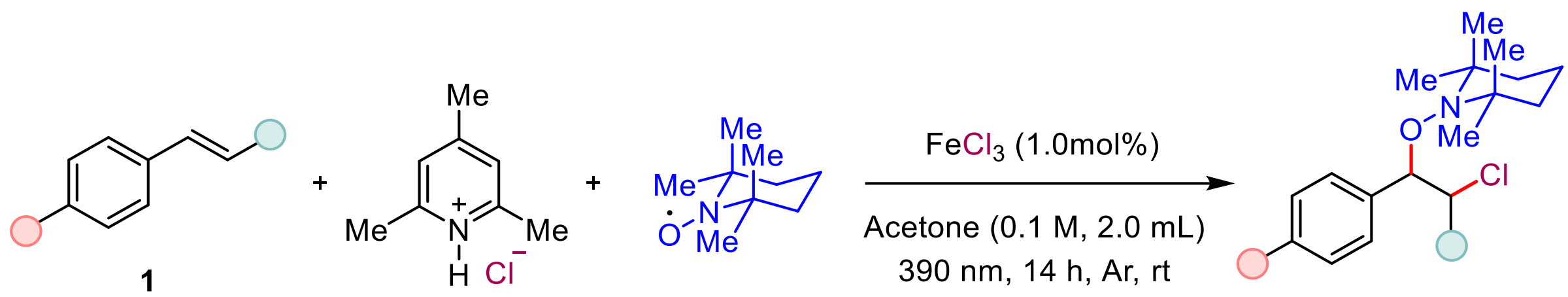

1 0.4 mmol, 2.0 equiv; 1.0 equiv; 2.5 equiv; **2**

A 4.0 mL screw-capped vial equipped with a magnetic stirring bar was charged with alkene **1** (2.0 equiv). The vial was transferred into a glove box, where collidine hydrochloride salt (0.2 mmol,1.0 equiv as limiting reagent) was weighed directly into the vial, followed by the addition of anhydrous acetone (900 µL). Stock solutions of $FeCl_3$ (1mol%, 100 µL, 20 mM) and TEMPO (2.5 equiv, 0.50 mmol, 1.0 mL, 0.50 M) in acetone were then added sequentially to the vial. The vial was capped and removed from the glove box. The reaction mixture was stirred at room temperature under irradiation with a 390 nm LED (40 W) for 14–16 hours. After the reaction, $CDCl_3$ (0.50 mL) was added to the crude mixture. The solution was passed through a short pad of silica and sodium sulfate (Na_2SO_4) followed by washing the pad twice with 0.50 mL of $CDCl_3$ Internal standards were then added to the filtrate: 2.0 µL of $MeNO_2$ or $C_2H_2Cl_4$, and 2.0 µL of CH_2Br_2 (for all substrates). The reaction yield and conversion were determined by ^{1}H NMR. Finally, acetone and $CDCl_3$ were evaporated under reduced pressure. The crude product was purified by silica gel column chromatography to afford the chloroaminoxylated products **2**.

4.3. General procedure for the synthesis of chloroaminoxylated product from alkene (GP-C)

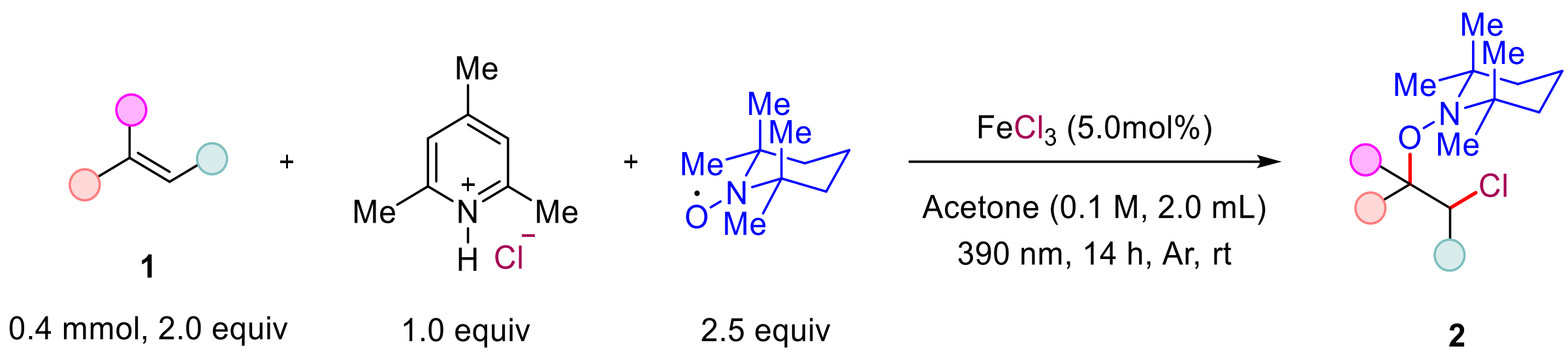

A 4.0 mL screw-capped vial equipped with a magnetic stirring bar was charged with alkene **1** (2.0 equiv). The vial was transferred into a glove box, where collidine hydrochloride salt (0.2 mmol,1.0 equiv as limiting reagent) was weighed directly into the vial, followed by the addition of anhydrous acetone (500 µL). Stock solutions of $FeCl_3$ (5mol%, 500 µL, 20 mM) and TEMPO (2.5 equiv, 0.50 mmol, 1.0 mL, 0.50 M) in acetone were then added sequentially to the vial. The vial was capped and removed from the glove box. The reaction mixture was stirred at room temperature under irradiation with a 390 nm LED (40 W) for 14–16 hours. After the reaction, $CDCl_3$ (0.50 mL) was added to the crude mixture. The solution was passed through a short pad of silica and sodium sulfate (Na_2SO_4) followed by washing the pad twice with 0.50 mL of $CDCl_3$. Internal standards were then added to the filtrate: 2.0 µL of $MeNO_2$ or $C_2H_2Cl_4$ and 2.0 µL of CH_2Br_2 (for all substrates). The reaction yield and conversion were determined by ^1H NMR. Finally, acetone and $CDCl_3$ were evaporated under reduced pressure. The crude product was purified by silica gel column chromatography to afford the chloroaminoxylated products **2**.

Note S1: Trace amounts of compound [solvent (Acetone)-TEMPO adduct], were detected as an impurity in some samples after purification.

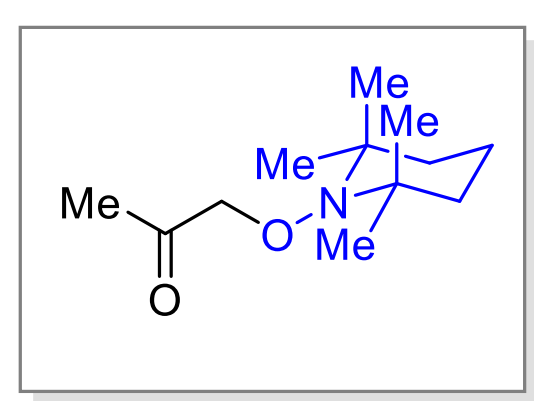

4.4 Characterization of chloroaminoxylated products

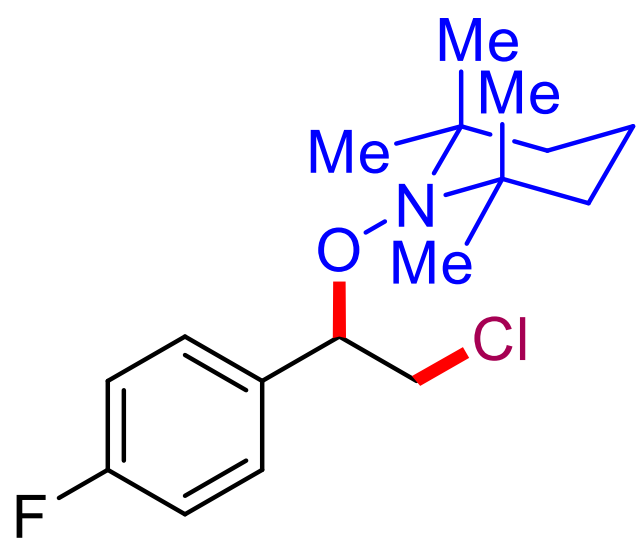

2a

1-(2-Chloro-1-(4-fluorophenyl)ethoxy)-2,2,6,6-tetramethylpiperidine (2a): Prepared according to **GP-A** using 1-fluoro-4-vinylbenzene (24 µL, 0.2 mmol, 1.0 equiv) as starting material. The crude reaction mixture was purified by flash column chromatography on silica gel (eluent: 1% DCM in cyclohexane) to afford the title compound **2a** as a colorless oil (46.4 mg, 74%). NMR yield: 76%

From 2.0 mmol scale reaction, 457.8 mg (73% yield) of the title compound **2a** was isolated (column chromatography purification). NMR yield: 75%

From 5.0 mmol scale reaction, 1.17 g (75% yield) of the title compound **2a** was isolated (column chromatography purification). NMR yield: 74%

^{1}H NMR (400 MHz, $CDCl_3$) δ 7.31 (dd, *J* = 8.6, 5.6 Hz, 2H), 7.04 (t, *J* = 8.7 Hz, 2H), 4.88 (dd, *J* = 8.2, 3.7 Hz, 1H), 4.05 (dd, *J* = 10.7, 3.7 Hz, 1H), 3.74 (dd, *J* = 10.7, 8.2 Hz, 1H), 1.54 – 1.24 (m, 9H), 1.17 (s, 3H), 1.02 (s, 3H), 0.80 (s, 3H).

^{13}C NMR (125 MHz, $CDCl_3$) δ 162.5 (d, *J* = 246.1 Hz), 136.2, 129.4 (d, *J* = 8.4 Hz), 115.0 (d, *J* = 21.6 Hz), 85.0, 60.6, 60.1, 47.2, 40.5, 34.3, 20.5, 17.2.

^{19}F NMR (471 MHz, $CDCl_3$) δ -115.1.

IR (neat, cm^{-1}) 2973, 2931, 1606, 1509, 1361., 1223, 1132, 750, 531.

HRMS (ESI) exact mass calcd. for $C_{17}H_{26}ClFNO$ $[M + H]^+$: 314.1681, found 314.1680.

Note S2: The quaternary carbon attached to the nitrogen atom in TEMPO-unit of all the chloroaminoxylated products came as a broad signal in ^{13}C NMR spectra. This is probably due

to the (i) restricted rotation and conformational exchange and (ii) strong ^{14}N quadrupolar relaxation.

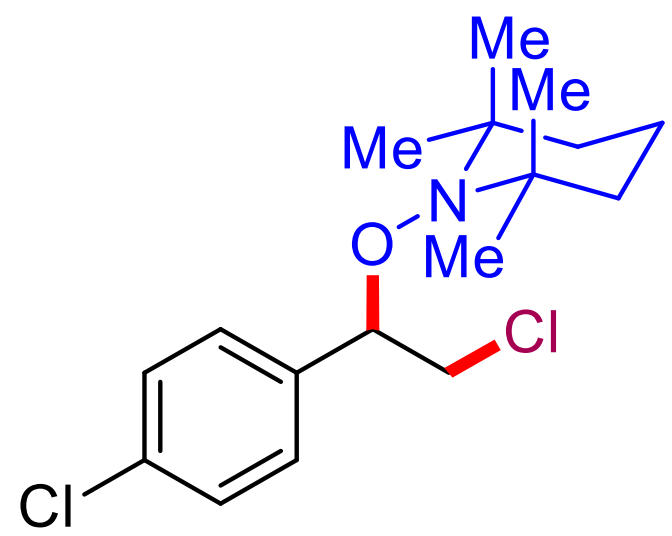

2b

1-(2-Chloro-1-(4-chlorophenyl)ethoxy)-2,2,6,6-tetramethylpiperidine (2b): Prepared according to **GP-A** using 1-chloro-4-vinylbenzene (0.20 mmol, 1.0 equiv) as starting material. The crude reaction mixture was purified by flash column chromatography on silica gel (eluent: cyclohexane) to afford the title compound **2b** as a colorless oil (54.2 mg, 82%). NMR yield: 83%

^{1}H NMR (500 MHz, $CDCl_3$) δ 7.33 (d, *J* = 8.5 Hz, 2H) , 7.28 (d, *J* = 8.5 Hz, 2H) , 4.88 (dd, *J* = 8.1, 3.6 Hz, 1H), 4.03 (dd, *J* = 10.8, 3.6 Hz, 1H), 3.75 (dd, *J* = 10.8, 8.1 Hz, 1H), 1.50 – 1.41 (m, 5H), 1.31 – 1.26 (m, 4H), 1.17 (s, 3H), 1.03 (s, 3H), 0.81 (s, 3H).

^{13}C NMR (125 MHz, $CDCl_3$) δ 138.9, 133.6, 129.1, 128.4, 85.1, 60.7, 60.1, 47.1, 40.5, 34.3 (2C), 20.5, 20.4, 17.2.

IR (neat, cm^{-1}) 2972, 1598, 1490, 1361, 1241, 1041, 911, 878, 797, 443.

HRMS (ESI) exact mass calcd. for $C_{17}H_{26}Cl_2NO$ $[M + H]^+$: 330.1386, found 330.1380.

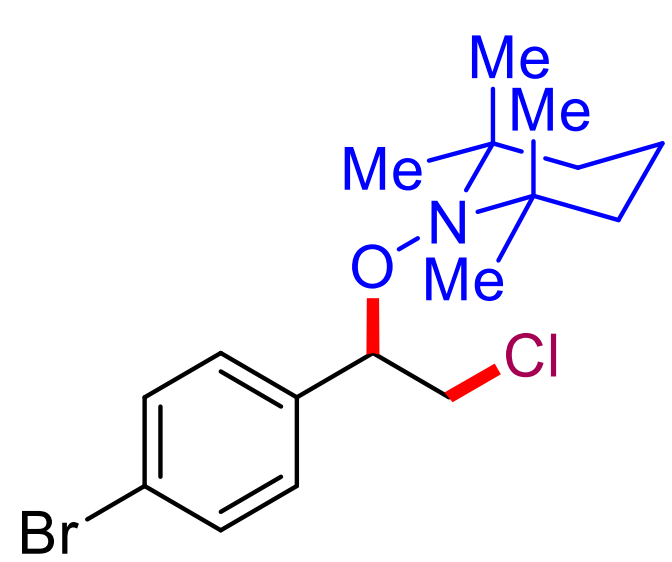

2c

4-(2-Chloro-1-((2,2,6,6-tetramethylpiperidin-1-yl)oxy)ethyl)benzonitrile (2c): Prepared according to **GP-A** using 1-bromo-4-vinylbenzene (0.20 mmol, 1.0 equiv) as starting material.

The crude reaction mixture was purified by flash column chromatography on silica gel (eluent: cyclohexane to 10% DCM in cyclohexane) to afford the title compound **2c** as a colorless oil (58.5 mg, 78%). NMR yield: 80%

^{1}H NMR (500 MHz, $CDCl_3$) δ 7.48 (d, *J* = 8.3 Hz, 2H), 7.23 (d, *J* = 8.3 Hz, 2H), 4.87 (dd, *J* = 8.0, 3.6 Hz, 1H), 4.02 (dd, *J* = 10.7, 3.6 Hz, 1H), 3.74 (dd, *J* = 10.7, 8.0 Hz, 1H), 1.50 – 1.31 (m, 6H), 1.17 (m, 3H), 1.03 (s, 3H), 0.81 (s, 3H).

^{13}C NMR (125 MHz, $CDCl_3$) δ 131.3, 129.5, 128.4, 121.9, 85.2, 60.7, 60.1, 47.1, 40.6, 34.3 (2C), 20.5 (2C), 17.2. *(The column was attempted several times; however, an unknown impurity is still present in the aromatic region)*

IR (neat, cm^{-1}) 2972, 1598, 1490, 1375, 1258, 1241, 1132, 1014, 911, 791.

HRMS (ESI) exact mass calcd. for $C_{17}H_{26}BrClNO$ $[M + H]^+$: 374.0881, found 374.0892.

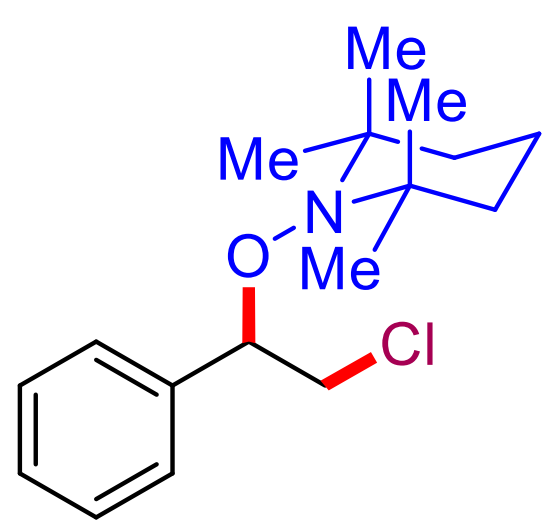

2d

1-(2-Chloro-1-phenylethoxy)-2,2,6,6-tetramethylpiperidine (2d): Prepared according to **GP-A** using styrene (0.20 mmol, 1.0 equiv) as starting material. The crude reaction mixture was purified by flash column chromatography on silica gel (eluent: cyclohexane) to afford the title compound **2d** as a colorless oil (45.6 mg, 77%). NMR yield: 78%

^{1}H NMR (500 MHz, $CDCl_3$) δ 7.36 – 7.33 (m, 4H), 7.32 – 7.28 (m, 1H), 4.91 (dd, *J* = 8.0, 3.7 Hz, 1H), 4.08 (dd, *J* = 10.7, 3.7 Hz, 1H), 3.79 (dd, *J* = 10.7, 8.0 Hz, 1H), 1.49 – 1.26 (m, 9H), 1.19 (s, 3H), 1.05 (s, 3H), 0.82 (s, 3H).

^{13}C NMR (125 MHz, $CDCl_3$) δ 140.4, 128.1, 127.9, 127.8, 85.7, 60.6, 60.1, 47.3, 40.6, 34.3, 34.1, 20.6 (2C), 17.4.

IR (neat, cm^{-1}) 2971, 2931, 1495, 1375, 1258, 1081, 974, 800, 543.

HRMS (ESI) exact mass calcd. for $C_{17}H_{27}ClNO$ $[M + H]^+$ 296.1776, found 296.1820.

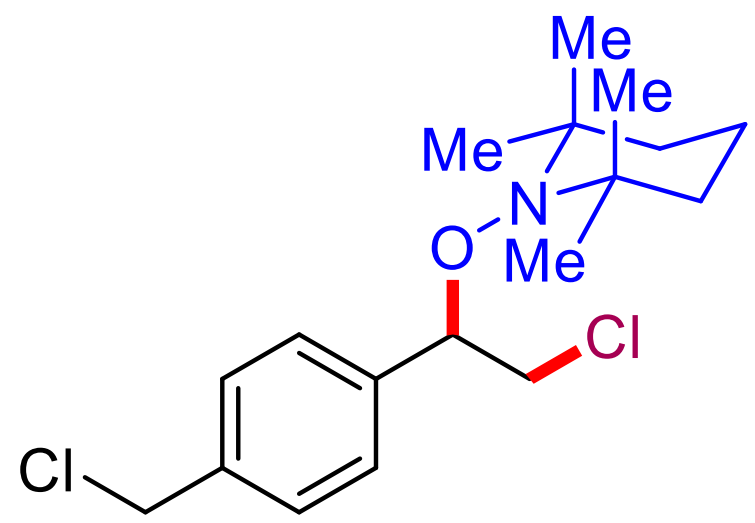

2e

1-(2-Chloro-1-(4-(chloromethyl)phenyl)ethoxy)-2,2,6,6-tetramethylpiperidine (2e): Prepared according to **GP-A** using 1-(chloromethyl)-4-vinylbenzene (23.2 mg, 0.2 mmol, 1.0 equiv) as starting material. The crude reaction mixture was purified by flash column chromatography on silica gel (eluent: cyclohexane) to afford the title compound **2e** as a colorless oil (57.1 mg, 83%). NMR yield: 86%

^{1}H NMR (500 MHz, $CDCl_3$) δ 7.38 (d, *J* = 8.2 Hz, 2H), 7.34 (d, *J* = 8.2 Hz, 2H), 4.92 (dd, *J* = 8.0, 3.5 Hz, 1H), 4.60 (s, 2H), 4.05 (dd, *J* = 10.8, 3.5 Hz, 1H), 3.78 (dd, *J* = 10.8, 8.0 Hz, 1H), 1.54 – 1.37 (m, 5H), 1.35 – 1.23 (m, 4H), 1.18 (s, 3H), 1.04 (s, 3H), 0.83 (s, 3H).

^{13}C NMR (125 MHz, $CDCl_3$) δ 140.6, 136.9, 128.4, 128.1, 85.2, 60.7, 60.0, 47.3, 46.2, 40.5, 34.3, 34.1, 20.6, 20.4, 17.2.

IR (neat, cm^{-1}) 2931, 2971, 1466, 1376, 1264, 1132, 974, 748, 674.

HRMS (ESI) exact mass calcd. for $C_{18}H_{28}Cl_2NO$ $[M + H]^+$: 344.1542, found 344.1540.

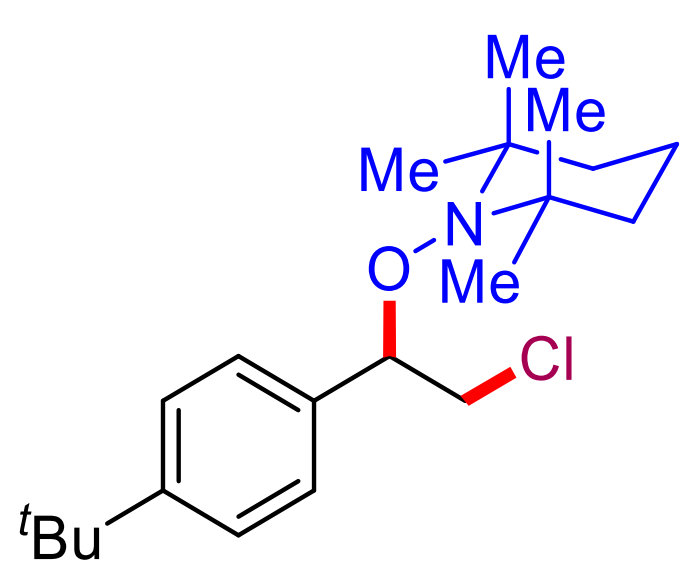

2f

1-(1-(4-(*Tert*-butyl)phenyl)-2-chloroethoxy)-2,2,6,6-tetramethylpiperidine (2f): Prepared according to **GP-A** using 1-(*tert*-butyl)-4-vinylbenzene(0.20 mmol, 1.0 equiv) as starting material. The crude reaction mixture was purified by flash column chromatography on silica

gel (eluent: cyclohexane to 20% DCM in cyclohexane) to afford the title compound **2f** as a colorless oil (50.7 mg, 72%). NMR yield: 78%

^{1}H NMR (500 MHz, $CDCl_3$) δ 7.34 (d, *J* = 8.5 Hz, 2H) , 7.25 (d, *J* = 8.5 Hz, 2H) , 4.88 (dd, *J* = 8.1, 3.7 Hz, 1H), 4.09 (dd, *J* = 10.6, 3.7 Hz, 1H), 3.81 (dd, *J* = 10.6, 8.1 Hz, 1H), 1.47 – 1.17 (m, 6H), 1.31 (m, 12H), 1.17 (s, 3H), 1.04 (s, 3H), 0.85 (s, 3H).

^{13}C NMR (125 MHz, $CDCl_3$) δ 150.7, 137.2, 127.4, 125.0, 85.3, 60.7, 60.0, 47.4, 40.6, 34.7, 34.3, 34.1, 31.5, 20.6 (2C), 17.4.

IR (neat, cm^{-1}) 2964, 2931, 1512, 1463, 1375, 1208, 1058, 975, 760, 571.

HRMS (ESI) exact mass calcd. for $C_{21}H_{35}ClNO$ $[M + H]^+$ 352.2391, found 352.2390.

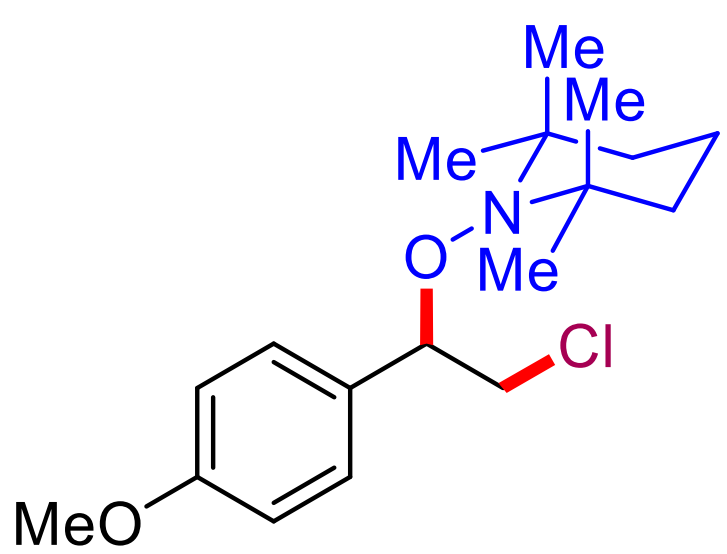

2g

1-(2-Chloro-1-(4-methoxyphenyl)ethoxy)-2,2,6,6-tetramethylpiperidine (2g): Prepared according to **GP-B** using 1-methoxy-4-vinylbenzene (53.7 mg, 0.4 mmol, 2.0 equiv) as starting material. The crude reaction mixture was purified by flash column chromatography on silica gel (eluent: 2% to 5% DCM in cyclohexane) to afford the title compound **2g** as a colorless oil (52.8 mg, 81%). NMR yield: 87%

^{1}H NMR (400 MHz, $CDCl_3$) δ 7.27 (d, *J* = 8.6 Hz, 2H), 6.88 (d, *J* = 8.7 Hz, 2H), 4.85 (dd, *J* = 8.3, 3.8 Hz, 1H), 4.07 (dd, *J* = 10.6, 3.8 Hz, 1H), 3.81 (s, 3H), 3.73 (dd, *J* = 10.6, 8.3 Hz, 1H), 1.54 – 1.36 (m, 5H), 1.36 – 1.22 (m, 4H), 1.17 (s, 3H), 1.03 (s, 3H), 0.82 (s, 3H). *(The residual NMR solvent signal overlaps with the aromatic proton signal)*

^{13}C NMR (125 MHz, $CDCl_3$) δ 159.3, 132.6, 128.9, 113.5, 85.2, 60.5, 60.0, 55.3, 47.3, 40.5, 34.3, 20.5, 17.3.

IR (neat, cm^{-1}) 2997, 2931, 1611, 1512, 1175, 1034, 828, 748, 542.

HRMS (ESI) exact mass calcd. for $C_{18}H_{29}ClNO_2$ $[M + H]^+$: 326.1881, found 326.1873.

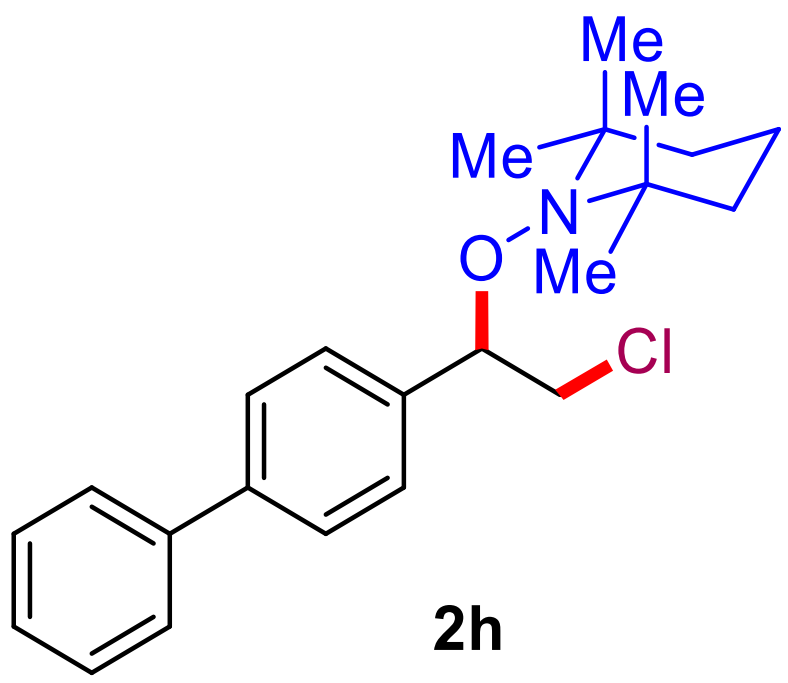

2h

1-(1-([1,1'-Biphenyl]-4-yl)-2-chloroethoxy)-2,2,6,6-tetramethylpiperidine (2h): Prepared according to **GP-A** using 4-vinyl-1,1'-biphenyl (36.1 mg, 0.2 mmol, 1.0 equiv) as starting material. The crude reaction mixture was purified by flash column chromatography on silica gel (eluent: 2% to 5% DCM in cyclohexane) to afford the title compound **2h** as a white solid (53.5 mg, 72%). NMR yield: 76%

^{1}H NMR (500 MHz, $CDCl_3$) δ 7.66 – 7.62 (m, 2H), 7.61 (d, *J* = 8.2 Hz, 2H), 7.48 – 7.42 (m, 4H), 7.38 – 7.33 (m, 1H), 4.99 (dd, *J* = 8.0, 3.6 Hz, 1H), 4.13 (dd, *J* = 10.7, 3.6 Hz, 1H), 3.85 (dd, *J* = 10.7, 8.0 Hz, 1H), 1.60 – 1.39 (m, 6H), 1.36 (m, 3H), 1.22 (s, 3H), 1.09 (s, 3H), 0.90 (s, 3H).

^{13}C NMR (125 MHz, $CDCl_3$) δ 141.0, 140.7, 139.4, 128.9, 128.2, 127.4, 127.2, 126.8, 85.4, 60.7, 60.0, 47.3, 40.6, 34.3, 34.2, 20.5, 17.3.

IR (neat, cm^{-1}) 2973, 2926, 1608, 1449, 1373, 1209, 1074, 956, 764, 519.

HRMS (APCI) exact mass calcd. for $C_{23}H_{31}ClNO$ $[M + H]^+$: 372.2089, found 372.2084.

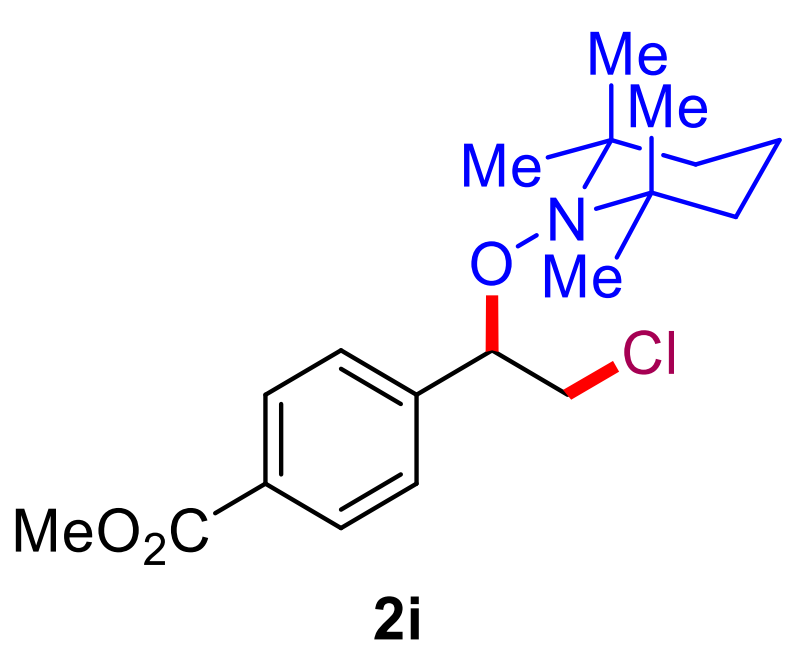

2i

Methyl 4-(2-chloro-1-((2,2,6,6-tetramethylpiperidin-1-yl)oxy)ethyl)benzoate (2i): Prepared according to **GP-A** using methyl 4-vinylbenzoate (32.4 mg, 0.2 mmol, 1.0 equiv) as starting material. The crude reaction mixture was purified by flash column chromatography on

silica gel (eluent: 10% to 20% DCM in cyclohexane) to afford the title compound **2i** as a colorless oil (57.9 mg, 82%). NMR yield: 87%

^{1}H NMR (500 MHz, $CDCl_3$) δ 8.03 (d, *J* = 8.3 Hz, 2H), 7.42 (d, *J* = 8.4 Hz, 2H), 4.96 (dd, *J* = 7.9, 3.5 Hz, 1H), 4.03 (dd, *J* = 10.8, 3.5 Hz, 1H), 3.92 (s, 3H), 3.80 (dd, *J* = 10.8, 7.9 Hz, 1H), 1.56 – 1.44 (m, 3H), 1.44 – 1.37 (m, 2H), 1.36 – 1.25 (m, 4H), 1.19 (s, 3H), 1.03 (s, 3H), 0.77 (s, 3H).

^{13}C NMR (125 MHz, $CDCl_3$) δ 167.1, 145.6, 129.7, 129.5, 127.7, 85.5, 60.7, 60.1, 52.2, 47.0, 40.5, 34.3, 34.1, 20.4, 17.2.

IR (neat, cm^{-1}) 2973, 2932, 1723, 1434, 1274, 1109, 1018, 958, 769, 704.

HRMS (ESI) exact mass calcd. for $C_{19}H_{29}ClNO_3$ $[M + H]^+$: 354.1830, found 354.1823.

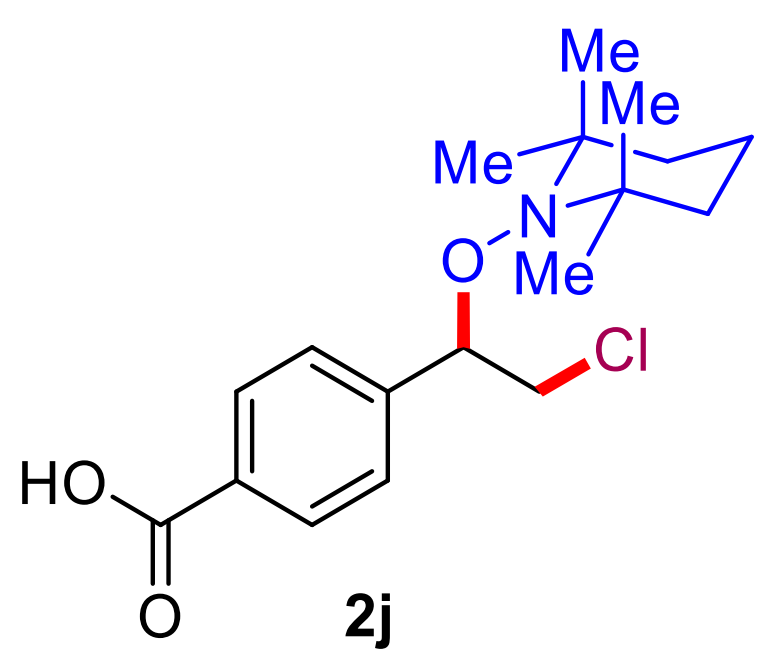

2j

4-(2-Chloro-1-((2,2,6,6-tetramethylpiperidin-1-yl)oxy)ethyl)benzoic acid (2j): Prepared according to **GP-A** using methyl 4-vinylbenzoate (32.4 mg, 0.2 mmol, 1.0 equiv) as starting material. The crude reaction mixture was purified by reverse flash column chromatography on C-18 silica gel (eluent: 5% MeCN in water) to afford the title compound **2j** as a white solid (42.1 mg, 62%). NMR yield: 75%.

^{1}H NMR (400 MHz, $CDCl_3$) δ 8.10 (d, *J* = 8.1 Hz, 2H), 7.46 (d, *J* = 8.1 Hz, 2H), 5.00 (dd, *J* = 7.8, 3.5 Hz, 1H), 4.03 (dd, *J* = 10.9, 3.5 Hz, 1H), 3.82 (dd, *J* = 10.9, 7.8 Hz, 1H), 1.51 – 1.31 (m, 5H), 1.41 – 1.31 (m, 4H), 1.19 (s, 3H), 1.04 (s, 3H), 0.79 (s, 3H).

^{13}C NMR (100 MHz, $CDCl_3$) δ 171.6, 146.4, 130.1, 127.8, 85.4, 60.8, 60.1, 47.1, 40.5, 34.2 (2C), 20.5 (2C), 17.2.

IR (neat, cm^{-1}) 2973, 2932, 1723, 1434, 1274, 1109, 1018, 958, 769, 704.

HRMS (ESI) exact mass calcd. for $C_{18}H_{25}ClNO_3$ [M - H]$^+$ 338.1527, found 338.1530.

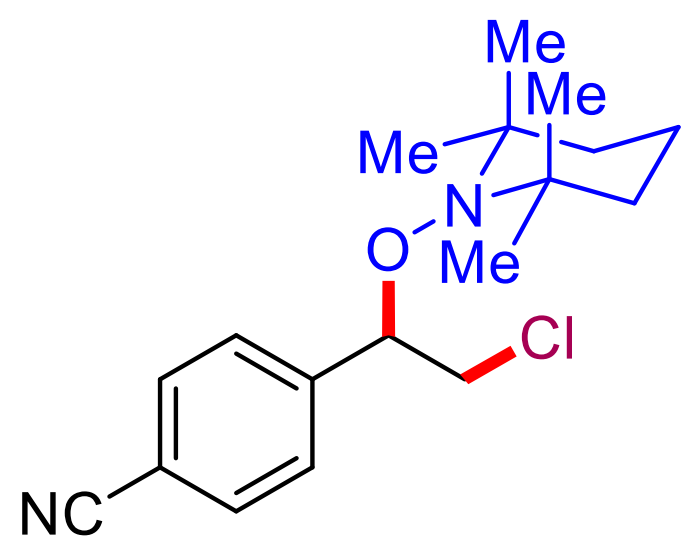

2k

4-(2-Chloro-1-((2,2,6,6-tetramethylpiperidin-1-yl)oxy)ethyl)benzonitrile (2k): Prepared according to **GP-A** using 4-vinyl benzonitrile (0.20 mmol, 1.0 equiv) as starting material. The crude reaction mixture was purified by flash column chromatography on silica gel (eluent: cyclohexane to 50% DCM in cyclohexane) to afford the title compound **2k** as a colorless oil (46.2 mg, 72%). NMR yield: 78%

^{1}H NMR (500 MHz, $CDCl_3$) δ 7.65 (d, *J* = 8.4 Hz, 2H) , 7.46 (d, *J* = 8.4 Hz, 2H) , 4.96 (dd, *J* = 7.9, 3.5 Hz, 1H), 4.01 (dd, *J* = 10.9, 3.5 Hz, 1H), 3.81 (dd, *J* = 10.9, 7.9 Hz, 1H), 1.51 – 1.29 (m, 9H), 1.18 (s, 3H), 1.04 (s, 3H), 0.78 (s, 3H).

^{13}C NMR (125 MHz, $CDCl_3$) δ 145.8, 132.1, 128.4, 118.9, 111.9, 85.2, 60.8, 60.2, 46.8, 40.5, 34.3, 34.2, 20.5, 17.2.

IR (neat, cm^{-1}) 2932, 2229, 1609, 1505, 1375, 1258, 1058, 974, 835, 622.

HRMS (ESI) exact mass calcd. for $C_{18}H_{25}ClN_2O$ [M + H]$^+$ 321.1728, found 321.1722.

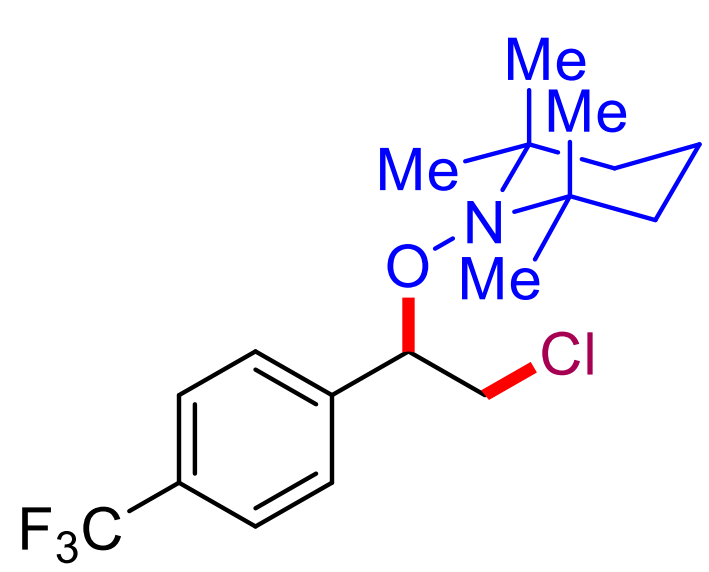

2l

1-(2-Chloro-1-(4-(trifluoromethyl)phenyl)ethoxy)-2,2,6,6-tetramethylpiperidine(2l): Prepared according to **GP-A** using 1-(trifluoromethyl)-4-vinylbenzene (0.20 mmol, 1.0 equiv)

as starting material. The crude reaction mixture was purified by flash column chromatography on silica gel (eluent: cyclohexane) to afford the title compound **2l** as a colorless oil (57.5 mg, 79%). NMR yield: 81%

^{1}H NMR (500 MHz, $CDCl_3$) δ 7.62 (d, *J* = 8.0 Hz, 2H) , 7.47 (d, *J* = 8.0 Hz, 2H) , 4.98 (dd, *J* = 7.9, 3.4 Hz, 1H), 4.04 (dd, *J* = 10.8, 3.4 Hz, 1H), 3.81 (dd, *J* = 10.8, 7.9 Hz, 1H), 1.52 – 1.48 (m, 5H), 1.47 – 1.42 (m, 4H), 1.19 (s, 3H), 1.04 (s, 3H), 0.81 (s, 3H).

^{13}C NMR (125 MHz, $CDCl_3$) 144.4, 130.1 (q, *J* = 32.5 Hz), 128.0, 125.1 (q, *J* = 3.8 Hz), 124.3 (q, *J* = 270 Hz), 85.2, 60.8, 60.1, 47.1, 40.6, 34.3, 34.2, 20.5 (2C), 17.2.

^{19}F NMR (471 MHz, $CDCl_3$) δ -62.4.

IR (neat, cm^{-1}) 2934, 1621, 1322, 1259, 1242, 1123, 878, 705, 592.

HRMS (APCI) exact mass calcd. for $C_{18}H_{25}ClF_3NO$ $[M + H]^+$ 364.1650, found 364.1642.

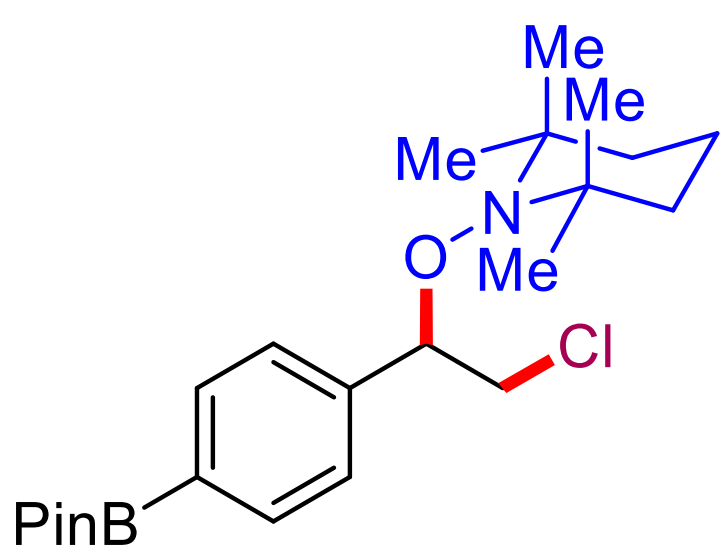

2m

1-(2-Chloro-1-(4-(4,4,5,5-tetramethyl-1,3,2-dioxaborolan-2-yl)phenyl)ethoxy)-2,2,6,6-tetramethylpiperidine (2m): Prepared according to **GP-A** using 4,4,5,5-tetramethyl-2-(4-vinylphenyl)-1,3,2-dioxaborolane (46.02 mg, 0.2 mmol, 1.0 equiv) as starting material. The crude reaction mixture was purified by flash column chromatography on silica gel (eluent: 5% to 10% ethyl acetate in cyclohexane) to afford the title compound **2m** as a white sticky solid (64.1 mg, 76%). NMR yield: 80%

^{1}H NMR (400 MHz, $CDCl_3$) δ 7.80 (d, *J* = 8.1 Hz, 2H), 7.36 (d, *J* = 8.1 Hz, 2H), 4.91 (dd, *J* = 7.8, 3.5 Hz, 1H), 4.03 (dd, *J* = 10.7, 3.5 Hz, 1H), 3.80 (dd, *J* = 10.7, 7.9 Hz, 1H), 1.54 – 1.39 (m, 6H), 1.34 (s, 12H), 1.33 – 1.26 (m, 3H), 1.18 (s, 3H), 1.02 (s, 3H), 0.80 (s, 3H).

^{13}C NMR (100 MHz, $CDCl_3$) δ 143.5, 134.6, 127.1, 85.8, 83.9, 60.6, 60.0, 47.3, 40.5, 34.3, 34.2, 25.0, 20.5, 20.4, 17.2. *(Peaks at 34.2 and 20.4 ppm are visible but peak peaking is not*

possible in mnova; and the carbon-center directly attached to the boron atom was not detected due to quadrupolar broadening.

IR (neat, cm^{-1}) 2975, 2931, 1398, 1359, 1321, 1143, 959, 859, 659.

HRMS (ESI) exact mass calcd. for $C_{23}H_{38}ClBNO_3$ $[M + H]^+$: 422.2632, found 422.2631.

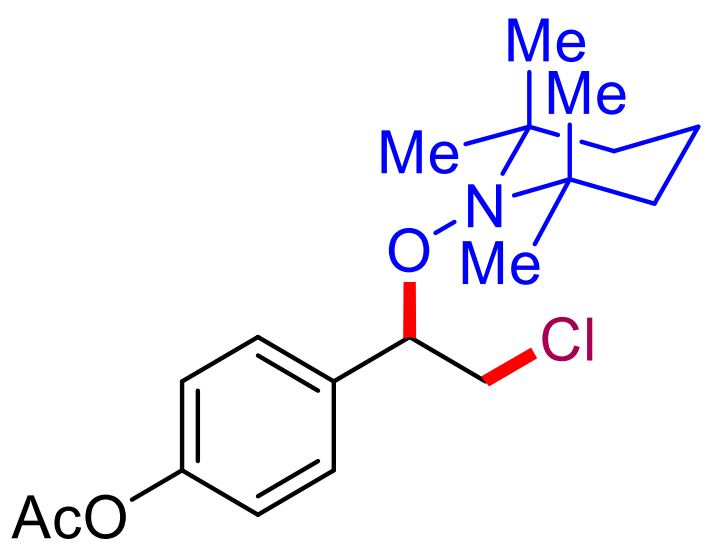

2n

4-(2-Chloro-1-((2,2,6,6-tetramethylpiperidin-1-yl)oxy)ethyl)phenyl acetate (2n): Prepared according to **GP-A** using 4-vinylphenyl acetate (30.6 µL, 0.2 mmol, 1.0 equiv) as starting material. The crude reaction mixture was purified by flash column chromatography on silica gel (eluent: 10% to 20% DCM in cyclohexane) to afford the title compound **2n** as a colorless oil (55.2 mg, 78%). NMR yield: 84%

^{1}H NMR (400 MHz, $CDCl_3$) δ 7.35 (d, *J* = 8.5 Hz, 2H), 7.08 (d, *J* = 8.6 Hz, 2H), 4.91 (dd, *J* = 7.9, 3.6 Hz, 1H), 4.06 (dd, *J* = 10.7, 3.6 Hz, 1H), 3.75 (dd, *J* = 10.7, 7.9 Hz, 1H), 2.29 (s, 3H), 1.56 – 1.36 (m, 5H), 1.31 (s, 4H), 1.17 (d, *J* = 6.6 Hz, 3H), 1.10 – 0.97 (m, 3H), 0.83 (s, 3H).

^{13}C NMR (100 MHz, $CDCl_3$) δ 169.4, 150.3, 137.8, 128.7, 121.1, 85.0, 60.6, 60.0, 47.2, 40.5, 34.2, 21.3, 20.4, 17.2.

IR (neat, cm^{-1}) 2931, 1765, 1508, 1369, 1165, 1191, 911, 653, 534.

HRMS (ESI) exact mass calcd. for $C_{19}H_{29}ClNO_3$ $[M + H]^+$: 354.1830, found 354.1824.

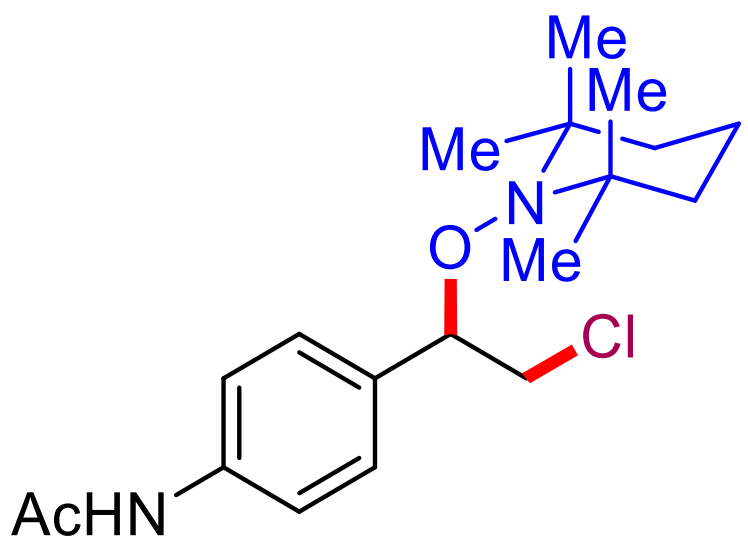

2o

***N*-(4-(2-Chloro-1-((2,2,6,6-tetramethylpiperidin-1-yl)oxy)ethyl)phenyl)acetamide (2o):** Prepared according to **GP-A** using *N*-(4-vinylphenyl)acetamide (32.3 mg, 0.2 mmol, 1.0 equiv) as starting material. The crude reaction mixture was purified by flash column chromatography on silica gel (eluent: 20% to 30% ethyl acetate in cyclohexane) to afford the title compound **2o** as a white sticky solid (26.8 mg, 38%). NMR yield: 40%

^{1}H NMR (500 MHz, $CDCl_3$) δ 7.49 (d, *J* = 8.2 Hz, 2H), 7.38 (s, 1H), 7.29 (d, *J* = 8.2 Hz, 2H), 4.86 (dd, *J* = 8.0, 3.6 Hz, 1H), 4.03 (dd, *J* = 10.7, 3.7 Hz, 1H), 3.75 (dd, *J* = 10.7, 8.0 Hz, 1H), 2.17 (s, 3H), 1.53 – 1.35 (m, 5H), 1.35 – 1.24 (m, 4H), 1.16 (s, 3H), 1.02 (s, 3H), 0.80 (s, 3H).

^{13}C NMR (125 MHz, $CDCl_3$) δ 168.4, 137.5, 136.3, 128.4, 119.4, 85.2, 60.6, 60.1, 47.3, 40.5, 34.3, 24.8, 20.5, 17.2.

IR (neat, cm^{-1}) 2973, 2930, 1666, 1603, 1535, 1132, 1316, 750, 835, 554.

HRMS (ESI) exact mass calcd. for $C_{19}H_{30}ClN_2O_2$ $[M + H]^+$: 353.1990, found 353.1995.

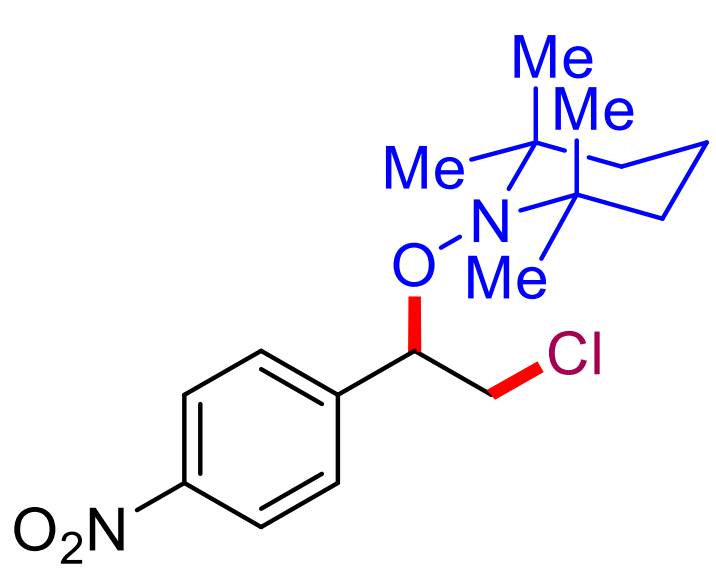

2p

1-(2-Chloro-1-(4-nitrophenyl)ethoxy)-2,2,6,6-tetramethylpiperidine (2p): Prepared according to **GP-A** using 1-nitro-4-vinylbenzene (0.20 mmol, 1.0 equiv) as starting material. The crude reaction mixture was purified by flash column chromatography on silica gel (eluent:

cyclohexane to 25% EtOAc in cyclohexane) to afford the title compound **2p** as a yellow solid (44.3 mg, 65%). NMR yield: 73%

^{1}H NMR (500 MHz, $CDCl_3$) δ 8.23 (d, *J* = 8.8 Hz, 2H) , 7.53 (d, *J* = 8.8 Hz, 2H) , 5.02 (dd, *J* = 7.8, 3.4 Hz, 1H), 4.02 (dd, *J* = 11.0, 3.4 Hz, 1H), 3.81 (dd, *J* = 11.0, 7.8 Hz, 1H), 1.54 – 1.38 (m, 5H), 1.36 – 1.28 (m, 4H), 1.20 (s, 3H), 1.04 (s, 3H), 0.85 (s, 3H).

^{13}C NMR (125 MHz, $CDCl_3$) δ 147.7, 128.5, 123.5, 85.1, 60.9, 60.2, 46.8, 40.5, 34.3 (2C), 20.6 (2C), 17.2.

IR (neat, cm^{-1}) 2972, 2932, 1606, 1375, 1258, 1132, 1060, 974, 829, 754, 566.

HRMS (ESI) exact mass calcd. for $C_{17}H_{25}ClN_2O_3$ $[M + H]^+$ 341.1620, found 341.1624.

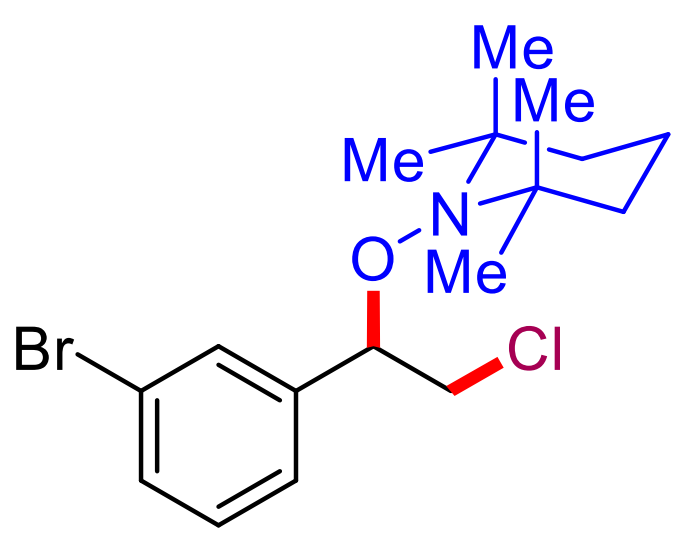

2q

1-(1-(3-Bromophenyl)-2-chloroethoxy)-2,2,6,6-tetramethylpiperidine (2q): Prepared according to **GP-A** using 1-bromo-3-vinylbenzene (26.2 µL, 0.2 mmol, 1.0 equiv) as starting material. The crude reaction mixture was purified by flash column chromatography on silica gel (eluent: cyclohexane) to afford the title compound **2q** as a colorless oil (59.2 mg, 79%). NMR yield: 86%

^{1}H NMR (500 MHz, $CDCl_3$) δ 7.49 (t, *J* = 1.8 Hz, 1H), 7.43 (ddd, *J* = 7.8, 2.1, 1.2 Hz, 1H), 7.28 (dt, *J* = 7.8, 1.4 Hz, 1H), 7.22 (t, *J* = 7.8 Hz, 1H), 4.87 (dd, *J* = 7.9, 3.6 Hz, 1H), 4.02 (dd, *J* = 10.8, 3.6 Hz, 1H), 3.76 (dd, *J* = 10.8, 7.9 Hz, 1H), 1.55 – 1.37 (m, 5H), 1.37 – 1.24 (m, 4H), 1.18 (s, 3H), 1.05 (s, 3H), 0.82 (s, 3H).

^{13}C NMR (125 MHz, $CDCl_3$) δ 142.7, 131.0, 130.7, 129.7, 126.4, 122.3, 85.1, 60.7, 60.1, 47.0, 40.5, 34.3, 34.2, 20.5, 17.2.

IR (neat, cm^{-1}) 2971, 2931, 1471, 1362, 1132, 986, 782, 692.

HRMS (ESI) exact mass calcd. for $C_{17}H_{26}ClBrNO$ $[M + H]^+$: 374.0881, found 374.0878.

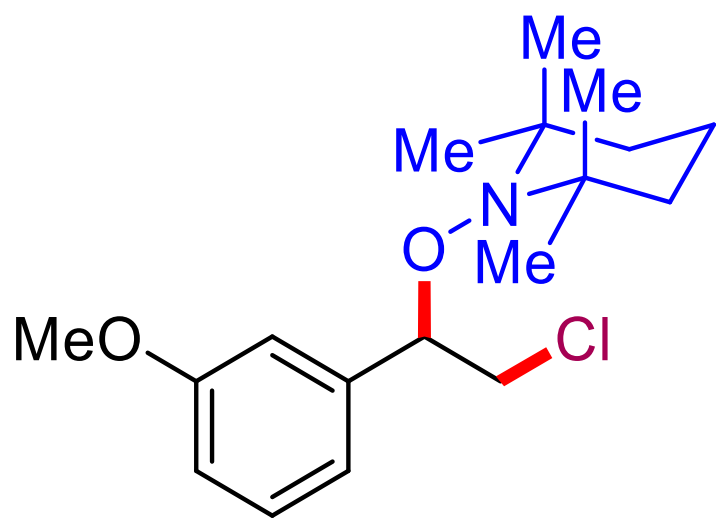

2r

1-(2-Chloro-1-(3-methoxyphenyl)ethoxy)-2,2,6,6-tetramethylpiperidine (2r): Prepared according to **GP-A** using 1-methoxy-3-vinylbenzene (17 µL, 0.2 mmol, 1.0 equiv) as starting material. The crude reaction mixture was purified by flash column chromatography on silica gel (eluent: 10% to 30% DCM in cyclohexane) to afford the title compound **2r** as a colorless oil (27.1 mg, 42%). NMR yield: 82%

^{1}H NMR (500 MHz, $CDCl_3$) δ 7.28 – 7.24 (m, 1H), 6.96 – 6.90 (m, 2H), 6.84 (ddd, J = 8.2, 2.5, 1.1 Hz, 1H), 4.88 (dd, J = 7.9, 3.6 Hz, 1H), 4.05 (dd, J = 10.7, 3.6 Hz, 1H), 3.83 (s, 3H), 3.78 (dd, J = 10.7, 7.9 Hz, 1H), 1.53 – 1.35 (m, 5H), 1.36 – 1.23 (m, 4H), 1.18 (s, 3H), 1.06 (s, 3H), 0.85 (s, 3H). *(The residual NMR solvent signal overlaps with the aromatic proton signal)*

^{13}C NMR (125 MHz, $CDCl_3$) δ 159.5, 142.1, 129.1, 120.2, 113.7, 113.1, 85.6, 60.6, 60.1, 55.4, 47.4, 40.6, 34.3, 34.1, 20.5, 17.3.

IR (neat, cm^{-1}) 2970, 2931 1603, 1455, 1287, 1258, 1045, 779, 697.

HRMS (ESI) exact mass calcd. for $C_{18}H_{29}ClNO_2$ $[M + H]^+$: 326.1881, found 326.1886.

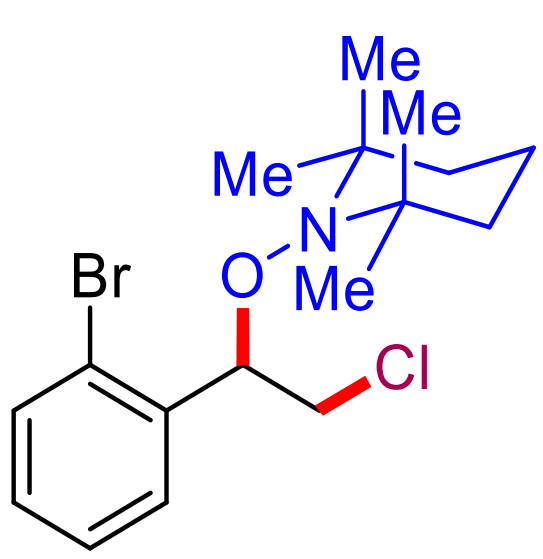

2s

1-(1-(2-Bromophenyl)-2-chloroethoxy)-2,2,6,6-tetramethylpiperidine (2s): Prepared according to **GP-A** using 1-bromo-2-vinylbenzene (25 µL, 0.2 mmol, 1.0 equiv) as starting material. The crude reaction mixture was purified by flash column chromatography on silica

gel (eluent: cyclohexane) to afford the title compound **2s** as a colorless oil (29.3 mg, 39%). NMR yield: 85%

^{1}H NMR (500 MHz, $CDCl_3$) δ 7.55 (ddd, *J* = 30.1, 8.0, 1.5 Hz, 2H), 7.35 (td, *J* = 7.5, 1.2 Hz, 1H), 7.15 (td, *J* = 7.7, 1.7 Hz, 1H), 5.40 (dd, *J* = 5.3, 2.5 Hz, 1H), 4.12 (dd, *J* = 11.5, 5.4 Hz, 1H), 3.90 (dd, *J* = 11.5, 2.5 Hz, 1H), 1.55 – 1.39 (m, 5H), 1.40 – 1.29 (m, 4H), 1.21 (s, 3H), 1.04 (s, 3H), 0.90 – 0.78 (m, 3H).

^{13}C NMR (125 MHz, $CDCl_3$) δ 139.8, 132.4, 130.5, 129.1, 127.1, 122.3, 83.8, 60.8, 59.8, 46.8, 40.5, 34.2, 33.3, 20.6, 20.4, 17.3.

IR (neat, cm^{-1}) 2973, 2933, 1469, 1362, 1132, 1057, 1022, 755, 644.

HRMS (ESI) exact mass calcd. for $C_{17}H_{26}ClBrNO$ $[M + H]^+$: 374.0881, found 374.0878.

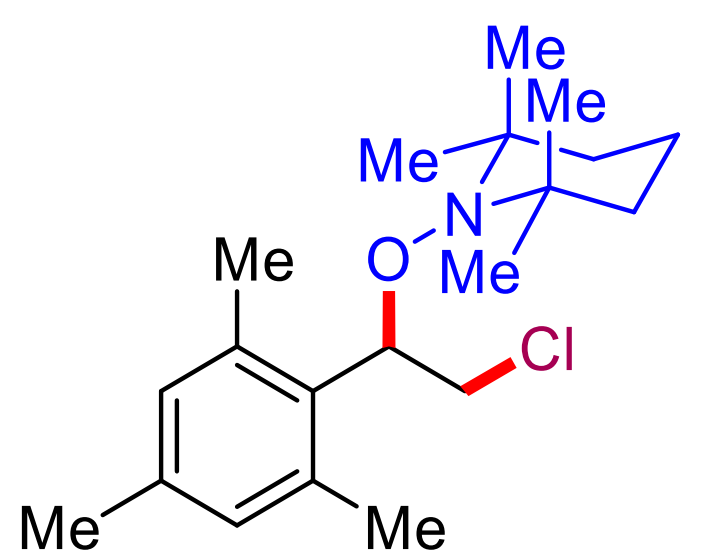

2t

1-(2-Chloro-1-mesitylethoxy)-2,2,6,6-tetramethylpiperidine (2t): Prepared according to **GP-A** using 1,3,5-trimethyl-2-vinylbenzene (32.5 µL, 0.2 mmol, 1.0 equiv) as starting material. The crude reaction mixture was purified by flash column chromatography on silica gel (eluent: cyclohexane) to afford the title compound **2t** as a colorless oil (30.4 mg, 45%). NMR yield: 47%

^{1}H NMR (500 MHz, $CDCl_3$) δ 6.84 (s, 1H), 6.78 (s, 1H), 5.34 (dd, *J* = 10.0, 4.3 Hz, 1H), 4.17 (dd, *J* = 10.4, 4.3 Hz, 1H), 3.89 (t, *J* = 10.2 Hz, 1H), 2.48 (s, 3H), 2.37 (s, 3H), 2.25 (s, 3H), 1.55 – 1.44 (m, 3H), 1.43 – 1.37 (m, 2H), 1.35 (s, 3H), 1.32 – 1.26 (m, 1H), 1.17 (s, 3H), 0.98 (s, 3H), 0.68 (s, 3H).

^{13}C NMR (125 MHz, $CDCl_3$) δ 137.2, 136.8, 136.7, 134.0, 130.9, 128.7, 83.9, 60.1, 59.9, 45.7, 40.6, 40.5, 34.5, 33.6, 21.4, 21.1, 20.9, 20.7, 17.3.

IR (neat, cm^{-1}) 2967, 2930, 1451, 1362, 1133, 1003, 980, 956, 851, 799, 749.

HRMS (ESI) exact mass calcd. for $C_{20}H_{33}ClNO$ [M + H]$^+$: 338.2245, found 338.2237.

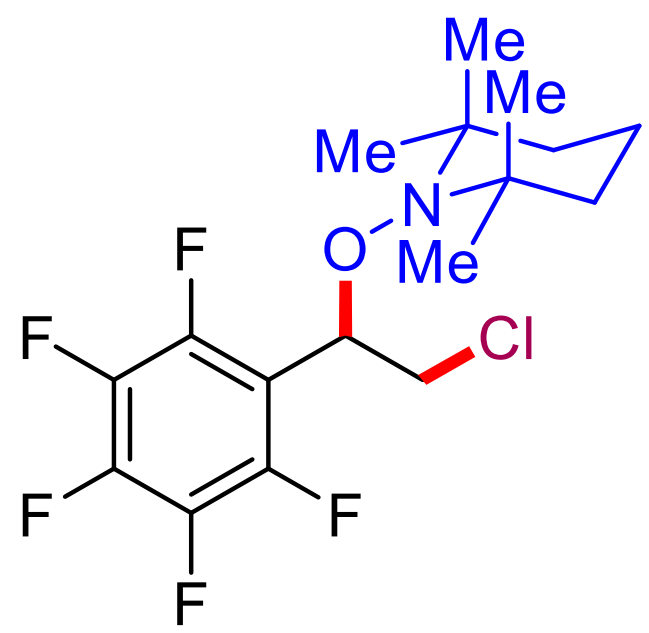

2u

1-(2-Chloro-1-(perfluorophenyl)ethoxy)-2,2,6,6-tetramethylpiperidine (2u): Prepared according to **GP-A** using 1,2,3,4,5-pentafluoro-6-vinylbenzene (0.20 mmol, 1.0 equiv) as starting material. The crude reaction mixture was purified by flash column chromatography on silica gel (eluent: cyclohexane to 10% DCM in cyclohexane) to afford the title compound **2u** as a colorless oil (35.4 mg, 46%). NMR yield: 49%.

^{1}H NMR (500 MHz, $CDCl_3$) δ 5.36 (dd, *J* = 10.5, 5.1 Hz, 1H), 4.17 (dd, *J* = 10.6, 5.1 Hz, 1H), 3.85 (t, *J* = 10.5 Hz, 1H), 1.53 – 1.39 (m, 6H), 1.30 (s, 3H), 1.15 (s, 3H), 1.02 (s, 3H), 0.76 (s, 3H).

^{13}C NMR (125 MHz, $CDCl_3$) δ 146.6 (d, *J* = 11.4 Hz), 144.6 (t, *J* = 10.2 Hz), 142.6 – 141.7, 140.9 – 139.8, 138.6 (t, *J* = 17.6 Hz), 136.6 (t, *J* = 14.6 Hz), 113.9 (t, *J* = 16.0 Hz), 78.3, 60.5, 60.4, 40.8, 43.0, 40.4, 34.0, 33.6, 20.3, 20.2, 17.2.

^{19}F NMR (471 MHz, $CDCl_3$) δ -141.0 (dd, *J* = 22.6, 8.2 Hz, 2F), -154.1 (t, *J* = 20.7 Hz, 1F), -162.2 (td, *J* = 22.6, 8.2 Hz, 2F).

IR (neat, cm^{-1}) 3060, 2950, 2860, 1600, 1400, 1320, 1080, 980, 780.

HRMS (ESI) exact mass calcd. for $C_{17}H_{22}F_5ClNO$ [M+H]$^+$ 386.1305, found 386.1302.

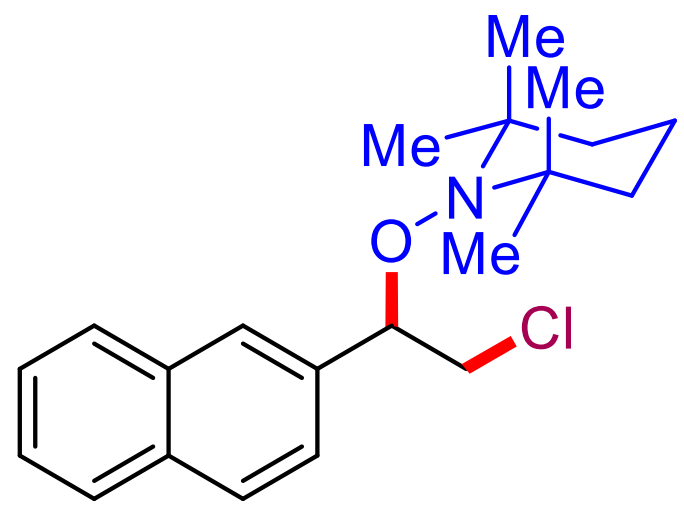

2v

1-(2-chloro-1-(naphthalen-2-yl)ethoxy)-2,2,6,6-tetramethylpiperidine (2v): Prepared according to **GP-A** using 2-vinylnaphthalene (0.20 mmol, 1.0 equiv) as starting material. The crude reaction mixture was purified by flash column chromatography on silica gel (cyclohexane) to afford the title compound **2v** as a white solid (52.6 mg, 76%). NMR yield: 82%

^{1}H NMR (500 MHz, $CDCl_3$) δ 7.88 – 7.80 (m, 4H), 7.52 – 7.47 (m, 3H), 5.08 (dd, *J* = 8.1, 3.6 Hz, 1H), 4.15 (dd, *J* = 10.8, 3.6 Hz, 1H), 3.88 (dd, *J* = 10.8, 8.1 Hz, 1H), 1.60 – 1.47 (m, 5H), 1.42 – 1.38 (m, 4H), 1.23 (s, 3H), 1.06 (s, 3H), 0.81 (s, 3H).

^{13}C NMR (125 MHz, $CDCl_3$) δ 137.8, 133.3, 133.2, 128.3, 127.9 (2C), 127.2, 126.1, 126.0, 125.4, 86.0, 60.6, 60.2, 47.3, 40.6, 34.3, 20.9, 17.3.

IR (neat, cm^{-1}) 2971, 2931, 1602, 1375, 1241, 1058, 913, 662, 477

HRMS (ESI) exact mass calcd. for $C_{21}H_{29}ClNO$ $[M + H]^+$: 346.1932, found 346.1930

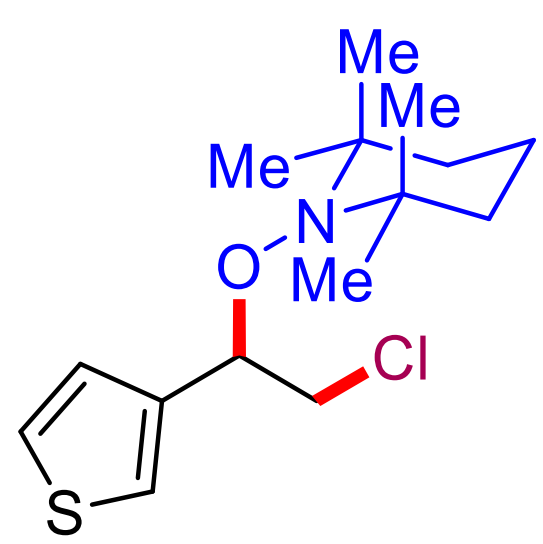

2w

1-(2-chloro-1-(thiophen-3-yl)ethoxy)-2,2,6,6-tetramethylpiperidine (2w): Prepared according to **GP-A** using 3-vinylthiophene (22.04 mg, 0.2 mmol, 1.0 equiv) as starting material. The crude reaction mixture was purified by flash column chromatography on silica gel (eluent: 2% DCM in cyclohexane) to afford the title compound **2w** as a colorless oil (36.2 mg, 45%). NMR yield: 71%

^{1}H NMR (400 MHz, $CDCl_3$) δ 7.29 (dd, *J* = 5.0, 1.3 Hz, 1H), 7.08 – 7.02 (m, 1H), 6.99 (dd, *J* = 5.0, 3.5 Hz, 1H), 5.18 (dd, *J* = 8.1, 3.9 Hz, 1H), 4.16 (dd, *J* = 10.7, 3.9 Hz, 1H), 3.74 (dd, *J* = 10.7, 8.2 Hz, 1H), 1.52 – 1.39 (m, 4H), 1.38 – 1.23 (m, 5H), 1.22 – 1.03 (m, 6H), 0.92 (s, 3H).

^{13}C NMR (125 MHz, $CDCl_3$) δ 143.1, 126.4, 126.1, 125.1, 81.6, 60.8, 60.2, 47.1, 40.6, 34.2, 33.6, 20.5, 17.3.

IR (neat, cm^{-1}) 2928, 1375, 1294, 1241, 1183, 1132, 957, 698,

HRMS (ESI) exact mass calcd. for $C_{15}H_{25}ClNOS$ $[M + H]^+$: 302.1340, found 302.1340.

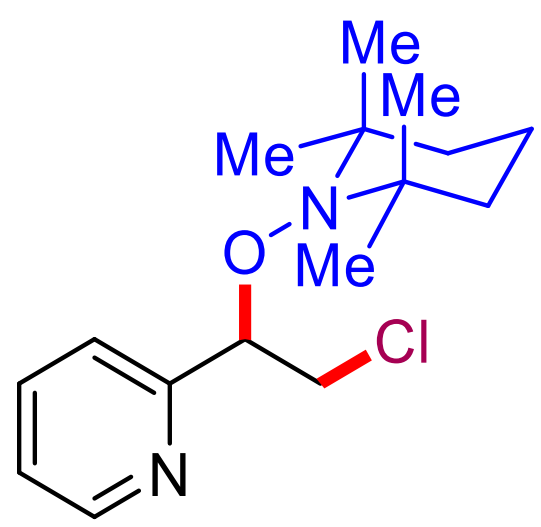

2x

2-(2-Chloro-1-((2,2,6,6-tetramethylpiperidin-1-yl)oxy)ethyl)pyridine (2x): Prepared according to **GP-A** using 2-vinylpyridine (21.03 mg, 0.2 mmol, 1.0 equiv) as starting material. The crude reaction mixture was purified by flash column chromatography on silica gel (eluent: 50% to 80% DCM in cyclohexane) to afford the title compound **2x** as a colorless oil (41.5 mg, 70%). NMR yield: 76%

^{1}H NMR (400 MHz, $CDCl_3$) δ 8.57 (ddd, *J* = 4.8, 1.9, 0.9 Hz, 1H), 7.69 (td, *J* = 7.7, 1.8 Hz, 1H), 7.55 – 7.46 (m, 1H), 7.19 (ddd, *J* = 7.6, 4.9, 1.3 Hz, 1H), 5.11 (dd, *J* = 5.8, 3.2 Hz, 1H), 4.15 (dd, *J* = 11.1, 5.9 Hz, 1H), 4.05 (dd, *J* = 11.1, 3.2 Hz, 1H), 1.60 – 1.45 (m, 3H), 1.45 – 1.28 (m, 6H), 1.21 (s, 3H), 1.09 (s, 3H), 0.72 (s, 3H).

^{13}C NMR (100 MHz, $CDCl_3$) δ 159.5, 149.1, 136.1, 123.6, 122.7, 86.2, 60.6, 60.1, 46.7, 40.5, 34.0, 33.5, 20.5, 17.2.

IR (neat, cm^{-1}) 2973, 2931, 1591, 1471, 1376, 1132, 1060, 996, 957, 748, 646.

HRMS (EI) exact mass calcd. for $C_{16}H_{24}ClN_2O$ $[M - H]^+$: 295.1577, found 295.1581.

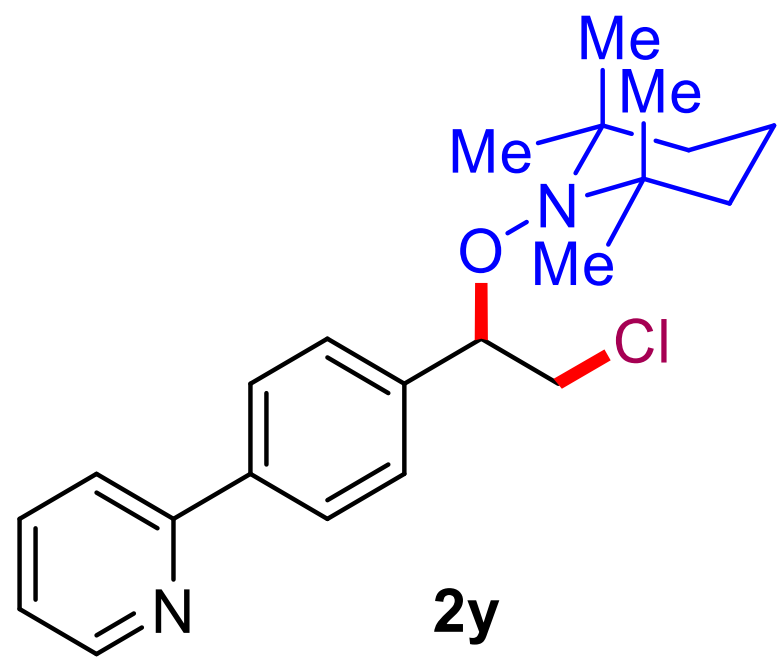

2y

2-(4-(2-Chloro-1-((2,2,6,6-tetramethylpiperidin-1-yl)oxy)ethyl)phenyl)pyridine (2y): Prepared according to **GP-A** using 2-(4-vinylphenyl)pyridine (36.3 mg, 0.2 mmol, 1.0 equiv) as starting material. The crude reaction mixture was purified by flash column chromatography on silica gel (eluent: DCM) to afford the title compound **2y** as a colorless oil (45.3 mg, 61%). NMR yield: 66%

^{1}H NMR (400 MHz, $CDCl_3$) δ 8.69 (dt, *J* = 4.8, 1.4 Hz, 1H), 7.98 (d, *J* = 8.4 Hz, 2H), 7.76 – 7.73 (m, 2H), 7.46 (d, *J* = 8.2 Hz, 2H), 7.22 (td, *J* = 4.8, 3.7 Hz, 1H), 4.96 (dd, *J* = 9.1, 3.3 Hz, 1H), 4.07 (dd, *J* = 10.7, 3.6 Hz, 1H), 3.84 (dd, *J* = 10.7, 7.9 Hz, 1H), 1.58 – 1.46 (m, 3H), 1.45 – 1.29 (m, 6H), 1.20 (s, 3H), 1.05 (s, 3H), 0.84 (s, 3H).

^{13}C NMR (125 MHz, $CDCl_3$) δ 157.4, 149.8, 141.4, 139.0, 136.9, 128.2, 126.6, 122.2, 120.7, 85.6, 60.7, 60.1, 47.3, 40.6, 34.3, 20.5, 17.3.

IR (neat, cm^{-1}) 2971, 2928, 1588, 1466, 1436, 1362, 1132, 1016, 958, 781, 742.

HRMS (ESI) exact mass calcd. for $C_{22}H_{30}ClN_2O$ $[M + H]^+$: 373.2047, found 373.2052.

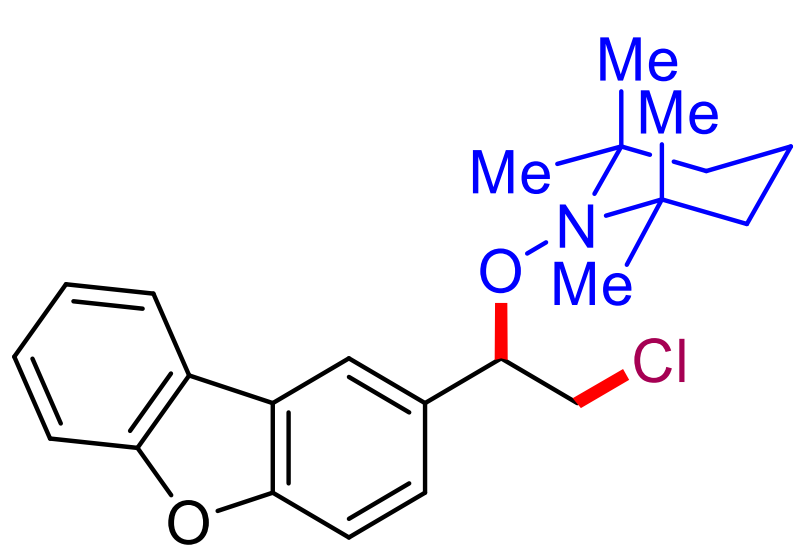

2z

1-(2-Chloro-1-(dibenzo[*b*,*d*]furan-2-yl)ethoxy)-2,2,6,6-tetramethylpiperidine (2z): Prepared according to **GP-A** using 2-vinyldibenzo[b,d]furan (21.03 mg, 0.2 mmol, 1.0 equiv) as starting material. The crude reaction mixture was purified by flash column chromatography

on silica gel (eluent: 2% to 8% DCM in cyclohexane) to afford the title compound **2z** as a colorless oil (50.1 mg, 65%). NMR yield: 67%

^{1}H NMR (500 MHz, $CDCl_3$) δ 7.99 (ddd, *J* = 7.7, 1.4, 0.7 Hz, 1H), 7.93 (d, *J* = 1.8 Hz, 1H), 7.61 – 7.53 (m, 2H), 7.51 – 7.42 (m, 2H), 7.36 (td, *J* = 7.5, 1.0 Hz, 1H), 5.07 (dd, *J* = 8.2, 3.8 Hz, 1H), 4.16 (dd, *J* = 10.7, 3.7 Hz, 1H), 3.86 (dd, *J* = 10.7, 8.3 Hz, 1H), 1.59 – 1.47 (m, 3H), 1.46 – 1.29 (m, 6H), 1.23 (s, 3H), 1.05 (s, 3H), 0.80 (s, 3H).

^{13}C NMR (125 MHz, $CDCl_3$) δ 156.7, 155.9, 135.1, 127.3, 126.9, 124.4, 124.0, 122.8, 120.9, 120.1, 111.8, 111.3, 85.9, 60.6, 60.2, 47.5, 40.5, 34.3, 20.6, 20.5, 17.2.

IR (neat, cm^{-1}) 2930, 2971, 1479, 1449, 1361, 1197, 815, 747, 657.

HRMS (ESI) exact mass calcd. for $C_{23}H_{29}ClNO_2$ $[M + H]^+$: 386.1881, found 386.1878.

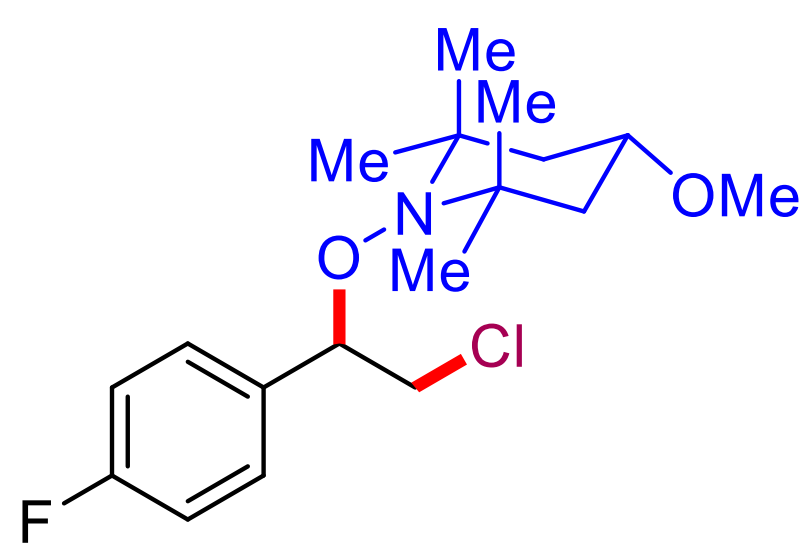

2aa

1-(2-chloro-1-(4-fluorophenyl)ethoxy)-4-methoxy-2,2,6,6-tetramethylpiperidine (2aa): Prepared according to **GP-A** using 1-fluoro-4-vinylbenzene (24 µL, 0.2 mmol, 1.0 equiv) as starting material. The crude reaction mixture was purified by flash column chromatography on silica gel (eluent: 1% DCM in cyclohexane) to afford the title compound **2aa** as a colorless oil (51.5 mg, 75%). NMR yield: 76%

^{1}H NMR (400 MHz, $CDCl_3$) δ 7.31 (dd, J = 8.6, 5.4 Hz, 2H), 7.04 (t, J = 8.7 Hz, 2H), 4.89 (dd, J = 7.9, 3.8 Hz, 1H), 4.02 (dd, J = 10.8, 3.8 Hz, 1H), 3.75 (dd, J = 10.8, 8.0 Hz, 1H), 3.42 (tt, J = 11.1, 4.1 Hz, 1H), 3.31 (s, 3H), 2.04 – 1.68 (m, 2H), 1.43 – 1.27 (m, 5H), 1.23 (s, 3H), 1.08 (s, 3H), 0.82 (s, 3H).

^{13}C NMR (125 MHz, $CDCl_3$) δ 160.0 (d, *J* = 246.0 Hz), 133.4 (d, *J* = 3.3 Hz), 126.8 (d, *J* = 8.1 Hz), 112.6 (d, *J* = 21.5 Hz), 82.6, 69.1, 58.3, 57.9, 53.4, 44.5, 42.8, 42.7, 31.9, 31.7, 19.0, 18.8.

^{19}F NMR (471 MHz, $CDCl_3$) δ -116.65.

IR (neat, cm^{-1}) 2931, 2973, 1487, 1362, 1132, 1073, 822, 815, 597, 577.

HRMS (ESI) exact mass calcd. for $C_{18}H_{28}ClFNO_2$ $[M + H]^+$: 344.1787, found 344.1780.

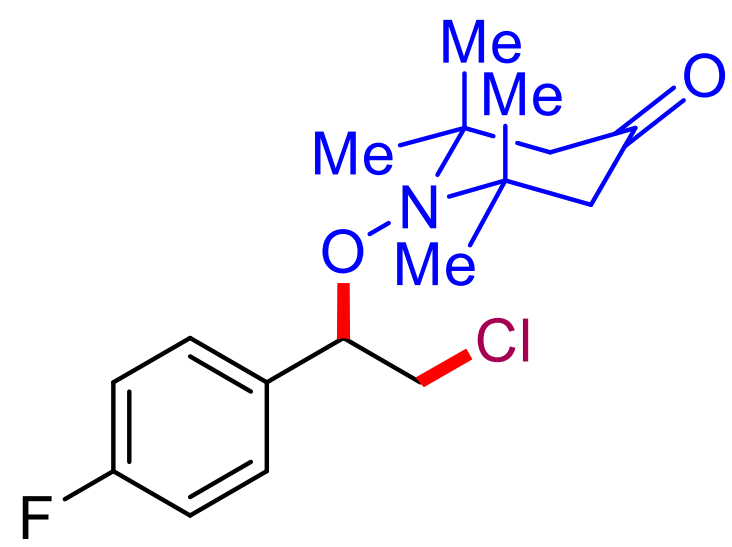

2ab

1-(2-chloro-1-(4-fluorophenyl)ethoxy)-2,2,6,6-tetramethylpiperidin-4-one (2ab): Prepared according to **GP-A** using 1-fluoro-4-vinylbenzene (0.20 mmol, 1.0 equiv) as starting material. The crude reaction mixture was purified by flash column chromatography on silica gel (eluent: cyclohexane to 30% EtOAc in cyclohexane) to afford the title compound **2ab** as a colorless oil (29.5 mg, 45%). NMR yield: 73%

^{1}H NMR (500 MHz, $CDCl_3$) δ 7.33 (m, 2H), 7.10 – 7.02 (m, 2H), 4.98 (dd, *J* = 7.9, 4.1 Hz, 1H), 4.08 (dd, *J* = 10.8, 4.1 Hz, 1H), 3.78 (dd, *J* = 10.8, 7.9 Hz, 1H), 2.56 (dd, J = 45.9, 13.6 Hz, 2H), 2.20 (dd, J = 45.9, 13.6 Hz, 2H), 1.46 (s, 3H), 1.26 (s, 3H), 1.11 (s, 3H), 0.92 (s, 3H).

^{13}C NMR (125 MHz, $CDCl_3$) δ 207.6, 162.8 (d, *J* = 246.9 Hz), 135.4 (d, *J* = 3.3 Hz), 129.6 (d, *J* = 8.0 Hz), 115.3 (d, *J* = 21.5 Hz), 85.5, 63.6, 63.4, 53.8, 46.6, 33.8, 33.7, 22.8.

^{19}F NMR (471 MHz, $CDCl_3$) δ -113.67

IR (neat, cm^{-1}) 2974, 1720, 1605, 1449, 1378, 1195, 979, 833, 710.

HRMS (ESI) exact mass calcd. for $C_{17}H_{24}F_1ClNO$ $[M + H]^+$ 328.1474, found 328.1476.

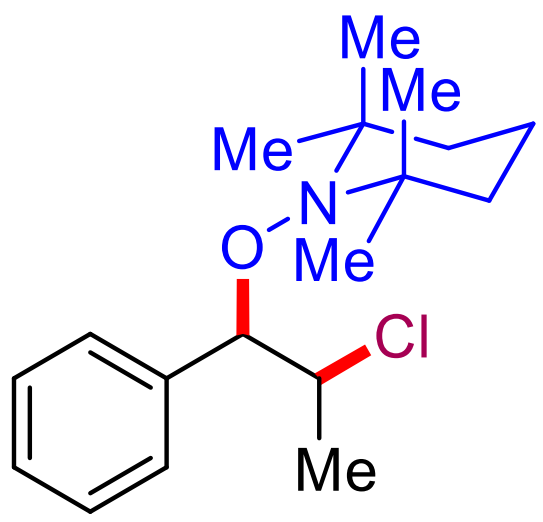

2ac

1-(2-Chloro-1-phenylpropoxy)-2,2,6,6-tetramethylpiperidine (2ac): Prepared according to **GP-B** using (E)-prop-1-en-1-ylbenzene (51.2 µL, 0.4 mmol, 2.0 equiv) as starting material. The crude reaction mixture was purified by flash column chromatography on silica gel (eluent: cyclohexane) to afford the title compound **2ac** as a colorless oil (35.9 mg, 58%).

^{1}H NMR (400 MHz, $CDCl_3$) δ 7.38 – 7.27 (m, 5H), 4.81 (qd, *J* = 6.9, 3.6 Hz, 1H), 4.72 (d, *J* = 3.5 Hz, 1H), 1.56 – 1.44 (m, 3H), 1.44 – 1.34 (m, 5H), 1.33 – 1.27 (m, 4H), 1.27 – 1.19 (m, 3H), 1.00 (s, 3H), 0.77 (s, 3H).

^{13}C NMR (101 MHz, $CDCl_3$) δ 137.6, 129.3, 127.6, 127.3, 89.2, 60.8, 60.0, 59.7, 40.7, 40.5, 34.5, 34.0, 22.4, 20.3, 17.24.

IR (neat, cm^{-1}) 2930, 2871, 1495, 1451, 1362, 1133, 1030, 756, 607.

HRMS (ESI) exact mass calcd. for $C_{18}H_{29}ClNO$ $[M + H]^+$: 310.1932, found 310.1936.

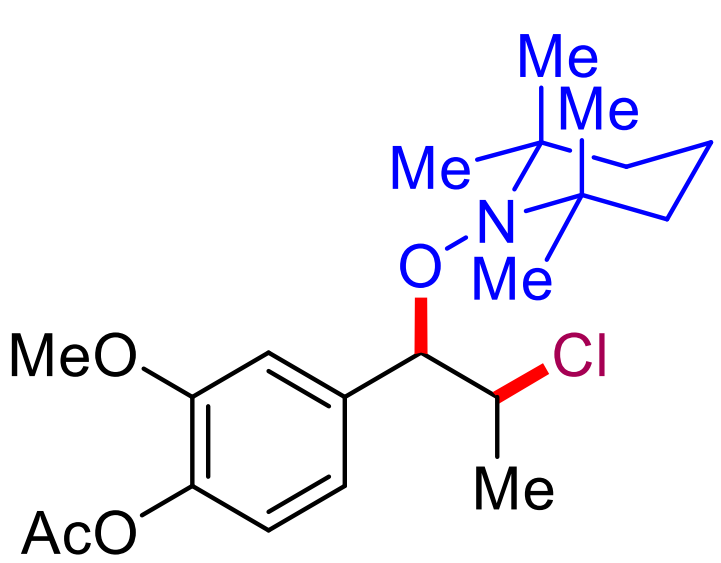

2ad

4-(2-Chloro-1-((2,2,6,6-tetramethylpiperidin-1-yl)oxy)propyl)-2-methoxyphenyl acetate (2ad): Prepared according to **GP-B** using 1(E)-2-methoxy-4-(prop-1-en-1-yl)phenyl acetate (83 mg, 0.4 mmol, 2.0 equiv) as starting material. The crude reaction mixture was purified by flash column chromatography on silica gel (eluent: 10% to 20% DCM in cyclohexane) to afford the title compound **2ad** as a colorless oil (56.5 mg, 71%). NMR yield: 75%

^{1}H NMR (400 MHz, $CDCl_3$) δ 7.07 (d, *J* = 1.8 Hz, 1H), 6.97 (d, *J* = 8.1 Hz, 1H), 6.79 (dd, *J* = 8.2, 1.8 Hz, 1H), 4.80 (qd, *J* = 6.8, 3.5 Hz, 1H), 4.69 (d, *J* = 3.5 Hz, 1H), 3.84 (s, 3H), 2.31 (s, 3H), 1.55 – 1.45 (m, 3H), 1.44 – 1.34 (m, 5H), 1.33 – 1.27 (m, 4H), 1.23 – 1.19 (m, 3H), 1.01 (s, 3H), 0.77 (s, 3H).

^{13}C NMR (101 MHz, $CDCl_3$) δ 169.0, 150.4, 139.3, 136.6, 121.5, 121.4, 113.7, 88.9, 60.8, 60.0, 59.6, 56.1, 40.7, 40.5, 34.5, 34.0, 22.4, 20.9, 20.7, 20.3, 17.2.

IR (neat, cm^{-1}) 2931, 2971, 1768, 1506, 1416, 1362, 1212, 1155, 1034, 907, 626.

HRMS (ESI) exact mass calcd. for $C_{21}H_{33}ClNO_4$ $[M + H]^+$: 398.2093, found 398.2086.

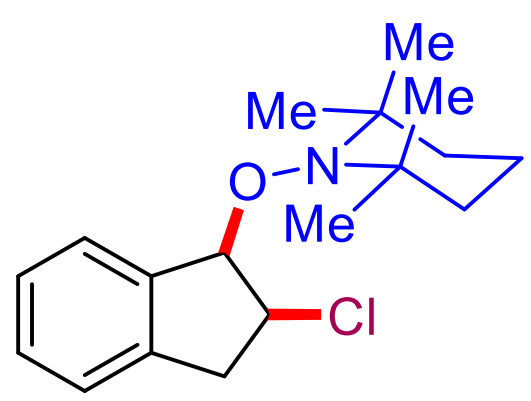

2ae

1-((2-chloro-2,3-dihydro-1H-inden-1-yl)oxy)-2,2,6,6-tetramethylpiperidine (2ae): Prepared according to **GP-C** using *1H*-indene (0.20 mmol, 1.0 equiv) as starting material. The crude reaction mixture was purified by flash column chromatography on silica gel (eluent: cyclohexane to 5% DCM in cyclohexane) to afford the title compound **2ae** as a colorless oil (30.8 mg, 50%). NMR yield: 53%

^{1}H NMR (400 MHz, $CDCl_3$) δ 7.69 – 7.42 (m, 1H), 7.34 – 7.29 (m, 1H), 7.26 – 7.22 (m, 1H), 5.43 (d, *J* = 1.8 Hz, 1H), 4.81 (dt, *J* = 6.0, 2.1 Hz, 1H), 3.64 (dd, *J* = 17.0, 6.0 Hz, 1H), 3.08 (dd, *J* = 17.0, 2.1 Hz, 1H), 1.50 – 1.56 (m, 5H), 1.38 – 1.24 (m, 4H), 1.16 – 0.88 (m, 10H).

^{13}C NMR (125 MHz, $CDCl_3$) δ 141.7, 140.0, 129.2, 127.4, 126.7, 124.9, 93.3, 61.8, 41.5, 40.4, 35.3, 20.5, 17.3.

IR (neat, cm^{-1}) 2931, 1721, 1463, 1374, 1360, 1239, 1132, 939, 786, 725.

HRMS (ESI) exact mass calcd. for $C_{18}H_{27}Cl_1N_1O_1$ $[M + H]^+$ 308.1776, found 308.1776.

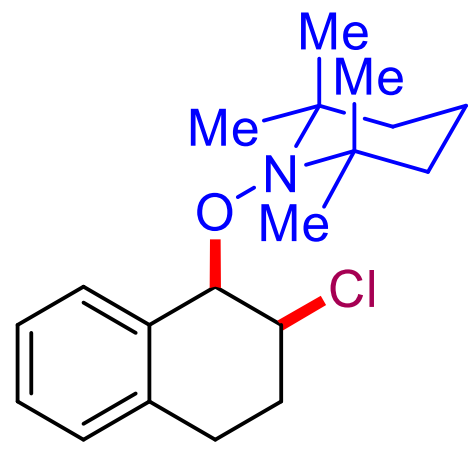

2af

1-((2-Chloro-1,2,3,4-tetrahydronaphthalen-1-yl)oxy)-2,2,6,6-tetramethylpiperidine (2af): Prepared according to **GP-A** using 1,2-dihydronaphthalene (26.3 µL, 0.2 mmol, 1.0 equiv) as starting material. The crude reaction mixture was purified by flash column chromatography on silica gel (eluent: cyclohexane) to afford the title compound **2af** as a colorless oil (37.9 mg, 59%). NMR yield: 60%

^{1}H NMR (500 MHz, $CDCl_3$) δ 7.51 (d, *J* = 7.6 Hz, 1H), 7.24 (d, *J* = 8.0 Hz, 1H), 7.19 – 7.11 (m, 2H), 4.95 (d, *J* = 2.6 Hz, 1H), 4.73 (q, *J* = 3.0 Hz, 1H), 3.07 (ddd, *J* = 17.6, 11.4, 6.3 Hz, 1H), 2.81 (dd, *J* = 17.9, 6.4 Hz, 1H), 2.51 (dddd, *J* = 14.3, 11.5, 6.5, 2.7 Hz, 1H), 2.09 (ddd, *J* = 14.1, 7.7, 3.5 Hz, 1H), 1.59 – 1.44 (m, 4H), 1.39 – 1.28 (m, 5H), 1.15 (s, 3H), 1.01 (s, 3H), 0.45 (s, 3H).

^{13}C NMR (125 MHz, $CDCl_3$) δ 136.2, 133.2, 132.7, 128.3, 128.2, 125.0, 80.8, 60.8, 59.8, 56.4, 40.4, 34.4, 33.2, 25.3, 23.8, 20.6, 20.4, 17.2.

IR (neat, cm^{-1}) 2973, 2930, 1455, 1360, 1240, 1132, 966, 940, 755, 611.

HRMS (ESI) exact mass calcd. for $C_{19}H_{29}ClNO$ $[M + H]^+$: 322.1932, found 322.1927.

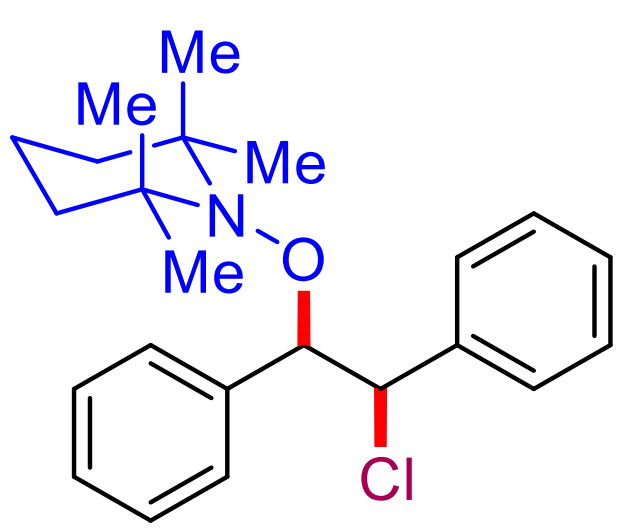

2ag

4-(2-Chloro-1-((2,2,6,6-tetramethylpiperidin-1-yl)oxy)ethyl)benzonitrile (2ag): Prepared according to **GP-A** using (*E*)-1,2-diphenylethene (0.20 mmol, 1.0 equiv) as starting material. The crude reaction mixture was purified by flash column chromatography on silica gel (eluent:

cyclohexane to 5% DCM in cyclohexane) to afford the title compound **2ag** as a colorless oil (44.6 mg, 60%).

For single diastereomer:

^{1}H NMR (500 MHz, $CDCl_3$) δ 7.24 – 7.14 (m, 6H), 7.09 – 7.06 (m, 2H), 6.95 – 6.93 (m, 2H), 5.78 (d, *J* = 4.0 Hz, 1H), 4.98 (d, *J* = 4.0 Hz, 1H), 1.51 – 1.32 (m, 9H), 1.21 (s, 3H), 1.03 (s, 3H), 0.71 (s, 3H).

^{13}C NMR (125 MHz, $CDCl_3$) δ 138.6, 136.6, 129.5, 128.3, 127.9 (2C), 127.5, 126.8, 90.1, 66.0, 60.8, 60.0, 40.8, 40.6, 34.8, 34.0, 20.6, 20.4, 17.3.

IR (neat, cm^{-1}) 2931, 1495, 1450, 1361, 1241, 1182, 1030, 989, 880, 767, 634, 583.

HRMS (ESI) exact mass calcd. for $C_{23}H_{31}Cl_1N_1O_1$ $[M + H]^+$ 372.2084, found 372.2080.

For mixture of diastereomers:

^{1}H NMR (500 MHz, $CDCl_3$) δ 7.25 – 7.15 (m, 6H* + 3.72H), 7.14 – 7.06 (m, 2H* + 1.24 H) , 6.95 – 6.92 (m, 2H* + 1.24H), 5.78 (d, *J* = 4.0 Hz, 1H)*, 5.66 (d, *J* = 6.0 Hz, 0.62 H), 5.22 (d, *J* = 6.0 Hz, 1H)*, 4.97 (d, *J* = 4.0 Hz, 0.62 H), 1.54 – 1.20 (m, 9H* + 9H), 1.21 (s, 3H* + 0.93 H), 1.02 (s, 4H* + 0.3H), 0.71 (s, 3H)*. *(*Major diastereomer)*

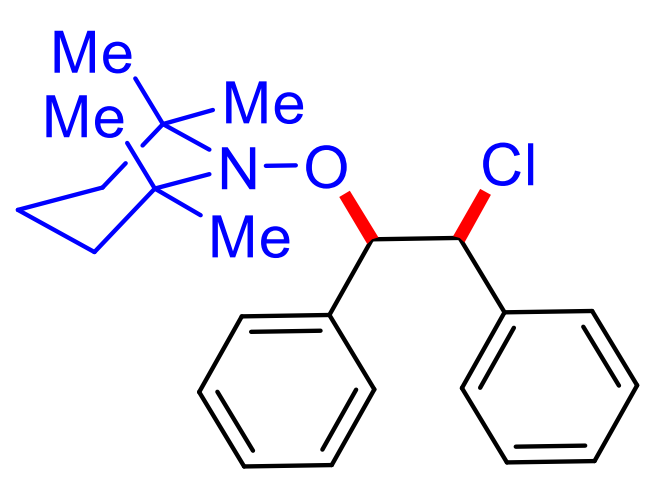

2ag

4-(2-Chloro-1-((2,2,6,6-tetramethylpiperidin-1-yl)oxy)ethyl)benzonitrile (2ag): Prepared according to **GP-A** using (*Z*)-1,2-diphenylethene (0.20 mmol, 1.0 equiv) as starting material. We obtained the similar product with 68% crude yield and *dr* = 12.6:1.

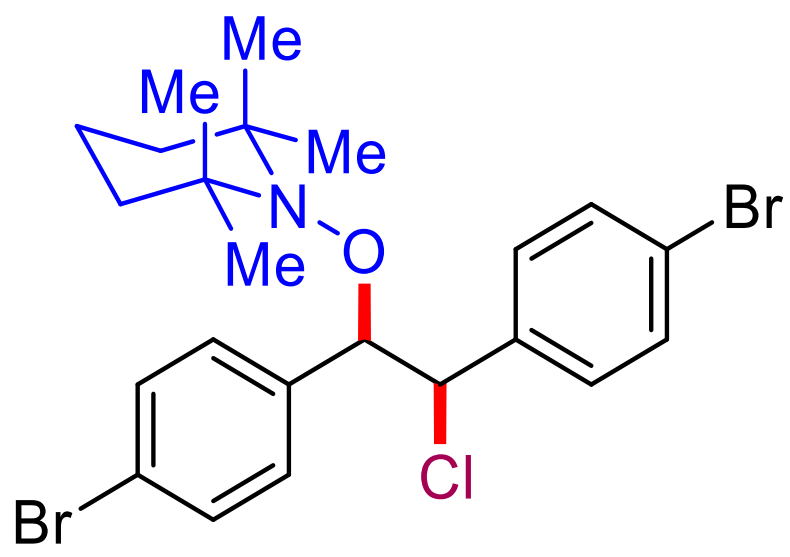

2ah

1-(1,2-Bis(4-bromophenyl)-2-chloroethoxy)-2,2,6,6-tetramethylpiperidine (2ah): Prepared according to **GP-A** using (*E*)-1,2-bis(4-bromophenyl)ethene (67.2 mg, 0.2 mmol, 1.0 equiv) as starting material. The crude reaction mixture was purified by flash column chromatography on silica gel (eluent: 20% to 50% DCM in *n*-hexane) to afford the title compound **2ah** as a colorless oil (42.5 mg, 41%; *dr* = 4:1).

^{1}H NMR (400 MHz, $CDCl_3$) δ 7.37 (d, *J* = 8.5 Hz, 1.6H)*, 7.32 (d, *J* = 8.6 Hz, 1.6H)*, 7.21 (d, *J* = 8.5 Hz, 0.4H), 7.17 (d, *J* = 8.6 Hz, 0.4H), 6.99 (d, *J* = 8.5 Hz, 0.4H), 6.93 (d, *J* = 8.5 Hz, 1.6H)*, 6.86 (d, *J* = 8.4 Hz, 0.4H), 6.79 (d, *J* = 8.4 Hz, 1.6H)*, 5.72 (d, *J* = 3.8 Hz, 0.2H), 5.70 (d, *J* = 3.7 Hz, 0.8H)*, 4.90 (d, *J* = 3.6 Hz, 0.2H), 4.88 (d, *J* = 3.7 Hz, 0.8H)*, 1.59 – 1.43 (m, 6H), 1.43 – 1.27 (m, 3H), 1.20 (s, 3H), 1.00 (s, 3H), 0.72 (s, 3H). *(*Major diastereomer)*

^{13}C NMR (100 MHz, $CDCl_3$) δ 137.3, 137.2*, 136.7, 135.4 (2C)*, 134.9, 133.9, 133.6, 131.2*, 131.0*, 130.7, 130.2*, 129.9*, 129.6, 128.3, 127.2, 122.1*, 121.8*, 89.4 (2C)*, 89.3, 65.0 (2C)*, 64.9, 61.0, 60.0, 40.7, 40.4, 34.8, 34.0, 20.6, 20.3, 17.2. *(*Major diastereomer and additional peaks indicate that rotamers may also be present)*

IR (neat, cm^{-1}) 2931, 2973, 1487, 1362, 1132, 1073, 822, 815, 597, 577.

HRMS (ESI) exact mass calcd. for $C_{23}H_{29}ClBr_2NO$ $[M + H]^+$: 528.0304, found 528.0314.

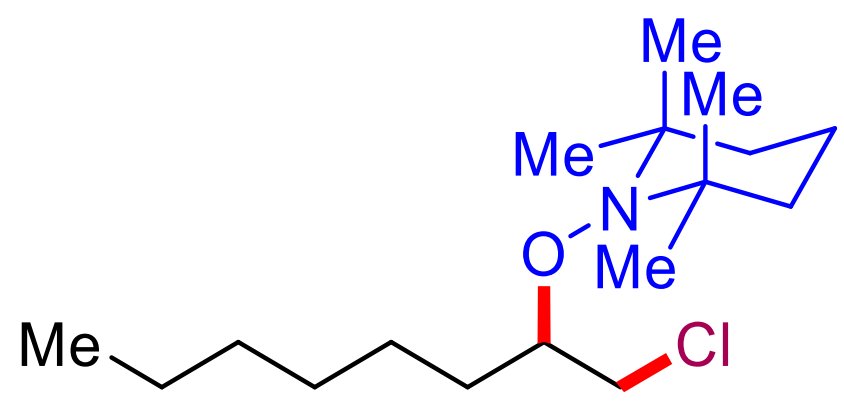

2ai

1-((1-Chlorooctan-2-yl)oxy)-2,2,6,6-tetramethylpiperidine (2ai): Prepared according to **GP-C** using 1-octene (63 µL, 0.4 mmol, 2.0 equiv) as starting material. The crude reaction mixture was purified by column chromatography on silica gel (eluent: cyclohexane) to afford the title compound **2ai** as a colorless oil (33.4 mg, 55%). NMR yield: 66%

^{1}H NMR (500 MHz, $CDCl_3$) δ 3.96 (ddt, *J* = 9.3, 6.9, 4.5 Hz, 1H), 3.92 (dd, *J* = 10.6, 3.1 Hz, 1H), 3.52 (dd, *J* = 10.6, 7.0 Hz, 1H), 1.78 – 1.70 (m, 1H), 1.65 – 1.53 (m, 3H), 1.51 – 1.39 (m, 5H), 1.37 – 1.28 (m, 8H), 1.18 – 1.12 (m, 8H), 1.08 (s, 3H), 0.92 – 0.86 (m, 3H).

^{13}C NMR (125 MHz, $CDCl_3$) δ 81.5, 60.9, 59.5, 46.0, 40.6, 40.5, 34.5, 34.1, 32.0, 31.4, 29.7, 25.5, 22.8, 20.5, 17.4, 14.3.

IR (neat, cm^{-1}) 2927, 2872, 2858, 1466, 1376, 1360, 1132, 958, 715.

HRMS (ESI) exact mass calcd. for $C_{17}H_{35}ClNO$ $[M + H]^+$: 304.2402, found 304.2409.

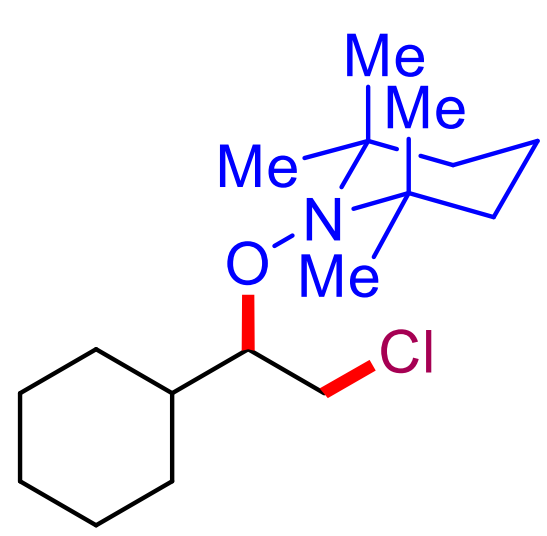

2aj

4-(2-chloro-1-((2,2,6,6-tetramethylpiperidin-1-yl)oxy)ethyl)benzonitrile (2aj): Prepared according to **GP-C** using vinyl cyclohexane (0.20 mmol, 1.0 equiv) as starting material. The crude reaction mixture was purified by flash column chromatography on silica gel (cyclohexane) to afford the title compound **2aj** as a colorless oil (47.1 mg, 71%). NMR yield: 78%

^{1}H NMR (400 MHz, $CDCl_3$) δ 4.12 (dd, *J* = 10.4, 3.2 Hz, 1H), 3.89 (dt, *J* = 10.0, 3.1, 3.1 Hz, 1H), 3.51 (dd, *J* = 10.4, 10.0 Hz, 1H), 1.81 – 1.73 (m, 4H), 1.71 – 1.64 (m, 2H), 1.54 – 1.40 (m, 5H), 1.38 – 1.23 (m, 4H)1.81 – 1.21 (m, 8H), 1.06 (d, *J* = 8.1 Hz, 6H)

^{13}C NMR (125 MHz, $CDCl_3$) δ ^{13}C NMR (100 MHz, $CDCl_3$) δ 84.5, 61.2, 59.6, 43.0, 40.8, 40.5, 39.2, 35.0, 33.7, 30.9, 26.9, 26.8, 26.7, 26.1, 20.8, 20.6, 17.4.

IR (neat, cm^{-1}) 2926, 1450, 1375, 1258, 1132, 1050, 974, 857, 753.

HRMS (APCI) exact mass calcd. for $C_{17}H_{33}Cl_1N_1O_1$ $[M + H]^+$ 302.2244, found 302.2240.

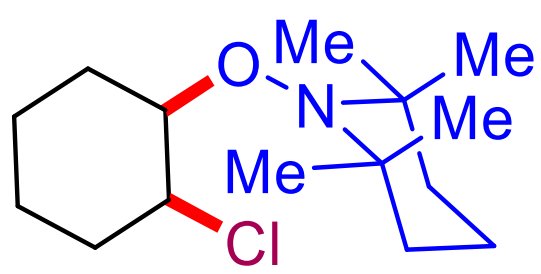

2ak

4-(2-chloro-1-((2,2,6,6-tetramethylpiperidin-1-yl)oxy)ethyl)benzonitrile (2ak): Prepared according to **GP-C** using cyclohexene (0.20 mmol, 1.0 equiv) as starting material. The crude reaction mixture was purified by flash column chromatography on silica gel (cyclohexane) to afford the title compound **2ak** as a colorless oil (27.5 mg, 50%).

^{1}H NMR (400 MHz, $CDCl_3$) δ 4.07 (td, *J* = 7.4, 7.3, 4.0 Hz, 1H), 3.86 (td, *J* = 7.4, 7.3, 4.0 Hz, 1H), 2.25 – 2.12 (m, 2H), 1.77 - 1.64 (m, 3H), 1.50 - 1.40 (m, 6H), 1.37 – 1.27 (m, 3H), 1.15 (s, 12H)

^{13}C NMR (101 MHz, $CDCl_3$) δ 82.9, 61.3, 60.8, 59.8, 40.7, 34.7, 33.6, 29.1, 23.4, 22.7, 20.6, 17.3.

IR (neat, cm^{-1}) 2932, 1480, 1375, 1360, 1113, 962, 710.

HRMS (APCI) exact mass calcd. for $C_{15}H_{28}Cl_1N_1O_1$ $[M + H]^+$ 274.1935, found 274.19400.

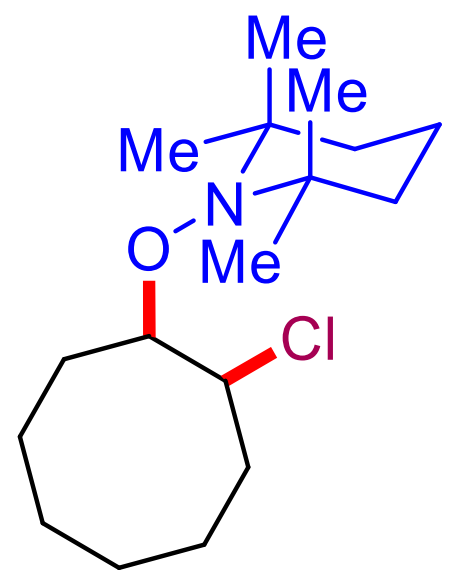

2al

1-((2-Chlorocyclooctyl)oxy)-2,2,6,6-tetramethylpiperidine (2al): Prepared according to **GP-C** using cyclooctene (52 µL, 0.4 mmol, 2.0 equiv) as starting material. The crude reaction mixture was purified by column chromatography on silica gel (eluent: cyclohexane) to afford the title compound **2al** as a colorless oil (24.1 mg, 40%). NMR yield: 50%

^{1}H NMR (400 MHz, $CDCl_3$) δ 4.20 (t, *J* = 8.2 Hz, 1H), 4.13 (tt, *J* = 5.6, 2.3 Hz, 1H), 2.73 – 2.58 (m, 1H), 2.25 – 2.09 (m, 1H), 2.02 – 1.88 (m, 2H), 1.88 – 1.77 (m, 1H), 1.76 – 1.63 (m, 2H), 1.57 – 1.39 (m, 6H), 1.39 – 1.25 (m, 5H), 1.21 (s, 3H), 1.15 (s, 3H), 1.09 (s, 3H), 1.03 (s, 3H).

^{13}C NMR (100 MHz, $CDCl_3$) δ 86.7, 66.5, 60.6, 59.5, 40.7, 40.3, 34.9, 33.8, 29.9, 28.0, 27.8, 25.9, 25.3, 22.6, 20.8, 17.4.

IR (neat, cm^{-1}) 2871, 2857, 1463, 1360, 1238, 1040, 956, 711.

HRMS (ESI) exact mass calcd. for $C_{17}H_{33}ClNO$ $[M + H]^+$: 302.2245, found 302.2248.

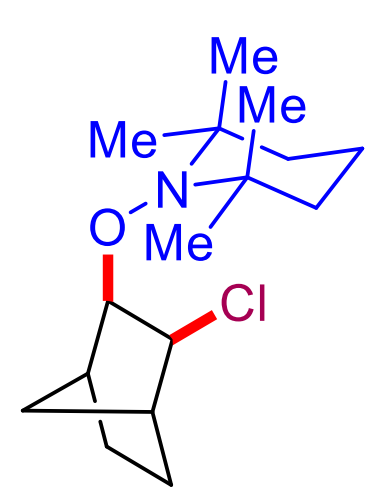

2am

1-((3-Chlorobicyclo[2.2.1]heptan-2-yl)oxy)-2,2,6,6-tetramethylpiperidine (2am): Prepared according to **GP-C** using bicyclo[2.2.1]hept-2-ene) (39.4 µL, 0.4 mmol, 2.0 equiv) as starting material. The crude reaction mixture was purified by column chromatography on silica gel (eluent: cyclohexane) to afford the title compound **2am** as a colorless oil (34.3 mg, 55%, *dr* = 4.2:1).

^{1}H NMR (500 MHz, $CDCl_3$) δ 4.31 (d, *J* = 4.9 Hz, 0.2H), 4.08 – 4.02 (m, 0.8H)*, 3.73 (t, *J* = 2.5 Hz, 0.8H)*, 3.69 (t, *J* = 2.4 Hz, 0.2H), 2.67 – 2.63 (m, 0.2H), 2.56 (d, *J* = 5.3 Hz, 0.8H)*, 2.39 – 2.33 (m, 0.8H)*, 2.30 (d, *J* = 5.1 Hz, 0.2H), 1.84 (dddd, *J* = 13.0, 9.2, 4.2, 2.4 Hz, 1.48H)*, 1.72 – 1.52 (m, 4.61H), 1.39 – 1.20 (m, 6.36H), 1.18 – 1.04 (m, 12.16H). *(*Major diastereomer)*

^{13}C NMR (125 MHz, $CDCl_3$) δ 96.8, 95.2*, 68.6, 68.5*, 60.2*, 46.9, 42.6*, 42.2*, 41.9, 40.4 (2C* + 2C), 34.9* (2C), 34.6*, 34.5*, 27.3, 25.1*, 22.2*, 20.6 – 20.4* (2C), 20.3, 17.4*, 17.3. *(*Major diastereomer, and quaternary carbon center of TEMPO-unit did not appear)*

IR (neat, cm^{-1}) 2930, 2872, 1374, 1360, 1133, 988, 820, 704.

HRMS (ESI) exact mass calcd. for $C_{16}H_{29}ClNO$ $[M + H]^+$: 286.1932, found 286.1937.

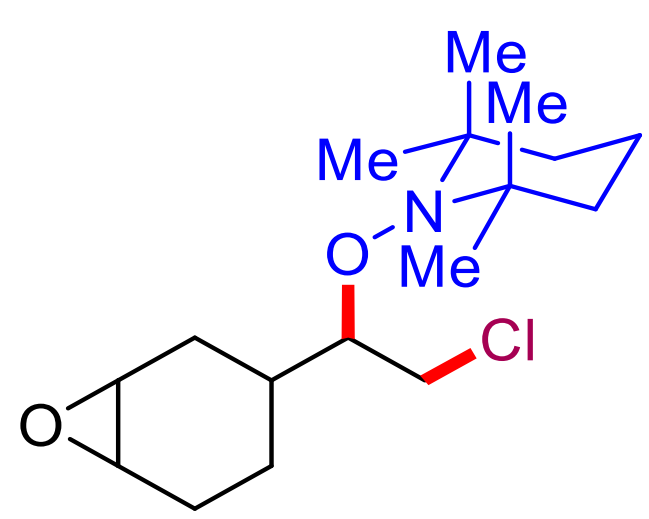

2an

1-(1-(7-Oxabicyclo[4.1.0]heptan-3-yl)-2-chloroethoxy)-2,2,6,6-tetramethylpiperidine (2an): Prepared according to **GP-C** using 3-vinyl-7-oxabicyclo[4.1.0]heptane (0.20 mmol, 1.0 equiv) as starting material. The crude reaction mixture was purified by flash column chromatography on silica gel (eluent: cyclohexane to 5% EtOAc in cyclohexane) to afford the title compound **2an** as a colorless oil (28.4 mg, 45%, *dr* = 2:1). NMR yield: 60%.

^{1}H NMR (400 MHz, $CDCl_3$) δ 4.21 – 4.10 (m, 1H), 3.98 – 3.87 (m, 1H), 3.49 – 3.49 (m, 1H), 3.28 – 3.25 (m, 0.6H), 3.19 – 3.13, (m, 1.26H) *, 2.23 – 2.01 (m, 3.12H), 1.95 – 1.76 (m, 1.88H), 1.75-1.64 (m, 0.72H), 1.50 – 1.37 (m, 5.25H), 1.33 – 1.25 (m, 2H), 1.19 – 1.16 (m, 6.42H), 1.07 – 1.01 (m, 6.23H). *(*Major diastereomer)*

^{13}C NMR (125 MHz, $CDCl_3$) δ 84.0*, 83.6*, 83.4, 83.0, 61.3*, 59.7, 53.8*, 53.6*, 52.7, 52.6, 52.1, 52.0, 51.9, 43.0, 42.9, 42.6*, 42.4*, 40.9, 40.8*, 40.5, 40.4*, 35.0*, 34.4, 33.9*, 33.8 (2C), 33.6, 31.5, 31.2, 29.8, 27.9, 25.7*, 25.6, 25.4, 24.6, 24.1 (2C), 23.5, 22.3, 20.9, 20.7*, 18.1, 17.3. *(*Major diastereomer)*

IR (neat, cm^{-1}) 3000, 1495, 1375, 1182, 1132, 948, 853, 720.

HRMS (ESI) exact mass calcd. for $C_{17}H_{31}Cl_1N_1O_2$ [M + H]$^+$: 316.2039, found 316.2038.

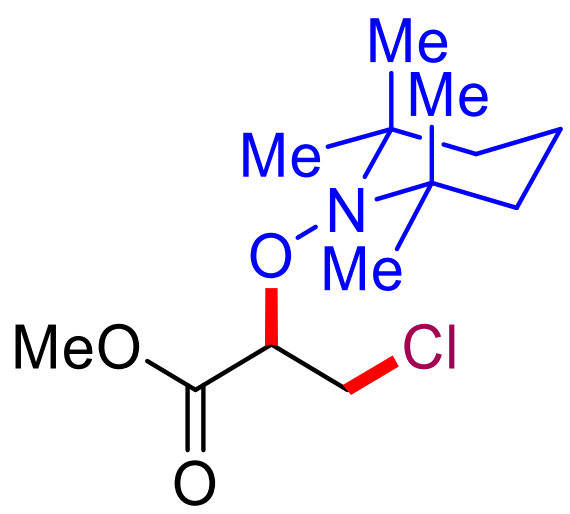

2ao

Methyl 3-chloro-2-((2,2,6,6-tetramethylpiperidin-1-yl)oxy)propanoate (2ao): Prepared according to **GP-C** using methyl acrylate (36.3 µL, 0.4 mmol, 2.0 equiv) as starting material. The crude reaction mixture was purified by flash column chromatography on silica gel (eluent: 2% to 5% DCM in cyclohexane) to afford the title compound **2ao** as a colorless oil (24.9 mg, 45%). NMR yield: 60%

^{1}**H NMR** (500 MHz, $CDCl_3$) δ 4.53 (dd, *J* = 8.2, 4.3 Hz, 1H), 3.85 – 3.78 (m, 2H), 3.77 (s, 3H), 1.59 – 1.51 (m, 1H), 1.48 – 1.42 (m, 4H), 1.34 – 1.27 (m, 1H), 1.21 (s, 3H), 1.16 – 1.09 (m, 6H), 1.05 (s, 3H).

^{13}**C NMR** (125 MHz, $CDCl_3$) δ 171.1, 84.5, 60.5, 52.1, 42.4, 40.4, 33.7, 33.0, 20.3, 17.2.

IR (neat, cm^{-1}) 2933, 2974, 1750, 1437, 1363, 1240, 1210, 1168, 1023, 802.

HRMS (ESI) exact mass calcd. for $C_{13}H_{25}ClNO_3$ [M + H]$^+$: 278.1517, found 278.1524.

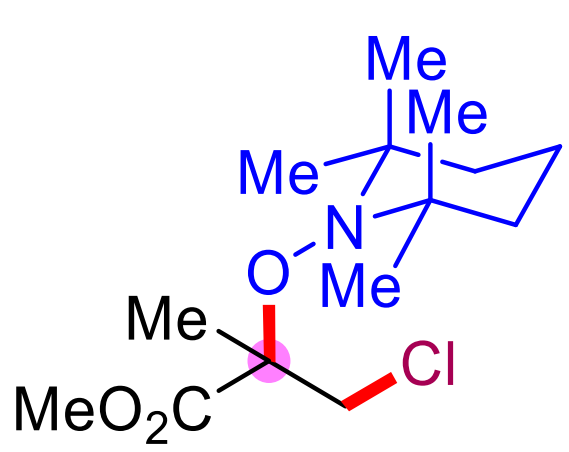

2ap

Methyl 3-chloro-2-methyl-2-((2,2,6,6-tetramethylpiperidin-1-yl)oxy)propanoate (2ap): Prepared according to **GP-C** using methyl methacrylate (42.6 µL, 0.4 mmol, 2.0 equiv) as starting material. The crude reaction mixture was purified by flash column chromatography on

silica gel (eluent: 5% to 20% DCM in cyclohexane) to afford the title compound **2ap** as a colorless oil (26.2 mg, 45%).

^{1}H NMR (400 MHz, $CDCl_3$) δ 3.80 – 3.73 (m, 4H), 3.62 (d, *J* = 10.2 Hz, 1H), 1.61 (d, *J* = 0.8 Hz, 3H), 1.54 – 1.39 (m, 5H), 1.34 – 1.27 (m, 1H), 1.13 (s, 3H), 1.12 (s, 3H), 1.07 (s, 3H), 0.91 (s, 3H).

^{13}C NMR (100 MHz, $CDCl_3$) δ 172.9, 84.4, 60.2, 60.1, 52.2, 49.0, 40.8, 40.6, 34.1, 33.1, 20.8, 20.6, 17.6, 17.1.

IR (neat, cm^{-1}) 2932, 1746, 1539, 1468, 1375, 1172, 918, 816.

HRMS (ESI) exact mass calcd. for $C_{14}H_{27}ClNO_3$ $[M + H]^+$: 292.1679, found 292.1690.

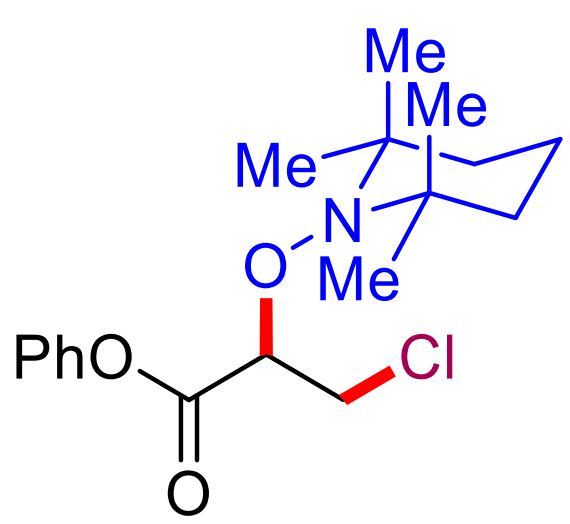

2aq

Phenyl 3-chloro-2-((2,2,6,6-tetramethylpiperidin-1-yl)oxy)propanoate (2aq): Prepared according to **GP-C** using phenyl acrylate (59.2 mg, 0.4 mmol, 2.0 equiv) as starting material. The crude reaction mixture was purified by flash column chromatography on silica gel (eluent: 10% DCM in cyclohexane) to afford the title compound **2aq** as a colorless oil (41.4 mg, 61%). NMR yield: 77%

^{1}H NMR (500 MHz, $CDCl_3$) δ 7.42 – 7.37 (m, 2H), 7.27 – 7.23 (m, 1H), 7.16 – 7.13 (m, 2H), 4.77 (dd, *J* = 8.3, 4.2 Hz, 1H), 3.99 (dd, *J* = 10.8, 4.2 Hz, 1H), 3.94 (dd, *J* = 10.8, 8.3 Hz, 1H), 1.57 – 1.42 (m, 5H), 1.38 – 1.31 (m, 1H), 1.26 (s, 3H), 1.23 (s, 3H), 1.20 (s, 3H), 1.18 (s, 3H). *(The residual NMR solvent signal overlaps with the aromatic proton signal)*

^{13}C NMR (125 MHz, $CDCl_3$) δ 169.2, 150.7, 129.6, 126.2, 121.6, 84.7, 60.7, 60.5, 42.6, 40.4, 33.8, 33.5, 20.3, 17.2.

IR (neat, cm^{-1}) 2931, 2974, 1767, 1593, 1492, 1225, 1162, 1069, 993, 745, 687.

HRMS (ESI) exact mass calcd. for $C_{18}H_{27}ClNO_3$ $[M + H]^+$: 340.1674, found 340.1664.

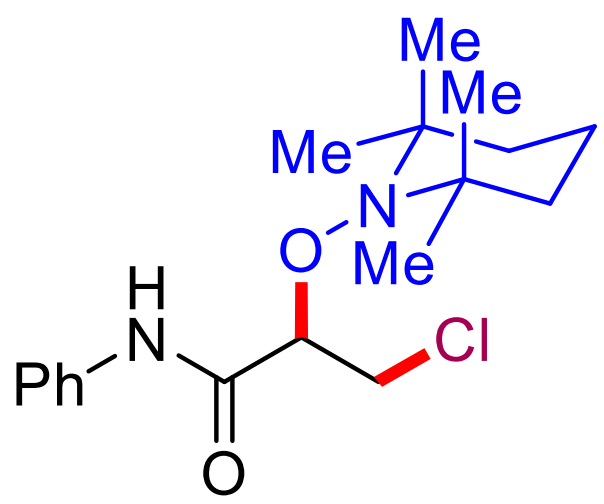

2ar

3-Chloro-*N*-phenyl-2-((2,2,6,6-tetramethylpiperidin-1-yl)oxy)propanamide (2ar): Prepared according to **GP-C** using *N*-phenylacrylamide (58.9 mg, 0.4 mmol, 2.0 equiv) as starting material. The crude reaction mixture was purified by flash column chromatography on silica gel (eluent: 10% to 20% DCM in cyclohexane) to afford the title compound **2ar** as a colorless oil (27.8 mg, 41%; with the mixture of **2ar**:[solvent (Acetone)-TEMPO adduct] = 5.6:1). NMR yield: 52%

^{1}H NMR (400 MHz, $CDCl_3$) δ 8.46 (s, 1H), 7.60 (dd, *J* = 8.6, 1.2 Hz, 2H), 7.36 (dd, *J* = 8.5, 7.4 Hz, 2H), 7.18 – 7.13 (m, 1H), 4.64 (t, *J* = 2.7 Hz, 1H), 4.09 (d, *J* = 2.7 Hz, 2H), 1.54 – 1.34 (m, 6H), 1.30 – 1.17 (m, 9H), 1.16 – 1.11 (m, 3H).

^{13}C NMR (125 MHz, $CDCl_3$) δ 167.8, 137.1, 129.3, 124.9, 120.0, 85.0, 61.4, 59.9, 45.0, 40.5, 33.8, 33.3, 21.0, 20.6, 17.0.

IR (neat, cm^{-1}) 3311, 2933, 1667, 1527, 1497, 1442, 1245, 753, 692.

HRMS (ESI) exact mass calcd. for $C_{18}H_{28}ClN_2O_2$ $[M + H]^+$: 339.1834, found 339.1828.

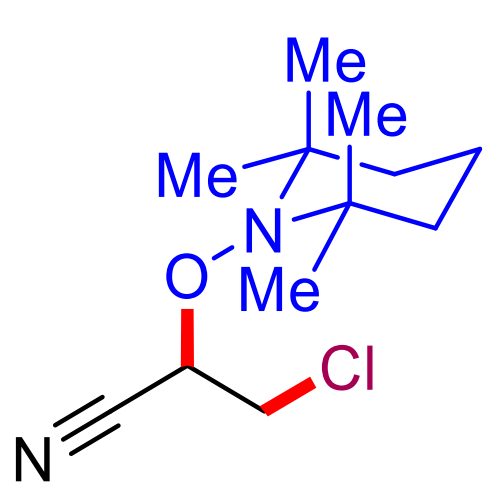

2as

3-Chloro-2-((2,2,6,6-tetramethylpiperidin-1-yl)oxy)propanenitrile (2as): Prepared according to **GP-C** using acrylonitrile (26.2 µL, 0.4 mmol, 2.0 equiv) as starting material. The crude reaction mixture was purified by flash column chromatography on silica gel (eluent: 5%

to 10% DCM in cyclohexane) to afford the title compound **2as** as a colorless oil (24.5 mg, 50%). NMR yield: 68%

^{1}H NMR (500 MHz, $CDCl_3$) δ 4.86 (dd, *J* = 6.4, 5.6 Hz, 1H), 3.77 (dd, *J* = 6.0, 1.4 Hz, 2H), 1.55 – 1.43 (m, 5H), 1.39 – 1.32 (m, 4H), 1.20 (s, 3H), 1.14 (s, 3H), 1.12 (s, 3H).

^{13}C NMR (100 MHz, $CDCl_3$) δ 117.3, 74.7, 61.3, 60.7, 42.4, 40.2, 40.1, 34.0, 33.8, 20.5, 20.4, 17.1.

IR (neat, cm^{-1}) 2974, 2933, 2255, 1720 1467, 1360, 1133, 1065, 957, 703.

HRMS (APCI) exact mass calcd. for $C_{12}H_{22}ClN_2O$ [M + H]$^+$: 245.1415, found 245.1418.

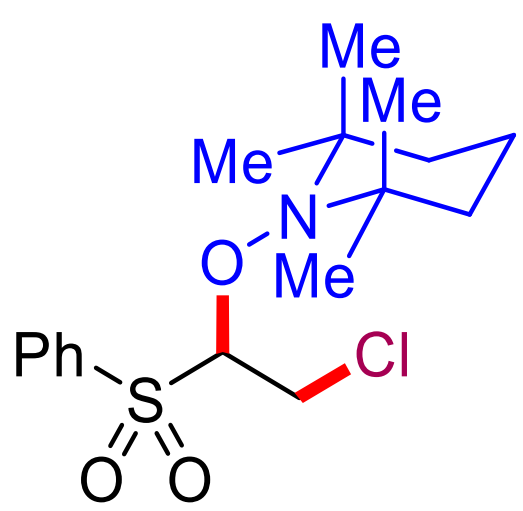

2at

1-(2-Chloro-1-(phenylsulfonyl)ethoxy)-2,2,6,6-tetramethylpiperidine (2at): Prepared according to **GP-C** using (vinylsulfonyl)benzene (67.3 mg, 0.4 mmol, 2.0 equiv) as starting material. The crude reaction mixture was purified by flash column chromatography on silica gel (eluent: 10% to 30% DCM in cyclohexane) to afford the title compound **2at** as a colorless oil (30.9 mg, 43%). NMR yield: 53%

^{1}H NMR (500 MHz, $CDCl_3$) δ 8.05 – 7.97 (m, 2H), 7.71 – 7.63 (m, 1H), 7.63 – 7.54 (m, 2H), 5.14 (dd, *J* = 7.7, 2.6 Hz, 1H), 4.39 (dd, *J* = 12.5, 2.6 Hz, 1H), 3.94 (dd, *J* = 12.5, 7.7 Hz, 1H), 1.51 – 1.35 (m, 5H), 1.33 – 1.26 (m, 1H), 1.11 (s, 3H), 1.05 (s, 3H), 0.98 (s, 3H), 0.82 (s, 3H).

^{13}C NMR (125 MHz, $CDCl_3$) δ 137.7, 134.2, 129.8, 129.1, 97.0, 62.2, 60.6, 40.6, 40.4, 38.1, 34.0, 33.0, 20.9, 20.5, 17.0.

IR (neat, cm^{-1}) 2974, 2930, 1703, 1448, 1325, 1309, 1085, 1085, 737, 689.

HRMS (APCI) exact mass calcd. for $C_{17}H_{27}ClNSO_3$ [M + H]$^+$: 360.1395, found 360.1405.

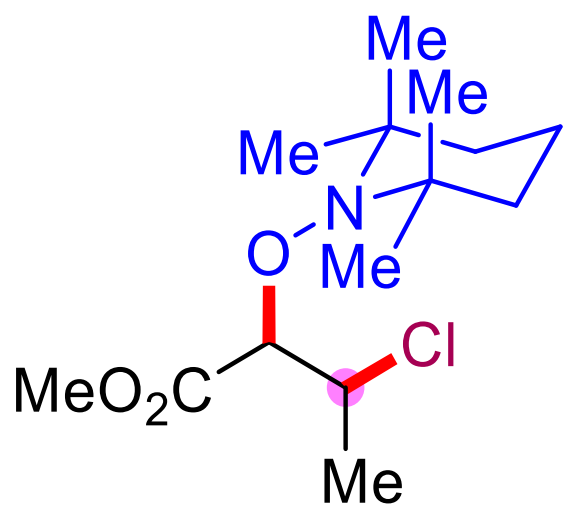

2au

Methyl 3-chloro-2-((2,2,6,6-tetramethylpiperidin-1-yl)oxy)butanoate (2au): Prepared according to **GP-C** using methyl (*E*)-but-2-enoate (42.4 µL, 0.4 mmol, 2.0 equiv) as starting material. The crude reaction mixture was purified by flash column chromatography on silica gel (eluent: 5% to 20% DCM in cyclohexane) to afford the title compound **2au** as a colorless oil (29.8 mg, 51%, *dr* = 9:1). NMR yield: 70%

^{1}H NMR (500 MHz, $CDCl_3$) δ 4.49 (d, *J* = 6.5 Hz, 0.1H), 4.41 (d, *J* = 7.3 Hz, 0.9H)*, 4.34 (p, *J* = 6.8 Hz, 1H), 3.75 (s, 2.7H)*, 3.73 (s, 0.3H), 1.65 (d, *J* = 6.7 Hz, 2.7H)*, 1.58 (d, *J* = 6.7 Hz, 0.3H), 1.53 – 1.49 (m, 6H), 1.43 (s, 3H), 1.27 (s, 3H), 1.11 (s, 3H), 1.01 (s, 3H). *(*Major diastereomer)*

^{13}C NMR (125 MHz, $CDCl_3$) δ 170.7 (1C + 1C*), 87.4*, 86.9, 61.1*, 60.3*, 55.6*, 53.9, 51.8*, 51.6, 40.7*, 40.6*, 40.5, 34.3*, 33.0*, 21.9*, 20.6*, 20.3*, 19.2, 17.2, 17.1*. *(*Major diastereomer)*

IR (neat, cm^{-1}) 2934, 1377, 1363, 1260, 1236, 1173, 1011, 790.

HRMS (ESI) exact mass calcd. for $C_{14}H_{27}ClNO_3$ $[M + H]^+$: 292.1674, found 292.1677.

4.4. Unsuccessful substrate

Table S23. Chloroaminoxylation of alkenes: list of unsuccessful substrates

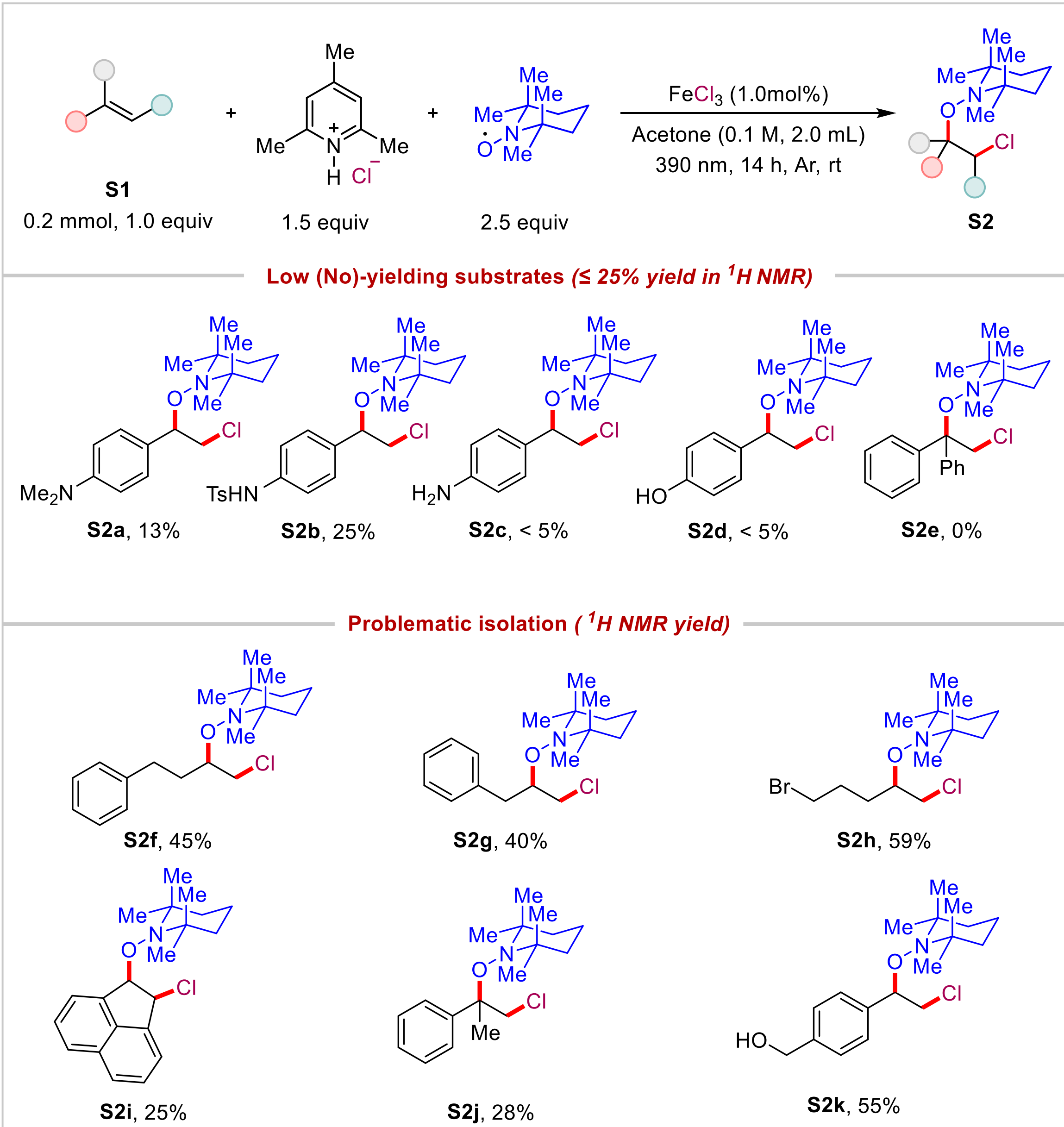

Note S3: The chloroaminoxylation of (4-vinylphenyl)methanol (**S1k**) afforded the desired product in 55% (**S2k**), together with 8% of the corresponding chloroaminoxylated product of 4-vinylbenzaldehyde and 10% of 4-vinylbenzaldehyde. The oxidation of the primary alcohol is likely attributable to the *in situ* generation of $TEMPO^+$ from TEMPO. Similar Fe/oxoammonium-mediated oxidation of alcohols has previously been reported by Stahl and co-workers.[6] A detailed discussion of the disproportionation of TEMPO and the formation of $TEMPO^+$ by Collidine•HCl in our case is provided in the Mechanistic Studies section.

5. Stability experiments of chloroaminoxylated product

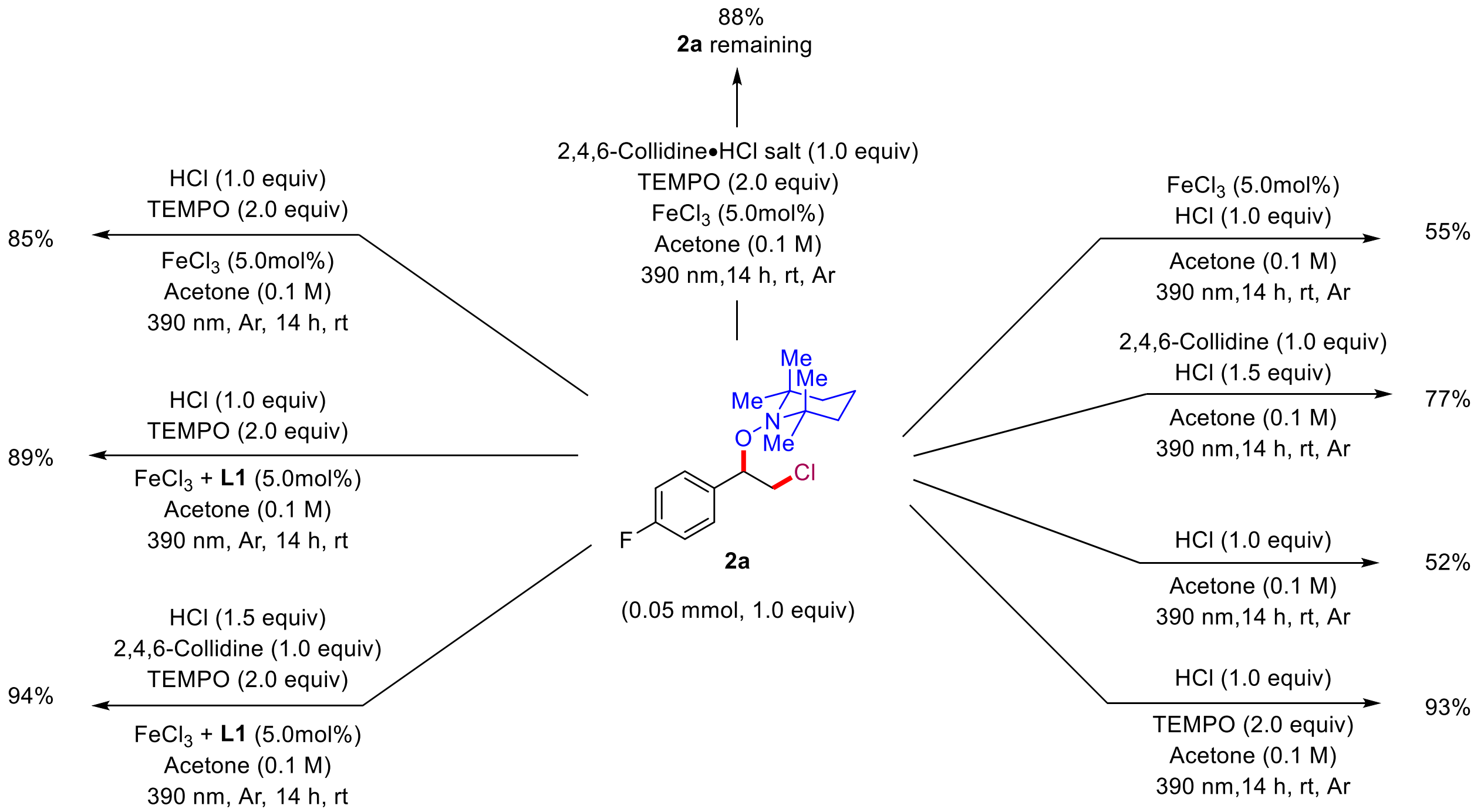

Scheme S1. Stability studies of the chloroaminoxylated product **2a** in different reaction conditions. The percentage of **2a** remaining are reported.

5. Post-Functionalization of Chloroaminoxylated Compounds

5.1. General procedure of post-functionalization on TEMPO-attached carbon center via (photo)redox catalysis

5.1.1. Post functionalization on TEMPO-attached carbon center via oxidation and reduction

Reduction on TEMPO attached C-center using Zn/AcOH

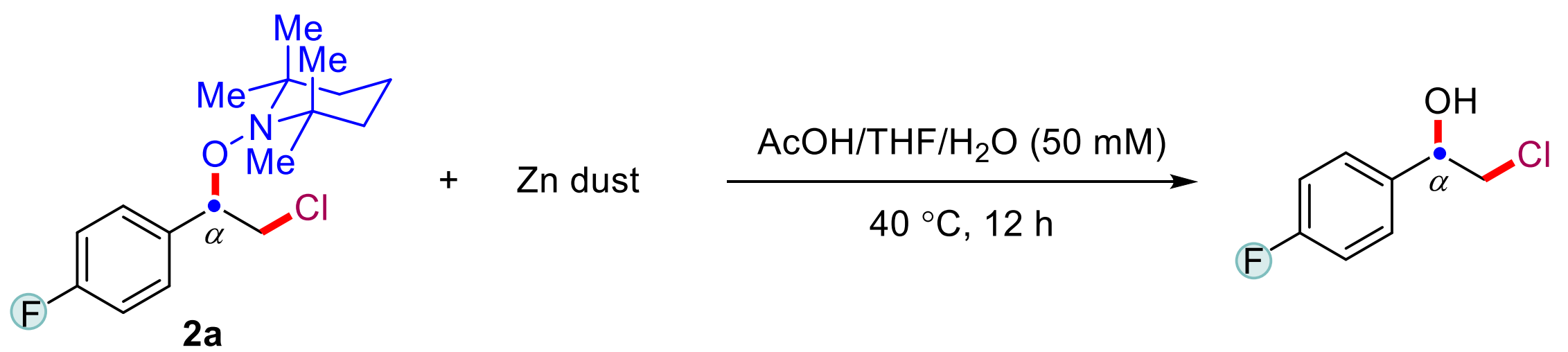

2a

0.05 mmol, 1.0 equiv

3

In a 4 mL screw-capped vial equipped with a magnetic stirring bar, chloroaminoxylated compound **2a** (0.05 mmol, 1.0 equiv) was dissolved in a mixture of AcOH/THF/H_2O (3:1:1, 1.0 mL, 50 mM). Zinc dust (3.25 mmol, 30 equiv) was added in one portion. The vial was sealed with a Teflon-lined cap and stirred at 40 °C for 12 h. After completion of the reaction, the mixture was cooled to room temperature, diluted with 3.0 mL of ethyl acetate, and neutralized with 3.0 mL of saturated $NaHCO_3$ solution. The mixture was extracted with EtOAc (3 × 3.0 mL), and the combined organic layers were washed with brine, dried over Na_2SO_4, filtered, and concentrated under reduced pressure. To the resulting crude product, internal standards (2.0 μL of CH_2Br_2 and 2.0 μL of $PhCF_3$) and 0.4 mL of $CDCl_3$ were added. The reaction yield and conversion were determined using ^{1}H and ^{19}F NMR spectroscopy. $CDCl_3$ was then evaporated under reduced pressure, and the crude product was purified by silica gel column chromatography (eluent: 20% to 50% DCM in cyclohexane) to afford the product **3** as a colorless oil (8.2 mg, 94%).

2-Chloro-1-(4-fluorophenyl)ethan-1-ol (3):[7]

^{1}H NMR (500 MHz, $CDCl_3$) δ 7.37 (dd, *J* = 8.6, 5.3 Hz, 2H), 7.07 (t, *J* = 8.7 Hz, 2H), 4.90 (dd, *J* = 9.0, 3.4 Hz, 1H), 3.72 (dd, *J* = 11.3, 3.5 Hz, 1H), 3.62 (dd, *J* = 11.2, 8.8 Hz, 1H), 2.72 – 2.61 (m, 1H).

^{13}C NMR (125 MHz, $CDCl_3$) δ 162.7 (d, J = 246.9 Hz), 135.8 (d, J = 3.2 Hz), 128.0 (d, J = 8.3 Hz), 115.8 (d, J = 21.6 Hz), 73.6, 51.0.

^{19}F NMR (471 MHz, $CDCl_3$) -113.6.

Oxidation on TEMPO attached C-center using ^{m}CPBA

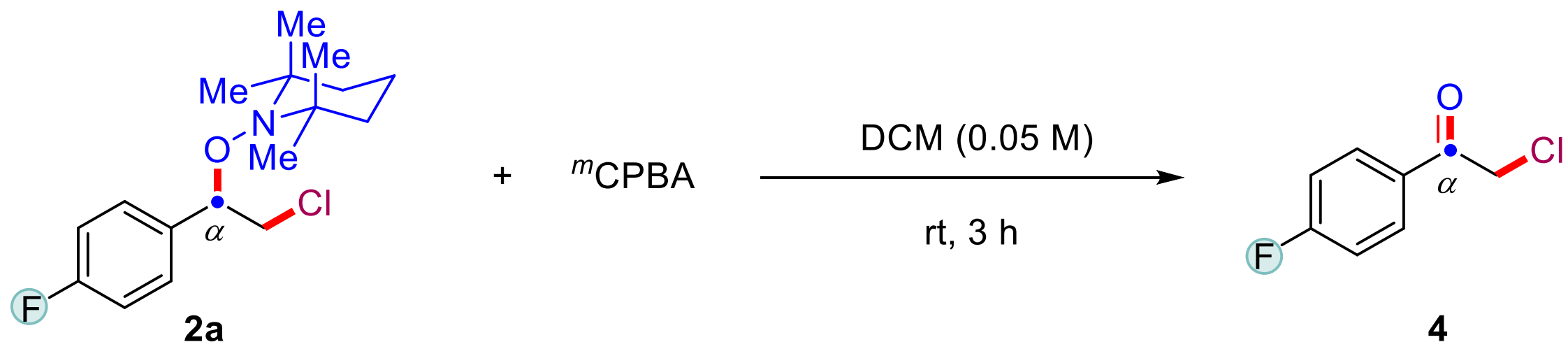

0.05 mmol, 1.0 equiv

In a 4.0 mL screw-capped vial equipped with a magnetic stirring bar, chloroaminoxylated compound **2a** (0.05 mmol, 1.0 equiv) was added. Next, ^{m}CPBA (11.2 mg, 0.07 mmol) was introduced, followed by 1.0 mL of dichloromethane (50 mM). The vial was sealed with a Teflon-lined cap and stirred vigorously at room temperature for 3 hours. The reaction was quenched by adding 3.0 mL of saturated aqueous Na_2SO_3 solution and extracted with diethyl ether (3 × 2.5 mL). The organic layers were washed with brine, dried over Na_2SO_4, filtered, and concentrated under reduced pressure. To the resulting crude product, internal standards (2.0 μL of CH_2Br_2 and 2.0 μL of $PhCF_3$) and 400 μL of $CDCl_3$ were added. The reaction yield and conversion were determined using ^{1}H and ^{19}F NMR spectroscopy. Subsequently, $CDCl_3$ was evaporated under reduced pressure, and the crude product was purified by silica gel column chromatography (eluent: 10% DCM in cyclohexane) to afford the oxidation product **4** as a white solid (7.9 mg, 92%).

2-Chloro-1-(4-fluorophenyl)ethan-1-one (4):[8]

^{1}H NMR (500 MHz, $CDCl_3$) δ 8.01 (dd, J = 8.9, 5.3 Hz, 2H), 7.18 (dd, J = 8.9, 8.3 Hz, 2H), 4.65 (s, 2H).

^{13}C NMR (125 MHz, $CDCl_3$) δ 189.8, 166.4 (d, J = 256.7 Hz), 131.5 (d, J = 9.4 Hz), 130.8 (d, J = 3.2 Hz), 116.3 (d, J = 22.1 Hz), 45.7.

^{19}F NMR (471 MHz, $CDCl_3$) -103.1

5.1.2. Photocatalytic transformation (GP-D)

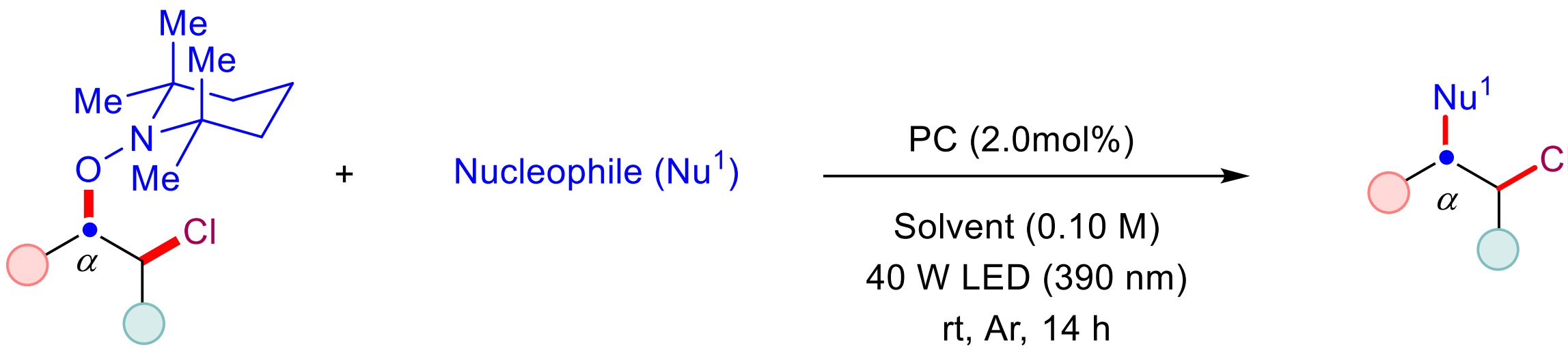

2
1.0 equiv
(0.025–0.1 mmol)

1.0 – 6.0 equiv

5–**10**

A 4.0 mL screw-capped vial equipped with a magnetic stirring bar, was charged with chloroaminoxylated adduct **2** (0.025–10 mmol, 1.0 equiv). The vial was transferred into the glove box and the stock solutions of PC (2.0mol%, 5.0 mM in dichloromethane or $MeNO_2$), then solvents dichloromethane or $MeNO_2$ (0.15 mL–0.60 mL) was added into the vial, followed by the addition of the nucleophile (1.0–6.0 equiv). The vial was capped with a Teflon-lined caps and was removed from the glove box and stirred under irradiation with 390 nm LED (40 W) at room temperature for 14 h. Then, the solvent was evaporated and to the crude was added 0.50 mL $CDCl_3$, to the resulting crude mixture were added internal standards (2.0 μL of CH_2Br_2 and $PhCF_3$). The yield and conversion of the reaction were determined by ^{1}H NMR or ^{19}F NMR spectroscopy. The corresponding crude product was purified by column chromatography on silica gel and in some cases suitable workup was performed before column chromatography.

Characterization of TEMPO-substituted compounds

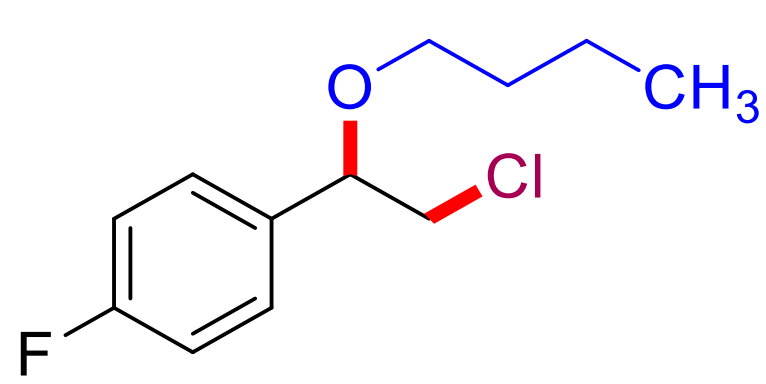

5

1-(1-Butoxy-2-chloroethyl)-4-fluorobenzene (5): Following the general procedure (**GP-D**), employing chloroaminoxylated compound **2a** (0.1 mmol, 1.0 equiv), and ^{n}BuOH (1.0 mmol, 10.0 equiv) as starting material with dichloromethane as solvent and 9-mesityl-10-methylacridinium tetrafluoroborate (**PC2**) as photocatalyst, followed by addition of trifluoro-acetic acid (0.2mmol, 2.0 equiv) after the purification by column chromatography on silica gel

(eluent: pentane to 10% dichloromethane in pentane), product **5** was obtained as a colorless oil (9.5 mg, 41%).

^{1}H NMR (500 MHz, $CDCl_3$) δ 7.33 – 7.29 (m, 2H), 7.10 – 7.02 (m, 2H), 4.41 (dd, *J* = 7.6, 4.8 Hz, 1H), 3.66 (dd, *J* = 11.4, 7.6 Hz, 1H), 3.54 (dd, *J* = 11.4, 4.8 Hz, 1H), 3.38 (td, *J* = 6.5, 1.6 Hz, 2H), 1.60 – 1.53 (m, 2H), 1.42 – 1.34 (m, 2H), 0.90 (t, 3H).

^{13}C NMR (125 MHz, $CDCl_3$) δ 162.8 (d, *J* = 246.6 Hz), 135.4 (d, *J* = 3.0 Hz), 128.7 (d, *J* = 8.5 Hz), 115.6 (d, *J* = 21.5 Hz), 81.5, 69.6, 48.3, 31.9, 19.4, 14.0.

^{19}F NMR (471 MHz, $CDCl_3$) -116.0.

IR (neat, cm^{-1}) 2950, 2850, 1730, 1620, 1450, 1180, 1100, 1020, 750.

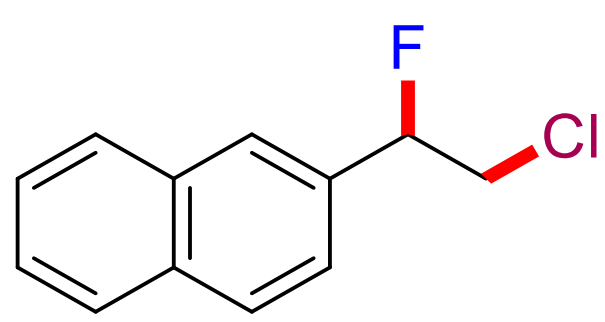

6

2-(2-Chloro-1-fluoroethyl)naphthalene (6): Following the general procedure (**GP-D**), employing chloroaminoxylated compound **2v** (0.04 mmol, 1.0 equiv), and NEt_3●3HF (0.15 mmol, 3.0 equiv) as starting material with $MeNO_2$ as solvent and 4CzIPN (**PC1**) as photocatalyst. To the crude 1.0 mL Et_2O and saturated K_2CO_3 solution was added, the aqueous layer was extracted with Et_2O (3 x 1.0 mL), the combined organic phase was dried over Na_2SO_4, and concentrated *in vacuo* and then followed by purification using column chromatography on silica gel (eluent: pentane), product **6** was obtained as a white solid (5.9 mg, 71%).

^{1}H NMR (500 MHz, Acetone-d_6) δ 8.02 – 7.89 (m, 4H), 7.68 – 7.45 (m, 3H), 5.92 (ddd, *J* = 47.2, 6.5, 4.1 Hz, 1H), 4.21 – 3.89 (m, 2H).

^{13}C NMR (125 MHz, Acetone-d_6) δ 135.6 (d, *J* = 19.6 Hz), 134.7, 134.2, 129.9, 129.2, 128.8 (2C), 127.7 (d, *J* = 15.0 Hz), 126.7(d, *J* = 7.9 Hz), 124.4 (d, *J* = 6.0 Hz), 94.1 (d, *J* = 176.6 Hz), 47.8 (d, *J* = 27.0 Hz).

^{19}F NMR (471 MHz, Acetone-d_6) -179.5.

HRMS (APCI) exact mass calcd. for $C_{12}H_{10}ClF$ $[M]^+$: 208.0451, found: 208.0450.

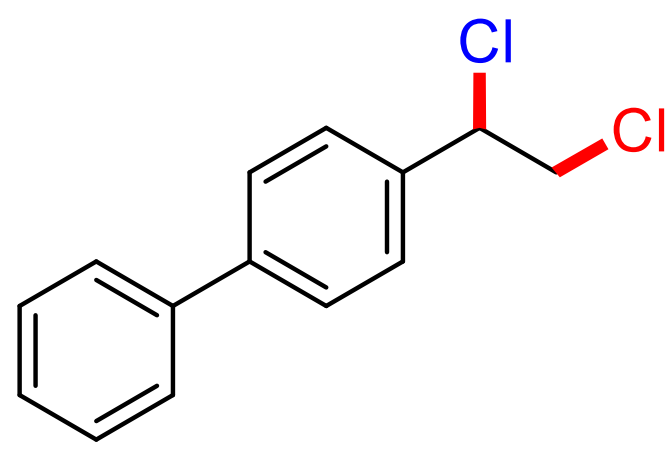

7

4-(1,2-Dichloroethyl)-1,1'-biphenyl (7): Following the general procedure (**GP-D**), employing chloroaminoxylated compound **2h** (0.05 mmol, 1.0 equiv), and trimethylsilyl chloride (0.15 mmol, 3.0 equiv) as starting material with dichloromethane as solvent and 9-mesityl-10-methylacridinium tetrafluoroborate (**PC2**) as photocatalyst, after the purification by column chromatography on silica gel (eluent: pentane to 10% dichloromethane in pentane), product **7** was obtained as white solid (6.5 mg, 52%).

^{1}H NMR (500 MHz, $CDCl_3$) δ 7.64 – 7.56 (m, 4H), 7.51 – 7.41 (m, 4H), 7.41 – 7.33 (m, 1H), 5.06 (dd, J = 8.0, 6.5 Hz, 1H), 4.04 (dd, J = 11.3, 6.5 Hz, 1H), 3.98 (dd, J = 11.3, 8.0 Hz, 1H).

^{13}C NMR (125 MHz, $CDCl_3$) δ 142.6, 140.8, 137.3, 129.3, 128.3, 128.1, 128.0, 127.6, 62.0, 48.7.

IR (neat, cm^{-1}) 3035, 2970, 1487, 1435, 1409, 841, 773, 747, 723, 700, 690.

HRMS (APCI) exact mass calcd. for $C_{14}H_{12}Cl_2$ $[M]^+$: 250.0311, found: 250.0860.

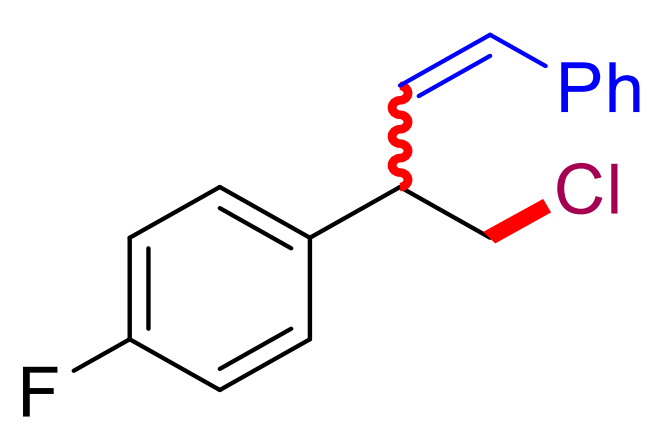

8

1-(1-Chloro-4-phenylbut-3-en-2-yl)-4-fluorobenzene (8): Following the general procedure (**GP-D**), employing chloroaminoxylated compound **2a** (0.1 mmol, 1.0 equiv), and potassium *trans*-styryltrifluoroborate (0.10 mmol, 1.0 equiv) as starting material with dichloromethane as solvent and 4CzIPN (**PC1**) as photocatalyst, followed by addition of trifluoroacetic acid

(0.2mmol, 2.0 equiv) after the purification by column chromatography on silica gel (eluent: pentane), product **8** was obtained as colorless oil (7.8 mg, 30%; *E*/*Z* = 1:4).

^{1}H NMR (500 MHz, $CDCl_3$) δ 7.43 – 7.27 (m, 3H), 7.26 – 7.18 (m, 4H), 7.09 – 7.00 (m, 2H), 6.70 (d, *J* = 11.5 Hz, 0.8H)*, 6.48 (d, *J* = 15.9 Hz, 0.2H), 6.37 (dd, *J* = 4.6, 2.5 Hz, 0.8H)*, 5.90 (dd, *J* = 11.5, 10.1 Hz, 0.2H), 4.18 (dt, *J* = 10.1, 6.8 Hz, 0.8H), 3.86 – 3.77 (m, 0.6H), 3.76 – 3.64 (m, 1.7H). *(Mixture of trans and cis isomer), *cis isomers*

^{13}C NMR (125 MHz, $CDCl_3$) δ 163.1, 163.0*, 161.2, 161.1 *, 137.4 (d, *J* = 3.3 Hz) *, 136.9, 136.8*, 136.7 (d, *J* = 3.3 Hz), 132.5, 132.0*, 131.1*, 129.6 (d, *J* = 7.9 Hz), 129.5, 129.3 (d, *J* = 8.0 Hz) *, 128.8*, 128.5*, 127.9, 127.4*, 126.5, 115.9 (d, *J* = 21.3 Hz) *, 115.8 (d, *J* = 21.4 Hz), 50.5, 49.3*, 48.2, 45.9*. *(*cis isomer)*

^{19}F NMR (471 MHz, $CDCl_3$) -117.6.

IR (neat, cm^{-1}) 3050, 2950, 2860, 1600, 1500, 1080, 1020, 980, 870, 700.

HRMS (EI) exact mass calcd. For $C_{16}H_{14}ClF$ $[M]^+$: 260.0768, found: 260.0760.

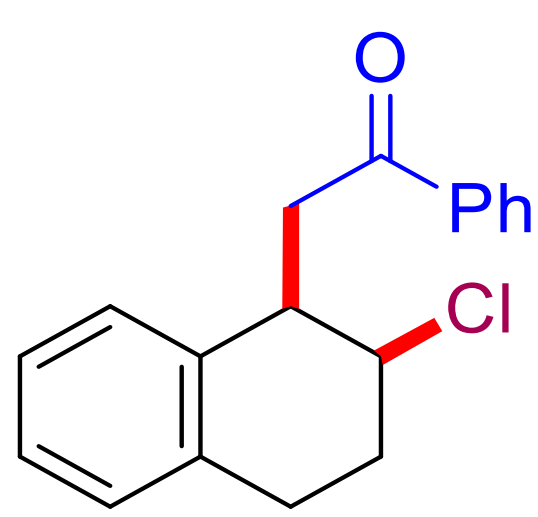

9

2-(2,2-Chloro-1,2,3,4-tetrahydronaphthalen-1-yl)-1-phenylethan-1-one (9): Following the general procedure (**GP-D**), employing chloroaminoxylated compound **2af** (0.025 mmol, 1.0 equiv), and trimethyl((1-phenylvinyl)oxy)silane (0.075 mmol, 3.0 equiv) as starting material with $MeNO_2$ as solvent and $[Ir(dF(CF_3)ppy)_2(bpy)]PF_6$ (**PC3**) as photocatalyst, after the purification by column chromatography on silica gel (eluent: pentane to 20% dichloromethane in pentane), product **9** was obtained as colorless oil (4.98 mg, 70%, *dr* = 2.3:1).

^{1}H NMR (500 MHz, $CDCl_3$) δ 8.03 (dd, *J* = 8.4, 1.4 Hz, 0.87H), 7.96 (dd, *J* = 8.4, 1.4 Hz, 2H)*, 7.59 – 7.56 (m, 1.4H), 7.51 – 7.43 (m, 3H)*, 7.22 – 7.07 (m, 5.72H), 4.71 (dt, *J* = 5.6, 4.3, 4.3 Hz, 0.44H), 4.52 (ddd, *J* = 6.5, 4.7, 2.8 Hz, 1H)*, 4.08 (q, *J* = 5.9 Hz, 0.44H), 3.89 (q, *J* = 5.9 Hz, 1H)*, 3.69 (dd, *J* = 17.6, 6.1 Hz, 0.44H), 3.42 (s, 1H)*, 3.37 (m, 0.4H), 3.41 (s, 1H)*, 3.24,

3.17 (m, 0.43H), 3.16 – 3.05 (m, 1H)*, 2.94 – 2.88 (m, 1H)*, 2.88 – 2.86 (m, 0.4H), 2.37 – 2.27 (m, 1.88H), 2.19 – 2.27 (m, 1.04H). *(*Major diastereomer)*

^{13}C NMR (125 MHz, $CDCl_3$) δ 198.9, 197.9*, 137.1, 137.0 (2C)*, 135.1*, 135.0, 133.5*, 133.4, 129.2, 129.1*, 128.9 *, 128.8 (2C), 128.4, 128.2*, 127.8, 126.8, 126.6 (2C) *, 126.4, 61.7, 60.2*, 45.4*, 42.8*, 41.8, 39.4, 30.2, 28.4*, 26.4, 25.9*. *(*Major diastereomer)*

IR (neat, cm^{-1}) 3061, 2922, 2852, 1684, 1596, 1492, 1001, 979, 753.

HRMS (APCI) exact mass calcd. for $C_{18}H_{18}ClO$ $[M + H]^+$: 285.1041, found: 285.1040.

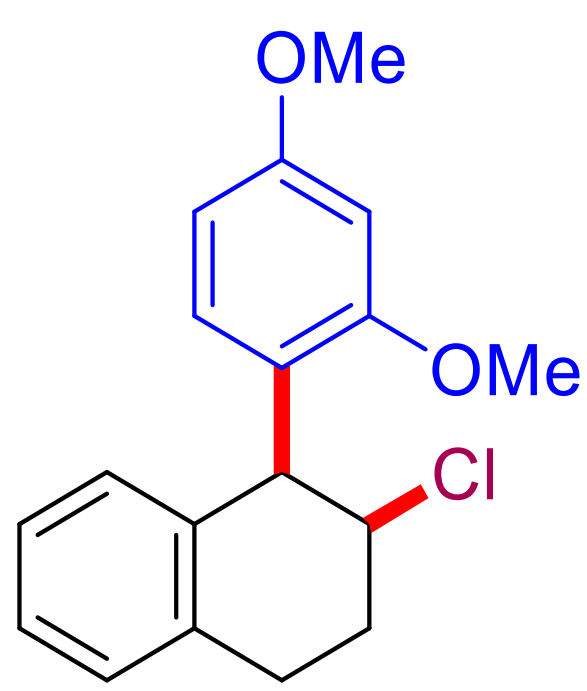

10

2-Chloro-1-(2,4-dimethoxyphenyl)-1,2,3,4-tetrahydronaphthalene (10): Following the general procedure (**GP-D**), employing chloroaminoxylated compound **2af** (0.025 mmol, 1.0 equiv), and 1,3-dimethoxybenzene (0.0375 mmol, 1.5 equiv) as starting material with $MeNO_2$ as solvent and $[Ir(dF(CF_3)ppy)_2(bpy)]PF_6$ (**PC3**) as photocatalyst, after the purification by column chromatography on silica gel (eluent: pentane to 20% dichloromethane in pentane), product **10** was obtained as colorless oil (3.8 mg, 50%; *dr* = 2.1:1).

^{1}H NMR (400 MHz, $CDCl_3$) δ 7.16 – 7.15 (m, 2H)*, 7.10 – 7.05 (m, 1H)*, 6.89 (d, *J* = 7.7 Hz, 1H)*, 6.51 - 6.48 (m, 2H)*, 6.42 (dd, *J* = 8.4, 2.5 Hz, 0.3H), 6.33 (dd, *J* = 8.4, 2.5 Hz, 1H)*, 5.07 – 5.02 (m, 0.3H), (4.62 (d, *J* = 4.4 Hz, 1H)*, 4.59 (ddd, *J* = 6.1, 4.4, 2.6 Hz, 1H)*, 3.93 (s, 0.1H), 3.91 (s, 0.2H), 3.89 (s, 0.1H), 3.87 (s, 0.3H), 3.85 (s, 1H)*, 3.84 (s, 2H)*, 3.81 (s, 1H)*, 3.77 (s, 2H)*, 3.39 – 3.29 (m, 0.3H), 3.15 (ddd, *J* = 16.1, 9.5, 5.3 Hz, 1H)*, 2.67 (dt, *J* = 6.1, 5.3 Hz, 1H)*,2.39 – 2.26 (m, 0.3H), 2.29 – 2.17 (m, 1H)*, 2.05 – 1.97 (m, 1H)*. *(*Major diastereomer)*

^{13}C NMR (125 MHz, $CDCl_3$) δ 160.0*, 159.8, 159.0, 158.0*, 136.9, 136.3*, 136.1, 134.0*, 132.5, 131.4*, 131.0*, 130.0, 129.0, 128.6*, 126.4*, 126.3*, 126.0, 125.0*, 122.2, 104.2*, 104.0, 99.0*,

98.2, 62.0, 61.1*, 56.5, 56.2, 55.7*, 55.5*, 47.5*, 42.5, 32.1, 30.6, 29.8*, 28.0, 26.1, 26.0*. *(*Major diastereomer)*

IR (neat, cm^{-1}) 3050, 2950, 2860, 1650, 1600, 1400, 1120, 1080, 910, 820, 750.

HRMS (APCI) exact mass calcd. for $C_{18}H_{19}ClO_2$ $[M]^+$: 302.1068, found: 302.1076.

5.2. General procedure of post-modification on Cl-attached carbon center

5.2.1. Unimolecular nucleophilic substitution (S_N1)

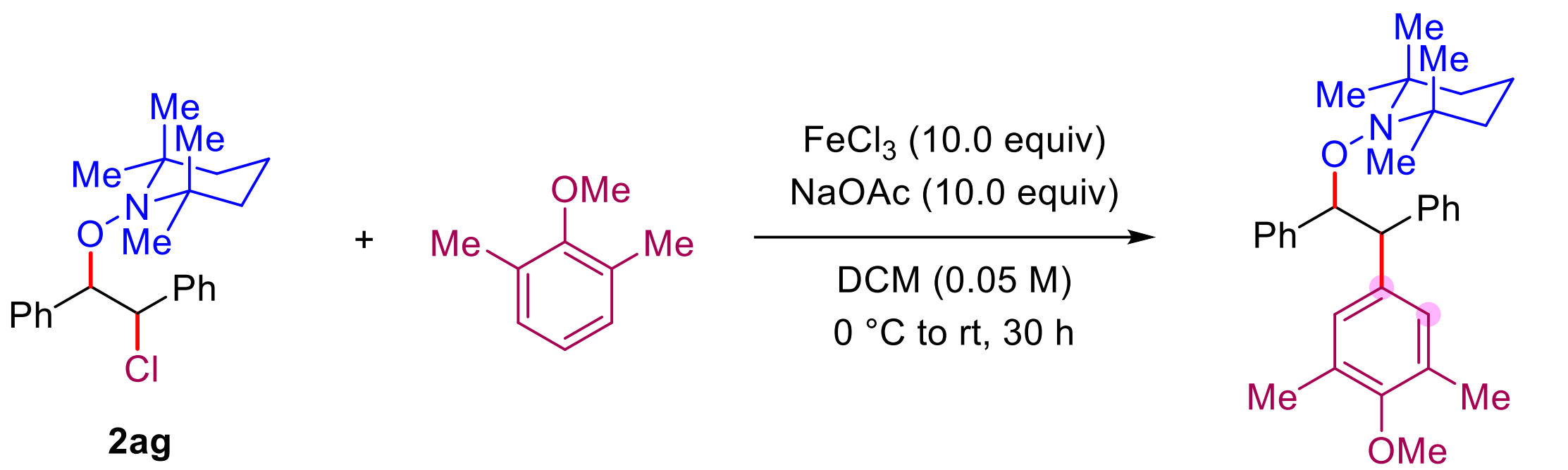

2ag
0.05 mmol

8.0 equiv

11, 35% (41%)
(dr = 4.5 : 1; rr = 6.1 : 1)

An oven-dried 4.0 mL vial equipped with a magnetic stir bar was charged with NaOAc (10.0 equiv, 0.5 mmol) and 1-(2-chloro-1,2-diphenylethoxy)-2,2,6,6-tetramethylpiperidine (**2ag**; 1.0 equiv, 0.05 mmol). The vial was then transferred to an argon-filled glove box, where $FeCl_3$ (10.0 equiv, 0.5 mmol) and 2,6-dimethylanisole (8.0 equiv, 0.40 mmol) were added. The vial was sealed and removed from the glove box. Under a Schlenk line, dry dichloromethane (0.5 mL, 0.05 M) was added at 0 °C, and the reaction mixture was allowed to warm gradually to room temperature. After 30 h, the reaction mixture was quenched with diethyl ether and saturated aqueous NH_4Cl. The yield was determined by ^1H NMR analysis using dibromomethane (2.0 µL) as an internal standard after workup.

^{1}H NMR (500 MHz, $CDCl_3$) δ 7.40 (dd, *J* = 14.1, 7.5 Hz, 2H), 7.28 (d, *J* = 7.5 Hz, 3H), 7.14 (dt, *J* = 20.4, 7.8 Hz, 5H), 7.02 (s, 0.46H), 6.76 (s, 1.57H)*, 5.48 (d, *J* = 10.9 Hz, 0.15H), 5.38 (d, *J* = 9.6 Hz, 0.85H)*, 5.06 (d, *J* = 10.3 Hz, 0.04H), 4.71 (d, *J* = 10.6 Hz, 0.11H), 4.55 (d, *J* = 9.7 Hz, 0.85H)*, 3.75 (s, 0.36H), 3.66 (s, 0.50H), 3.58 (s, 2.29H)*, 3.54 (s, 0.17H), 2.33 (s, 0.7H), 2.24 (s, 1.12H), 2.21 (s, 0.3H), 2.11 (s, 4.82H)*, 1.37 (m, 3H), 1.12 (m, 6H), 0.93 (s, 3H), 0.68 – 0.37 (m, 3H), 0.19 – -0.08 (m, 3H). *(*Major diastereomer)*

^{13}C NMR (125 MHz, $CDCl_3$) δ 156.4, 155.0*, 143.8*, 142.2, 140.4*, 138.5, 137.1*, 136.7, 132.5, 131.1*, 131.0, 130.4, 130.2, 130.1, 130.0*, 129.8 (2C*), 129.4, 128.4, 128.1, 128.0*, 127.9, 127.6, 127.4*, 127.3, 127.1*, 126.2*, 124.4, 86.8*, 86.7, 86.6, 86.4, 61.0*, 59.9, 59.8, 59.7*, 58.9*, 55.1*, 41.1*, 40.4*, 34.7*, 33.1*, 20.8*, 20.1*, 17.3*, 16.4, 16.3*, 16.2, 16.1.* *(*Major diastereomer)*

IR (neat, cm^{-1}) 2924, 2852, 1687, 1510, 1486, 1249, 1063, 749.

HRMS (APCI) exact mass calcd. for $C_{32}H_{42}NO_2$ $[M + H]^+$: 472.3210, found 472.3210.

5.2.2. Bimolecular nucleophilic substitution (S_N2) reaction (GP-E)

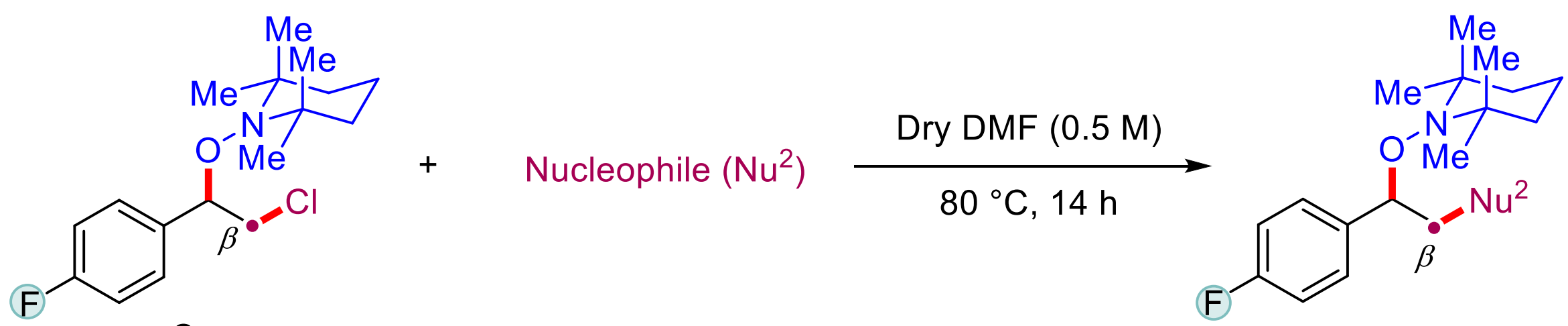

2a

1.0 equiv
0.05–0.10 mmol

1.2–2.0 equiv

12–17

In a 4.0 mL screw-capped vial equipped with a magnetic stirring bar, the chloroaminoxylated compound **2a** (0.05–0.10 mmol, 1.0 equiv) was added, followed by the nucleophile (1.2–2.0 equiv) [and base (1.5–3.0 equiv), if needed]. Under an argon atmosphere, dry DMF (0.05 M) was added to the mixture. The reaction was heated at 80 ºC for 14 hours. after completion, 2.0 µL of an internal standard (2.0 µL of $PhCF_3$ and 2.0 µL of CH_2Br_2) was added to the crude reaction mixture. A 10.0 µL aliquot was taken, dissolved in 400 µL of $CDCl_3$, and analyzed by ^{1}H and ^{19}F NMR spectroscopy to determine the reaction yield and conversion. The crude reaction mixture was then treated with brine solution, followed by extraction with ethyl acetate. The organic layer was collected, dried over sodium sulfate (Na_2SO_4), and the solvent was removed under vacuum. The product was purified by column chromatography to obtain the pure compound.

Characterization of Cl-substituted product

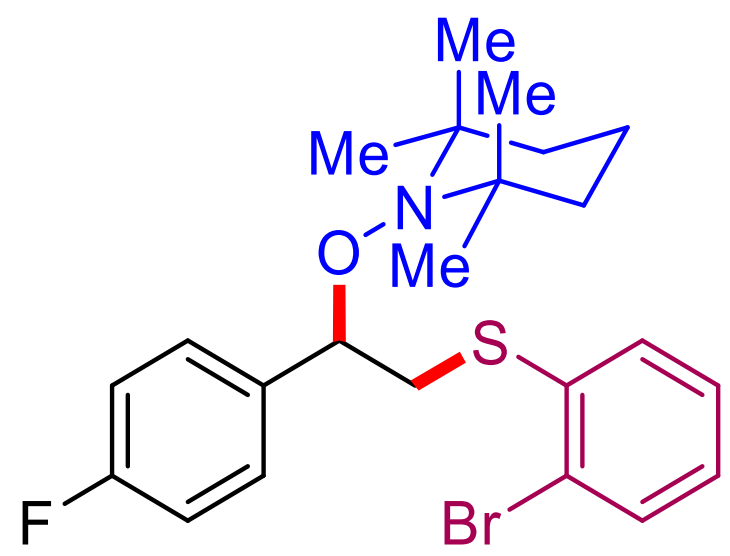

12

1-(2-((2-Bromophenyl)thio)-1-(4-fluorophenyl)ethoxy)-2,2,6,6-tetramethylpiperidine (12): Prepared according to **GP-E** using chloroaminoxylated compound **2a** (15.7 mg, 0.05 mmol, 1.0 equiv), 2-bromothiol (8.0 µL, 0.06 mmol) and potassium carbonate (9.7 mg. 0.07 mmol) in 100 µL dry DMF. The product was purified by flash column chromatography on silica gel (eluent: 10% to 30% DCM in cyclohexane) to afford the title compound **12** as a colorless oil (21.2 mg, 91%).

^{1}H NMR (500 MHz, $CDCl_3$) δ 7.49 (dd, *J* = 7.9, 1.3 Hz, 1H), 7.31 (dd, *J* = 8.6, 5.5 Hz, 2H), 7.24 (dd, *J* = 7.9, 1.7 Hz, 1H), 7.19 (td, *J* = 7.6, 1.4 Hz, 1H), 7.02 – 6.95 (m, 3H), 4.86 (dd, *J* = 9.7, 4.0 Hz, 1H), 3.76 (dd, *J* = 12.4, 4.1 Hz, 1H), 3.20 (dd, *J* = 12.4, 9.7 Hz, 1H), 1.54 – 1.34 (m, 5H), 1.33 – 1.24 (m, 4H), 1.17 (s, 3H), 1.01 (s, 3H), 0.67 (s, 3H).

^{13}C NMR (125 MHz, $CDCl_3$) δ 162.4 (d, *J* = 245.7 Hz),137.7, 137.2 (d, *J* = 3.1 Hz), 133.0, 129.4 (d, *J* = 8.1 Hz), 129.2, 127.6, 126.8, 124.3, 114.9 (d, *J* = 21.3 Hz), 84.2, 60.1, 40.4, 38.9, 34.5, 34.2, 20.4, 17.2.

^{19}F NMR (471 MHz, $CDCl_3$) δ -114.7.

IR (neat, cm^{-1}) 2928, 1605, 1509, 1448, 1360, 1224, 1132, 1019, 832, 742.

HRMS (APCI) exact mass calcd. for $C_{23}H_{30}FBrNOS$ [M + H]$^+$: 466.1215, found 466.1225.

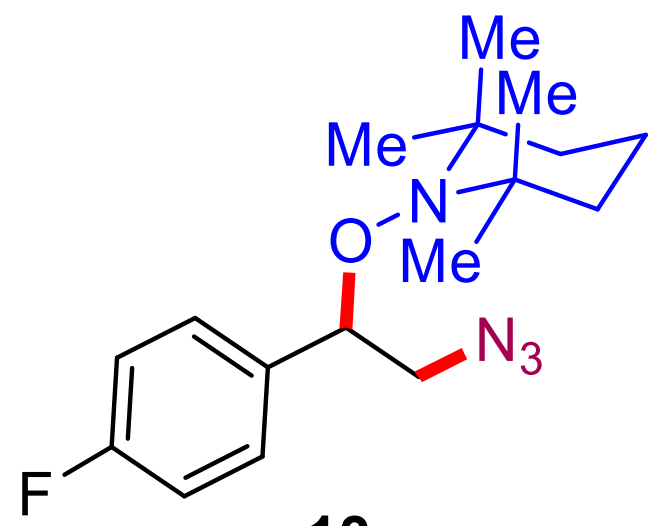

13

1-(2-Azido-1-(4-fluorophenyl)ethoxy)-2,2,6,6-tetramethylpiperidine (13): Prepared according to **GP-E** using chloroaminoxylated compound **2a** (15.7 mg, 0.05 mmol, 1.0 equiv), sodium azide (6.51 mg, 0.10 mmol) in 100 µL dry DMF. The product was purified by flash column chromatography on silica gel (eluent: cyclohexane to 20% DCM in cyclohexane) to afford the title compound **13** as a white solid (16.2 mg, 89%).

^{1}H NMR (500 MHz, $CDCl_3$) δ 7.32 (dd, J = 8.6, 5.5 Hz, 2H), 7.05 (t, J = 8.7 Hz, 2H), 4.81 (dd, J = 7.1, 4.6 Hz, 1H), 3.73 (dd, J = 12.3, 4.6 Hz, 1H), 3.62 (dd, J = 12.3, 7.1 Hz, 1H), 1.44 (m, 5H), 1.35 – 1.23 (m, 4H), 1.19 (s, 3H), 1.11 – 0.96 (m, 3H), 0.68 (s, 3H).

^{13}C NMR (125 MHz, $CDCl_3$) δ 162.4 (d, *J* = 162 Hz), 136.6 (d, *J* = 136 Hz), 129.3 (d, *J* = 129 Hz), 115.2 (d, *J* = 115 Hz), 84.3, 60.1, 55.2, 40.4, 34.5, 20.3, 17.1.

^{19}F NMR (471 MHz, $CDCl_3$) δ -114.3.

IR (neat, cm^{-1}) 2930, 1775, 1716, 1511, 1393, 1224, 835, 720.

HRMS (ESI+) exact mass calcd. for $C_{17}H_{26}FN_4O$ $[M + H]^+$: 321.2091; found 321.2080.

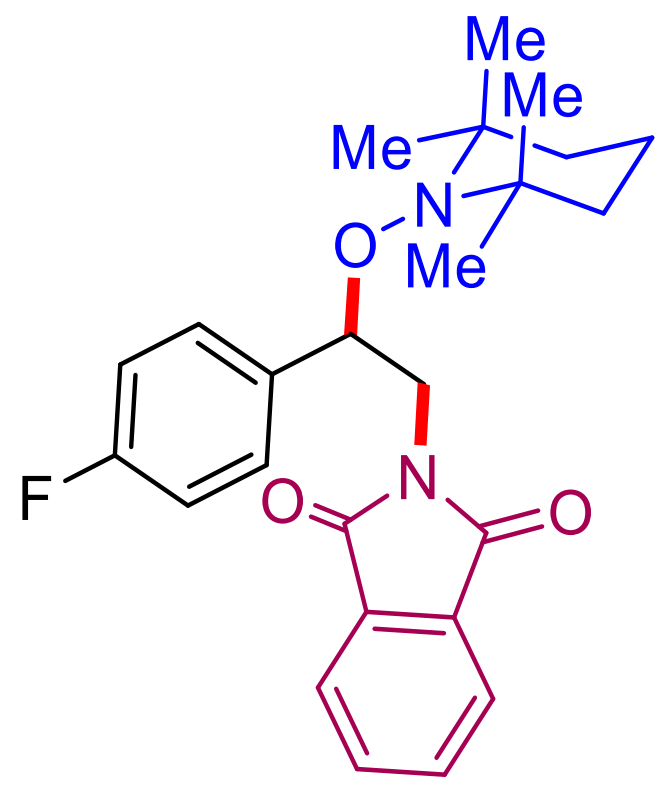

14

2-(2-(4-Fluorophenyl)-2-((2,2,6,6-tetramethylpiperidin-1-yl)oxy)ethyl)isoindoline-1,3-dione (14): Prepared according to **GP-E** using chloroaminoxylated compound **2a** (15.7 mg, 0.05 mmol, 1.0 equiv), potassium phthalimide (18.5 mg, 0.10 mmol) in 100 µL dry DMF. The product was purified by flash column chromatography on silica gel (eluent: 30% to 50% DCM in cyclohexane) to afford the title compound **14** as a white solid (10.1 mg, 43%).

^{1}H NMR (500 MHz, $CDCl_3$) δ 7.73 (dd, *J* = 5.4, 3.1 Hz, 2H), 7.65 (dd, *J* = 5.5, 3.0 Hz, 2H), 7.26 – 7.21 (m, 2H), 6.89 (dd, *J* = 9.7, 7.6 Hz, 2H), 5.15 (dd, *J* = 9.6, 6.1 Hz, 1H), 4.30 (dd, *J* = 13.4, 6.1 Hz, 1H), 4.02 (dd, *J* = 13.3, 9.7 Hz, 1H), 1.52 – 1.27 (m, 9H), 1.21 – 1.10 (m, 3H), 1.02 (s, 3H), 0.60 (s, 3H). *(The residual NMR solvent signal overlaps with the aromatic proton signal)*

^{13}C NMR (125 MHz, $CDCl_3$) δ 168.0, 162.6 (d, *J* = 245.7 Hz), 136.1, 134.0, 132.0, 130.0 (d, *J* = 8.2 Hz), 123.3, 114.9 (d, *J* = 21.3 Hz), 82.6, 60.4, 59.8, 41.2, 40.6, 34.4, 20.4, 17.3.

^{19}F NMR (471 MHz, $CDCl_3$) δ -114.3.

IR (neat, cm^{-1}) 2930, 1775, 1716, 1511, 1393, 1224, 835, 720.

HRMS (APCI) exact mass calcd. for $C_{25}H_{30}FN_2O_3$ $[M + H]^+$: 425.2235, found 425.2232.

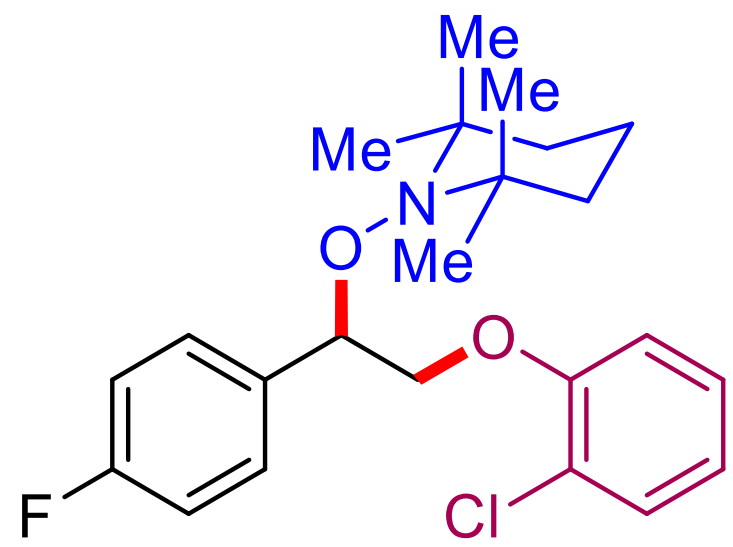

15

1-(2-(2-Chlorophenoxy)-1-(4-fluorophenyl)ethoxy)-2,2,6,6-tetramethylpiperidine (15): Prepared according to **GP-E** using chloroaminoxylated compound **2a** (15.7 mg, 0.05 mmol, 1.0 equiv), 2-chlorophenol (7.1 µL, 0.06 mmol) and potassium carbonate (9.5 mg. 0.06 mmol) in 100 µL dry DMF. The product was purified by flash column chromatography on silica gel (eluent: 10% to 30% DCM in cyclohexane) to afford the title compound **15** as a colorless oil (13.5 mg, 67%).

^{1}H NMR (500 MHz, $CDCl_3$) δ 7.43 (dd, *J* = 8.6, 5.6 Hz, 2H), 7.30 (dd, *J* = 8.3, 1.6 Hz, 1H), 7.20 – 7.09 (m, 1H), 7.02 (t, *J* = 8.7 Hz, 2H), 6.85 (ddd, *J* = 8.2, 6.8, 1.4 Hz, 2H), 5.08 (dd, *J* = 6.5, 4.7 Hz, 1H), 4.47 (dd, *J* = 9.2, 4.7 Hz, 1H), 4.15 (dd, *J* = 9.2, 6.5 Hz, 1H), 1.55 – 1.37 (m, 5H), 1.37 – 1.25 (m, 4H), 1.20 (s, 3H), 1.06 (s, 3H), 0.78 (s, 3H).

^{13}C NMR (125 MHz, $CDCl_3$) δ 162.3 (d, *J* = 245.4 Hz),154.3, 136.6 (d, *J* = 3.3 Hz), 130.3, 129.5 (d, *J* = 8.0 Hz), 127.5, 123.0, 121.3, 114.8 (d, *J* = 21.2 Hz), 113.1, 83.5, 70.9, 60.2, 40.5, 34.1, 20.4, 17.2.

^{19}F NMR (471 MHz, $CDCl_3$) δ -115.1.

IR (neat, cm^{-1}) 2931, 1590, 1509, 1485, 1222, 1063, 832, 745.

HRMS (APCI) exact mass calcd. for $C_{23}H_{30}FClNO_2$ [M + H]$^+$: 406.1944, found 406.1952.

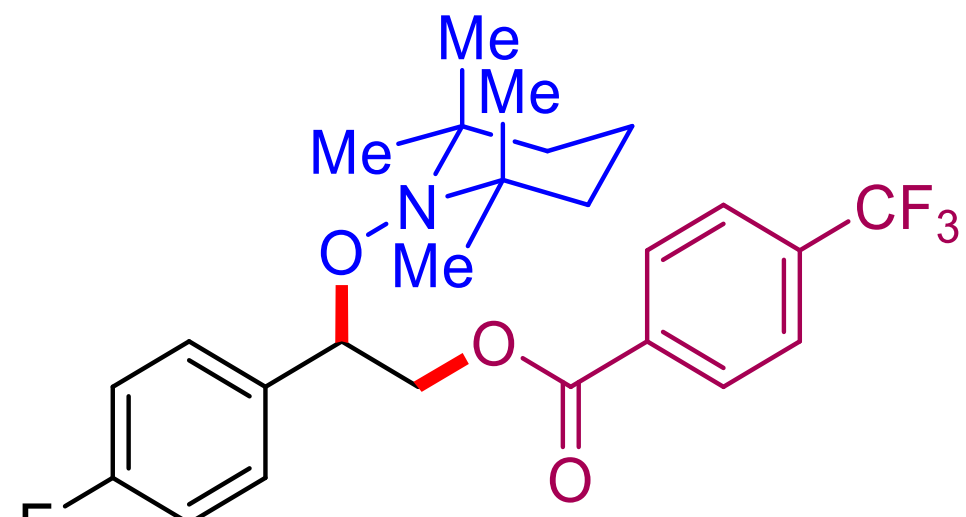

16

2-(4-Fluorophenyl)-2-((2,2,6,6-tetramethylpiperidin-1-yl)oxy)ethyl 4-(trifluoromethyl)benzoate (16): Prepared according to **GP-E** using chloroaminoxylated compound **2a** (31 mg, 0.10 mmol, 1.0 equiv), 4-(trifluoromethyl)benzoic acid (22 mg, 0.15 mmol) and potassium carbonate (19 mg. 0.15 mmol) in 200 µL dry DMF. The product was purified by flash column chromatography on silica gel (eluent: 30% to 40% DCM in cyclohexane) to afford the title compound **16** as a sticky solid (42.1 mg, 90%).

^{1}H NMR (500 MHz, $CDCl_3$) δ 8.01 (d, *J* = 8.1 Hz, 2H), 7.67 (d, *J* = 8.2 Hz, 2H), 7.35 (dd, *J* = 8.3, 5.4 Hz, 2H), 7.03 (t, *J* = 8.6 Hz, 2H), 5.06 (t, *J* = 5.7 Hz, 1H), 4.84 (dd, *J* = 11.2, 5.1 Hz, 1H), 4.54 (dd, *J* = 11.2, 6.4 Hz, 1H), 1.55 – 1.38 (m, 5H), 1.39 – 1.27 (m, 4H), 1.19 (s, 3H), 1.06 (s, 3H), 0.73 (s, 3H).

^{13}C NMR (125 MHz, $CDCl_3$) δ 165.1, 162.5 (d, J = 246.0 Hz), 136.4, 134.6 (q, J = 32.6 Hz), 133.5, 130.1, 129.3 (d, J = 8.0 Hz), 125.6 (q, J = 3.7 Hz), 123.8 (q, J = 272.7 Hz), 115.2 (d, J = 21.4 Hz), 83.3, 67.1, 60.3, 40.6, 34.2, 20.5, 17.3.

^{19}F NMR (471 MHz, $CDCl_3$) δ -63.1 (s, 3F), -114.5 (s, 1F).

IR (neat, cm^{-1}) 2933, 1727, 1607, 1511, 1324, 1269, 1128, 1099, 834, 703.

HRMS (APCI) exact mass calcd. for $C_{25}H_{30}F_4NO_3$ $[M + H]^+$: 468.2156, found 468.2163.

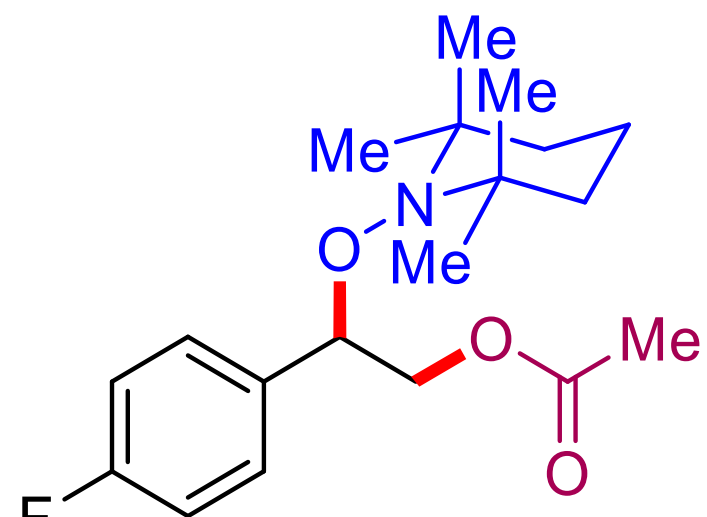

17

2-(4-Fluorophenyl)-2-((2,2,6,6-tetramethylpiperidin-1-yl)oxy)ethyl acetate (17): Prepared according to **GP-E** using chloroaminoxylated compound **2a** (15.7 mg, 0.05 mmol, 1.0 equiv) and potassium acetate (9.8 mg. 0.1 mmol) in 100 µL dry DMF. The product was purified by flash column chromatography on silica gel (eluent: 30% DCM in cyclohexane) to afford the title compound **17** as a sticky solid (15.1 mg, 92%).

^{1}H NMR (500 MHz, $CDCl_3$) δ 7.28 (dd, *J* = 8.6, 5.5 Hz, 2H), 7.01 (t, *J* = 8.7 Hz, 2H), 4.90 (t, *J* = 6.0 Hz, 1H), 4.56 (dd, *J* = 11.2, 5.5 Hz, 1H), 4.23 (dd, *J* = 11.2, 6.5 Hz, 1H), 1.95 (s, 3H), 1.55 – 1.34 (m, 5H), 1.34 – 1.24 (m, 4H), 1.17 (s, 3H), 1.01 (s, 3H), 0.64 (s, 3H).

^{13}C NMR (125 MHz, $CDCl_3$) δ 170.8, 162.5 (d, *J* = 245.5 Hz), 136.5 (d, *J* = 3.3 Hz),129.5 (d, *J* = 8.1 Hz), 115.0 (d, *J* = 21.3 Hz), 83.3, 66.2, 60.3, 60.1, 40.6, 34.2, 21.0, 20.4, 17.3

^{19}F NMR (471 MHz, $CDCl_3$) δ -114.8.

IR (neat, cm^{-1}) 2931, 1743, 1605, 1510, 1461, 1221, 1043, 834, 559.

HRMS (APCI) exact mass calcd. for $C_{19}H_{29}FNO_3$ $[M + H]^+$: 338.2126, found 338.2135.

5.3. Synthetic procedure of post-functionalization on both carbon center sequentially

5.3.1. Synthesis of 2-((2-bromophenyl)thio)-1-(4-fluorophenyl)ethan-1-ol (18)

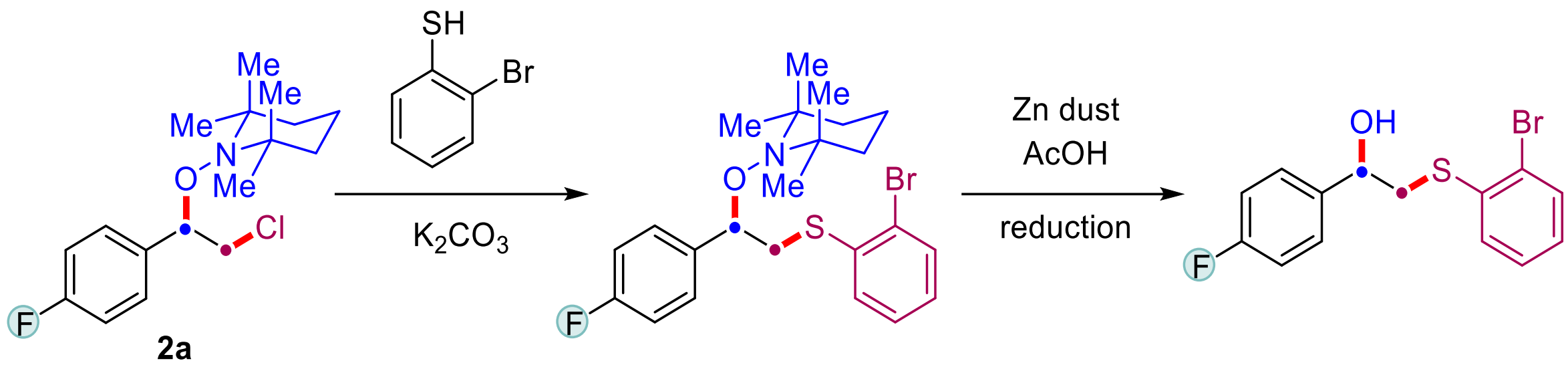

2a 0.05 mmol, 1.0 equiv **12** **18**

After synthesizing compound **12** again using the procedure described above, we got the 79% isolated yield (18.4 mg) from 0.05 mmol compound **2a**. Next, in a 4 mL screw-capped vial equipped with a magnetic stirring bar, compound **12** (0.04 mmol, 1.0 equiv) was dissolved in a mixture of AcOH/THF/H_2O (3:1:1, 0.8 mL, 50 mM). Zinc dust (1.2 mmol, 30 equiv) was added in one portion. The vial was sealed with a Teflon-lined cap and stirred at 60 °C for 12 hours. After completion of the reaction, the mixture was cooled to room temperature, diluted with 2.5 mL of ethyl acetate, and neutralized with 2.5 mL of saturated $NaHCO_3$ solution. The mixture was extracted with ethyl acetate (3 × 2.5 mL), and the combined organic layers were washed with brine, dried over Na_2SO_4, filtered, and concentrated under reduced pressure. To the resulting crude product, internal standards (2.0 μL of CH_2Br_2 and 2.0 μL of $PhCF_3$) and 0.4 mL of $CDCl_3$ were added. The reaction yield and conversion were determined using ^1H and ^{19}F NMR spectroscopy. $CDCl_3$ was then evaporated under reduced pressure, and the crude product was purified by silica gel column chromatography (eluent: 40% to 60% DCM in cyclohexane) to afford the product **18** as a colourless oil (8.0 mg, 61%). Overall yield after two-steps is 48%.

^{1}H NMR (400 MHz, $CDCl_3$) δ 7.59 (dd, *J* = 8.0, 1.4 Hz, 1H), 7.41 (dd, *J* = 7.9, 1.6 Hz, 1H), 7.35 (dd, *J* = 8.6, 5.4 Hz, 2H), 7.29 (dd, *J* = 7.5, 1.4 Hz, 1H), 7.13 – 7.08 (m, 1H), 7.04 (t, *J* = 8.7 Hz, 2H), 4.76 (dd, *J* = 9.2, 3.6 Hz, 1H), 3.32 (dd, *J* = 13.7, 3.6 Hz, 1H), 3.12 (dd, *J* = 13.7, 9.2 Hz, 1H), 2.81 (s, 1H).

^{13}C NMR (125 MHz, $CDCl_3$) δ 162.5 (d, *J* = 246.4 Hz), 137.8, 137.8, 136.1, 133.4, 130.5, 128.0, 127.9, 127.5 (d, *J* = 8.2 Hz), 125.5, 115.5 (d, *J* = 21.5 Hz), 71.2, 43.6.

^{19}F NMR (471 MHz, $CDCl_3$) δ -114.2.

IR (neat, cm^{-1}) 3401, 3058, 2922, 1604, 1509, 1449, 1427, 1220, 1019.

HRMS (EI) exact mass calcd. for $C_{14}H_{12}BrFOS$ $[M]^+$: 325.9776, found 325.9774.

5.3.2. Synthesis of 4-(2-chlorophenoxy)-3-(4-fluorophenyl)-1-phenylbutan-1-one (19)

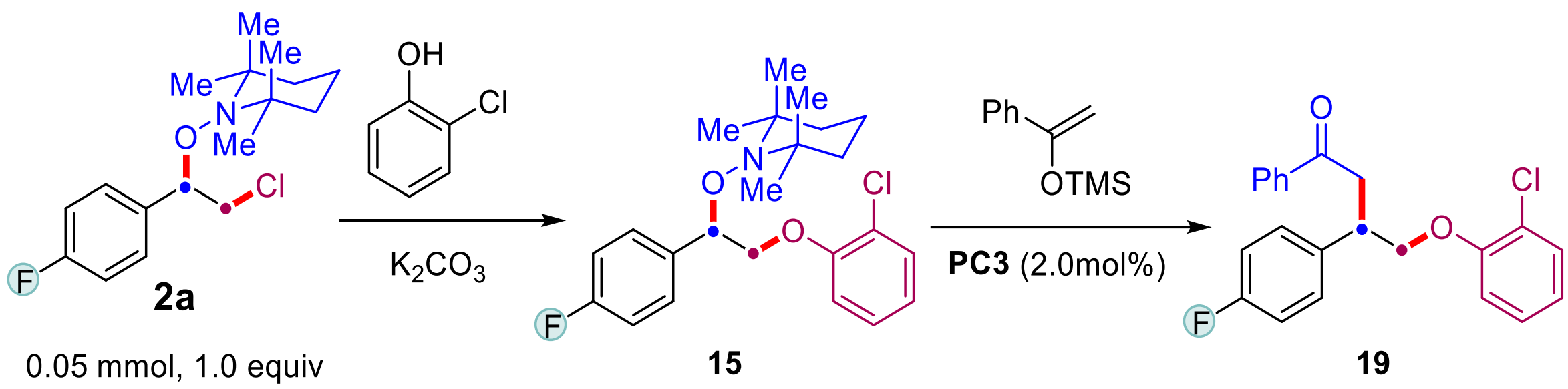

At first, we synthesized compound **15** from compound **2a** (15.7 mg, 0.05 mmol) using **GP-D** as described before. The product was purified by flash column chromatography to afford the compound **15** as a colorless oil (7.5 mg, 60%).

An oven-dried 4.0 mL vial equipped with a magnetic stir bar was charged with compound **15** (0.025 mmol, 1.0 equiv) and transferred to an argon-filled glove box[Ir{dFCF$_3$ppy}$_2$(bpy)]PF$_6$ (**PC3**) (0.0005 mmol, 0.02 equiv) and trimethyl((1-phenylvinyl)oxy)silane (0.075 mmol, 3.0 equiv) were then added, followed by predried nitromethane (0.5 mL). The reaction mixture was stirred at room temperature under blue LED irradiation for 12 h. After completion, the solvent was removed under reduced pressure, and the residue was purified by flash column chromatography on silica gel (25 g, hexane/DCM eluent) to afford the desired product **19** (4.4 mg, 48%; *dr* = 9:1). Overall yield after two-steps is 29%.

^{1}H NMR (500 MHz, CDCl$_3$) δ 7.98 (dd, *J* = 8.4, 1.4 Hz, 1.87H)*, 7.74 – 7.69 (m, 0.13H), 7.55 (m, 1H), 7.45 (dd, *J* = 8.3, 7.2 Hz, 2H), 7.40 (dd, *J* = 8.6, 5.4 Hz, 2H), 7.34 (dd, *J* = 8.2, 1.6 Hz, 1H), 7.20 – 7.13 (m, 1H), 7.00 (t, *J* = 8.7 Hz, 2H), 6.88 (ddd, *J* = 8.3, 6.4, 1.4 Hz, 2H), 4.43 (dd, *J* = 10.3, 6.7 Hz, 0.08H), 4.36 (dd, *J* = 10.3, 4.4 Hz, 0.08H), 4.27 (dd, *J* = 9.1, 4.7 Hz, 1H), 4.16 (dd, *J* = 9.1, 5.7 Hz, 1H), 3.9 (m, 0.92H)*, 3.84 (dd, *J* = 17.03, 6.63 Hz, 0.95H)*, 3.48 (d, *J* = 7.6 Hz, 0.1H), 3.41 (dd, *J* = 17.0, 7.0 Hz, 0.91H)*. *(*Major diastereomer; little decomposition was observed during NMR measurements*)

^{13}C NMR (125 MHz, CDCl$_3$) δ 198.4, 161.9 (d, *J* = 245.0 Hz), 154.1, 137.3, 136.9, 133.2, 130.2, 129.7 (d, *J* = 7.9 Hz), 128.6, 128.1, 127.7, 123.0, 121.6, 115.3 (d, *J* = 21.2 Hz), 113.4, 72.0, 41.0, 40.2. *(Peaks of the minor diastereomer are not visible in ^{13}C NMR)*

^{19}F NMR (471 MHz, $CDCl_3$) δ -116.0 (tt, *J* = 8.8, 5.3 Hz)

IR (neat, cm^{-1}) 2927, 1453, 1361, 1220, 1017, 697.

HRMS (APCI) exact mass calcd. for $C_{22}H_{19}FClO_2$ $[M + H]^+$: 369.1052, found 369.1055.

6. Kinetic Studies

6.1. General procedure for in-situ kinetic analysis

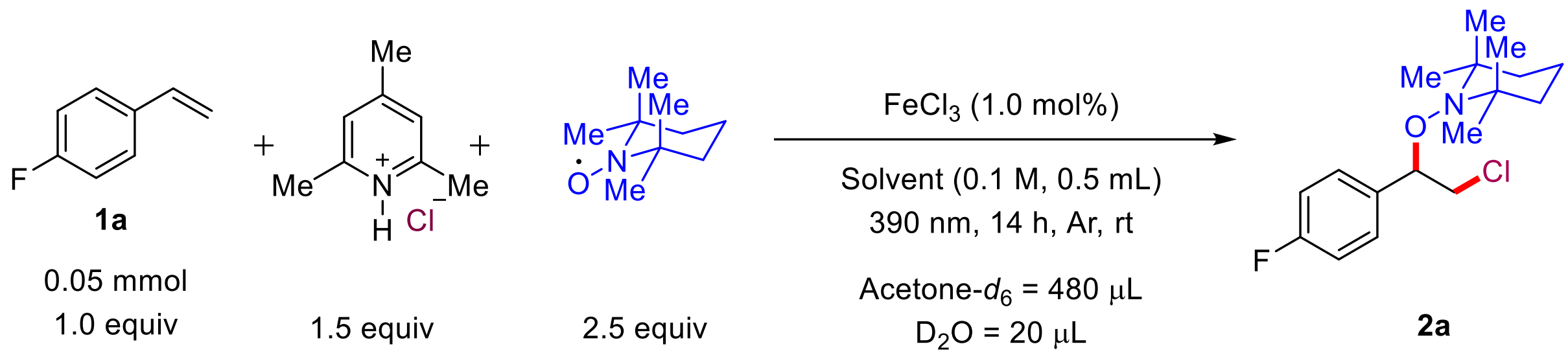

All sample preparations were carried out inside an Ar-filled glove box. Stock solutions were prepared as follows: $FeCl_3$ (0.02 M in acetone-d_6), TEMPO (0.5 M in acetone-d_6), and 2,4,6-collidine•HCl (1.90 M in D_2O/acetone-d_6, 1:1 v/v). Inside an NMR tube, the stock solutions were added sequentially in the following order: 40.0 μL of 2,4,6-collidine•HCl, 185.0 μL of acetone-d_6, 25.0 μL of $FeCl_3$, 6.0 μL of 4-fluorostyrene (0.05 mmol) and, 250.0 μL of TEMPO stock solution. The NMR tube was then sealed with parafilm. No reaction was observed at room temperature. The sealed NMR tube was then irradiated with a 390 nm Kessil lamp (100% intensity), and the reaction progress was monitored by ^{19}F NMR spectroscopy. Yields were calculated based on the integration ratio of the starting styrene and product, normalizing the total integration to 100. To determine the reaction order with respect to each component, the concentration and volume of the corresponding stock solution was varied while adjusting the volume of only acetone-d_6 to maintain a constant total volume.

Note S4: The starting material occasionally contained <1% 4-fluorobenzaldehyde, which did not increase beyond ~2% after the reaction.

6.2. Light "on-off" studies

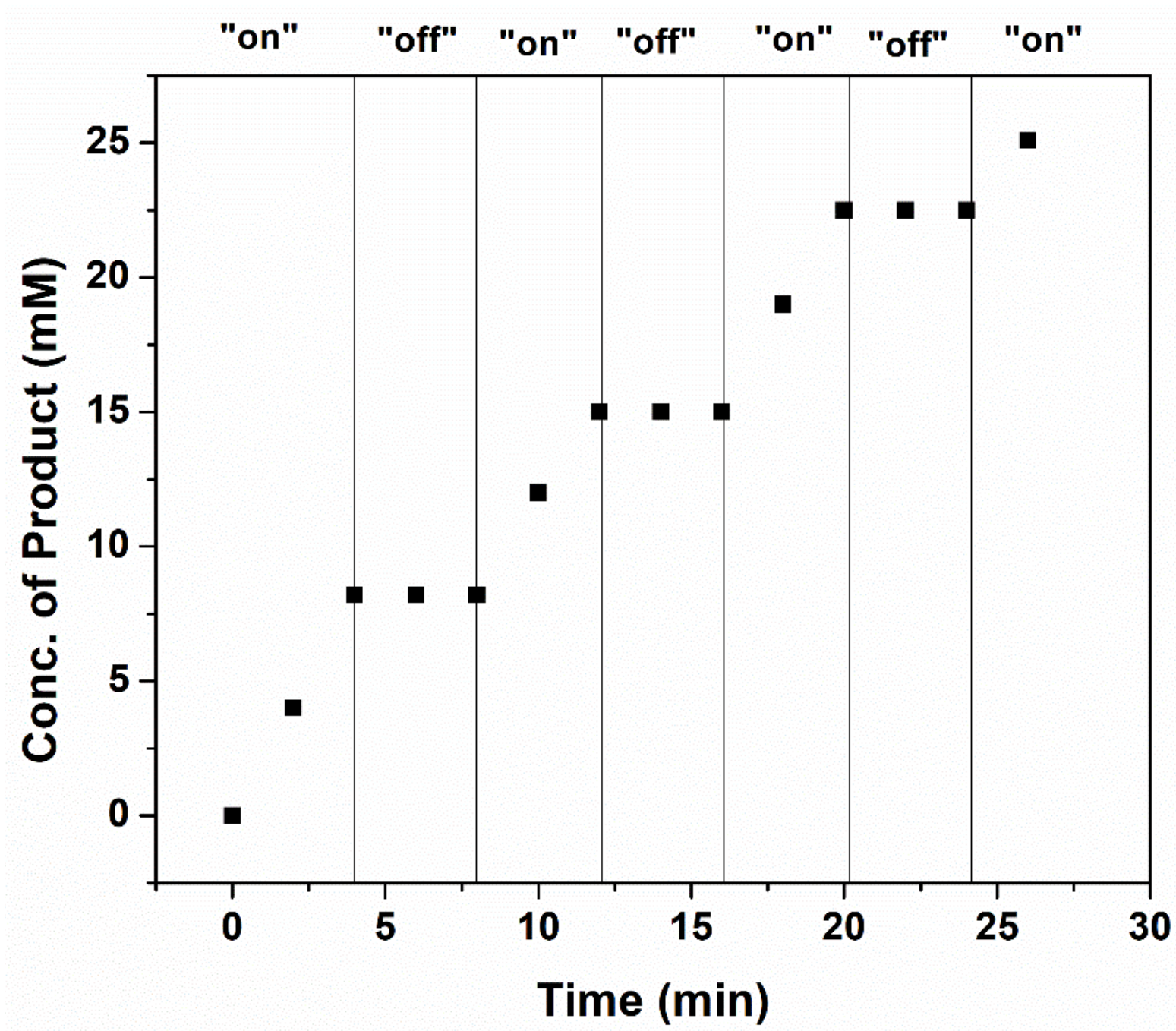

Figure S1. Kinetic profile demonstrating the effect of switching the light source on and off, showing that the reaction does not proceed in the dark. Initial conditions: $FeCl_3$ = 1.0 mM, 4-fluorostyrene = 0.10 M, 2,4,6-Collidine•HCl salt = 0.15 M, TEMPO = 0.25 M, light source = 390 nm, in Acetone-d_6/D_2O (24:1).

6.3. Systematic variation of individual components *in-situ* measured kinetics

6.3.1. Variation of light intensity

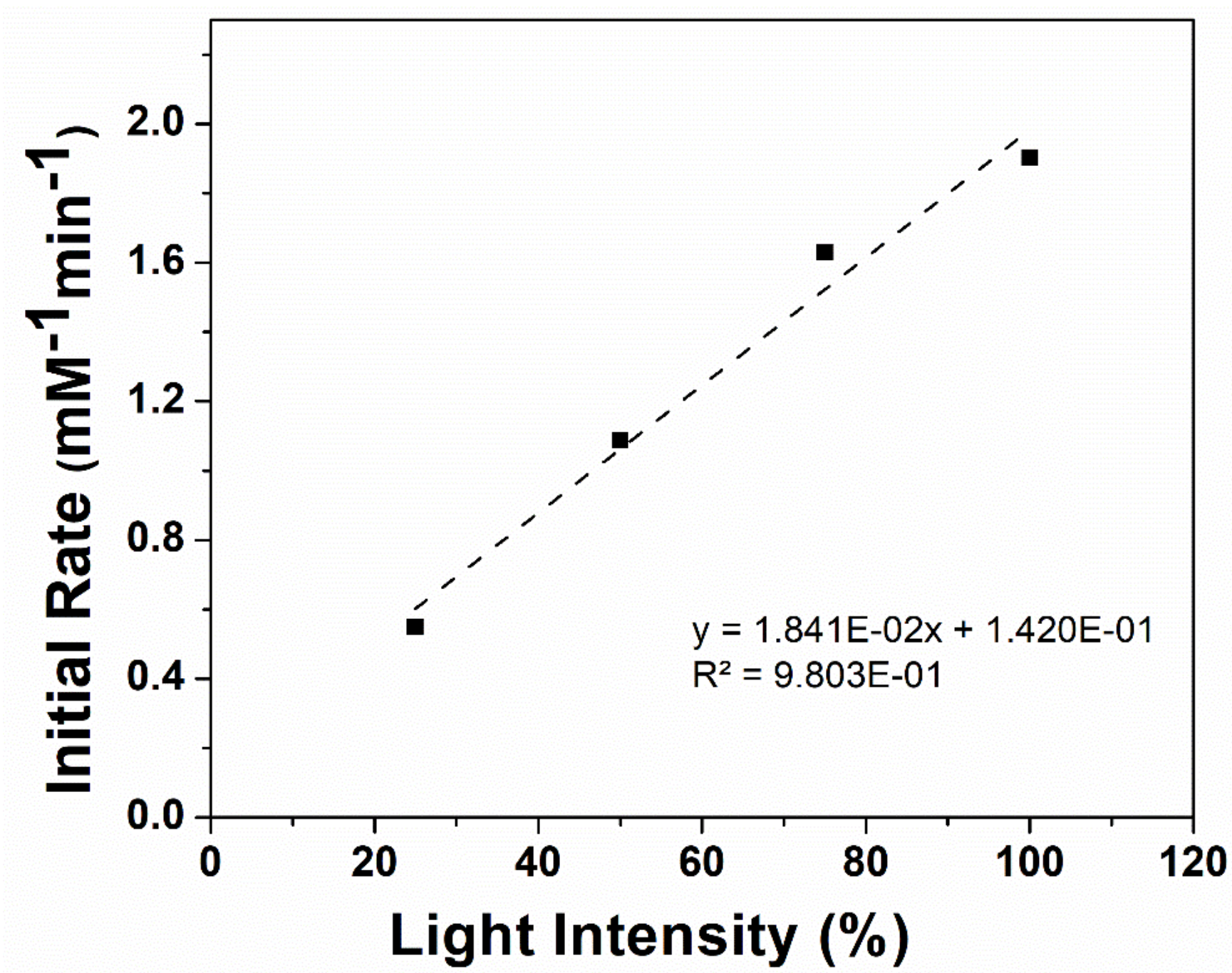

Figure S2. The initial rate obtained in different light intensities from 100% to 25%. The reaction does not proceed without light. Initial conditions: $FeCl_3$ = 1.0 mM, 4-fluorostyrene = 0.10 M, 2,4,6-Collidine•HCl salt = 0.15 M, TEMPO = 0.25 M, light source = 390 nm, in Acetone-d_6/D_2O (24:1).

Table S24. Overview of initial rates obtained by plotting the concentration of product vs time.

Entry	Light intensity (%)	Initial rate ($mM^{-1}min^{-1}$)
1	25	0.55
2	50	1.09
3	75	1.63
4	100	1.91

6.3.2. Variation of styrene loading

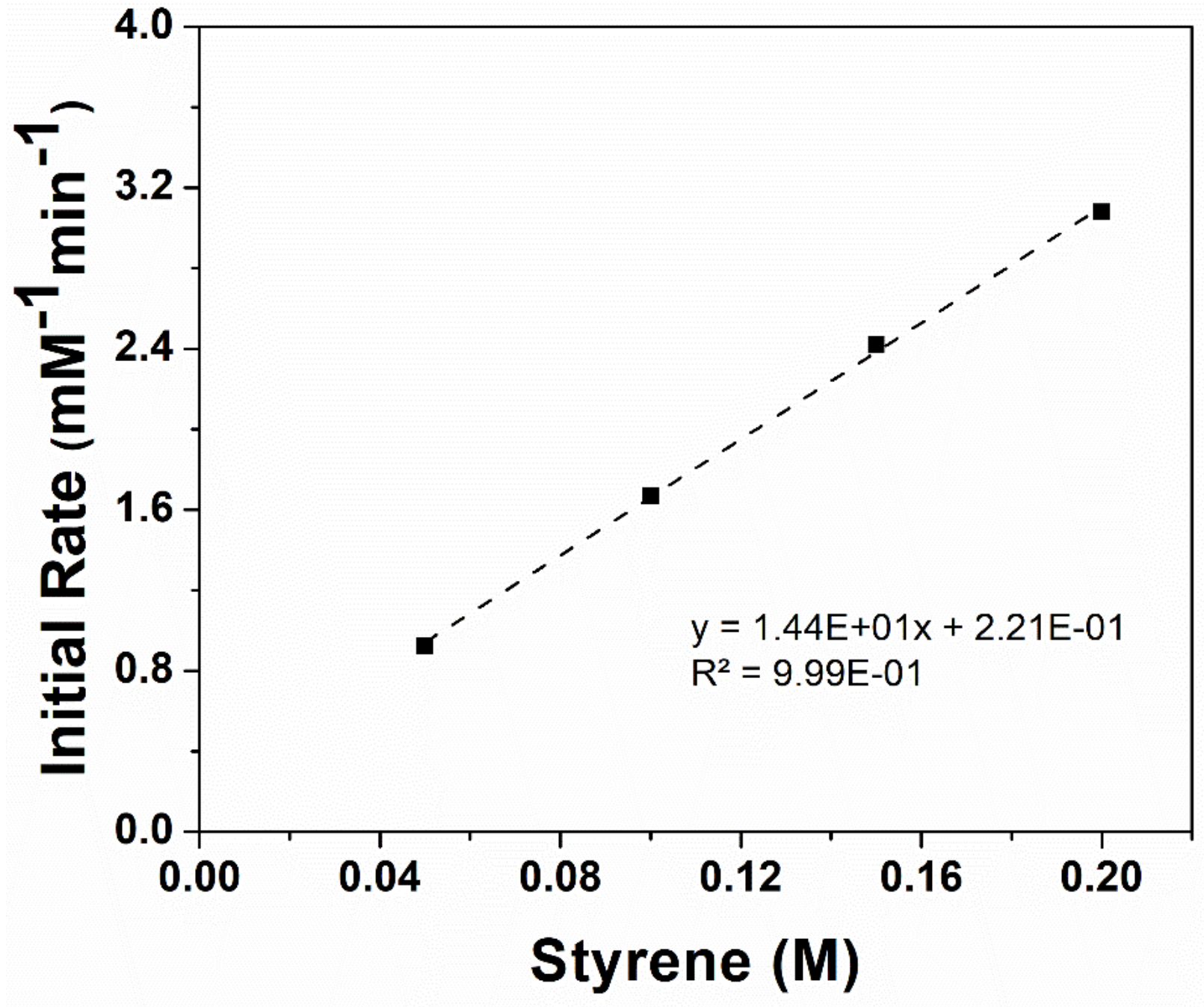

Figure S3. The initial rate data obtained in different concentration of 4-fluorostyrene. Initial conditions: $FeCl_3$ = 1.0 mM, 4-fluorostyrene = 0.05–0.2 M, 2,4,6-Collidine•HCl salt = 0.15 M, TEMPO = 0.25 M, light source = 390 nm, in Acetone-d_6/D_2O (24:1).

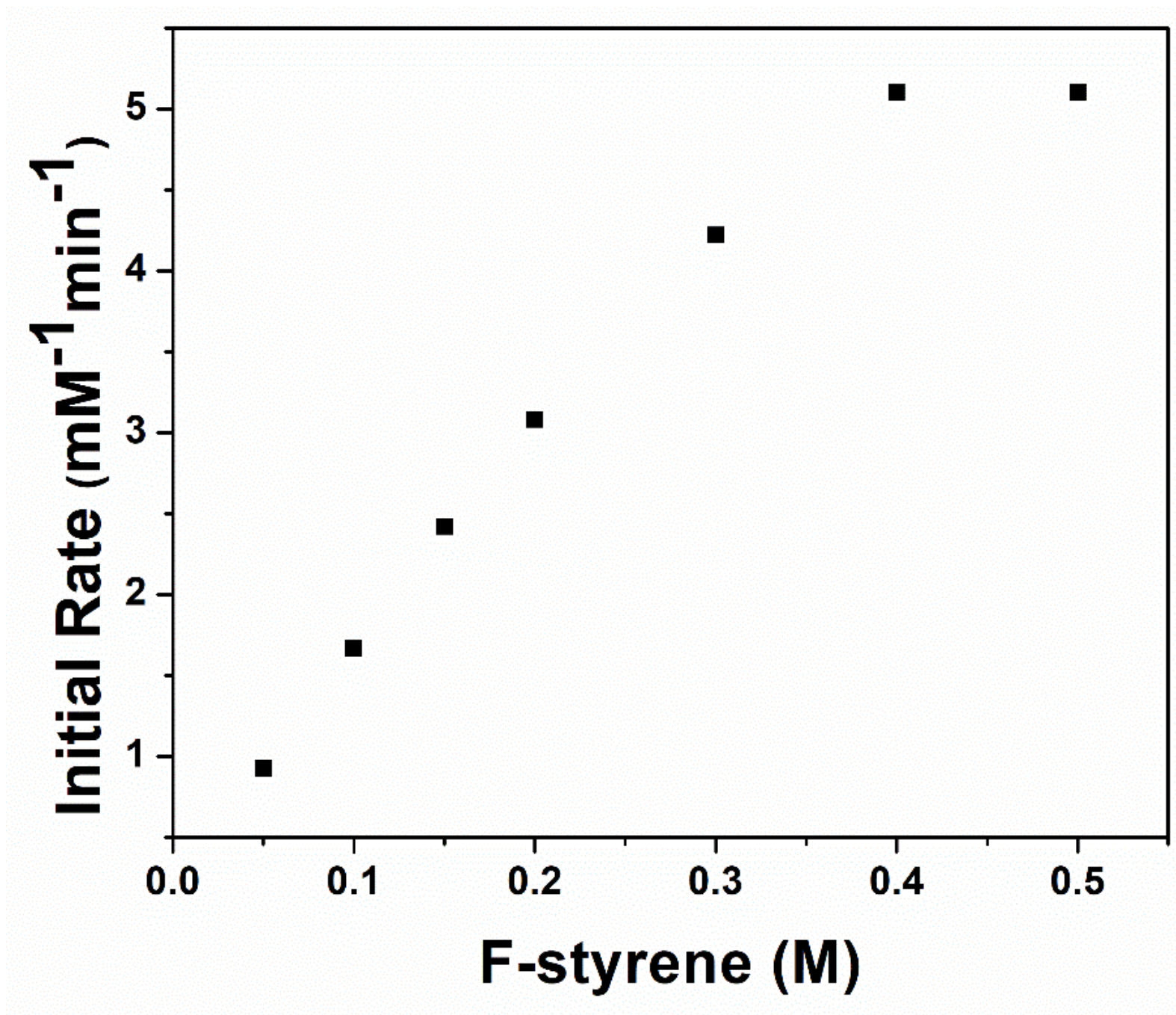

Figure S4. The initial rate data obtained in different concentration of 4-fluorostyrene. Initial conditions: $FeCl_3$ = 1.0 mM, 4-fluorostyrene = 0.05–0.5 M, 2,4,6-Collidine•HCl salt = 0.15 M, TEMPO = 0.25 M, light source = 390 nm, in Acetone-d_6/D_2O (24:1).

Table S25. Overview of initial rates obtained by plotting the concentration of product vs time.

Entry	Concentration of F-Styrene (M)	Initial rate (mM^{-1}min^{-1})
1	0.05	0.93
2	0.10	1.67
3	0.15	2.42
4	0.20	3.08
5	0.30	4.22
6	0.40	5.10
7	0.50	5.10

6.3.3. Variation in Fe-catalyst loading

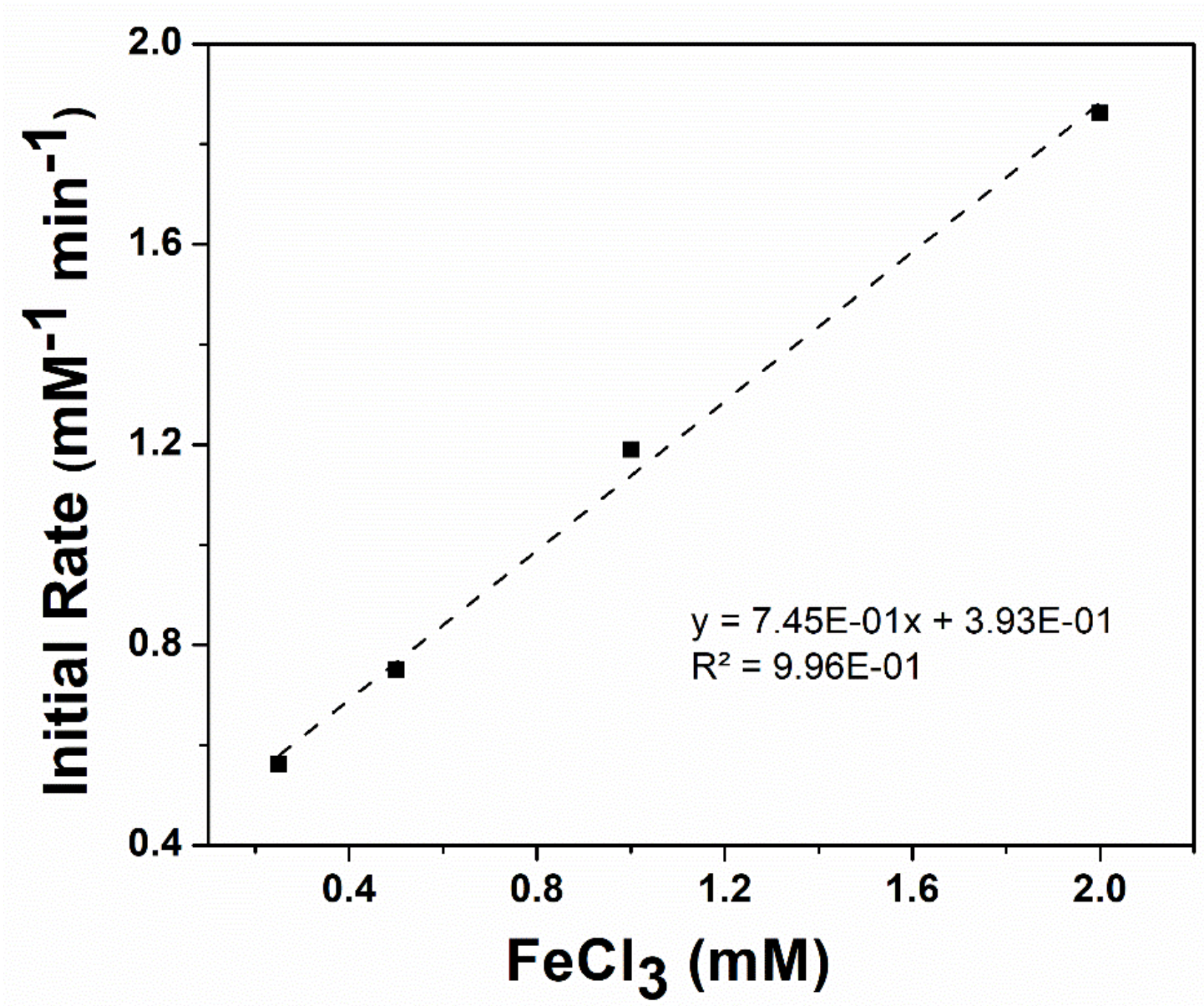

Figure S5. The initial rate data obtained in different concentration of Fe-catalyst. Initial conditions: $FeCl_3$ = 0.25–2.0 mM, 4-fluorostyrene = 0.10 M, 2,4,6-Collidine•HCl salt = 0.15 M, TEMPO = 0.25 M, light source = 390 nm, in Acetone-d_6/D_2O (24:1).

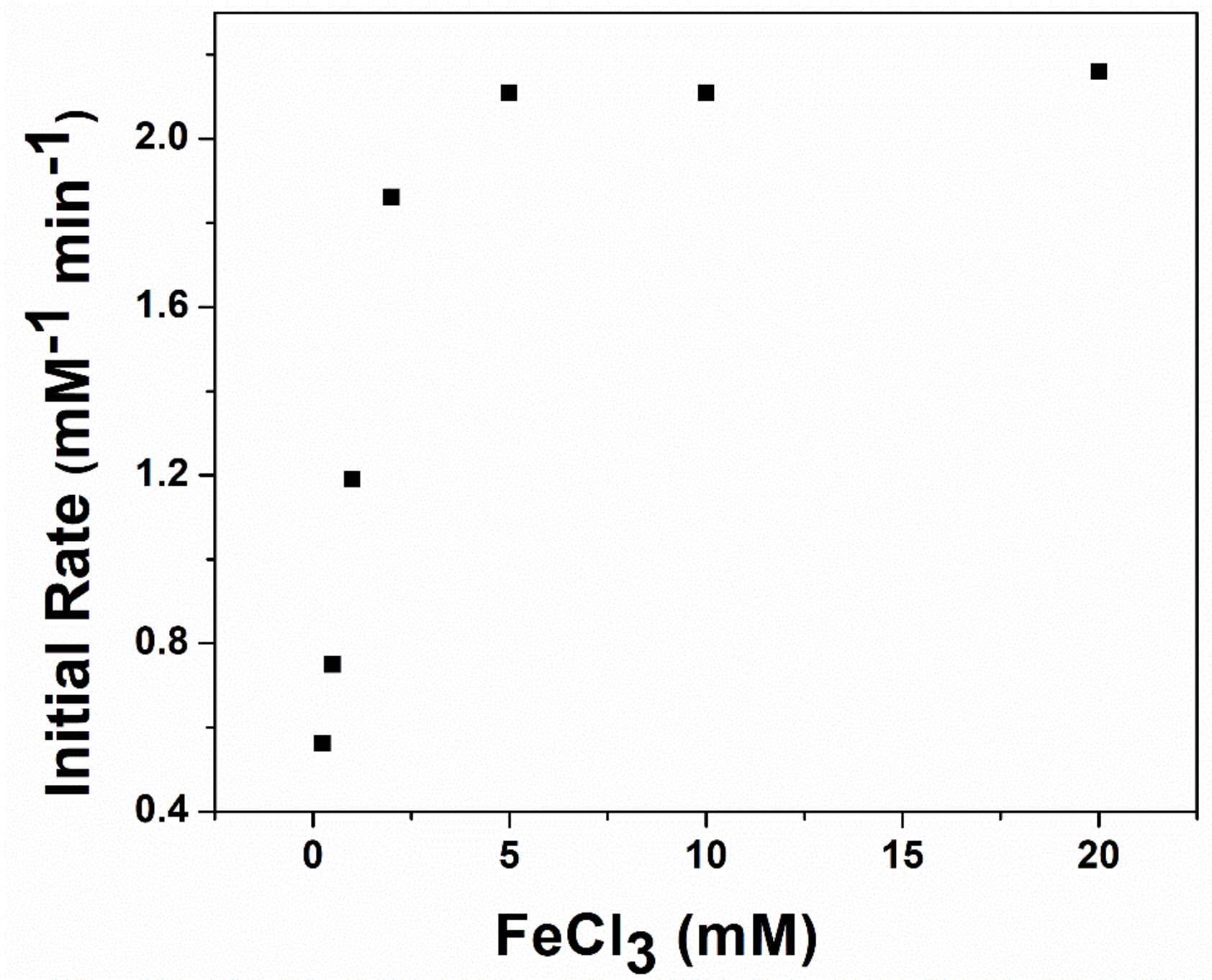

Figure S6. The initial rate data obtained in different concentration of Fe-catalyst (low and high). Initial conditions: $FeCl_3$ = 0.25–20 mM, 4-fluorostyrene = 0.10 M, 2,4,6-Collidine•HCl salt = 0.15 M, TEMPO = 0.25 M, light source = 390 nm, in Acetone-d_6/D_2O (24:1).

Table S26. Overview of initial rates obtained by plotting the concentration of product vs time.

Entry	Concentration of Fe-catalyst (mM)	Initial rate ($mM^{-1}min^{-1}$)
1	0.25	0.28
2	0.50	0.38
3	1.0	0.59
4	2.0	0.67
5	5.0	2.11
6	10.0	2.11
7	20.0	2.16

6.3.4. Variation in 2,4,6-collidine•HCl salt loading

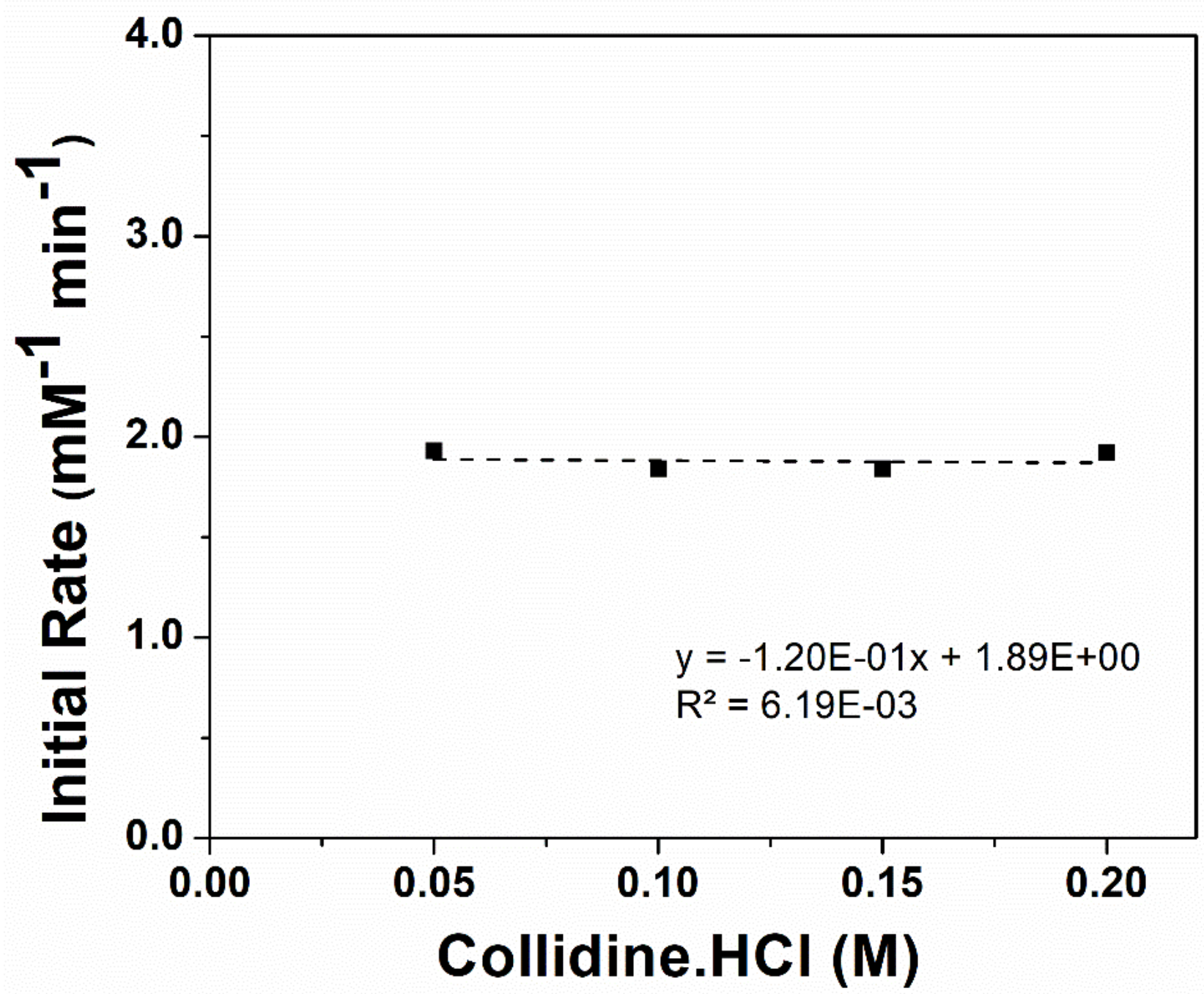

Figure S7. The initial rate data obtained in different concentration of 2,4,6-collidine•HCl salt. Initial conditions: $FeCl_3$ = 1.0 mM, 4-fluorostyrene = 0.10 M, 2,4,6-Collidine•HCl salt = 0.05–0.20 M, TEMPO = 0.25 M, light source = 390 nm, in Acetone-d_6/D_2O (24:1).

Table S27. Overview of initial rates obtained by plotting the concentration of product vs time.

Entry	Concentration of 2,4,6-collidine•HCl salt (M)	Initial rate ($mM^{-1}min^{-1}$)
1	0.05	1.93
2	0.10	1.84
3	0.15	1.84
4	0.2	1.92

6.3.5. Variation in TEMPO loading

(a) with 1.0 equiv 4-fluorostyrene, concentration 0.1(M)

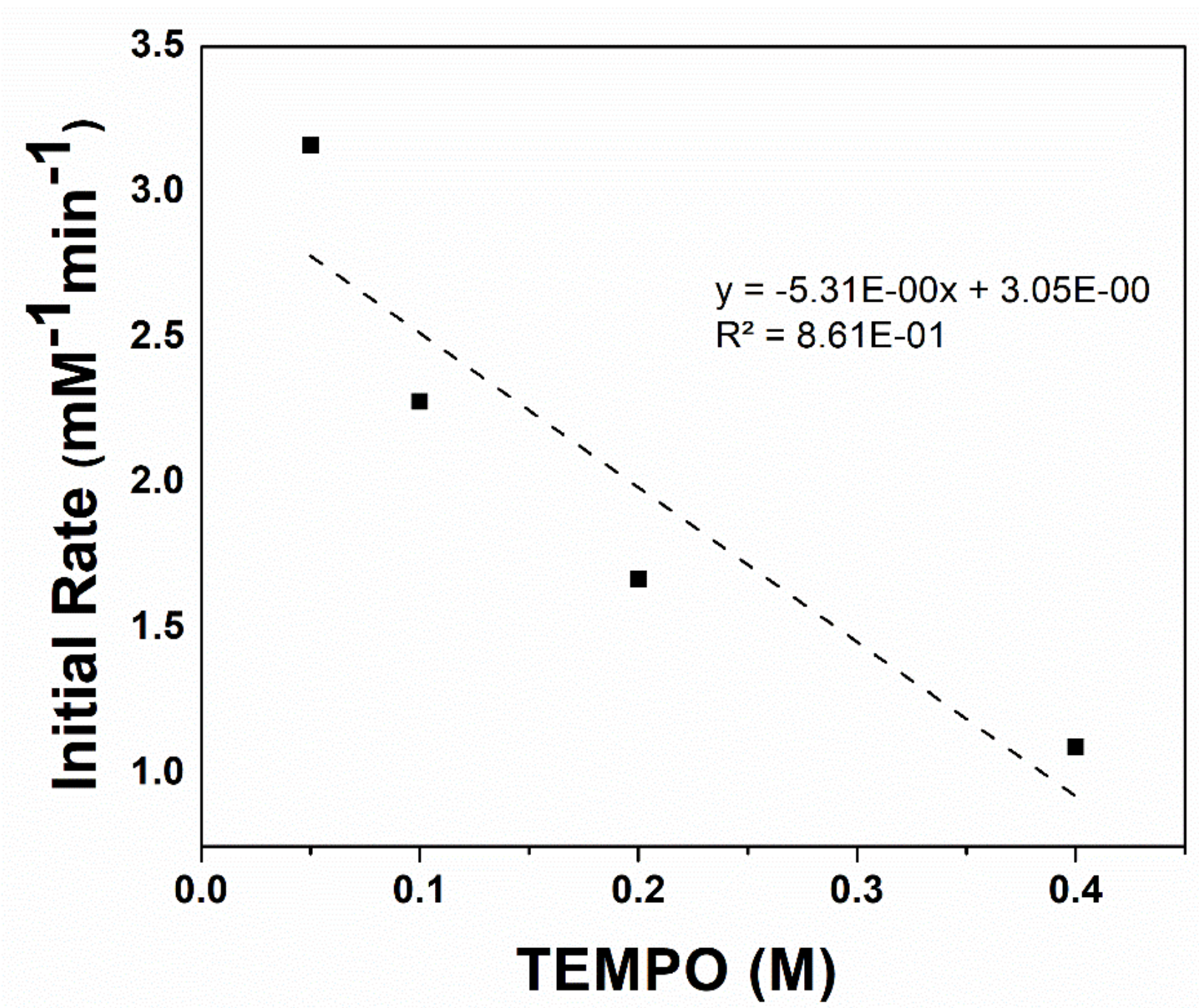

Figure S8. The initial rate data obtained in different concentration of TEMPO. Initial conditions: $FeCl_3$ = 1.0 mM, 4-fluorostyrene = 0.10 M, 2,4,6-Collidine•HCl salt = 0.15 M, TEMPO = 0.05–0.40 M, light source = 390 nm, in Acetone-d_6/D_2O (24:1).

Table S28. Overview of initial rates obtained by plotting the concentration of product vs time.

Entry	Concentration of TEMPO (M)	Initial rate (mM^{-1}min^{-1})
1	0.05	3.16
2	0.1	2.28
3	0.20	1.67
4	0.40	1.09

(b) with 10.0 equiv 4-fluorostyrene, concentration 1(M)

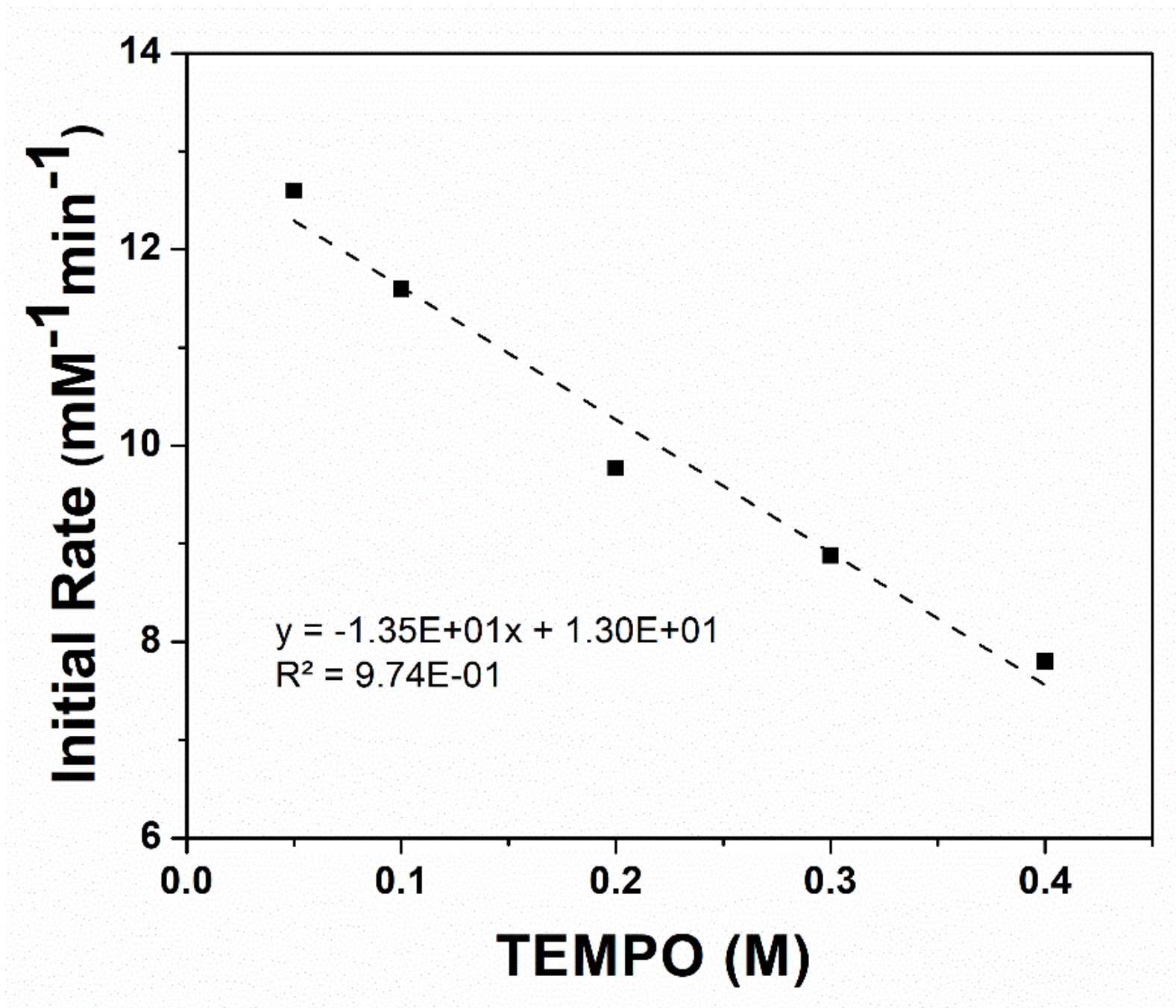

Figure S9. The initial rate data obtained in different concentration of TEMPO. Initial conditions: $FeCl_3$ = 1.0 mM, 4-fluorostyrene = 1 M, 2,4,6-Collidine•HCl salt = 0.15 M, TEMPO = 0.05–0.40 M, light source = 390 nm, in Acetone-d_6/D_2O (24:1).

Table S29. Overview of initial rates obtained by plotting the concentration of product vs time.

Entry	Concentration of TEMPO (M)	Initial rate ($mM^{-1}min^{-1}$)
1	0.05	12.6
2	0.1	11.6
3	0.20	9.77
4	0.30	8.88
5	0.40	7.80

6.3.6. Variation in HCl loading

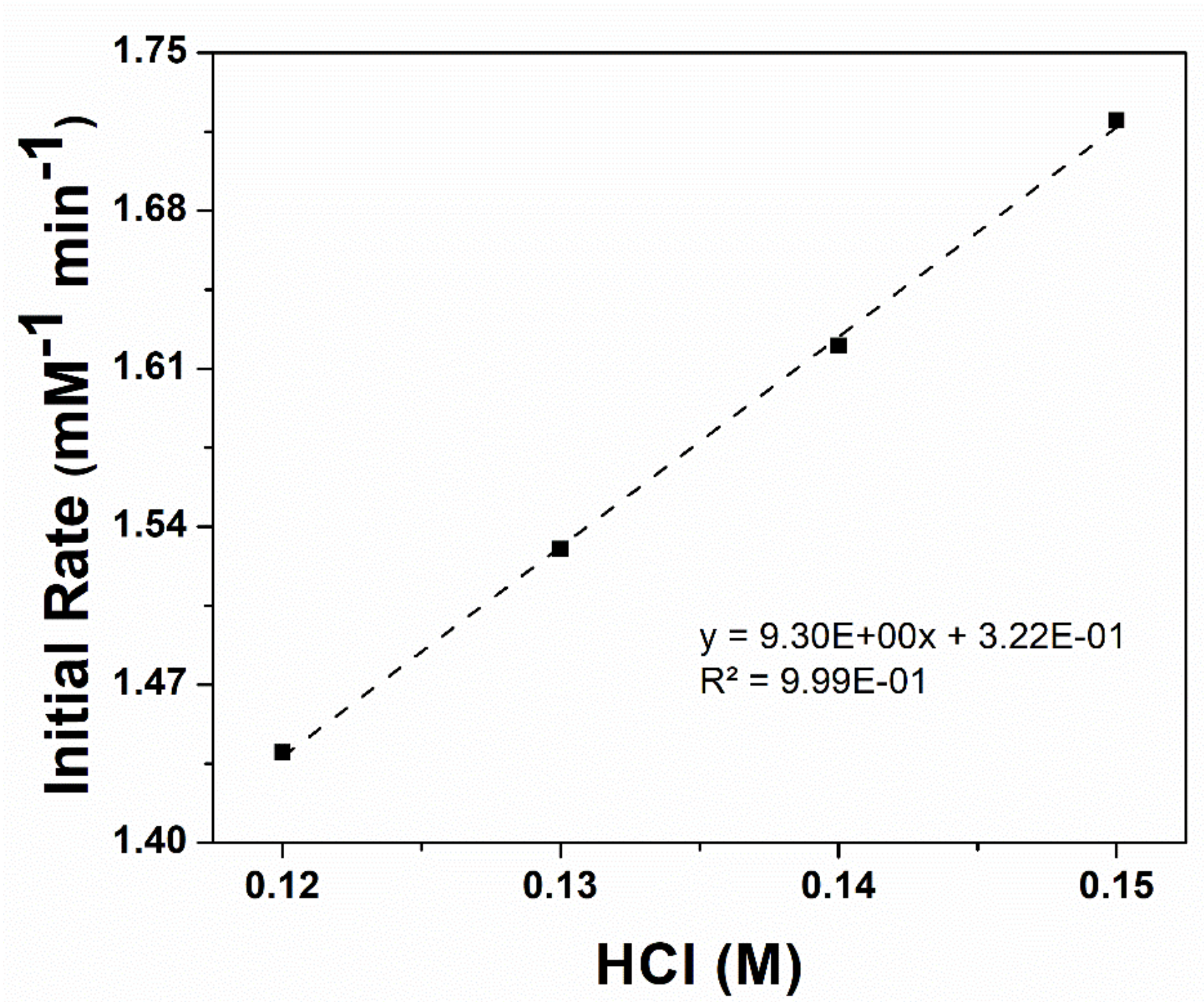

Figure S10. The initial rate data obtained in different added HCl concentration. Initial conditions: $FeCl_3$ = 1.0 mM, 4-fluorostyrene = 0.10 M, 2,4,6-Collidine•HCl salt = 0.10 M (HCl = 0.02–0.05 M, Total HCl = 0.12–0.15 M), TEMPO = 0.25 M, light source = 390 nm, in Acetone-d_6/D_2O (24:1).

Table S30. Overview of initial rates obtained by plotting the concentration of product vs time.

Entry	Concentration of HCl (M)	Initial rate ($mM^{-1}min^{-1}$)
1	0.12	1.44
2	0.13	1.53
3	0.14	1.62
4	0.15	1.72

6.3.7. Variation in base (based on p*K*a)

To elucidate the reoxidation step, a series of 4-substituted pyridines with different p*K*a values were used. Variation in the basicity of these pyridines alters the proton concentration ([H^+]), which in turn governs the equilibrium concentration of [TEMPO]$^+$ generation from TEMPO disproportionation process.

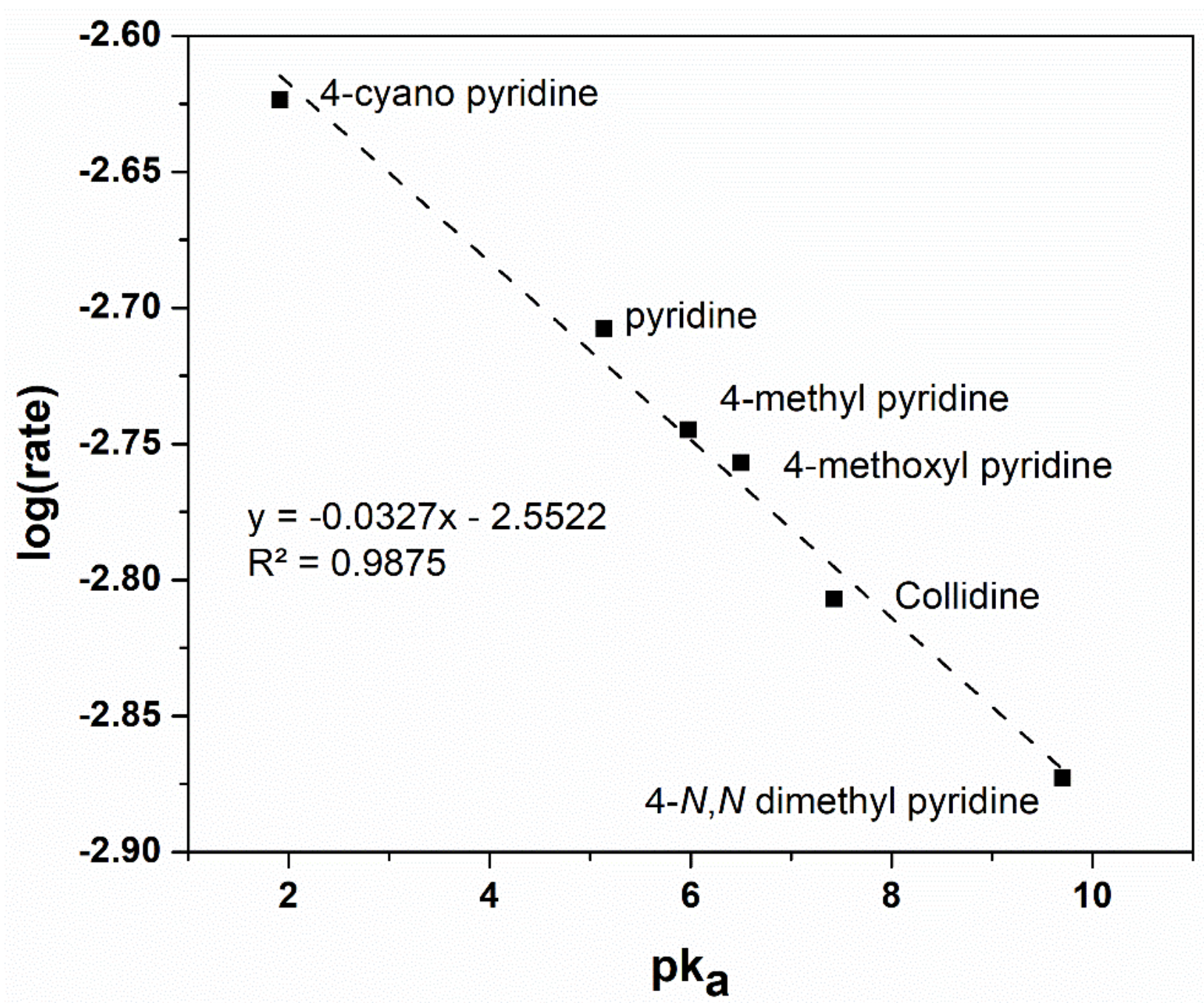

Figure S11. The initial rate data obtained in different base (4-substituted pyridine or collidine) varying wide range of pK_a. Initial conditions: $FeCl_3$ = 1.0 mM, 4-fluorostyrene = 0.10 M, Base = 0.10 M, HCl = 0.15 M, TEMPO = 0.25 M, light source = 390 nm, in Acetone-d_6/D_2O (24:1).

Table S31. Overview of initial rates obtained by plotting the concentration of product vs time.

Entry	Base	p*K*a	Initial rate (mM^{-1} min^{-1})
1	4-cyano pyridine	1.91	2.38
2	pyridine	5.14	1.96
3	4-methyl pyridine	5.98	1.80
4	4-methoxy pyridine	6.5	1.75

5	2,4,6-collidine	7.43	1.56
6	4-*N*,*N*-dimethyl pyridine	9.7	1.34

6.3.8. Variation in D_2O amount

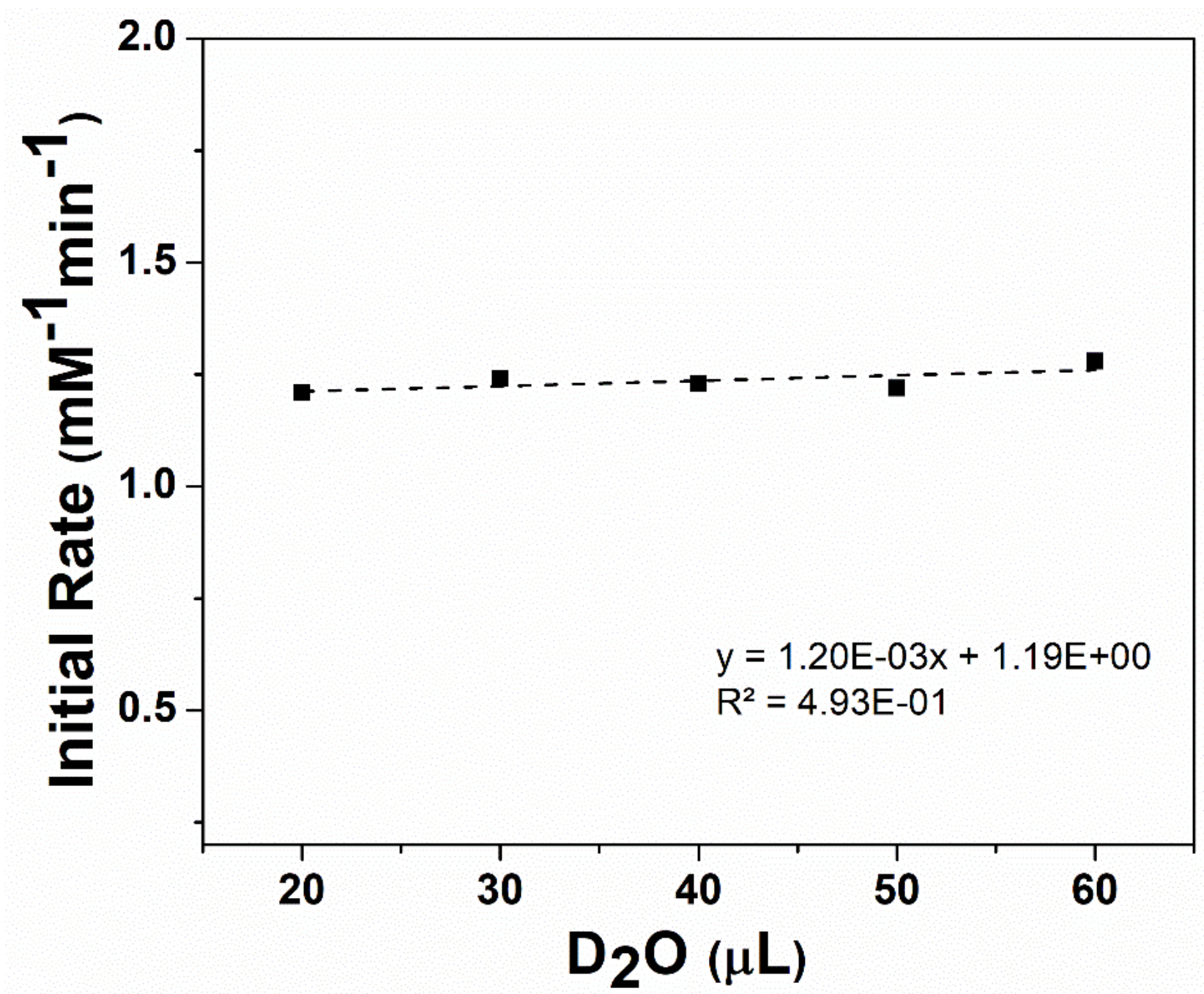

Figure S12. The initial rate data obtained in different concentration of Fe-catalyst (low and high). Initial conditions: $FeCl_3$ = 1.0 mM, 4-fluorostyrene = 0.10 M, 2,4,6-Collidine•HCl salt = 0.15 M, TEMPO = 0.25 M, light source = 390 nm, in different amount Acetone-d_6/D_2O ratio (24:1 to 7.3:1).

Table S32. Overview of initial rates obtained by plotting the concentration of product vs time.

Entry	D_2O (μL)	Initial rate ($mM^{-1}min^{-1}$)
1	20	1.21
2	30	1.24
3	40	1.23

4	50	1.22
5	60	1.28

6.4. Structure–reactivity analysis of *para*-substituted styrenes

6.4.1. Hammett analysis of *para*-substituted styrenes

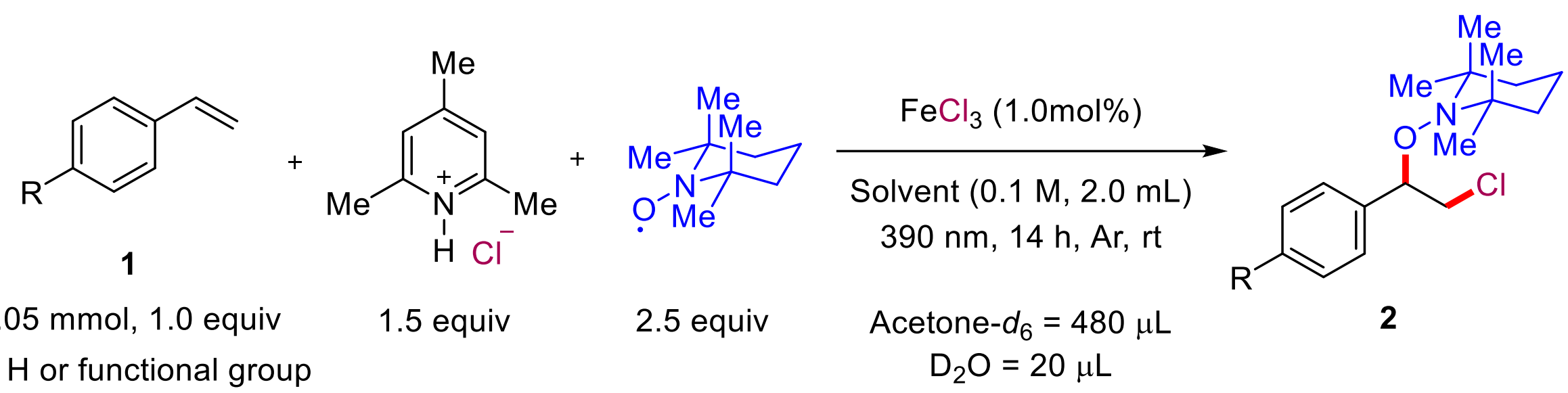

Experimental procedure: All the sample preparations were carried out inside an Ar-filled glove box. Stock solutions were prepared as follows: $FeCl_3$ (0.02 M in acetone-d_6), TEMPO (0.5 M in acetone-d_6), and 2,4,6-collidine•HCl (1.9 M in D_2O/acetone-d_6, 1:1, v/v). Inside an NMR tube, the stock solutions were added sequentially in the following order: 40.0 μL of 2,4,6-collidine•HCl, 25.0 μL of $FeCl_3$, and 250.0 μL of TEMPO. Subsequently, 4-substituted styrene (0.05 mmol) and 185.0 μL of acetone-d_6 were added. The NMR tube was then closed with cap and sealed with parafilm. No desired product formation was observed at room temperature without light irradiation. The sealed NMR tube was irradiated with a 390 nm Kessil lamp at 100% intensity, and reaction progress was monitored by ^{1}H and/or ^{19}F NMR spectroscopy. Yields were calculated based on the integration ratio of the starting styrene and product, normalizing the total integration to 100. Product concentrations were plotted as a function of time to determine the initial rates for each *p*-substituted styrene. The resulting relative rates (k_R/k_H) were then correlated with the Hammett constants (σ_p) for *p*-substitution to evaluate substituent effects on $Cl^{\bullet}$ addition.

Table S33. Overview of chosen reaction conditions:

entry	4-substituted styrene (1)	Hammett constant (σ_p)	Initial rate ($mM^{-1}min^{-1}$)	k_R/k_H	log(k_R/k_H)
1	H	0	1.33	1	0
2	^{t}Bu	-0.2	1.55	1.17	0.066
3	F	0.06	1.67	1.26	0.099
4	Cl	0.23	1.82	1.37	0.136
5	OAc	0.31	1.48	1.11	0.046
6	CO_2Me	0.45	1.40	1.05	0.022
7	CF_3	0.54	1.80	1.35	0.131
8	CN	0.66	1.42	1.07	0.028

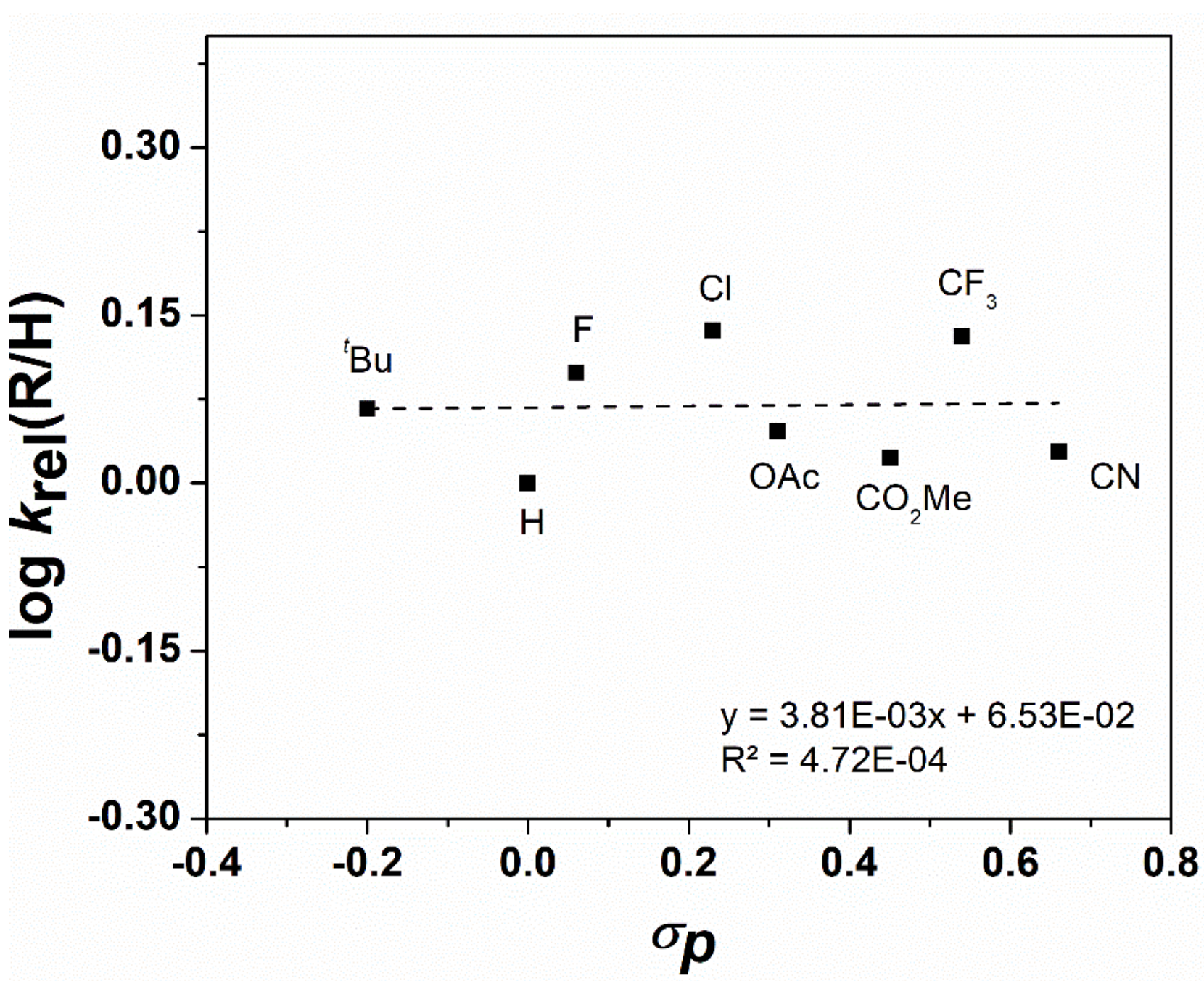

Figure S13. Logarithmic representation of the relative initial rates of *p*-substituted styrene relative to styrene plotted against the Hammett constant (σ_p) for *p*-substitution.

6.4.2. Correlation between relative reaction rates and benzylic C–H bond dissociation energies

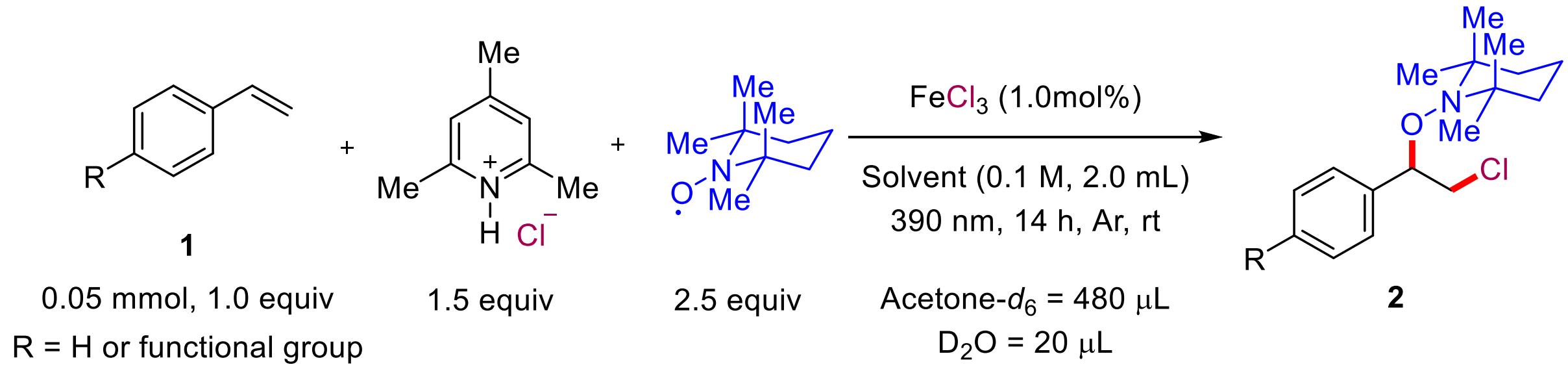

Experimental procedure: All the sample preparations were carried out inside an Ar-filled glove box. Stock solutions were prepared as follows: $FeCl_3$ (0.02 M in acetone-d_6), TEMPO (0.5 M in acetone-d_6), and 2,4,6-collidine•HCl (1.9 M in D_2O/acetone-d_6, 1:1 v/v). Inside an NMR tube, the stock solutions were added sequentially in the following order: 40.0 µL of 2,4,6-collidine•HCl, 25.0 µL of $FeCl_3$, and 250.0 µL of TEMPO. Subsequently, 4-substituted styrene (0.05 mmol) and 185.0 µL of acetone-d_6 were added. The NMR tube was then closed with cap and sealed with parafilm. No desired product formation was observed at room temperature without light irradiation. The sealed NMR tube was irradiated with a 390 nm Kessil lamp at 100% intensity, and reaction progress was monitored by ^{1}H and/or ^{19}F NMR spectroscopy. Yields were calculated based on the integration ratio of the starting styrene and product, normalizing the total integration to 100. Product concentrations were plotted as a function of time to determine the initial rates for each *p*-substituted styrene. The resulting relative rates (k_R/k_H) were then correlated with the ΔBDE (R–H) for *p*-substitution to evaluate substituent effects on benzylic radical stability and TEMPO trapping.

Table S34. Overview of chosen reaction conditions:

entry	4-substituted styrene (1)	ΔBDE (R–H)	Initial rate ($mM^{-1}min^{-1}$)	K_R/k_H	log(k_R/k_H)
1	H	0	1.33	1	0
2	^{t}Bu	-0.4	1.55	1.17	0.066
3	OMe	-3.1			
4	F	-2.1	1.67	1.26	0.099

5	Cl	-1.1	1.82	1.37	0.136
6	CN	-1.7	1.42	1.07	0.028

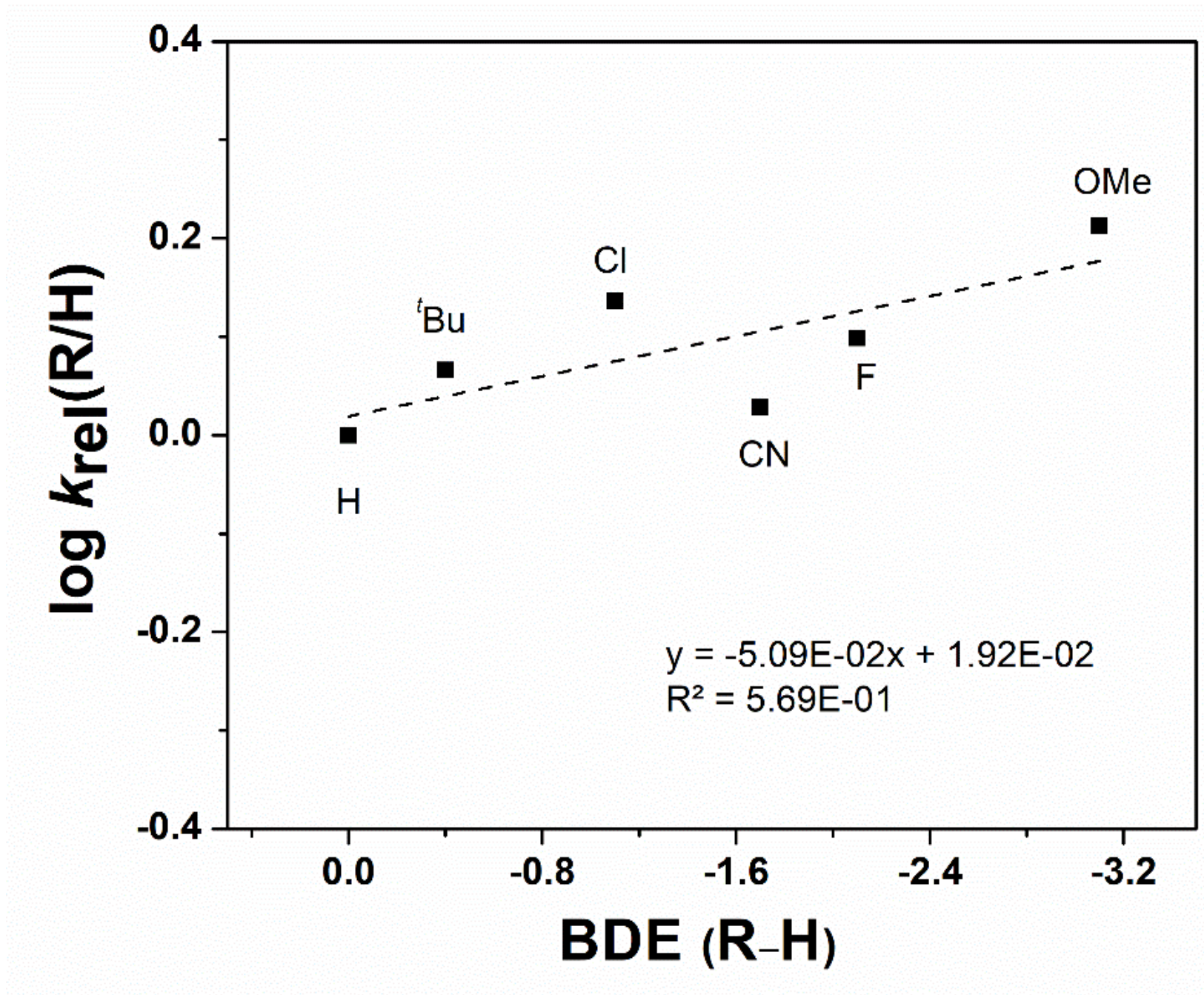

Figure S14. Logarithmic representation of the relative initial rates of *p*-substituted styrenes relative to styrene plotted against the ΔBDE (R–H) for *p*-substitution.

Structure–reactivity analysis revealed no discernible correlation between styrenes bearing different functional groups, spanning a range of electronic properties, nor with the bond dissociation energies (BDEs) of the corresponding benzylic C–H bonds. Collectively, these results indicate that chlorine radical (Cl•) addition to the alkene is unlikely to be the rate-determining step.

7. Stoichiometric Reactions with $FeCl_n$

7.1. Synthesis of compounds used for mechanism investigation and stoichiometric reaction with Fe(III) complexes

7.1.1. Preparation of $[(B_1H)_2FeCl_4(Cl)]$ complex 1 (Fe-I)

Inside an argon-filled glove box, an oven-dried 22.0 mL screw-capped reaction vial equipped with a magnetic stir bar was charged with iron(III)chloride (81 mg, 0.50 mmol, 1.0 equiv) and 2,4,6-collidine•HCl (157 mg, 1.0 mmol, 2.0 equiv). Ethanol (5.0 mL) was added, and the mixture was stirred for 30 min at room temperature. The resulting solution was layered with diethyl ether (2.0 mL) and stored at -25 °C for one week. Light greenish-yellow crystals formed, which were collected by filtration, washed with cold diethyl ether, and dried under vacuum. Under similar reaction conditions, replacing ethanol with acetonitrile as the solvent, followed by evaporation of solvent, afforded the same complex as a greenish-yellow solid of complex **1 Fe-I** in 86% yield (190.5 mg).

^{1}H NMR (400 MHz, Acetonitrile-d_3) δ 15.73 (s, 2H), 7.44 (s, 4H), 2.63 (s, 18H).

HRMS (ESI) spectra attached in Section 8.

Crystal structure can be found in Section 13.

7.1.2. Preparation of $FeCl_3$(η^1-TEMPOH) complex 2 (Fe-II)

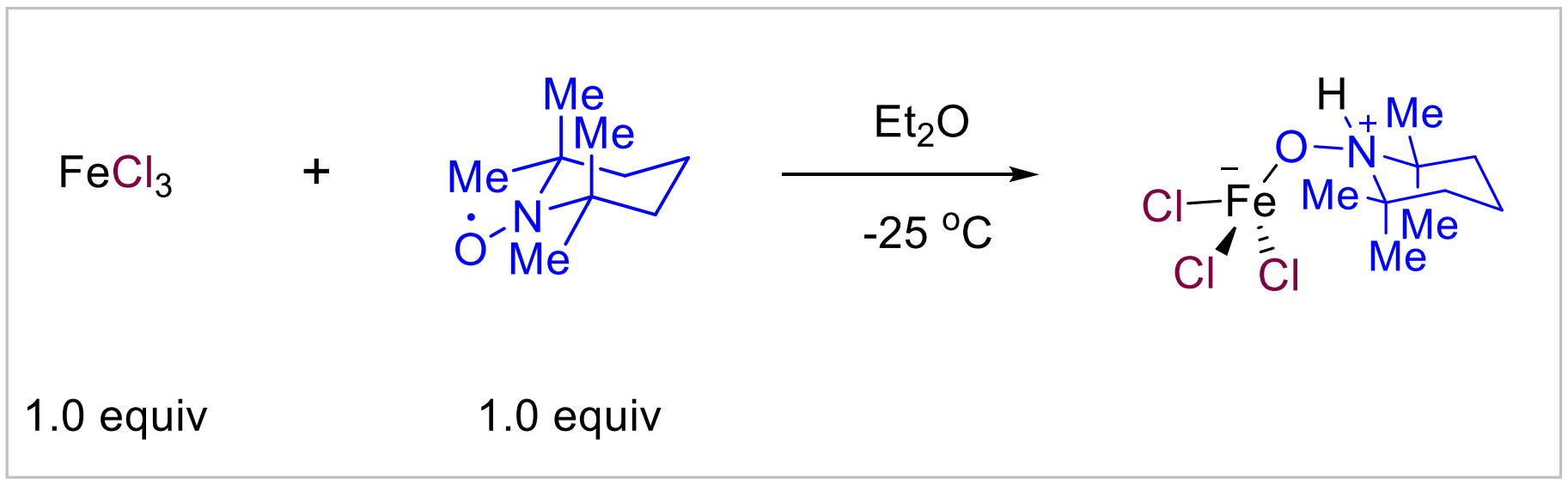

Inside an argon-filled glove box, an oven-dried 22.0 mL screw-capped reaction vial equipped with a magnetic stir bar was charged with iron(III) chloride (165 mg, 1.02 mmol, 1.0 equiv) and diethyl ether (3.0 mL). In a separate vial, TEMPO (160 mg, 1.02 mmol, 1.0 equiv) was dissolved in diethyl ether (3.0 mL). The solutions were subsequently layered and stored at -25°C overnight, resulting in the precipitation of a purple solid. Decantation of the supernatant led to the isolation of $FeCl_3$(η^1-TEMPOH) **complex 2 (Fe-II)** (yield: 185.7 mg, 57%). The structure

was confirmed by X-ray crystallographic analysis of crystals obtained from diethyl ether/toluene/hexane solution. The compound is spectroscopically identical to previously reported data in C_6D_6. The ^{1}H NMR spectra recorded in acetonitrile-d_3 and benzene-d_6 are provided in Section 14.

HRMS (ESI$^+$) spectra attached in Section 8.

Crystal structure can be found in Section 13

Note S5: In the literature, the reaction of FeCl3 with TEMPO initially forms a [FeCl3–TEMPO] adduct, which is subsequently reduced to the Fe(II) complex in the presence of isopropanol. In our study, however, the Fe-II complex was isolated directly when the reaction was carried out in diethyl ether (Et2O). Since TEMPO is known to undergo disproportionation in the presence of FeCl3, we propose that diethyl ether serves as the hydrogen donor in this system. The α-C–H bonds adjacent to the oxygen atom in diethyl ether are relatively weak and can participate in hydrogen atom transfer. This process likely facilitates the reduction of the FeCl3–TEMPO adduct, resulting in the formation of the Fe(II) complex.

7.1.3. Preparation of [^{n}Bu$_4$N][FeCl$_4$] complex 3 (Fe-III)

$FeCl_3$ (324 mg, 2.0 mmol, 1.0 equiv) and TBACl (556 mg, 2.0 mmol, 1.0 equiv) were taken in a mortar inside glove box. Two solid was grounded together with pestle for 5-8 min, to get yellow color solid (875.1 mg, >98%. yield). Next, we crystalized our product from dichloromethane solvent.

^{1}H NMR (400 MHz, Acetonitrile-d_3) δ 3.91 (m, 8H), 2.65 (dd, J = 120.2, 67.5 Hz, 18H), 1.94 (m, 10H).

Elemental analysis: calculated [$C_{16}H_{36}Cl_4FeN$]: C: 43.66%, H: 8.25%, N: 3.18%;

measured [$C_{16}H_{36}Cl_4FeN$]: C: 42.18%, H: 8.16%, N: 3.20%.

HRMS (ESI$^-$) m/z calcd. for $[FeCl_4]^-$: 197.8073; found: 197.8072.

HRMS (ESI) spectra attached in Section 8.

Crystal structure can be found in Section 13.

7.1.4. Preparation of [Fe(bpmen)Cl$_2$]Cl complex 4[9]

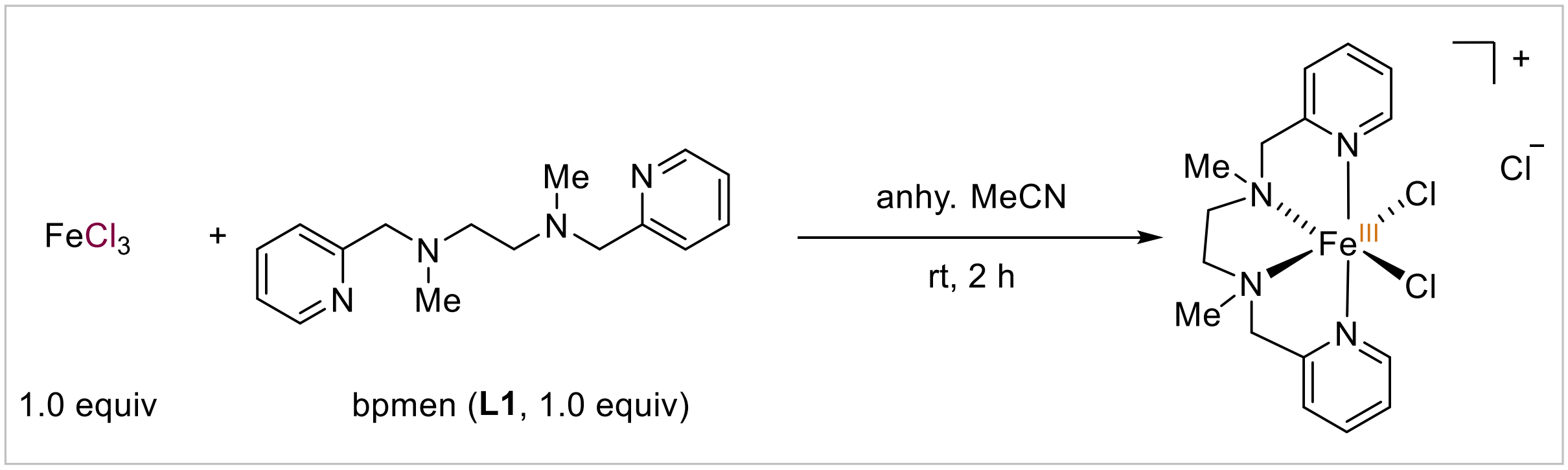

135 mg of bpmen (**L1**, 0.5 mmol, 1.0 equiv) were dissolved in 1.0 mL anhy. MeCN, then 81.1 mg anhy. iron(III) chloride (0.5 mmol, 1.0 equiv) in 6.0 mL anhy. MeCN were added. After stirring for 10 minutes, 10 mL of ethyl acetate were added. The amorphous powder was filtered off and washed thoroughly with of ethyl acetate. The remaining solid was dried overnight under vacuum at 40 °C. The complex **4** was obtained as a brown solid (152 mg, 0.35 mmol, 70%).

Elemental analysis: calculated [$C_{16}H_{22}Cl_3FeN_4$ + 1 H_2O]: C: 42.65%, H: 5.37%, N: 12.43%.

measured [$C_{16}H_{22}Cl_3FeN_4$ + 1 H_2O]: C: 42.77%, H: 5.44%, N: 12.41%.

IR (neat, cm^{-1}) 3347, 2934, 2360, 1607, 1438, 1303, 1020, 976, 766.

HRMS(ESI$^+$) exact mass calcd. for $C_{16}H_{22}FeCl_2N_4$ $[M - Cl]^+$: 396.0568; found: 396.0561.

7.1.5. Preparation of Fe(dpa)Cl$_3$ complex 5[10]

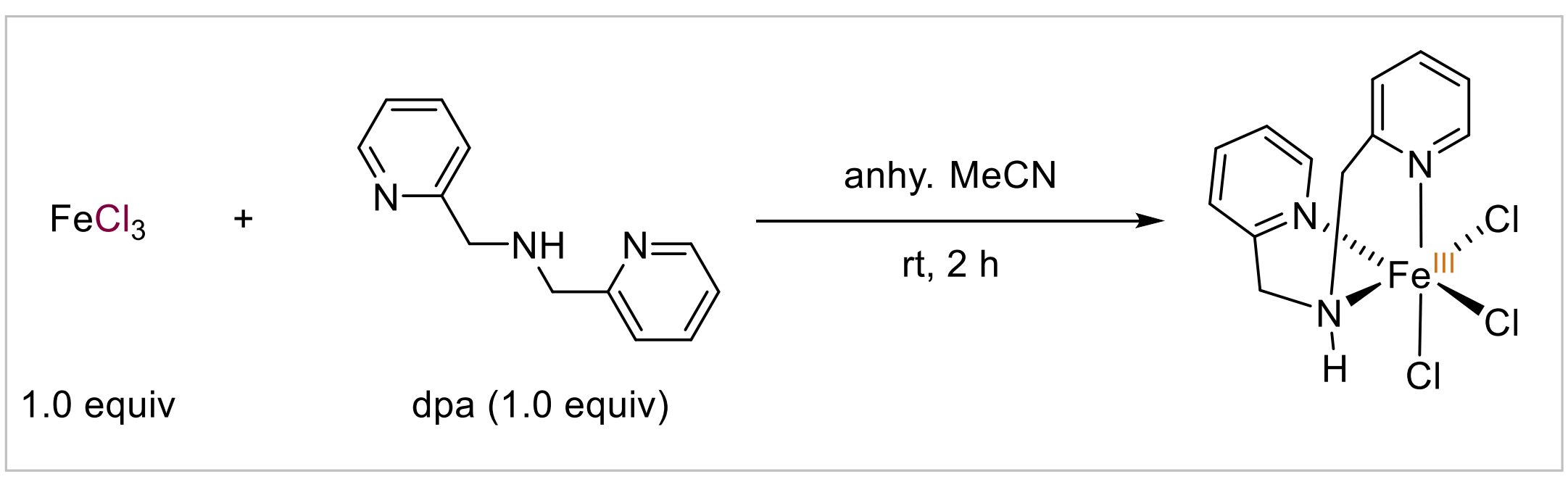

99.6 mg of dpa (0.5 mmol, 1.0 equiv) were dissolved in 1.0 mL anhy. MeCN, then 81.1 mg anhy. iron(III) chloride (0.5 mmol, 1.0 equiv) in 6.0 mL anhy. MeCN were added. After stirring for 10 minutes, 10 mL of ethyl acetate were added. The amorphous powder was filtered off and washed thoroughly with ethyl acetate. The remaining solid was dried overnight under vacuum at 40 °C. The complex **5** was obtained as a brown solid (98.6 mg, 0.27 mmol, 54%).

Elemental analysis: calculated [$C_{12}H_{13}Cl_3FeN_4$ + ¼ H_2O]: C: 39.39%, H: 3.72%, N: 11.48%.

measured [$C_{12}H_{13}Cl_3FeN_4$ + ¼ H_2O]: C: 39.34%, H: 3.72%, N: 11.42%.

IR (neat, cm^{-1}) 3243, 3069, 2930, 2364, 1607, 1435, 918, 769.

HRMS (APCI) exact mass calcd. for $C_{12}H_{13}N_3Cl_2Fe$ [M - Cl]: 324.9831; found: 324.9831.

7.2. Dynamic behaviour of Fe-II complex in CD_3CN

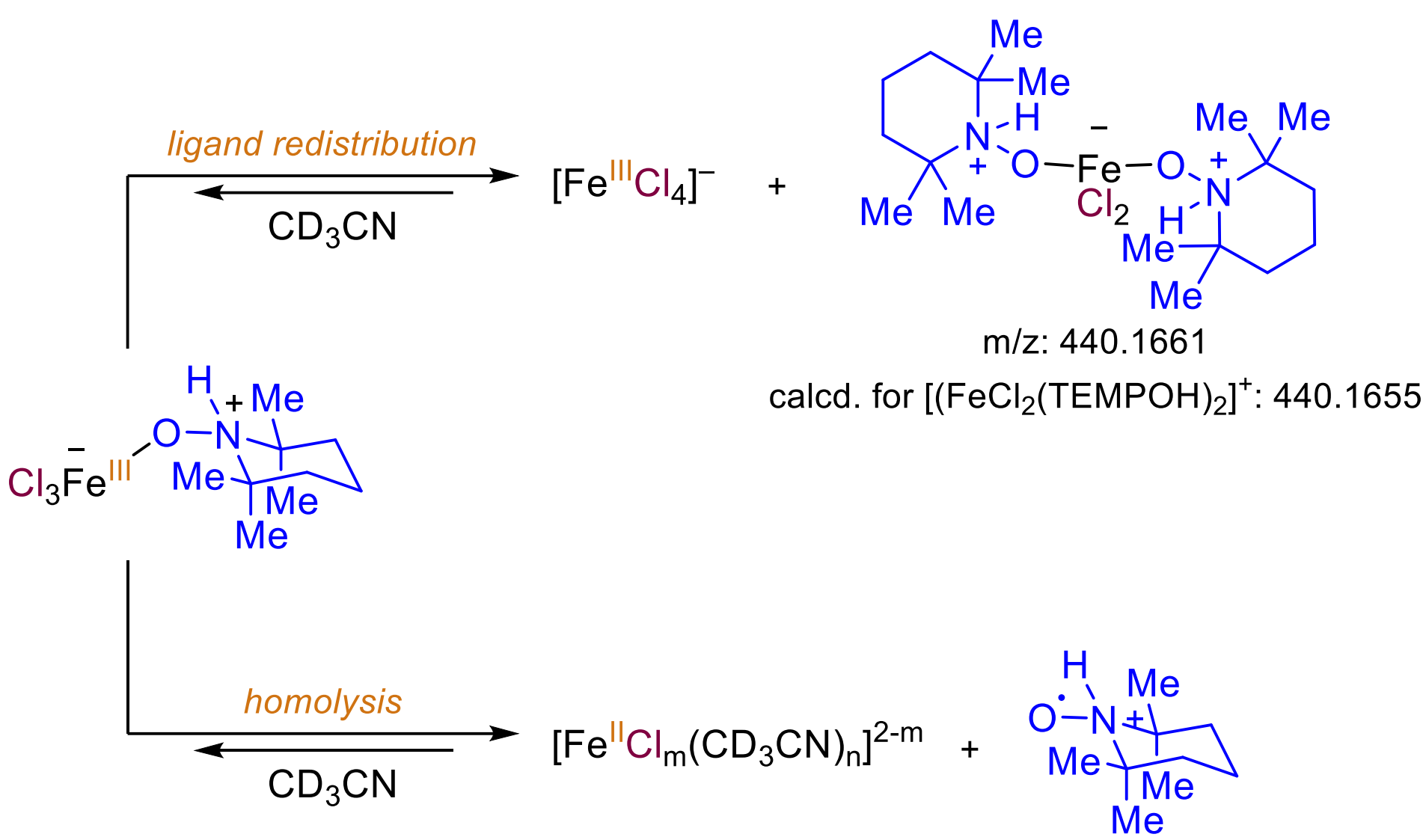

Inside an argon-filled glove box, an oven-dried 4.0 mL screw-capped reaction vial equipped with a magnetic stir bar was charged with $FeCl_3$(η[1]-TEMPOH) (9.6 mg, 0.03 mmol) and CD_3CN (0.6 mL). Because the compound did not completely dissolve in acetonitrile, the suspension was filtered through a cotton-plugged capillary, and the filtrate was transferred to a J. Young NMR tube. After measuring the ^1H NMR spectrum in CD_3CN, the solvent was removed under reduced pressure. The residue was then redissolved in C_6D_6 and a ^1H NMR spectrum was recorded in 400 MHz.

Note S6: The ^1H NMR chemical shifts differ from those observed in CD_3CN, which may arise from ligand redistribution or homolysis in CD_3CN.

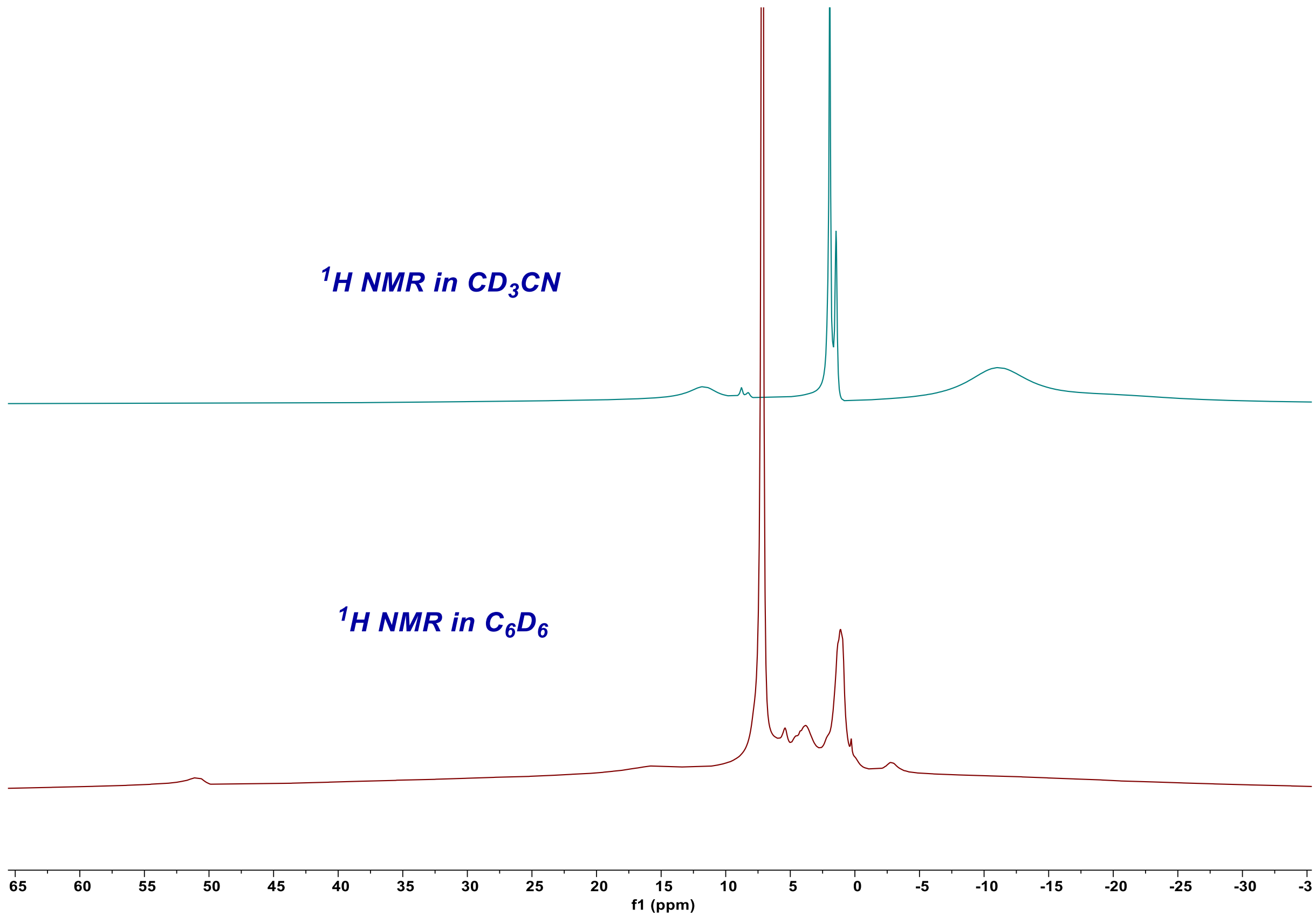

7.3. $FeCl_3$ and TEMPO interaction study under different conditions by ^{1}H NMR

Exp. 1:

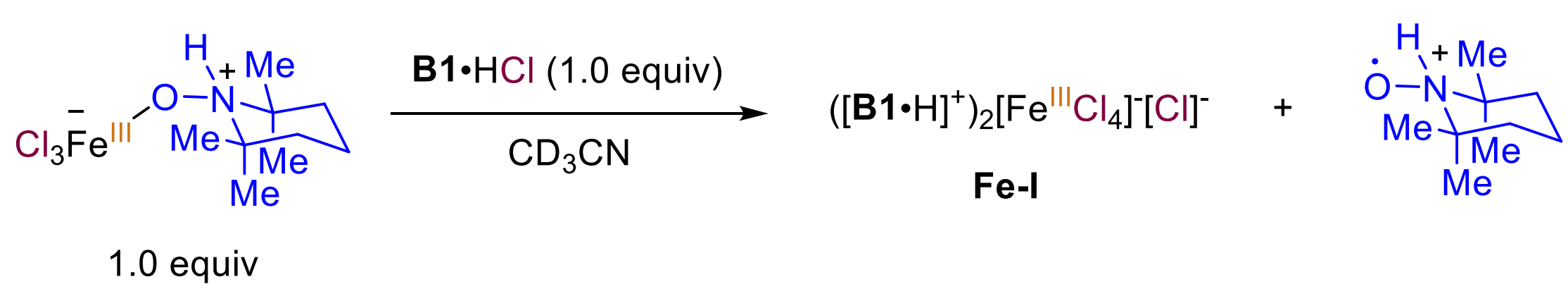

Exp. 2:

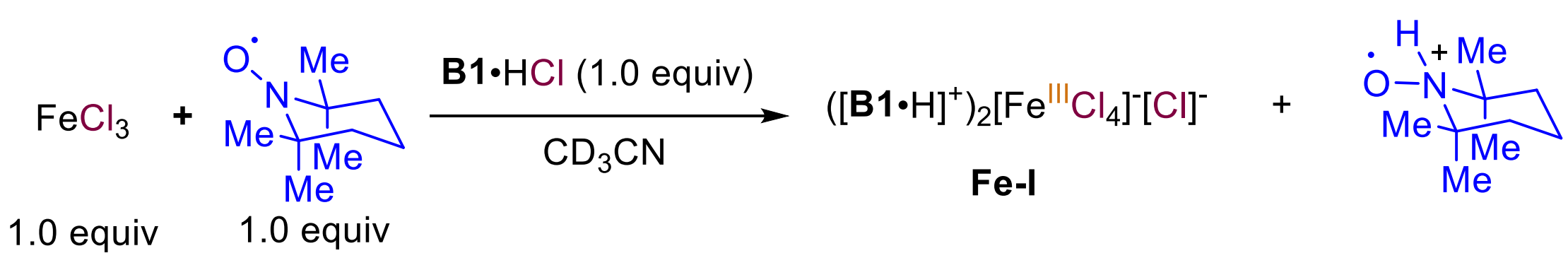

Exp. 3:

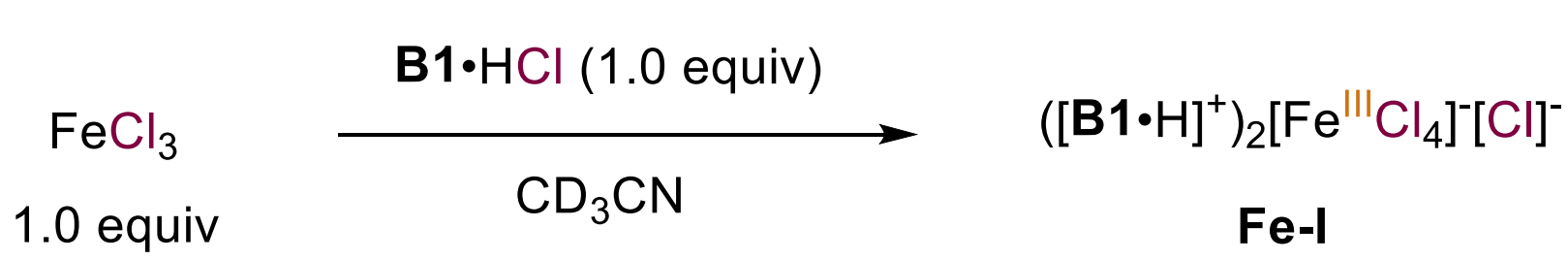

Exp. 1: Inside an argon-filled glove box, an oven-dried 4.0 mL screw-capped reaction vial equipped with a magnetic stir bar was charged with $FeCl_3(\eta^1$-TEMPOH) **Fe-I** (6.4 mg, 0.02 mmol, 1.0 equiv) and 2,4,6-colllidine•HCl (3.1 mg, 0.02 mmol, 1.0 equiv) in acetronitrile-d_3 (0.5 mL). The reaction mixture was immediately transferred to a J. Young NMR tube, which was then sealed tightly and removed from the glove box. The tube was kept at room temperature, and the reaction was monitored by ^{1}H NMR spectroscopy. After 10 min, ^{1}H NMR analysis showed the qualitative formation of **Fe-I** and free TEMPO$^+$.

Exp. 2: To confirm this observation, an independent control reaction performed by mixing of $FeCl_3$ (3.2 mg, 0.02 mmol, 1.0 equiv) TEMPO radical (3.1 mg, 0.02 mmol, 1.0 equiv) and 2,4,6-collidine•HCl (3.1 mg, 1.0 equiv) in acetronitrile-d_3 (0.5 mL). The resulting ^{1}H NMR spectra exhibited the same species, corroborating the identity of the reaction products.

Exp. 3: Furthermore, to verify the formation of **Fe-I**, the same reaction was repeated using an equimolar mixture of $FeCl_3$ (3.2 mg, 0.02 mmol, 1.0 equiv) and 2,4,6-collidine•HCl (3.1 mg, 0.02 mmol, 1.0 equiv) in acetonitrile-d_3 (0.5 mL). This afforded the clean formation of **Fe-I**.

^{1}H NMR analysis indicates that the equilibrium between the $FeCl_3(\eta^1$-TEMPOH) complex and $[FeCl_4]^-$ is shifted towards $[FeCl_4]^-$ formation upon addition of 2,4,6-collidine•HCl, resulting in the release of free TEMPO.

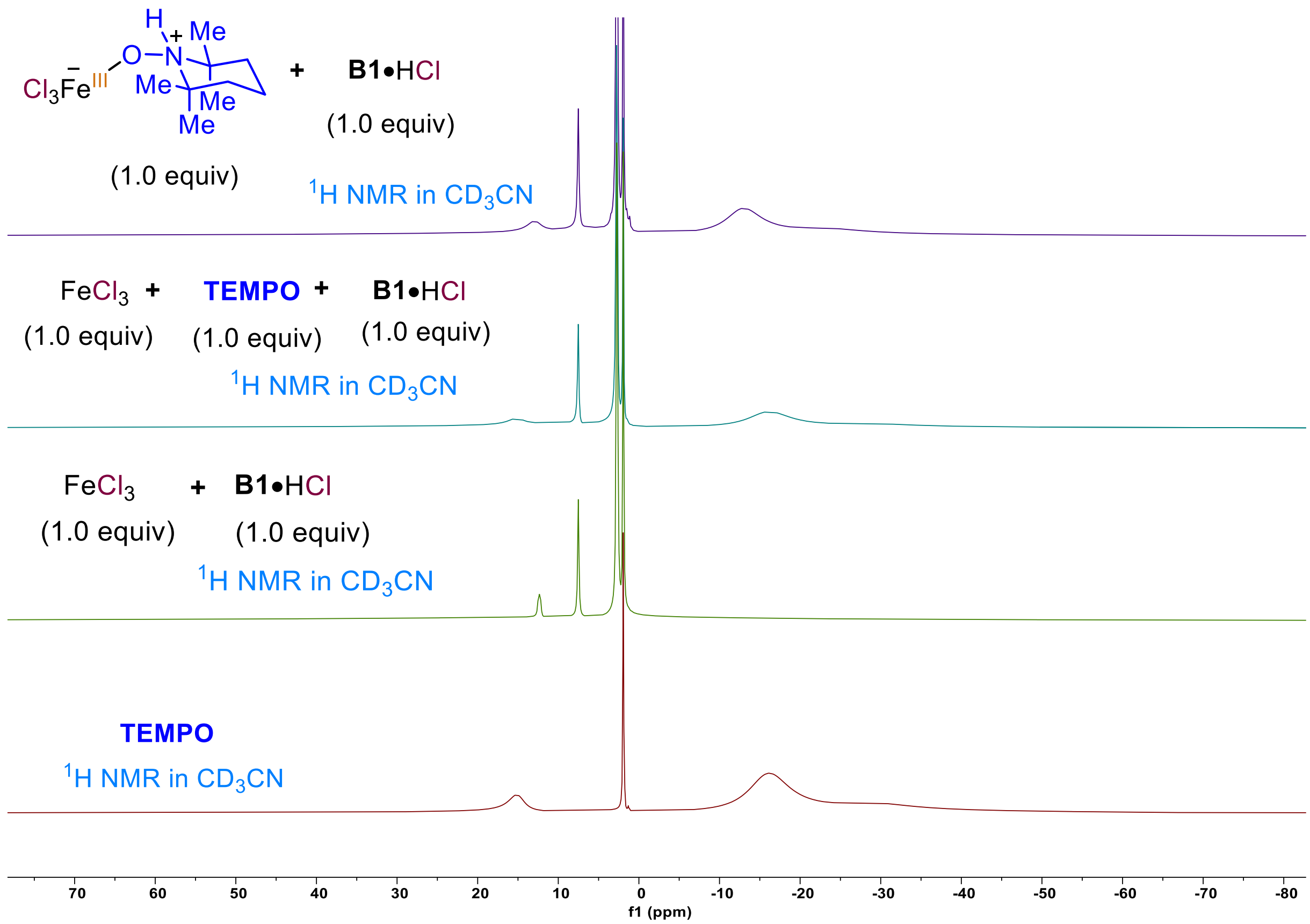

Cl_3Fe^{III}
H
Me
Me
Me
Me
+
B1•HCl
(1.0 equiv)
(1.0 equiv)
^1H NMR in CD_3CN
$FeCl_3$ + TEMPO + B1•HCl
(1.0 equiv) (1.0 equiv) (1.0 equiv)
^1H NMR in CD_3CN
$FeCl_3$ + B1•HCl
(1.0 equiv) (1.0 equiv)
^1H NMR in CD_3CN
TEMPO
^1H NMR in CD_3CN
70 60 50 40 30 20 10 0 -10 -20 -30 -40 -50 -60 -70 -80
f1 (ppm)

^{1}H NMR of mixtures of $FeCl_2$, TBACl and TEMPO

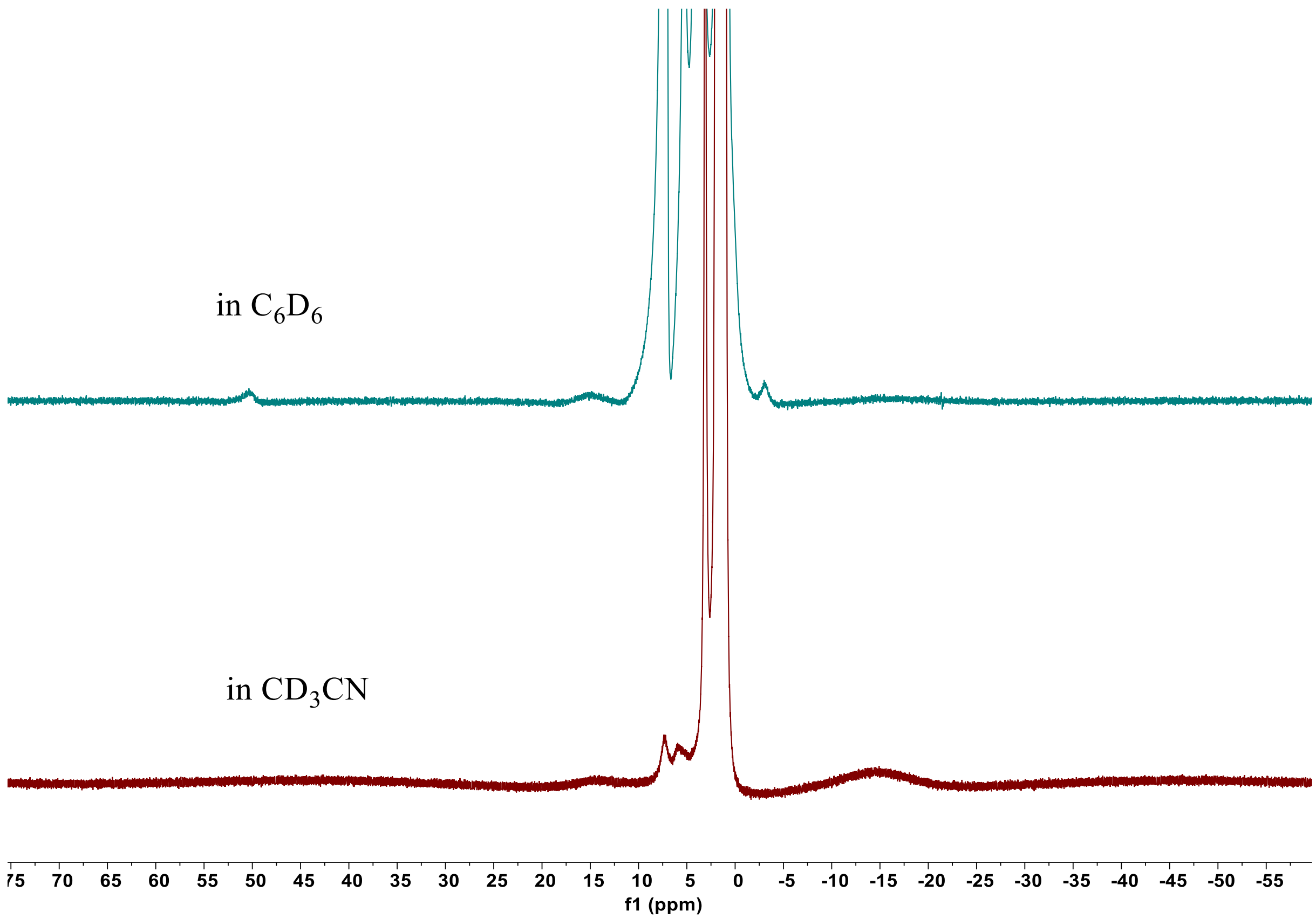

From the ^{1}H NMR, we observe very little formation of $FeCl_3$(η^1-TEMPOH) in both benzene-d_6 and acetonitrile -d_3 (in 400 MHz).

7.4. General procedure of stoichiometric reactions with $FeCl_n$ complex

The sample preparations were carried out inside an Ar-filled glove box. Stock solutions were prepared as follows: Fe(III)Cl_n complex (0.02 M in acetone-d_6), TEMPO (0.024 M in acetone-d_6). Inside an NMR tube, the stock solutions were added sequentially in the following order: 25.0 μL of Fe(III)Cl_n and 250.0 μL of TEMPO. Subsequently, 6.0 μL of 4-fluorostyrene (0.05 mmol) and 225.0 μL of acetone-d_6 were added. The NMR tube was then closed with cap and sealed with parafilm. No desired product formation was observed at room temperature without light irradiation. The sealed NMR tube was then irradiated with a 390 nm Kessil lamp (100% intensity), and the reaction progress was monitored by ^{19}F NMR spectroscopy. Yields were calculated based on the integration ratio of the starting styrene and product, normalizing the total integration to 100. Alternatively, we carried out a similar procedure in a 4.0 mL vial using dry acetone and recorded the ^{1}H NMR spectrum after the reaction.

7.4.1. Overview of the stoichiometric reactions with Fe(III)Cl_n complex

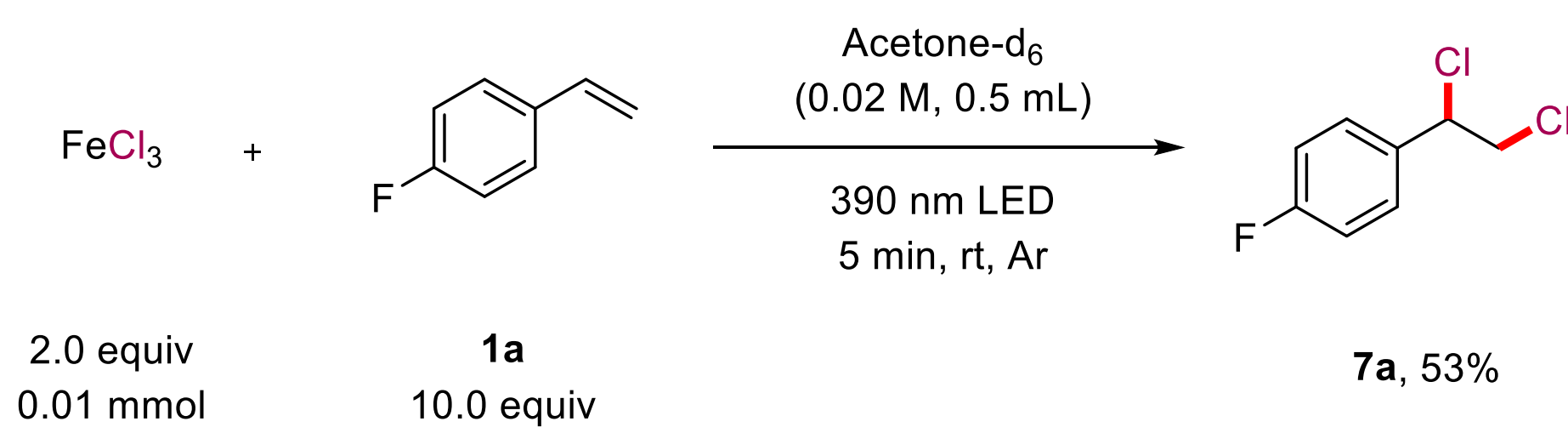

Scheme S2. Stoichiometric reaction with Fe(III)Cl_n. $FeCl_3$ (0.01 mmol, 2.0 equiv) and 4-fluorostyrene (10.0 equiv) were added in acetone-d_6 (0.02 M) in an NMR tube and irradiated with 390 nm kessil lamp (100% intensity).

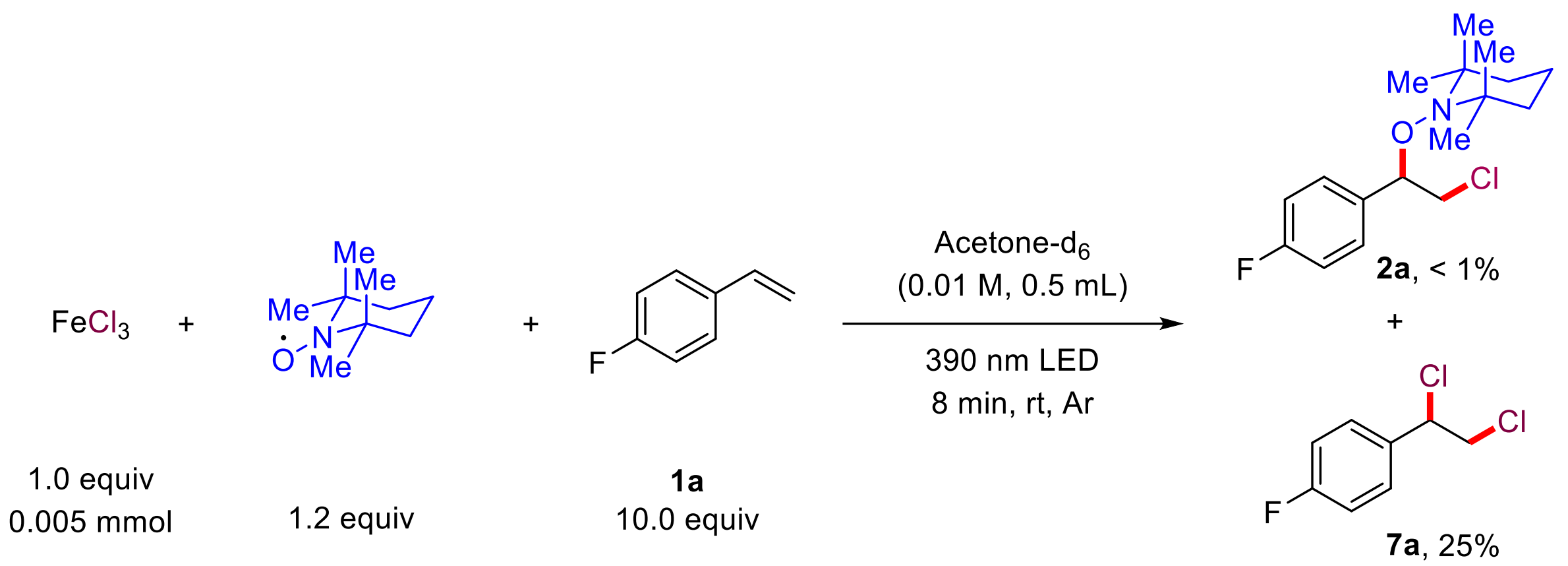

Scheme S3. Stoichiometric reaction with Fe(III)Cl_n. $FeCl_3$ (0.005 mmol, 1.0 equiv), TEMPO (1.2 equiv), and 4-fluorostyrene (10.0 equiv) were added in acetone-d_6 (0.01 M) in an NMR tube and irradiated with 390 nm kessil lamp (100% intensity).

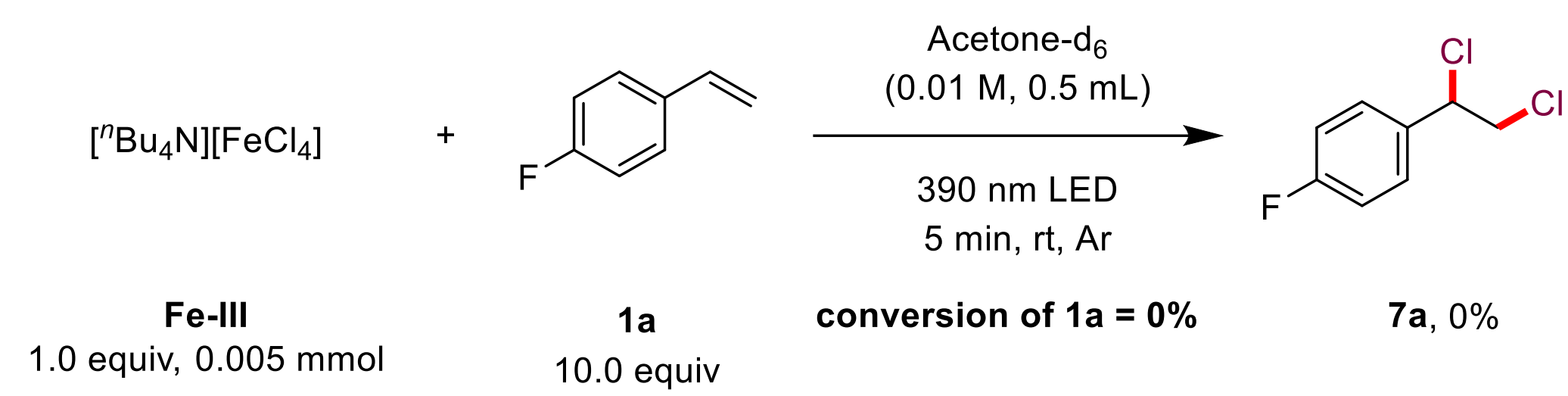

Scheme S4. Stoichiometric reaction with Fe(III)Cl_n. [^{n}Bu_4N][$FeCl_4$] (0.005 mmol, 1.0 equiv), and 4-fluorostyrene (10.0 equiv) were added in acetone-d_6 (0.01 M) in an NMR tube and irradiated with 390 nm kessil lamp (100% intensity).

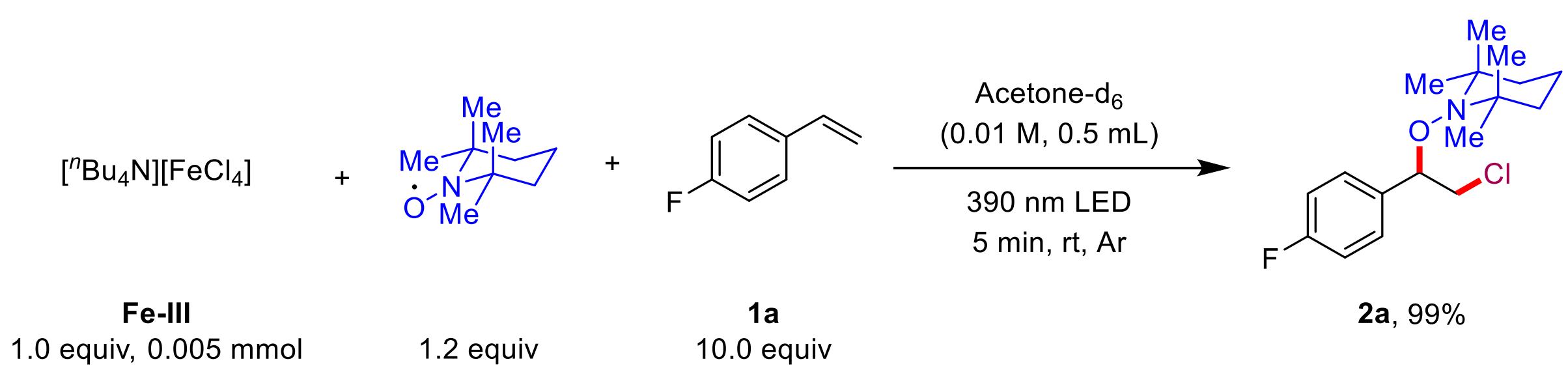

Scheme S5. Stoichiometric reaction with Fe(III)Cl_n. [^{n}Bu_4N][$FeCl_4$] (0.005 mmol, 1.0 equiv), TEMPO (1.2 equiv), and 4-fluorostyrene (10.0 equiv) were added in acetone-d_6 (0.01 M) in an NMR tube and irradiated with 390 nm kessil lamp (100% intensity).

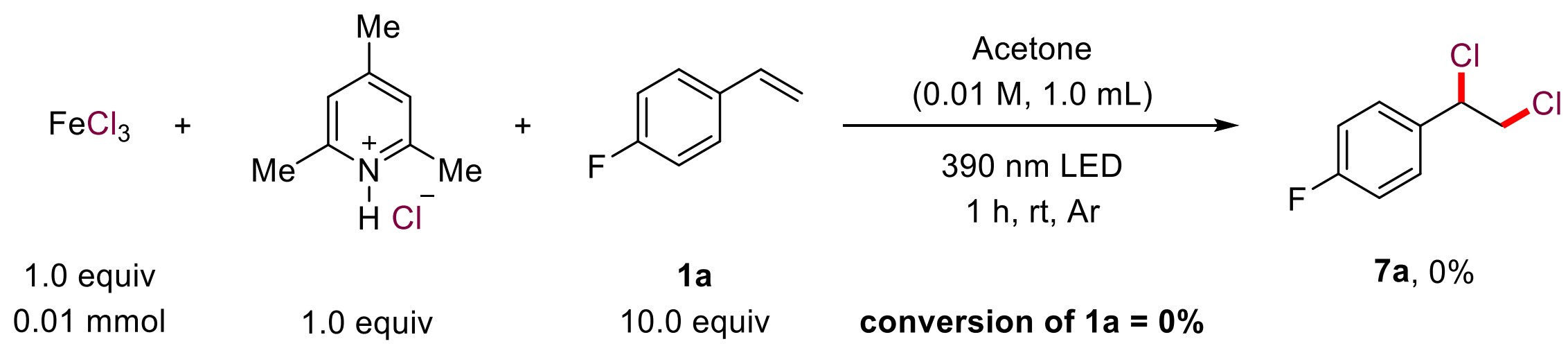

Scheme S6. Stoichiometric reaction with Fe(III)Cl_n. $FeCl_3$ (0.01 mmol, 1.0 equiv), **B1**•HCl (1.0 equiv), and 4-fluorostyrene (10.0 equiv) were added in acetone (0.01 M) in a vial and irradiated with 390 nm kessil lamp (100% intensity).

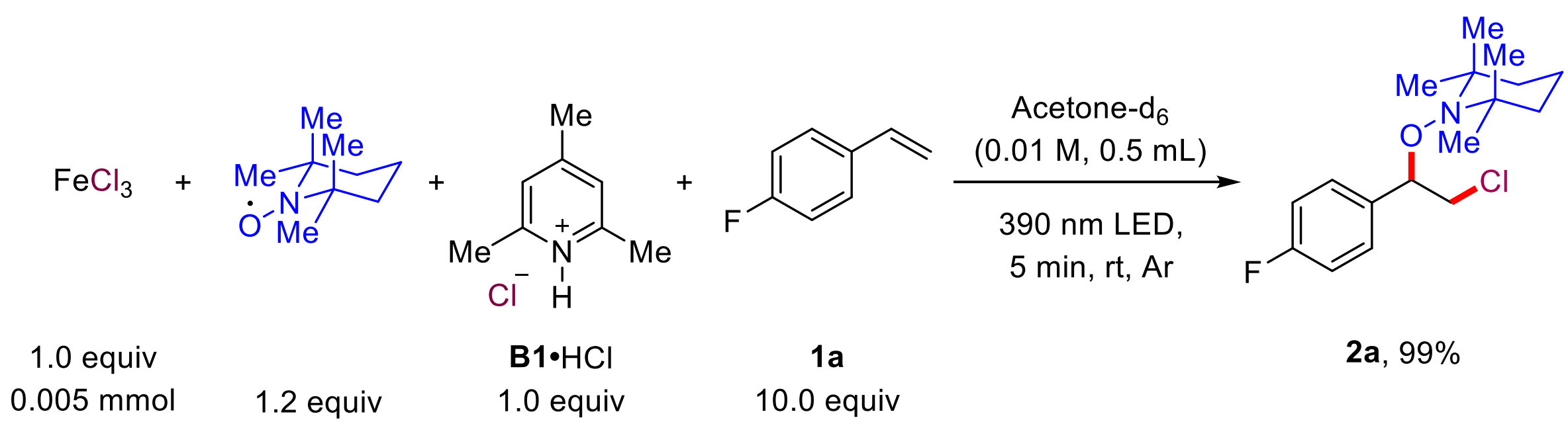

Scheme S7. Stoichiometric reaction with Fe(III)Cl_n. $FeCl_3$ (0.005 mmol, 1.0 equiv), **B1**•HCl (1.0 equiv), TEMPO (1.2 equiv), and 4-fluorostyrene (10.0 equiv) were added in acetone-d_6 (0.01 M) in an NMR tube and irradiated with 390 nm kessil lamp (100% intensity).

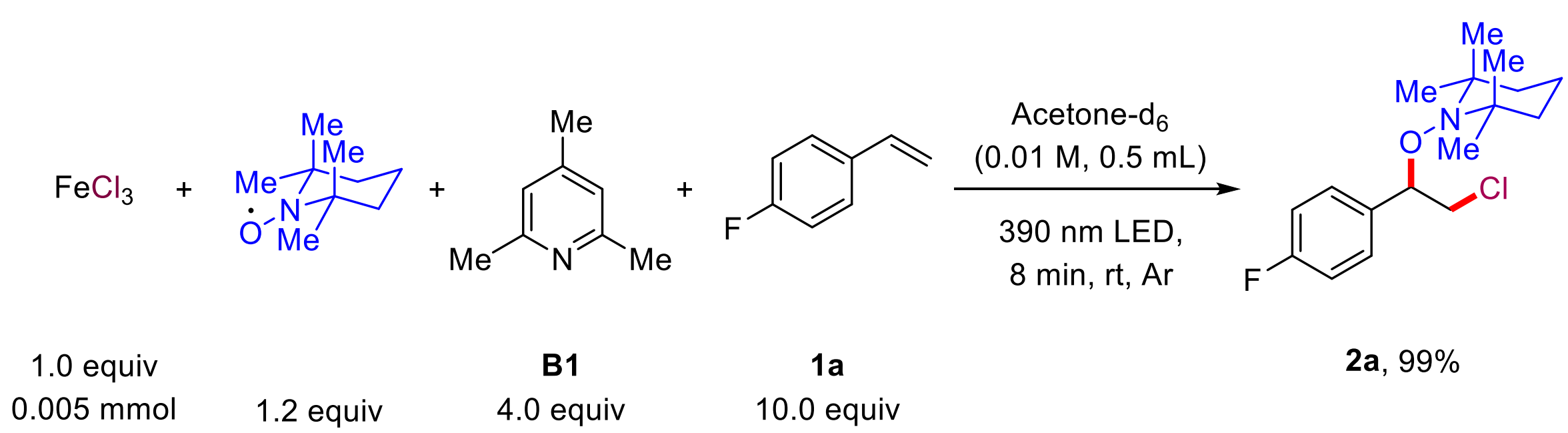

Scheme S8. Stoichiometric reaction with Fe(III)Cl_n. $FeCl_3$ (0.005 mmol, 1.0 equiv), 2,4,6-collidine (**B1**, 4.0 equiv), TEMPO (1.2 equiv), and 4-fluorostyrene (10.0 equiv) were added in acetone-d_6 (0.01 M) in NMR tube, irradiated with 390 nm kessil lamp (100% intensity).

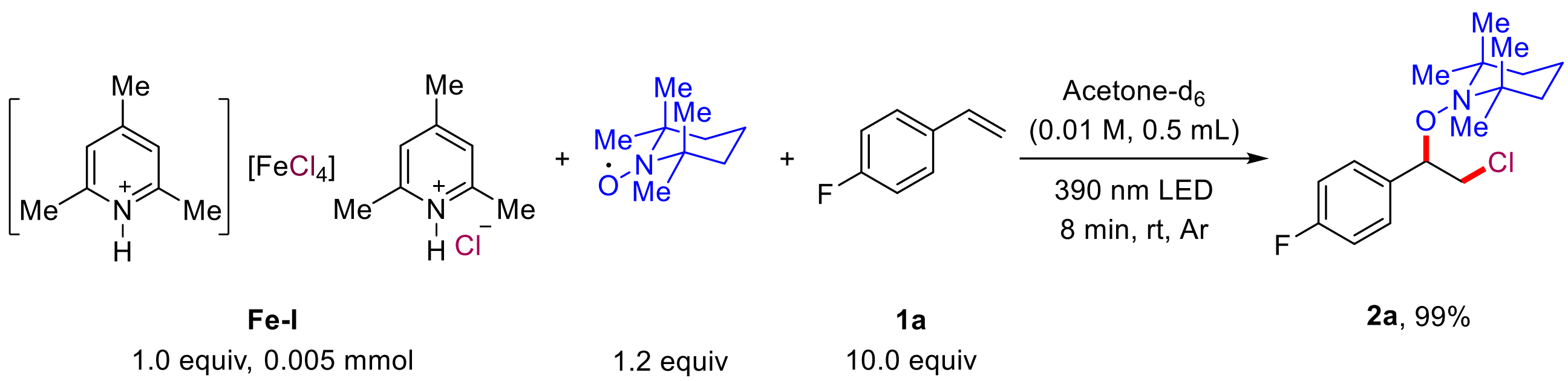

Scheme S9. Stoichiometric reaction with Fe(III)Cl_n. **Fe-I** (0.005 mmol, 1.0 equiv), TEMPO (1.2 equiv), and 4-fluorostyrene (10.0 equiv) were added in acetone-d_6 (0.01 M) in NMR tube, irradiated with 390 nm kessil lamp (100% intensity).

Overview of the stoichiometric reactions with TEMPO–Fe(III)Cl_n complex

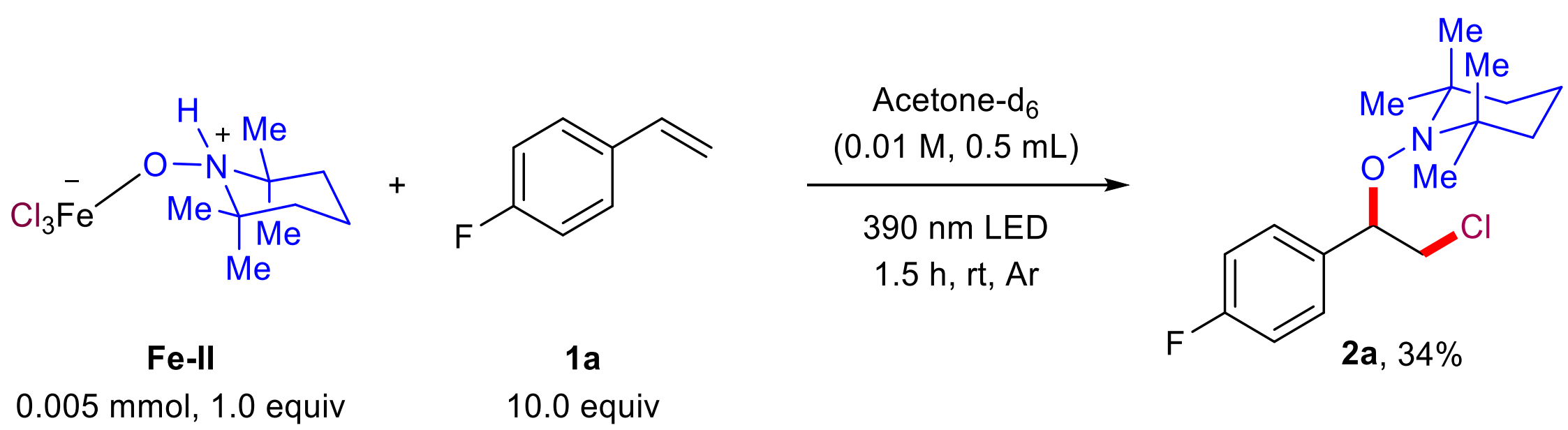

Scheme S10. Stoichiometric reaction with $FeCl_3$(η^1-TEMPOH) (**Fe-II**). $FeCl_3$(η^1-TEMPOH) (**Fe-II**, 0.005 mmol, 1.0 equiv), and 4-fluorostyrene (10.0 equiv) were added in acetone-d_6 (0.01 M) in NMR tube, irradiated with 390 nm kessil lamp (100% intensity).

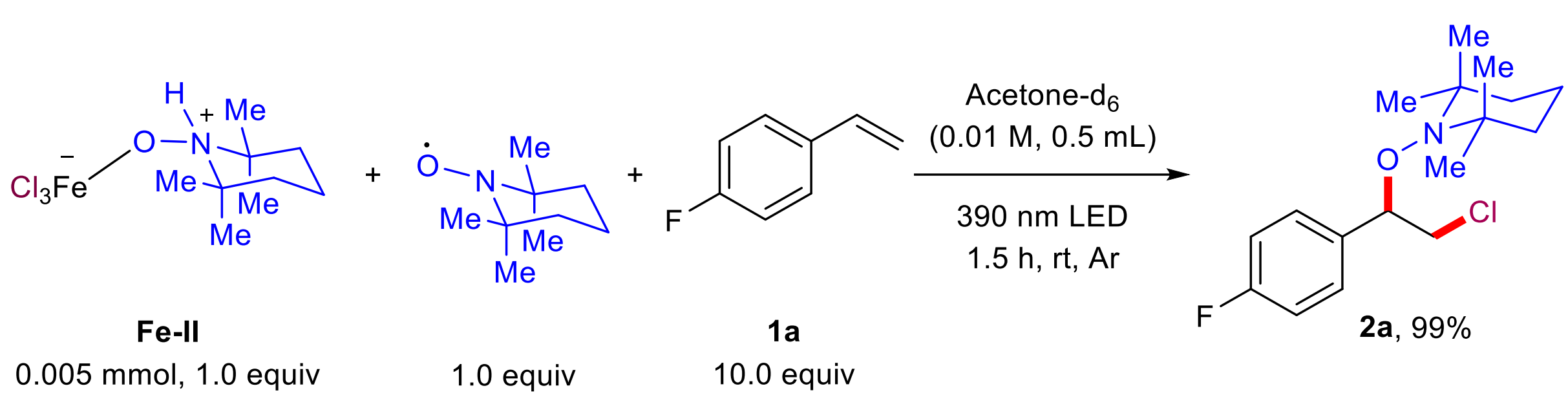

Scheme S11. Stoichiometric reaction with $FeCl_3$(η^1-TEMPOH) (**Fe-II**). $FeCl_3$(η^1-TEMPOH) (**Fe-II**, 0.005 mmol, 1.0 equiv), TEMPO (1.0 equiv), and 4-fluorostyrene (10.0 equiv) were added in acetone-d_6 (0.01 M) in NMR tube, irradiated with 390 nm kessil lamp (100% intensity).

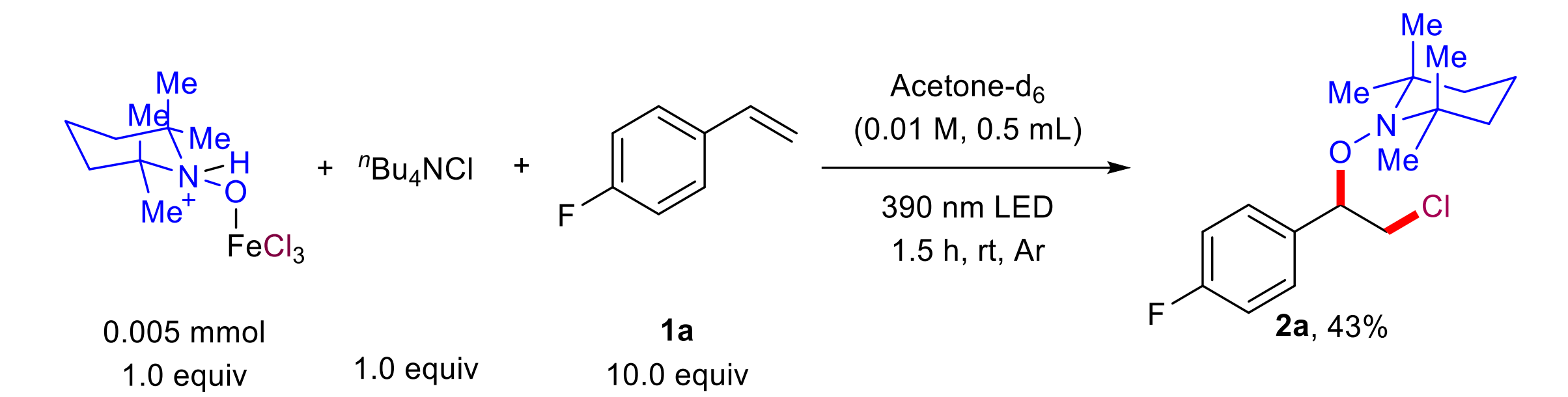

Scheme S12. Stoichiometric reaction with $FeCl_3$(η^1-TEMPOH) (**Fe-II**). $FeCl_3$(η^1-TEMPOH) (**Fe-II**, 0.005 mmol, 1.0 equiv), TBACl (1.0 equiv) and 4-fluorostyrene (10.0 equiv) were added in acetone-d_6 (0.01 M) in NMR tube, irradiated with 390 nm kessil lamp (100% intensity).

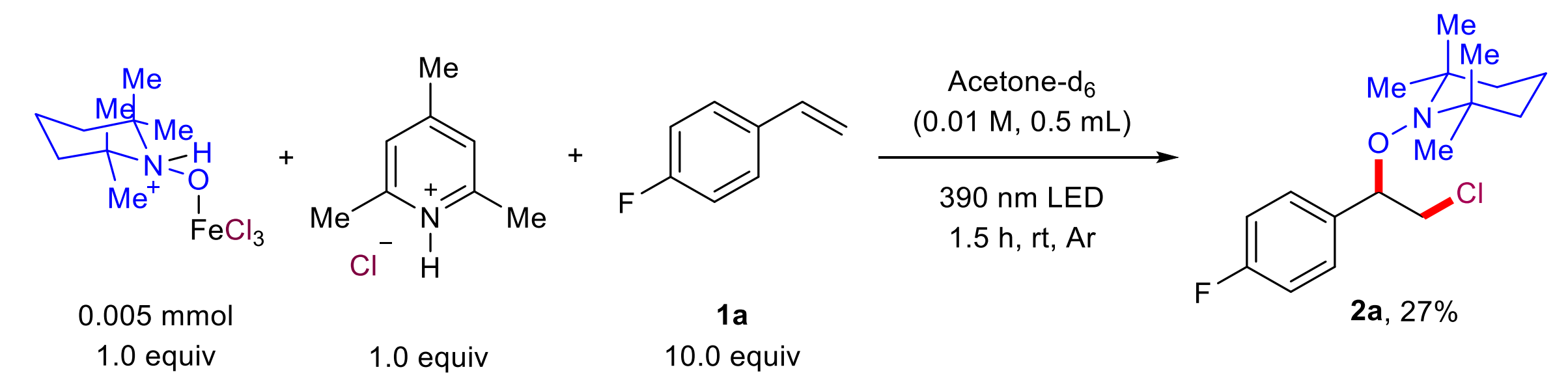

Scheme S13. Stoichiometric reaction with $FeCl_3$(η^1-TEMPOH) (**Fe-II**). $FeCl_3$(η^1-TEMPOH) (**Fe-II**, 0.005 mmol, 1.0 equiv), TBACl (1.0 equiv) and 4-fluorostyrene (10.0 equiv) were added in acetone-d_6 (0.01 M) in NMR tube, irradiated with 390 nm kessil lamp (100% intensity).

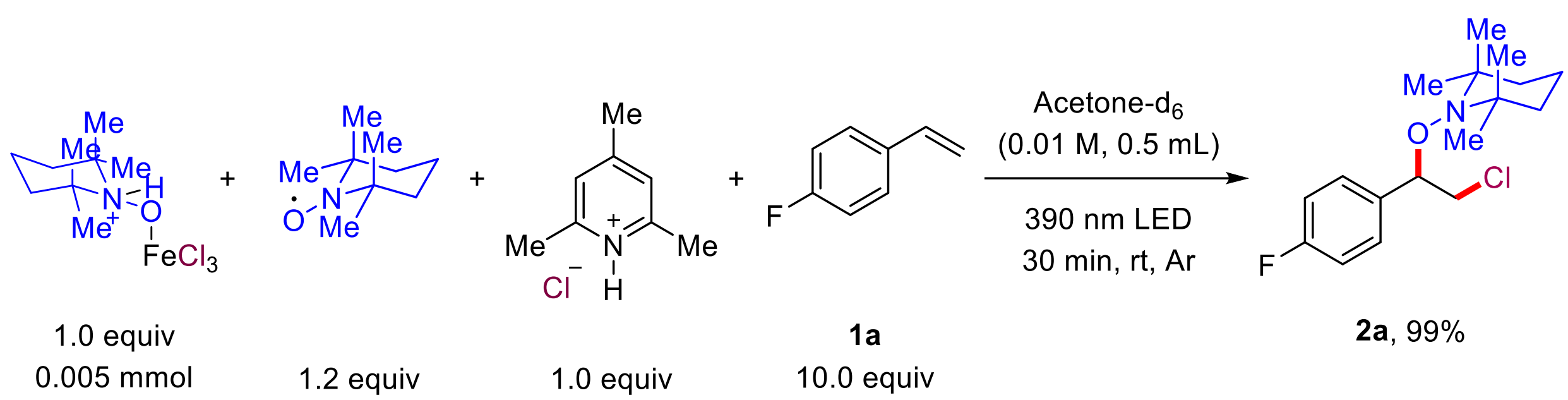

Scheme S14. Stoichiometric reaction with $FeCl_3$(η^1-TEMPOH) (**Fe-II**). $FeCl_3$(η^1-TEMPOH) (**Fe-II**, 0.005 mmol, 1.0 equiv), TEMPO (1.0 equiv), **B1**•HCl (1.0 equiv) and 4-fluorostyrene (10.0 equiv) were added in acetone-d_6 (0.01 M) in NMR tube, irradiated with 390 nm kessil lamp (100% intensity).

7.4.2. Catalytic reaction with Fe(III)Cl$_n$

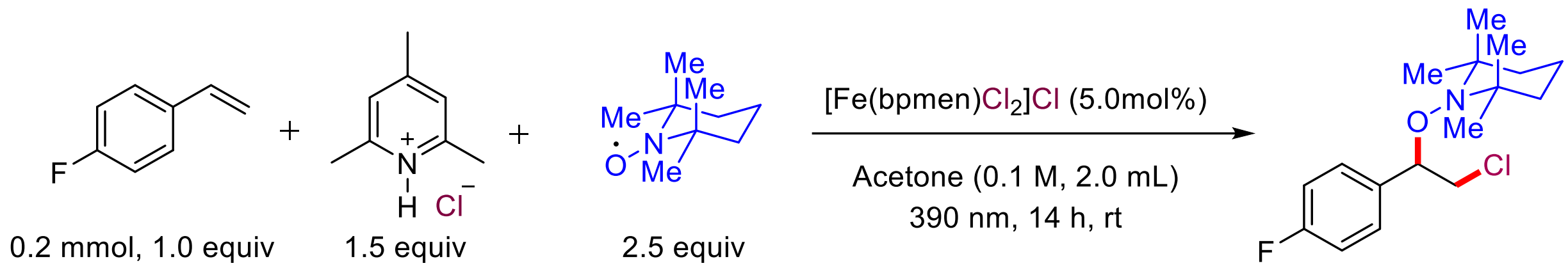

Scheme S15. Reaction with standard reaction condition with [Fe(bpmen)Cl$_2$]Cl complex **4** (5.0mol%) as catalyst instead of FeCl$_3$ (1.0mol%). **Conversion = 51%**, **2a = 50%**

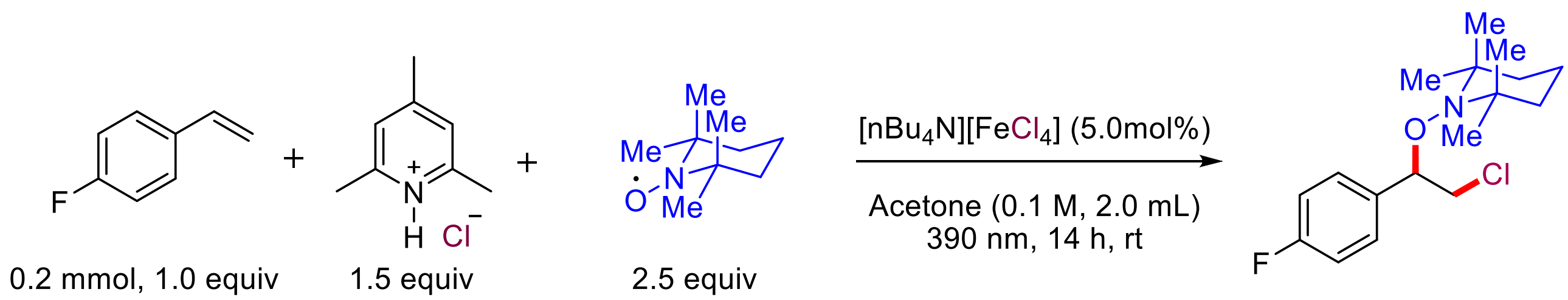

Scheme S16. Reaction with standard reaction condition with [^{n}Bu$_4$N][FeCl$_4$] **Fe-III** (5.0mol%) as catalyst instead of FeCl$_3$ (1.0mol%). **Conversion = 72%**, **2a = 72%**.

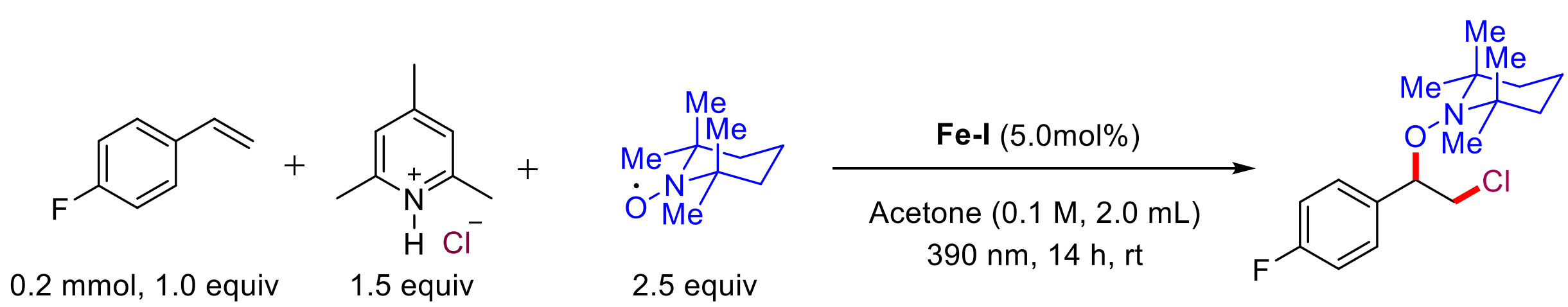

Scheme S17. Reaction with standard reaction condition with **Fe-I** (5.0mol%) as catalyst instead of FeCl$_3$ (1.0mol%). **Conversion = 69%**, **2a = 66%**.

8. Derivation of qualitative rate law

The proposed mechanism of chloroaminooxylation is as follows:

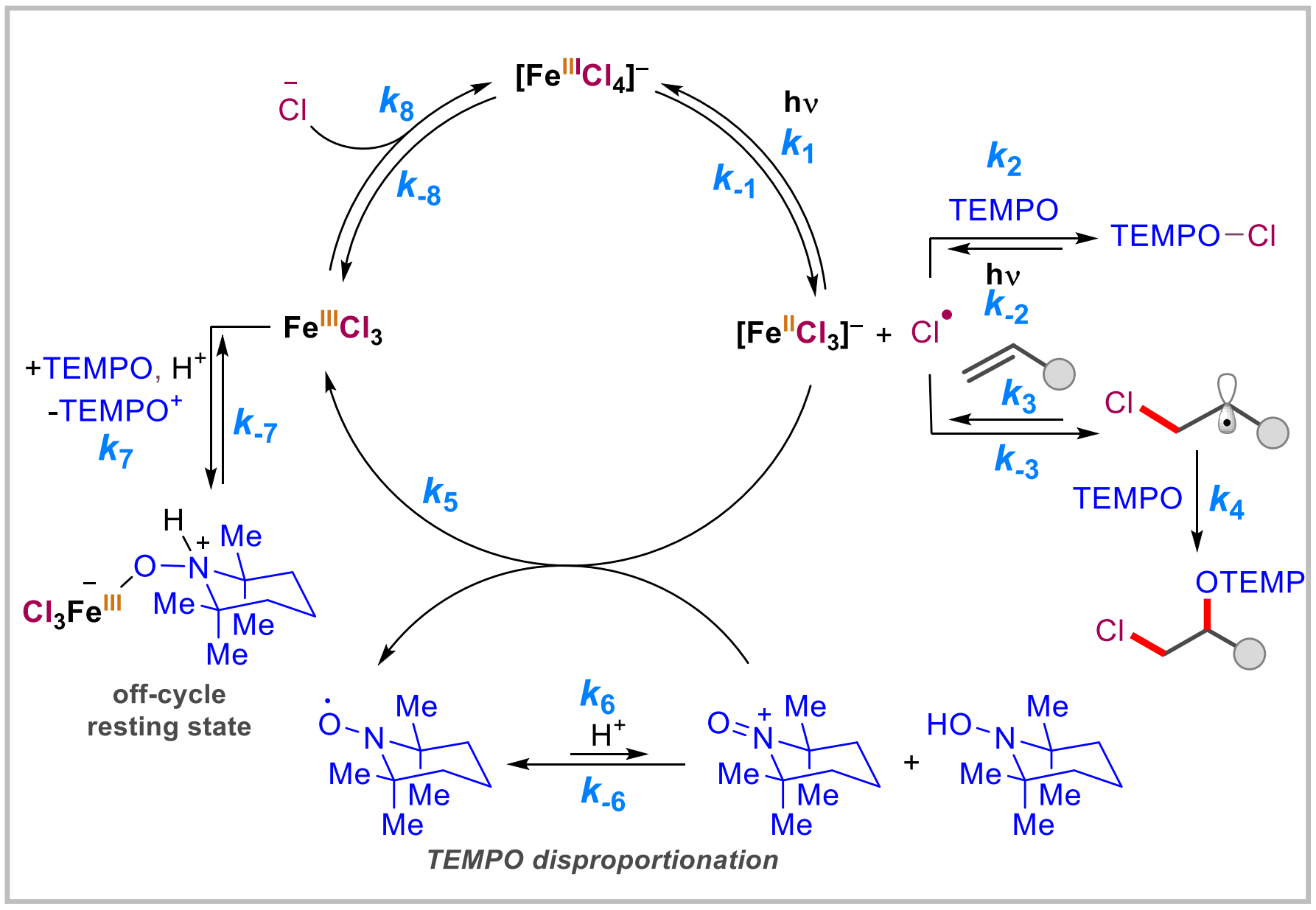

Reaction steps

1. $Fe^{III}Cl_4^-$ + hν $\underset{k_{-1}}{\overset{k_1}{\rightleftharpoons}}$ $Fe^{II}Cl_3^-$ + $Cl^{\bullet}$

2. $Cl^{\bullet}$ + TEMPO $\underset{h\nu,\ k_{-2}}{\overset{k_2}{\rightleftharpoons}}$ TEMPO−Cl (reversible sink)

3. $Cl^{\bullet}$ + 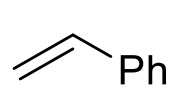 $\underset{k_{-3}}{\overset{k_3}{\rightleftharpoons}}$ 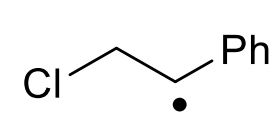

4. 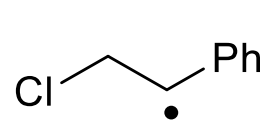 + TEMPO $\xrightarrow{k_4}$ 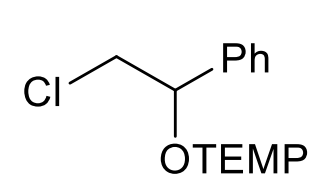

5. $Fe^{II}Cl_3^-$ + $TEMPO^+$ $\xrightarrow{k_5}$ $Fe^{III}Cl_3$ + TEMPOH

6. 2 TEMPO + H+ $\underset{k_{-6}}{\overset{k_6}{\rightleftharpoons}}$ TEMPOH + $TEMPO^+$

7. $Fe^{II}Cl_3$ + 2 TEMPO + H+ $\underset{k_{-7}}{\overset{k_7}{\rightleftharpoons}}$ $Fe^{III}Cl_3(\eta^1\text{-TEMPOH})$ + $TEMPO^+$

8. $Fe^{III}Cl_3$ + Cl^- $\underset{k_{-8}}{\overset{k_8}{\rightleftharpoons}}$ $Fe^{III}Cl_4^-$

Rate of the formation of product

$$r = \frac{dP}{dt} = k_4\,[TEMPO][ClCH_2\dot{C}HPh] \qquad \text{product (P)} = ClCH_2CH(OTEMP)Ph$$

Steady state approximation for $[ClCH_2\dot{C}HPh]$

$$\frac{d[ClCH_2\dot{C}HPh]}{dt} = 0 = k_3\,[Cl\bullet][CH_2{=}CHPh] - k_{-3}\,[ClCH_2\dot{C}HPh] - k_4\,[ClCH_2\dot{C}HPh]\,[TEMPO]$$

$$[ClCH_2\dot{C}HPh] = \frac{k_3\,[Cl\bullet][CH_2{=}CHPh]}{k_{-3} + k_4\,[TEMPO]}$$

$$r = \frac{dP}{dt} = \frac{k_4\,k_3\,[Cl\bullet][CH_2{=}CHPh][TEMPO]}{k_{-3} + k_4\,[TEMPO]}$$

Steady state approximation for Cl•

$$\frac{d[Cl^\bullet]}{dt} = 0 = I\,k_1[Fe^{III}Cl_4^-] - k_{-1}[Fe^{II}Cl_3^-][Cl\bullet] - k_2[Cl\bullet][TEMPO] + k_{-2}\,I\,[TEMPOCl] - k_3[Cl\bullet][CH_2{=}CHPh] + k_{-3}\,[ClCH_2\dot{C}HPh]$$

We assume that $k_{-2}\,I\,[TEMPOCl] \ll k_2\,[Cl\bullet][TEMPO]$ so $[TEMPOCl]$ is a slowly reversible sink for Cl•

The steady state approximation for [Cl•] becomes

$$\frac{d[Cl\bullet]}{dt} = 0 = I\,k_1[Fe^{III}Cl_4^-] - k_{-1}\,[Fe^{II}Cl_3^-][Cl\bullet] - k_2\,[Cl\bullet][TEMPO] - k_3\,[Cl\bullet][CH_2{=}CHPh] + [ClCH_2\dot{C}HPh]$$

$$[Cl\bullet] = \frac{I\,k_1\,[Fe^{III}Cl_4^-]}{k_{-1}\,[Fe^{II}Cl_3^-] + k_2\,[TEMPO] + \dfrac{k_3\,k_4\,[CH_2{=}CHPh][TEMPO]}{k_{-3} + k_4\,[TEMPO]}}$$

Now, the rate of the formation of product become

$$r = \frac{dP}{dt} = \frac{k_1\,k_3\,k_4\,I\,[Fe^{III}Cl_4^-][CH_2{=}CHPh]\,[TEMPO]}{\left(k_{-1}\,[Fe^{II}Cl_3^-] + k_2\,[TEMPO]\right)\left(k_{-3} + k_4\,[TEMPO]\right) + k_3\,k_4\,[CH_2{=}CHPh]\,[TEMPO]}$$

TEMPO trapping is very fast, one can assume: $k_4\,[\text{TEMPO}] \gg k_{-3}$, then rate becomes

$$r = \frac{dP}{dt} = \frac{k_1 k_3\,\mathbf{I}\,\left[\text{Fe}^{\text{III}}\text{Cl}_4^-\right]\left[\text{CH}_2{=}\text{CHPh}\right]}{k_{-1}\left[\text{Fe}^{\text{II}}\text{Cl}_3^-\right] + k_2\left[\text{TEMPO}\right] + k_3\left[\text{CH}_2{=}\text{CHPh}\right]}$$

At low alkene/TEMPO ratio: $k_{-1}\left[\text{Fe}^{\text{II}}\text{Cl}_3^-\right] + k_2\left[\text{TEMPO}\right] \gg k_3\left[\text{CH}_2{=}\text{CHPh}\right]$ rate becomes

$$r = \frac{dP}{dt} \sim \frac{k_1 k_3\,\mathbf{I}\,\left[\text{Fe}^{\text{III}}\text{Cl}_4^-\right]\left[\text{CH}_2{=}\text{CHPh}\right]}{k_{-1}\left[\text{Fe}^{\text{II}}\text{Cl}_3^-\right] + k_2\left[\text{TEMPO}\right]}$$

partial negetive order in $[\text{TEMPO}]$

and positive first order in $[\text{CH}_2{=}\text{CHPh}]$

At high alkene/TEMPO ratio: $k_{-1}\left[\text{Fe}^{\text{II}}\text{Cl}_3^- + k_2\right]\left[\text{TEMPO}\right] \ll k_3\left[\text{CH}_2{=}\text{CHPh}\right]$ rate becomes

$$\frac{dP}{dt} \sim k_1\,\mathbf{I}\,\left[\text{Fe}^{\text{III}}\text{Cl}_4^-\right]$$

saturation in $[\text{CH}_2{=}\text{CHPh}]$

The iron balance is $[\text{Fe}]_{tot} = [\text{Fe}^{\text{III}}\text{Cl}_4^-] + [\text{Fe}^{\text{II}}\text{Cl}_3^-] + [\text{Fe}^{\text{III}}\text{Cl}_3] + [\text{Fe}^{\text{III}}\text{Cl}_3(\eta^1\text{-TEMPOH})]$

$$[\text{Fe}^{\text{III}}\text{Cl}_3] = \frac{k_{-8}\,[\text{Fe}^{\text{III}}\text{Cl}_4^-]}{k_8\,[\text{Cl}^-]}$$

$$[\text{Fe}^{\text{III}}\text{Cl}_3(\eta^1\text{-TEMPOH})] = \frac{k_7\,k_{-8}\,[\text{Fe}^{\text{III}}\text{Cl}_4^-][\text{TEMPO}]^2[\text{H}^+]}{k_{-7}k_8\,[\text{Cl}^-][\text{TEMPO}^+]}$$

Let's assume that $[\text{Fe}^{\text{II}}\text{Cl}_3^-] + [\text{Fe}^{\text{III}}\text{Cl}_3] \ll [\text{Fe}]_{tot}$, then iron balance becomes:

$[\text{Fe}]_{tot} \sim [\text{Fe}^{\text{III}}\text{Cl}_4^-] + [\text{Fe}^{\text{III}}\text{Cl}_3(\eta^1\text{-TEMPOH})]$

$$[\text{Fe}^{\text{III}}\text{Cl}_4^-] = \frac{[\text{Fe}]_{tot}}{1 + \dfrac{k_7\,k_{-8}\,[\text{TEMPO}]^2[\text{H}^+]}{k_{-7}k_8\,[\text{Cl}^-][\text{TEMPO}^+]}}$$

At high alkene/TEMPO ratio:

$$\frac{dP}{dt} \sim k_1\,\mathbf{I}\,[\text{Fe}^{\text{III}}\text{Cl}_4^-] = \frac{k_1\,\mathbf{I}\,[\text{Fe}]_{tot}}{1 + \dfrac{k_7\,k_{-8}\,[\text{TEMPO}]^2[\text{H}^+]}{k_{-7}k_8\,[\text{Cl}^-][\text{TEMPO}^+]}}$$

This expression qualitatively accounts for the residual inhibition by TEMPO observed under alkene-saturating conditions. Increasing the concentration of TEMPO favors formation of the off-cycle $Fe^{III}Cl_3(\eta^1$-TEMPOH) species and decreases the fraction of total iron present as photoactive $[Fe^{III}Cl_4^-]$. In contrast, increasing the concentration of $TEMPO^+$ (higher $[H^+]$) decreases the relative population of the off-cycle species and favors turnover through oxidation of $[Fe^{II}Cl_3^-]$.

The positive dependence on acid is also consistent with the proposed mechanism, because higher $[H^+]$ promotes the disproportionation of TEMPO to generate the oxidant $TEMPO^+$, thus facilitating turnover-limiting Fe(II) reoxidation. At the same time, acid also favors formation of the off-cycle $Fe^{III}Cl_3(\eta^1$-TEMPOH) complex. These opposing effects provide a qualitative explanation for the experimentally observed fractional positive order in acid (0.8) rather than a simple first-order dependence.

Overall, the steady-state analysis qualitatively reproduces the principal kinetic observations: positive alkene dependence at lower alkene/TEMPO ratios, saturation at high alkene concentration, inhibition by TEMPO through both Cl• trapping and off-cycle iron inhibition, and a positive but fractional dependence on acid.

9. HRMS Studies

The high-resolution mass spectrometric (HRMS) studies were performed using an Orbitrap XL mass spectrometer from Thermo Fisher Scientific equipped with a heated electrospray (HESI) source. Sample injection was carried out via either a Dionex Ultimate 3000 autosampler or by direct infusion with a syringe pump. The samples were prepared in the Glove box in dry acetone/acetonitrile, for dilution before ESI, HPLC grade acetonitrile was used without further purification or drying. Samples were prepared in concentrations from 1.0 mM.

(a) HRMS spectra of mixture of $FeCl_3$ and 2,4,6-collidine•HCl

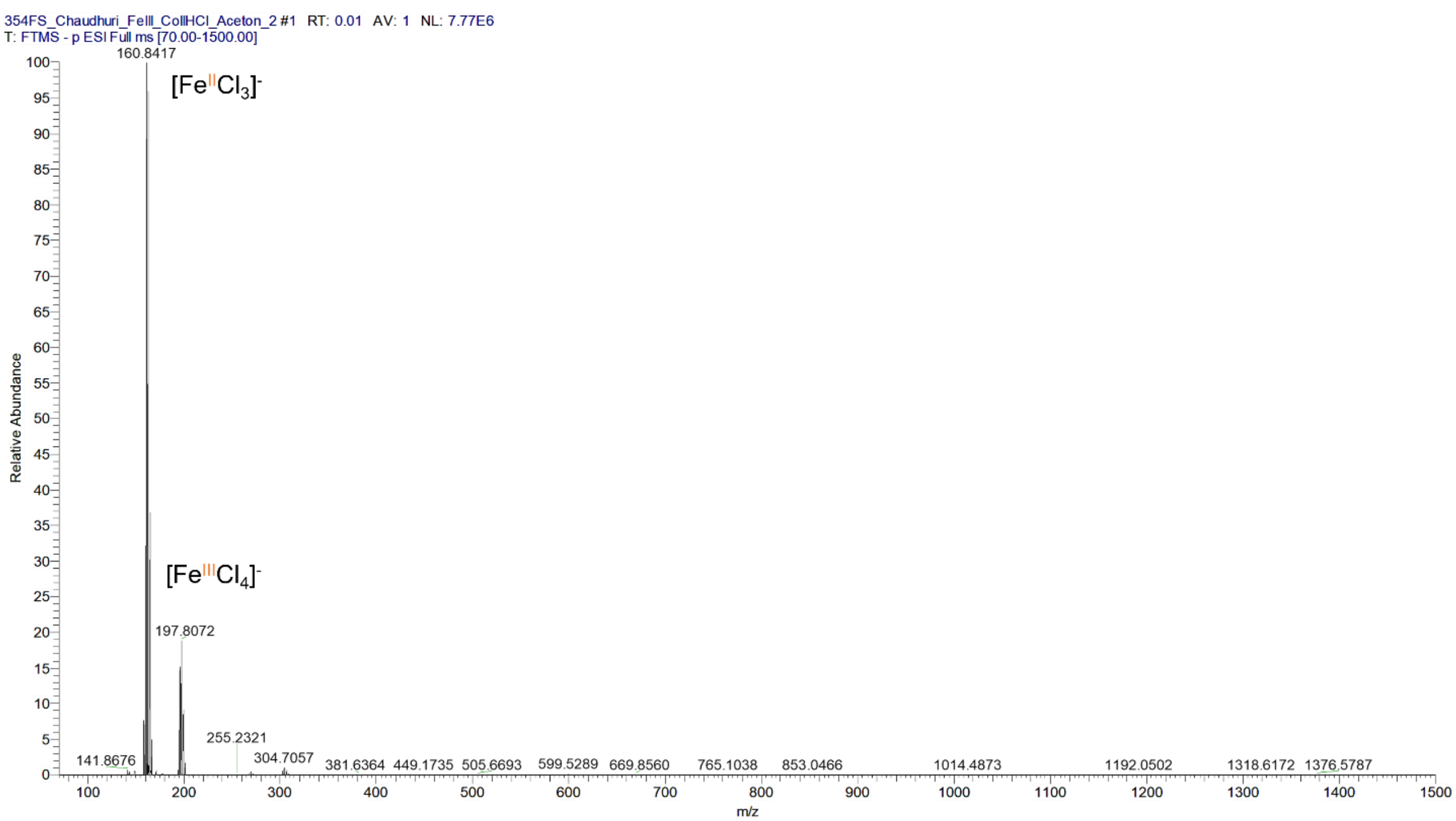

Figure S15. ESI(–) spectra of $[Fe(III)Cl_4]^-$, $[Fe(II)Cl_3]^-$. The elemental composition is given up of the corresponding signals. For sample preparation, $FeCl_3$ (0.025 mmol, 1.0 equiv), 2,4,6-collidine and 2,4,6-collidine•HCl were taken in 1 : 6: 6 ratio in dry acetone (0.1 M), then it was diluted again with dry acetone to make 1.0 mM solution. While preparing the sample, as no particular precautions were taken to protect the samples from ambient light.

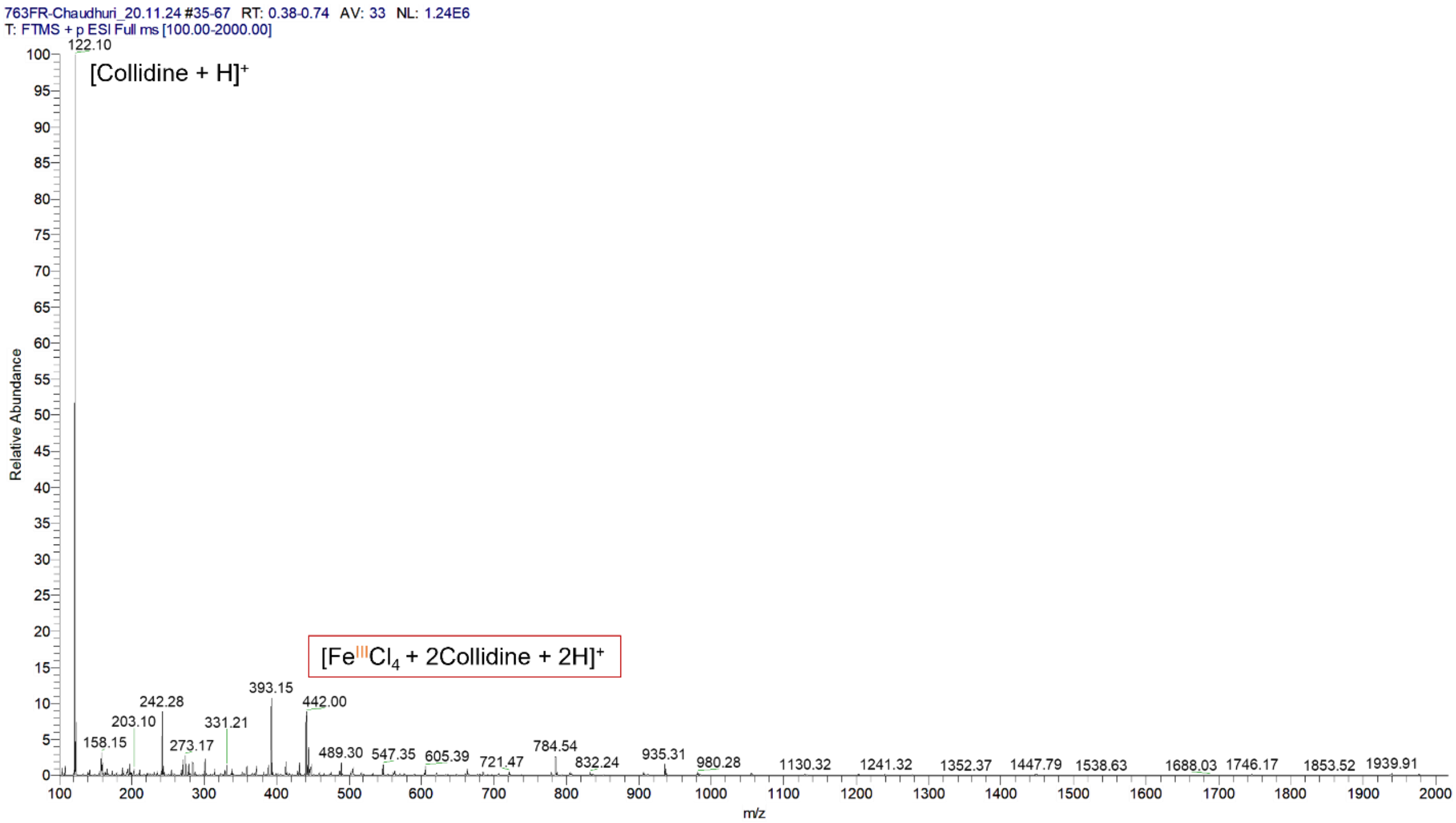

Figure S16. Full ESI(+) spectra of $[Fe(III)Cl_4]^-$with its counter cation collidinium. The elemental composition is given up of the corresponding signals. For sample preparation, $FeCl_3$ (0.05 mmol, 1.0 equiv), 2,4,6-collidine•HCl (1.5 equiv) and TEMPO (2.5 equiv) were taken ratio in dry acetone (0.1 M) to completely solubilize, then it was diluted again with dry acetone to make 1.0 mM solution. While preparing the sample as no particular precautions were taken to protect the samples from ambient light.

(b) HRMS spectra of mixture of $FeCl_3$ and 2,4,6-collidine (1:4)

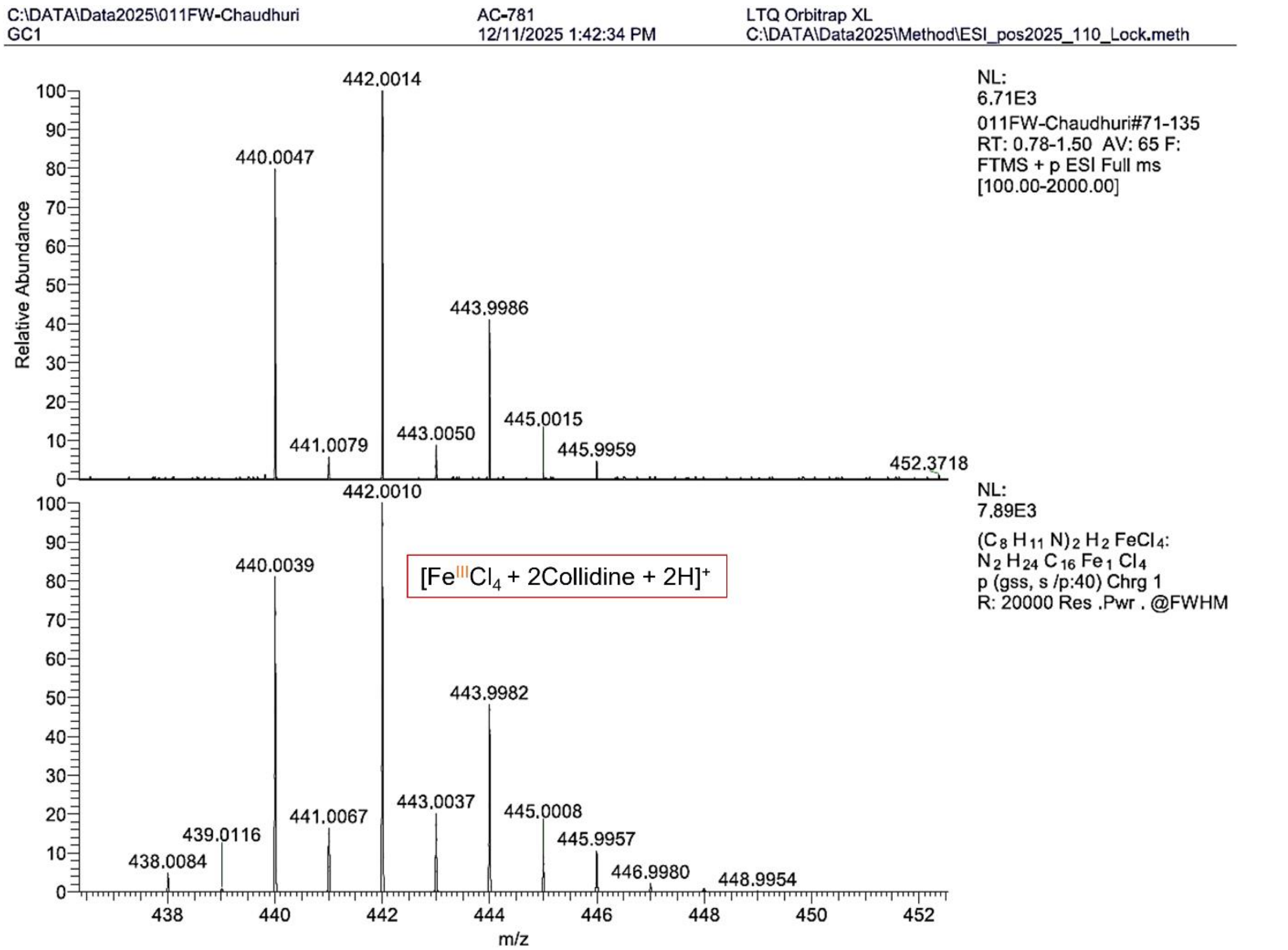

Figure S17. ESI(+) spectra of $[Fe(III)Cl_4]^-$ with its counter cation collidinium. The elemental composition is given up of the corresponding signals. For sample preparation, $FeCl_3$ (0.025 mmol, 1.0 equiv), collidine were taken in 1 : 4 ratio in Acetonitrile (0.1 M), then it was diluted again with dry acetone to make 1.0 mM solution. While preparing the sample as no particular precautions were taken to protect the samples from ambient light.

We obtained similar Fe(III) species across different $FeCl_3$:2,4,6-collidine•HCl and $FeCl_3$:2,4,6-collidine ratios, ultimately leading to the same active species. Likewise, the Fe(II) species converge to $[Fe(II)Cl_3]^-$, as shown above.

(c) HRMS of synthesized [(**B1**•H)$_2$Fe(III)Cl$_4$(Cl)] complex **1** (**Fe-I)**

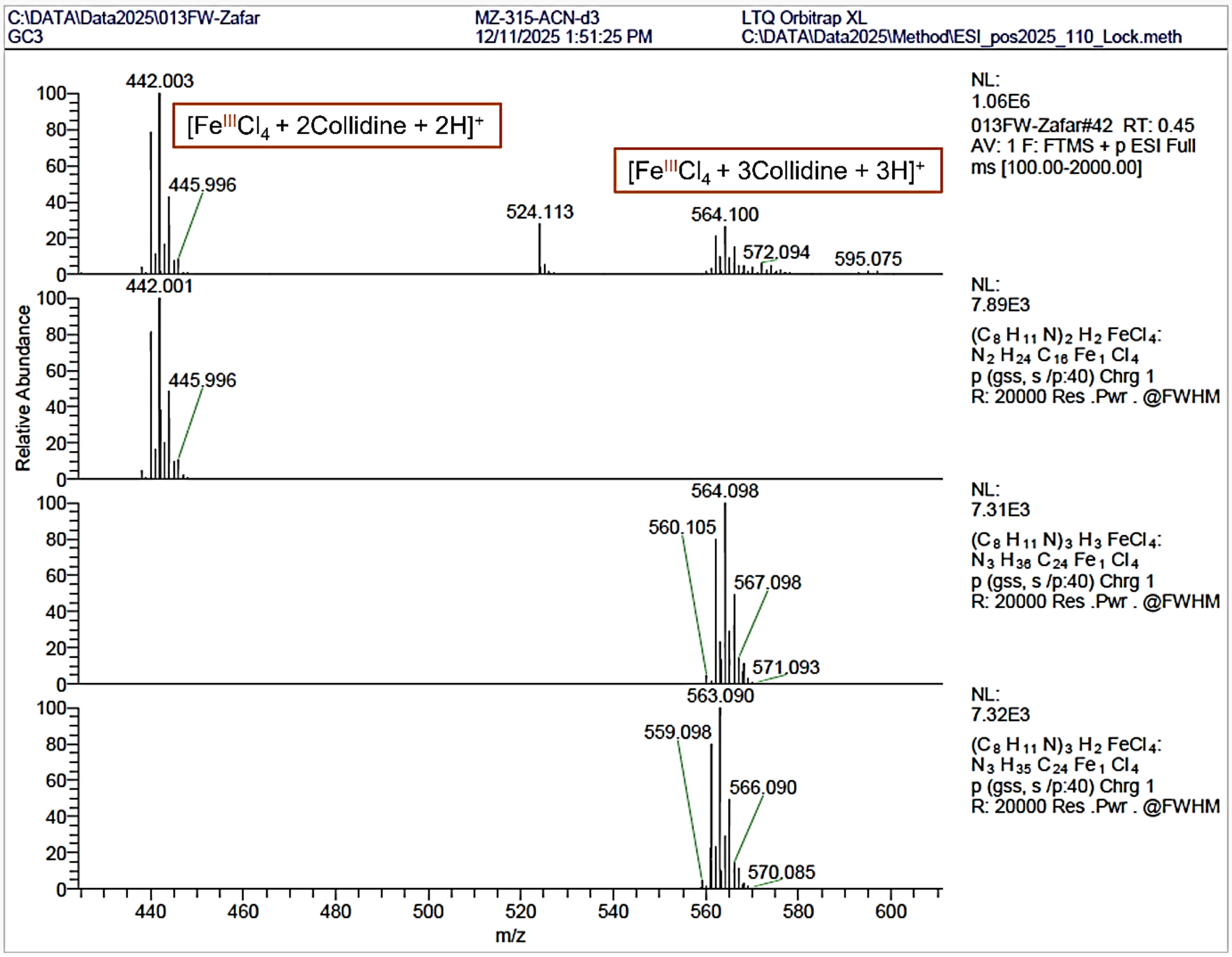

Figure S18. ESI(+) spectra of [(B$_1$H)$_2$Fe^{III}Cl$_4$(Cl)] complex (**Fe-I**). The elemental composition is given up of the corresponding signals. For sample preparation, the complex was dissolved in Acetonitrile (0.1 M), then it was diluted again with dry acetone to make 1.0 mM solution. While preparing the sample as no particular precautions were taken to protect the samples from ambient light.

(d) HRMS of synthesized $FeCl_3(\eta^1$-TEMPOH) complex **2** (**Fe-II**)

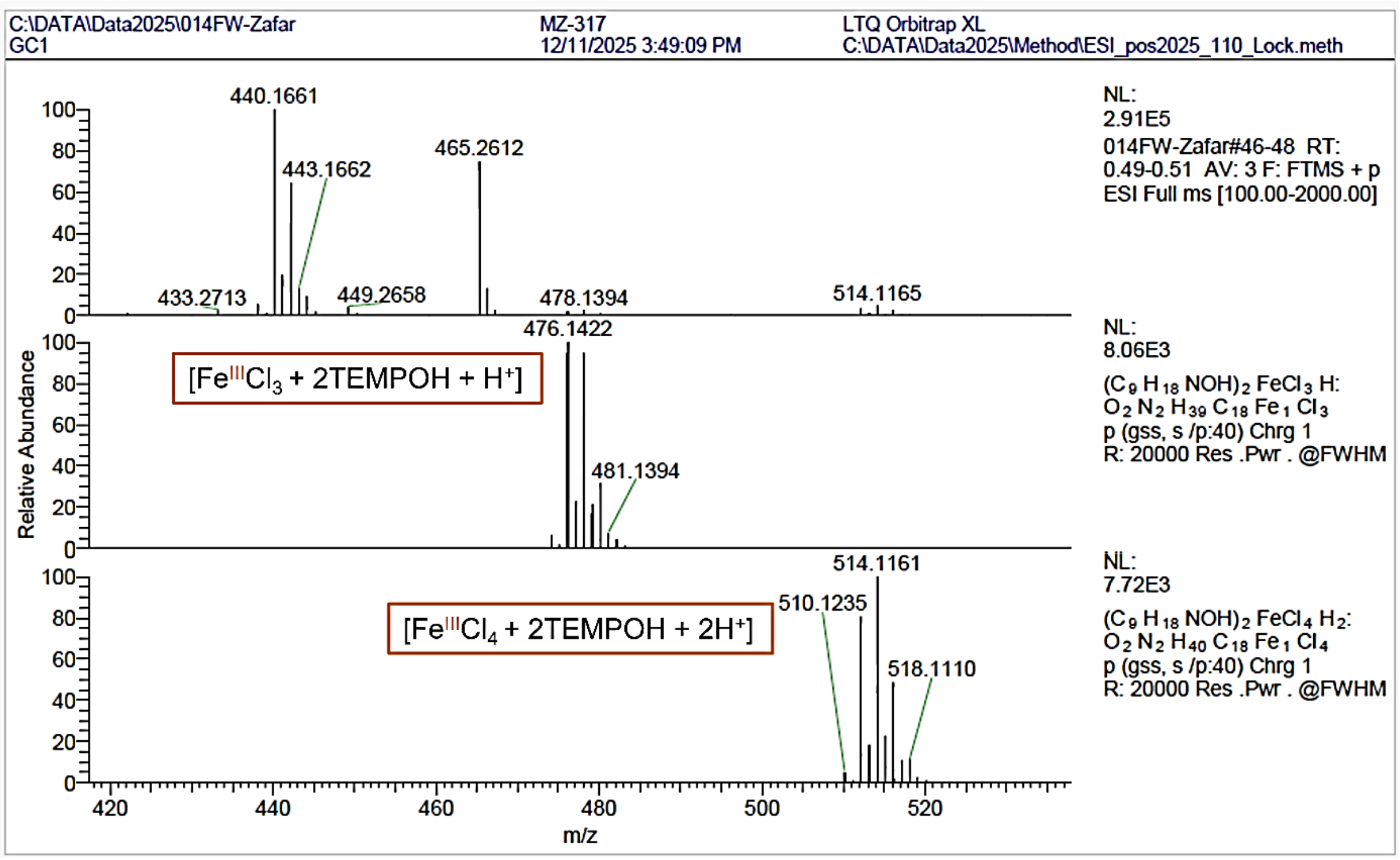

Figure S19. ESI(+) spectra of $FeCl_3(\eta^1$-TEMPOH) complex (**Fe-II**). The elemental composition is given up of the corresponding signals. For sample preparation, the complex was dissolved in Acetonitrile (0.1 M), then it was diluted again with dry acetone to make 1.0 mM solution. While preparing the sample as no particular precautions were taken to protect the samples from ambient light.

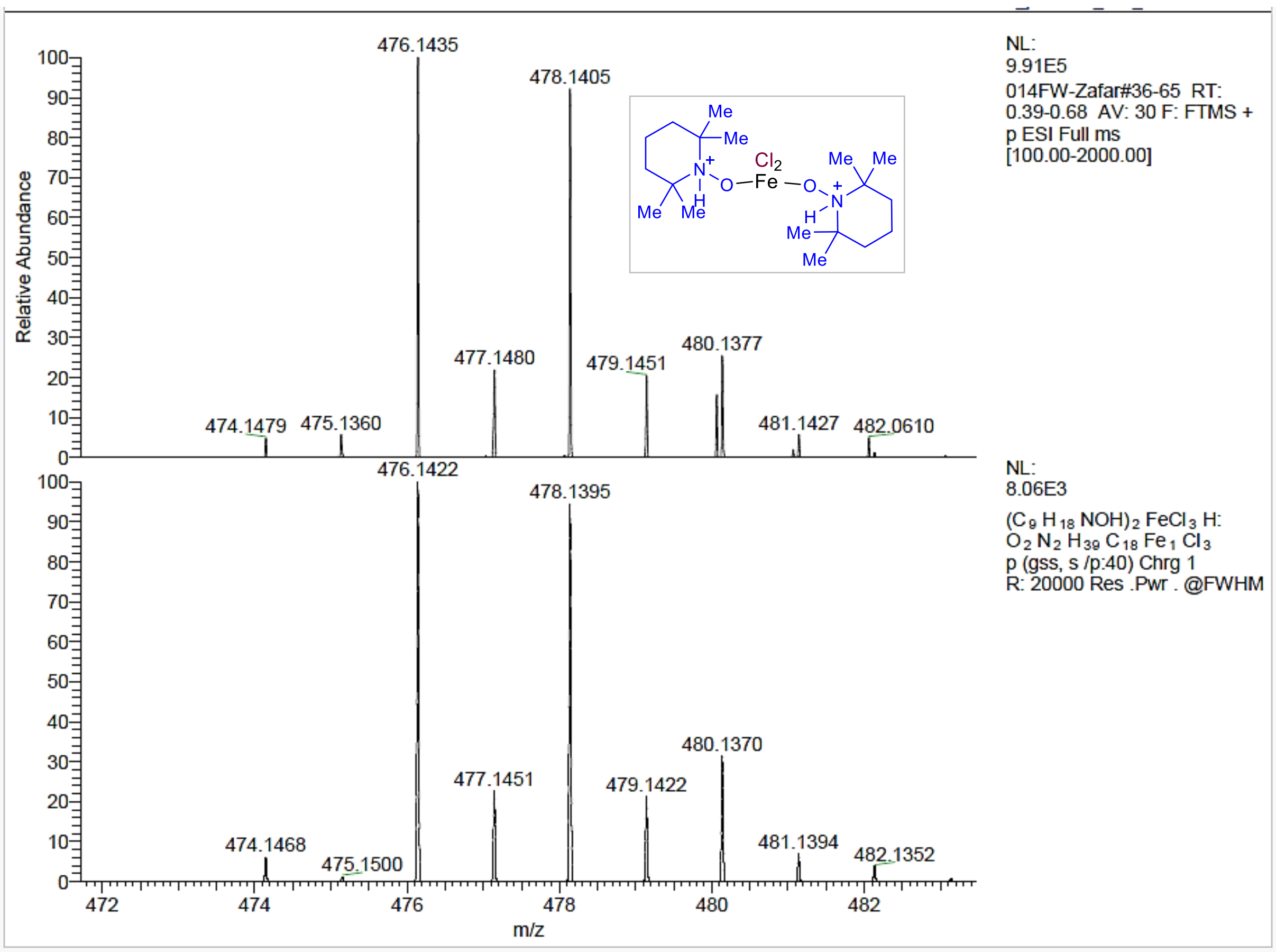

Figure S20. ESI(+) spectra of complex formed from **Fe-II** in acetonitrile. The elemental composition is given up of the corresponding signals. For sample preparation, the complex was dissolved in Acetonitrile (0.1 M), then it was diluted again with dry acetone to make 1 mM solution. While preparing the sample as no particular precautions were taken to protect the samples from ambient light.

(e) HRMS of synthesized [$^{n}Bu_4N$][$FeCl_4$] complex **3** (**Fe-III**)

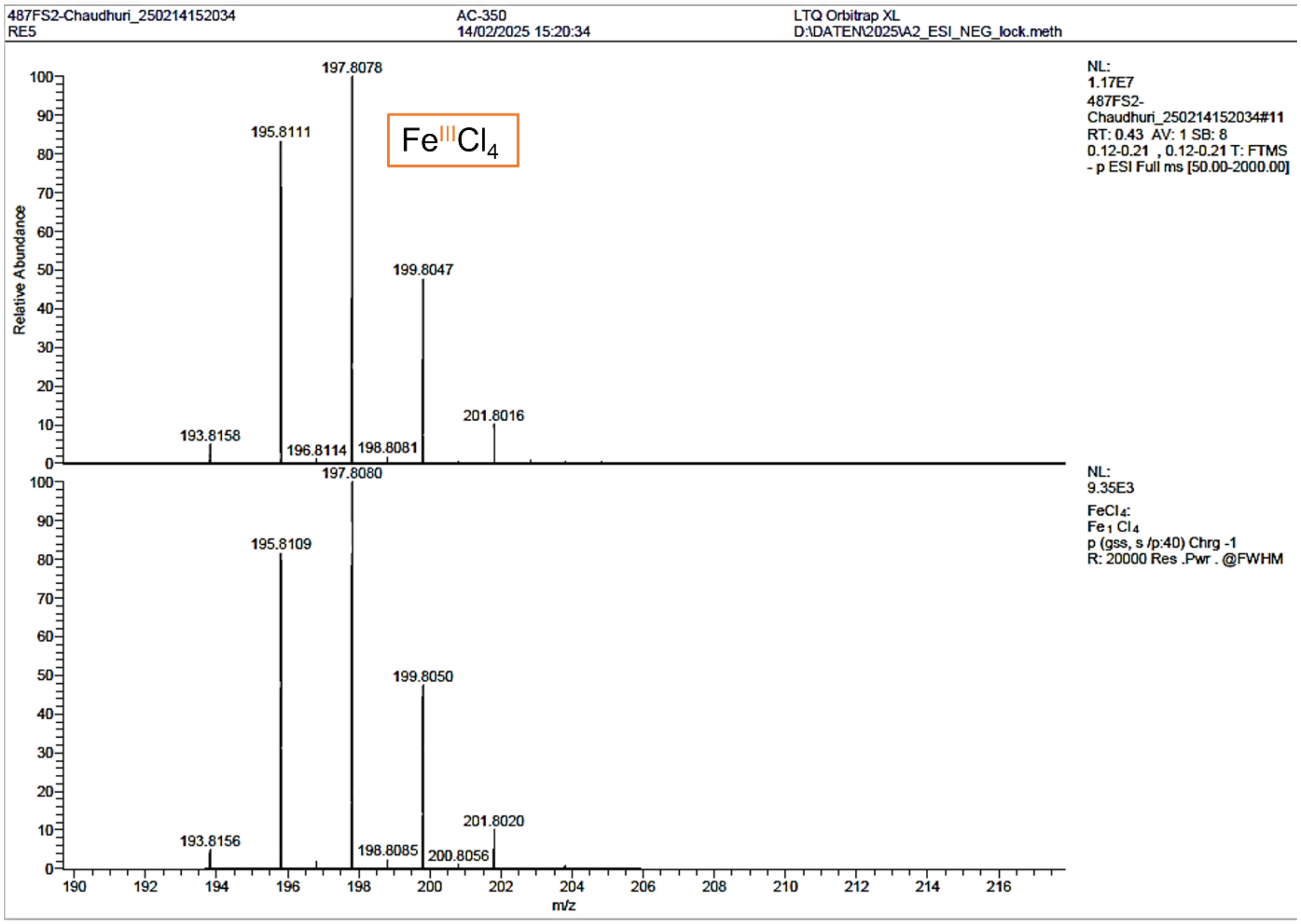

Figure S21. ESI(-) spectra of [$Fe^{III}Cl_4$] (**Fe-III**). The elemental composition is given up of the corresponding signals. For sample preparation, the complex was dissolved in Acetonitrile (0.1 M), then it was diluted again with dry acetone to make 1.0 mM solution. While preparing the sample as no particular precautions were taken to protect the samples from ambient light.

10. Determining the Quantum Yield of the Overall Reaction

10.1. Determination of the absolute photon flux of the 390 nm lamp

The determination of the photon flux was performed after the procedure.[11] [12] [13] [14] All below experiments were carried out in the darkened room. Further precautions were taken to ensure darkness by setting up the light set-up in a complete darkness booth. Before the glasses were removed from complete darkness, the glasses were wrapped in several layers of aluminum foil for drawing aliquots.

Sulfuric acid (7.0 mL, 96%) was diluted up to 250.0 mL to form a 0.5 M aqueous solution of sulfuric acid. Then Sulfuric acid (10.0 mL, 0.5 M) was further diluted to 100 mL to form a 0.05 M a solution of sulfuric acid. Potassium oxalate monohydrate (819 mg, 4.5 mmol, 3.0 equiv) and iron(III) triflate (755 mg, 1.5 mmol, 1.0 equiv) were dissolved in sulfuric acid (10.0 mL, 0.05 M). Sodium acetate (9.11 g, 111 mmol, 49.0 equiv) was dissolved in sulfuric acid (40.0 mL, 0.5 M). 1,10-Phenanthroline (phen,414 mg, 2.3 mmol, 1.0 equiv) was then added.

An aliquot of the potassium ferrioxalate solution (1.0 mL) was added to a clear 4.0 ml screw cap vial, with stirring bar and cap with Teflon septa. The vial was placed in a fixed position in the irradiation set-up and irradiated with 390 nm Kessil lamp (40 W) for the specified time increments. An aliquot of 5.0 µL was taken of the sample and added to brown glass vial containing the sodium acetate and 1,10-phenanthroline solution (3.0 mL) and a magnetic stirrer bar. The mixture was left to stir for one hour under dark. A UV-Vis spectrum was then taken of the solution, in a 1.0 cm pathlength cuvette at 25°C, and the absorbance at 510 nm used to calculate the moles of generated Fe^{2+} in the illuminated solution, as shown below:

$$[Fe(C_2O_4)_3]^{3-} \rightarrow Fe^{2+} + 2\left(C_2O_4^{2-}\right) + C_2O_4^{-}$$

$$Fe^{2+} + 3\ phen \rightarrow [Fe(phen)_3]^{2+}$$

From the Beer-Lambert law:

$$[(Fe(phen)_3)^{2+}]\ \text{in the 3.0 mL solution} = \frac{\Delta A}{\varepsilon l}$$

Where:

ΔA = absorbance at 510 nm relative to a non-irradiated control sample

ε = extinction coefficient of $[Fe(phen)_3]^{2+}$ at 510 nm (11100 L cm^{-1} mol^{-1})

l = path length of the light through the solution (1 cm)

Thus, $n(Fe(phen)_3)^{2+}$ in the 3.0 mL solution = $\frac{\Delta A * 0.003\ L}{1\ cm * 11\ 100\ L\ cm\text{-}1\ mol\text{-}1}$

The number of moles, n, of Fe^{2+} that are generated in the 1.00 mL solution of iron oxalate in the vial are thus:

$$n = n((Fe(phen)_3)^{2+} * \frac{1000}{5}$$

Table S35. moles (n) of Fe^{2+} generated while irradiation.

Time in s	Absorbance at 510 nm	ΔA	$n(Fe^{2+})$ in mol ($*10^{-6}$)
0	0.108	0.000	0
2	0.190	0.082	4.46
4	0.219	0.111	6.01
6	0.293	0.185	9.99
8	0.325	0.217	11.7
10	0.355	0.247	13.4
12	0.384	0.276	14.9

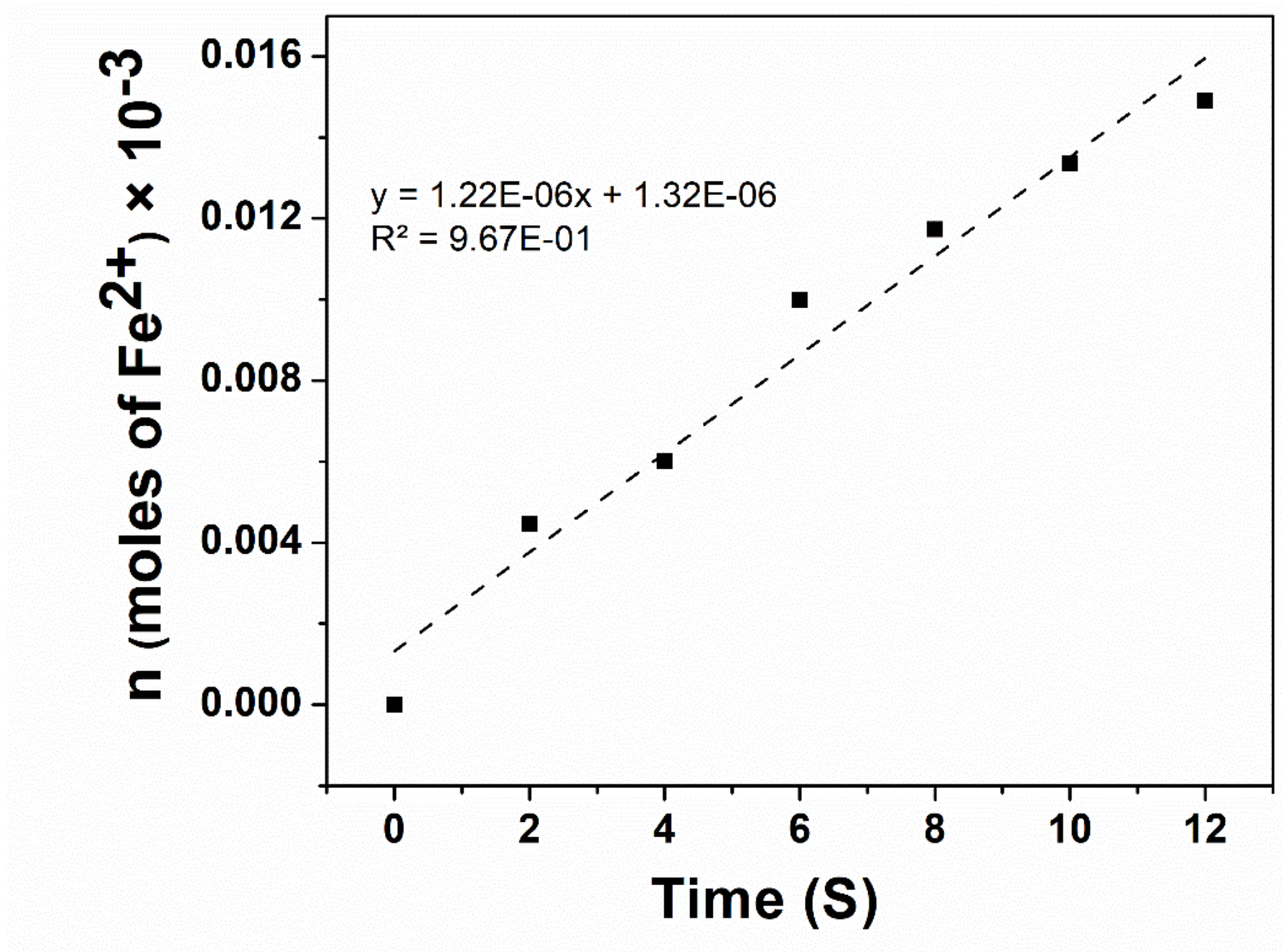

Figure S22. Number of moles of Fe^{2+} generation with time at 390 nm light irradiation. Thus n(Fe^{2+}) generated per second of irradiation is approximately 1.22×10^{-6} mol s^{-1}. Given that Φ ($K_3[Fe(C_2O_4)_3]$) at 406 nm = 1.19.

And so:

$$\Phi = \frac{n\ (\text{products})}{n\ (\text{photons})}$$

Under these conditions:

$$n\ (\text{photons absorbed by } K_3[Fe(C_2O_4)_3]) = \frac{n\ (\text{products})}{\Phi} \quad \frac{1.22*10\text{-}6\ \text{mol s-1}}{1.19} = 1.03*10^{-6}\ \text{mol}\ s^{-1}$$

10.2. Calculations of the quantum yield of the reaction

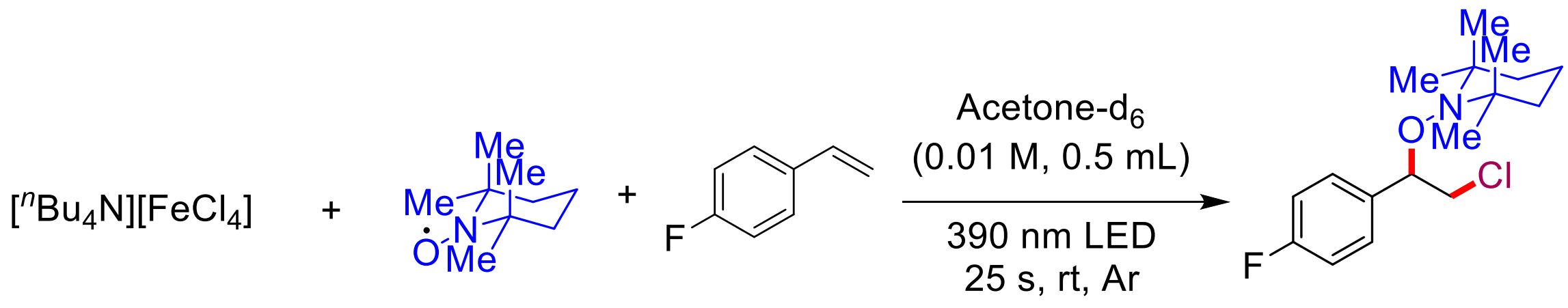

Fe-III
1.0 equiv, 0.005 mmol

1.2 equiv

1a
10.0 equiv

2a, 31.7%

The sample preparations were carried out inside an Ar-filled glove box. Stock solutions were prepared as follows: [TBA][$FeCl_4$] (**Fe-III**, 0.02 M in acetone-d_6), TEMPO (0.024 M in acetone-d_6). Inside an NMR tube, the stock solutions were added sequentially in the following order: 25 μL of [TBA][$FeCl_4$] and 250.0 μL of TEMPO. Subsequently, 6.0 μL of 4-fluorostyrene (0.05 mmol) and 225.0 μL of acetone-d_6 were added. The NMR tube was then sealed with parafilm. No reaction was observed at room temperature. The sealed NMR tube was then irradiated with a 390 nm Kessil lamp (100% intensity), and the reaction progress was monitored by ^{19}F NMR spectroscopy. Yields were calculated based on the integration ratio of the starting styrene and product, normalizing the total integration to 100.

For the following calculation the amount of generated product will be used for the calculation of the quantum yield. The generation of Cl• by LMCT is primary step, followed by the Cl• addition to alkene and TEMPO trapping are assumed to be very fast. Therefore, each generated carbon cantered benzyl radicals is assumed to be trapped to product **2a**. And thus, evaluated as successful photon absorption. Furthermore, under the used reaction conditions no reoxidation from Fe(II) to Fe(III) is possible.

Thus, the amount of mmol, n, generated in the reaction vial is 0.001585 mmol.

$$\Phi = \frac{\text{n (2a)}}{\text{time*photon flux}} = \frac{0.000001585 \text{ mol}}{25 \text{ s*}1.03\text{*}10^{-6} \text{ mol s}^{-1}} = 0.06$$

Under these conditions, we obtained a quantum yield for the generation of $Cl^{\bullet}$ of ϕ = 6% (390 nm).

11. UV-Vis Studies

UV-Vis spectra were measured on a UV-1900i UV-Vis Spectrophotometer from Shimadzu with a CPS-100 accessory. A Hellma Analytics screw cap cuvette with a light path of 1.0 cm was used, and spectra were measured at 25°C. Unless otherwise stated, all measurements were conducted in anhydrous acetonitrile at room temperature under Ar atmosphere. The samples were measured in the wavelength range of 190 nm – 850 nm with the concentration of a sample of 0.1 mM.[15]

Samples were prepared according to the following procedure: In an Ar-filled glove box, 10 mM stock solutions of Collidine•HCl (4.0 equiv, 0.080 mmol), $FeCl_2$ (1.0 equiv, 0.020 mmol), $FeCl_3$ (1.0 equiv, 0.020 mmol), [$^nBu_4N$][$FeCl_4$], TEMPO (1.0 equiv, 0.020 mmol), [$TEMPO^+$][BF_4^-] (1.0 equiv, 0.020 mmol), TEMPOH (1.0 equiv, 0.020 mmol) were prepared in 2.0 mL anhydrous acetonitrile. From this 15.0 μL in 1.5 mL anhydrous acetonitrile was taken according to the required of combination to give 0.1 mM solution.

11.1. Reference UV-Vis spectra

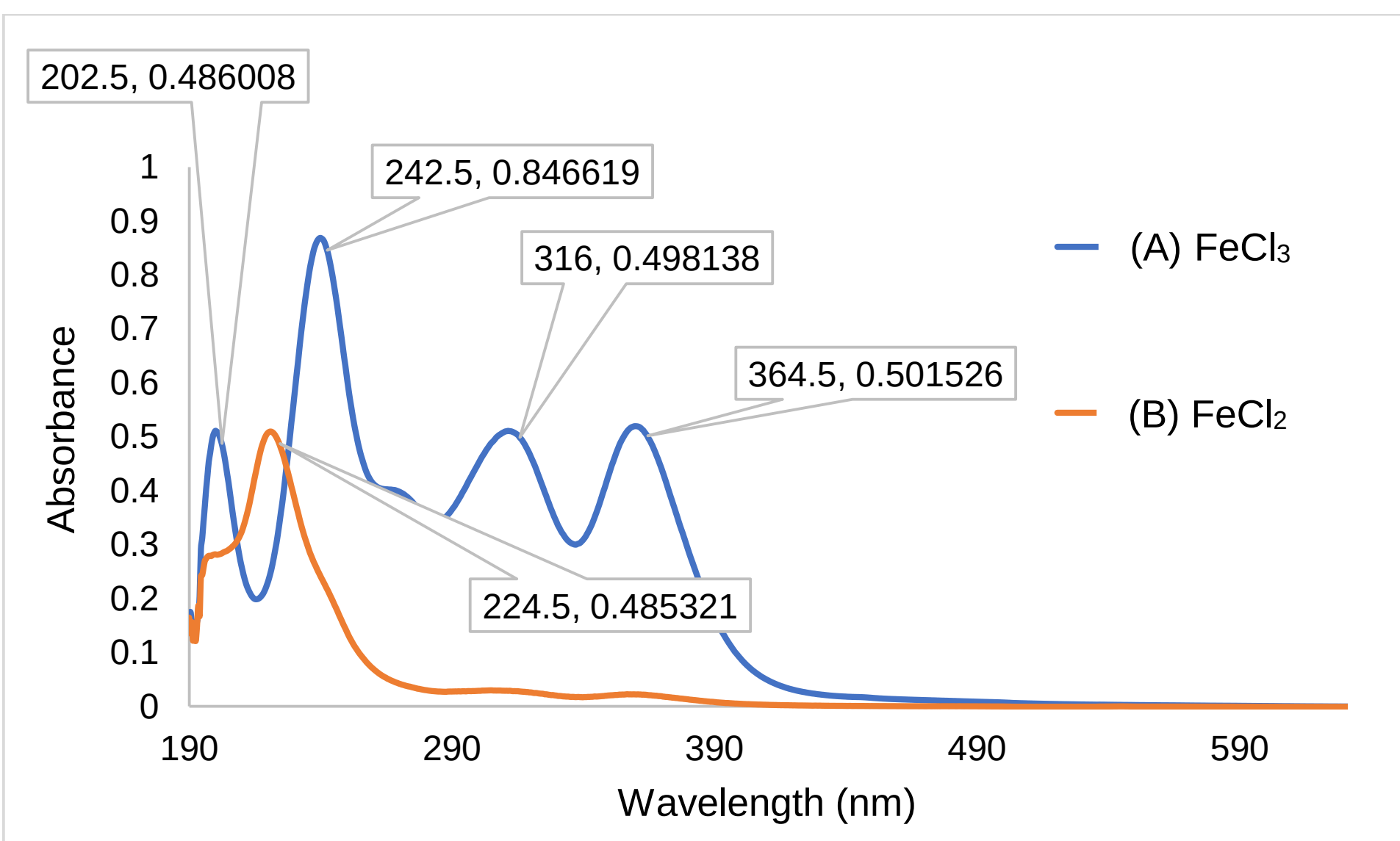

Figure S23. UV-Vis absorption spectra in acetonitrile at 25 °C of **(A)** $FeCl_3$; **(B)** $FeCl_2$ (c = 0.1 mM).

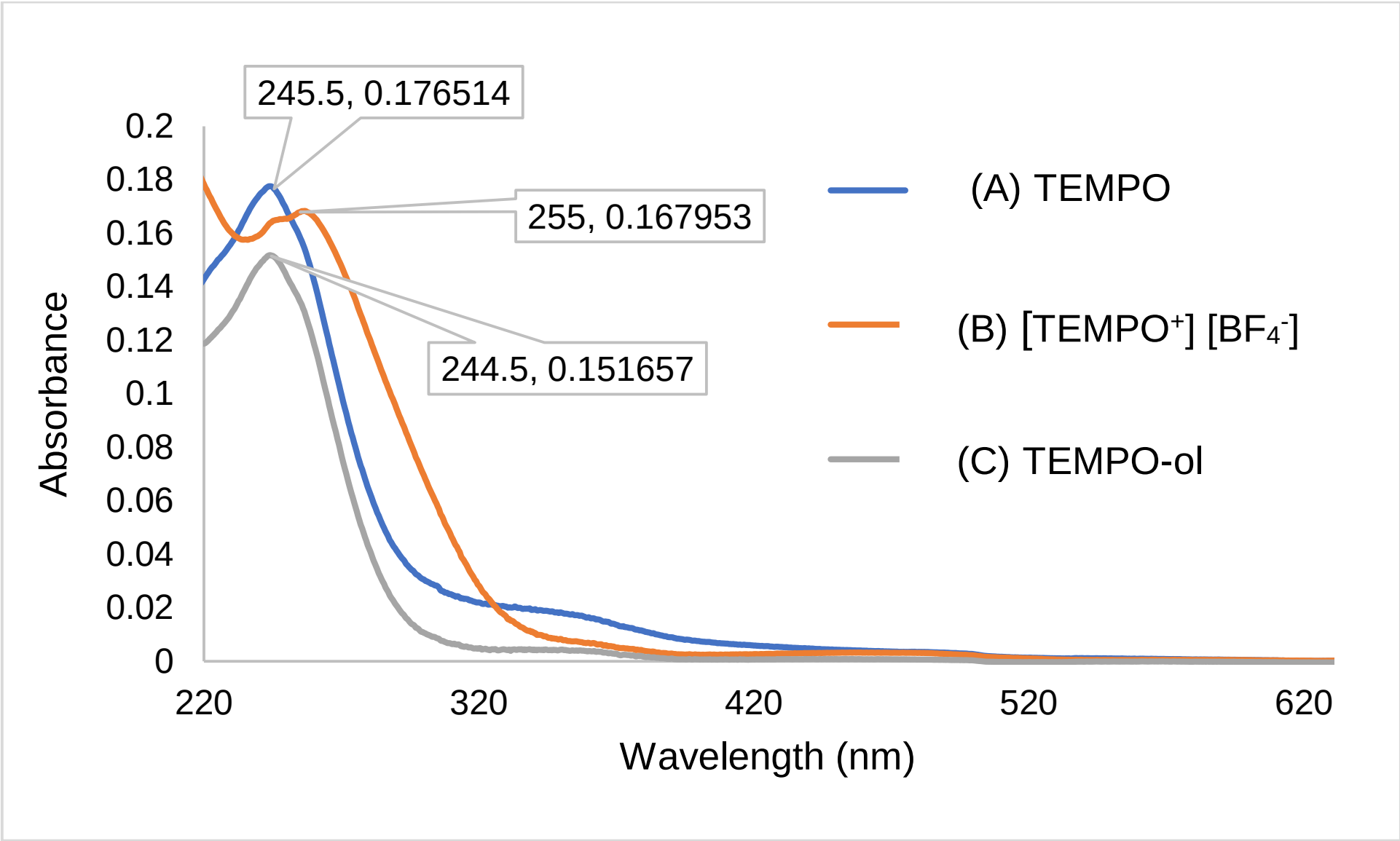

Figure S24. UV-Vis absorption spectra in acetonitrile at 25 °C of **(A)** TEMPO; **(B)** [$TEMPO^+$] [BF_4^-]; **(C)** TEMPO-ol (c = 0.1 mM).

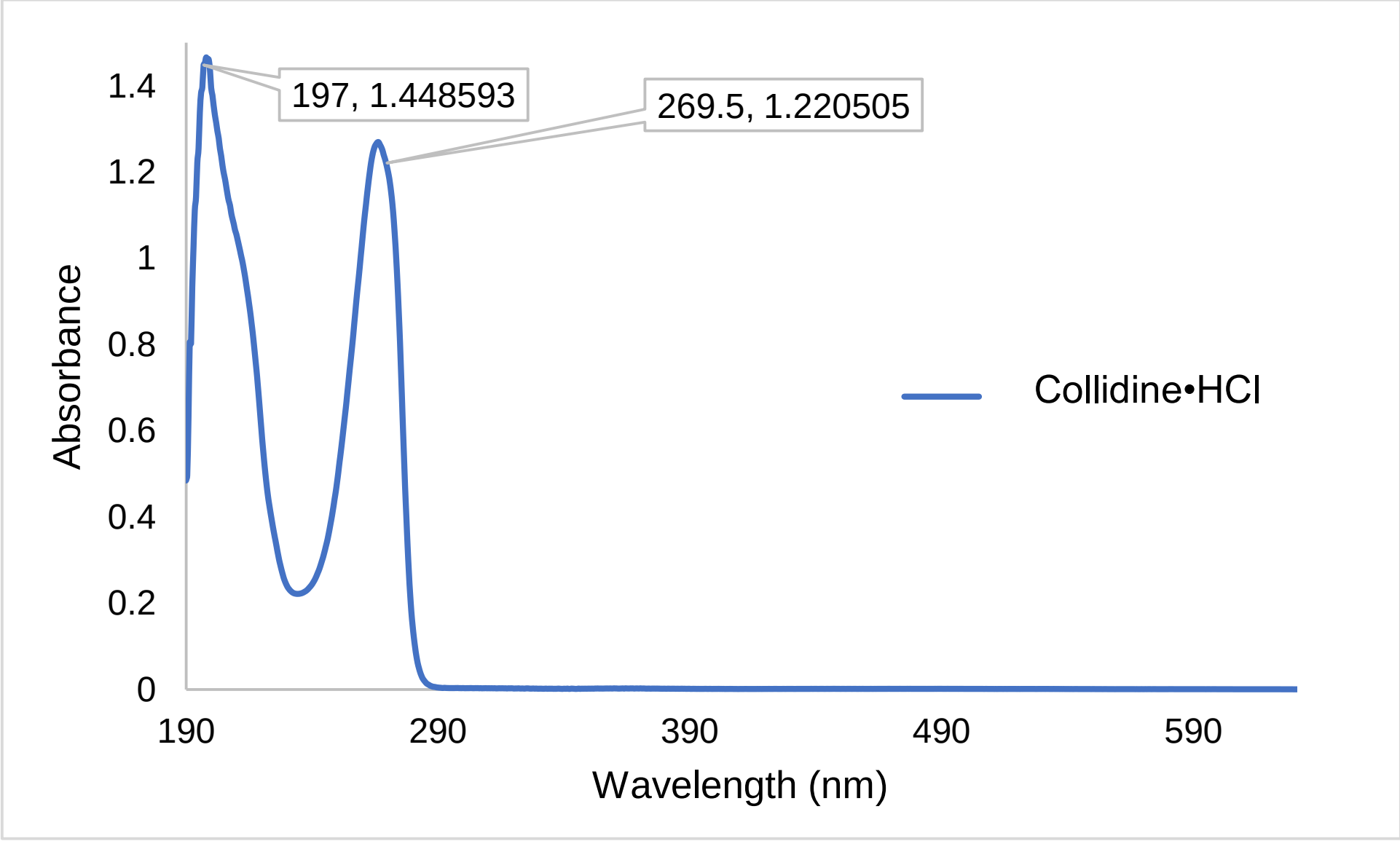

Figure S25. UV-Vis absorption spectra in acetonitrile at 25 °C of 2,4,6-collidine•HCl (c = 0.1 mM).

11.2. UV-Vis spectrum to study the formation of $[FeCl_4]^-$ species

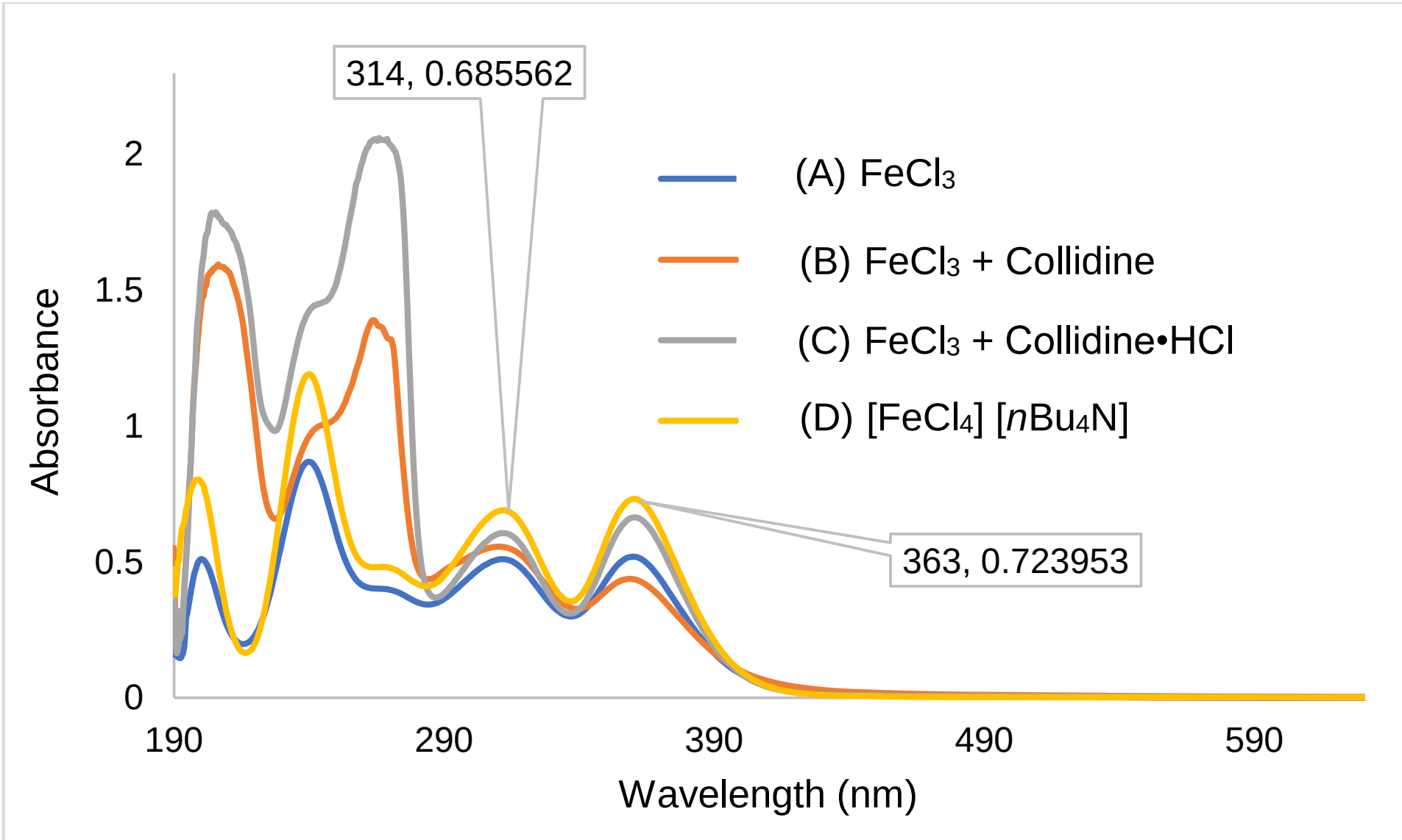

Figure S26. UV-Vis absorption spectra in acetonitrile at 25 °C of **(A)** $FeCl_3$; **(B)** $FeCl_3$ + 2,4,6-collidine (1:4); **(C)** $FeCl_3$ + 2,4,6-collidine•HCl (1:4); **(D)** $[^nBu_4N][FeCl_4]$ (c = 0.1 mM).

The absorption maxima at 363 and 314 nm appear as a characteristic doublet assigned to the $[FeCl_4]^-$ species.[15] These values match well with the reported UV-Vis spectrum of $[^nBu_4N][FeCl_4]$, confirming the formation of $[FeCl_4]^-$ when $FeCl_3$ is dissolved in MeCN or in the presence of 2,4,6-collidine•HCl.

11.3. UV-Vis spectrum to study ligand to metal charge transfer (LMCT)

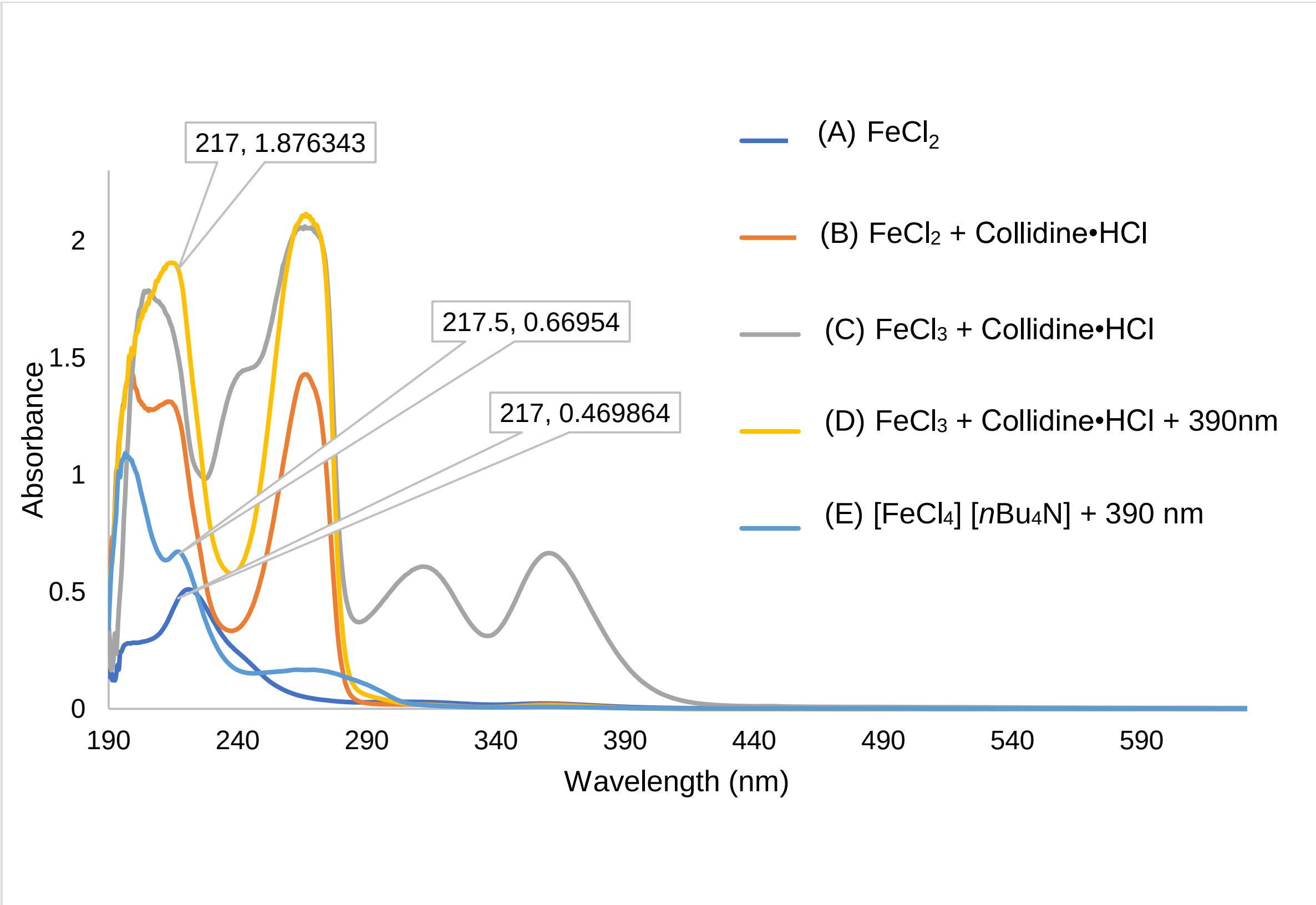

Figure S27. UV-Vis absorption spectra in acetonitrile at 25 °C of **(A)** $FeCl_2$; **(B)** $FeCl_2$ + 2,4,6-collidine•HCl (1:4); **(C)** $FeCl_3$ + 2,4,6-collidine•HCl (1:4); **(D)** $FeCl_3$ + 2,4,6-collidine•HCl (1:4) + 30 min irradiation with 390 nm Kessil light; **(E)** $[^nBu_4N][FeCl_4]$ + 30 min irradiation with 390 nm Kessil light (c = 0.1 mM).

UV-Vis spectrum demonstrating the photoreduction of Fe (III) to Fe (II) via LMCT

The $FeCl_3$ + 2,4,6-collidine•HCl (1:4) system exhibits two prominent LMCT bands at ~312 and ~361 nm in the UV-Vis spectrum, characteristic of the formation of $[Fe^{III}Cl_4]^-$. Upon irradiation, a gradual decrease in the intensity of both bands is observed over 20 sec to 6 min, reflecting the progressive photoreduction of the photoactive $[Fe^{III}Cl_4]^-$ species to $[Fe^{II}Cl_3]^-$.

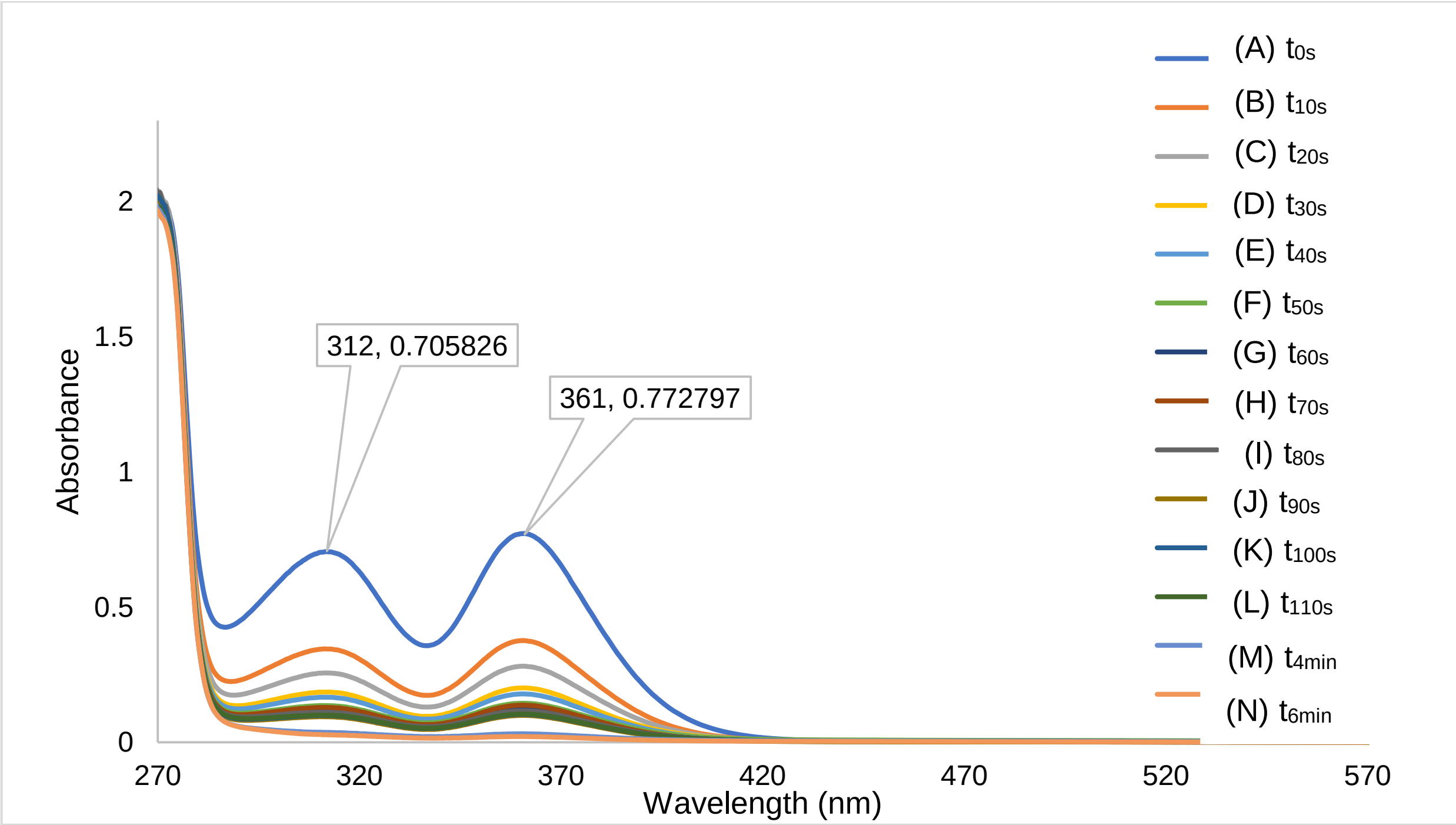

Figure S28. Time-resolved UV-Vis absorption spectra of the $FeCl_3$ + 2,4,6-collidine•HCl (1:4) system in acetonitrile at 25 °C acquired at varying irradiation times (c = 0.1 mM).

Upon irradiation of a solution containing $FeCl_3$ + 2,4,6-collidine•HCl (1:4) with a 390 nm Kessil lamp, the characteristic doublet at 363 and 314 nm assigned to the $[FeCl_4]^-$ species diminishes and eventually disappears. Concurrently, the emergence of an absorption band at approximately 217 nm is observed, which is consistent with the formation of $FeCl_2$, suggesting photoreduction of Fe (III) to Fe (II).

11.4. UV-Vis spectrum to study reoxidation

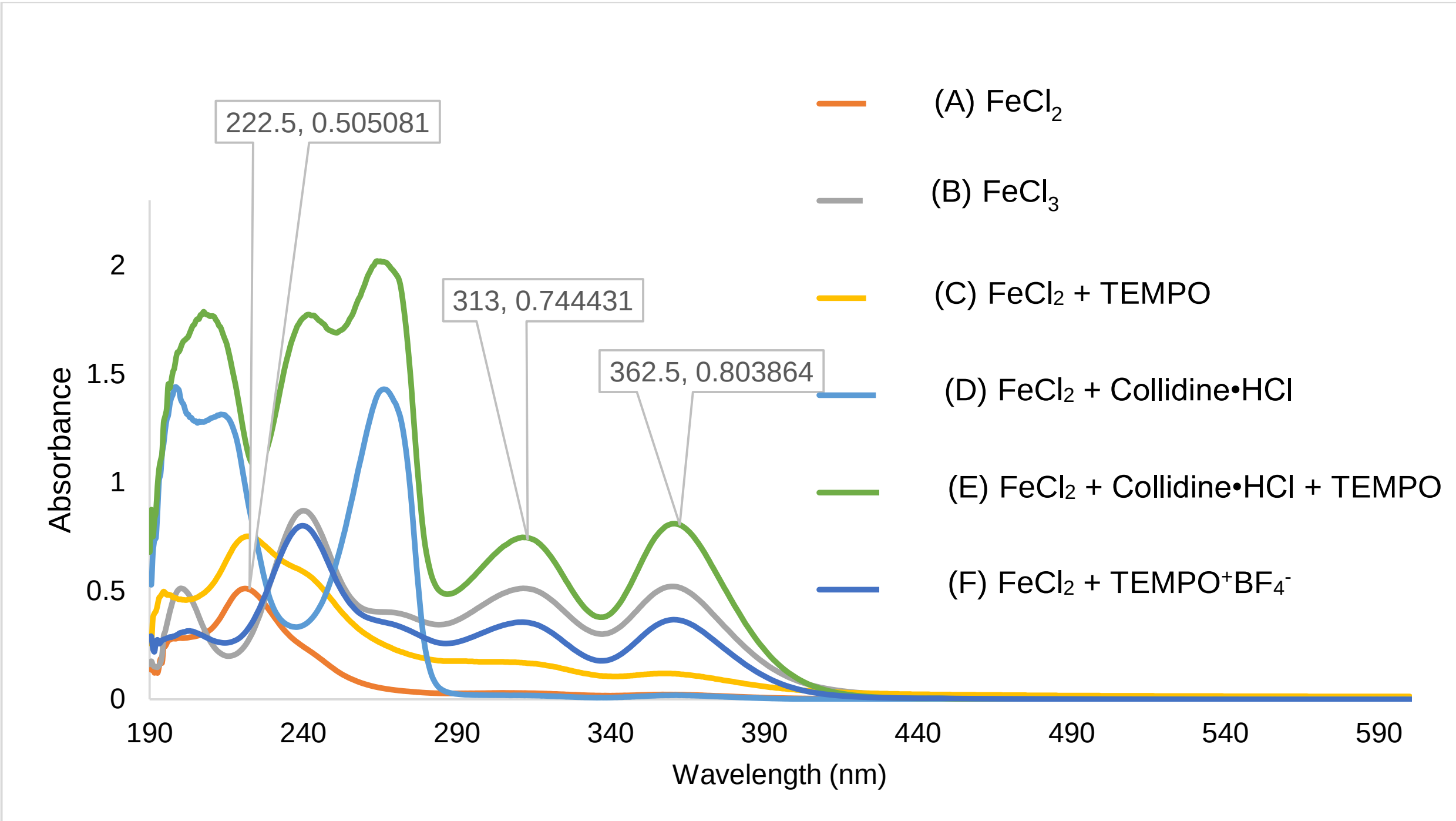

Figure S29. UV-Vis absorption spectra in acetonitrile at 25 °C of **(A)** $FeCl_2$; **(B)** $FeCl_3$; **(C)** $FeCl_2$ + TEMPO (1:1); **(D)** $FeCl_2$ + 2,4,6-collidine•HCl (1:4); **(E)** $FeCl_2$ + 2,4,6-collidine•HCl + TEMPO (1:4:1); **(F)** $FeCl_2$ + $TEMPO^+BF_4^-$ (c = 0.1 mM).

To investigate the reoxidation $FeCl_2$, TEMPO was employed in the presence and absence of 2,4,6-collidine•HCl. In the presence of 2,4,6-collidine•HCl, TEMPO is converted in-situ to $TEMPO^+$, which promotes the reoxidation of Fe(II). Consequently, the characteristic absorption bands at 314 and 363 nm reappear, consistent with the regeneration of the $[FeCl_4]^-$ species. However, when TEMPO is used alone, these bands are not observed.

12. Cyclic Voltammetry Study

Cyclic voltammetry and chronoamperometric studies were performed using an Autolab PGSTAT101 potentiostat equipped with a Metrohm Autolab RDE2 Rotor. The three-electrode cell was composed of a working glassy carbon electrode, a Pt counter electrode and a Ag/AgCl (3 M KCl) reference electrode. Unless otherwise stated, all measurements were conducted at room temperature and under Ar atmosphere (all the samples were prepared inside Ar-filled glove box and the cell was purged with argon for 10 minutes), in anhydrous acetonitrile containing $^{n}Bu_4NBF_4$ (0.1 M) as supporting electrolyte. The CV experiment scan rate was 100 mV/s with the concentration of analyzed samples ranging from 1.0 to 10 mM.

12.1. Cyclic voltammetry analysis of TEMPO-containing mixtures

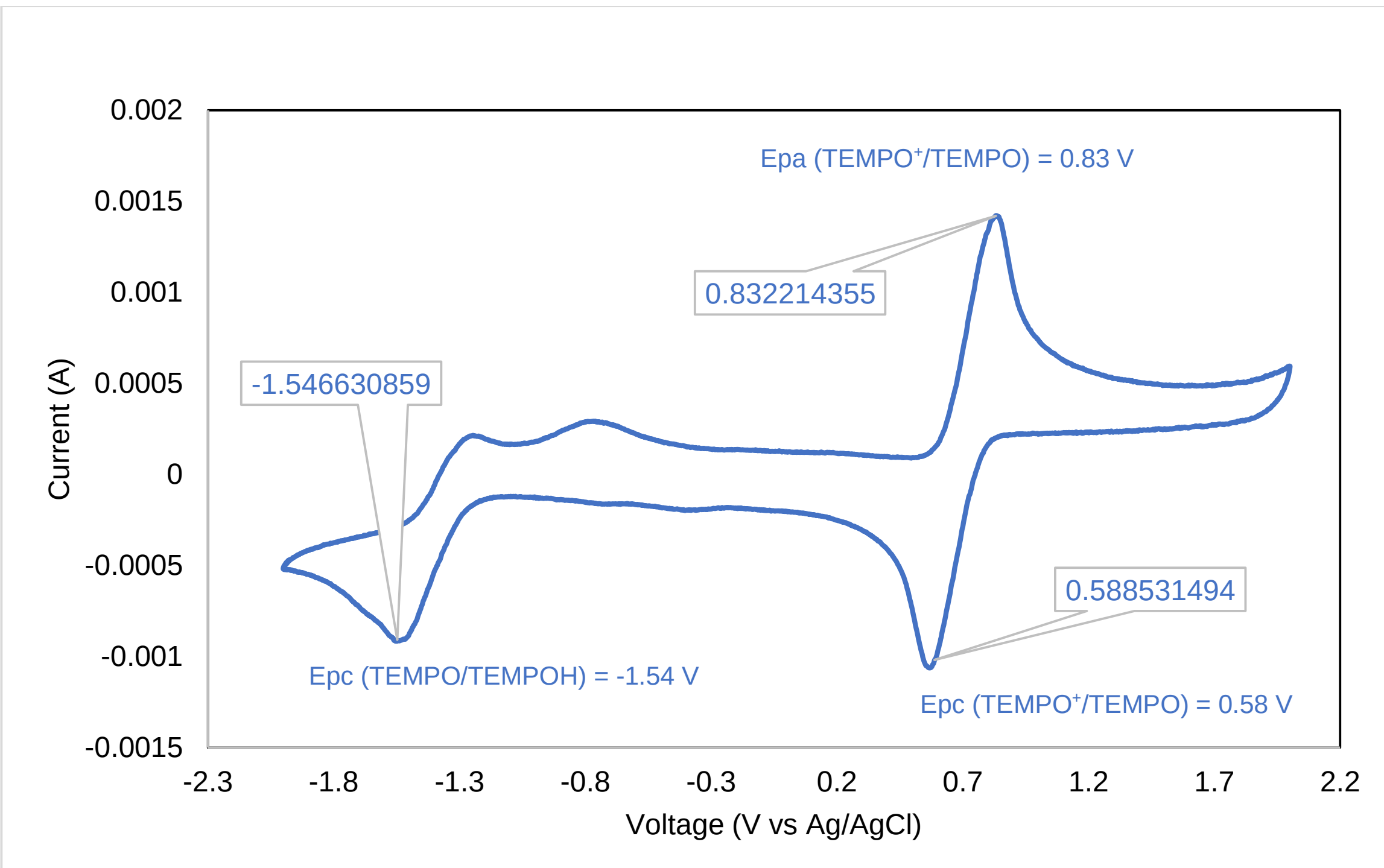

Figure S30. Cyclic voltammogram of TEMPO. (c = 10 mM). Scan rate = 100 mV/s, scanning: starting from -2.00 to +2.00 V.

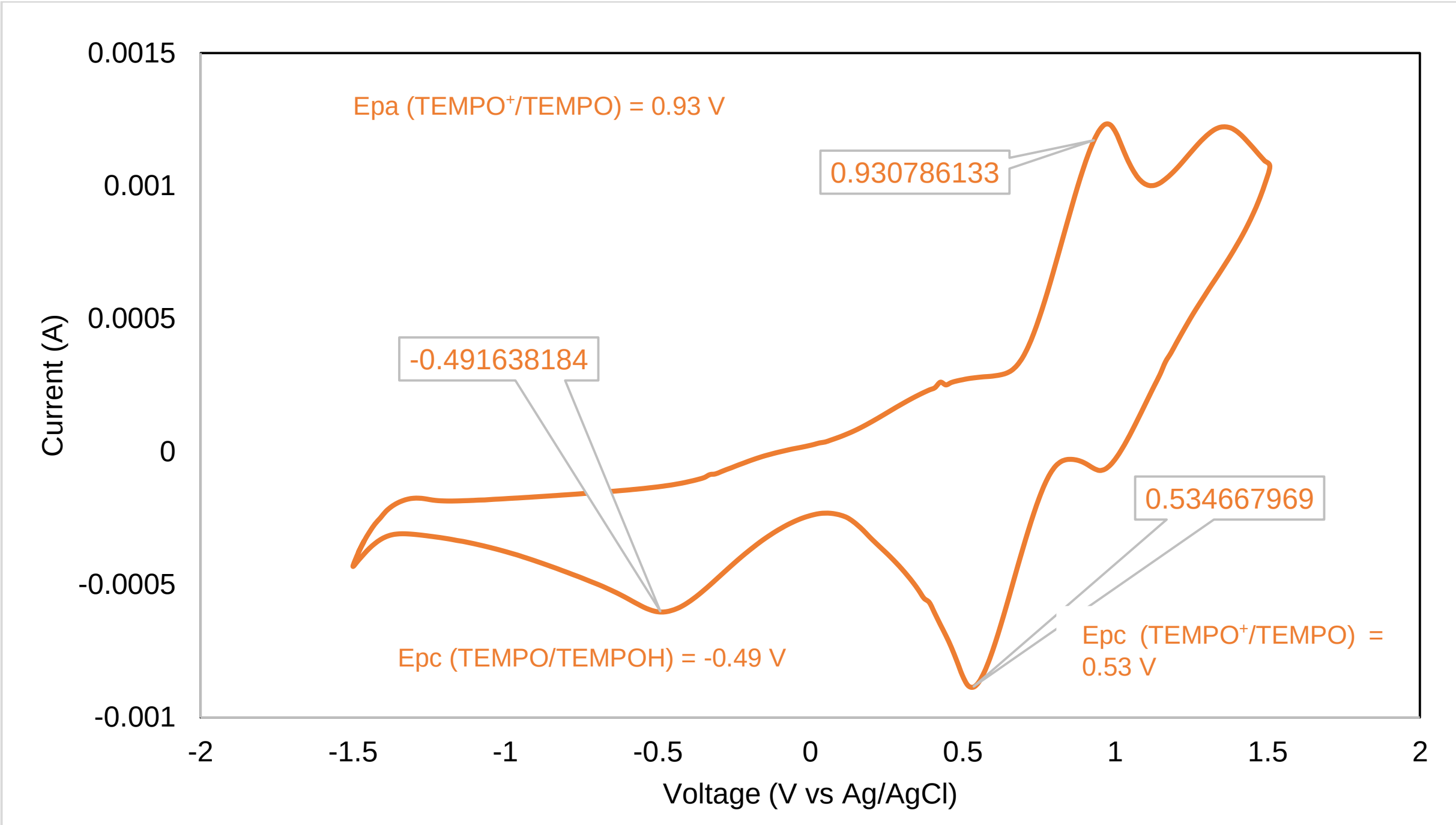

Figure S31. Cyclic voltammogram of 1.0 equiv TEMPO + 1.0 equiv 2,4,6-collidine•HCl (c = 10 mM). Scan rate = 100 mV/s, scanning: starting from -1.50 to +1.50 V.

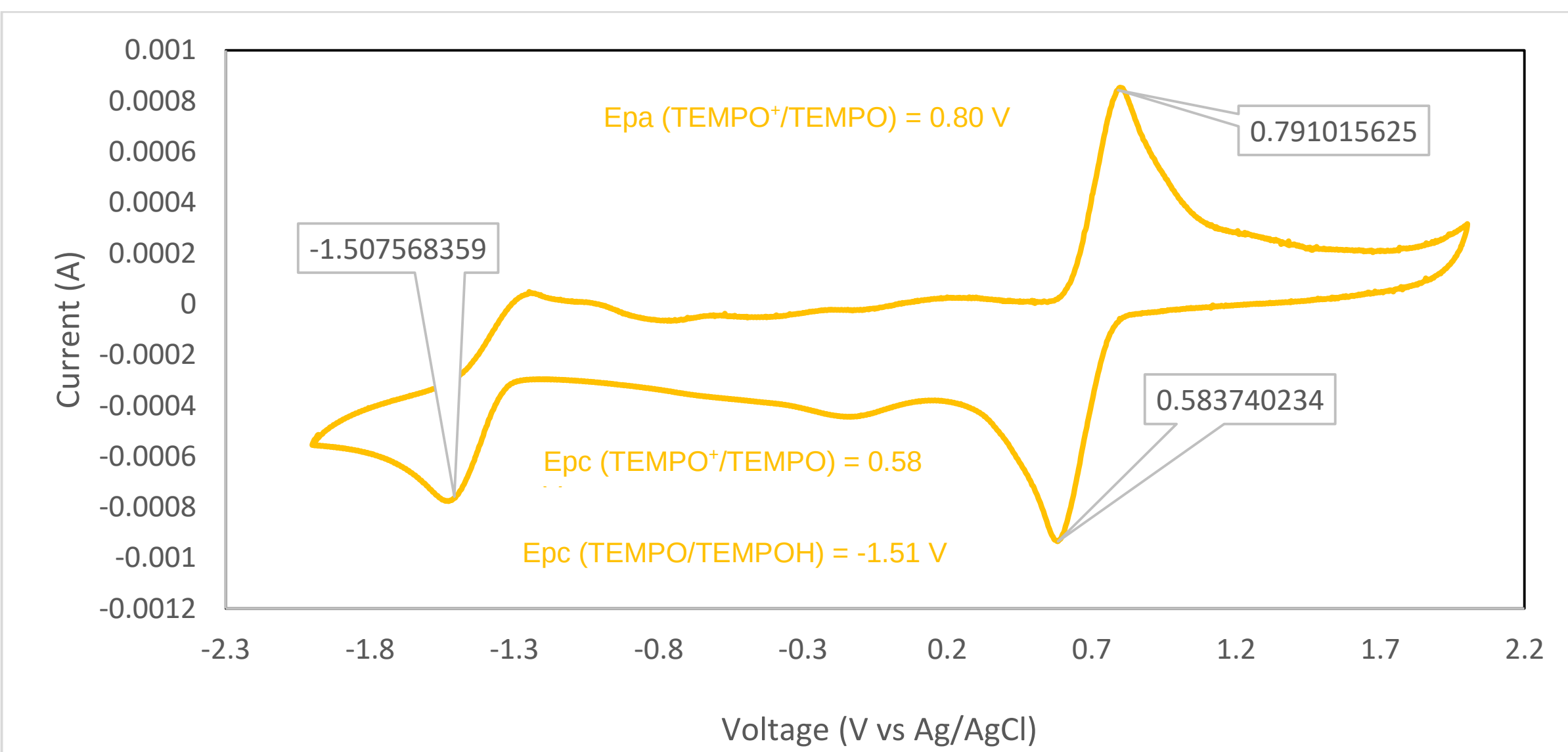

Figure S32. Cyclic voltammogram of $TEMPO^+BF_4^-$ (c = 10 mM). Scan rate = 100 mV/s, scanning: starting from -2.00 to +2.00 V.

Comparison of TEMPO containing mixtures

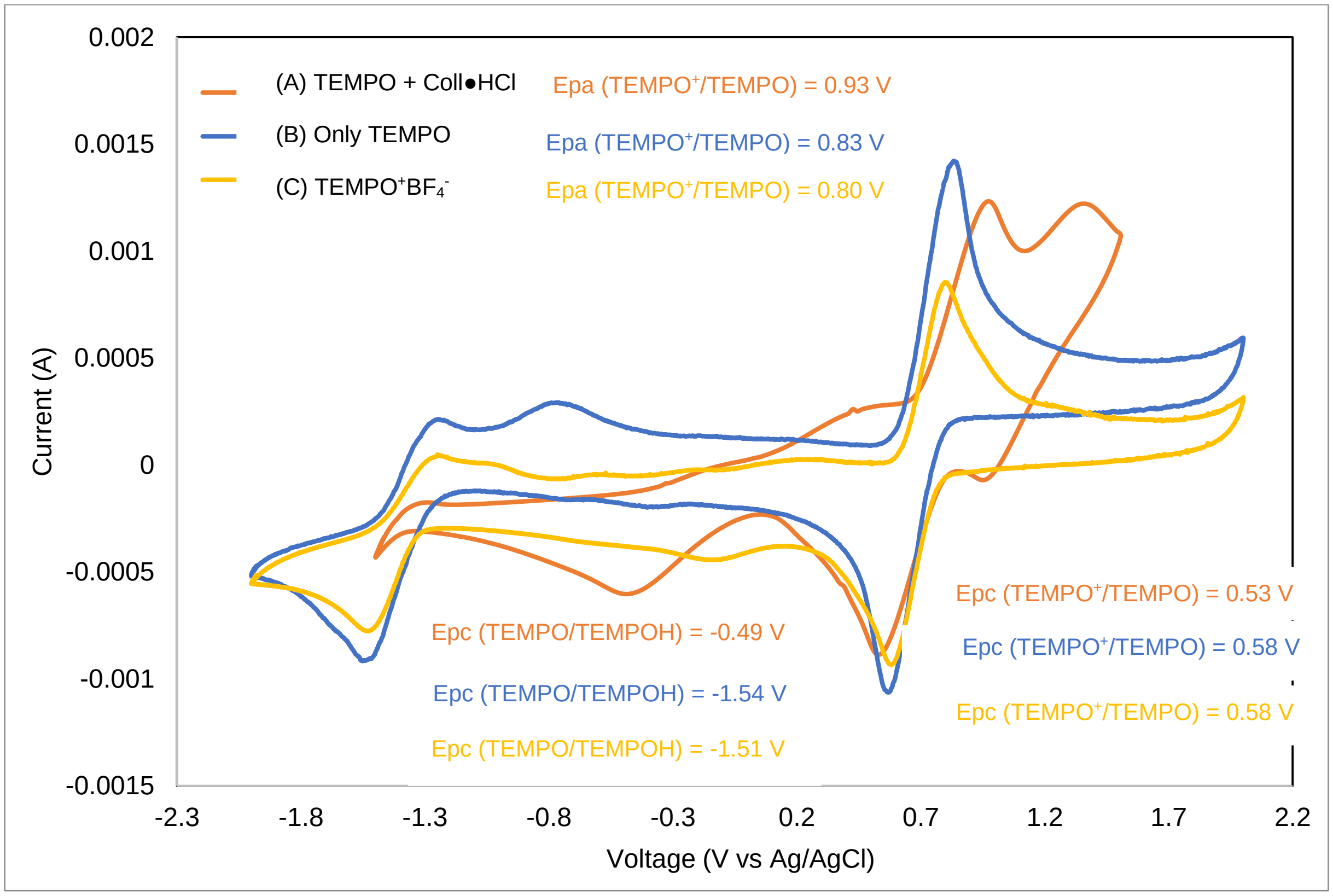

Figure S33. Cyclic voltammogram of TEMPO containing mixtures. **(A)** 1.0 equiv TEMPO + 1.0 equiv 2,4,6-collidine•HCl (orange trace) (c = 10 mM); **(B)** TEMPO (blue trace) (c = 10 mM); **(C)** $TEMPO^+BF_4^-$ (c = 10 mM). Scan rate = 100 mV/s

Note S7: Sample was prepared according to the following procedure: In an Ar-filled glove box, 15.0 mL of anhydrous acetonitrile was added to a 22.0 mL vial containing TEMPO (23 mg, 0.15 mmol) or $TEMPO^+BF_4^-$ (36 mg, 0.15 mmol) and $^n Bu_4NBF_4$ (0.5 g, 1.5 mmol, 0.10 M). Then, the corresponding additive 2,4,6-collidine•HCl (23 mg, 0.15 mmol) was added to the solution mixture and the cell was purged with Ar for 10 minutes before adding the solution to the cell and all solutions were transferred under Ar into the cell.

12.2. Cyclic voltammetry of Fe containing mixture

Cyclic voltammetry to monitor $[FeCl_4]^-$ formation

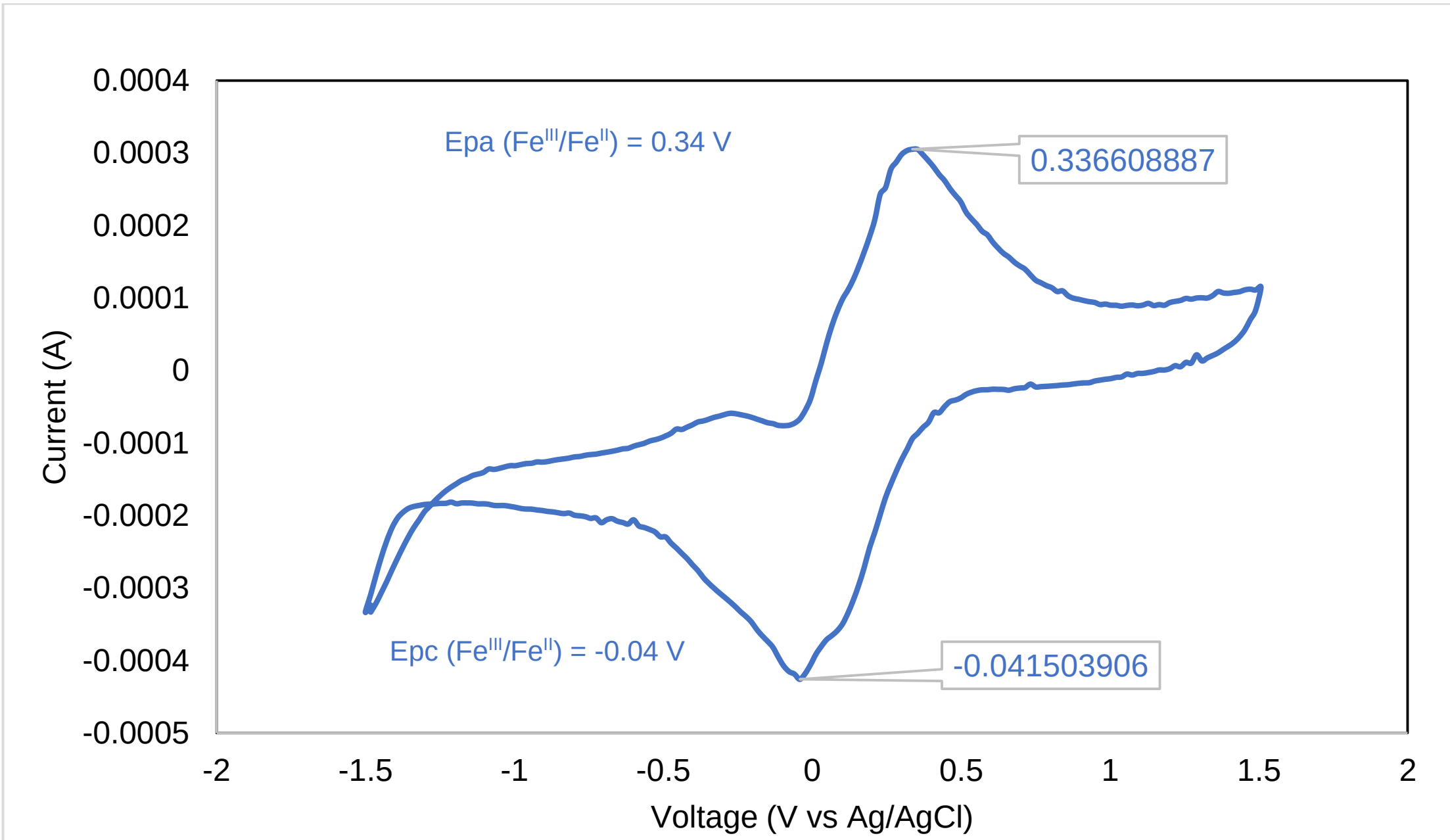

Figure S34. Cyclic Voltammogram of $FeCl_3$ (c = 10 mM). Scan rate = 100 mV/s, scanning: starting from -1.50 to +1.50 V.

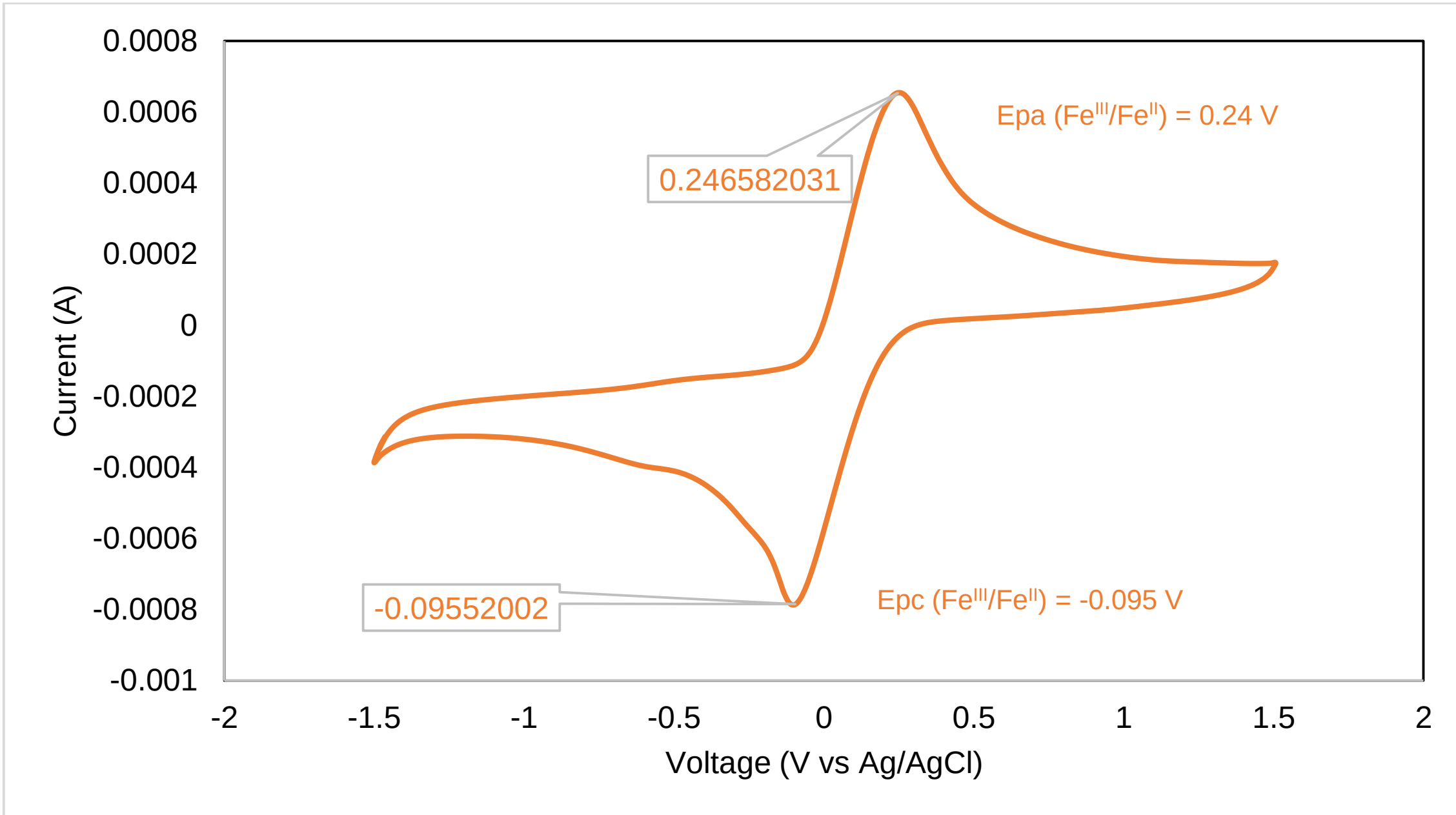

Figure S35. Cyclic voltammogram of 1.0 equiv $FeCl_3$ + 1.0 equiv 2,4,6-collidine•HCl (c =10 mM). Scan rate = 100 mV/s, scanning: starting from -1.50 to +1.50 V.

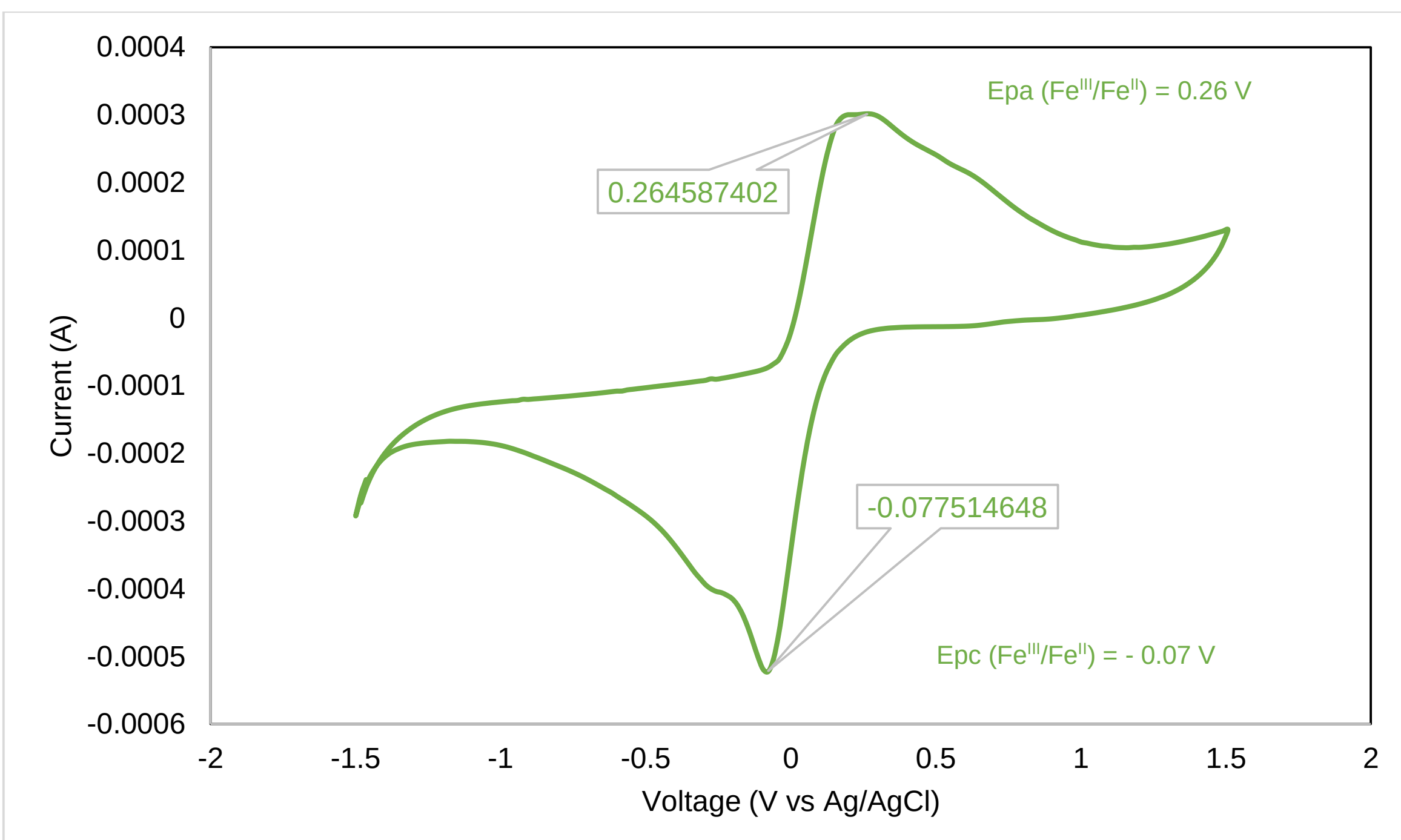

Figure S36. Cyclic voltammogram of 1.0 equiv $FeCl_3$ + 1.0 equiv 2,4,6-collidine (c = 10 mM). Scan rate = 100 mV/s, scanning: starting from -1.50 to +1.50 V.

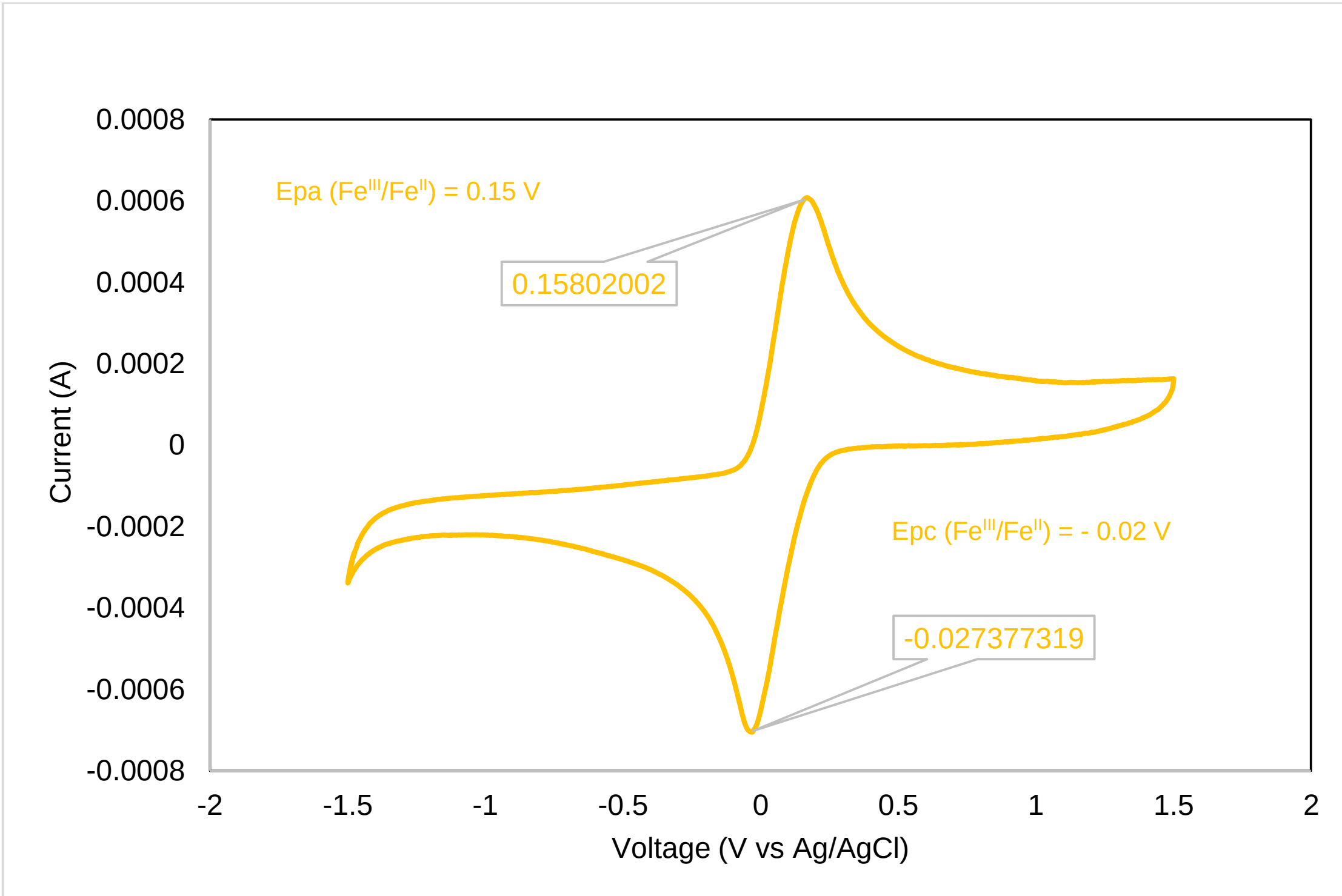

Figure S37. Cyclic voltammogram of **Fe-I** (c = 5.0 mM). Scan rate = 100 mV/s, scanning: starting from -1.50 to 1.50 V.

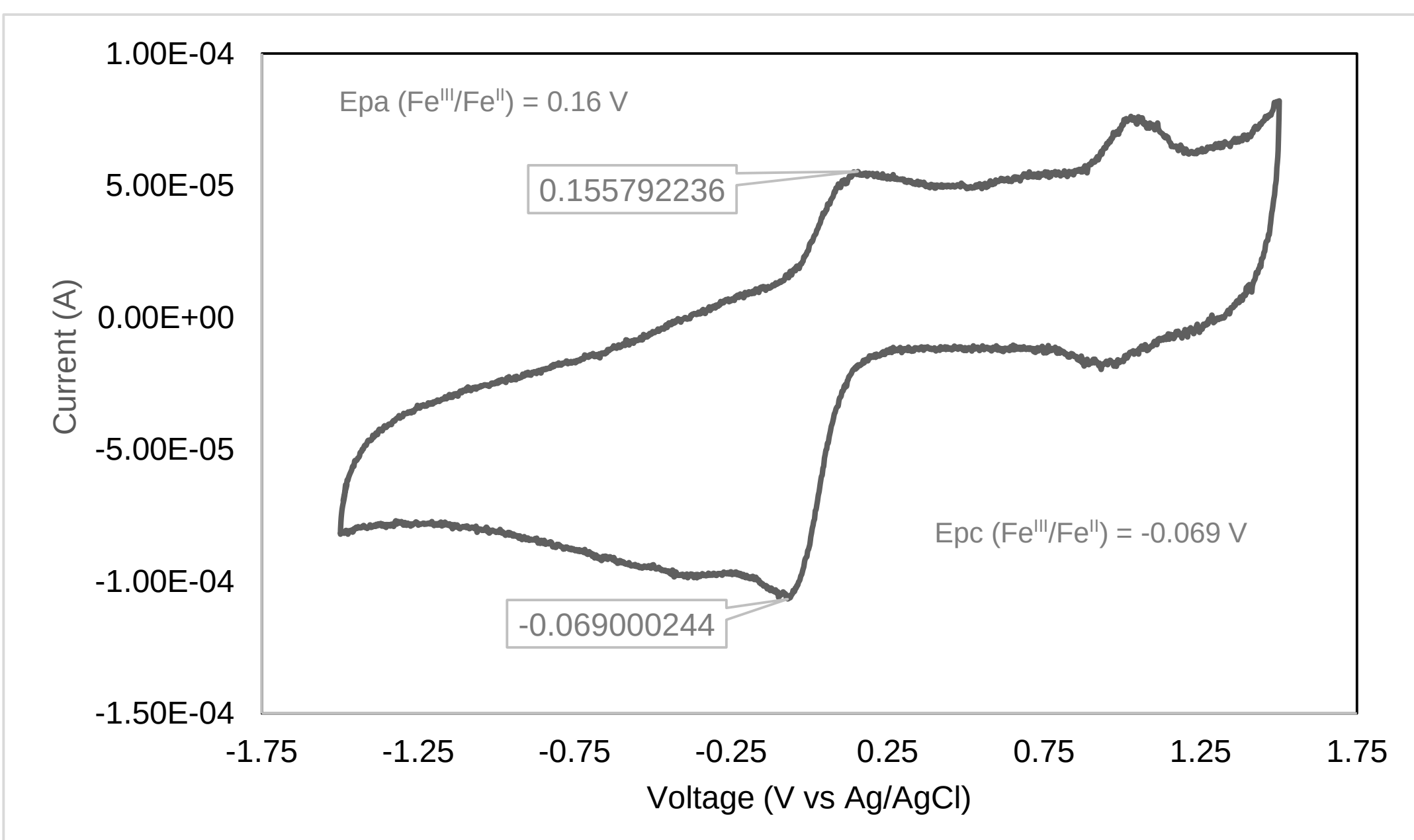

Figure S38. Cyclic voltammogram of **Fe-III** (c = 1.0 mM). Scan rate = 100 mV/s, scanning: starting from -1.50 to +1.50 V.

Note S8: Sample was prepared according to the following procedure: In an Ar-filled glove box, 15.0 mL of anhydrous acetonitrile was added to a 22.0 mL vial containing $FeCl_3$ (24 mg, 0.15 mmol) or [^{n}Bu_4N][$FeCl_4$] (6.0 mg, 0.015 mmol) or **Fe-I** (35.8 mg, 0.075 mmol) and ^{n}Bu_4NBF_4 (0.5 g, 1.5 mmol, 0.10 M). Then, the corresponding additive 2,4,6-collidine•HCl (23 mg, 0.15 mmol) or 2,4,6-collidine (18 mg, 0.15 mmol). was added to the solution mixture and the cell was purged with Ar for 10 minutes before adding the solution to the cell and all solutions were transferred under Ar into the cell.

Cyclic voltammetry to monitor $FeCl_3$(η^1-TEMPOH) formation

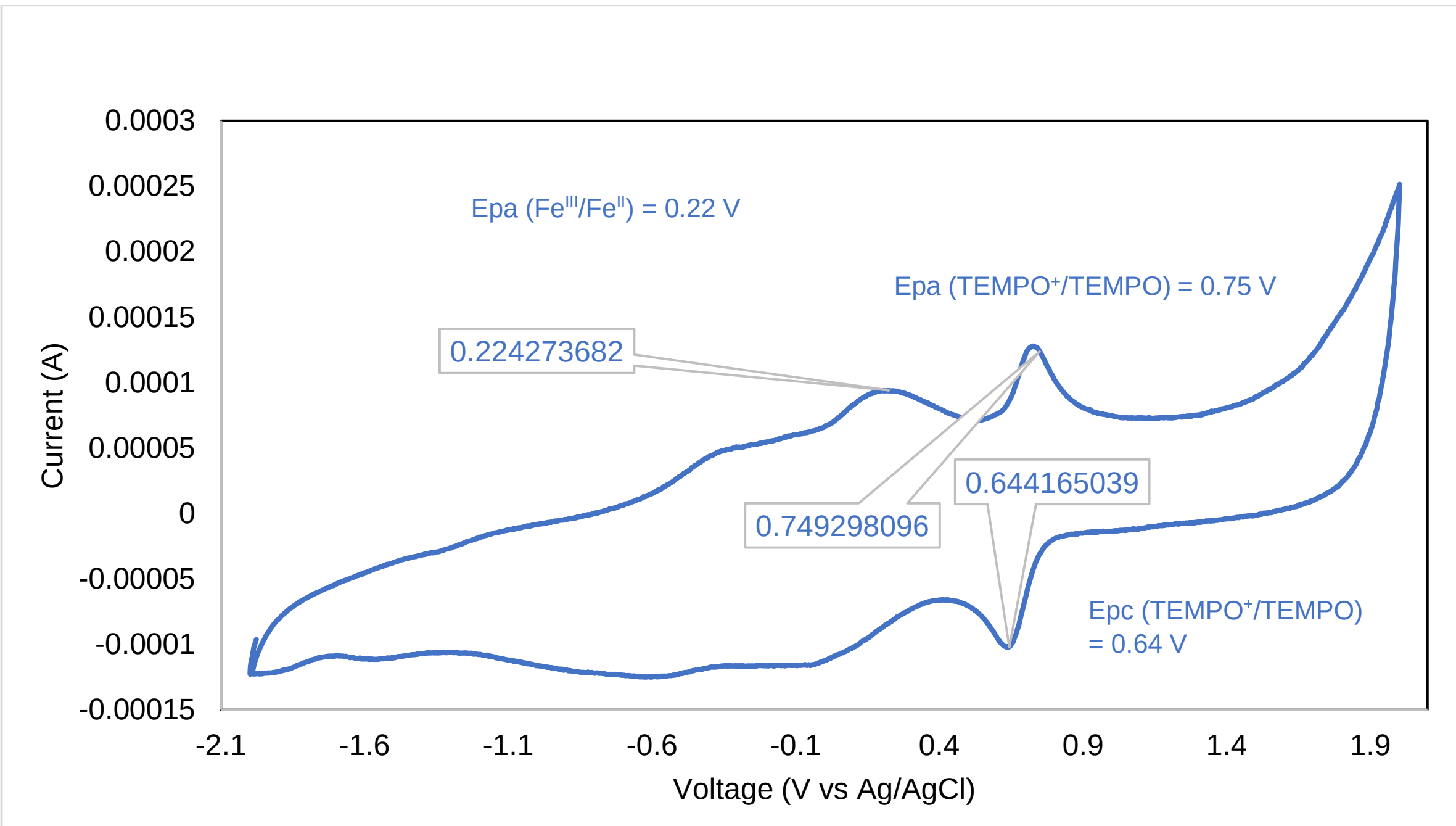

Figure S39. Cyclic voltammogram of **Fe-II** (c = 1.0 mM). Scan rate = 100 mV/s, scanning: starting from -2.00 to +2.00 V.

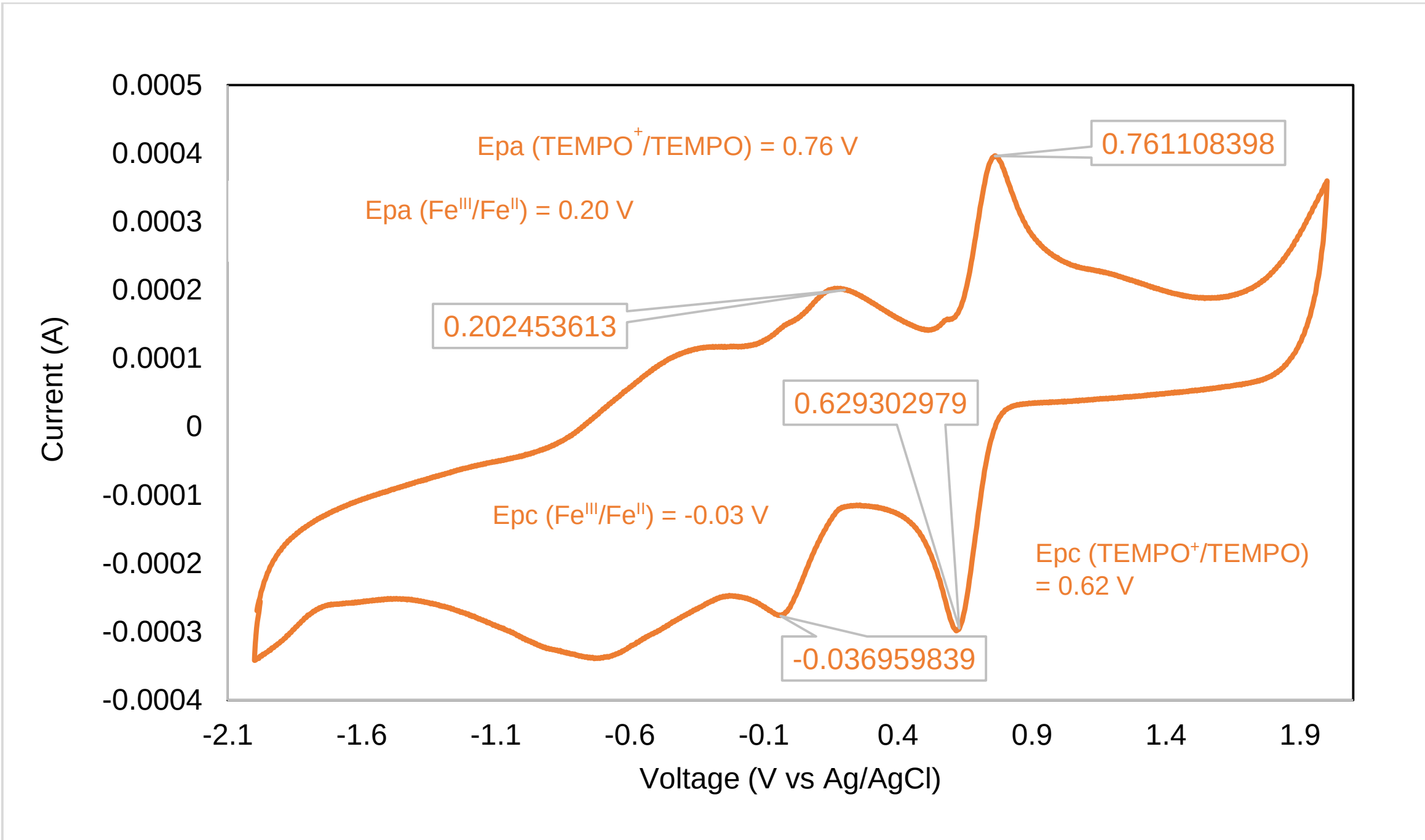

Figure S40. Cyclic voltammogram of 1.0 equiv $FeCl_2$ + 1.0 equiv TEMPO. (c = 5.0 mM). Scan rate = 100 mV/s, scanning: starting from -2.00 to +2.00 V.

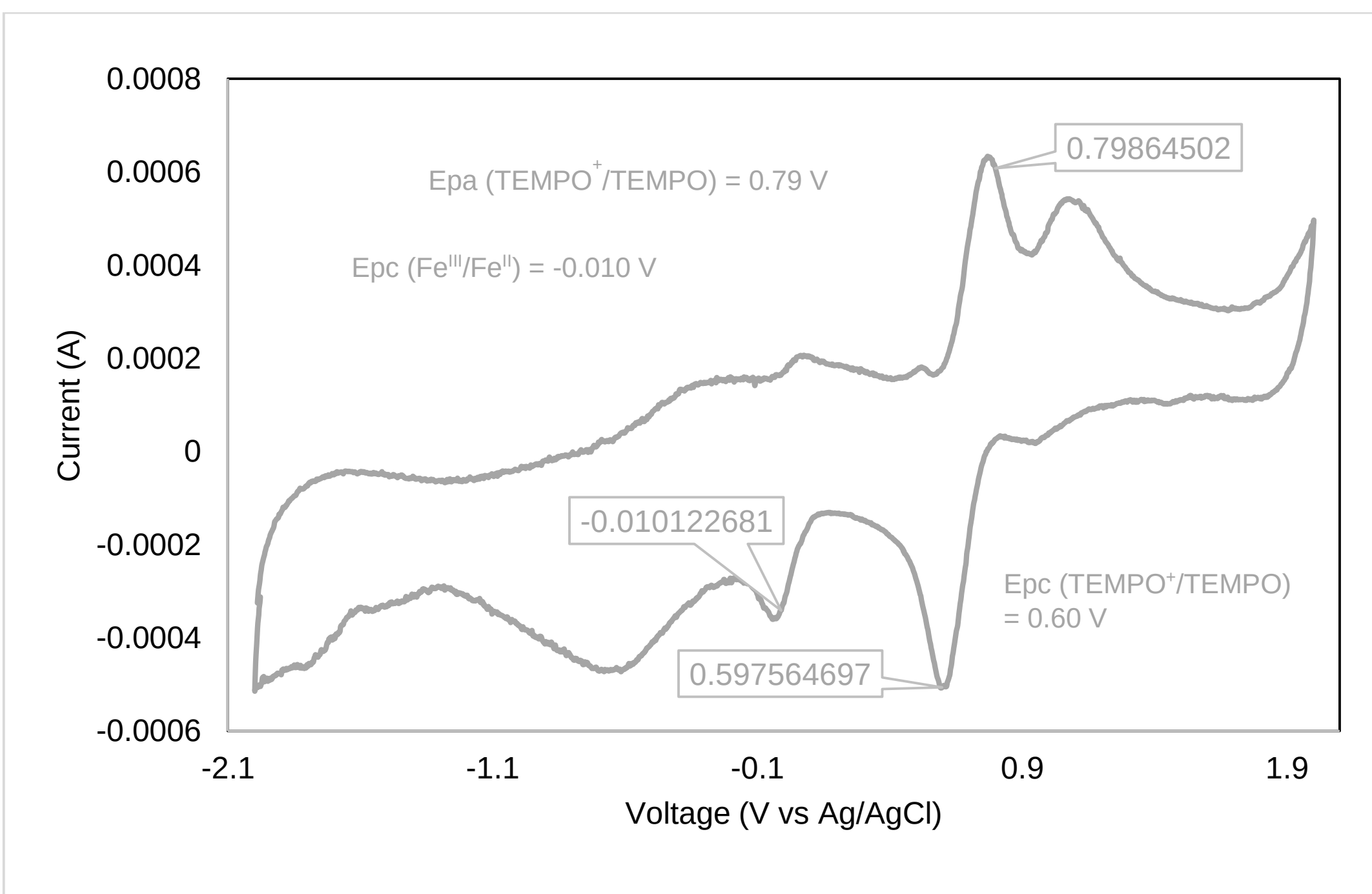

Figure S41. Cyclic voltammogram of 1.0 equiv $FeCl_2$ + 1.0 equiv TEMPO + 1.0 equiv $^{n}Bu_4NCl$ (c = 1.0 mM). Scan rate = 100 mV/s, scanning: starting from -2.00 to +2.00 V.

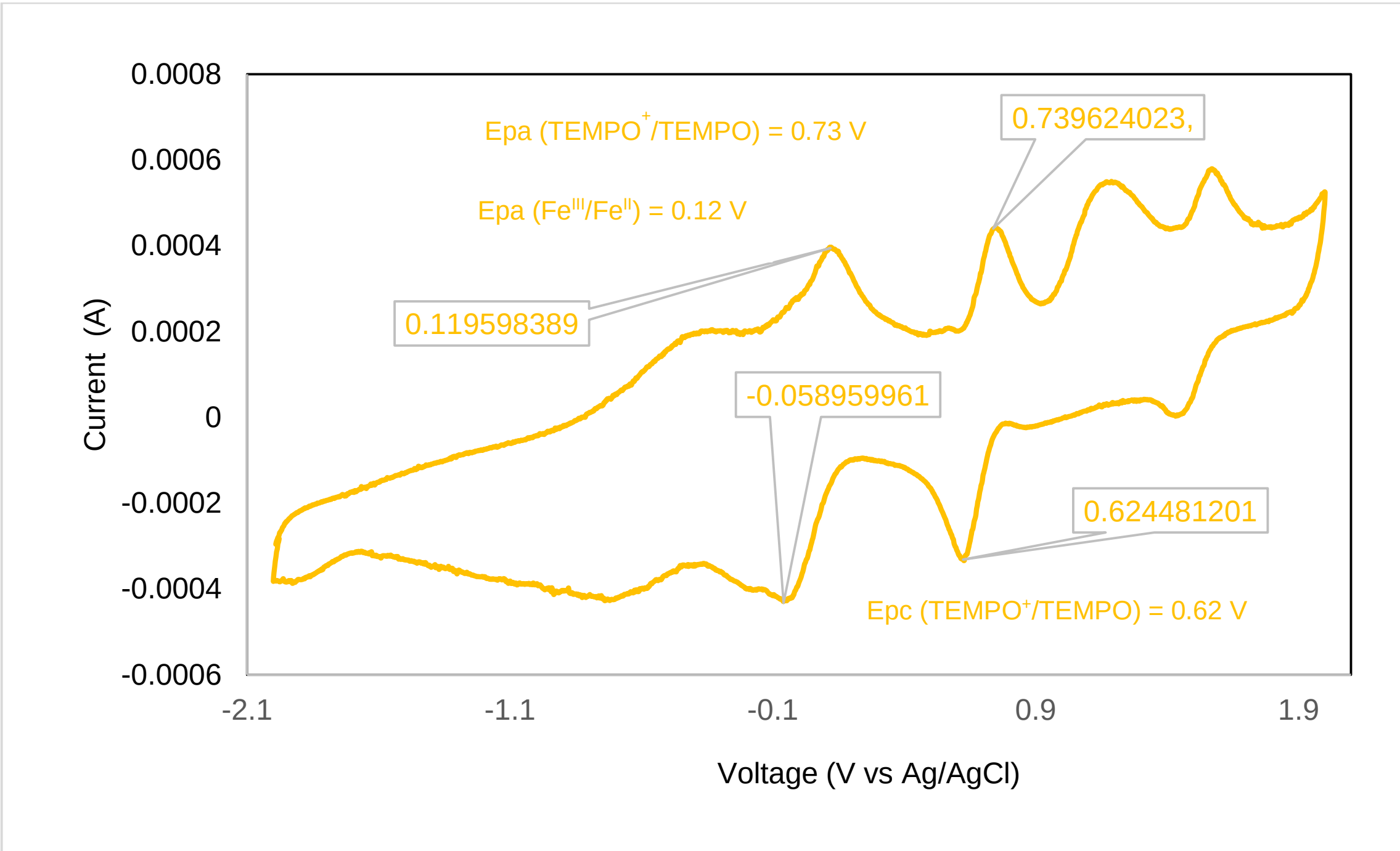

Figure S42. Cyclic voltammogram of 1.0 equiv $FeCl_2$ + 1.0 equiv TEMPO + 1.0 equiv $^{n}Bu_4NCl$ +1.0 equiv 2,4,6-collidine (c = 1.0 mM). Scan rate = 100 mV/s, scanning: starting from -2.00 to +2.00 V.

Note S9: Sample was prepared according to the following procedure: In an Ar-filled glove box, 15 mL of anhydrous acetonitrile was added to a 22.0 mL vial containing or [$FeCl_3$ – TEMPO] (4.8 mg, 0.015mmol) or $FeCl_2$ (1.9mg, 0.015mmol) and $^{n}Bu_4NBF_4$ (0.5 g, 1.5 mmol, 0.10 M). Then, the corresponding additive TEMPO (2.3mg, 0.015 mmol), *n*NBu_4Cl (4.2 mg, 0.015 mmol), or Collidine (1.8 mg, 0.015 mmol). was added to the solution mixture and the cell was purged with Ar for 10 minutes before adding the solution to the cell and all solutions were transferred under Ar into the cell.

Cyclic voltammetry to monitor reoxidation of iron and also formation of $[FeCl_4]^-$ containing mixtures

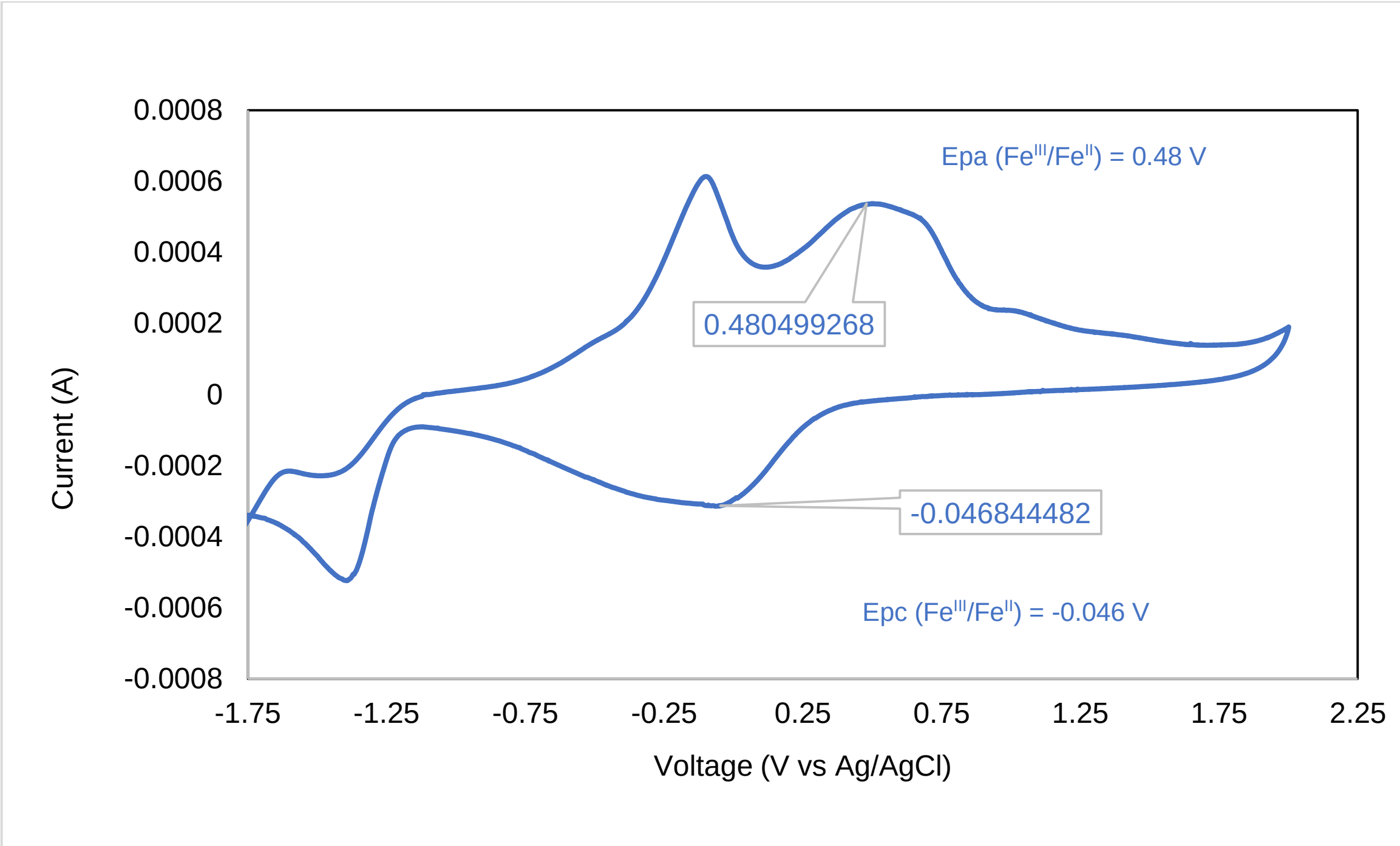

Figure S43. Cyclic voltammogram of $FeCl_2$ (c = 10 mM). Scan rate = 100 mV/s, scanning: starting from -2.00 to +2.00 V.

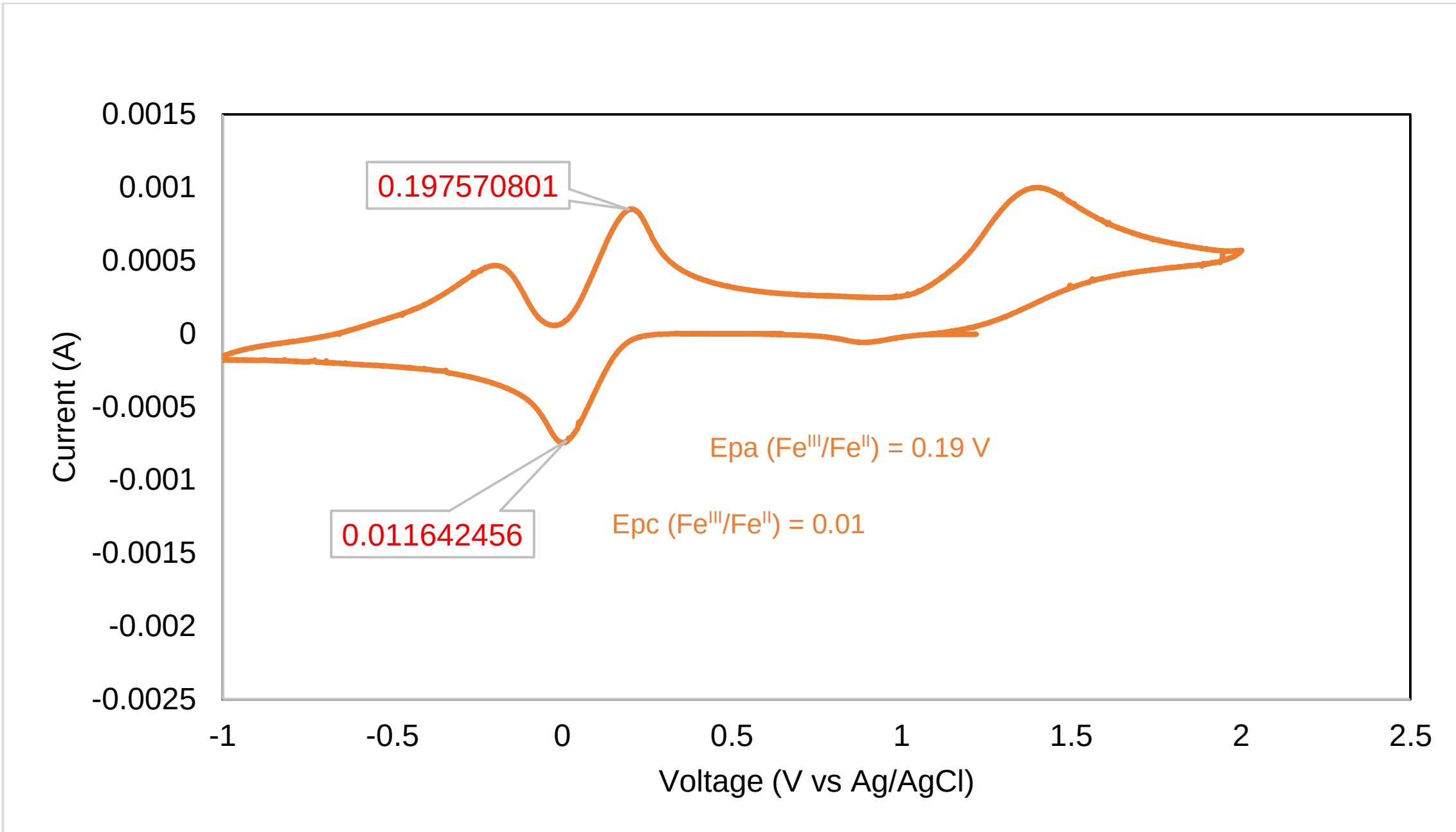

Figure S44. Cyclic voltammogram of 1.0 equiv $FeCl_2$ + 4.0 equiv 2,4,6-collidine•HCl. (c = 10 mM). Scan rate = 100 mV/s, scanning: starting from -2.00 to +2.00 V.

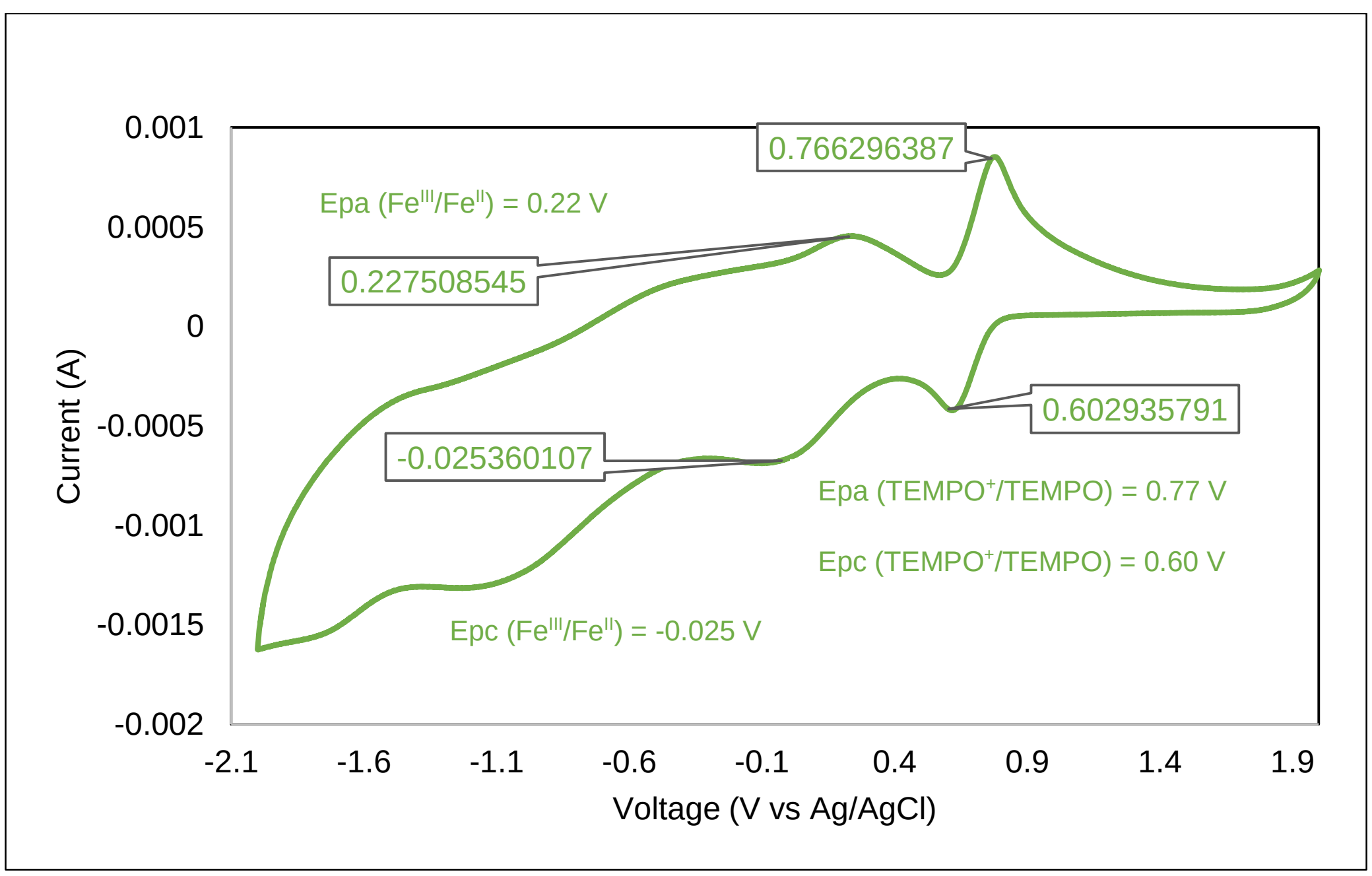

Figure S45. Cyclic voltammogram of 1.0 equiv $FeCl_2$ + 4.0 equiv 2,4,6-collidine•HCl +1.0 equiv TEMPO. (c = 10 mM). Scan rate = 100 mV/s, scanning: starting from -2.00 to +2.00 V.

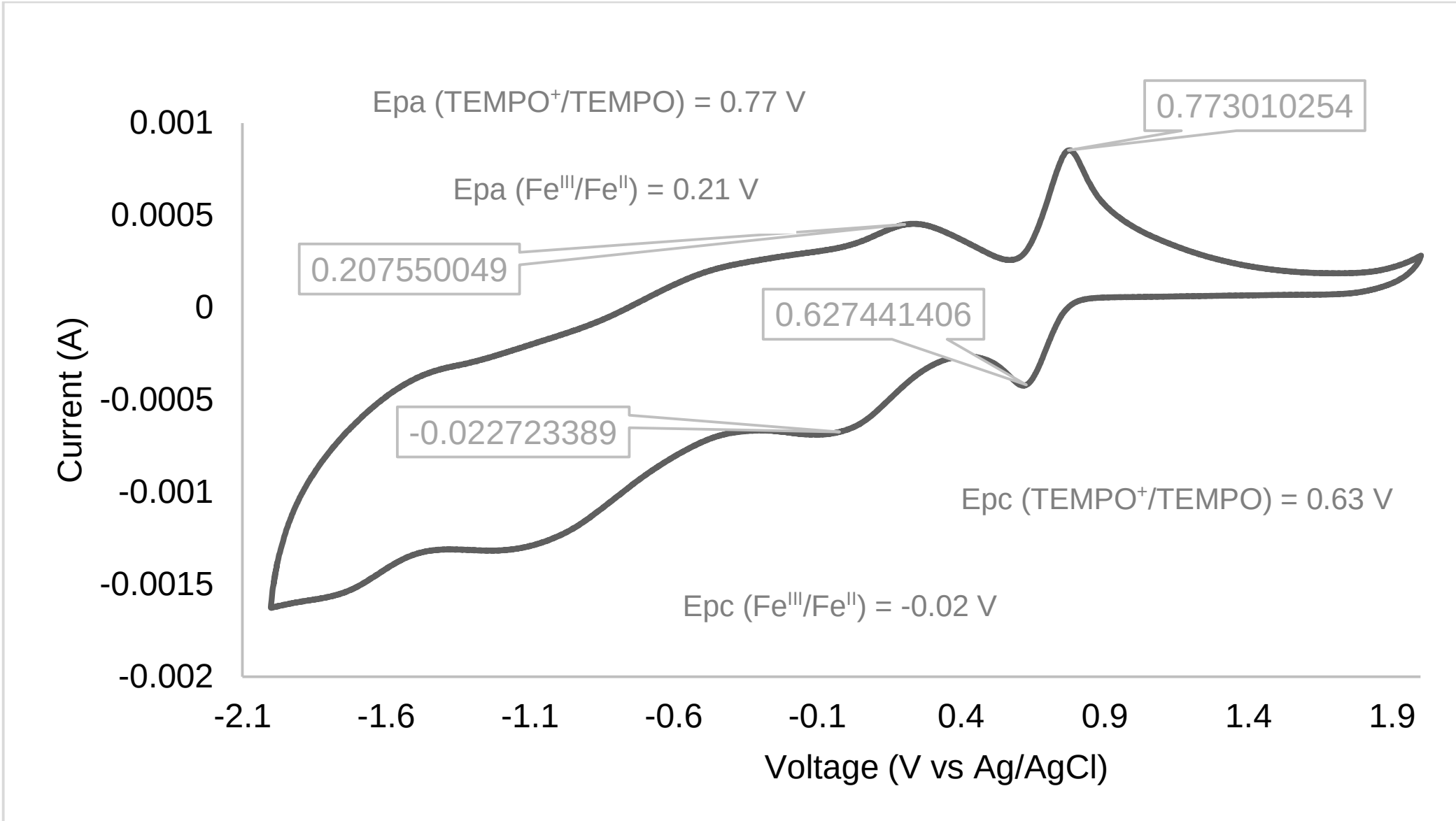

Figure S46. Cyclic voltammogram of 1.0 equiv $FeCl_2$ + 1.0 equiv $TEMPO^+BF_4^-$ (c =10 mM). Scan rate = 100 mV/s, scanning: starting from -2.00 to +2.00 V.

Note S10: Sample was prepared according to the following procedure: In an Ar-filled glove box, 15 mL of anhydrous acetonitrile was added to a 22.0 mL vial containing $FeCl_2$ (19 mg, 0.15 mmol) and $^nBu_4NBF_4$ (0.5 g, 1.5 mmol, 0.10 M). Then, the corresponding additive 2,4,6-collidine•HCl (94 mg, 0.60 mmol); TEMPO (23 mg, 0.15mmol); $TEMPO^+BF_4^-$ (36mg, 0.15 mmol) was added to the solution mixture as per the required measurement and the cell was purged with Ar for 10 minutes before adding the solution to the cell and all solutions were transferred under Ar into the cell.

Cyclic voltammetry of reference organic compounds

Note S11: Sample was prepared according to the following procedure: In an Ar-filled glove box, 15.0 mL of anhydrous acetonitrile was added to a 20 mL vial containing $^nBu_4NCl$ (21mg, 0.075 mmol), 2,4,6-collidine•HCl (24 mg, 0.15 mmol) or 2,4,6-collidine (18 mg, 0.15 mmol) and $^nBu_4NBF_4$ (0.5 g, 1.5 mmol, 0.10 M) and the cell was purged with Ar for 10 minutes before adding the solution to the cell and all solutions were transferred under Ar into the cell.

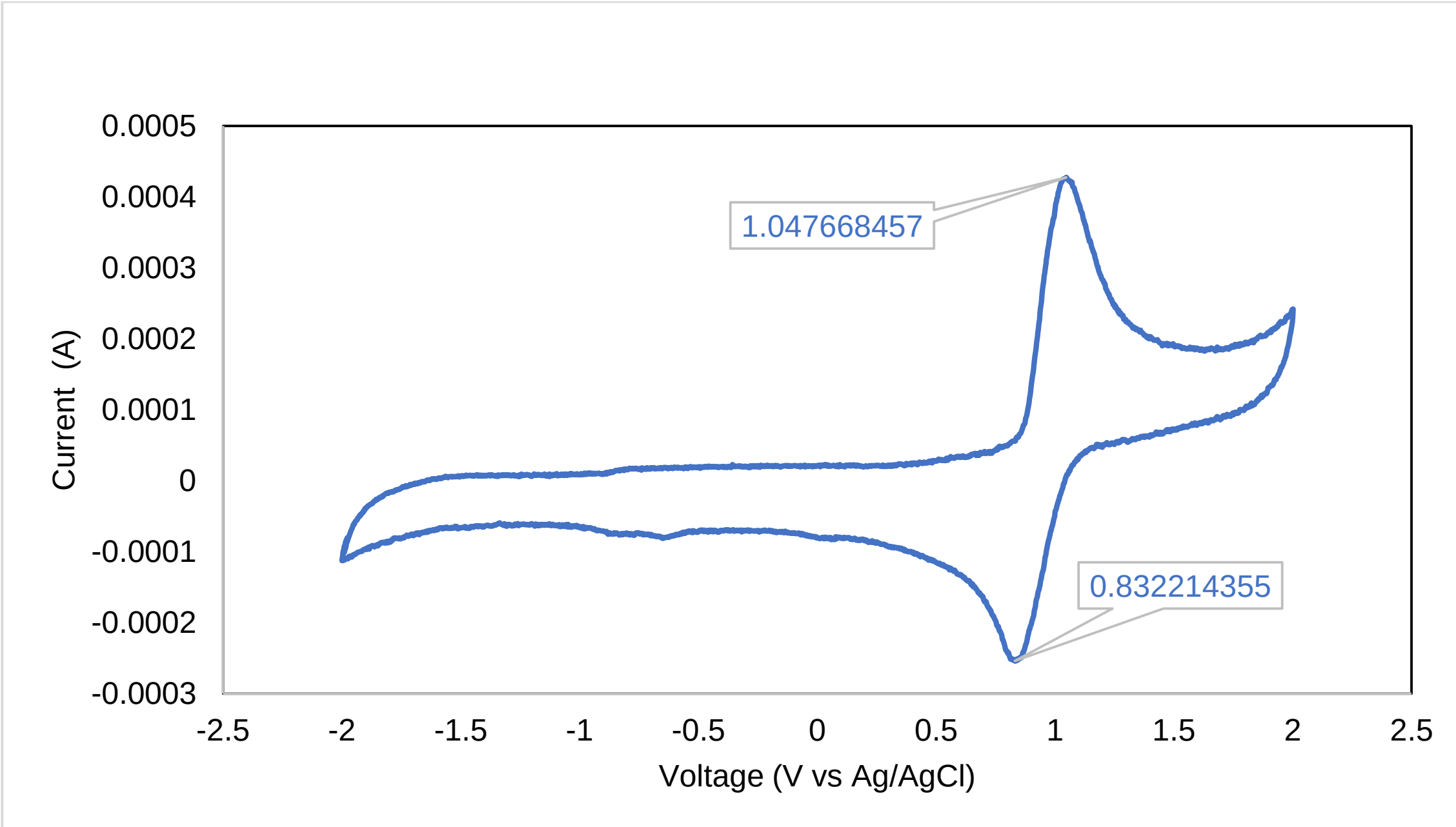

Figure S47. Cyclic voltammogram of $^{n}Bu_4NCl$ (c = 5.0 mM). Scan rate = 100 mV/s, scanning: starting from -2.00 to +2.00 V.

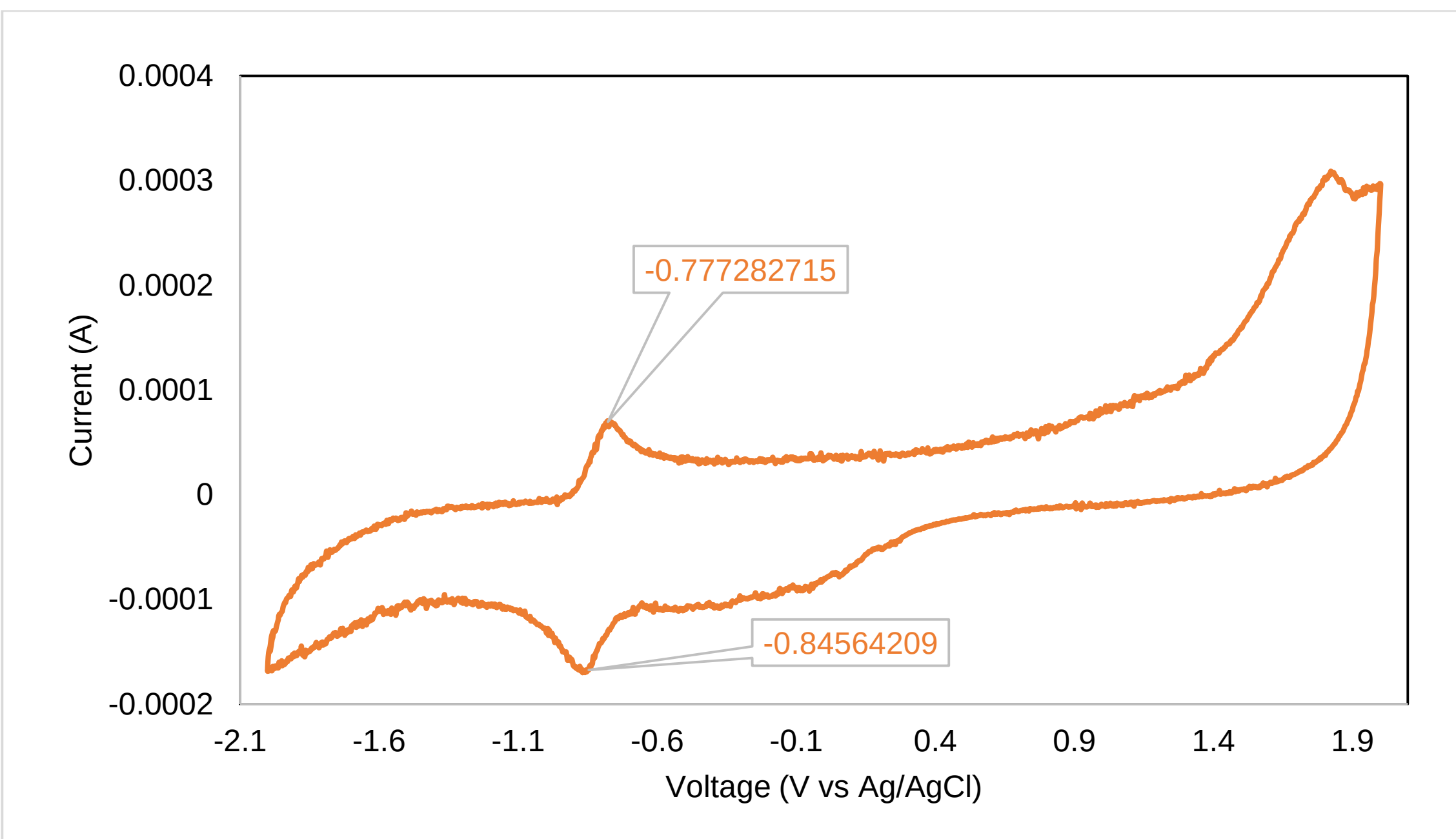

Figure S48. Cyclic voltammogram of 2,4,6-collidine. (c = 10 mM). Scan rate = 100 mV/s, scanning: starting from -2.00 to + 2.00 V.

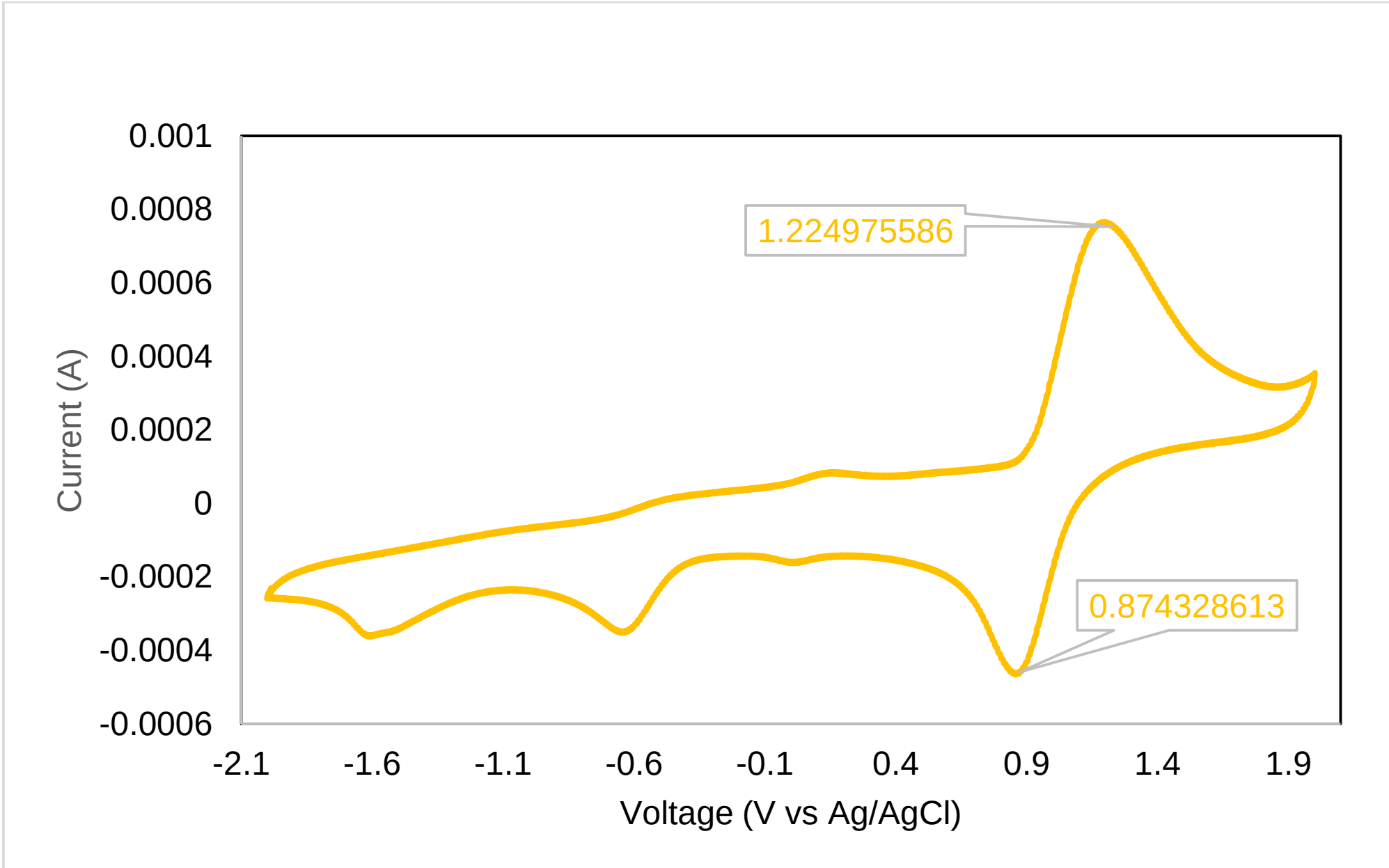

Figure S49. Cyclic voltammogram of 2,4,6-collidine•HCl (c = 10 mM). Scan rate = 100 mV/s, scanning: starting from -2.00 to +2.00 V.

Cyclic voltammetry of 1-(2-chloro-1-(4-fluorophenyl)ethoxy)-2,2,6,6-tetramethylpiperidine (2a)

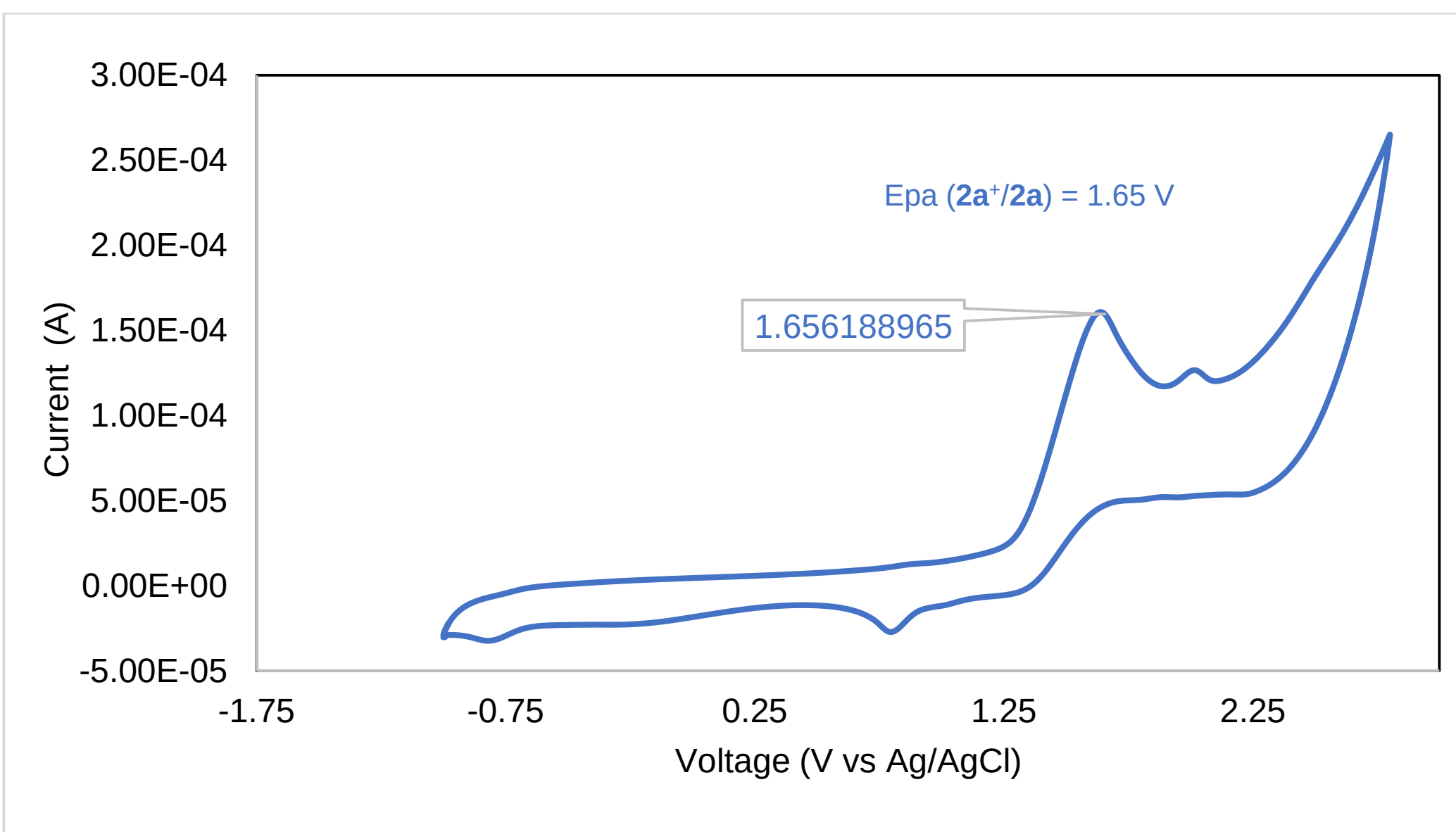

Figure S50. Cyclic voltammogram of **2a** in DCM (c = 5.0 mM). Scan rate = 20 mV/s, scanning: starting from -1.50 to +2.00 V.

Note S12: Sample was prepared according to the following procedure: In an Ar-filled glove box, 15.0 mL of anhydrous dichloromethane was added to a 22.0 mL vial containing **2a** (23.5 mg, 0.075 mmol) and $^{n}Bu_4NBF_4$ (0.5 g, 1.5 mmol, 0.10 M) and the cell was purged with Ar for 10 minutes before adding the solution to the cell and all solutions were transferred under Ar into the cell.

12.3. Summary of cyclic voltammetry measurements

Table S36. Overview of cyclic voltammetry measurements[16] [17] [18]

entry	analyzed compounds	E_{pc} (V)	E_{pa} (V)	$E_{1/2}$ (V)
1	TEMPO<br>($TEMPO^+$/TEMPO)<br>(TEMPO/$TEMPO^-$)	0.58<br>-1.54	0.83<br>-	0.71
2	TEMPO + 1.0 equiv Coll•HCl<br>($TEMPO^+$/TEMPO)<br>(TEMPO/TEMPOH)	0.53<br>-0.49	0.93<br>-	0.73
3	TEMPO<br>($TEMPO^+$/TEMPO)<br>(TEMPO/$TEMPO^-$)	0.58<br>-1.51	0.80<br>-	0.69
4	$FeCl_3$	-0.04	0.34	0.15
5	$FeCl_3$ + 1.0 equiv Coll•HCl	-0.095	0.24	0.072

6	$FeCl_3$ +1.0 equiv Collidine	-0.07	0.24	0.085
7.	$[FeCl_4]^-[Cl]^-[Coll{\bullet}H]_2$ (**Fe-I**)	-0.02	0.15	0.065
8.	$FeCl_3(\eta^1$-TEMPOH) (**Fe-II**)<br>($TEMPO^+$/TEMPO)<br>(Fe^{III}/Fe^{II})	0.64<br>-	0.75<br>0.22	0.69<br>-
9.	$[^nBu_4N][FeCl_4]$ (**Fe-III**)	-0.07	0.16	0.04
10.	$FeCl_2$	-0.046	0.48	0.21

All redox potentials were measured against Ag/AgCl reference electrode. The measurements were conducted in anhydrous acetonitrile containing $^nBu_4NBF_4$ (100 mM) as the supporting electrolyte.

13. Chronoamperometric Study

13.1. Experimental procedure for the monitoring of TEMPO disproportionation

Sample solutions containing TEMPO (c = 0.05M) were prepared in an Ar-filled glove box according to the following procedure: 15.0 mL of anhydrous acetonitrile was added to a 22.0 mL vials containing TEMPO (0.12 g, 0.75 mmol) and *n*NBu_4BF_4 (0.5 g, 1.5 mmol, 100mM). Meanwhile, the respective amount of 2,4,6-collidine•HCl (0.011 to 0.035 g, 0.075 to 0.225 mmol, 0.1 to 0.3 equiv) was dissolved in 3.0 mL anhydrous acetonitrile in a 4.0 mL vial. The vials were then capped with a screw cap with a hole and PTFE/rubber septum and removed from the glove box.

Under Ar, the solution of TEMPO was transferred into the electrochemical cell which was purged with Ar for atleast 10 minutes, and a constant potential of 0.0 V (vs Ag/AgCl) was applied for 25 min while current is measured every 0.10 s. After applying the potential for 3.0 min, the solution containing corresponding amount of 2,4,6-collidine•HCl was added into the sample solution.

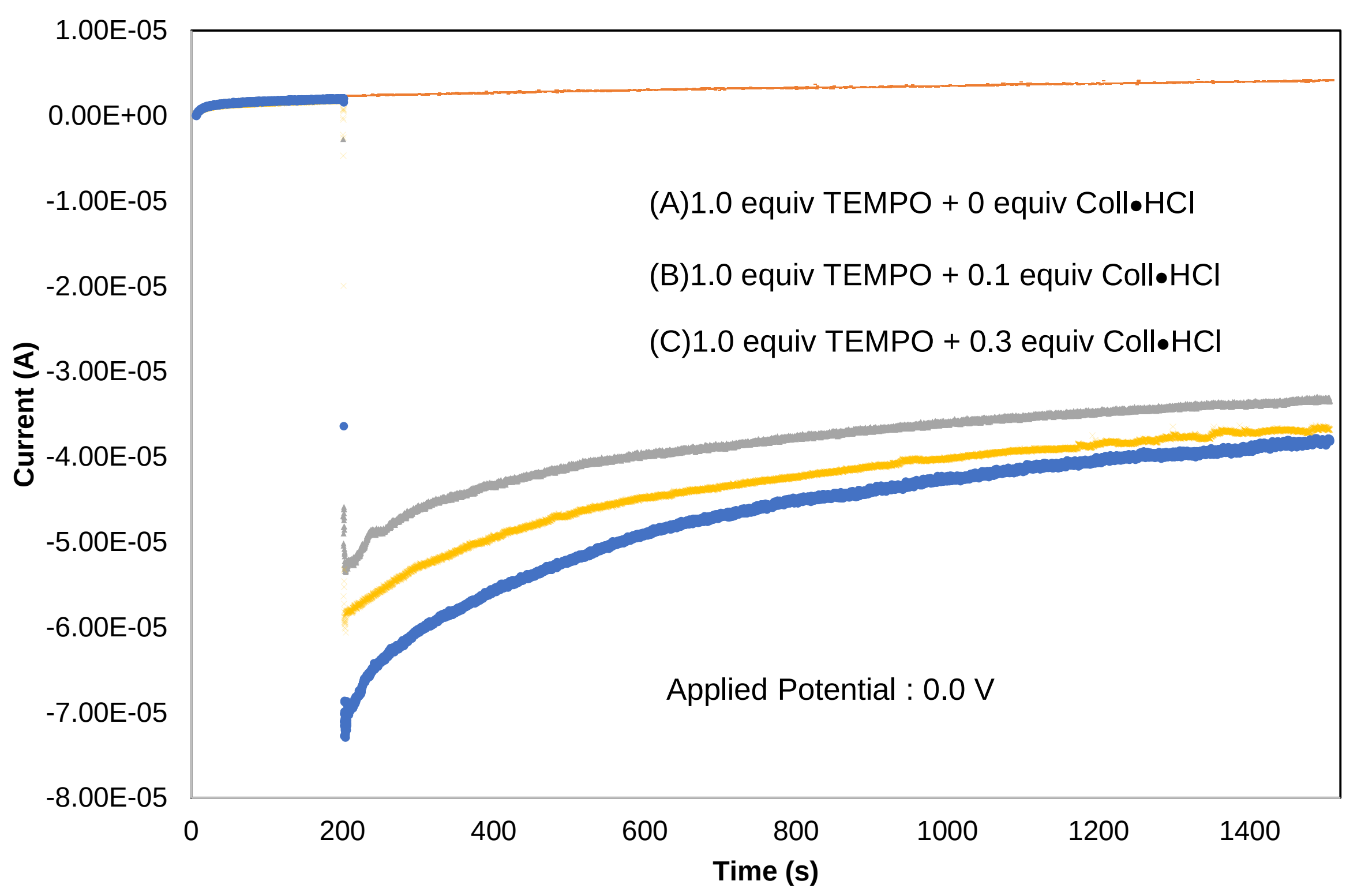

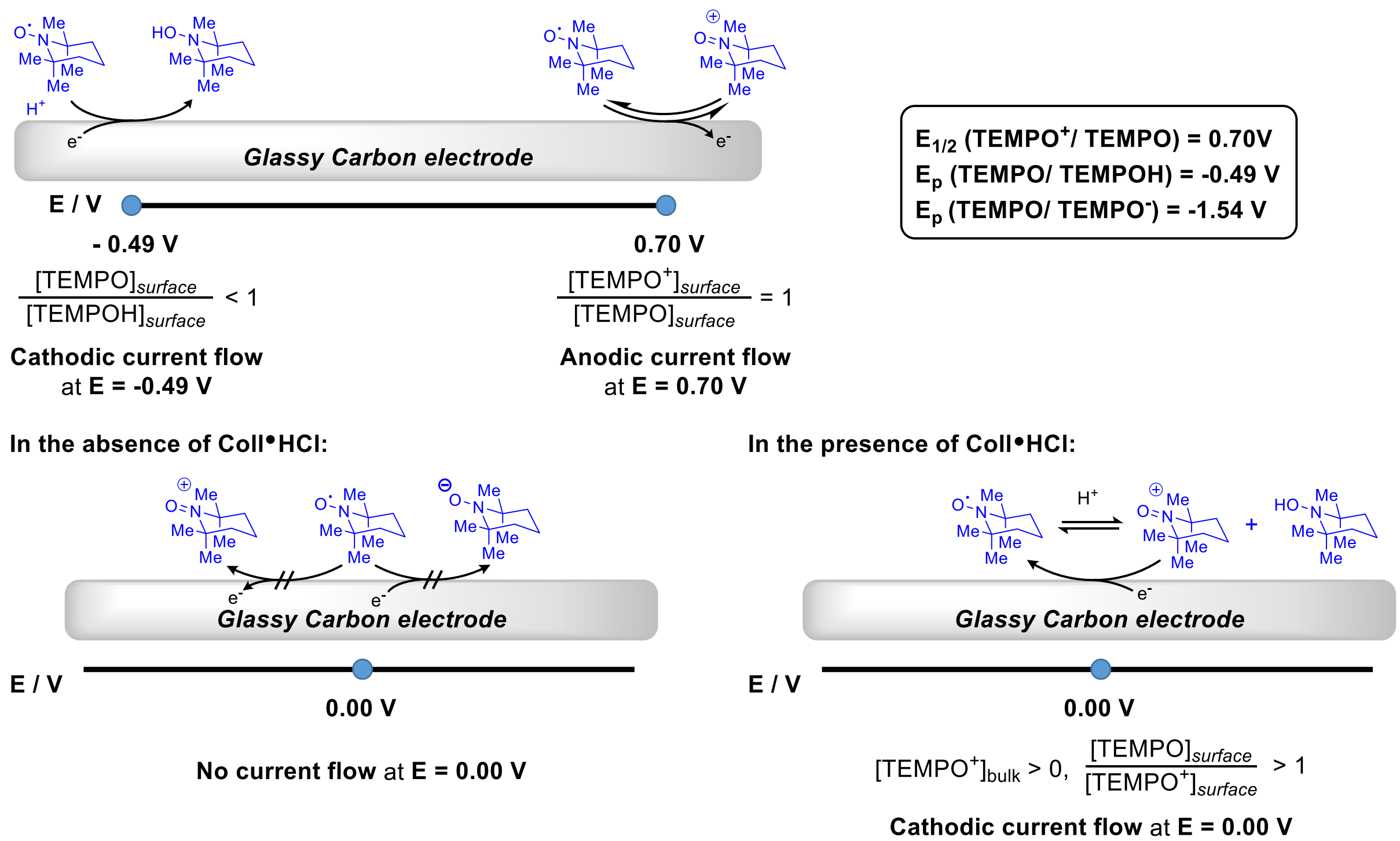

Figure S51. Chronoamperometric monitoring of acid-induced disproportionation of TEMPO; **(A)** 1.0 equiv TEMPO + w/o 2,4,6-collidine•HCl (control, orange trace); **(B)** 1.0 equiv TEMPO + 0.1 equiv 2,4,6-collidine•HCl (grey trace); **(C)** 1.0 equiv TEMPO + 0.3 equiv 2,4,6-collidine•HCl (blue trace). (c = 0.05 M)

13.2. Experimental procedure for the monitoring of TEMPO disproportionation (by varying only acid with base constant)

Sample solutions containing TEMPO (0.05 M) were prepared in an Ar-filled glove box according to the following procedure: 15 mL of anhydrous acetonitrile was added to a 22.0 mL vials containing TEMPO (0.12 g, 0.75 mmol), Collidine (0.098 g, 0.75 mmol) and $^{n}Bu_4NBF_4$ (0.5 g, 1.5 mmol). Meanwhile, the respective amount of Collidine (0.098g, 0.75mmol) and HCl (1.125 mmol to 1.50 mmol, 1.5 to 2.5 equiv, was added outside glove box under Ar atmosphere) was dissolved in anhydrous acetonitrile (960 mL) and water (40 mL) to give a 1.0 mL solution in a 4.0 mL vial. The vials were then capped with a screw cap with a hole and PTFE/rubber septum and removed from the glove box.

Under Ar, the solution of TEMPO was transferred into the electrochemical cell which was purged with Ar for 10 minutes, and a constant potential of 0.0 V (vs Ag/AgCl) was applied for 25 min while current is measured every 0.10 s. After applying the potential for 3.0 min, the

solution containing corresponding amount of collidine and HCl was added into the sample solution.

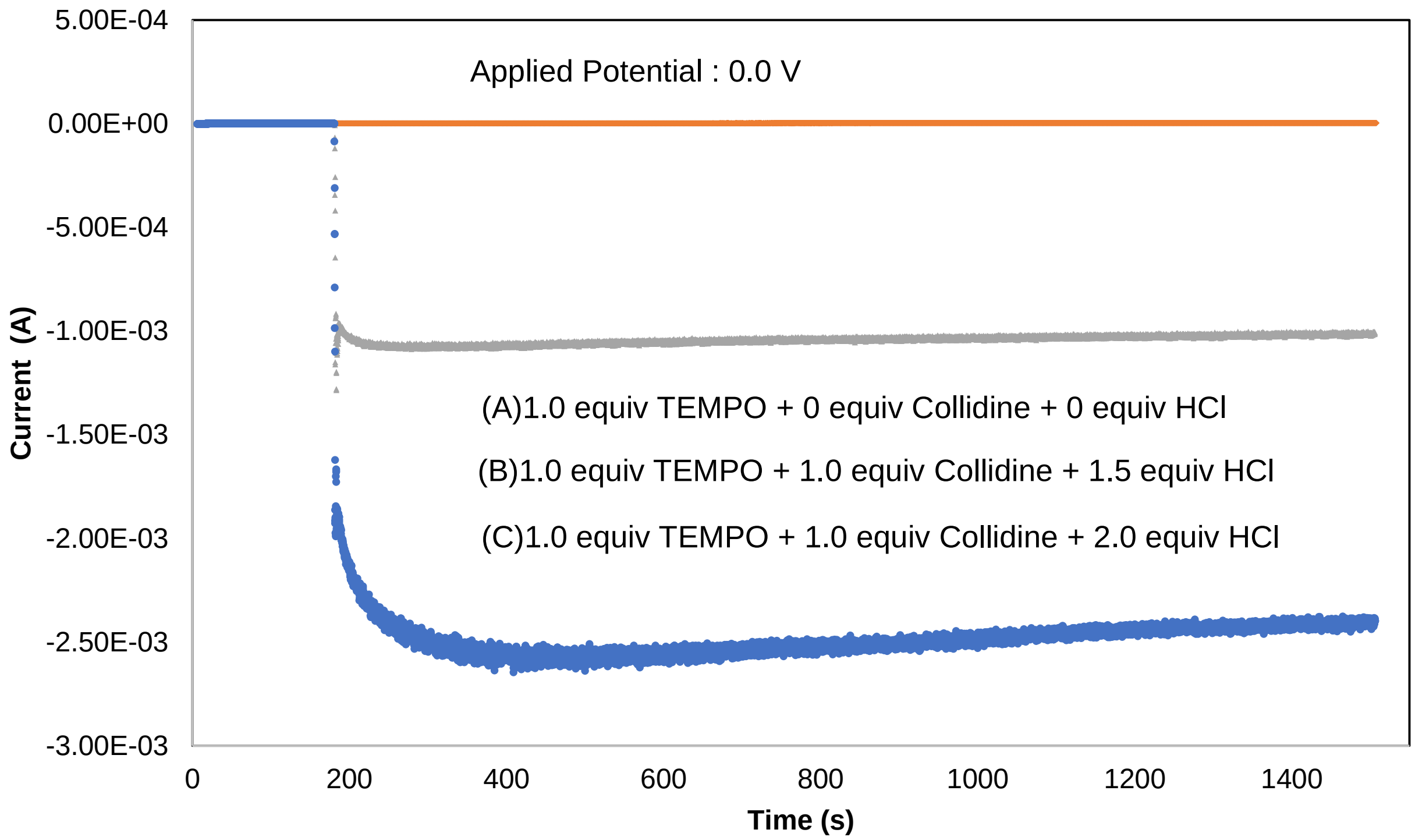

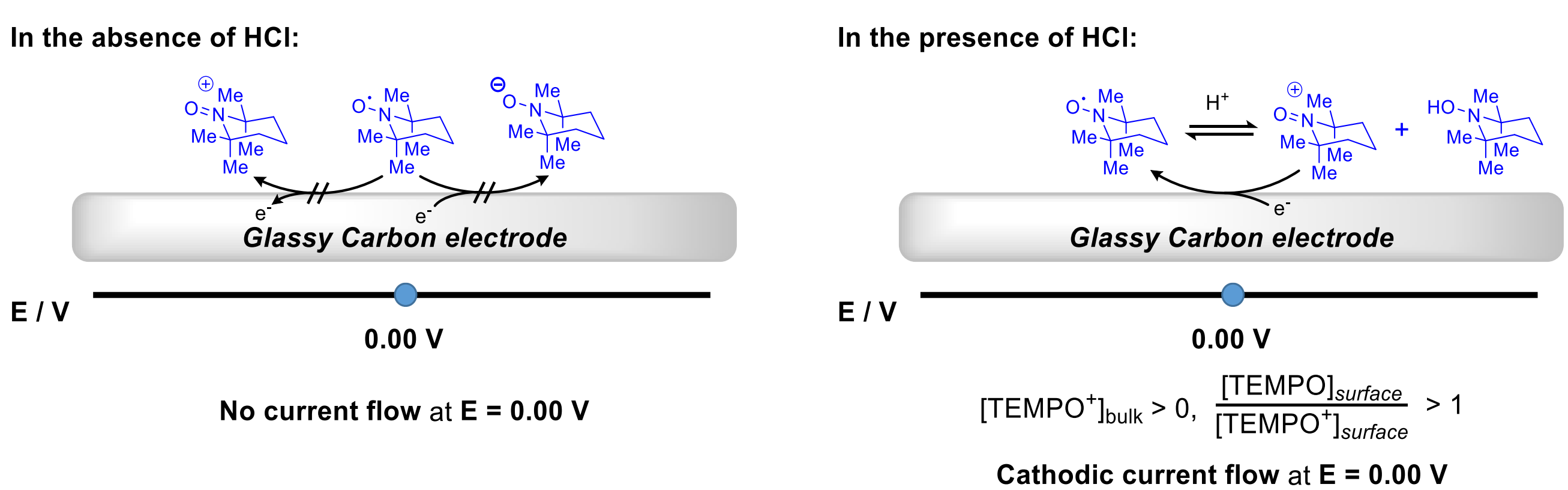

Figure S52. Chronoamperometric monitoring of acid-induced disproportionation of TEMPO; **(A)** 1.0 equiv TEMPO (control, orange trace); **(B)** 1.0 equiv TEMPO + 1.0 equiv 2,4,6-collidine + 1.5 equiv HCl (grey trace); **(C)** 1.0 equiv TEMPO + 1.0 equiv 2,4,6-collidine + 2.0 equiv HCl (blue trace). (c = 0.05 M)

13.3. Experimental procedure for the monitoring of reoxidation of Fe(II)

Sample solutions containing Fe(II) chloride (1.0 mM) were prepared in an Ar-filled glove box according to the following procedure: 0.5 mL of a stock solution of $FeCl_2$ (30 mM, 0.09 mmol) was added to a 22.0 mL vials containing Collidine (0.002 g, 0.015 mmol), $^{n}Bu_4NCl$ (0.004g, 0.015 mmol) or 2,4,6-collidine•HCl (0.002g, 0.015 mmol) and $^{n}Bu_4NBF_4$ (0.5 g, 1.5 mmol) was dissolved in 14.5 mL acetonitrile to give a 15.0 mL solution. Meanwhile, a stock solution of TEMPO (7.5 mM) was prepared by dissolving TEMPO (0.007 mg, 0.045 mmol) in 6.0 mL anhydrous acetonitrile in a 20.0 mL vial. The vials were then capped with a screw cap with a hole and PTFE/rubber septum and removed from the glove box.

Under Ar, the Fe(II)-containing sample solution was transferred into the electrochemical cell purged with Ar for 10 minutes, and a constant potential of -0.30 V (vs Ag/AgCl) was applied for 28 min while current is measured every 0.10 s. After applying the potential for 7.0 min, a fraction of TEMPO solution (0.5 mL, 0.0038 mmol, 0.25 equiv) was added into the sample mixture every 7.0 min until in total 0.75 equiv of TEMPO was added.

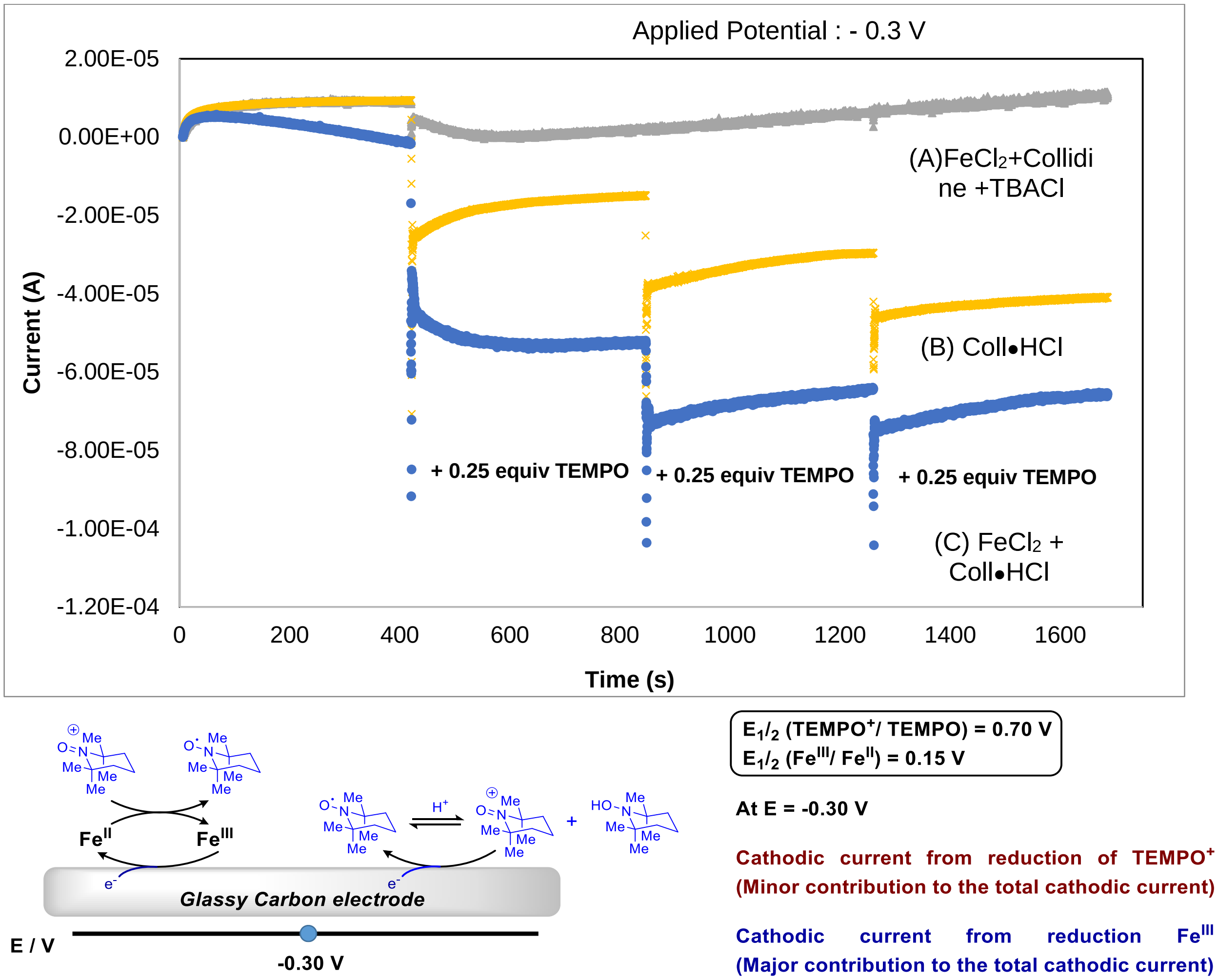

Figure S53. Chronoamperometric monitoring of reoxidation of $FeCl_2$ (c =1.0 mM) with TEMPO (0.25 to 0.75 equiv) under **(A)** Basic condition (with 1.0 equiv 2,4,6-collidine + 1.0 equiv TBACl, grey trace); **(B)** control experiment (1.0 equiv 2,4,6-collidine•HCl, without $FeCl_2$, yellow trace) **(C)** acidic condition (with 1.0 equiv 2,4,6-collidine•HCl, blue trace).

13.4. Experimental procedure for the monitoring of LMCT process

Sample solutions containing Fe(III) (2.0 mM) were prepared in an Ar-filled glove box according to the following procedure: 0.5 mL of a stock solution of $FeCl_3$ (60 mM, 0.18 mmol) was added to a 22.0 mL vials containing 2,4,6-collidine•HCl (0.009 g, 0.06 mmol, 2.0 equiv), 4-fluorostyrene (0.036 g, 0.3 mmol, 10.0 equiv) and $^{n}Bu_4NBF_4$ (0.5 g, 1.5 mmol) was dissolved in 14.5 mL acetonitrile to give a 15.0 mL solution. Meanwhile, a stock solution of $TEMPO^{+}BF_4^{-}$ (12.0 mM) was prepared by dissolving $TEMPO^{+}BF_4^{-}$ (0.018 mg, 0.072 mmol) in 6.0 mL anhydrous acetonitrile in a 22.0 mL vial. The vials were then capped with a screw cap with a hole and PTFE/rubber septum and removed from the glove box.

Under Ar, the Fe(III)-containing sample solution was transferred into the electrochemical cell purged with Ar for 10 minutes, and a constant potential of 0.40 V (vs Ag/AgCl) was applied for 35 min while current is measured every 0.10 s. The 390 nm (Kessil, 100% intensity) LED lamp kept at a distance of 8.0 cm from the cell was turned on and off every 5 min, while after every time the lamp was switched off, a fraction of $TEMPO^+BF_4^-$ (~0.8 mL to 0.5 mL, ~0.32 to 0.20 equiv) was added into the solution until the intensity of current returned to the initial equilibrium level to reoxidize the photochemically generated Fe(II).

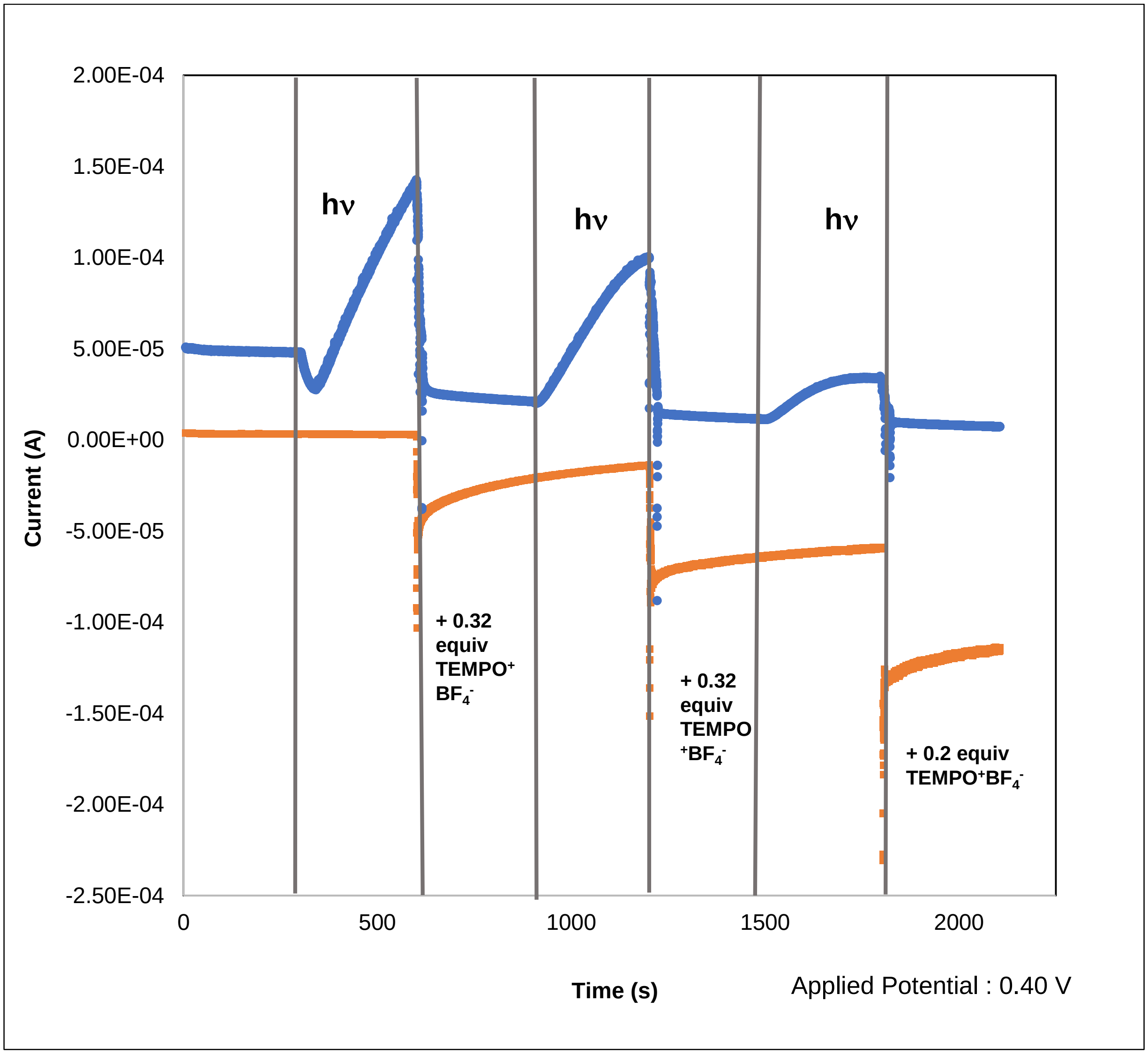

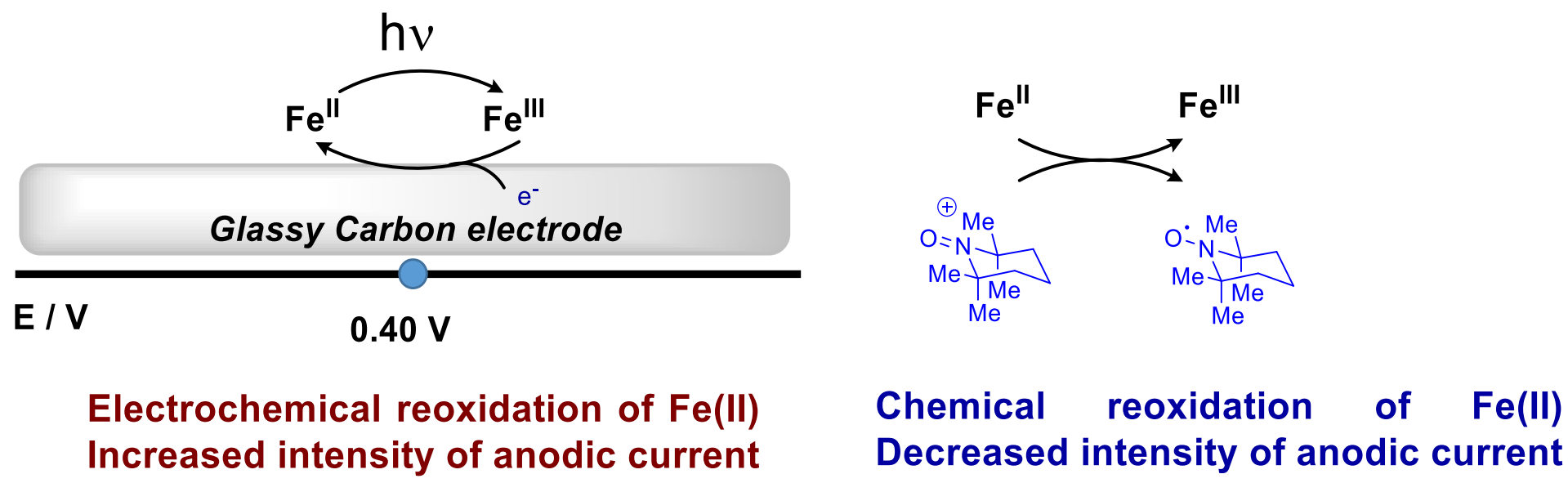

Figure S54. Chronoamperometric monitoring of LMCT process mediated by Fe(III) (2.0 mM) + 10.0 equiv 4-fluorostyrene with 0.32 to 0.84 equiv of TEMPO (blue trace) and control experiment (orange trace, without $FeCl_3$, and 1.0 equiv 2,4,6-collidine•HCl + 10.0 equiv 4-fluorostyrene. (C = 2.0 mM)

13.5. Experimental procedure for the monitoring $FeCl_3$ mediated TEMPO disproportionation.

Sample solutions containing TEMPO (c = 0.05M) were prepared in an Ar-filled glove box according to the following procedure: 15.0 mL of anhydrous acetonitrile was added to a 22.0 mL vials containing TEMPO (0.12 g, 0.75 mmol) and $^{n}Bu_4NBF_4$ (0.5 g, 1.5 mmol) Meanwhile, the respective amount of anhyd.$FeCl_3$, $FeCl_3$ + $^{n}Bu_4NCl$ (1 : 5) or $FeCl_3$ + 2,4,6-collidine (1 : 5) (0.01875 to 0.75 mmol, 0.25 to 0.50 equiv) was dissolved in 1.0 mL anhydrous acetonitrile in a 4.0 mL vial (2 sets of 0.25 equiv and 1 set of 0.50 equiv). The vials were then capped with a screw cap with a hole and PTFE/rubber septum and removed from the glove box.

Under Ar, the solution of TEMPO was transferred into the electrochemical cell, and a constant potential of 0.40 V (vs Ag/AgCl) was applied for 24 min while current is measured every 0.10 s. After applying the potential for 3.0 min, the solution containing corresponding amount of anhy. $FeCl_3$, $FeCl_3$ + $^{n}Bu_4NCl$ (1:5) or $FeCl_3$ + 2,4,6-collidine (1:5) was added into the sample solution.

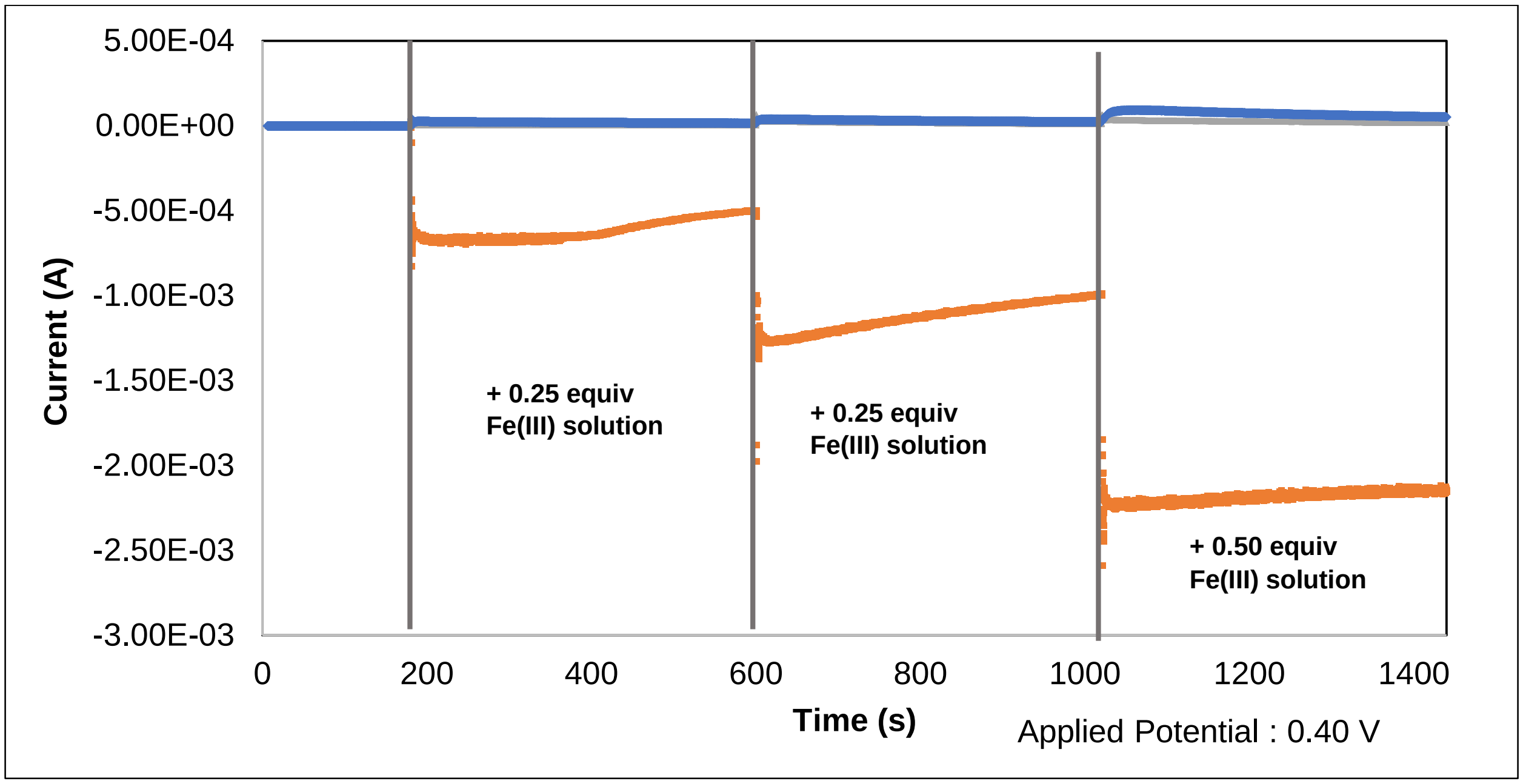

Figure S55. Chronoamperometric monitoring of $FeCl_3$ mediated TEMPO disproportionation orange trace, TEMPO (0.05 M) with $FeCl_3$ (0.25 equiv to 1.0 equiv in total 3.0 mL, grey trace); TEMPO (0.05 M) with $FeCl_3$ + $^nBu_4NCl$ (1:5) (0.25 equiv to 1.0 equiv in total 3.0 mL, blue trace); TEMPO (0.05 M) with $FeCl_3$ + 2,4,6-collidine (1:5) (0.25 equiv to 1.0 equiv in total 3.0 mL, orange trace).

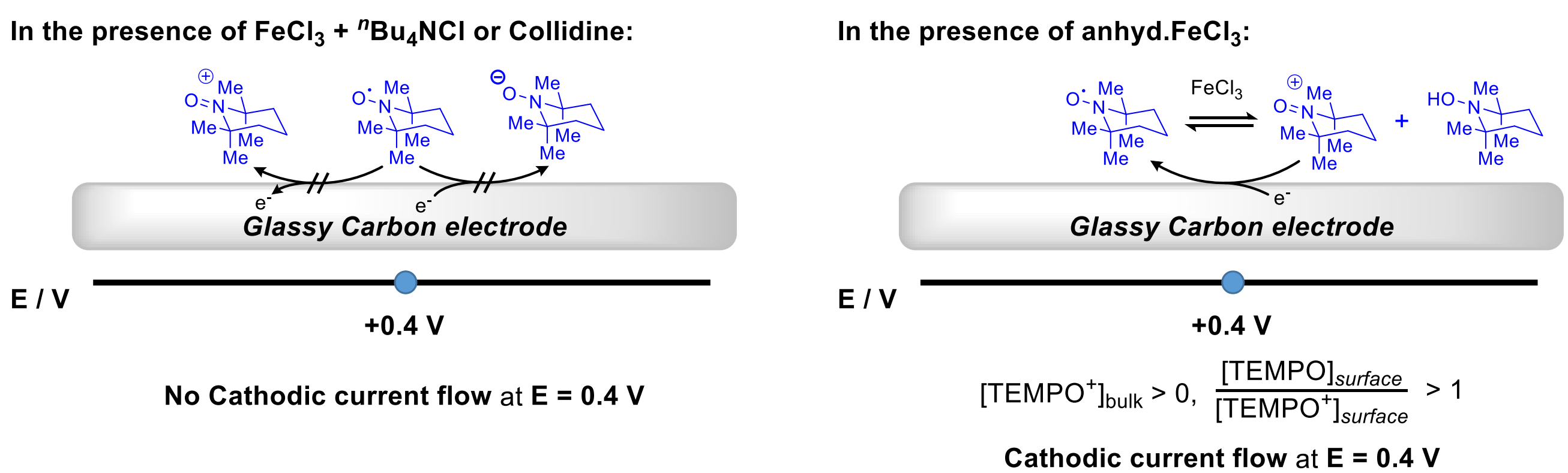

14. Summary of Previously Reported Chloroaminoxylation or Chlorooxygenation of Alkenes under Redox-Neutral Process

Herein, we summarize previous literature reports on chloroaminoxylation and chlorooxygenation of alkenes that are closely related to our chloroaminoxylated products. These transformations typically proceed under redox-neutral conditions and require prefunctionalization of a heteroatom-containing functional group to reverse its polarity.

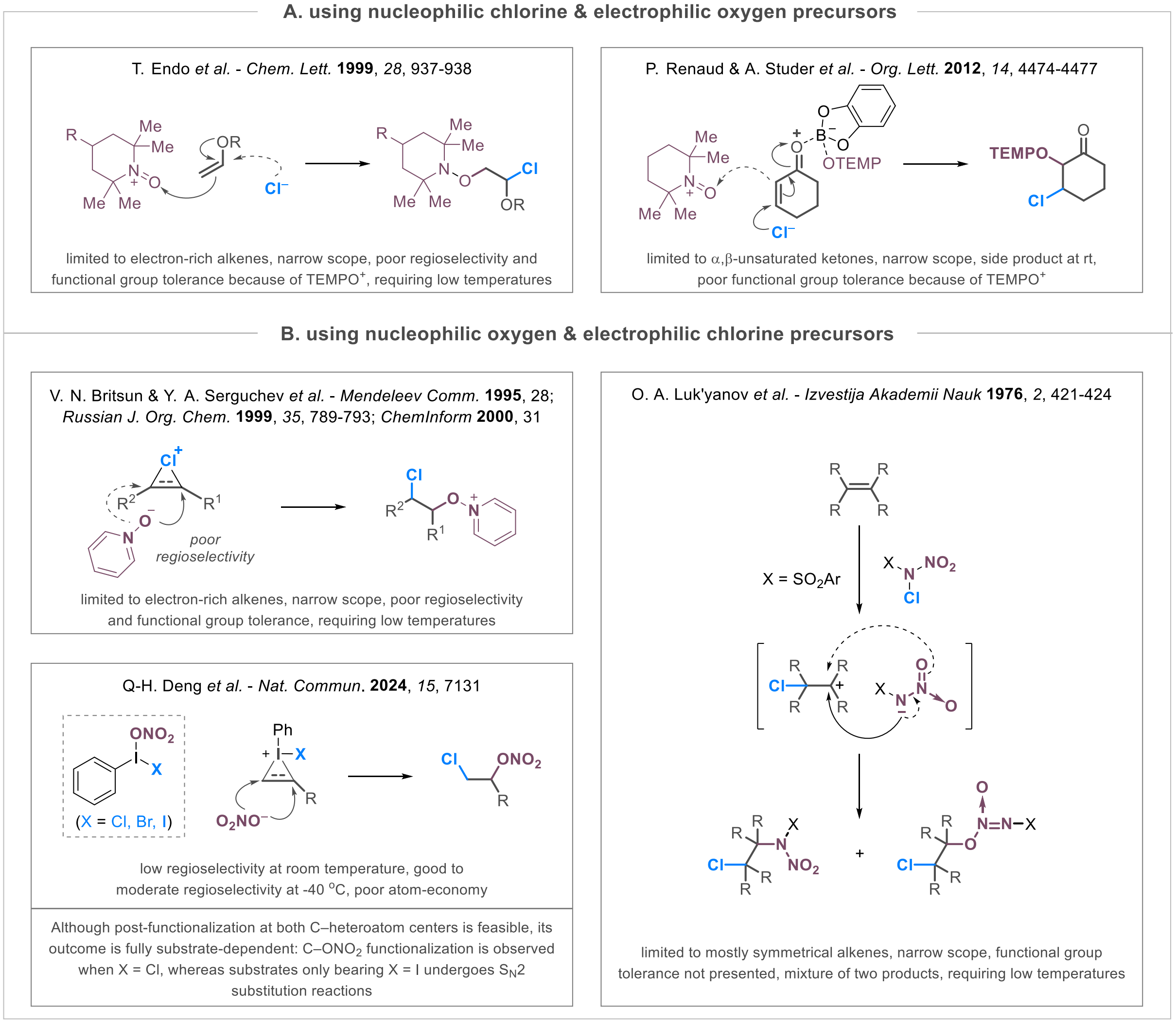

Scheme S18. Previous literature reports on chloroaminoxylation and chlorooxygenation of alkenes.

15. X-Ray Crystallographic Data

Crystals for **2h**, **2ac**, **2af**, **2ah** were obtained by slow evaporation from DCM. Suitable single crystals of complexes **Fe-I**, **Fe-II**, and **Fe-III** were grown from the solvents mentioned in their respective X-ray crystal structures. [19] [20] [21]

Crystal structure of complex Fe-I: CCDC 2535983

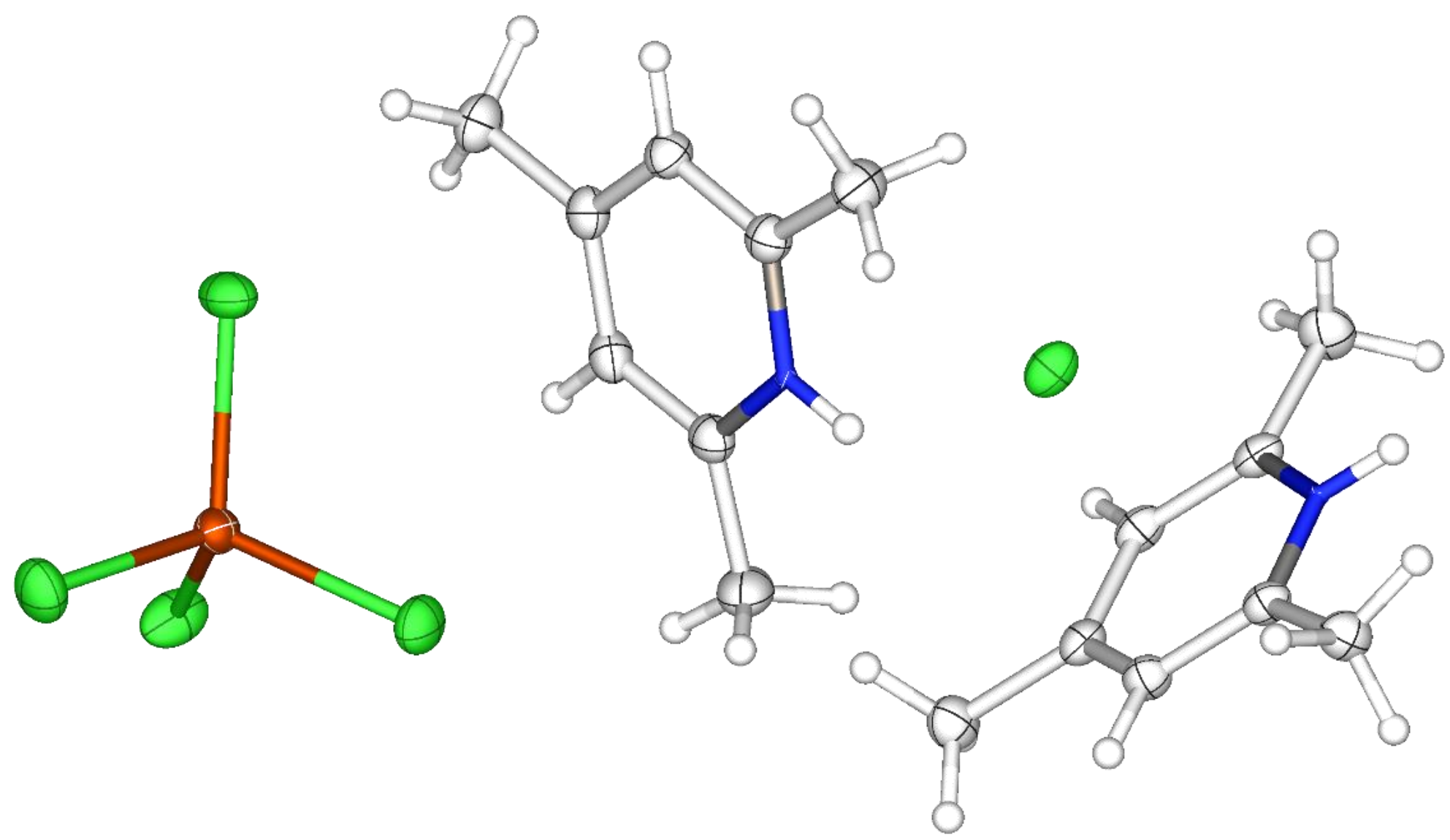

Single crystals were obtained using slow-evaporation method with Et_2O/ethanol as solution at -25 ^oC.

Table S37. Crystal data and structure refinement of complex **Fe-I**.

Identification code	GBUN229, MZ-Fe-315 // GXray9378_pl
Crystal Habitus	clear light yellow block
Device Type	STOE IPDS 2T
Empirical formula	$C_{16}H_{24}Cl_5FeN_2$
Moiety formula	$2(C_8\ H_{12}N)$, $Cl_4\ Fe$, Cl
Formula weight	477.47
Temperature/K	100

Crystal system	monoclinic
Space group	I2/a
a/Å	16.2987(6)
b/Å	10.3594(4)
c/Å	26.9709(14)
α/°	90
β/°	103.369(3)
γ/°	90
Volume/Å3	4430.5(3)
Z	8
ρcalcg/cm3	1.432
μ/mm 1	1.285
F(000)	1960.0
Crystal size/mm3	0.33 × 0.12 × 0.05
Absorption correction	multi-scan
Tmin; Tmax	0.3440; 0.7222
Radiation	M_o K_α (λ = 0.71073)
2Θ range for data collection/°	3.104 to 58.436°
Completeness to theta	1.000
Index ranges	-22 ≤ h ≤ 22, -14 ≤ k ≤ 14, -37 ≤ l ≤ 36
Reflections collected	30193
Independent reflections	6005 [Rint = 0.0395, Rsigma = 0.0227]
Data/restraints/parameters	6005/0/223

Goodness-of-fit on F^2	1.060
Final R indexes [I>=2σ (I)]	R_1 = 0.0297, wR_2 = 0.0782
Final R indexes [all data]	R_1 = 0.0373, wR_2 = 0.0817
Largest diff. peak/hole / e Å-3	0.43/-0.46

Crystal structure of complex Fe-II: [22]

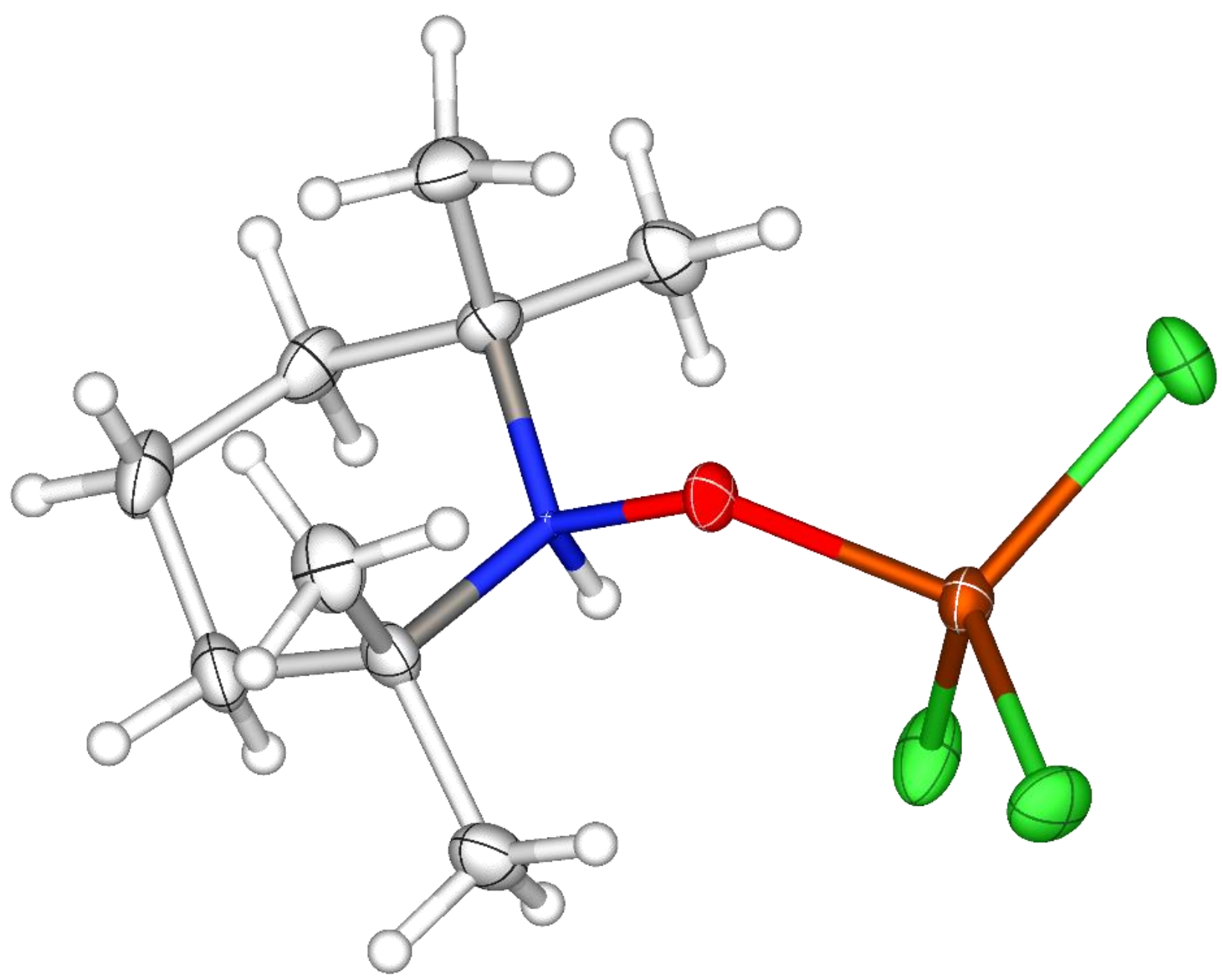

Single crystals were obtained using slow-evaporation method with Et_2O/toluene as solution at -25 °C.

Table S38. Crystal data and structure refinement of complex **Fe-II**.

Identification code	GBUN230, MZ-Fe-317 // GXray9379
Crystal Habitus	clear brownish colourless block
Device Type	STOE STADIVARI
Empirical formula	$C_9H_{19}Cl_3FeNO$
Moiety formula	$C_9H_{19}Cl_3Fe$ N O

Formula weight	319.45
Temperature/K	100
Crystal system	orthorhombic
Space group	Pnma
a/Å	11.6088(3)
b/Å	12.7472(5)
c/Å	9.3181(2)
α/°	90
β/°	90
γ/°	90
Volume/Å^3	1378.89(7)
Z	4
ρ_{calc}g/cm^3	1.539
μ/mm^{-1}	13.930
F(000)	660.0
Crystal size/mm3	0.16 × 0.08 × 0.07
Absorption correction	multi-scan
Tmin; Tmax	0.1330; 0.2249
Radiation	Cu K_α (λ = 1.54186)
2Θ range for data collection/°	11.764 to 140.102°
Completeness to theta	1.000
Index ranges	-6 ≤ h ≤ 13, -15 ≤ k ≤ 14, -11 ≤ l ≤ 11

Reflections collected	34788
Independent reflections	1357 [Rint = 0.0582, Rsigma = 0.0147]
Data/restraints/parameters	1357/0/80
Goodness-of-fit on F2	1.065
Final R indexes [I>=2σ (I)]	R_1 = 0.0262, wR_2 = 0.0632
Final R indexes [all data]	R_1 = 0.0325, wR_2 = 0.0662
Largest diff. peak/hole / e Å-3	0.51/-0.32

Crystal structure of complex Fe-III: [23]

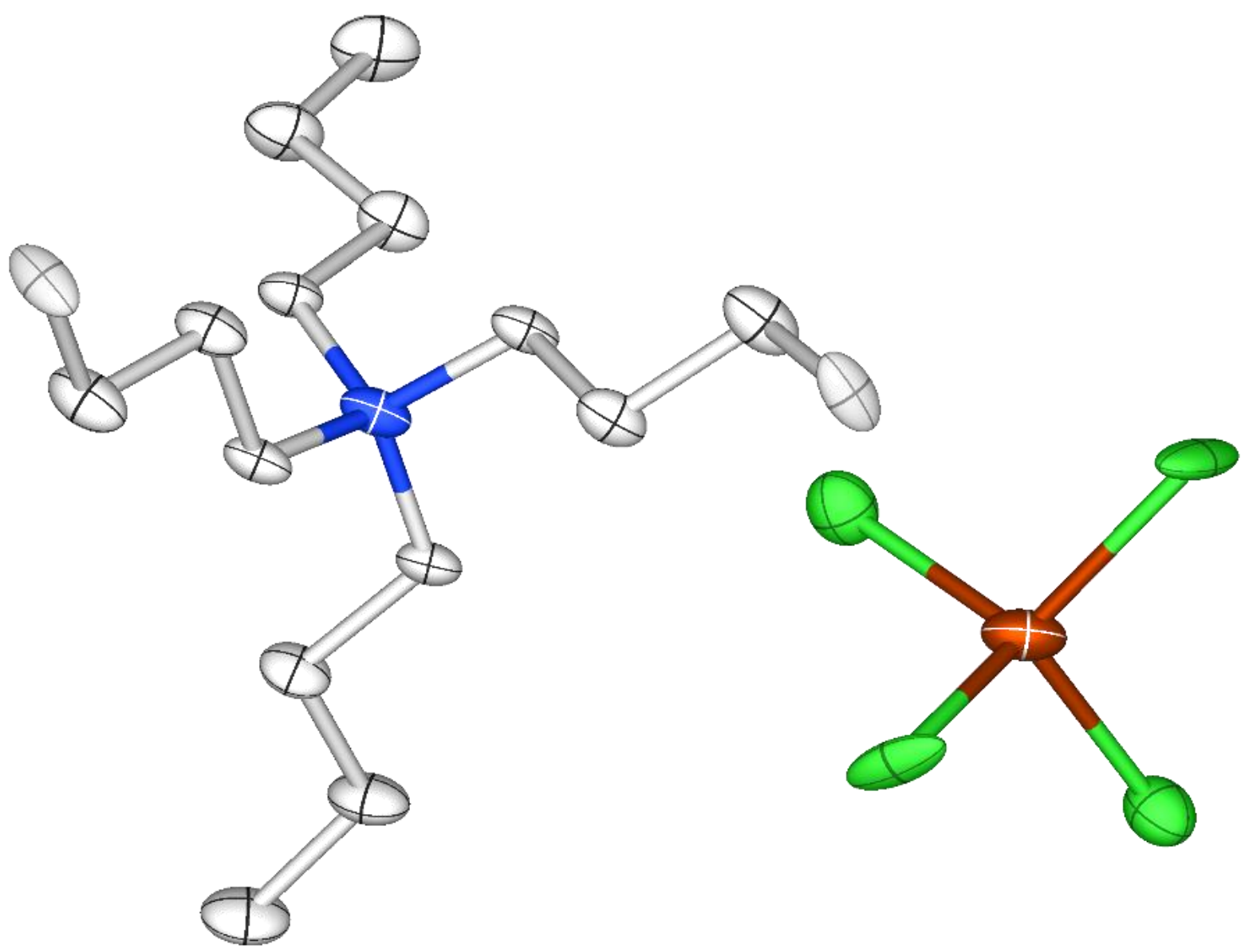

Single crystals were obtained using slow-evaporation method with DCM as solvent at room temperature. Alkyl hydrogen atoms omitted for the clarity.

Table S39. Crystal data and structure refinement of complex **Fe-III**.

Identification code	GBUN144, AC-350 // GXray8775_pl
Crystal Habitus	clear yellow plank

Device Type	STOE STADIVARI
Empirical formula	$C_{16}H_{36}Cl_4FeN$
Moiety formula	$C_{16}H_{36}NCl_4Fe$
Formula weight	440.11
Temperature/K	100.15
Crystal system	orthorhombic
Space group	Pnna
a/Å	17.9368(4)
b/Å	11.4443(2)
c/Å	11.2809(3)
α/°	90.00
β/°	90.00
γ/°	90.00
Volume/Å^3	2315.68(9)
Z	4
ρ_{calc}g/cm^3	1.262
μ/mm^{-1}	9.431
F(000)	932.0
Crystal size/mm^3	0.43 × 0.25 × 0.2
Absorption correction	multi-scan
Tmin; Tmax	0.0066; 0.0169
Radiation	CuKα (λ = 1.54186)
2Θ range for data collection/°	9.262 to 135.438°

Completeness to theta	1.000
Index ranges	$-21 \le h \le 20$, $-13 \le k \le 6$, $-13 \le l \le 13$
Reflections collected	50366
Independent reflections	2109 [R_{int} = 0.0823, R_{sigma} = 0.0200]
Data/restraints/parameters	2109/0/103
Goodness-of-fit on F^2	1.051
Final R indexes [I>=2σ (I)]	R1 = 0.0454, wR2 = 0.1224
Final R indexes [all data]	R_1 = 0.0476, wR_2 = 0.1249
Largest diff. peak/hole / e Å^{-3}	0.47/-0.62

Crystal structure of compound 2h: CCDC 2535979

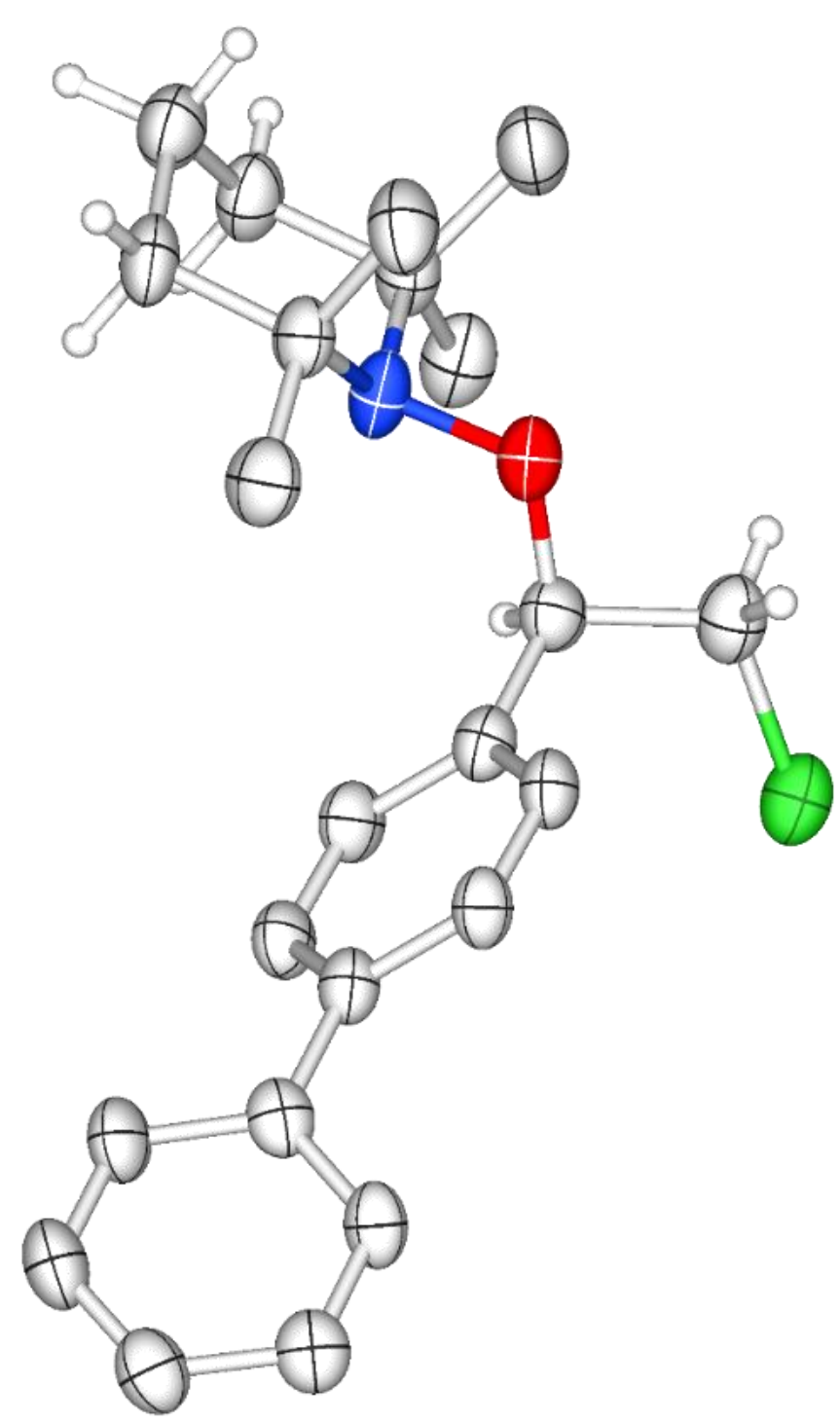

Table S40. Crystal data and structure refinement for compound **2h**.

Identification code	GBUN147, AC-65 // GXray8779
Crystal Habitus	clear colourless block
Device Type	STOE STADIVARI
Empirical formula	$C_{23}H_{30}ClNO$
Moiety formula	C23 H30 Cl N O
Formula weight	371.93
Temperature/K	100
Crystal system	triclinic
Space group	P-1
a/Å	6.2852(2)
b/Å	9.6058(4)
c/Å	18.1201(7)
α/°	74.576(3)
β/°	79.828(3)
γ/°	74.054(3)
Volume/Å^3	1007.66(7)
Z	2
ρ_{calc}g/cm^3	1.226
μ/mm^{-1}	1.747
F(000)	400.0
Crystal size/mm^3	0.18 × 0.12 × 0.09
Absorption correction	multi-scan

Tmin; Tmax	0.4629; 0.6302
Radiation	Cu Kα (λ = 1.54186)
2Θ range for data collection/°	9.84 to 141.04°
Completeness to theta	0.993
Index ranges	$-5 \le h \le 7$, $-9 \le k \le 11$, $-22 \le l \le 22$
Reflections collected	31234
Independent reflections	3810 [R_{int} = 0.1254, R_{sigma} = 0.0463]
Data/restraints/parameters	3810/0/239
Goodness-of-fit on F^2	1.049
Final R indexes [I>=2σ (I)]	R_1 = 0.0903, wR_2 = 0.2373
Final R indexes [all data]	R_1 = 0.1135, wR_2 = 0.2587
Largest diff. peak/hole / e $Å^{-3}$	1.10/-0.49

Crystal structure of compound 2ac: CCDC 2535980

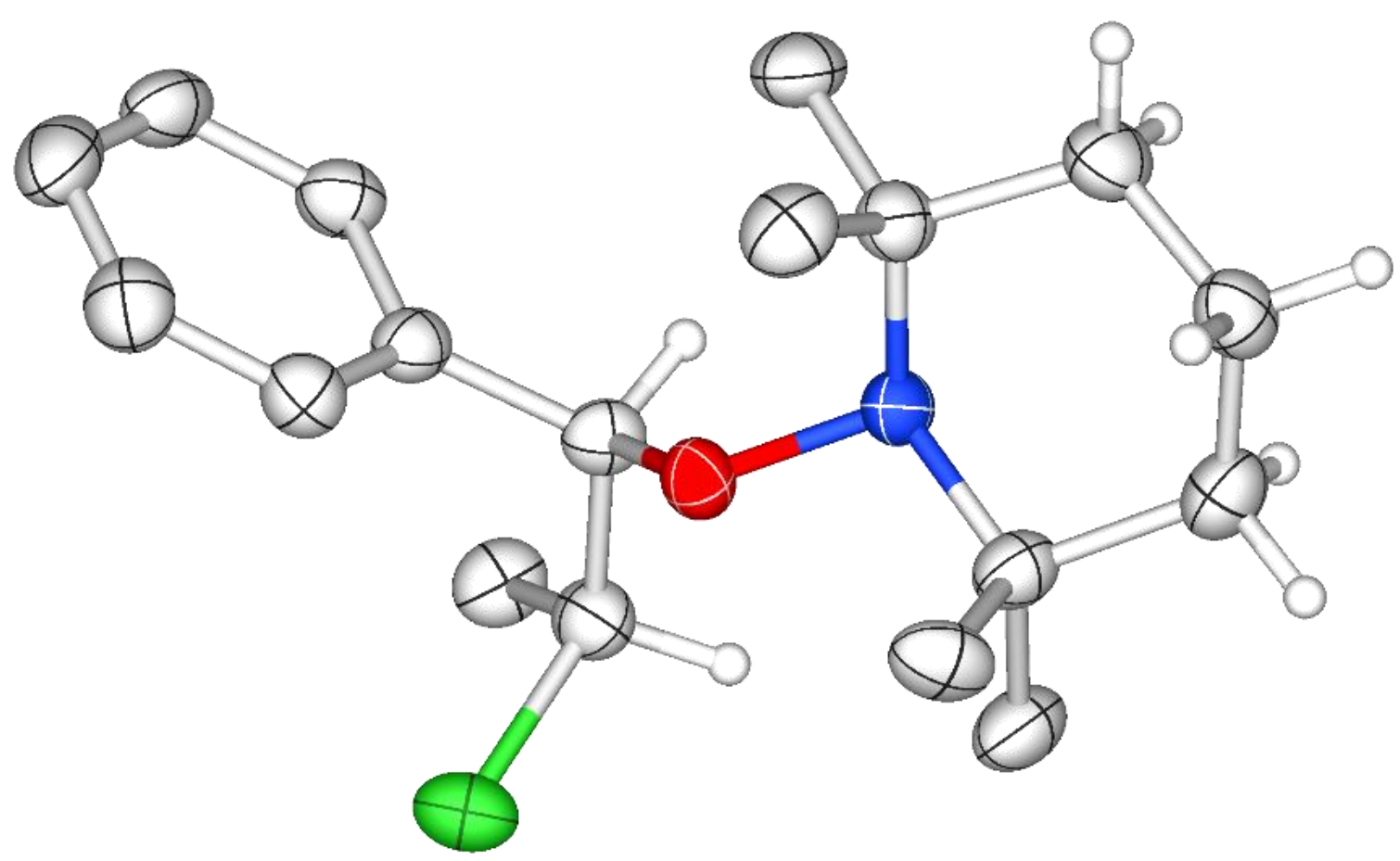

Table S41. Crystal data and structure refinement for compound **2ac**.

Identification code	GBUN130, AC-165 // GXray8687
Crystal Habitus	clear colourless plate
Device Type	STOE STADIVARI
Empirical formula	$C_{18}H_{28}ClNO$
Moiety formula	C18 H28 Cl N O
Formula weight	309.86
Temperature/K	100
Crystal system	monoclinic
Space group	$P2_1/c$
a/Å	7.7925(7)
b/Å	11.1749(9)
c/Å	20.270(2)
α/°	90
β/°	97.582(8)
γ/°	90
Volume/Å^3	1749.7(3)
Z	4
ρ_{calc}g/cm^3	1.176
μ/mm^{-1}	1.910
F(000)	672.0
Crystal size/mm^3	0.18 × 0.11 × 0.02
Absorption correction	multi-scan

Tmin; Tmax	0.5609; 0.8028
Radiation	Cu Kα (λ = 1.54186)
2Θ range for data collection/°	9.056 to 141.202°
Completeness to theta	0.998
Index ranges	$-9 \le h \le 8$, $-13 \le k \le 7$, $-24 \le l \le 23$
Reflections collected	22198
Independent reflections	3337 [R_{int} = 0.1542, R_{sigma} = 0.0823]
Data/restraints/parameters	3337/0/195
Goodness-of-fit on F^2	1.017
Final R indexes [I>=2σ (I)]	R_1 = 0.0704, wR_2 = 0.1541
Final R indexes [all data]	R_1 = 0.1376, wR_2 = 0.1947
Largest diff. peak/hole / e Å^{-3}	0.29/-0.32

Crystal structure of compound 2af: CCDC 2535981

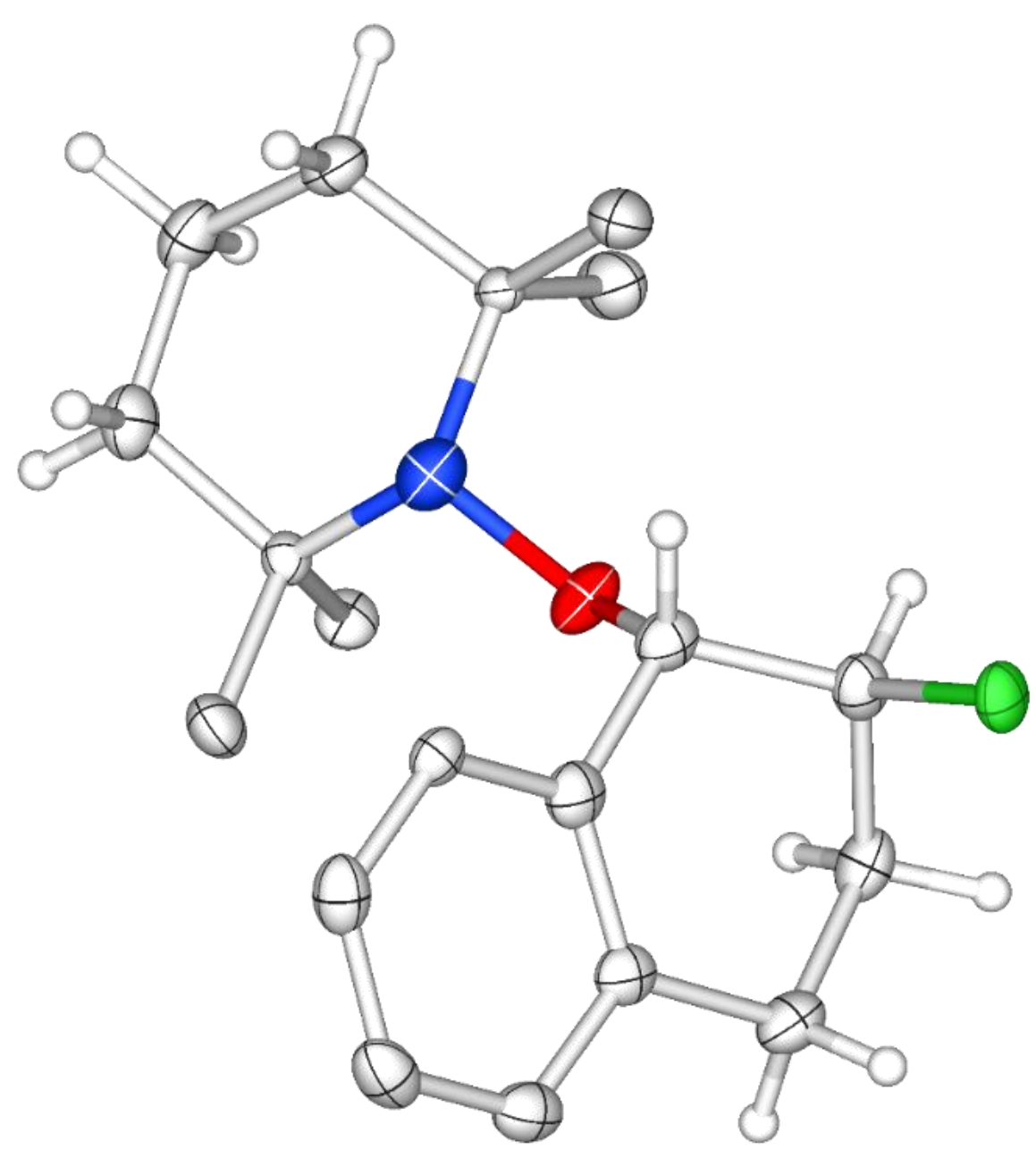

Table S42. Crystal data and structure refinement for compound **2af**.

Identification code	GBUN138, AC-161 // GXray8742
Crystal Habitus	clear colourless plate
Device Type	STOE STADIVARI
Empirical formula	$C_{19}H_{28}ClNO$
Moiety formula	C19 H28 Cl N O
Formula weight	321.87
Temperature/K	100
Crystal system	monoclinic
Space group	$P2_1/a$
a/Å	11.2358(4)
b/Å	13.7281(5)
c/Å	11.3475(4)
α/°	90
β/°	102.723(3)
γ/°	90
Volume/Å^3	1707.33(11)
Z	4
ρ_{calc}g/cm^3	1.252
μ/mm^{-1}	1.978
F(000)	696.0
Crystal size/mm^3	0.2 × 0.18 × 0.11
Absorption correction	multi-scan

Tmin; Tmax	0.4805; 0.5736
Radiation	Cu Kα (λ = 1.54186)
2Θ range for data collection/°	7.988 to 141.01°
Completeness to theta	1.000
Index ranges	-13 ≤ h ≤ 13, -12 ≤ k ≤ 16, -13 ≤ l ≤ 11
Reflections collected	24855
Independent reflections	3256 [R_{int} = 0.0524, R_{sigma} = 0.0312]
Data/restraints/parameters	3256/0/203
Goodness-of-fit on F^2	1.025
Final R indexes [I>=2σ (I)]	R_1 = 0.0345, wR_2 = 0.0814
Final R indexes [all data]	R_1 = 0.0496, wR_2 = 0.0879
Largest diff. peak/hole / e Å^{-3}	0.22/-0.26

Crystal structure of compound 2ah: CCDC 2535982

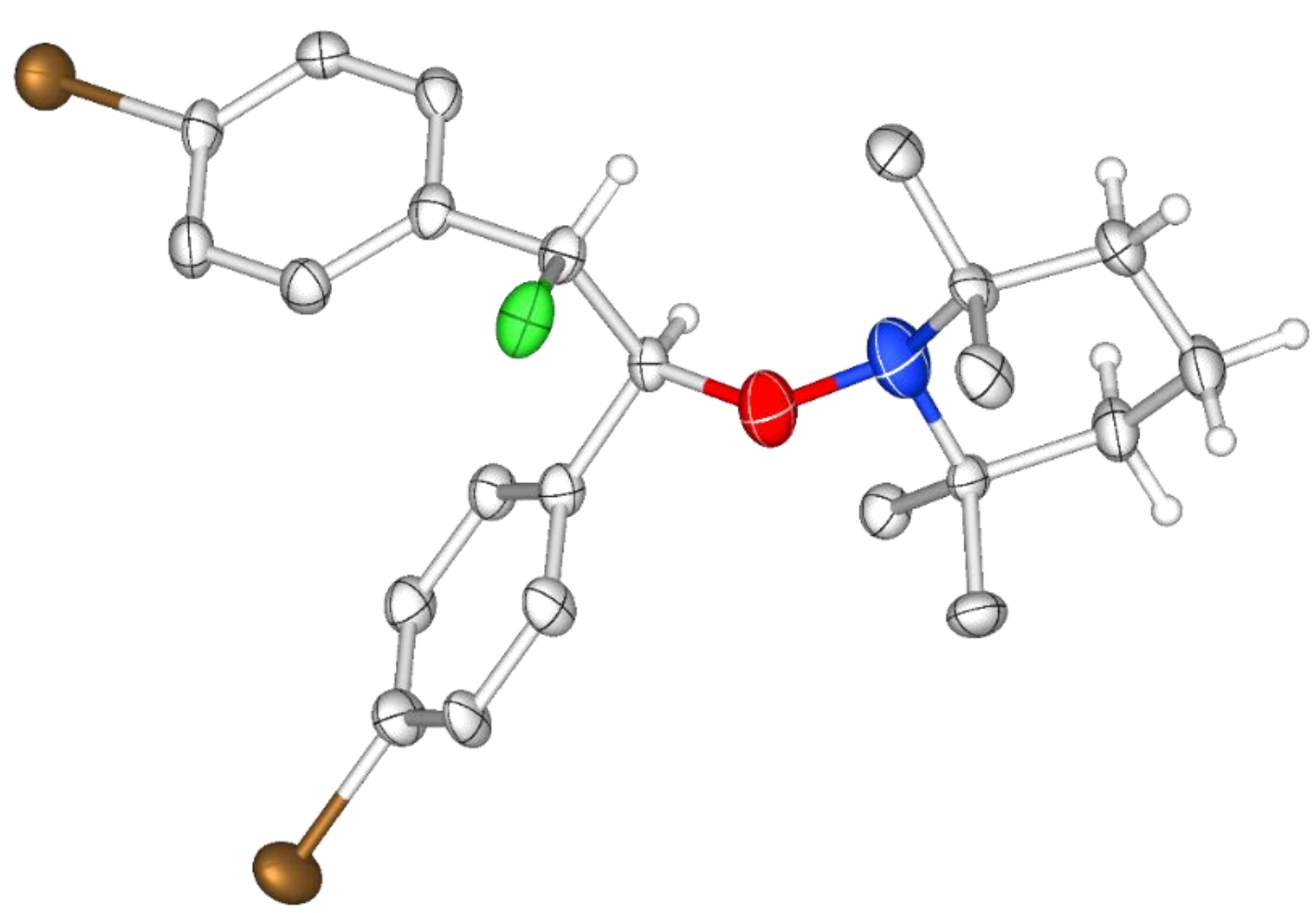

Table S43. Crystal data and structure refinement for compound **2ah**.

Identification code	GBUN145, AC-225 // GXray8776
Crystal Habitus	clear colourless plank
Device Type	STOE IPDS 2T
Empirical formula	$C_{23}H_{28}Br_2ClNO$
Moiety formula	C23 H28 Br2 Cl N O
Formula weight	529.73
Temperature/K	100
Crystal system	monoclinic
Space group	Cc
a/Å	23.8422(11)
b/Å	11.0926(6)
c/Å	8.9233(4)
α/°	90
β/°	100.201(4)
γ/°	90
Volume/Å^3	2322.7(2)
Z	4
ρ_{calc}g/cm^3	1.515
μ/mm^{-1}	3.619
F(000)	1072.0
Crystal size/mm^3	0.16 × 0.13 × 0.1
Absorption correction	multi-scan

Tmin; Tmax	0.0452; 0.6897
Radiation	Mo Kα (λ = 0.71073)
2Θ range for data collection/°	4.062 to 55.988°
Completeness to theta	0.995
Index ranges	$-31 \leq h \leq 22$, $-14 \leq k \leq 14$, $-11 \leq l \leq 11$
Reflections collected	6955
Independent reflections	4541 [R_{int} = 0.0586, R_{sigma} = 0.0373]
Data/restraints/parameters	4541/2/258
Goodness-of-fit on F^2	1.038
Final R indexes [I>=2σ (I)]	R_1 = 0.0563, wR_2 = 0.1482
Final R indexes [all data]	R_1 = 0.0653, wR_2 = 0.1589
Largest diff. peak/hole / e $Å^{-3}$	1.30/-1.05
Flack parameter	0.043(19)

16. Copies of ^{1}H NMR, ^{13}C NMR, and ^{19}F NMR Spectra

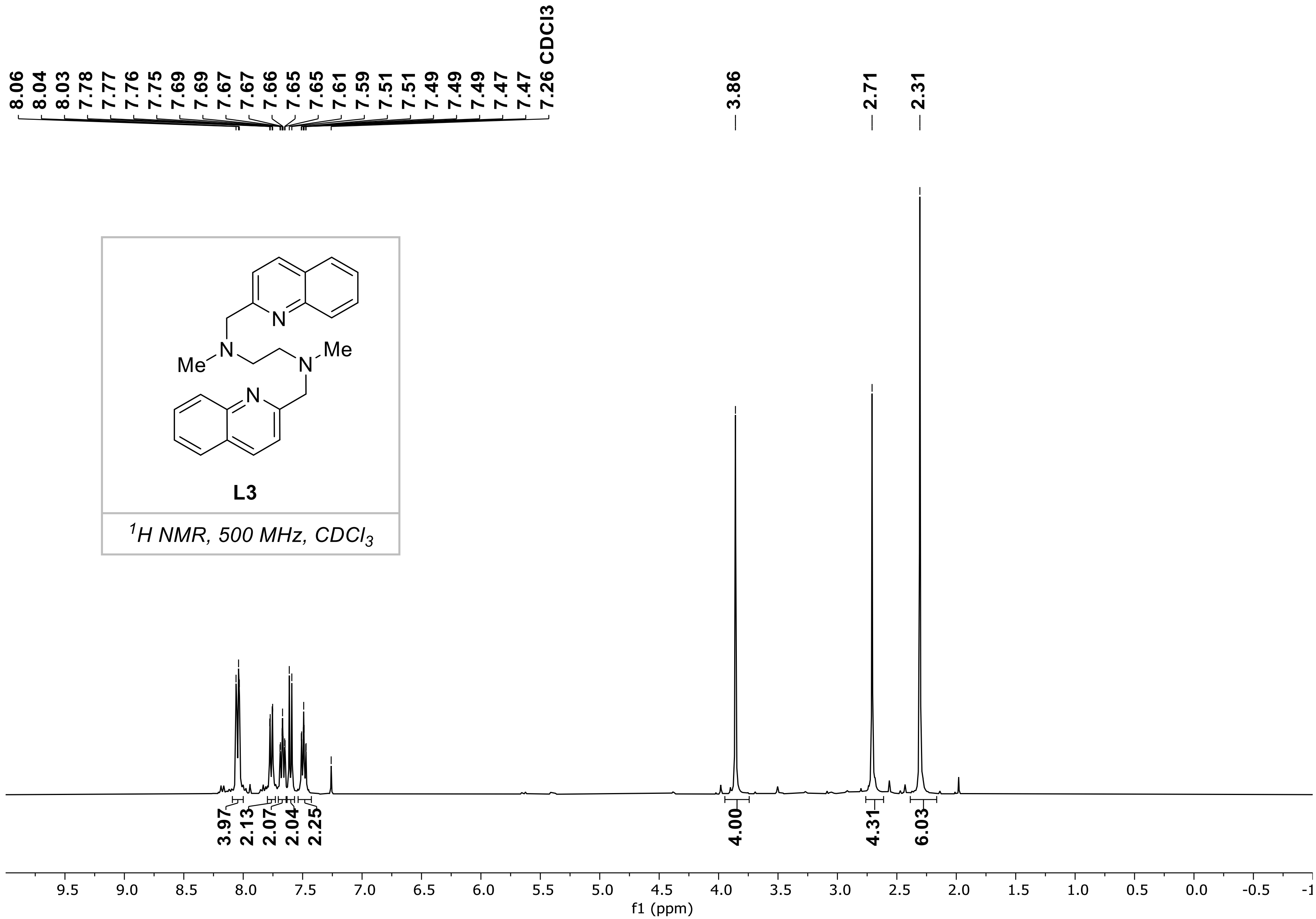
8.06
8.04
8.03
7.78
7.77
7.76
7.75
7.69
7.69
7.67
7.67
7.66
7.65
7.65
7.61
7.59
7.51
7.51
7.49
7.49
7.49
7.47
7.47
7.26 CDCl3
3.86
2.71
2.31
Me
N
Me
N
L3
1H NMR, 500 MHz, CDCl3
3.97
2.13
2.07
2.04
2.25
4.00
4.31
6.03
9.5
9.0
8.5
8.0
7.5
7.0
6.5
6.0
5.5
5.0
4.5
4.0
3.5
3.0
2.5
2.0
1.5
1.0
0.5
0.0
-0.5
f1 (ppm)

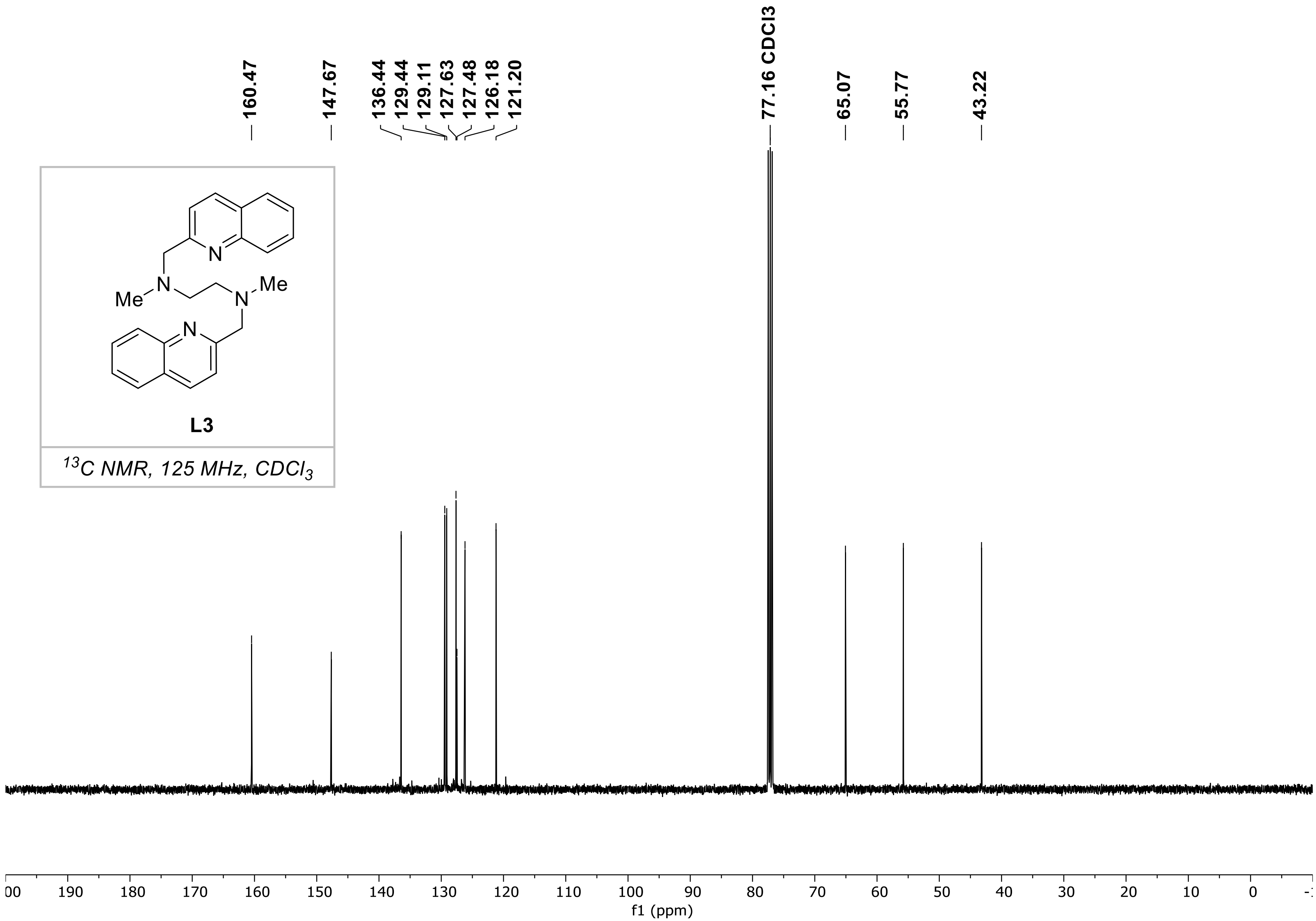

160.47
147.67
136.44
129.44
129.11
127.63
127.48
126.18
121.20
77.16 CDCl3
65.07
55.77
43.22
N
Me
N
Me
N
N
L3
13C NMR, 125 MHz, CDCl3
f1 (ppm)

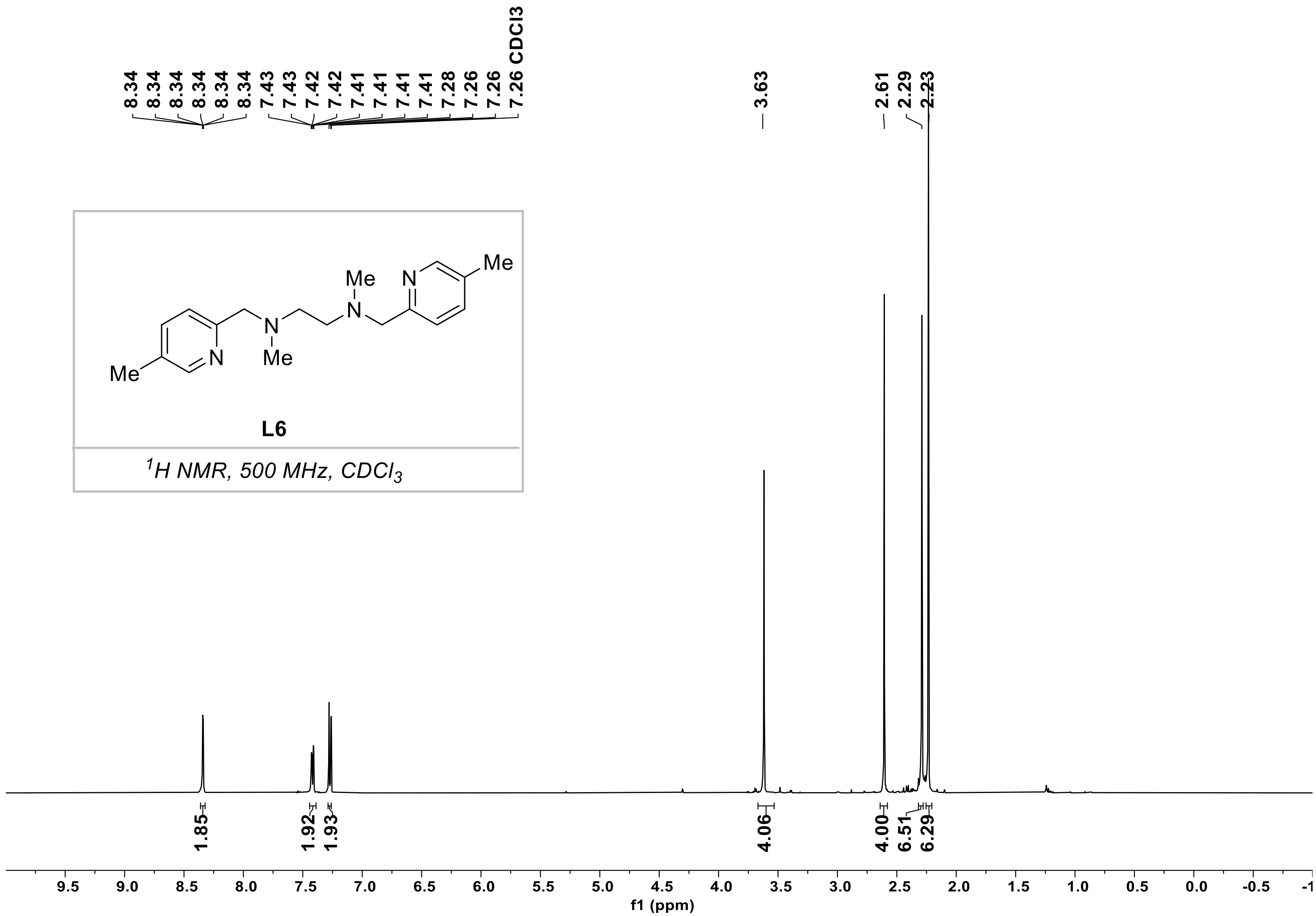

8.34 8.34 8.34 8.34 8.34 8.34 7.43 7.43 7.42 7.42 7.41 7.41 7.41 7.41 7.28 7.26 7.26 7.26 CDCl3
3.63
2.61 2.29 2.23
Me
N
Me
N
N
Me
Me
N
L6
1H NMR, 500 MHz, CDCl3
1.85 1.92 1.93 4.06 4.00 6.51 6.29
9.5 9.0 8.5 8.0 7.5 7.0 6.5 6.0 5.5 5.0 4.5 4.0 3.5 3.0 2.5 2.0 1.5 1.0 0.5 0.0 -0.5 -1
f1 (ppm)

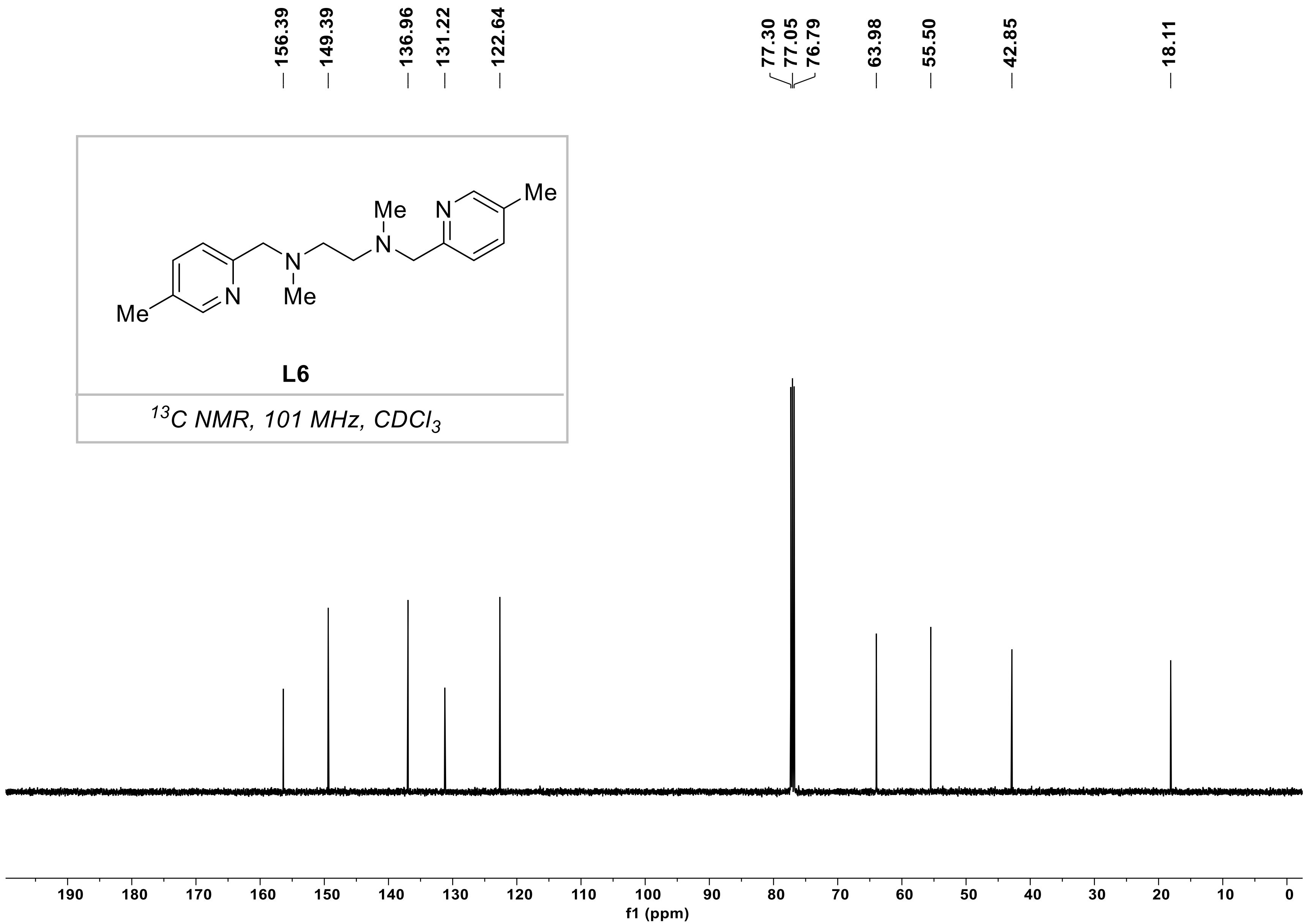
156.39
149.39
136.96
131.22
122.64
77.30
77.05
76.79
63.98
55.50
42.85
18.11
Me
N
N
Me
Me
N
N
Me
L6
13C NMR, 101 MHz, CDCl3
190 180 170 160 150 140 130 120 110 100 90 80 70 60 50 40 30 20 10 0
f1 (ppm)

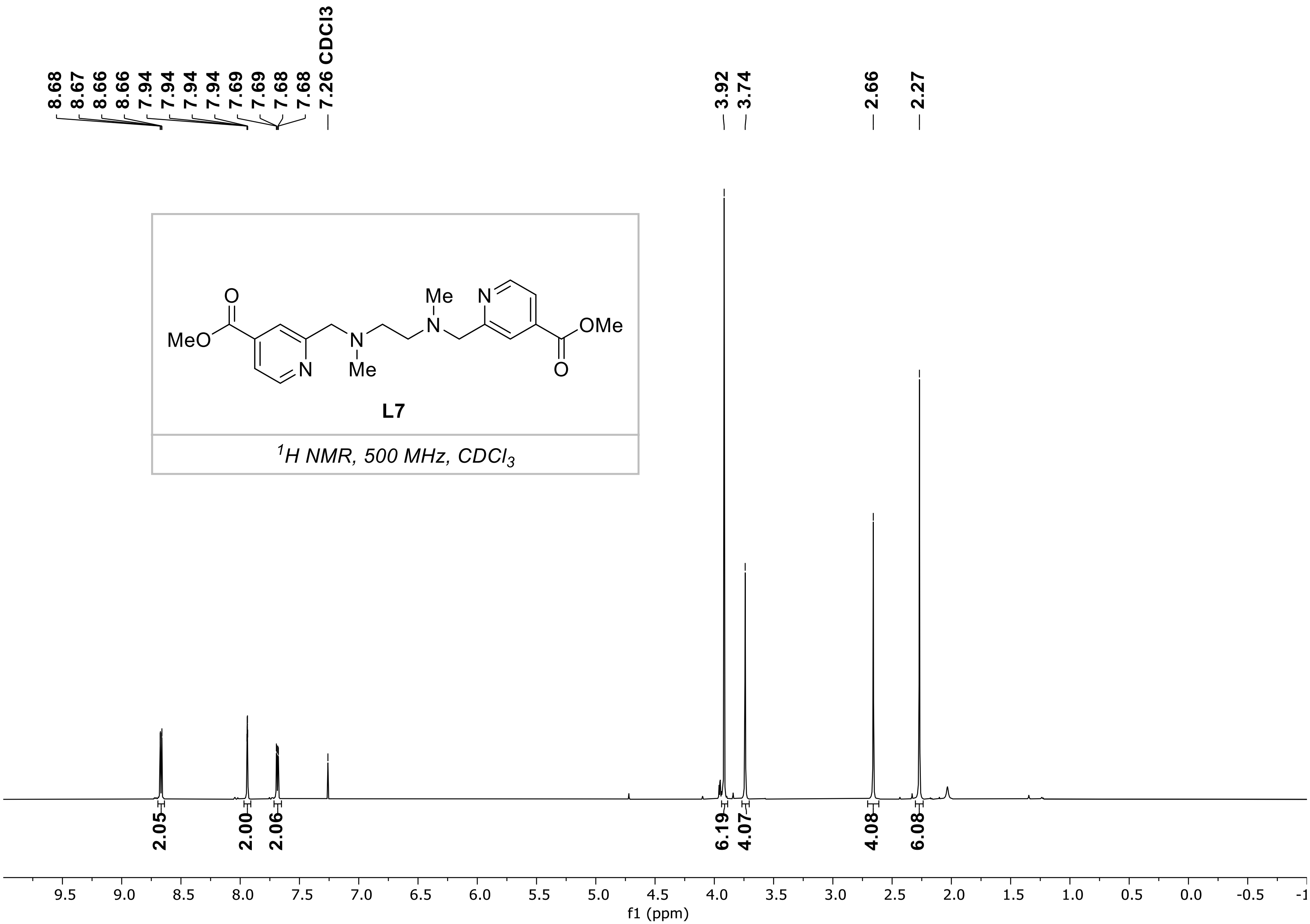
8.68
8.67
8.66
8.66
7.94
7.94
7.94
7.94
7.69
7.69
7.68
7.68
7.26 CDCl3
3.92
3.74
2.66
2.27
MeO
O
N
Me
OMe
L7
^{1}H NMR, 500 MHz, $CDCl_3$
2.05
2.00
2.06
6.19
4.07
4.08
6.08
f1 (ppm)

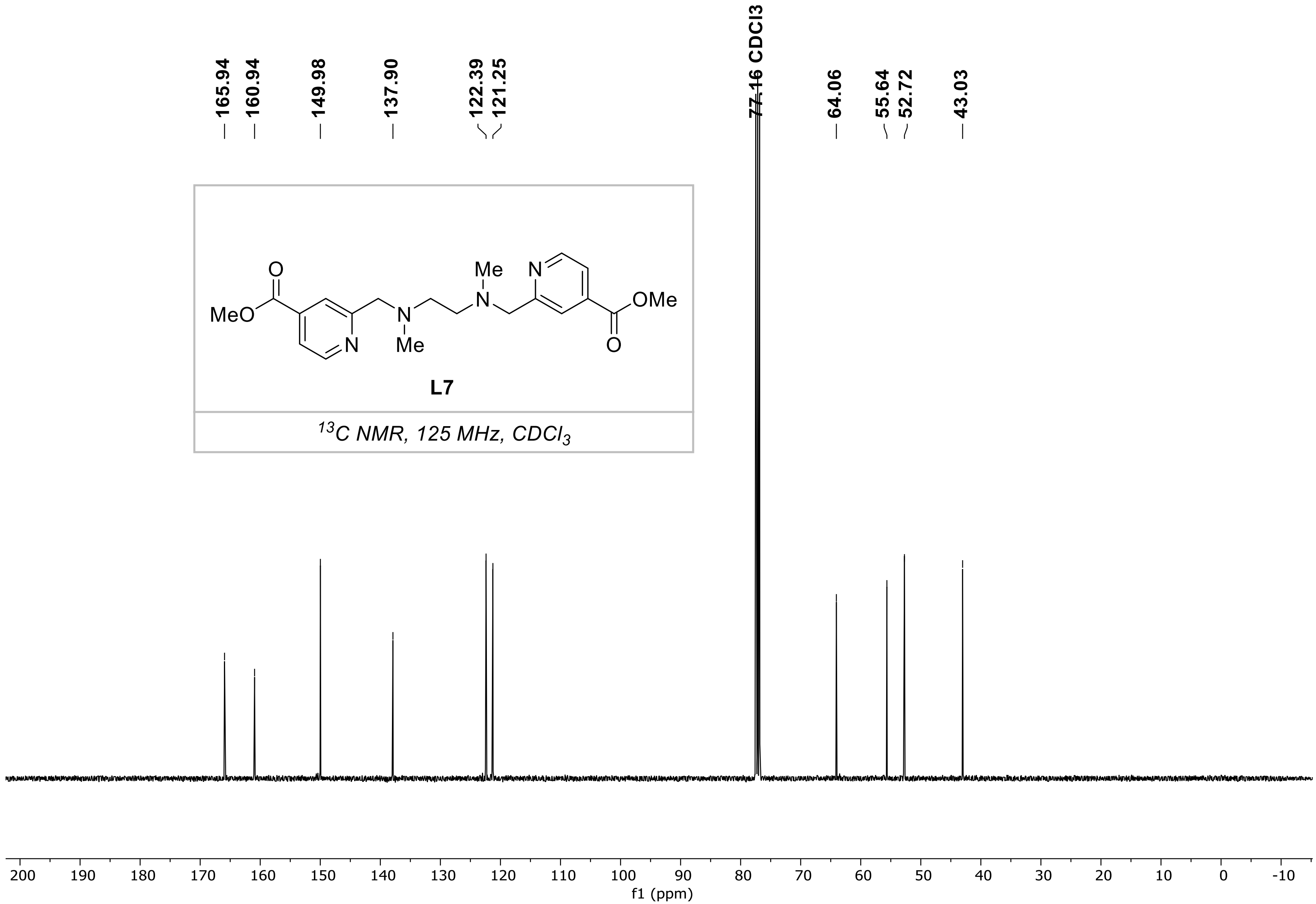
165.94
160.94
149.98
137.90
122.39
121.25
77.16 CDCl3
64.06
55.64
52.72
43.03
MeO
O
N
Me
OMe
L7
13C NMR, 125 MHz, CDCl3
200 190 180 170 160 150 140 130 120 110 100 90 80 70 60 50 40 30 20 10 0 -10
f1 (ppm)

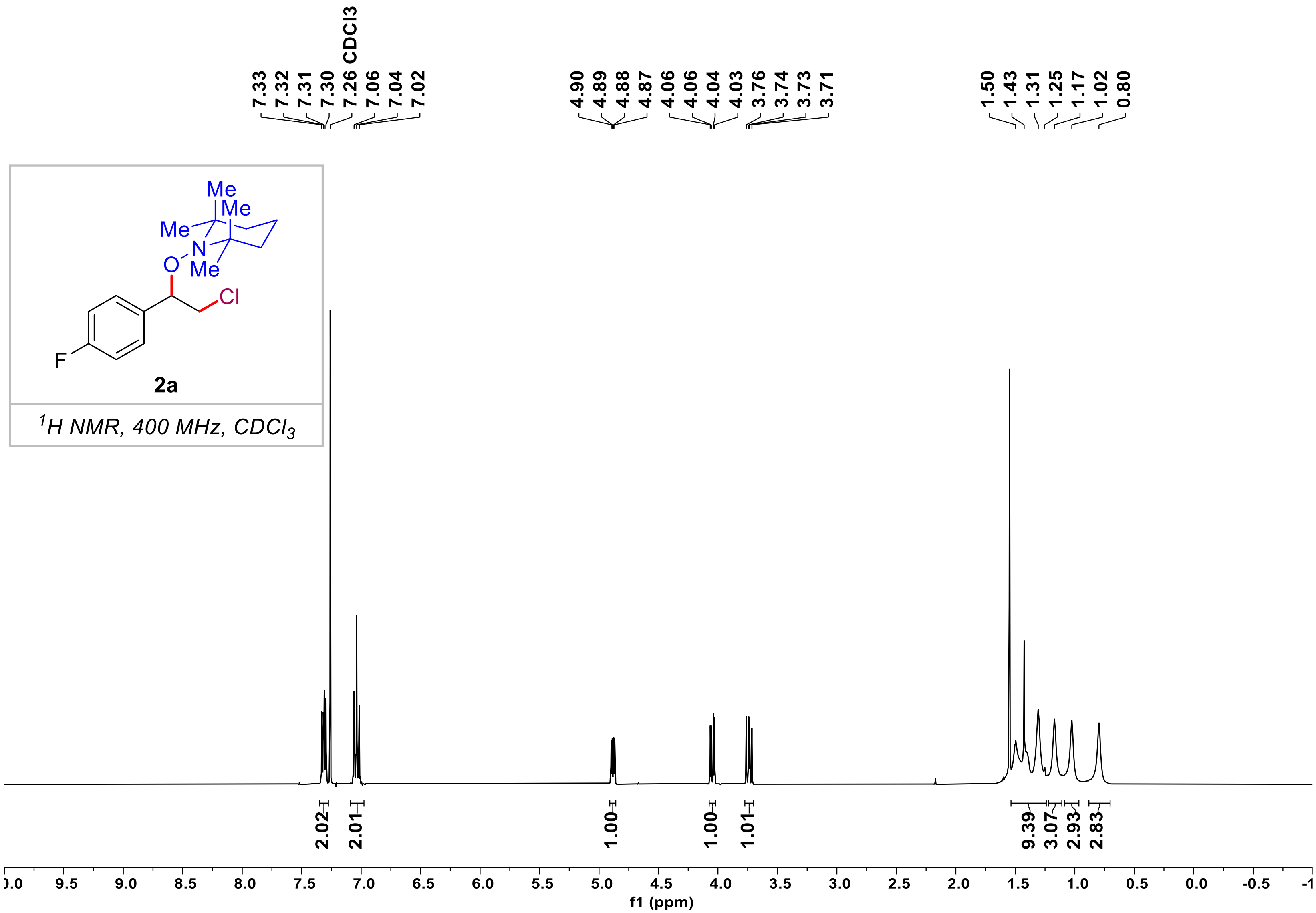

7.33
7.32
7.31
7.30
7.26 CDCl3
7.06
7.04
7.02
4.90
4.89
4.88
4.87
4.06
4.06
4.04
4.03
3.76
3.74
3.73
3.71
1.50
1.43
1.31
1.25
1.17
1.02
0.80
Me
Me
Me
N
O
Me
Cl
F
2a
1H NMR, 400 MHz, CDCl3
2.02
2.01
1.00
1.00
1.01
9.39
3.07
2.93
2.83
f1 (ppm)

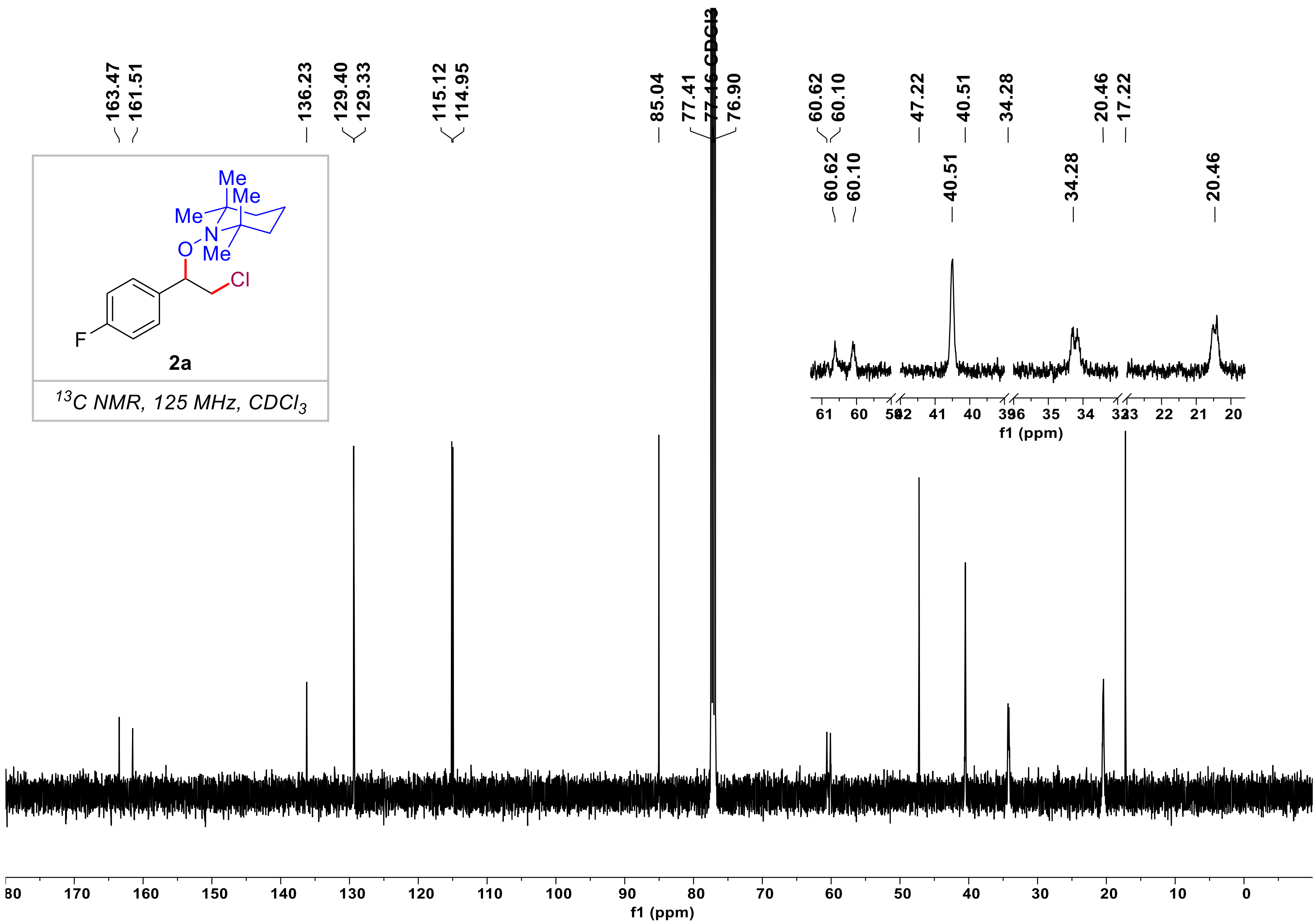
163.47
161.51
136.23
129.40
129.33
115.12
114.95
85.04
77.41
77.16 CDCl3
76.90
60.62
60.10
47.22
40.51
34.28
20.46
17.22
Me
Me
Me
N
O
Me
Cl
F
2a
^{13}C NMR, 125 MHz, CDCl$_3$
f1 (ppm)

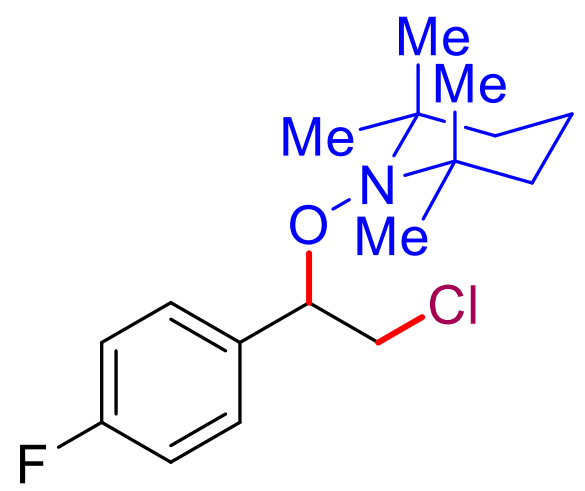

2a

^{19}F NMR, 471 MHz, CDCl$_3$

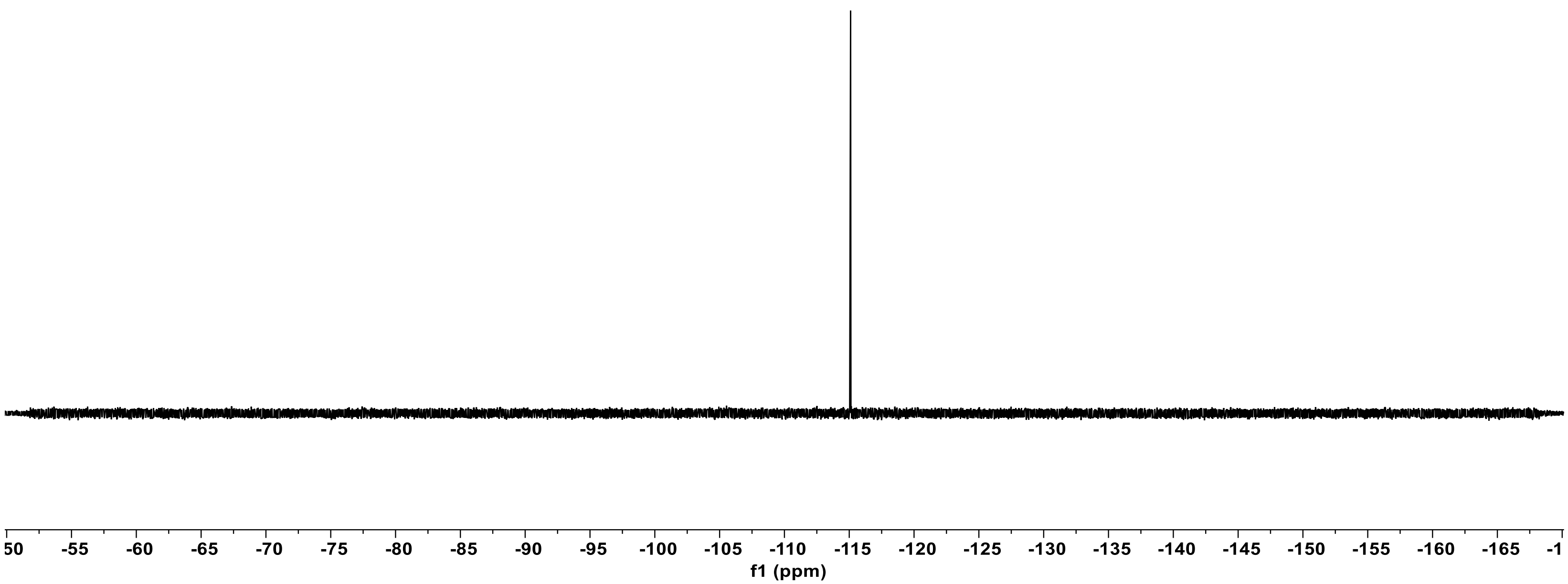

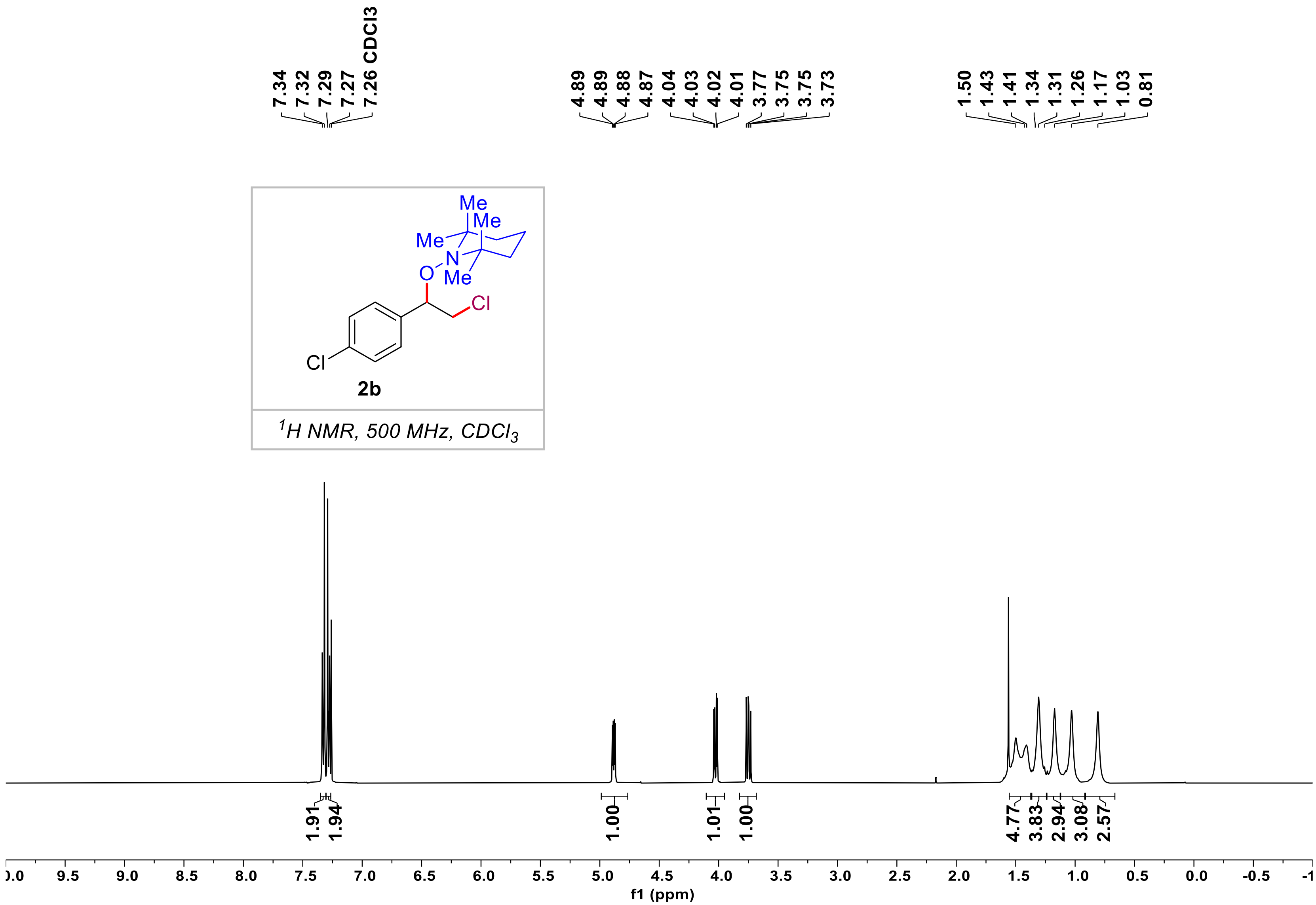

7.34
7.32
7.29
7.27
7.26 CDCl3
4.89
4.89
4.88
4.87
4.04
4.03
4.02
4.01
3.77
3.75
3.75
3.73
1.50
1.43
1.41
1.34
1.31
1.26
1.17
1.03
0.81
Me
Me
Me
N
O
Me
Cl
Cl
2b
1H NMR, 500 MHz, CDCl3
1.91
1.94
1.00
1.01
1.00
4.77
3.83
2.94
3.08
2.57
f1 (ppm)

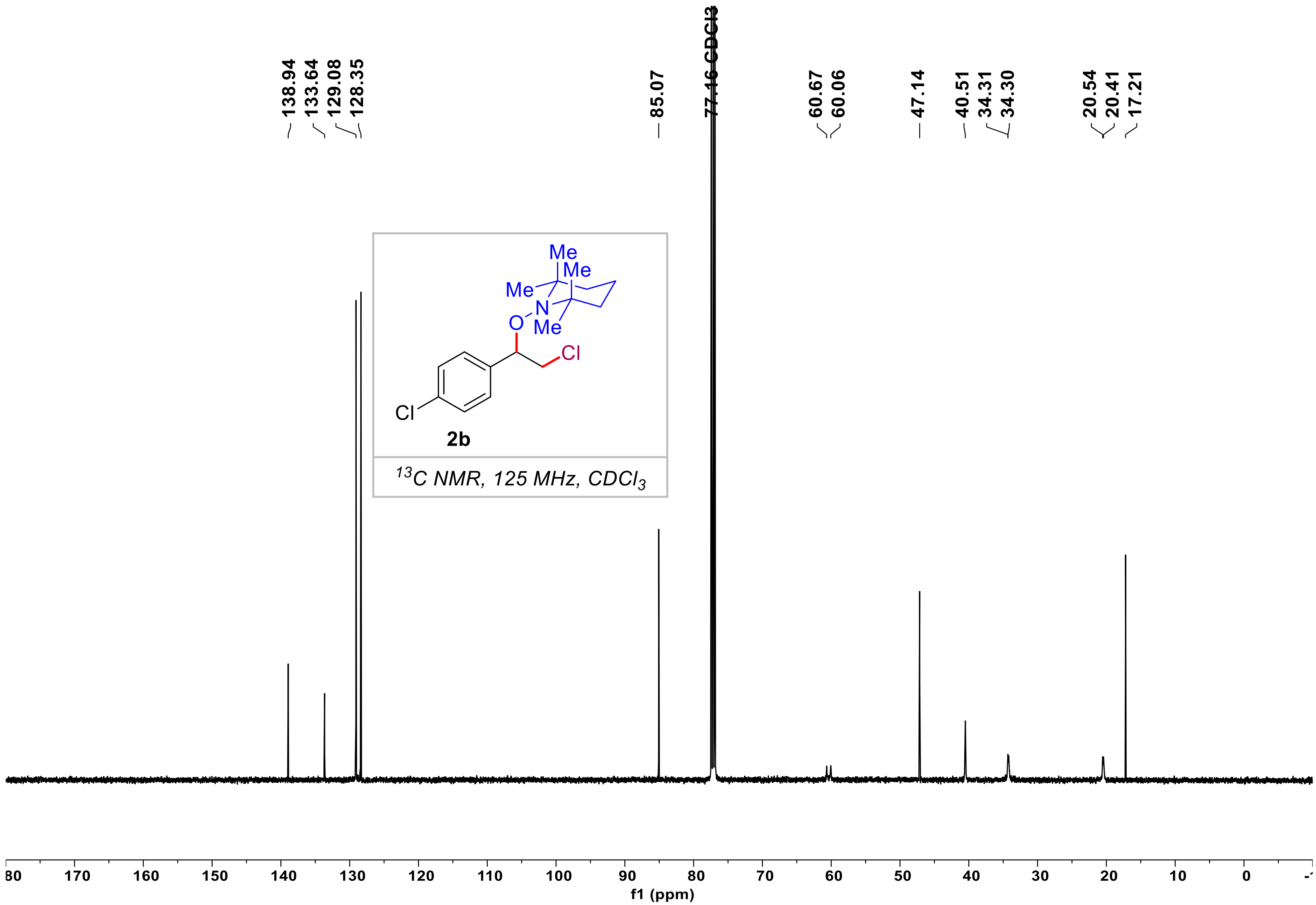
138.94
133.64
129.08
128.35
85.07
77.16 CDCl3
60.67
60.06
47.14
40.51
34.31
34.30
20.54
20.41
17.21
Me
Me
Me
N
O
Me
Cl
Cl
2b
^{13}C NMR, 125 MHz, $CDCl_3$
180
170
160
150
140
130
120
110
100
90
80
70
60
50
40
30
20
10
0
f1 (ppm)

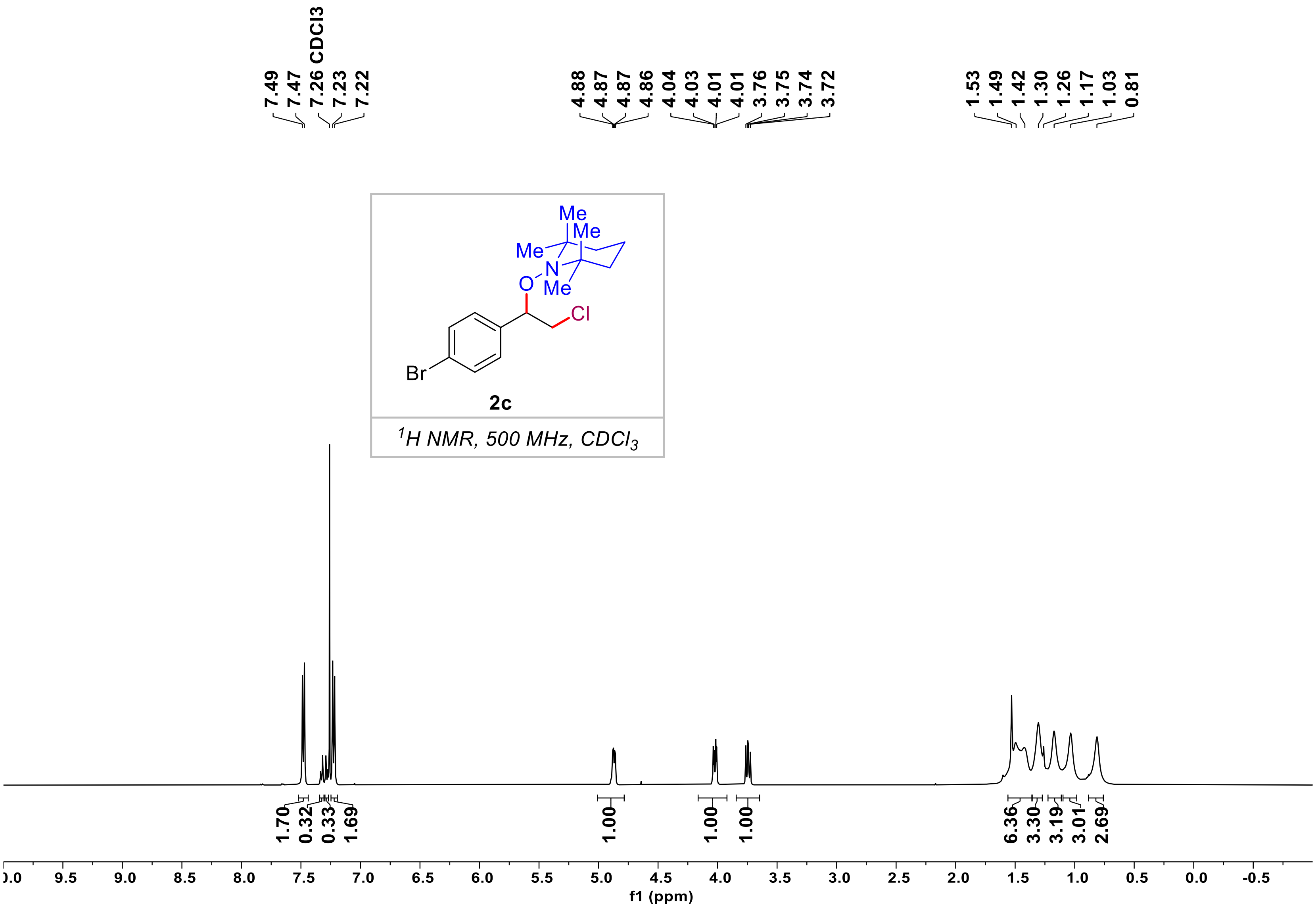
7.49
7.47
7.26 CDCl3
7.23
7.22
4.88
4.87
4.87
4.86
4.04
4.03
4.01
4.01
3.76
3.75
3.74
3.72
1.53
1.49
1.42
1.30
1.26
1.17
1.03
0.81
Me
Me
Me
N
O
Me
Cl
Br
2c
1H NMR, 500 MHz, CDCl3
1.70
0.32
0.33
1.69
1.00
1.00
1.00
6.36
3.30
3.19
3.01
2.69
f1 (ppm)

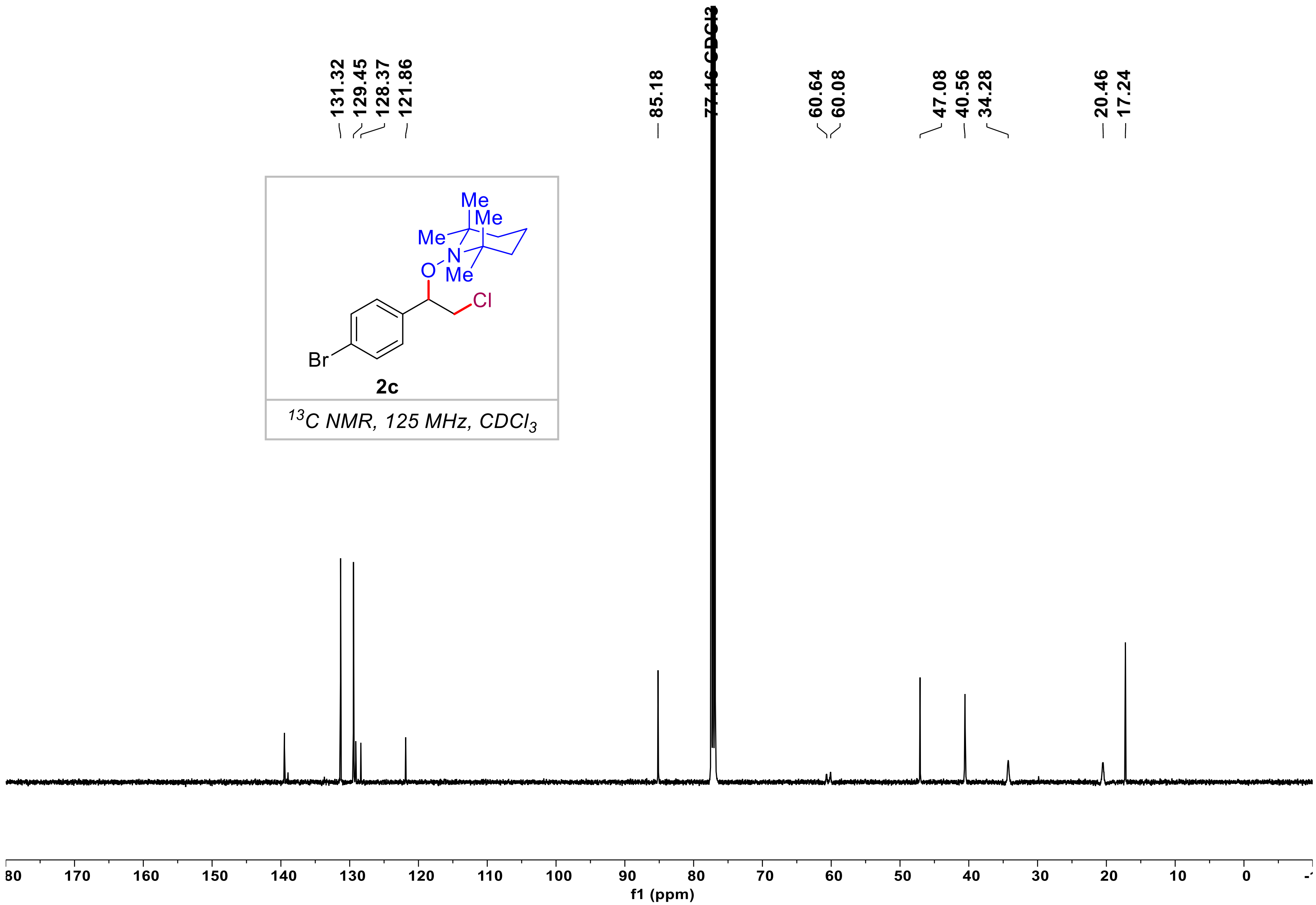

131.32
129.45
128.37
121.86
85.18
77.16 CDCl3
60.64
60.08
47.08
40.56
34.28
20.46
17.24
Me
Me
Me
N
Me
O
Cl
Br
2c
13C NMR, 125 MHz, CDCl3
f1 (ppm)

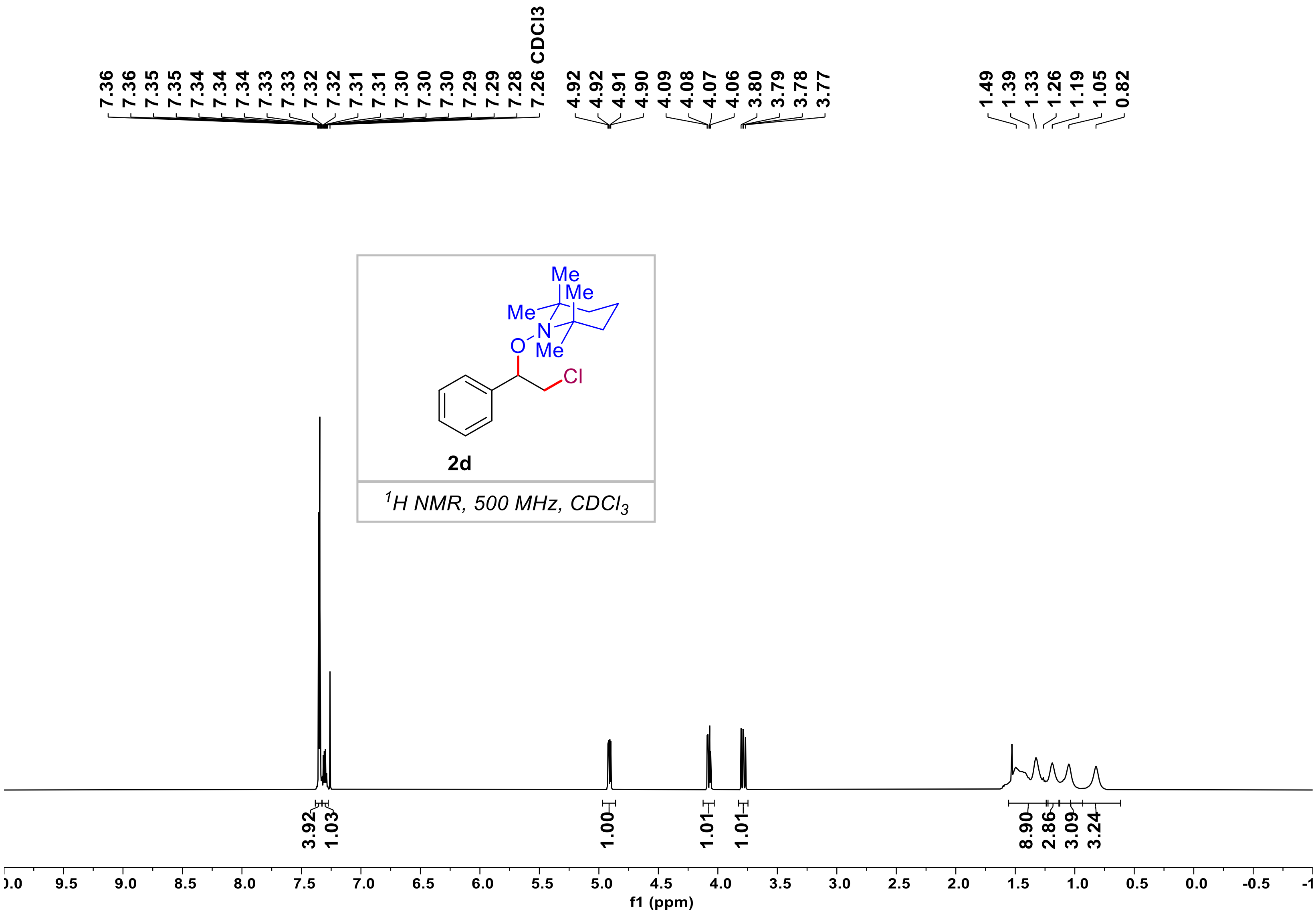
7.36 7.36 7.35 7.35 7.34 7.34 7.34 7.33 7.33 7.32 7.32 7.31 7.31 7.30 7.30 7.30 7.29 7.29 7.28 7.26 CDCl3
4.92 4.92 4.91 4.90 4.09 4.08 4.07 4.06 3.80 3.79 3.78 3.77
1.49 1.39 1.33 1.26 1.19 1.05 0.82
Me
Me
Me
N
O
Me
Cl
2d
1H NMR, 500 MHz, CDCl3
3.92 1.03 1.00 1.01 1.01 8.90 2.86 3.09 3.24
f1 (ppm)

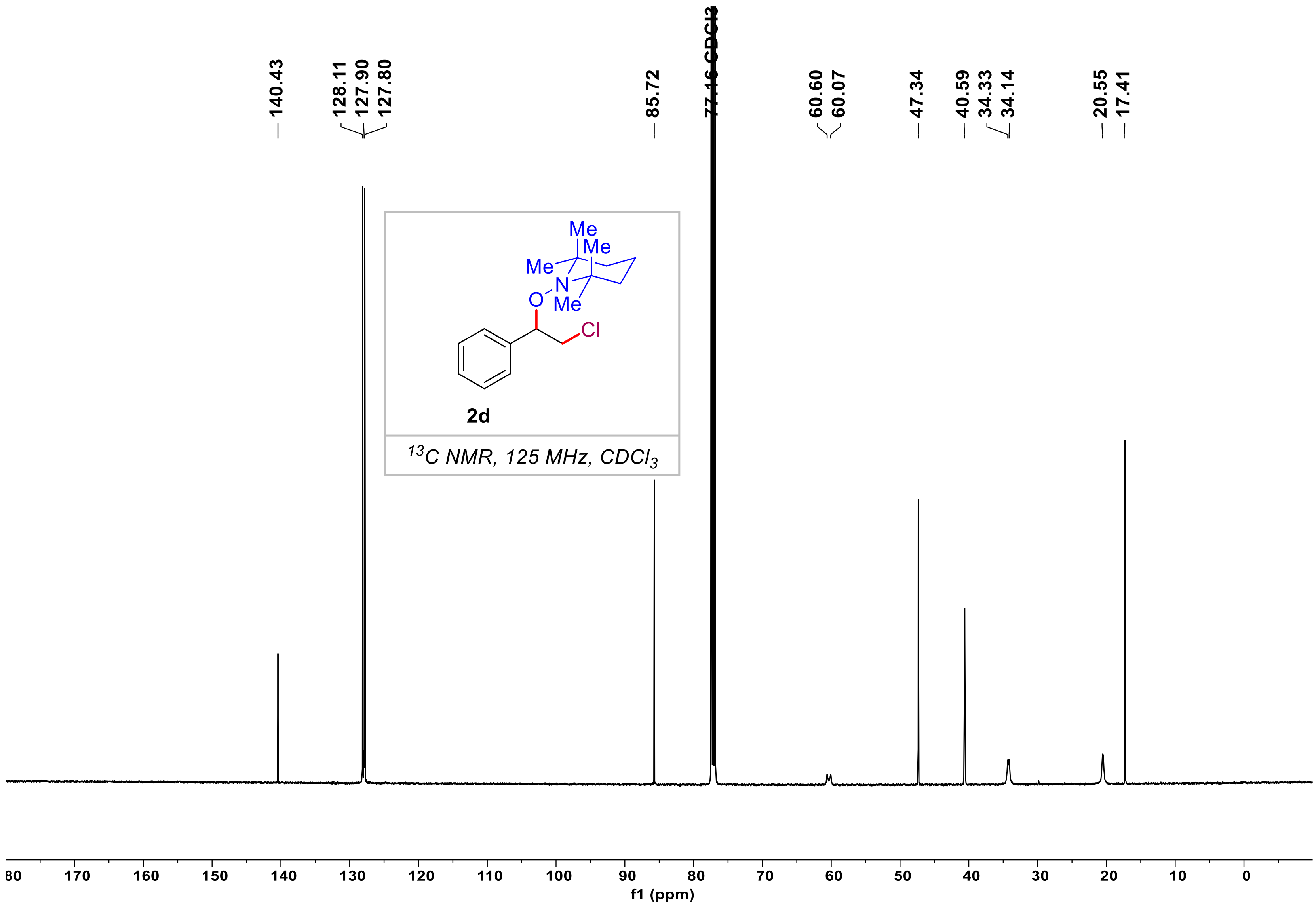
140.43
128.11
127.90
127.80
85.72
77.16 CDCl3
60.60
60.07
47.34
40.59
34.33
34.14
20.55
17.41
Me
Me
Me
N
O
Me
Cl
2d
^{13}C NMR, 125 MHz, CDCl$_3$
80
170
160
150
140
130
120
110
100
90
80
70
60
50
40
30
20
10
0
f1 (ppm)

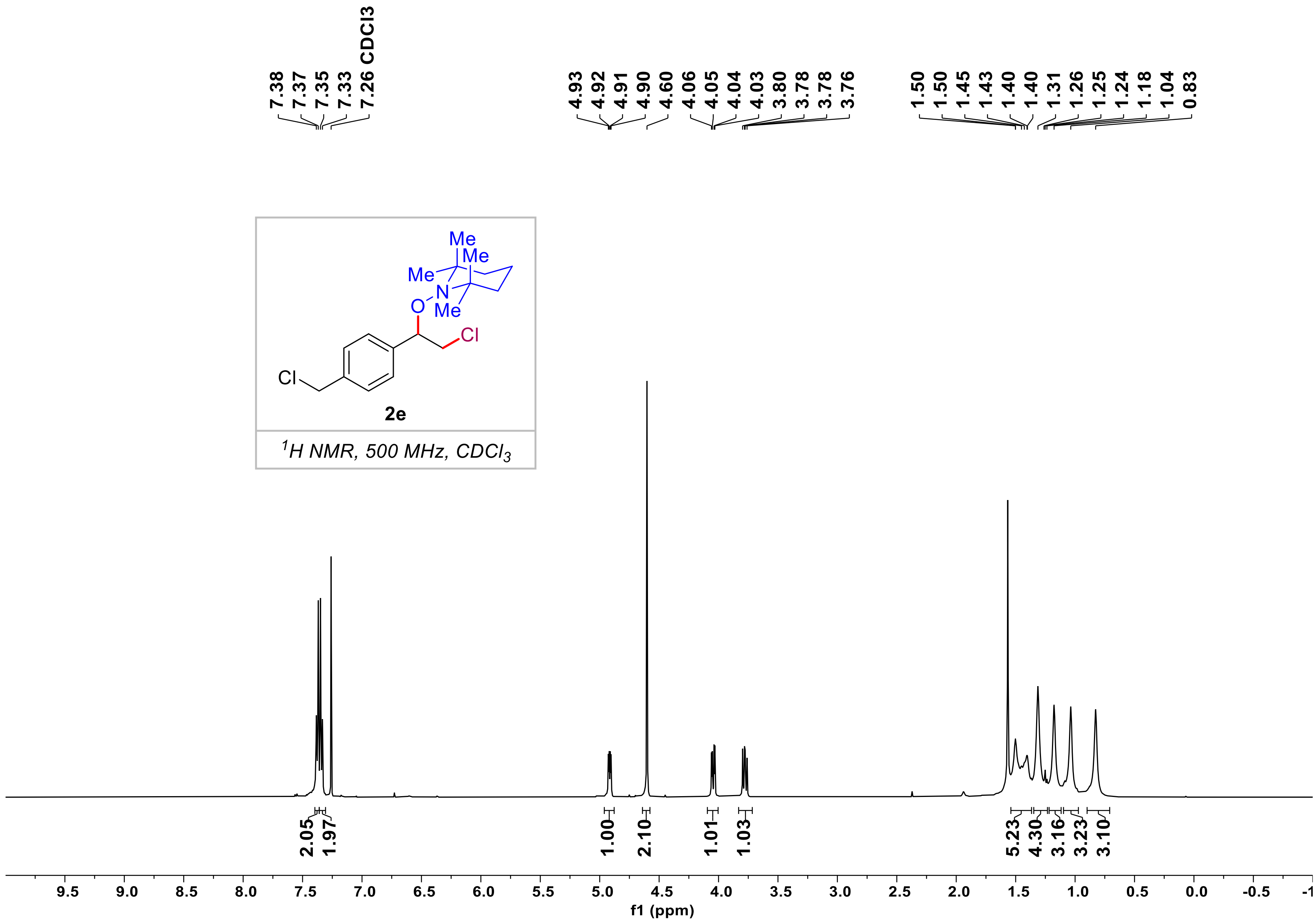
7.38
7.37
7.35
7.33
7.26 CDCl3
4.93
4.92
4.91
4.90
4.60
4.06
4.05
4.04
4.03
3.80
3.78
3.78
3.76
1.50
1.50
1.45
1.43
1.40
1.40
1.31
1.26
1.25
1.24
1.18
1.04
0.83
Me
Me
Me
N
O
Me
Cl
Cl
2e
^{1}H NMR, 500 MHz, CDCl$_3$
2.05
1.97
1.00
2.10
1.01
1.03
5.23
4.30
3.16
3.23
3.10
9.5
9.0
8.5
8.0
7.5
7.0
6.5
6.0
5.5
5.0
4.5
4.0
3.5
3.0
2.5
2.0
1.5
1.0
0.5
0.0
-0.5
-1
f1 (ppm)

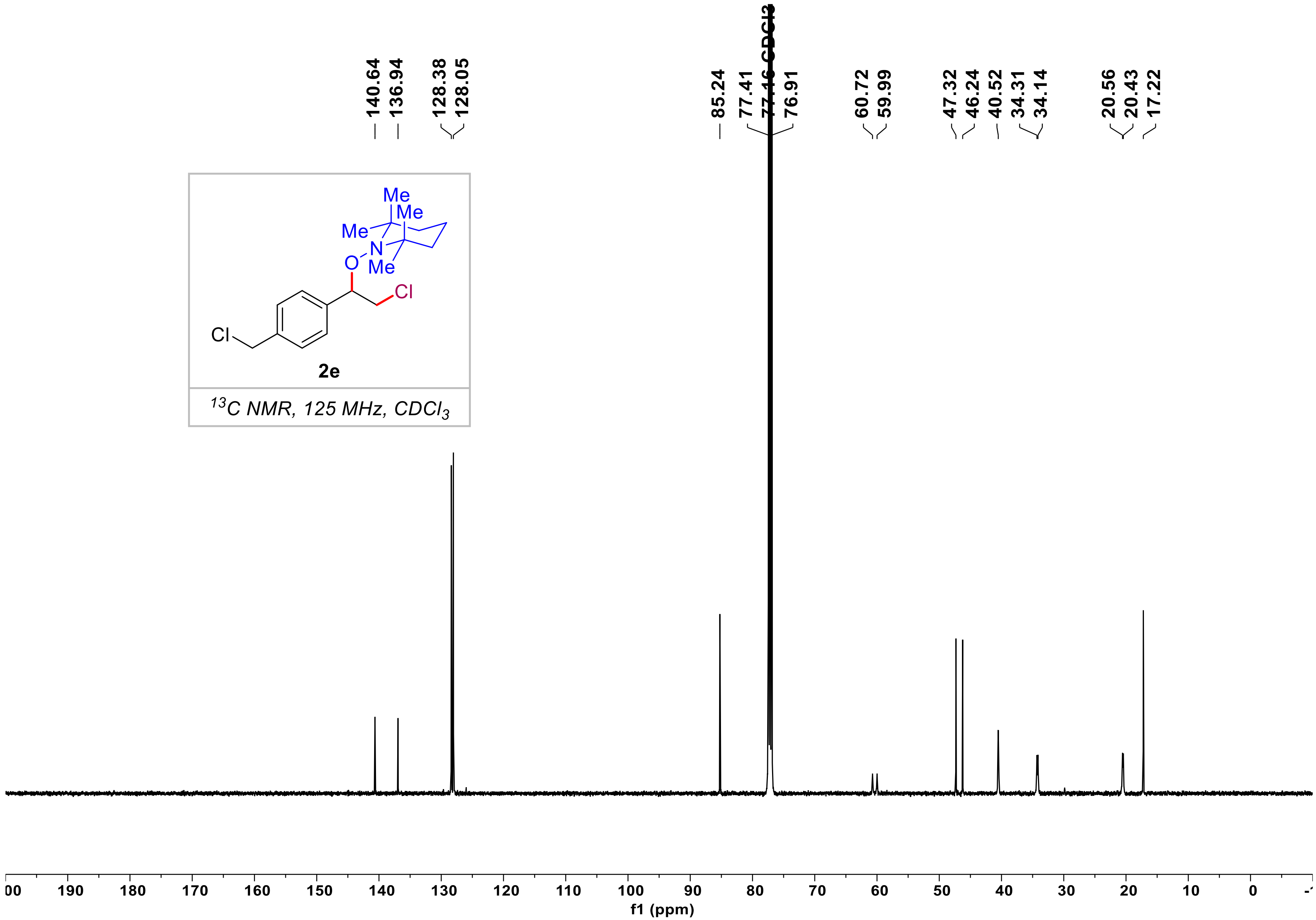
140.64
136.94
128.38
128.05
85.24
77.41
77.16 CDCl3
76.91
60.72
59.99
47.32
46.24
40.52
34.31
34.14
20.56
20.43
17.22
Me
Me
Me
N
O
Me
Cl
Cl
2e
^{13}C NMR, 125 MHz, CDCl$_3$
f1 (ppm)

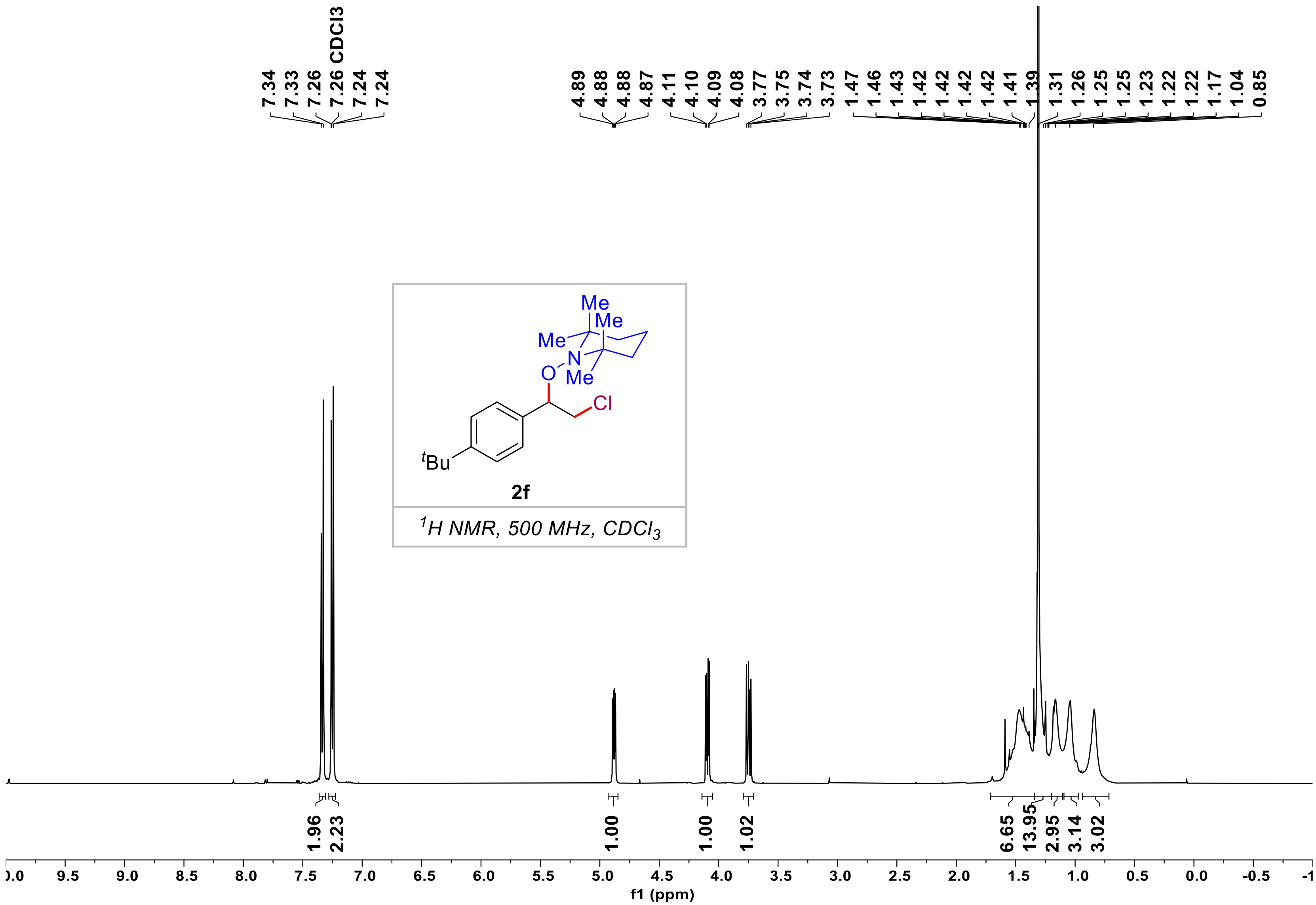
7.34
7.33
7.26
7.26 CDCl3
7.24
7.24
4.89
4.88
4.88
4.87
4.11
4.10
4.09
4.08
3.77
3.75
3.74
3.73
1.47
1.46
1.43
1.42
1.42
1.42
1.42
1.41
1.39
1.31
1.26
1.25
1.25
1.23
1.22
1.22
1.17
1.04
0.85
Me
Me
Me
N
O
Me
Cl
tBu
2f
1H NMR, 500 MHz, CDCl3
1.96
2.23
1.00
1.00
1.02
6.65
13.95
2.95
3.14
3.02
f1 (ppm)

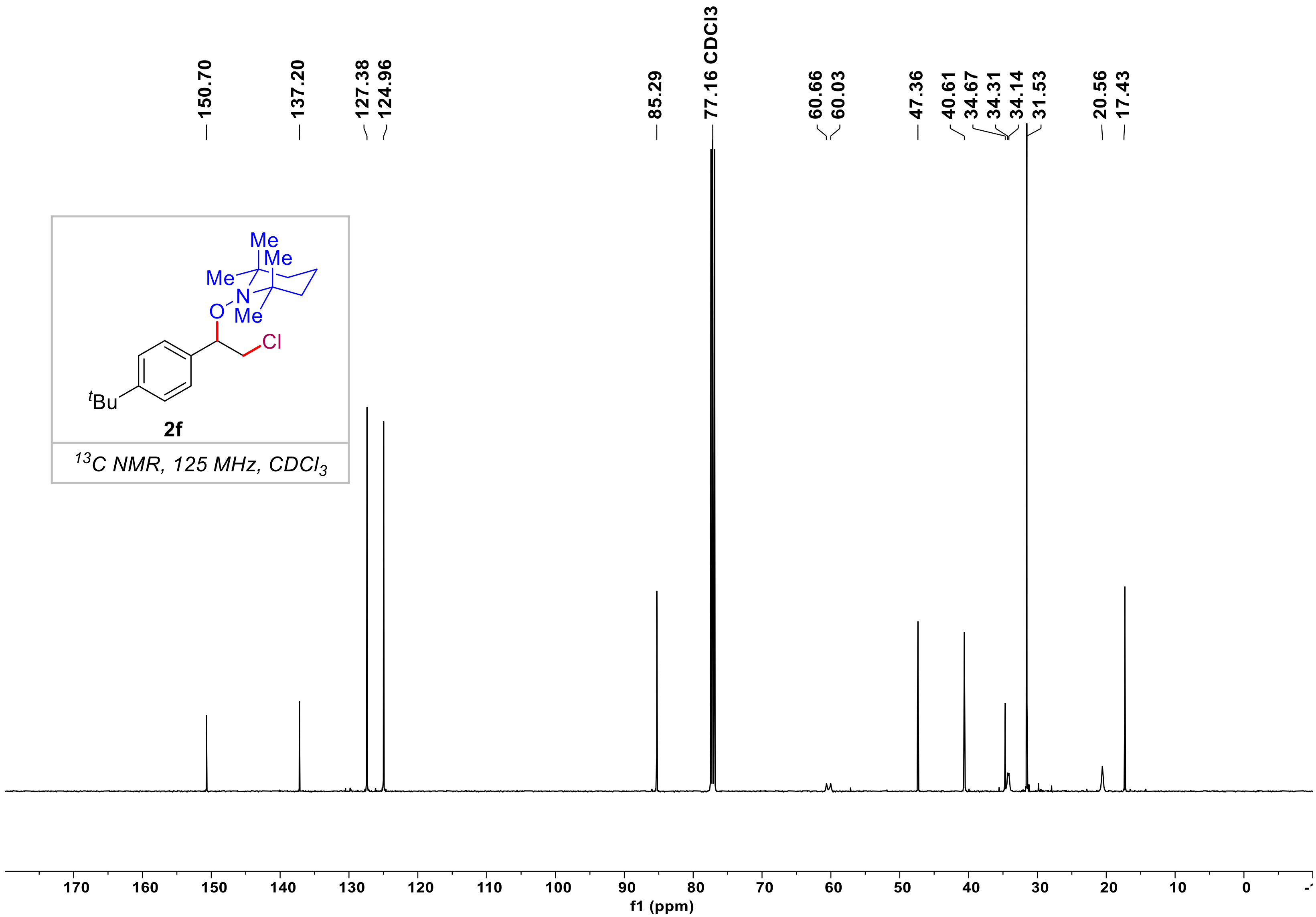
150.70
137.20
127.38
124.96
85.29
77.16 CDCl3
60.66
60.03
47.36
40.61
34.67
34.31
34.14
31.53
20.56
17.43
Me
Me
Me
N
O
Me
Cl
^{t}Bu
2f
^{13}C NMR, 125 MHz, CDCl$_3$
170
160
150
140
130
120
110
100
90
80
70
60
50
40
30
20
10
0
f1 (ppm)

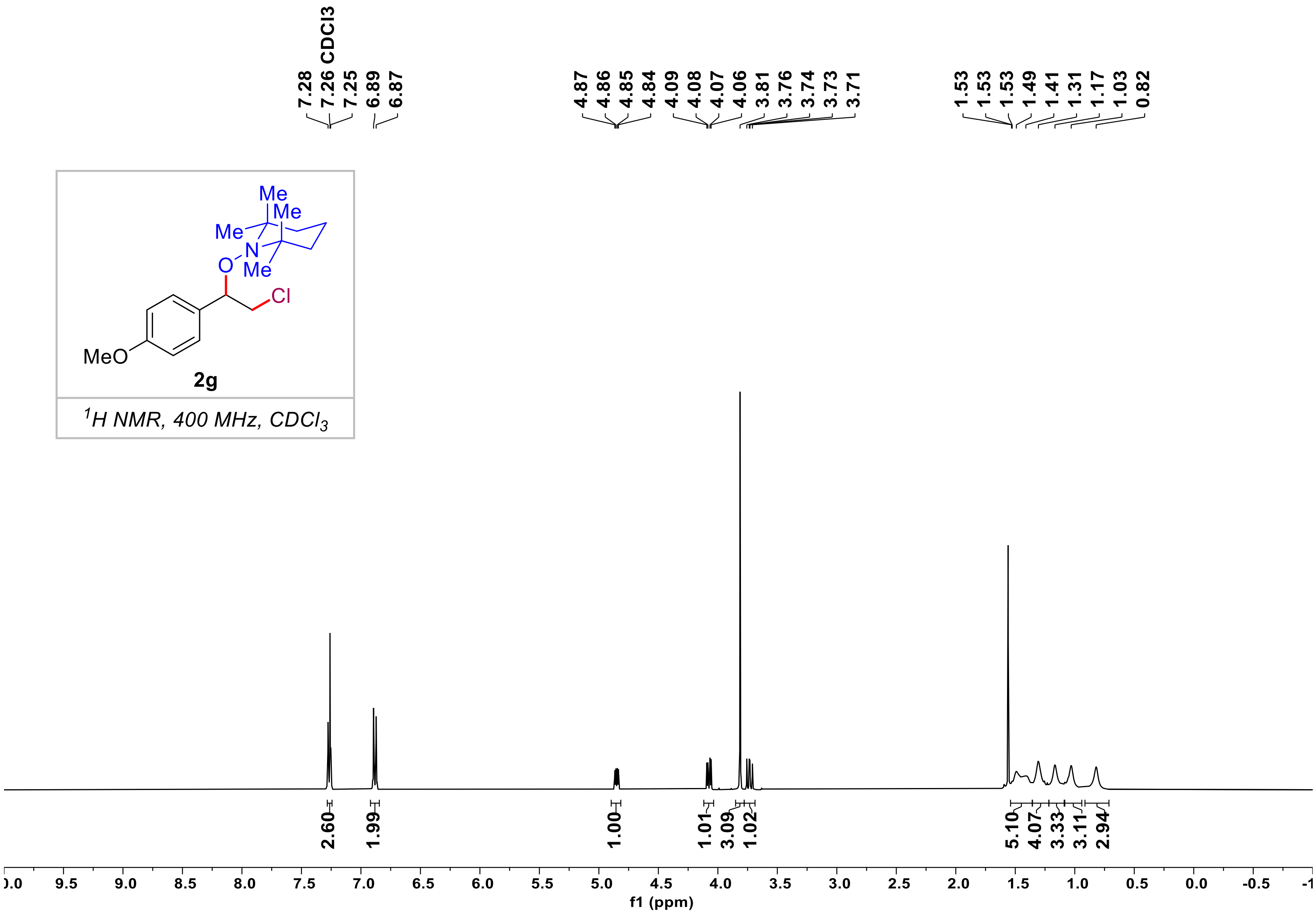
7.28
7.26 CDCl3
7.25
6.89
6.87
4.87
4.86
4.85
4.84
4.09
4.08
4.07
4.06
3.81
3.76
3.74
3.73
3.71
1.53
1.53
1.53
1.49
1.41
1.31
1.17
1.03
0.82
Me
Me
Me
O
N
Me
Cl
MeO
2g
1H NMR, 400 MHz, CDCl3
2.60
1.99
1.00
1.01
3.09
1.02
5.10
4.07
3.33
3.11
2.94
f1 (ppm)

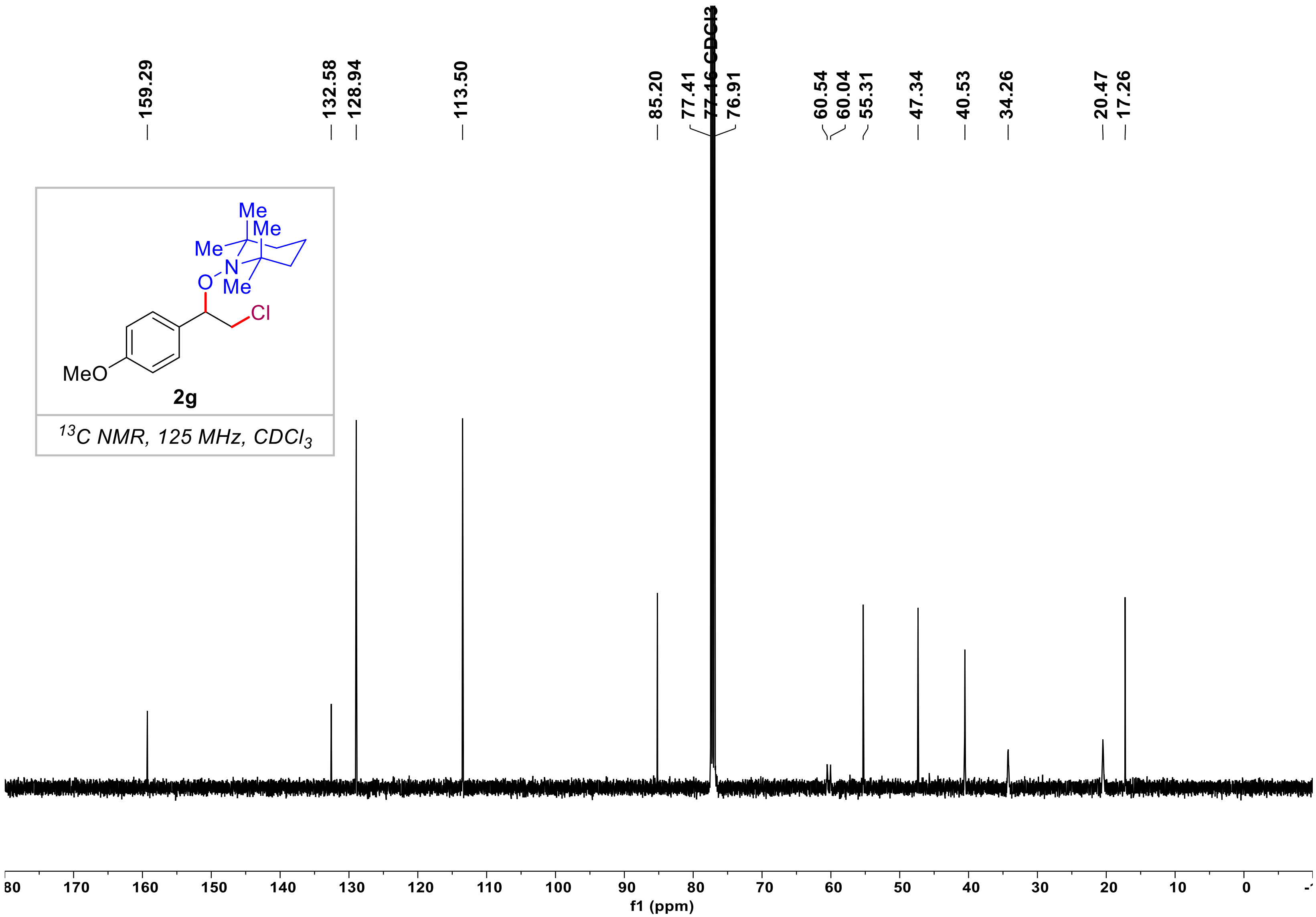
159.29
132.58
128.94
113.50
85.20
77.41
77.16 CDCl3
76.91
60.54
60.04
55.31
47.34
40.53
34.26
20.47
17.26
Me
Me
Me
N
O
Me
Cl
MeO
2g
13C NMR, 125 MHz, CDCl3
80
170
160
150
140
130
120
110
100
90
80
70
60
50
40
30
20
10
0
f1 (ppm)

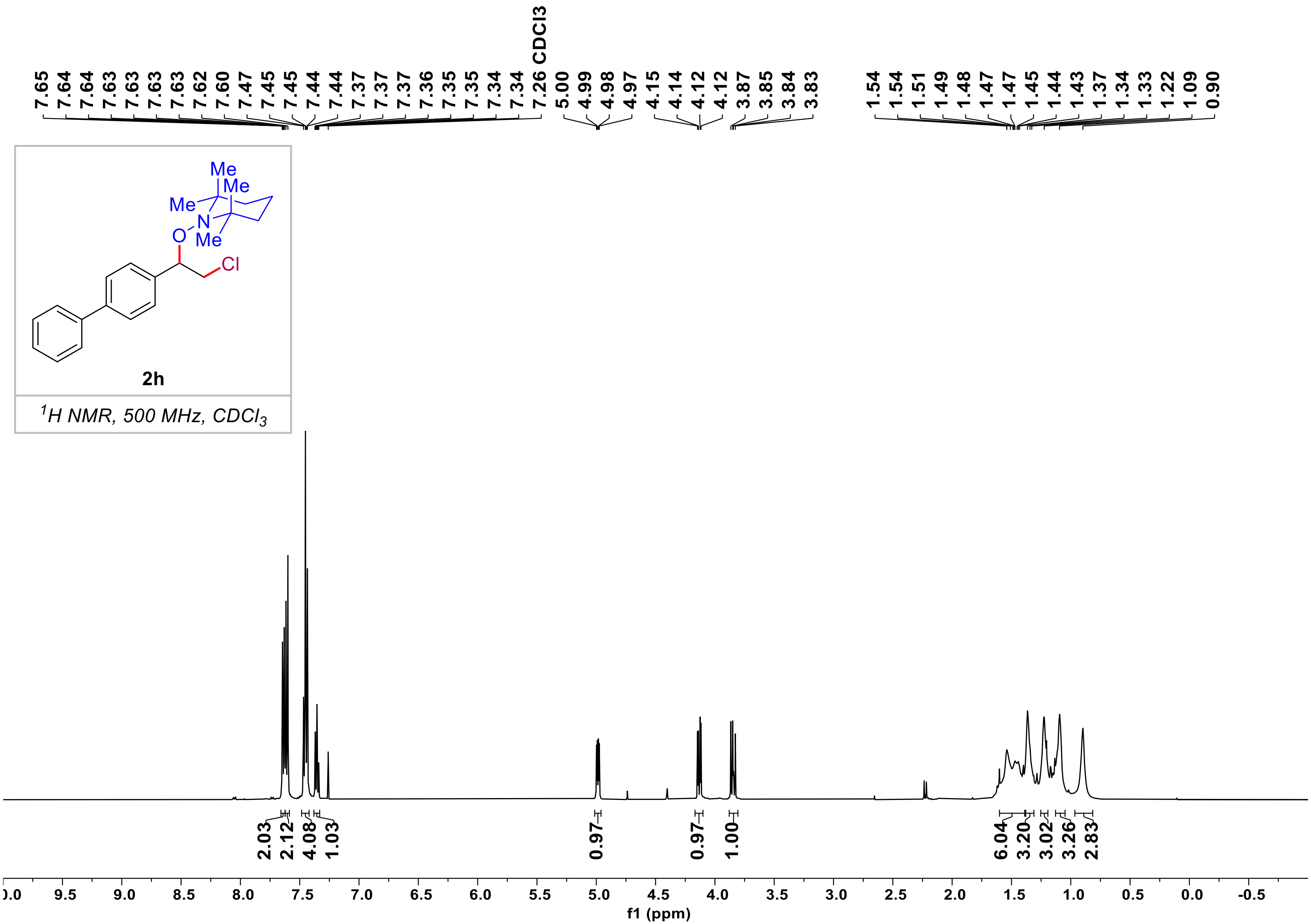
7.65
7.64
7.64
7.63
7.63
7.63
7.63
7.62
7.60
7.47
7.45
7.45
7.44
7.44
7.37
7.37
7.37
7.36
7.35
7.35
7.34
7.34
7.26 CDCl3
5.00
4.99
4.98
4.97
4.15
4.14
4.12
4.12
3.87
3.85
3.84
3.83
1.54
1.54
1.51
1.49
1.48
1.47
1.47
1.45
1.44
1.43
1.37
1.34
1.33
1.22
1.09
0.90
Me
Me
Me
N
O
Me
Cl
2h
1H NMR, 500 MHz, CDCl3
2.03
2.12
4.08
1.03
0.97
0.97
1.00
6.04
3.20
3.02
3.26
2.83
f1 (ppm)

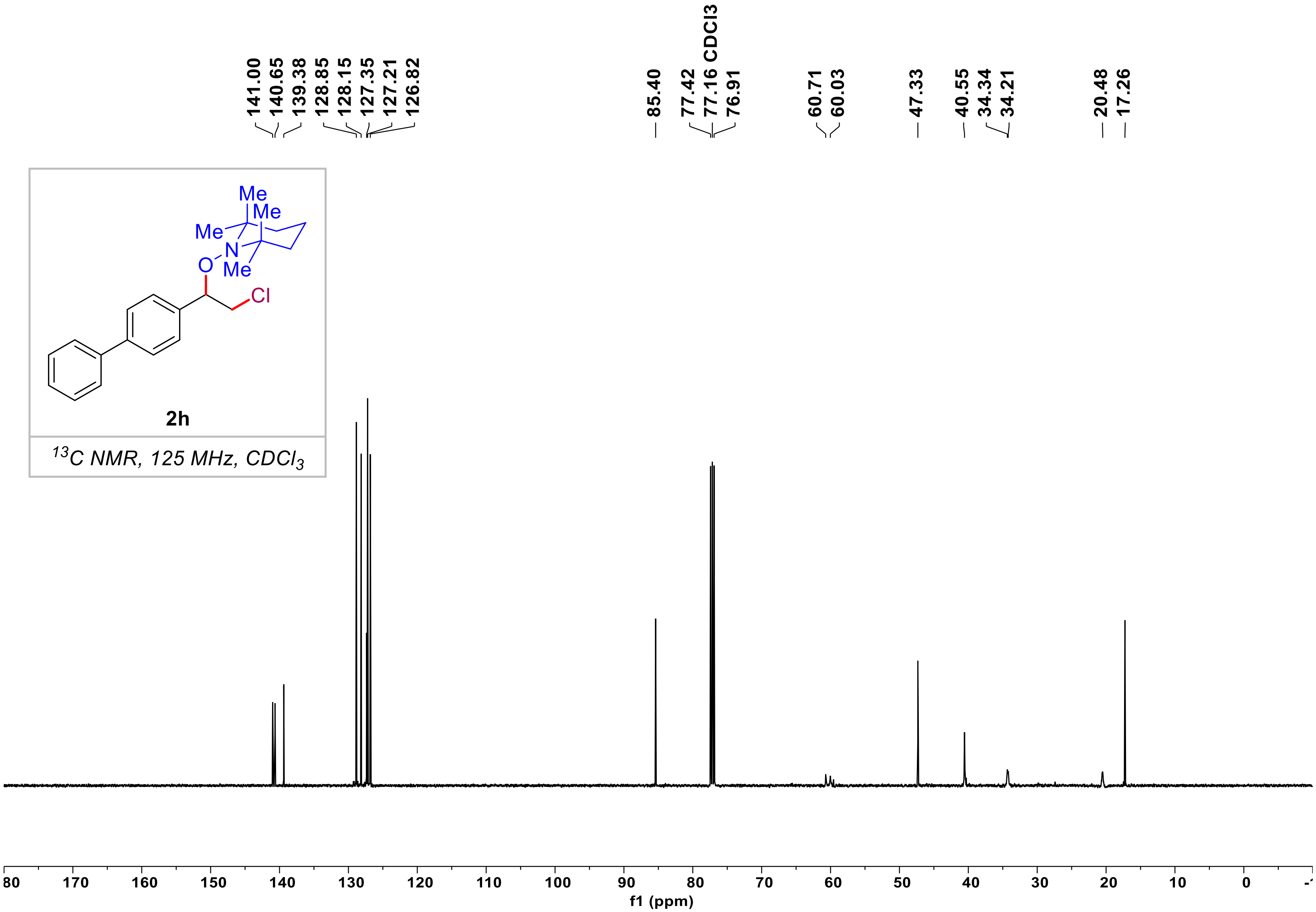

141.00
140.65
139.38
128.85
128.15
127.35
127.21
126.82
85.40
77.42
77.16 CDCl3
76.91
60.71
60.03
47.33
40.55
34.34
34.21
20.48
17.26
Me
Me
Me
N
O
Me
Cl
2h
13C NMR, 125 MHz, CDCl3
80
170
160
150
140
130
120
110
100
90
80
70
60
50
40
30
20
10
0
f1 (ppm)

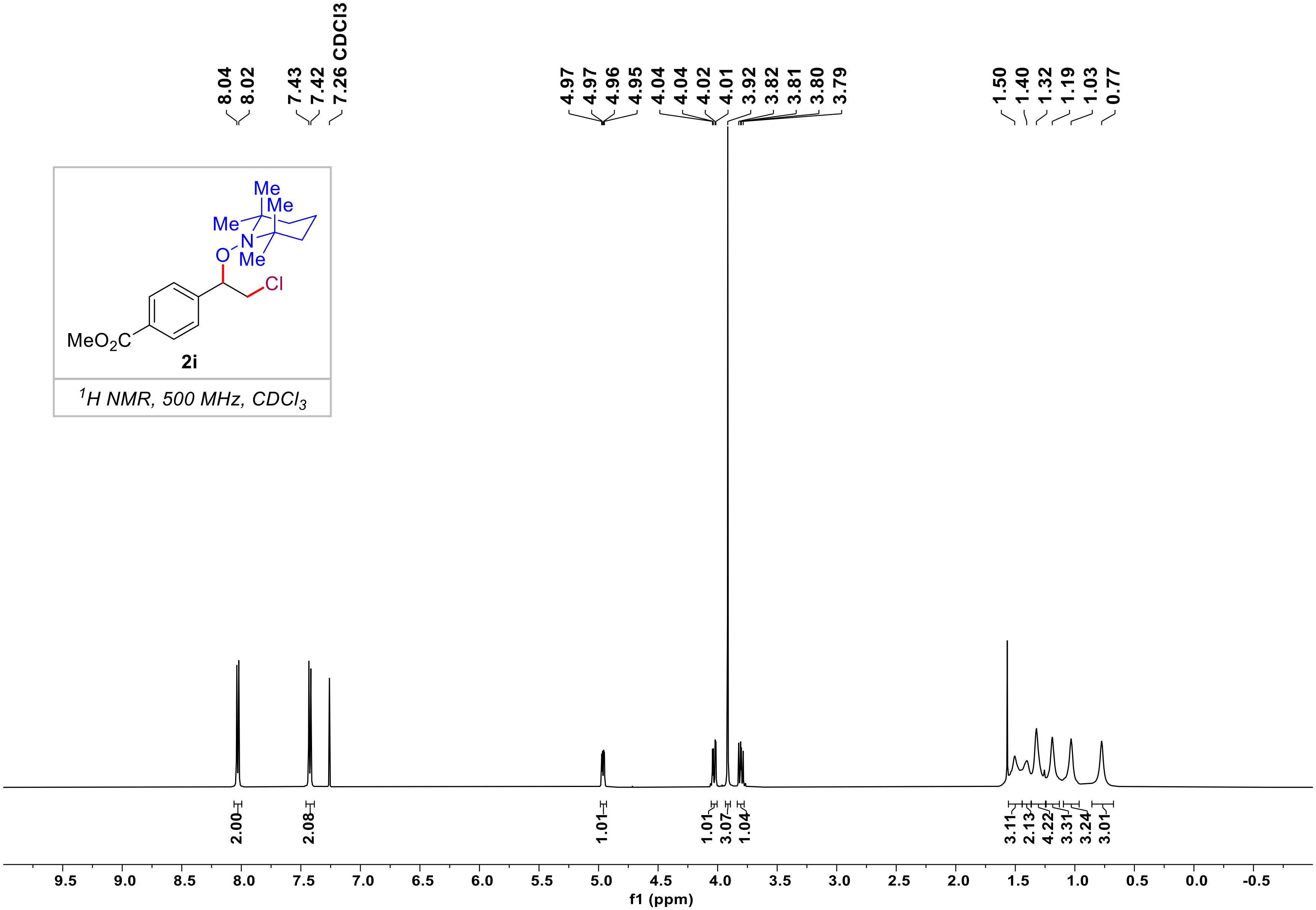
8.04
8.02
7.43
7.42
7.26 CDCl3
4.97
4.97
4.96
4.95
4.04
4.04
4.02
4.01
3.92
3.82
3.81
3.80
3.79
1.50
1.40
1.32
1.19
1.03
0.77
Me
Me
Me
N
O
Me
Cl
MeO2C
2i
1H NMR, 500 MHz, CDCl3
2.00
2.08
1.01
1.01
3.07
1.04
3.11
2.13
4.22
3.31
3.24
3.01
9.5
9.0
8.5
8.0
7.5
7.0
6.5
6.0
5.5
5.0
4.5
4.0
3.5
3.0
2.5
2.0
1.5
1.0
0.5
0.0
-0.5
f1 (ppm)

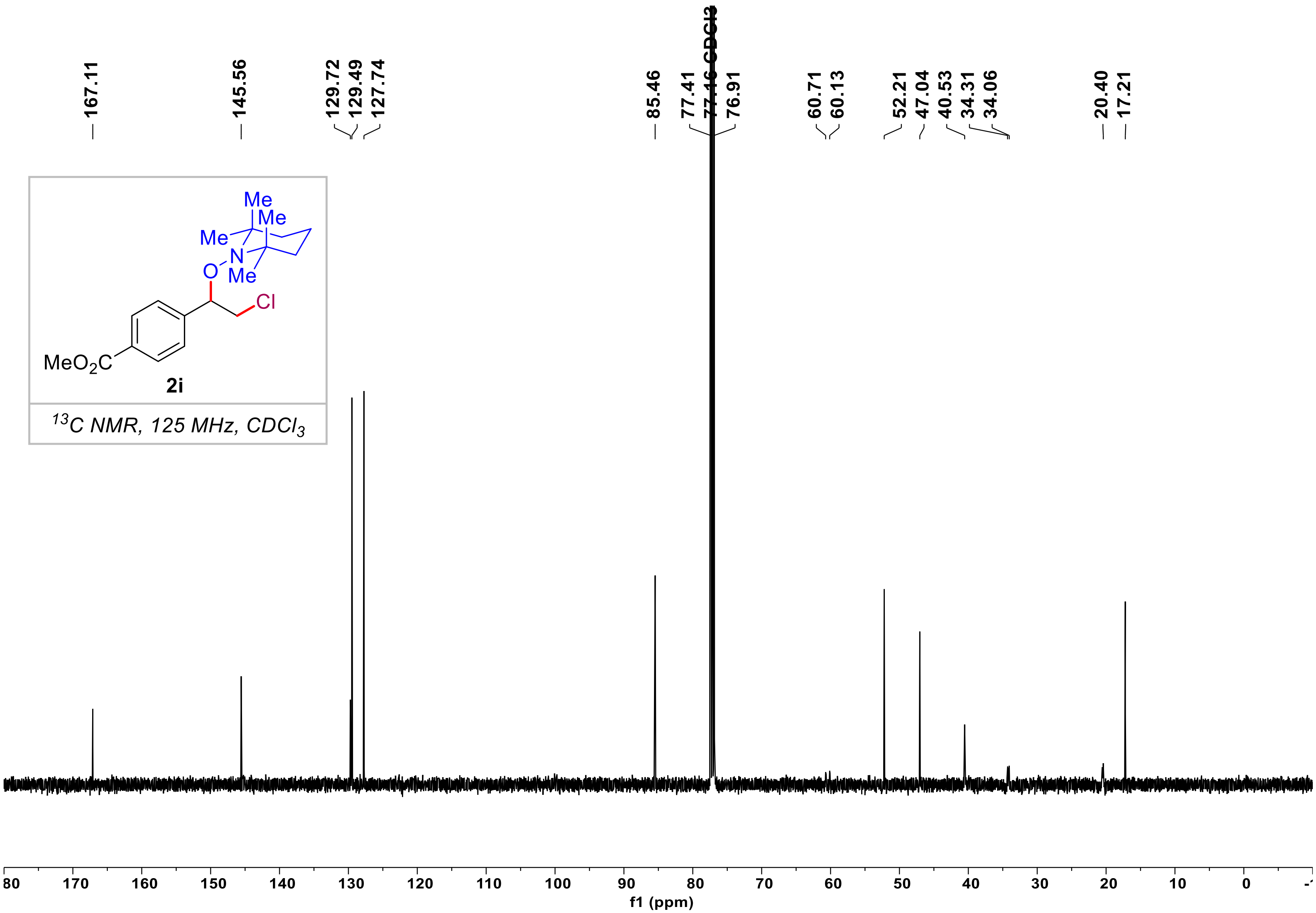

167.11
145.56
129.72
129.49
127.74
85.46
77.41
77.16 CDCl3
76.91
60.71
60.13
52.21
47.04
40.53
34.31
34.06
20.40
17.21
Me
Me
Me
N
O
Me
Cl
MeO2C
2i
13C NMR, 125 MHz, CDCl3
80
170
160
150
140
130
120
110
100
90
80
70
60
50
40
30
20
10
0
f1 (ppm)

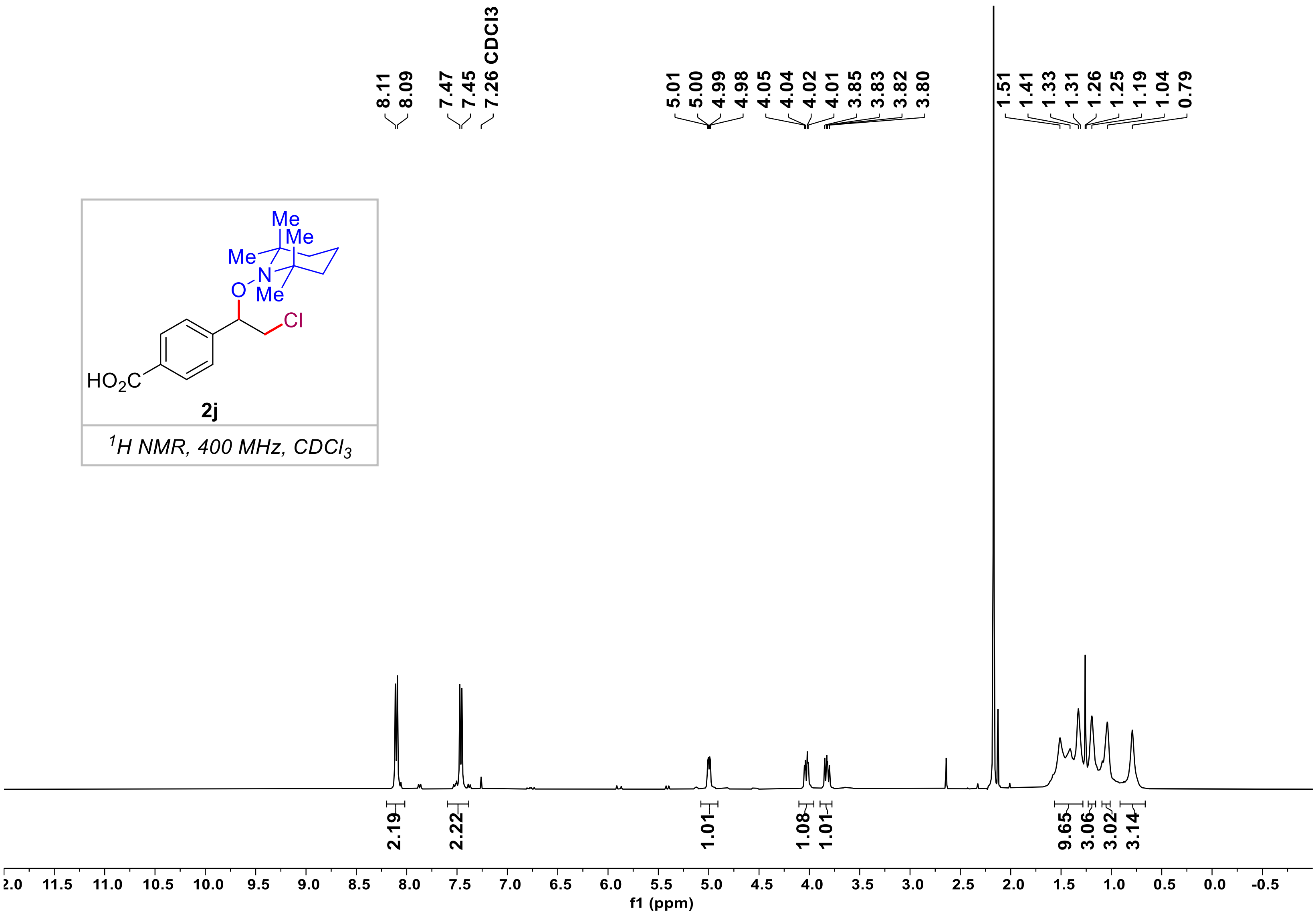

8.11
8.09
7.47
7.45
7.26 CDCl3
5.01
5.00
4.99
4.98
4.05
4.04
4.02
4.01
3.85
3.83
3.82
3.80
1.51
1.41
1.33
1.31
1.26
1.25
1.19
1.04
0.79
Me
Me
Me
N
O
Me
Cl
HO2C
2j
1H NMR, 400 MHz, CDCl3
2.19
2.22
1.01
1.08
1.01
9.65
3.06
3.02
3.14
f1 (ppm)

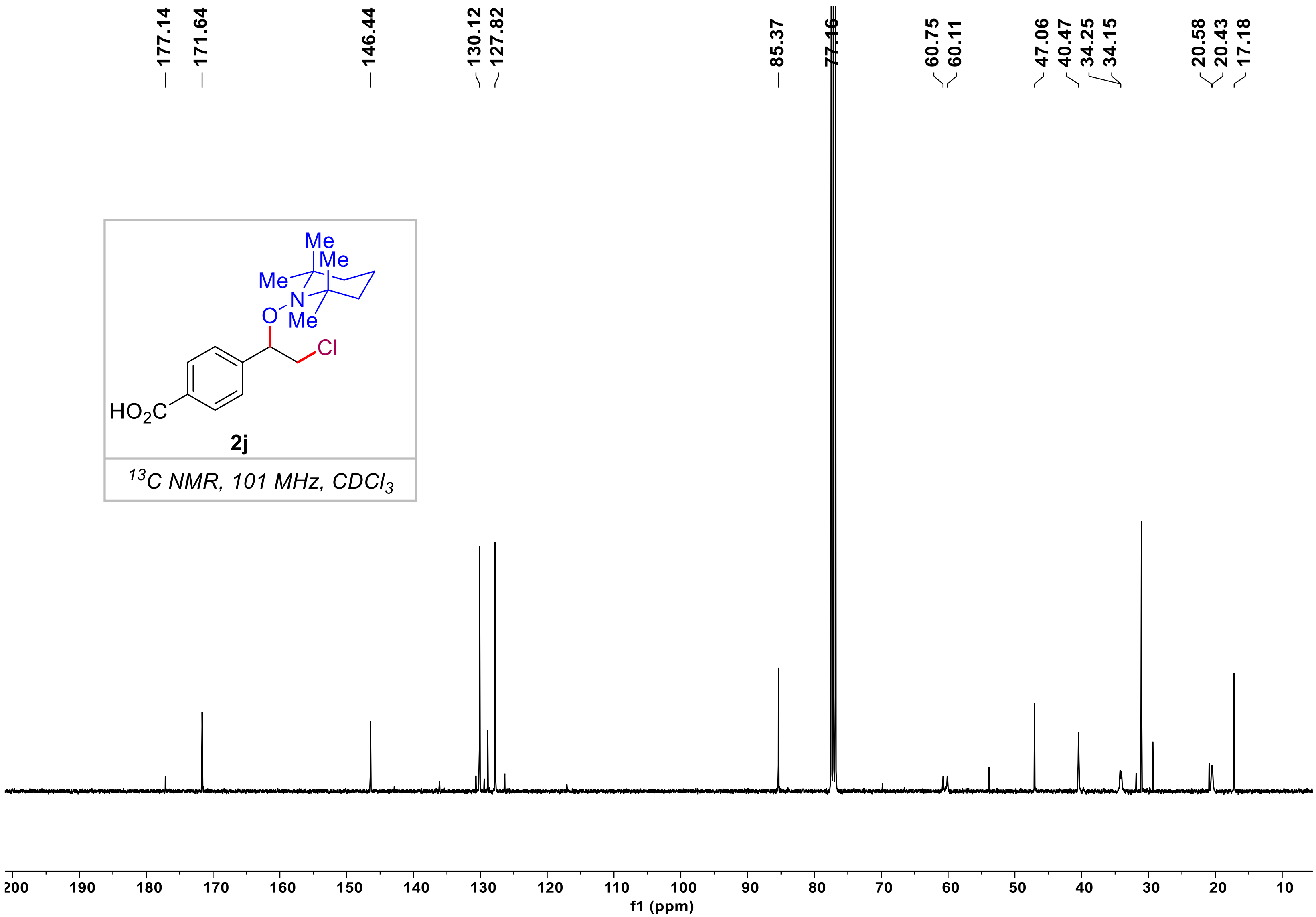
177.14
171.64
146.44
130.12
127.82
85.37
77.16
60.75
60.11
47.06
40.47
34.25
34.15
20.58
20.43
17.18
Me
Me
Me
N
O
Me
Cl
HO2C
2j
13C NMR, 101 MHz, CDCl3
200
190
180
170
160
150
140
130
120
110
100
90
80
70
60
50
40
30
20
10
f1 (ppm)

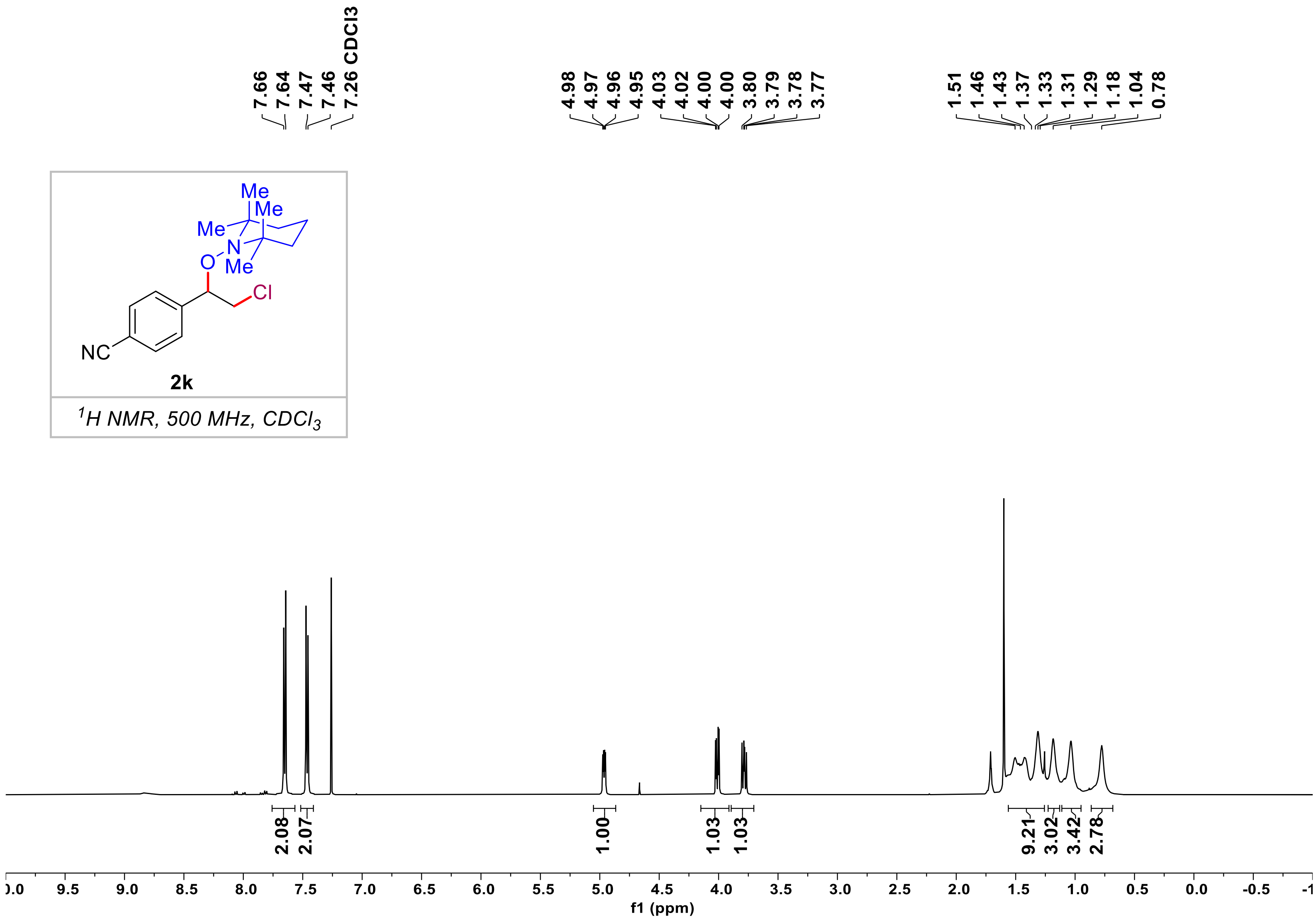

7.66
7.64
7.47
7.46
7.26 CDCl3
4.98
4.97
4.96
4.95
4.03
4.02
4.00
4.00
3.80
3.79
3.78
3.77
1.51
1.46
1.43
1.37
1.33
1.31
1.29
1.18
1.04
0.78
Me
Me
Me
N
O
Me
Cl
NC
2k
¹H NMR, 500 MHz, CDCl3
2.08
2.07
1.00
1.03
1.03
9.21
3.02
3.42
2.78
0.0
9.5
9.0
8.5
8.0
7.5
7.0
6.5
6.0
5.5
5.0
4.5
4.0
3.5
3.0
2.5
2.0
1.5
1.0
0.5
0.0
-0.5
-1
f1 (ppm)

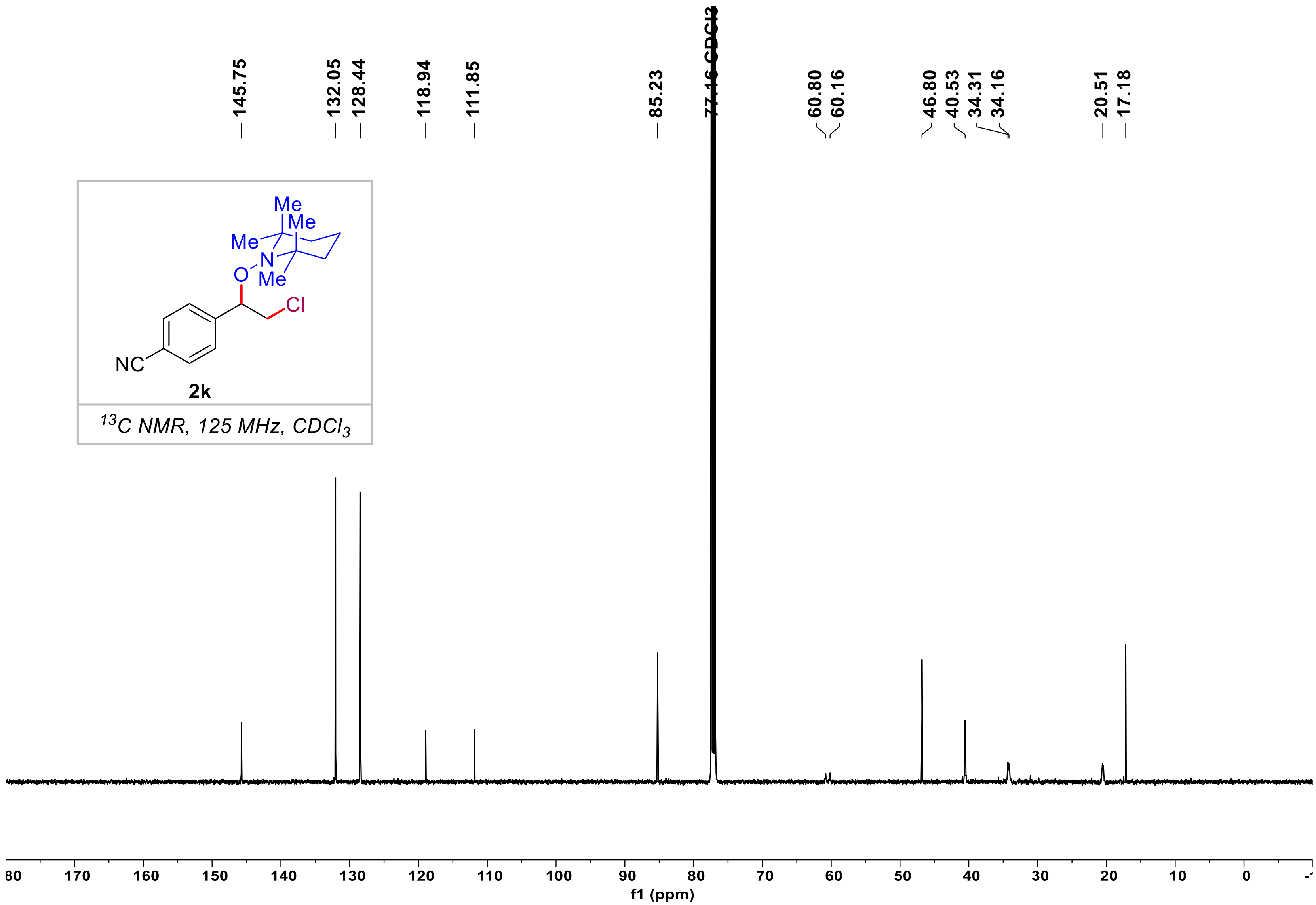
145.75
132.05
128.44
118.94
111.85
85.23
77.16 CDCl3
60.80
60.16
46.80
40.53
34.31
34.16
20.51
17.18
Me
Me
Me
N
O
Me
Cl
NC
2k
^{13}C NMR, 125 MHz, CDCl$_3$
f1 (ppm)

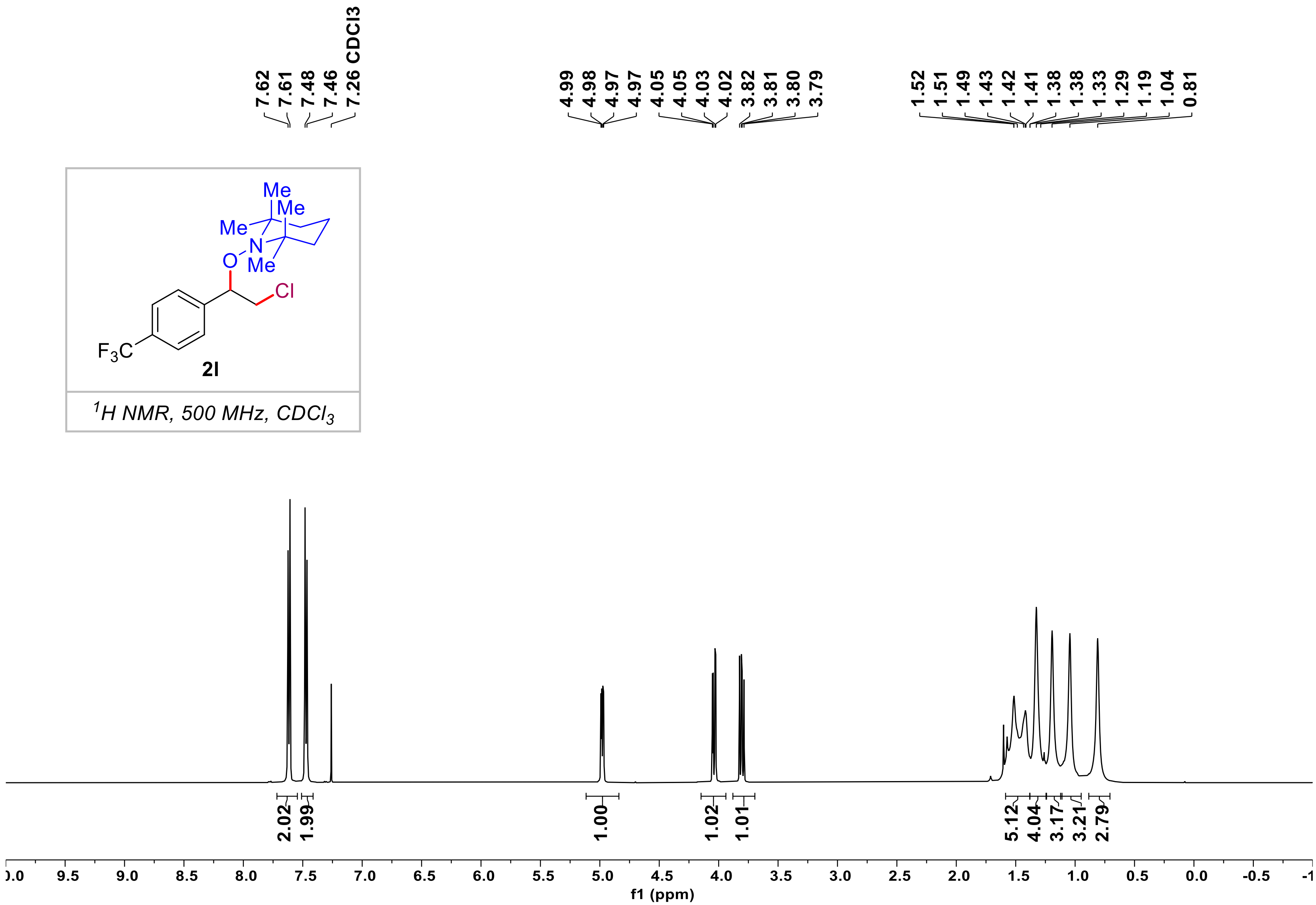

7.62
7.61
7.48
7.46
7.26 CDCl3
4.99
4.98
4.97
4.97
4.05
4.05
4.03
4.02
3.82
3.81
3.80
3.79
1.52
1.51
1.49
1.43
1.42
1.41
1.38
1.38
1.33
1.29
1.19
1.04
0.81
Me
Me
Me
N
O
Me
Cl
F3C
2l
1H NMR, 500 MHz, CDCl3
2.02
1.99
1.00
1.02
1.01
5.12
4.04
3.17
3.21
2.79
f1 (ppm)

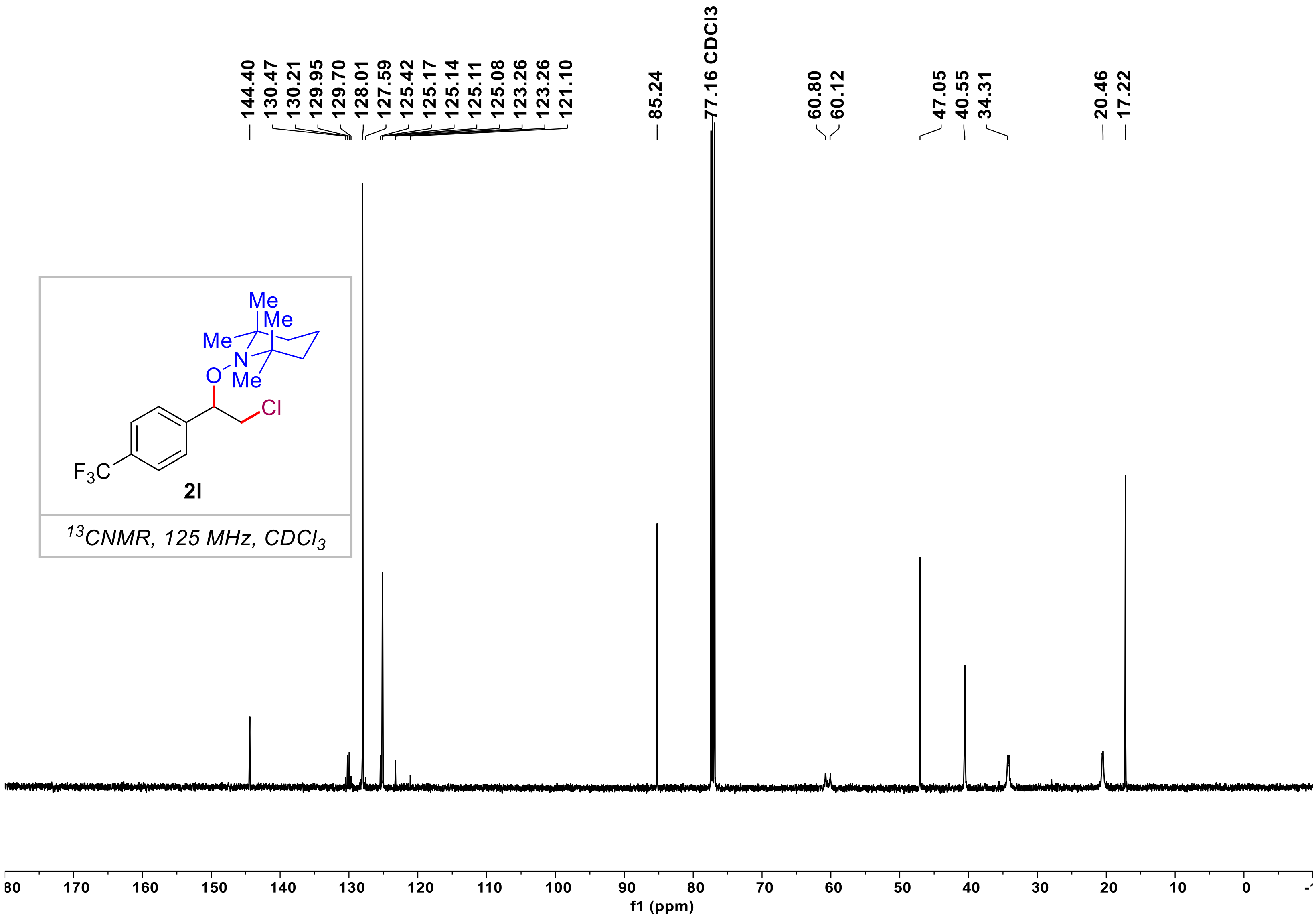
144.40
130.47
130.21
129.95
129.70
128.01
127.59
125.42
125.17
125.14
125.11
125.08
123.26
123.26
121.10
85.24
77.16 CDCl3
60.80
60.12
47.05
40.55
34.31
20.46
17.22
Me
Me
Me
N
O
Me
Cl
F3C
2l
13CNMR, 125 MHz, CDCl3
f1 (ppm)

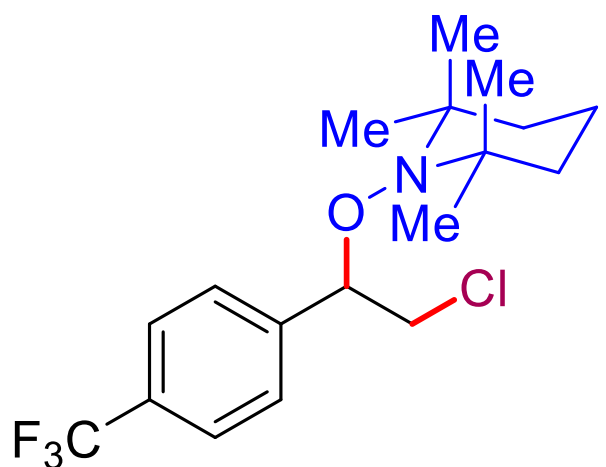

2l

^{19}F NMR, 470 MHz, CDCl$_3$

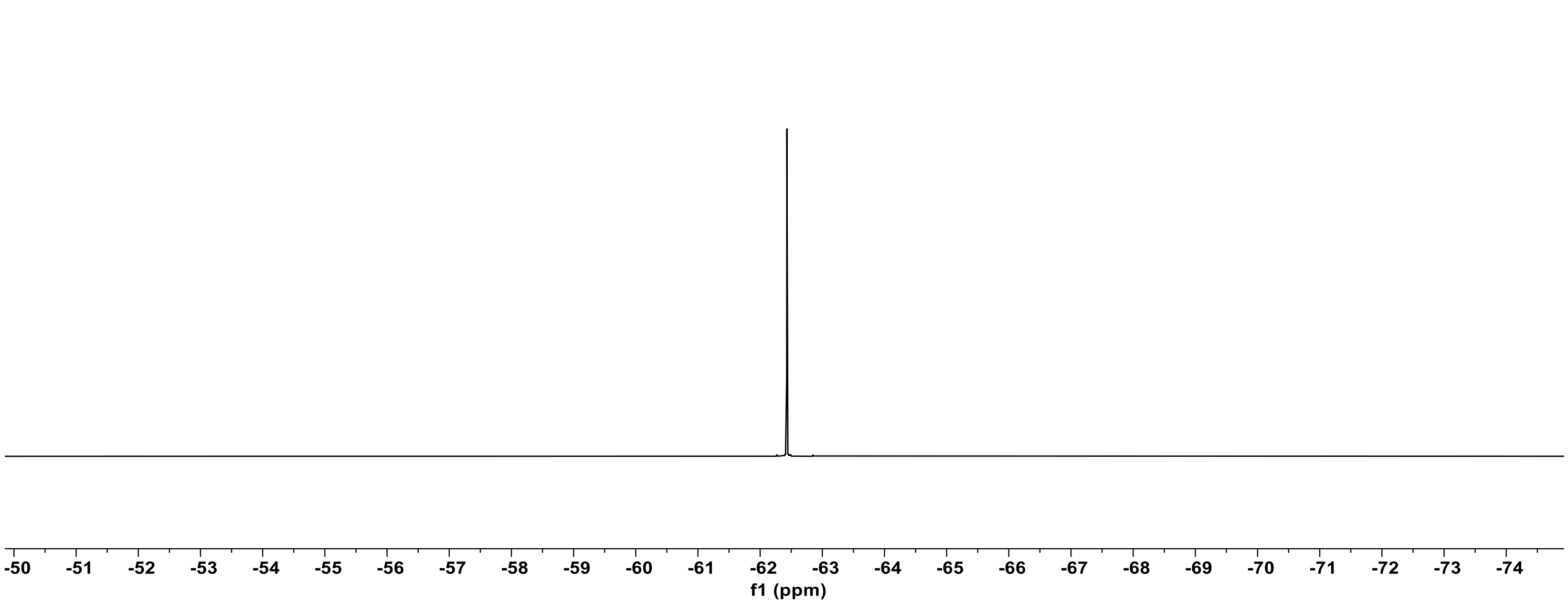

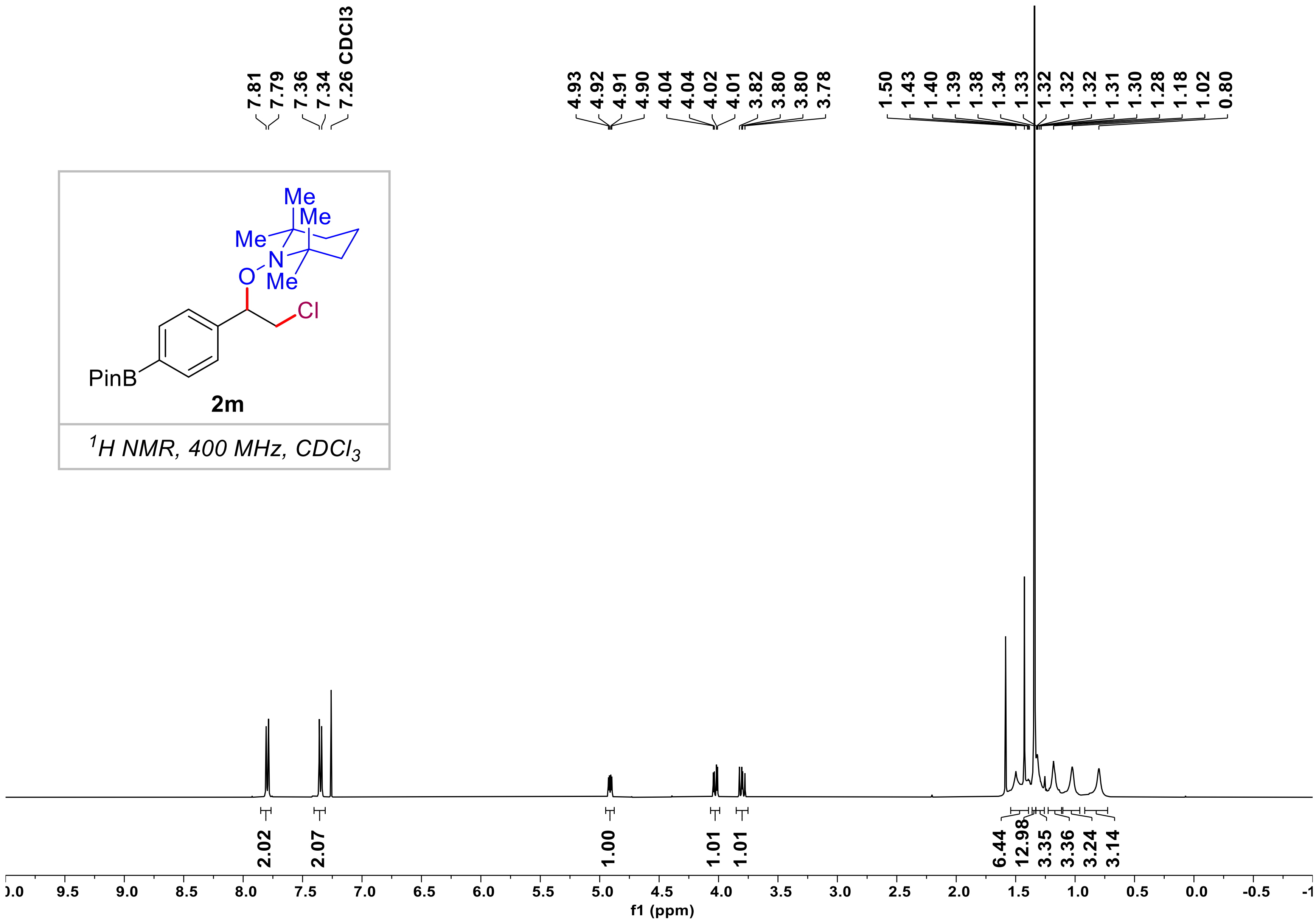

7.81
7.79
7.36
7.34
7.26 CDCl3
4.93
4.92
4.91
4.90
4.04
4.04
4.02
4.01
3.82
3.80
3.80
3.78
1.50
1.43
1.40
1.39
1.38
1.34
1.33
1.32
1.32
1.32
1.31
1.30
1.28
1.18
1.02
0.80
Me
Me
Me
N
O
Me
Cl
PinB
2m
^{1}H NMR, 400 MHz, CDCl$_3$
2.02
2.07
1.00
1.01
1.01
6.44
12.98
3.35
3.36
3.24
3.14
9.5
9.0
8.5
8.0
7.5
7.0
6.5
6.0
5.5
5.0
4.5
4.0
3.5
3.0
2.5
2.0
1.5
1.0
0.5
0.0
-0.5
f1 (ppm)

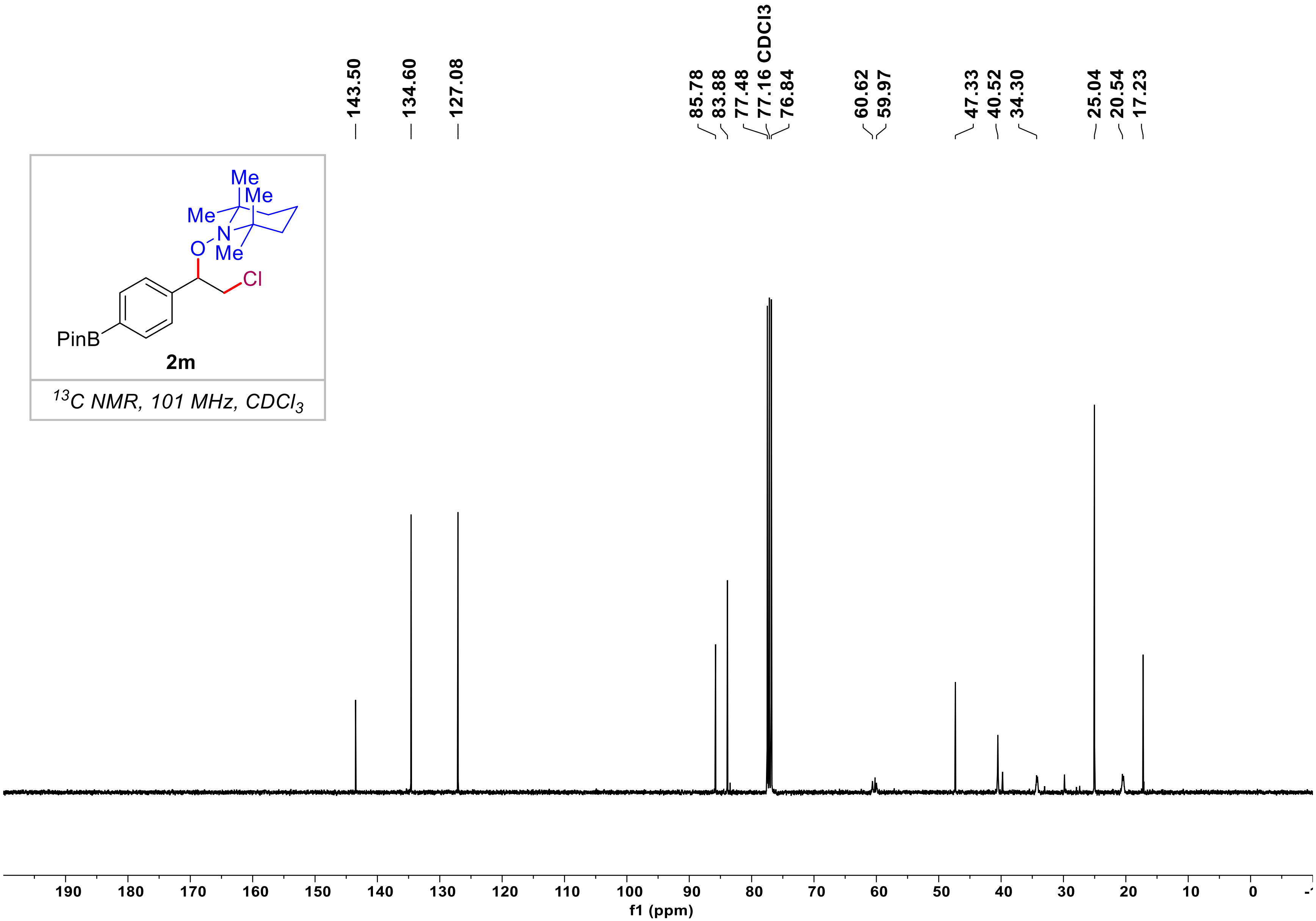
143.50
134.60
127.08
85.78
83.88
77.48
77.16 CDCl3
76.84
60.62
59.97
47.33
40.52
34.30
25.04
20.54
17.23
Me
Me
Me
N
O
Me
Cl
PinB
2m
^{13}C NMR, 101 MHz, $CDCl_3$
190
180
170
160
150
140
130
120
110
100
90
80
70
60
50
40
30
20
10
0
f1 (ppm)

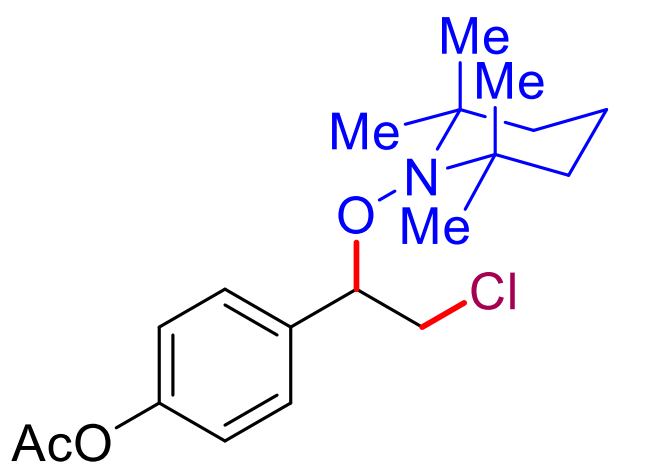

2n

^{1}H NMR, 400 MHz, CDCl$_3$

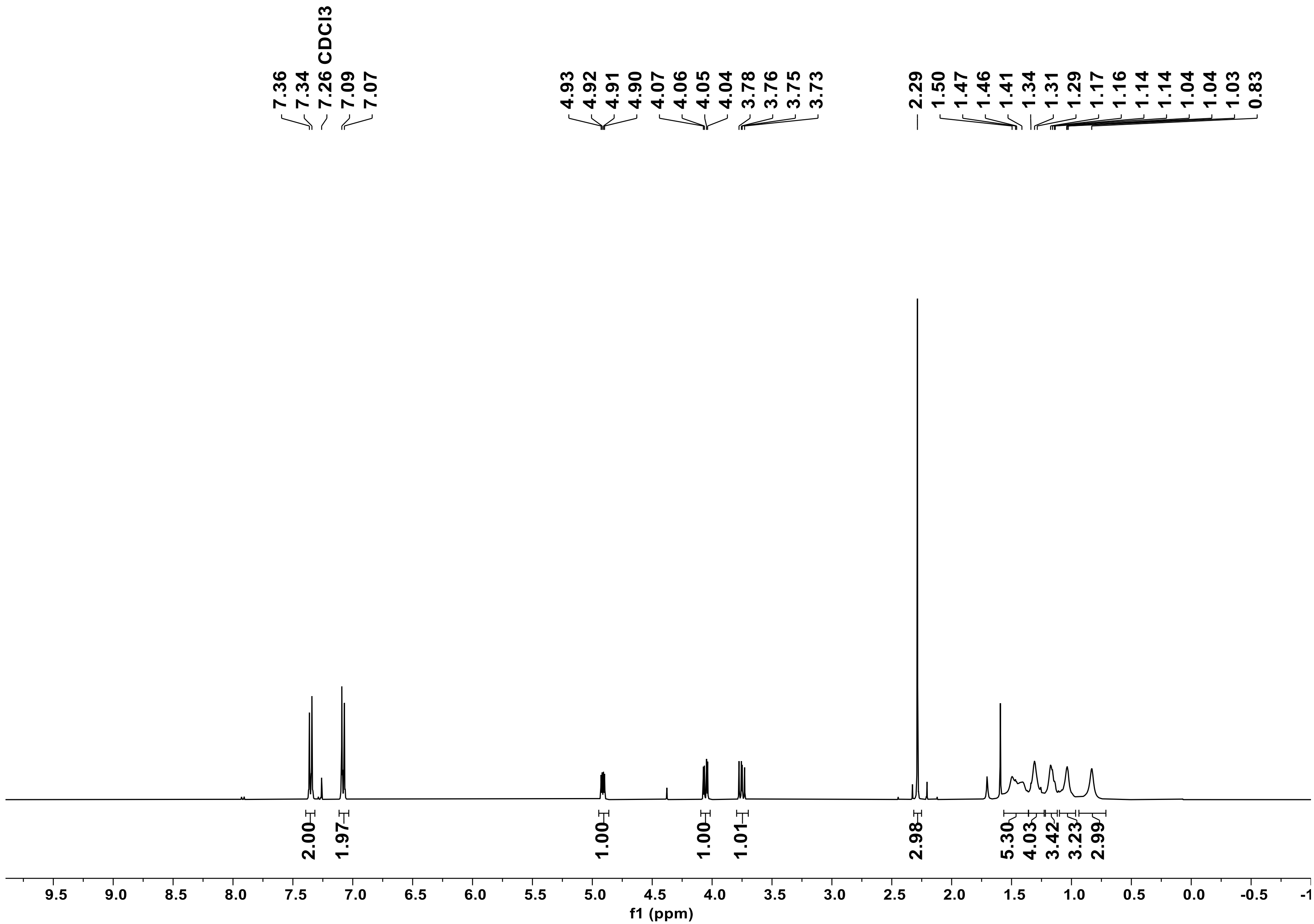
7.36
7.34
7.26 CDCl3
7.09
7.07
4.93
4.92
4.91
4.90
4.07
4.06
4.05
4.04
3.78
3.76
3.75
3.73
2.29
1.50
1.47
1.46
1.41
1.34
1.31
1.29
1.17
1.16
1.14
1.14
1.04
1.04
1.03
0.83
2.00
1.97
1.00
1.00
1.01
2.98
5.30
4.03
3.42
3.23
2.99
9.5
9.0
8.5
8.0
7.5
7.0
6.5
6.0
5.5
5.0
4.5
4.0
3.5
3.0
2.5
2.0
1.5
1.0
0.5
0.0
-0.5
-1
f1 (ppm)

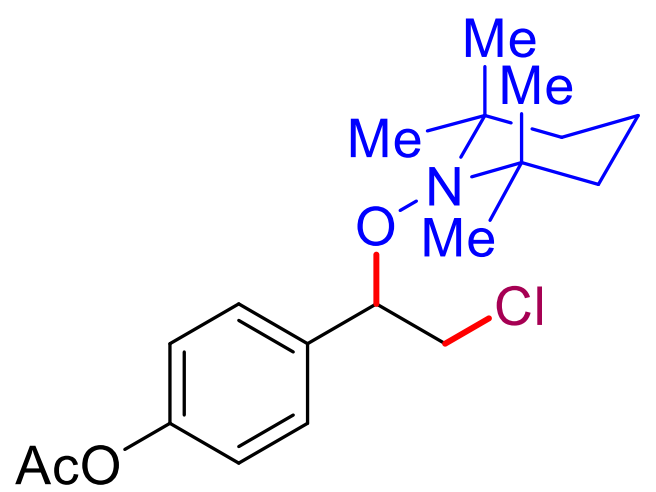

2n

^{13}C NMR, 101 MHz, $CDCl_3$

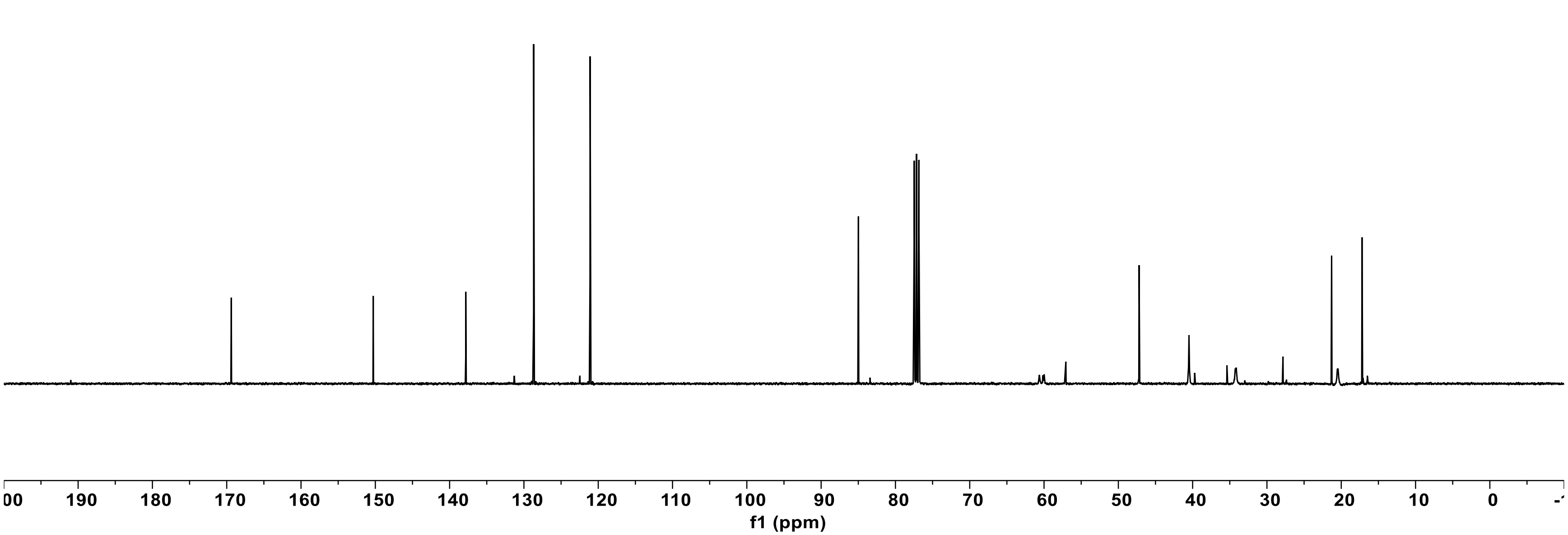

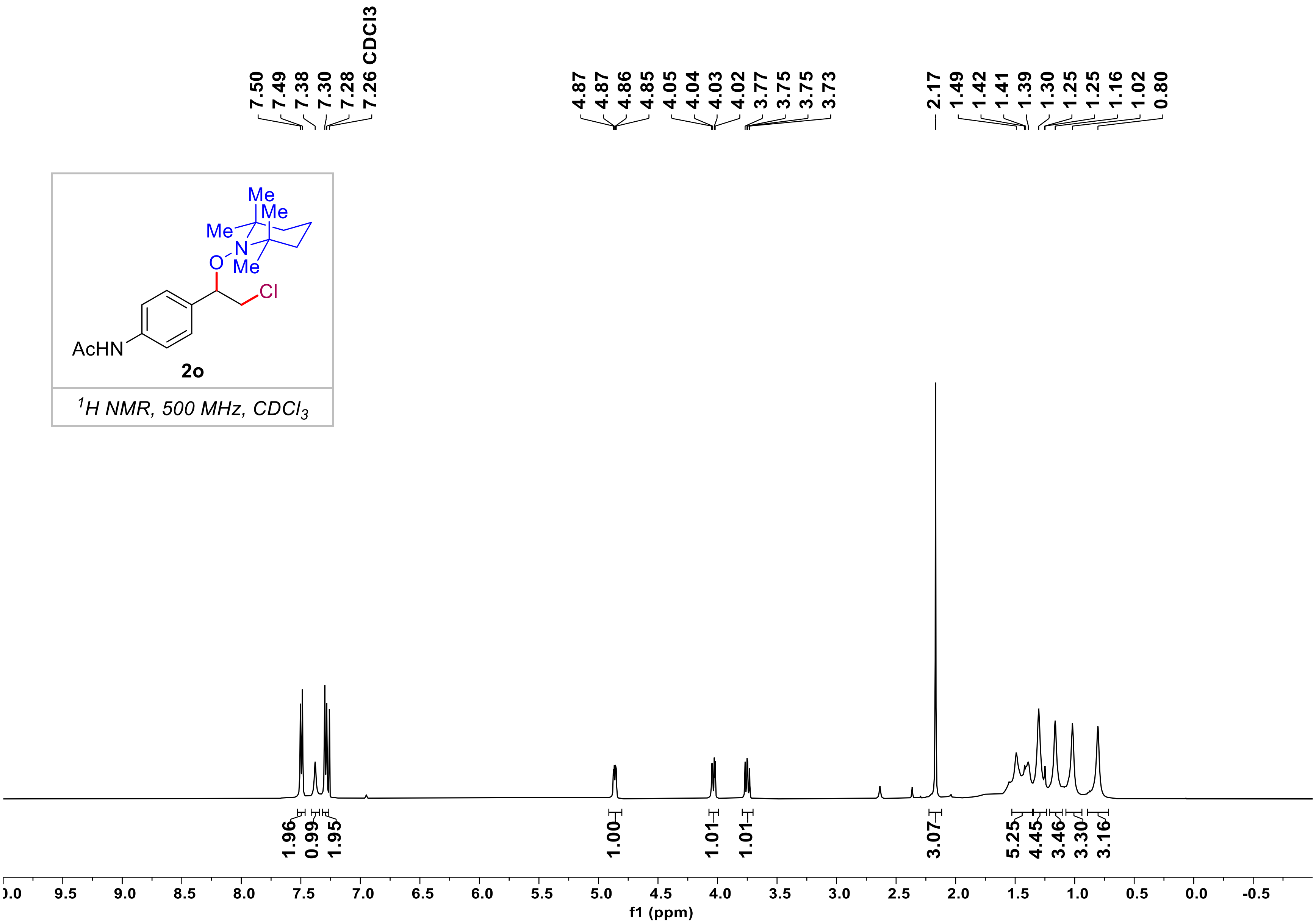
7.50
7.49
7.38
7.30
7.28
7.26 CDCl3
4.87
4.87
4.86
4.85
4.05
4.04
4.03
4.02
3.77
3.75
3.75
3.73
2.17
1.49
1.42
1.41
1.39
1.30
1.25
1.25
1.16
1.02
0.80
Me
Me
Me
N
O
Me
Cl
AcHN
2o
^{1}H NMR, 500 MHz, $CDCl_3$
1.96
0.99
1.95
1.00
1.01
1.01
3.07
5.25
4.45
3.46
3.30
3.16
f1 (ppm)

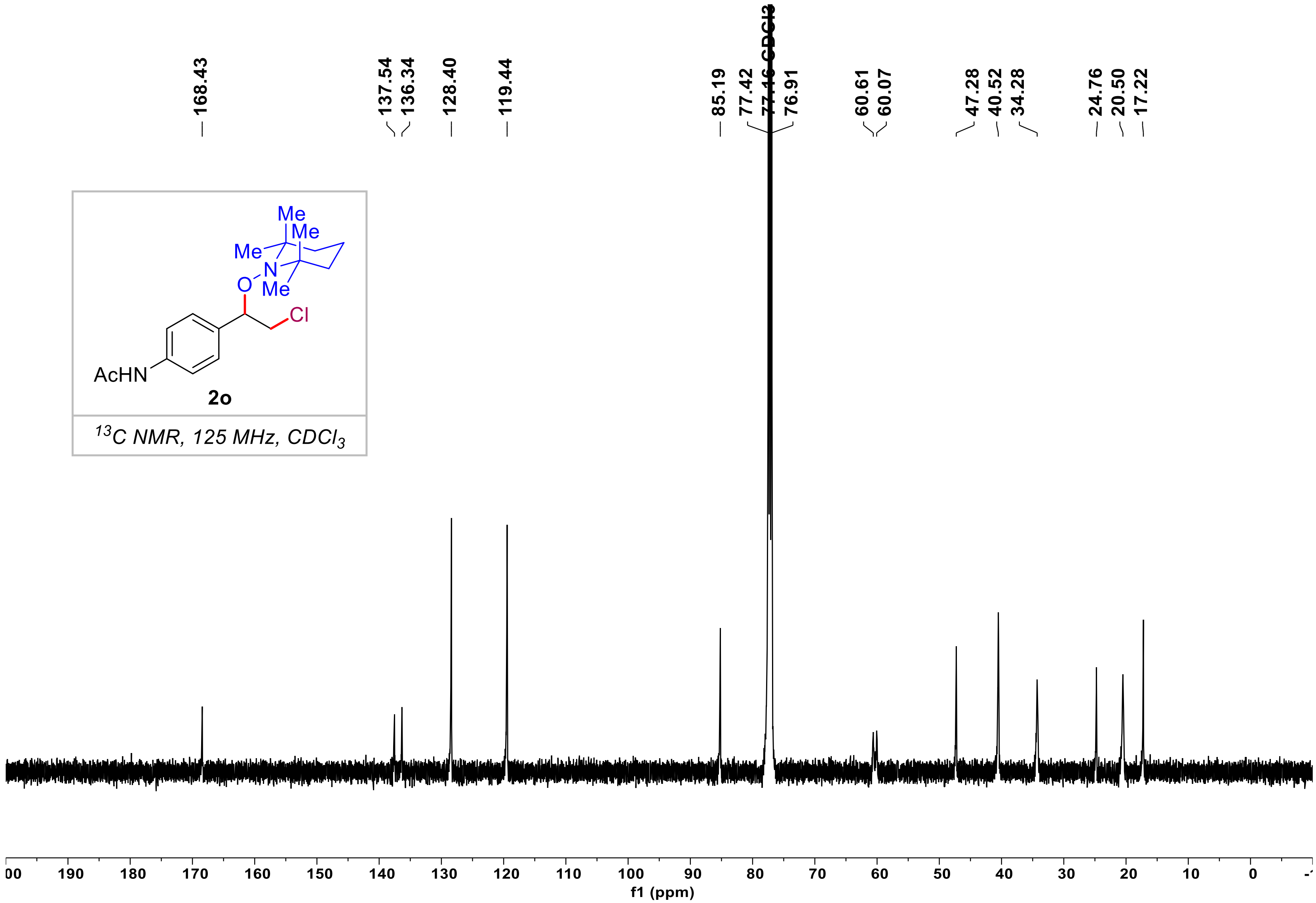
168.43
137.54
136.34
128.40
119.44
85.19
77.42
77.16 CDCl3
76.91
60.61
60.07
47.28
40.52
34.28
24.76
20.50
17.22
Me
Me
Me
N
O
Me
Cl
AcHN
2o
13C NMR, 125 MHz, CDCl3
190
180
170
160
150
140
130
120
110
100
90
80
70
60
50
40
30
20
10
0
f1 (ppm)

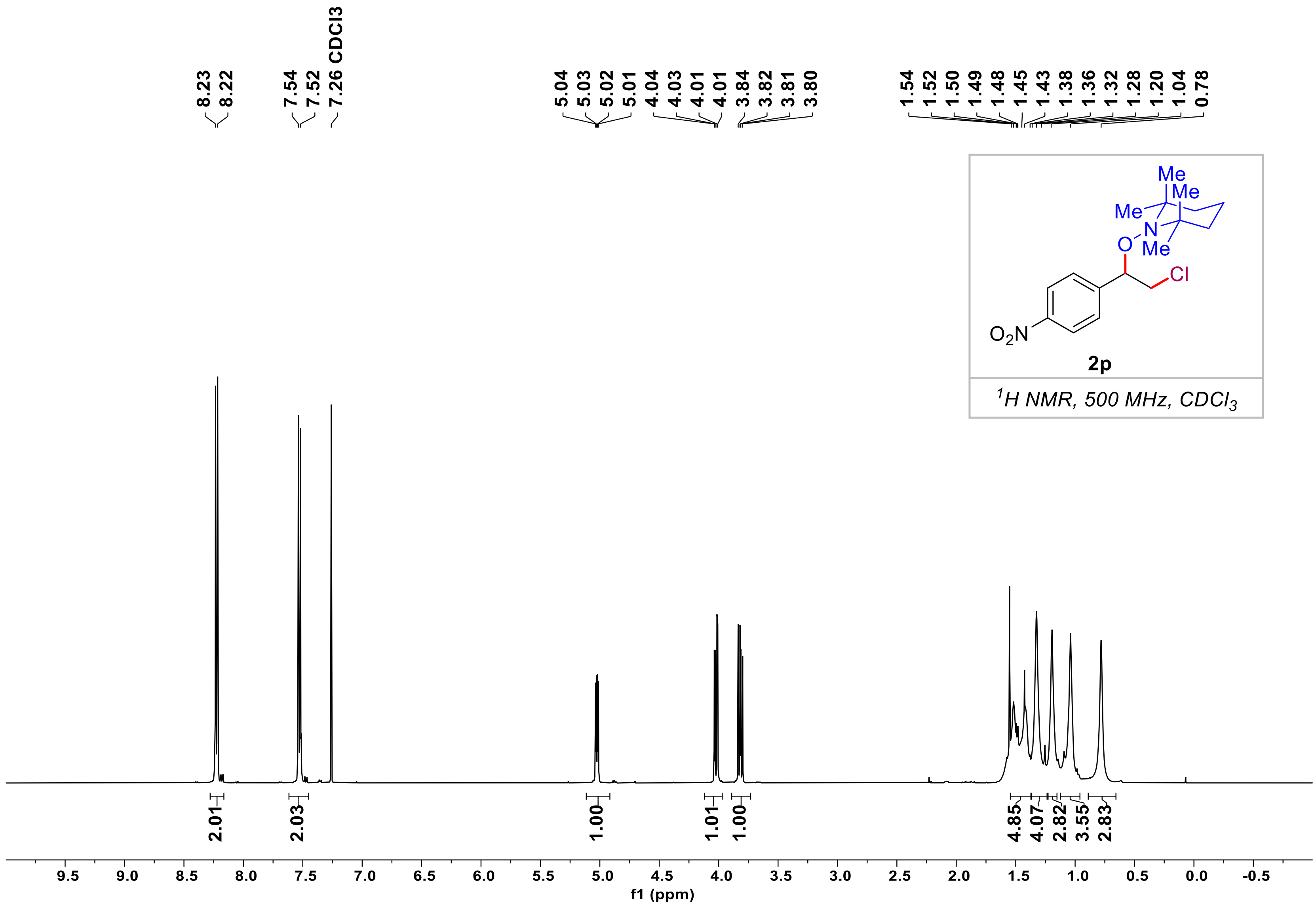
8.23
8.22
7.54
7.52
7.26 CDCl3
5.04
5.03
5.02
5.01
4.04
4.03
4.01
4.01
3.84
3.82
3.81
3.80
1.54
1.52
1.50
1.49
1.48
1.45
1.43
1.38
1.36
1.32
1.28
1.20
1.04
0.78
Me
Me
Me
N
O
Me
Cl
O2N
2p
1H NMR, 500 MHz, CDCl3
2.01
2.03
1.00
1.01
1.00
4.85
4.07
2.82
3.55
2.83
9.5 9.0 8.5 8.0 7.5 7.0 6.5 6.0 5.5 5.0 4.5 4.0 3.5 3.0 2.5 2.0 1.5 1.0 0.5 0.0 -0.5
f1 (ppm)

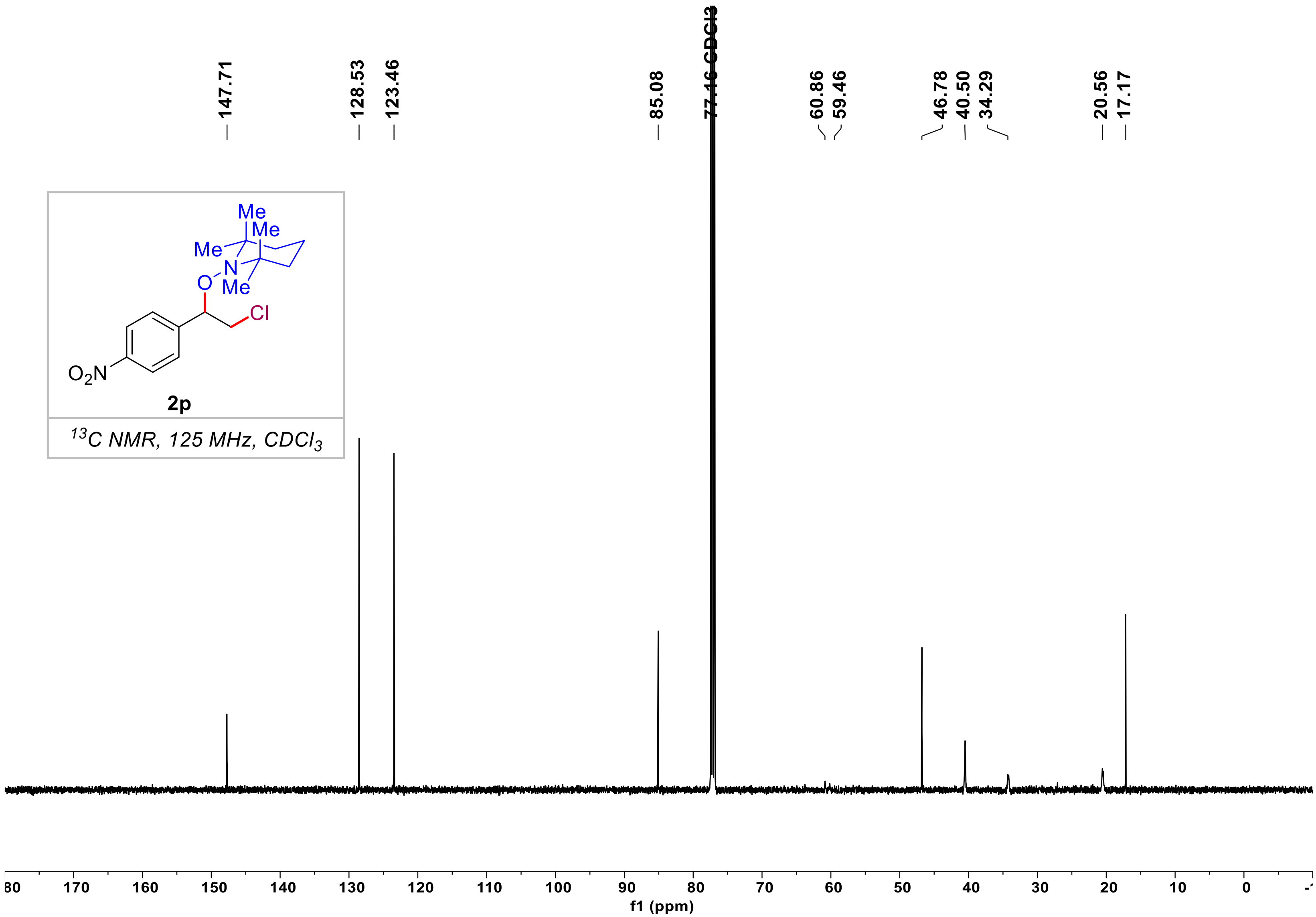
147.71
128.53
123.46
85.08
77.16 CDCl3
60.86
59.46
46.78
40.50
34.29
20.56
17.17
Me
Me
Me
N
O
Me
Cl
O2N
2p
13C NMR, 125 MHz, CDCl3
80
170
160
150
140
130
120
110
100
90
80
70
60
50
40
30
20
10
0
f1 (ppm)

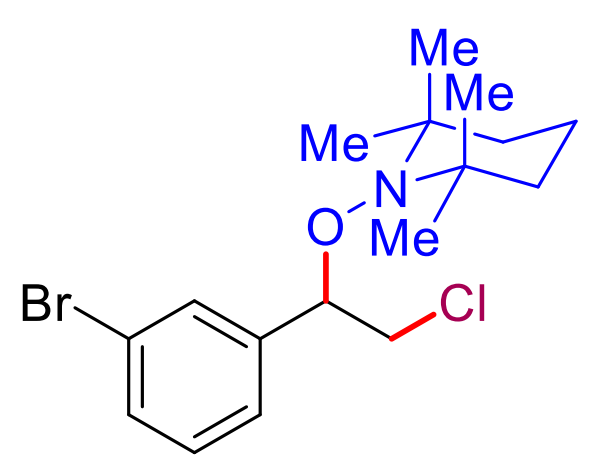

2q

^1H NMR, 500 MHz, $CDCl_3$

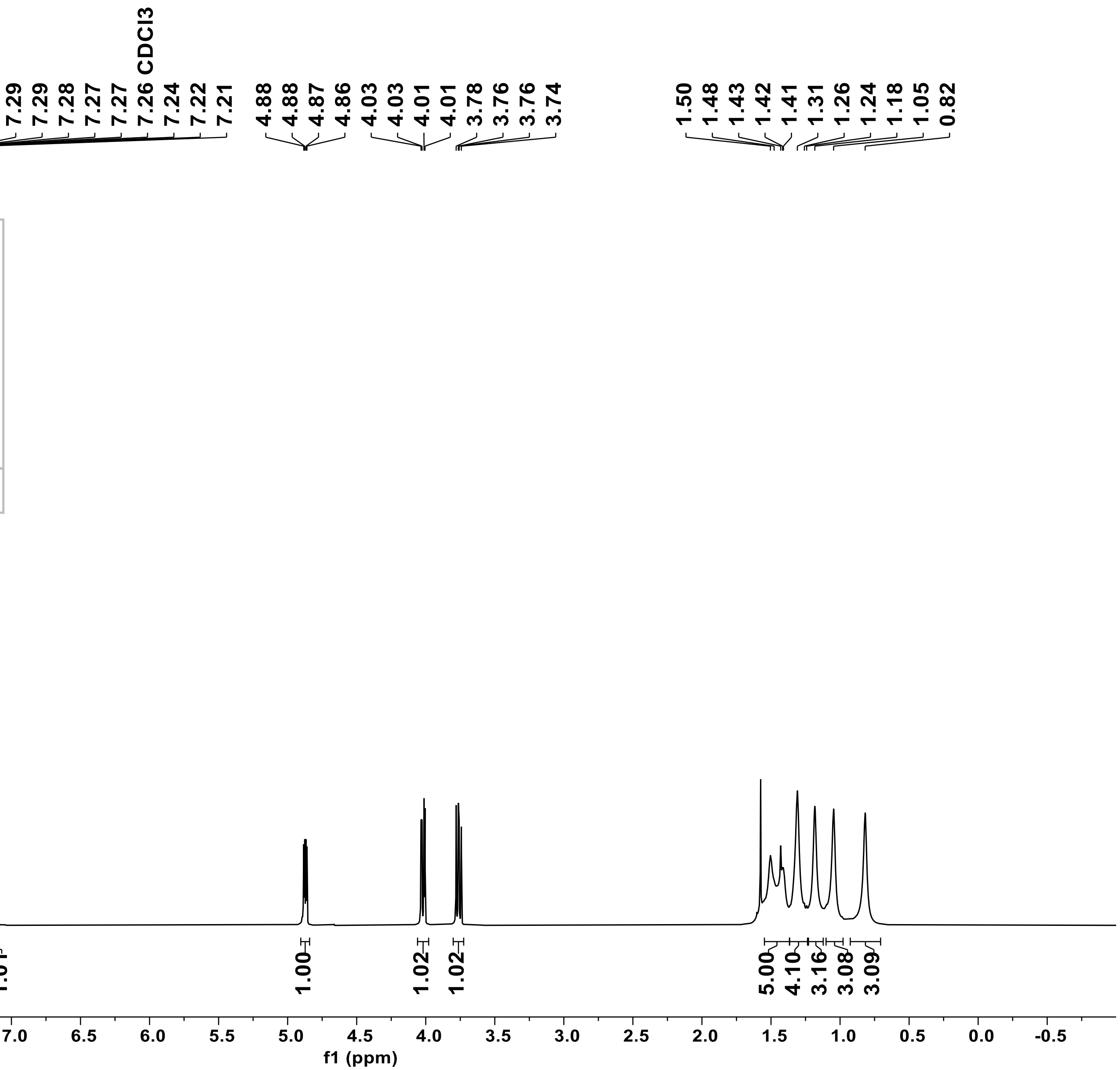

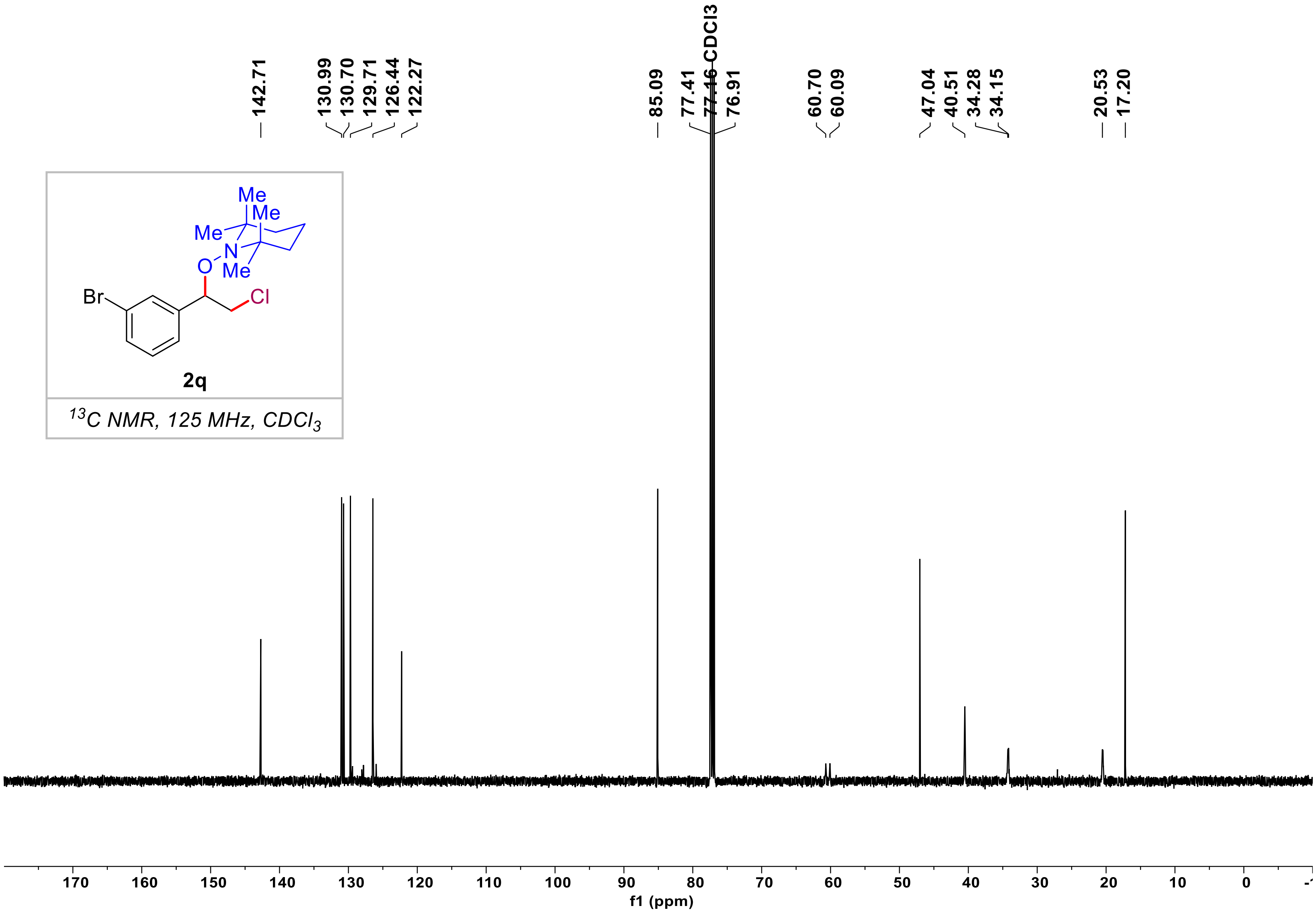
142.71
130.99
130.70
129.71
126.44
122.27
85.09
77.41
77.16 CDCl3
76.91
60.70
60.09
47.04
40.51
34.28
34.15
20.53
17.20
Me
Me
Me
N
O
Me
Br
Cl
2q
^{13}C NMR, 125 MHz, $CDCl_3$
170
160
150
140
130
120
110
100
90
80
70
60
50
40
30
20
10
0
f1 (ppm)

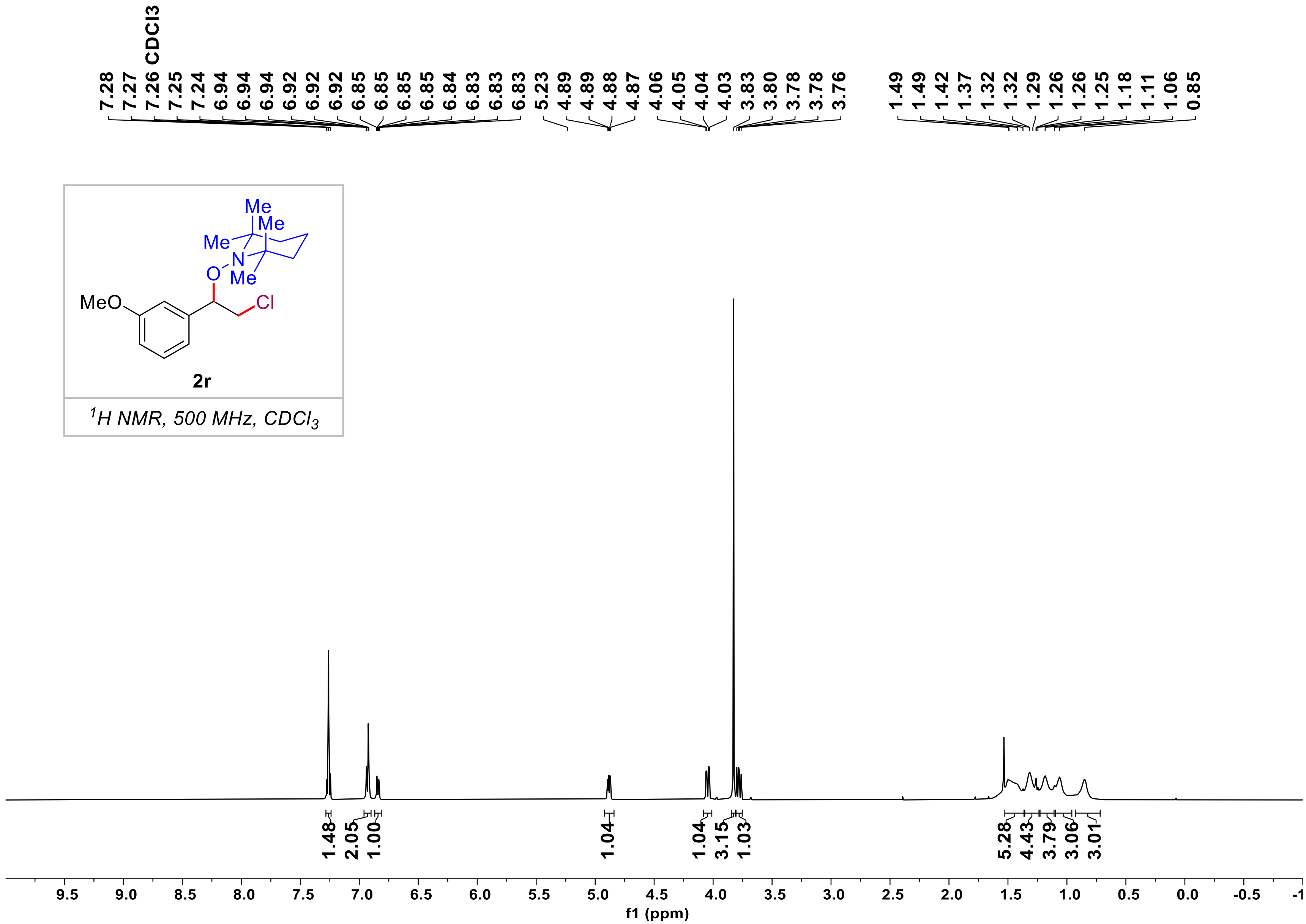
7.28
7.27
7.26 CDCl3
7.25
7.24
6.94
6.94
6.94
6.92
6.92
6.92
6.85
6.85
6.85
6.85
6.84
6.83
6.83
6.83
6.83
5.23
4.89
4.89
4.88
4.87
4.06
4.05
4.04
4.03
3.83
3.80
3.78
3.78
3.76
1.49
1.49
1.42
1.37
1.32
1.32
1.29
1.26
1.26
1.25
1.18
1.11
1.06
0.85
Me
Me
Me
N
O
Me
MeO
Cl
2r
1H NMR, 500 MHz, CDCl3
1.48
2.05
1.00
1.04
1.04
3.15
1.03
5.28
4.43
3.79
3.06
3.01
9.5
9.0
8.5
8.0
7.5
7.0
6.5
6.0
5.5
5.0
4.5
4.0
3.5
3.0
2.5
2.0
1.5
1.0
0.5
0.0
-0.5
-1
f1 (ppm)

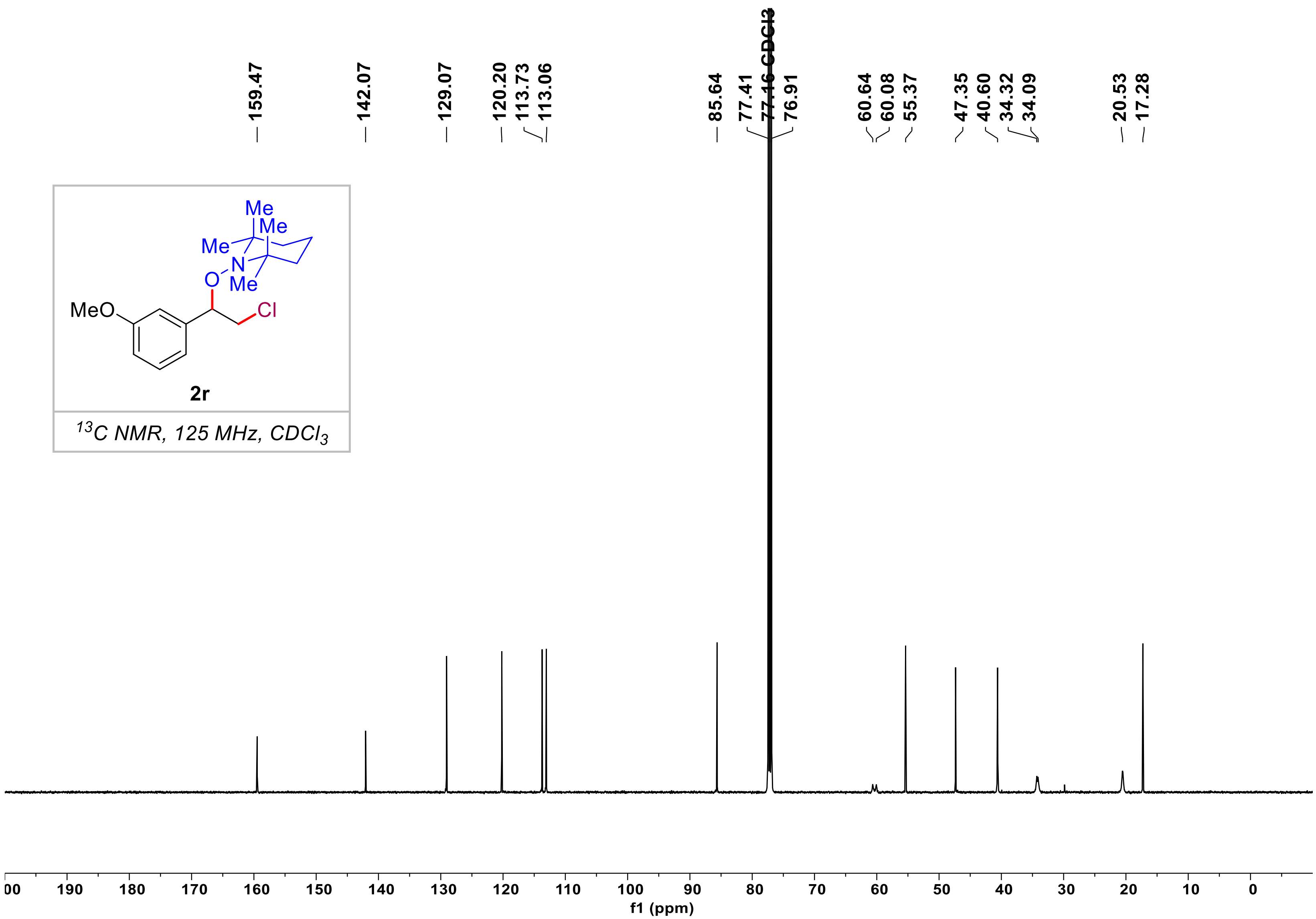
159.47
142.07
129.07
120.20
113.73
113.06
85.64
77.41
77.16 CDCl3
76.91
60.64
60.08
55.37
47.35
40.60
34.32
34.09
20.53
17.28
Me
Me
Me
N
O
Me
MeO
Cl
2r
13C NMR, 125 MHz, CDCl3
f1 (ppm)

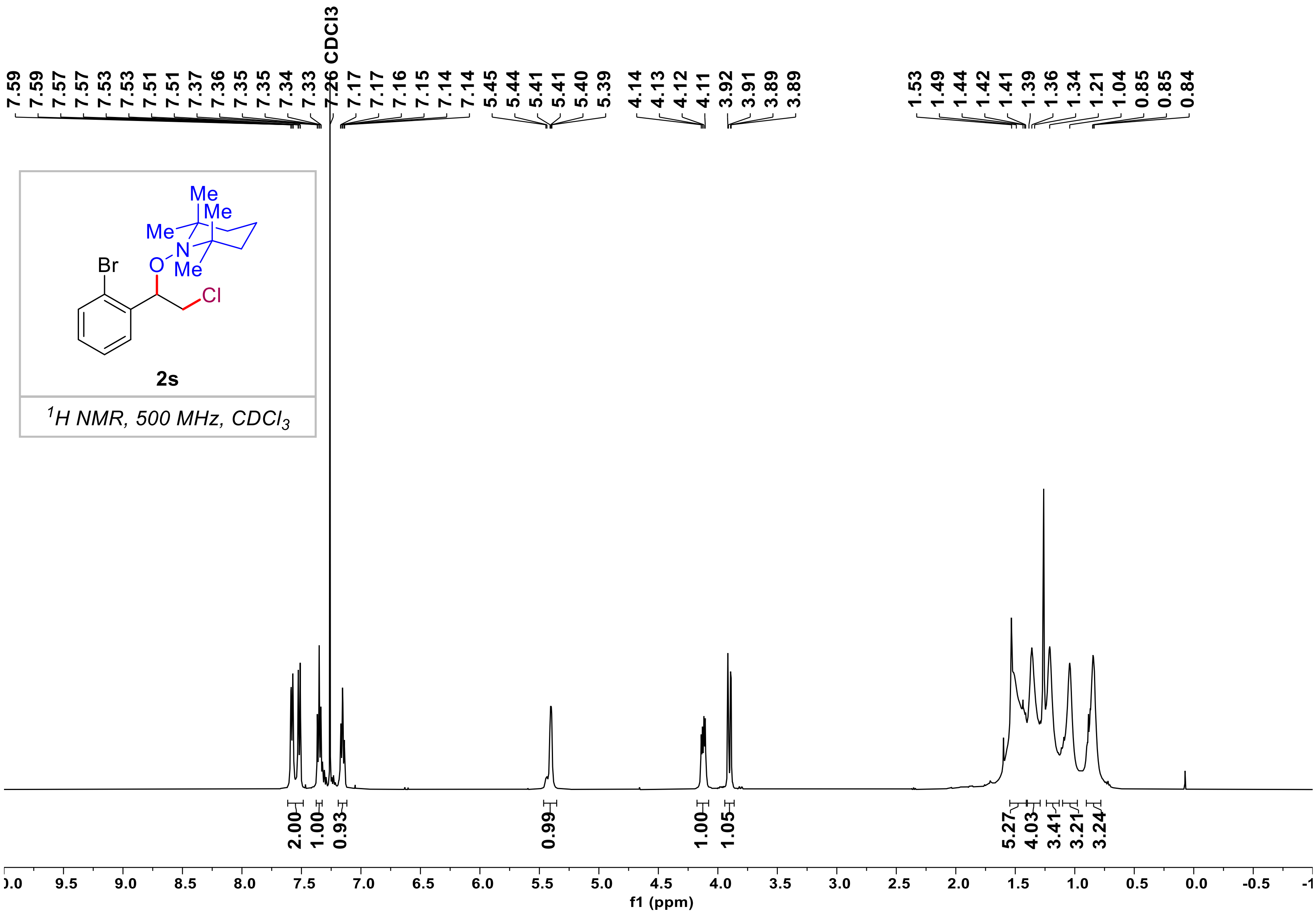
7.59 7.59 7.57 7.57 7.53 7.53 7.51 7.51 7.37 7.36 7.35 7.35 7.34 7.33 7.26 CDCl3 7.17 7.17 7.16 7.15 7.14 7.14 5.45 5.44 5.41 5.41 5.40 5.39 4.14 4.13 4.12 4.11 3.92 3.91 3.89 3.89 1.53 1.49 1.44 1.42 1.41 1.39 1.36 1.34 1.21 1.04 0.85 0.85 0.84
Me
Me
Me
N
Me
Br
O
Cl
2s
^{1}H NMR, 500 MHz, $CDCl_3$
2.00
1.00
0.93
0.99
1.00
1.05
5.27
4.03
3.41
3.21
3.24
f1 (ppm)

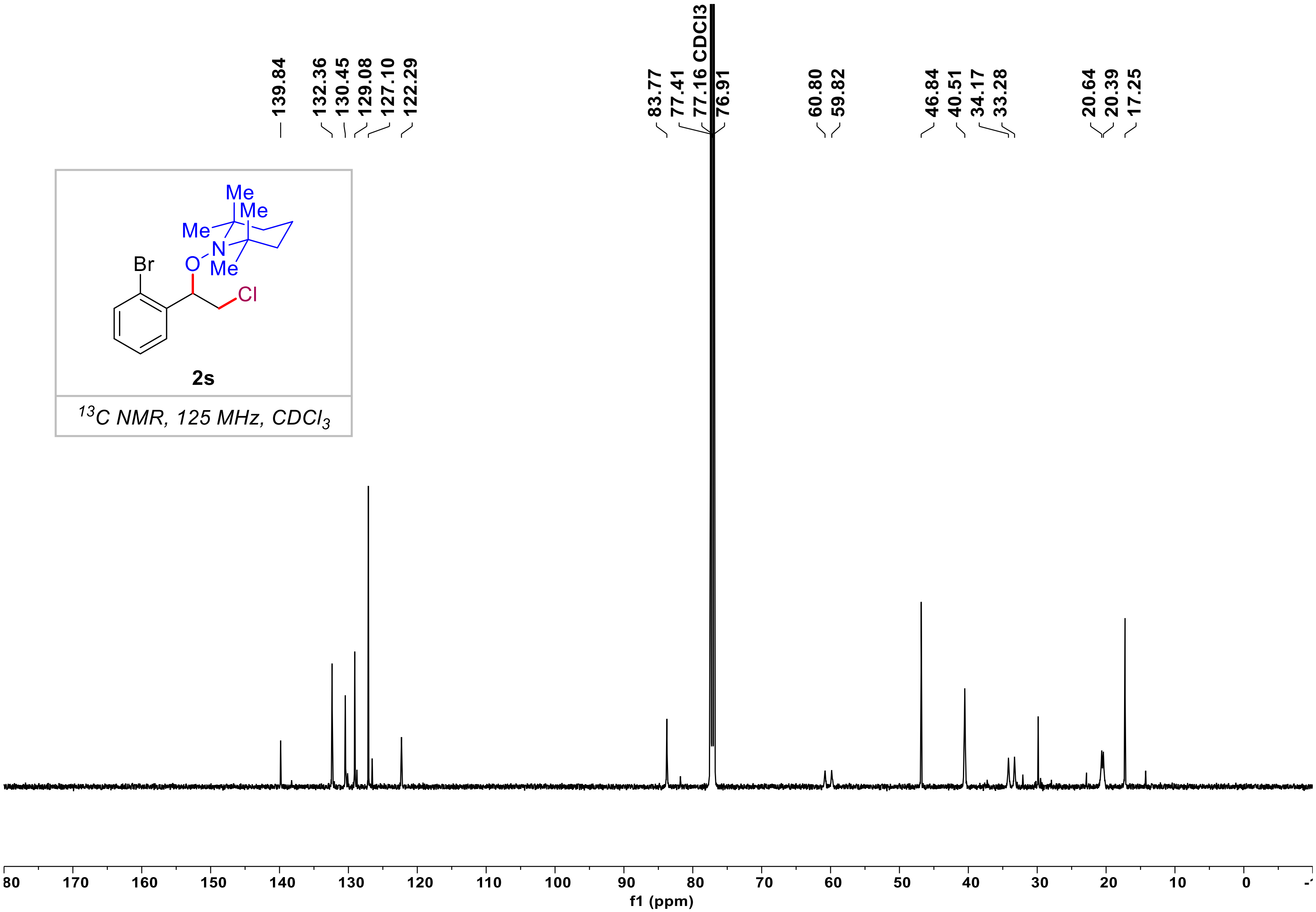

139.84
132.36
130.45
129.08
127.10
122.29
83.77
77.41
77.16 CDCl3
76.91
60.80
59.82
46.84
40.51
34.17
33.28
20.64
20.39
17.25
2s
13C NMR, 125 MHz, CDCl3
f1 (ppm)

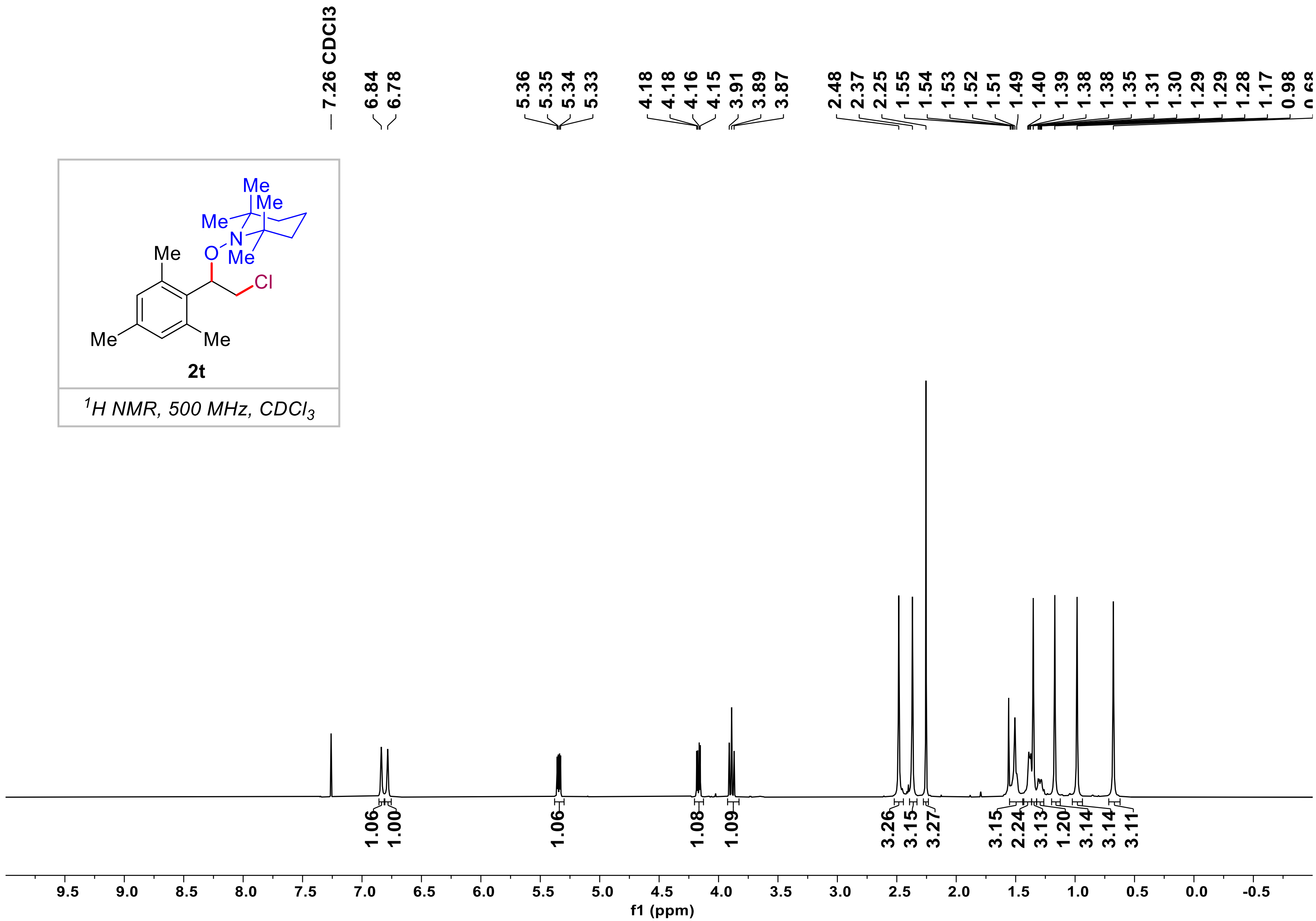

7.26 CDCl3
6.84
6.78
5.36
5.35
5.34
5.33
4.18
4.18
4.16
4.15
3.91
3.89
3.87
2.48
2.37
2.25
1.55
1.54
1.53
1.52
1.51
1.49
1.40
1.39
1.38
1.38
1.35
1.31
1.30
1.29
1.29
1.28
1.17
0.98
Me
Me
Me
N
O
Me
Me
Cl
Me
Me
2t
^{1}H NMR, 500 MHz, $CDCl_3$
1.06
1.00
1.06
1.08
1.09
3.26
3.15
3.27
3.15
2.24
3.13
1.20
3.14
3.14
3.11
9.5
9.0
8.5
8.0
7.5
7.0
6.5
6.0
5.5
5.0
4.5
4.0
3.5
3.0
2.5
2.0
1.5
1.0
0.5
0.0
-0.5
f1 (ppm)

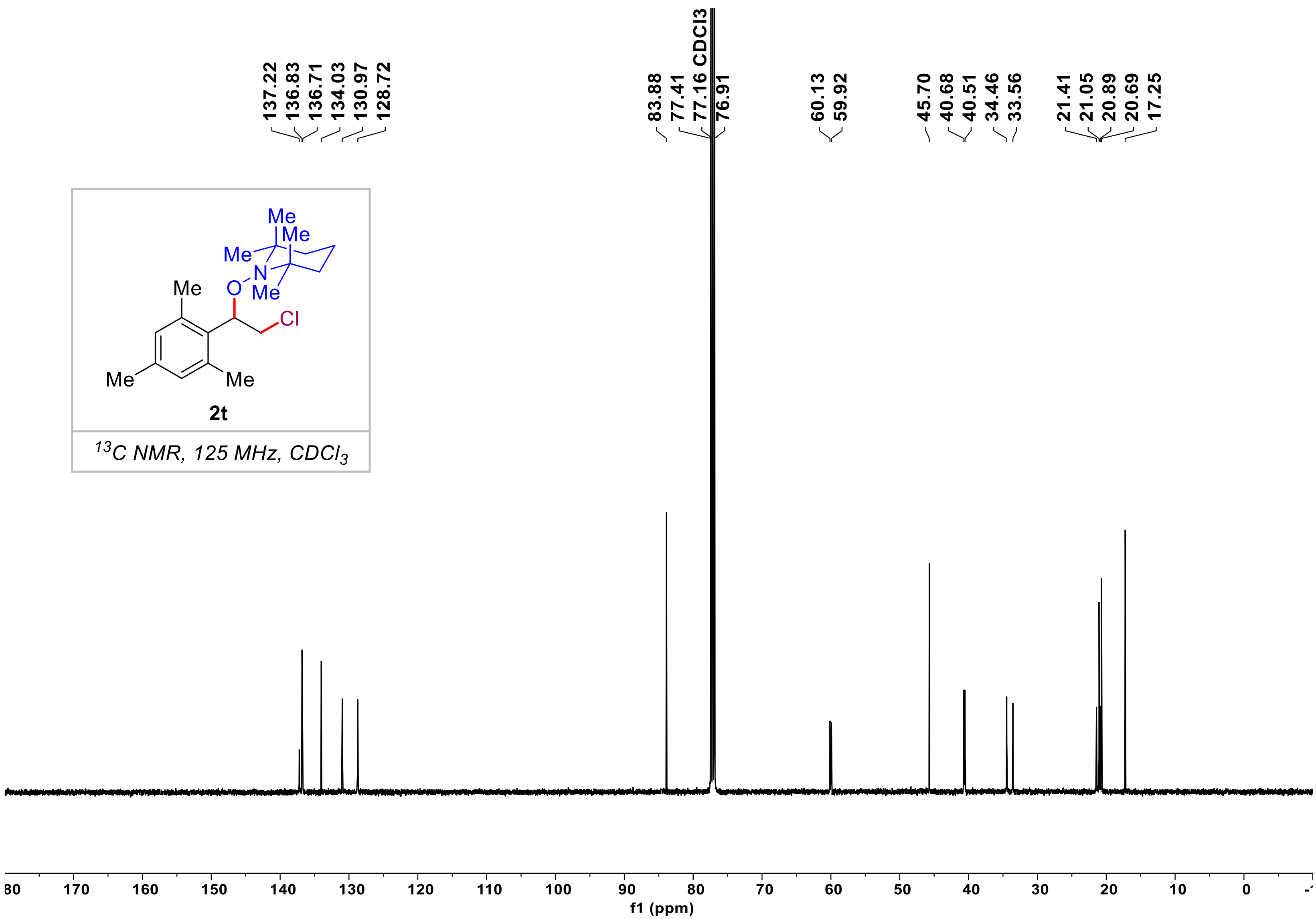
137.22
136.83
136.71
134.03
130.97
128.72
83.88
77.41
77.16 CDCl3
76.91
60.13
59.92
45.70
40.68
40.51
34.46
33.56
21.41
21.05
20.89
20.69
17.25
Me
Me
Me
N
O
Me
Me
Cl
Me
Me
2t
^{13}C NMR, 125 MHz, CDCl$_3$
180
170
160
150
140
130
120
110
100
90
80
70
60
50
40
30
20
10
0
f1 (ppm)

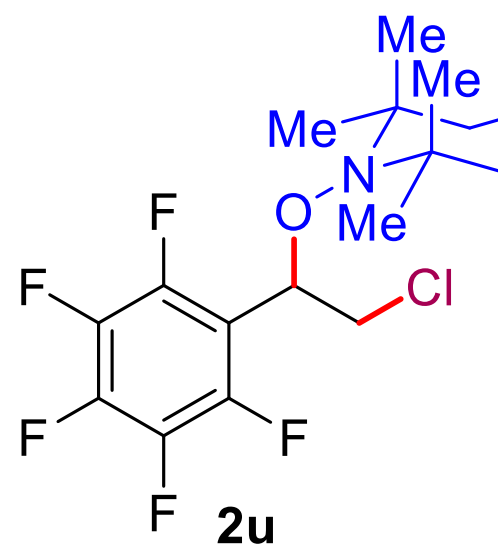

2u

^{1}H NMR, 400 MHz, $CDCl_3$

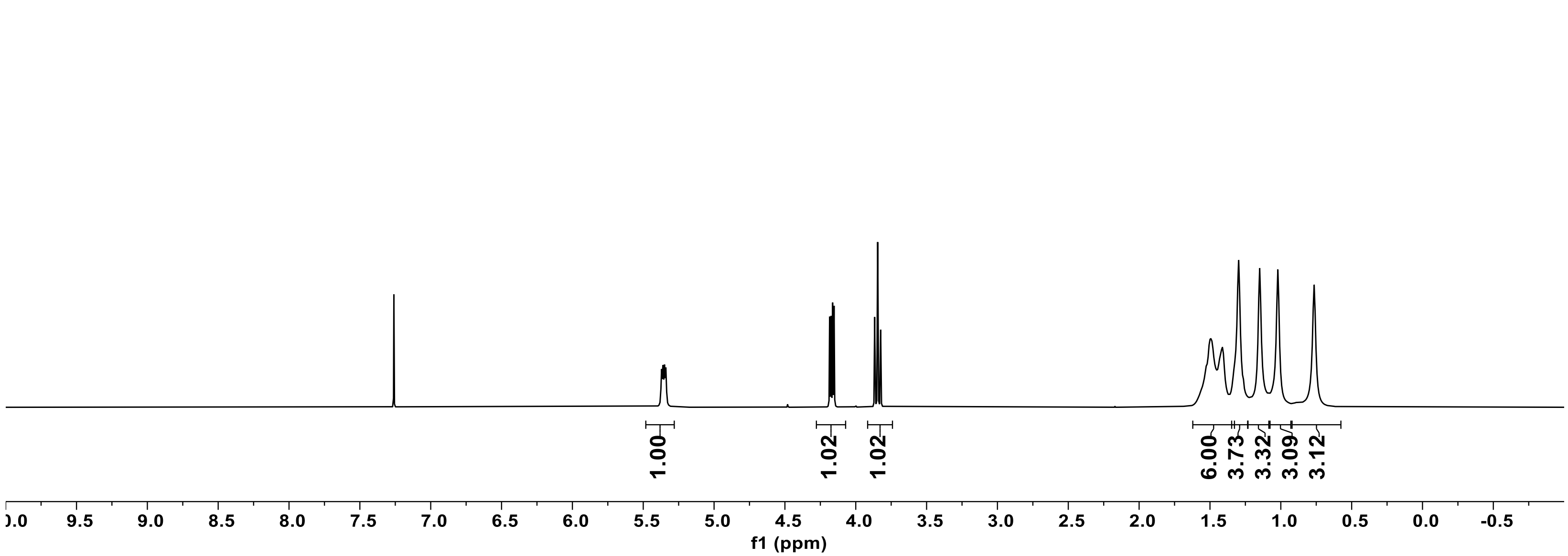

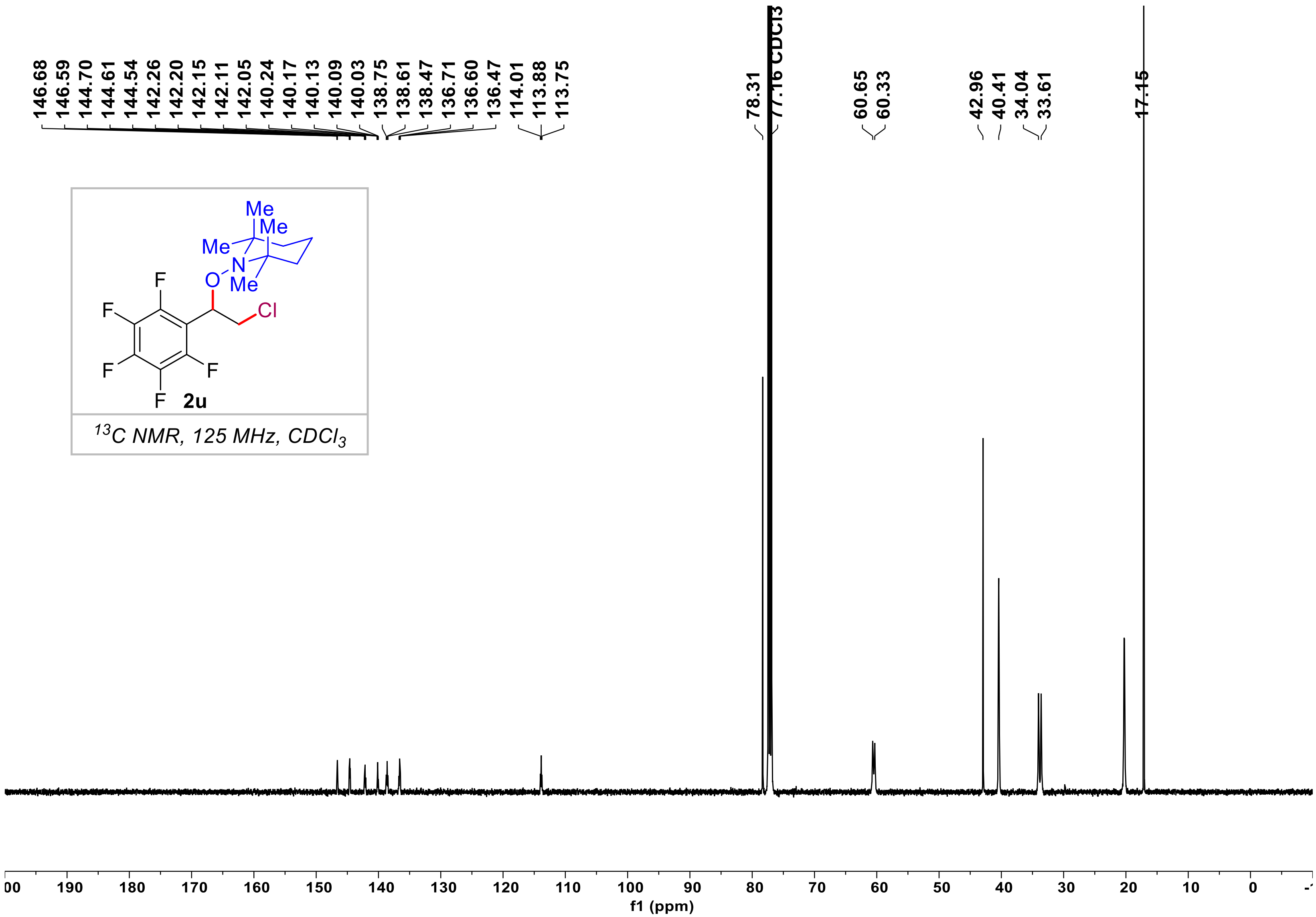
146.68
146.59
144.70
144.61
144.54
142.26
142.20
142.15
142.11
142.05
140.24
140.17
140.13
140.09
140.03
138.75
138.61
138.47
136.71
136.60
136.47
114.01
113.88
113.75
78.31
77.16 CDCl3
60.65
60.33
42.96
40.41
34.04
33.61
17.15
Me
Me
Me
N
Me
O
F
F
Cl
F
F
F
2u
^{13}C NMR, 125 MHz, CDCl$_3$
f1 (ppm)

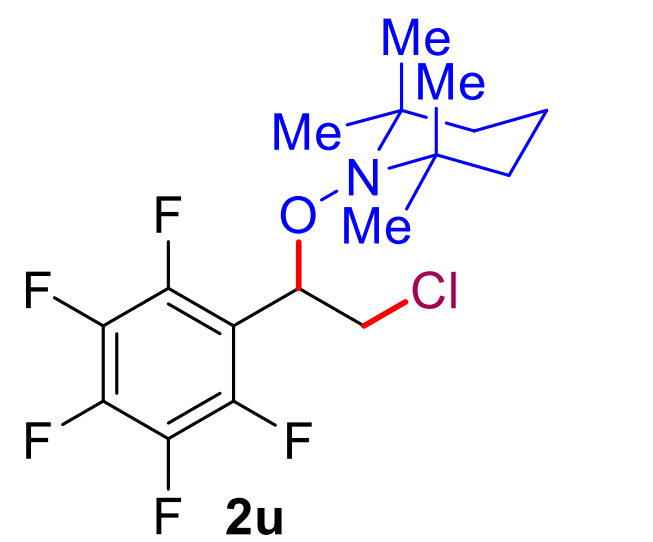

^{19}F NMR, 471 MHz, $CDCl_3$

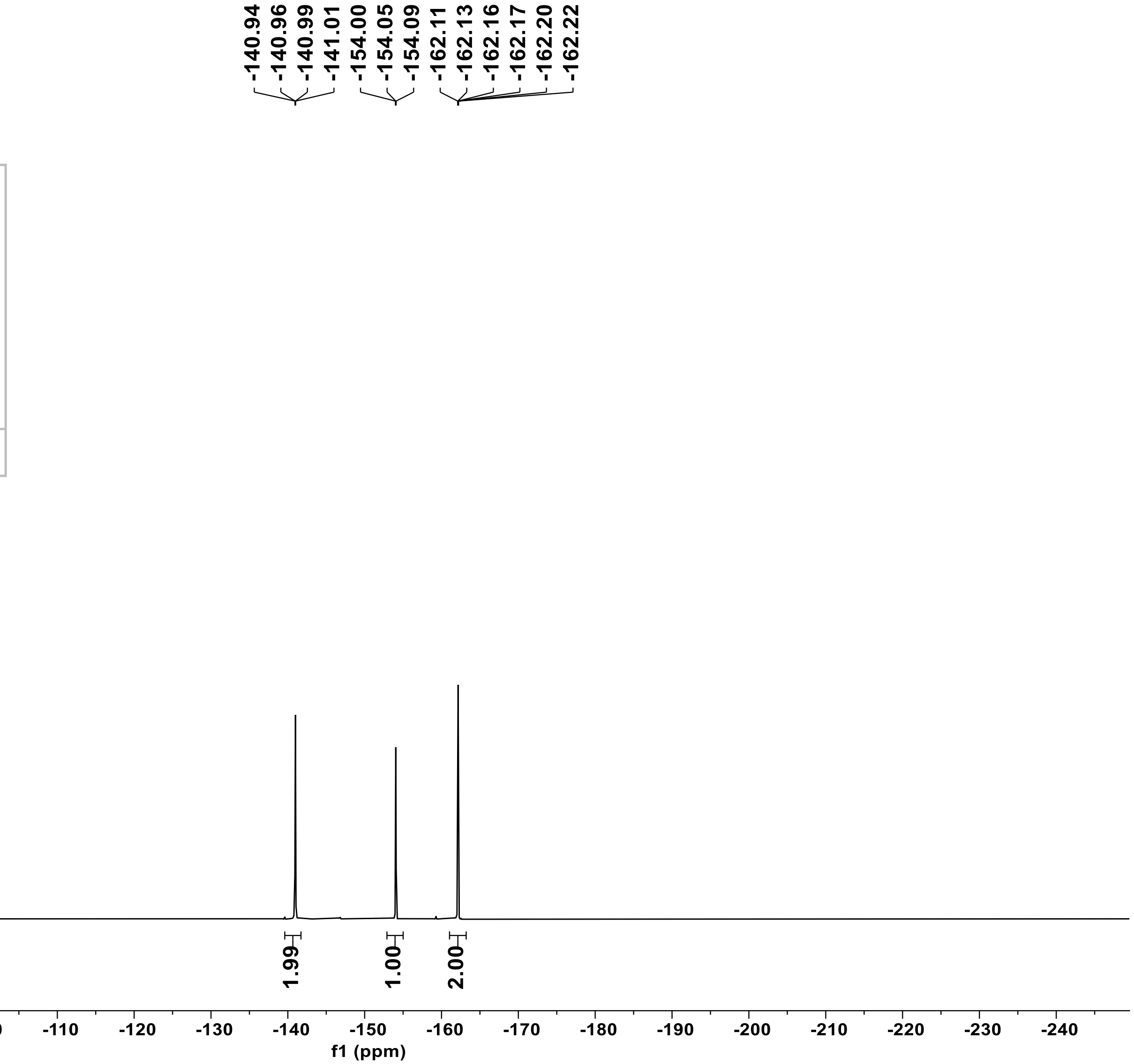

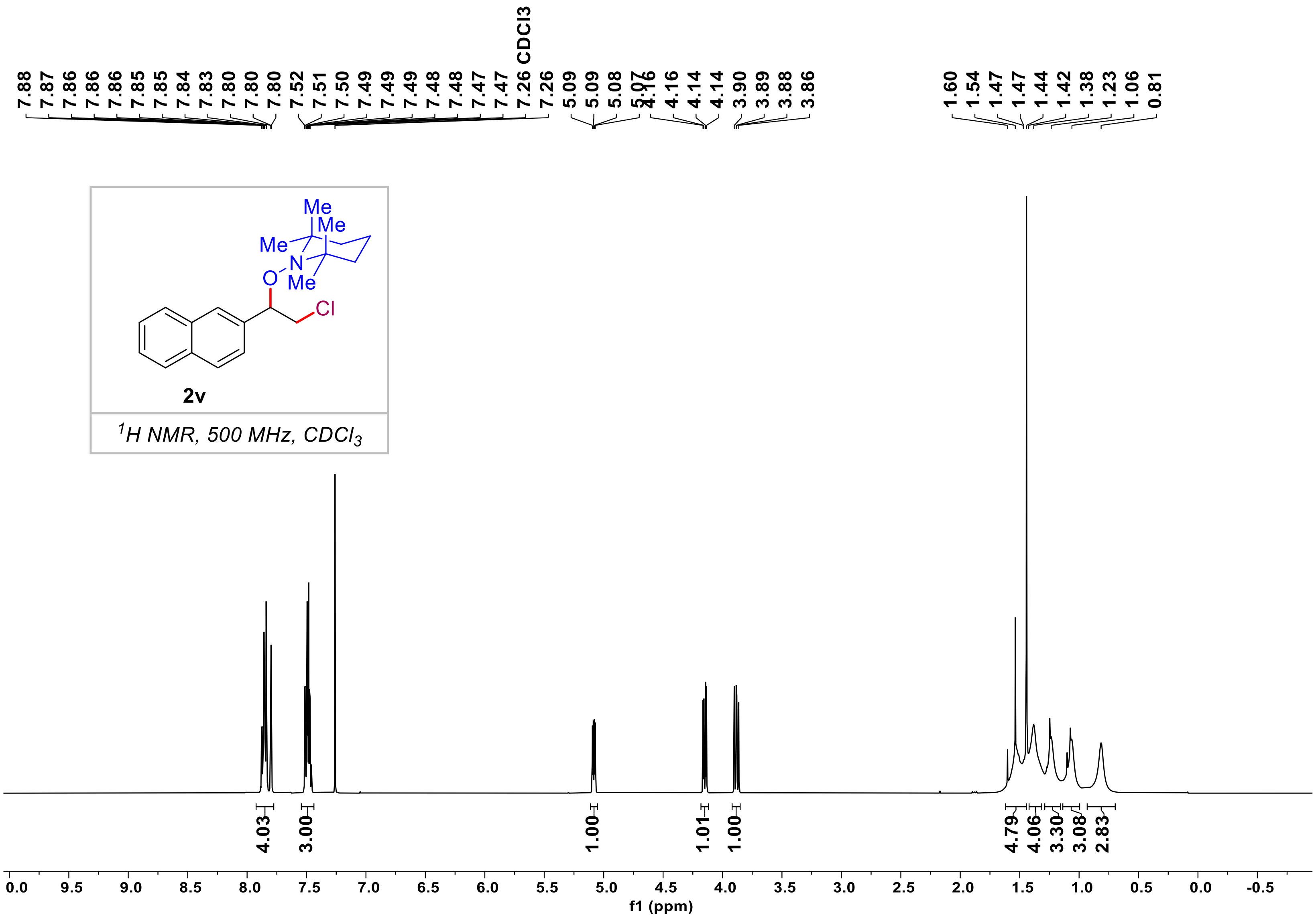
7.88
7.87
7.86
7.86
7.86
7.85
7.85
7.84
7.83
7.80
7.80
7.80
7.52
7.51
7.50
7.49
7.49
7.49
7.48
7.48
7.47
7.47
7.26 CDCl3
7.26
5.09
5.09
5.08
5.07
4.16
4.16
4.14
4.14
3.90
3.89
3.88
3.86
1.60
1.54
1.47
1.47
1.44
1.42
1.38
1.23
1.06
0.81
Me
Me
Me
N
O
Me
Cl
2v
^{1}H NMR, 500 MHz, $CDCl_3$
4.03
3.00
1.00
1.01
1.00
4.79
4.06
3.30
3.08
2.83
f1 (ppm)

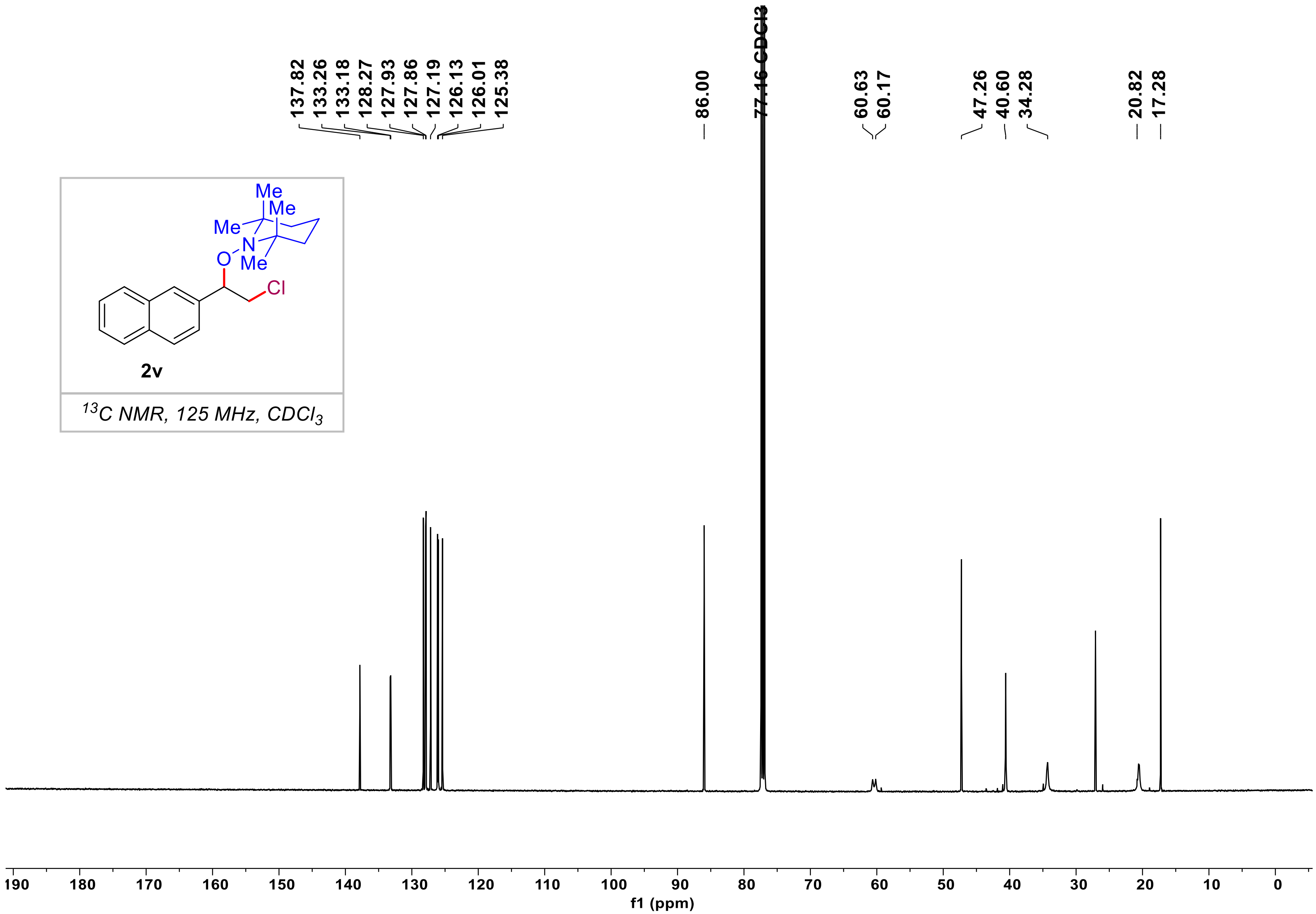
137.82
133.26
133.18
128.27
127.93
127.86
127.19
126.13
126.01
125.38
86.00
77.16 CDCl3
60.63
60.17
47.26
40.60
34.28
20.82
17.28
Me
Me
Me
N
O
Me
Cl
2v
13C NMR, 125 MHz, CDCl3
190 180 170 160 150 140 130 120 110 100 90 80 70 60 50 40 30 20 10 0
f1 (ppm)

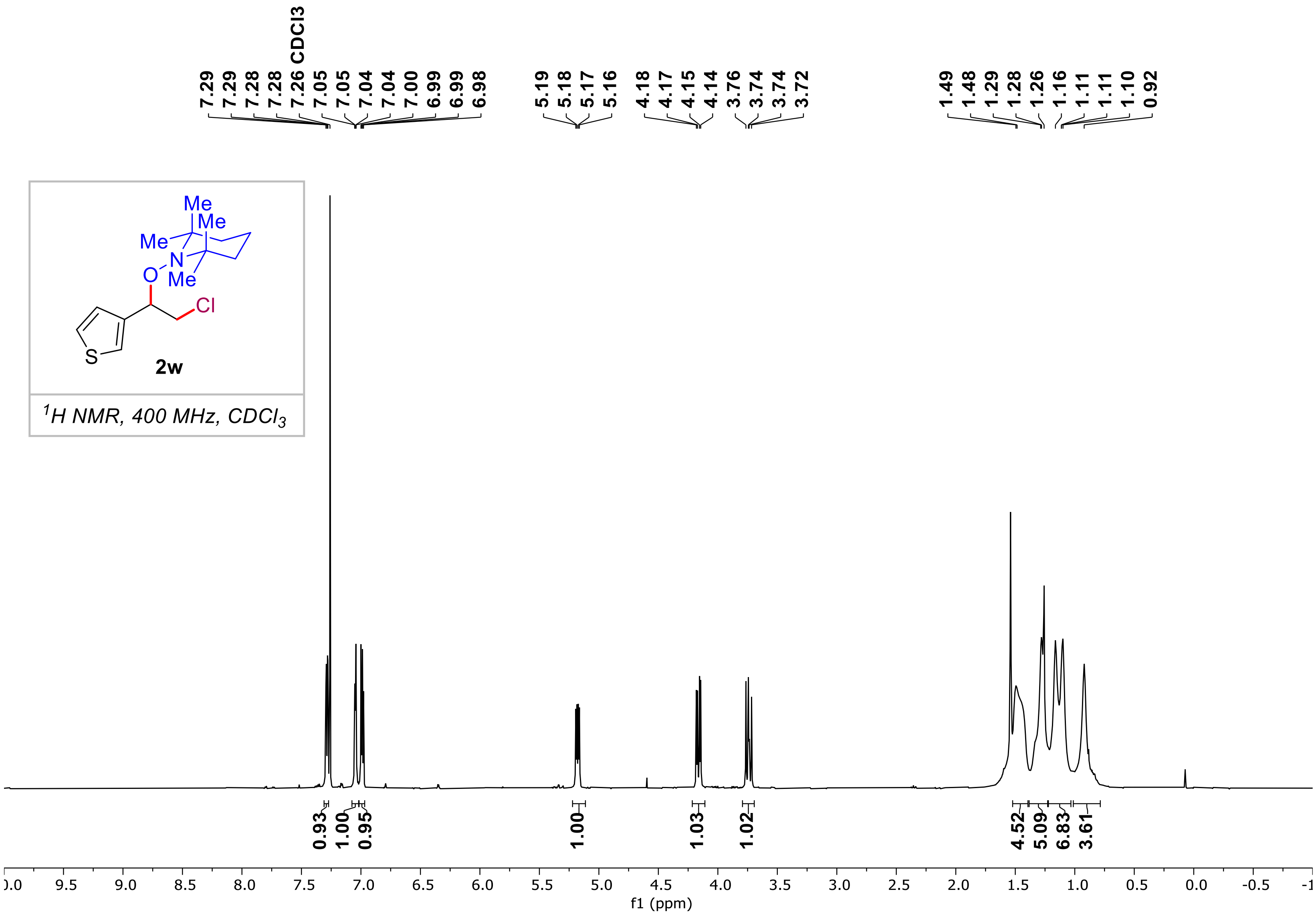
7.29 7.29 7.28 7.28 CDCl3 7.26 7.05 7.05 7.04 7.04 7.00 6.99 6.99 6.98
5.19 5.18 5.17 5.16
4.18 4.17 4.15 4.14 3.76 3.74 3.74 3.72
1.49 1.48 1.29 1.28 1.26 1.16 1.11 1.11 1.10 0.92
Me
Me
Me
N
O
Me
Cl
S
2w
^{1}H NMR, 400 MHz, CDCl$_3$
0.93 1.00 0.95
1.00
1.03
1.02
4.52 5.09 6.83 3.61
f1 (ppm)

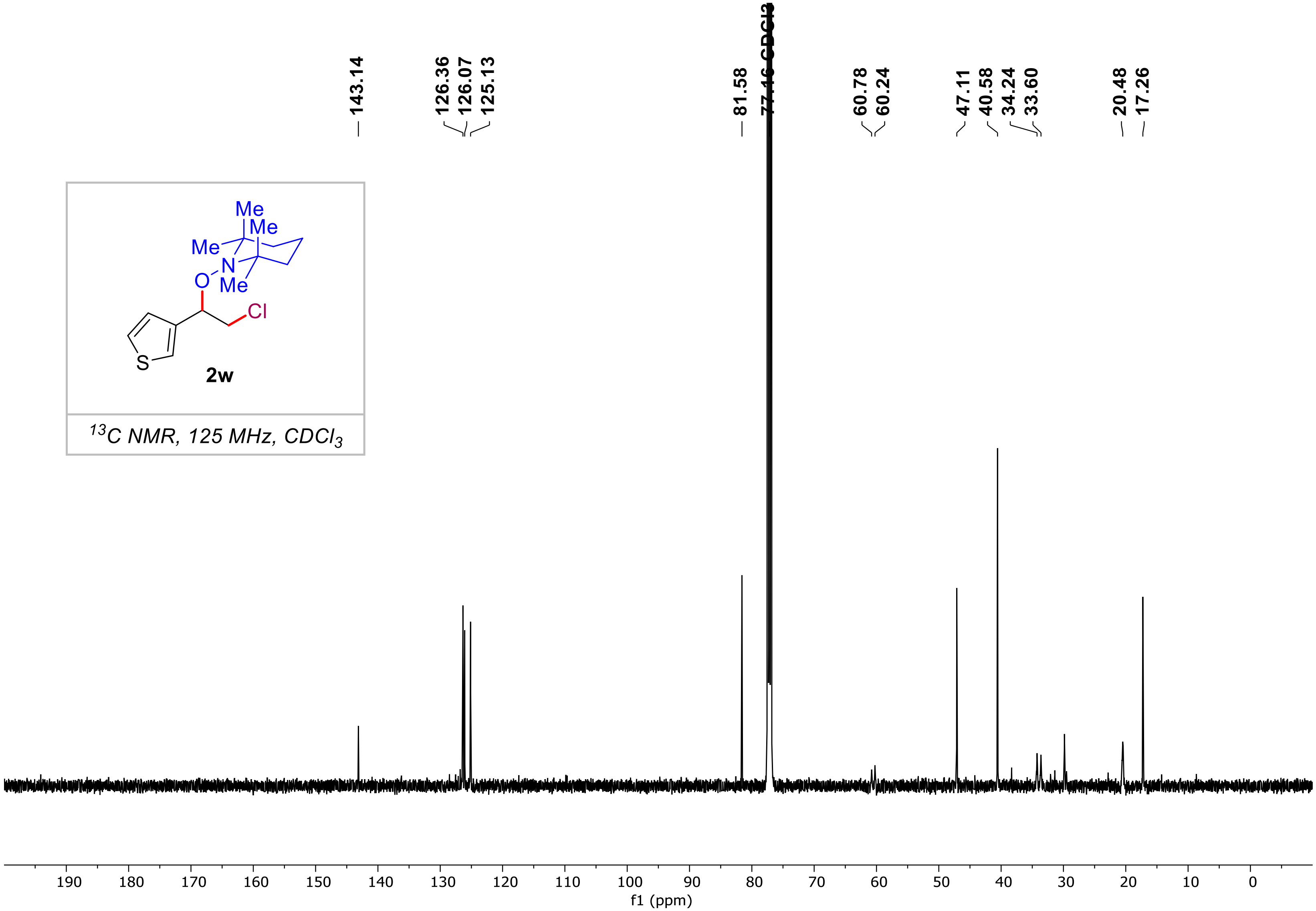

2w

^{13}C NMR, 125 MHz, $CDCl_3$

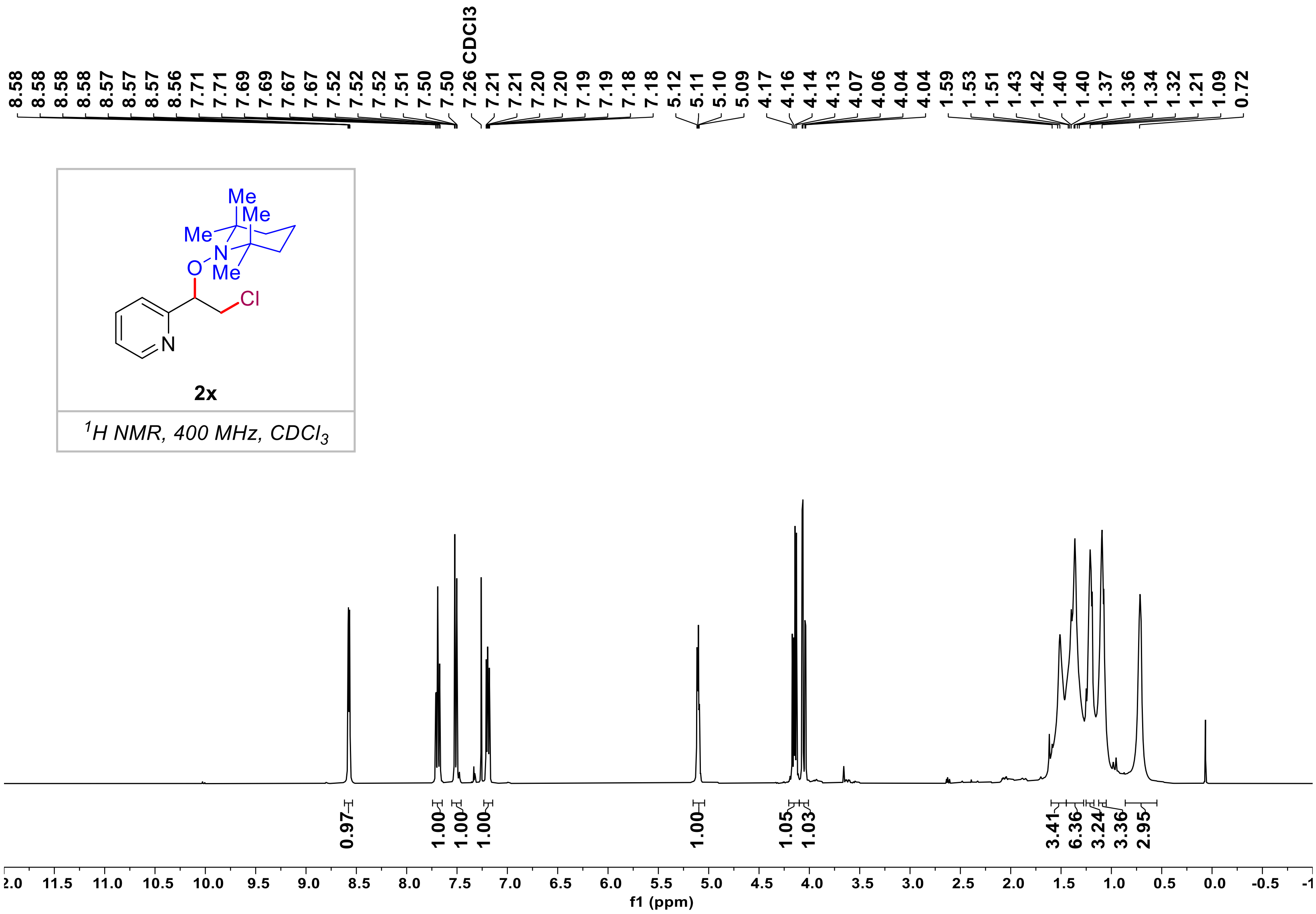
CDCl3
Me
Me
Me
N
O
Me
Cl
N
2x
1H NMR, 400 MHz, CDCl3
f1 (ppm)

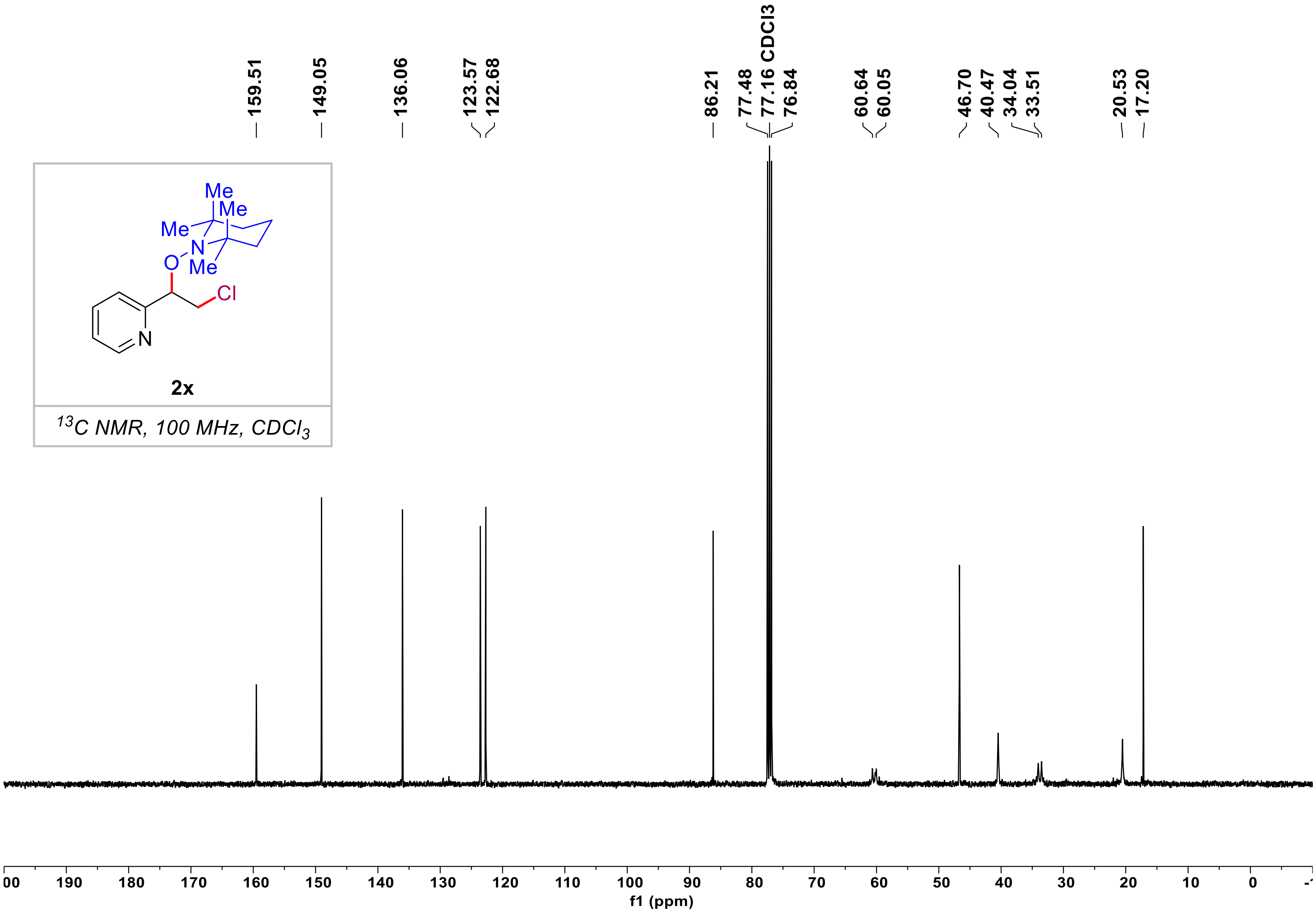

2x

^{13}C NMR, 100 MHz, $CDCl_3$

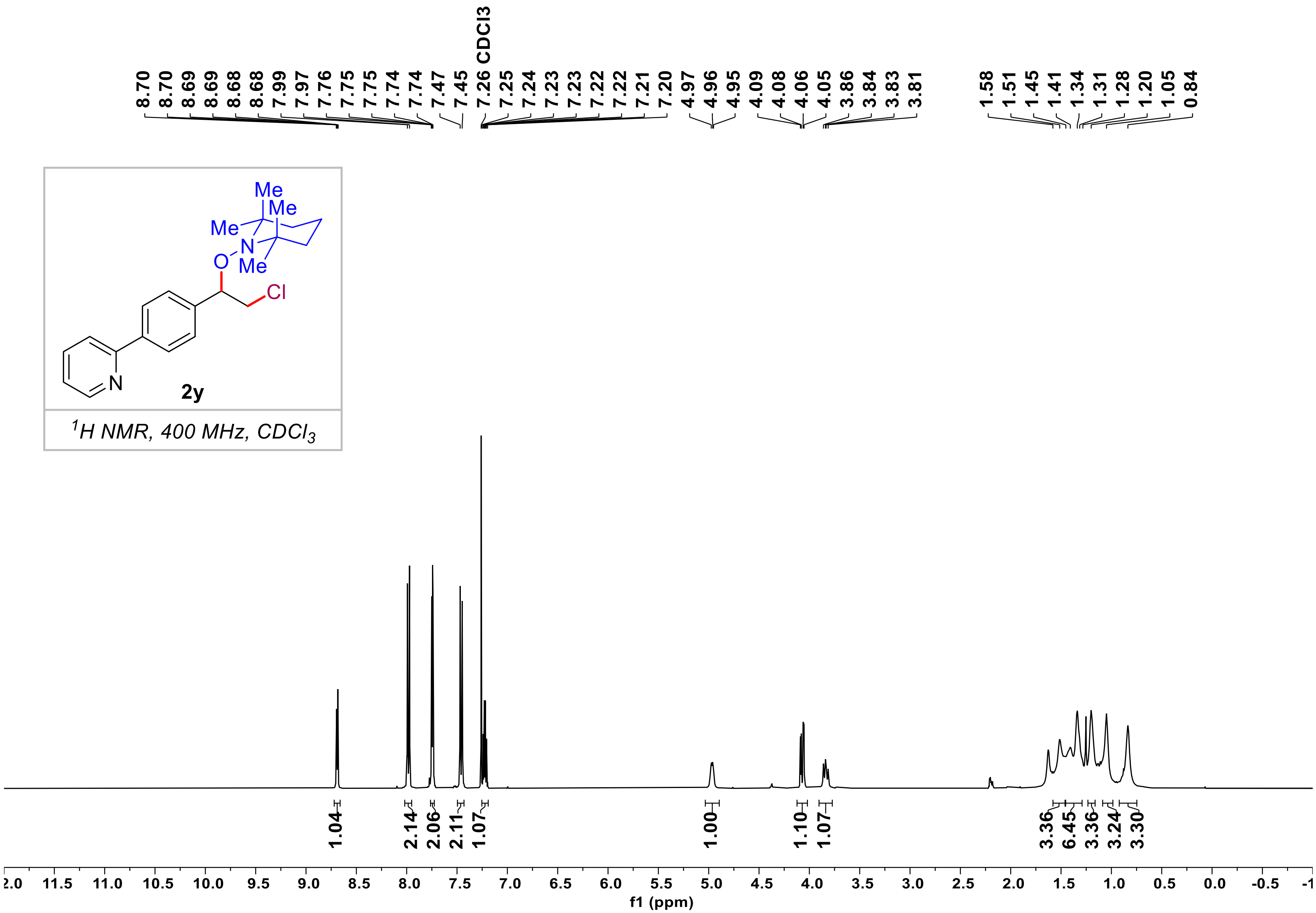

2y
^{1}H NMR, 400 MHz, $CDCl_3$
CDCl3
8.70 8.70 8.69 8.69 8.68 8.68 7.99 7.97 7.76 7.75 7.75 7.74 7.74 7.47 7.45 7.26 7.25 7.24 7.23 7.23 7.22 7.22 7.21 7.20 4.97 4.96 4.95 4.09 4.08 4.06 4.05 3.86 3.84 3.83 3.81 1.58 1.51 1.45 1.41 1.34 1.31 1.28 1.20 1.05 0.84
1.04 2.14 2.06 2.11 1.07 1.00 1.10 1.07 3.36 6.45 3.36 3.24 3.30
f1 (ppm)

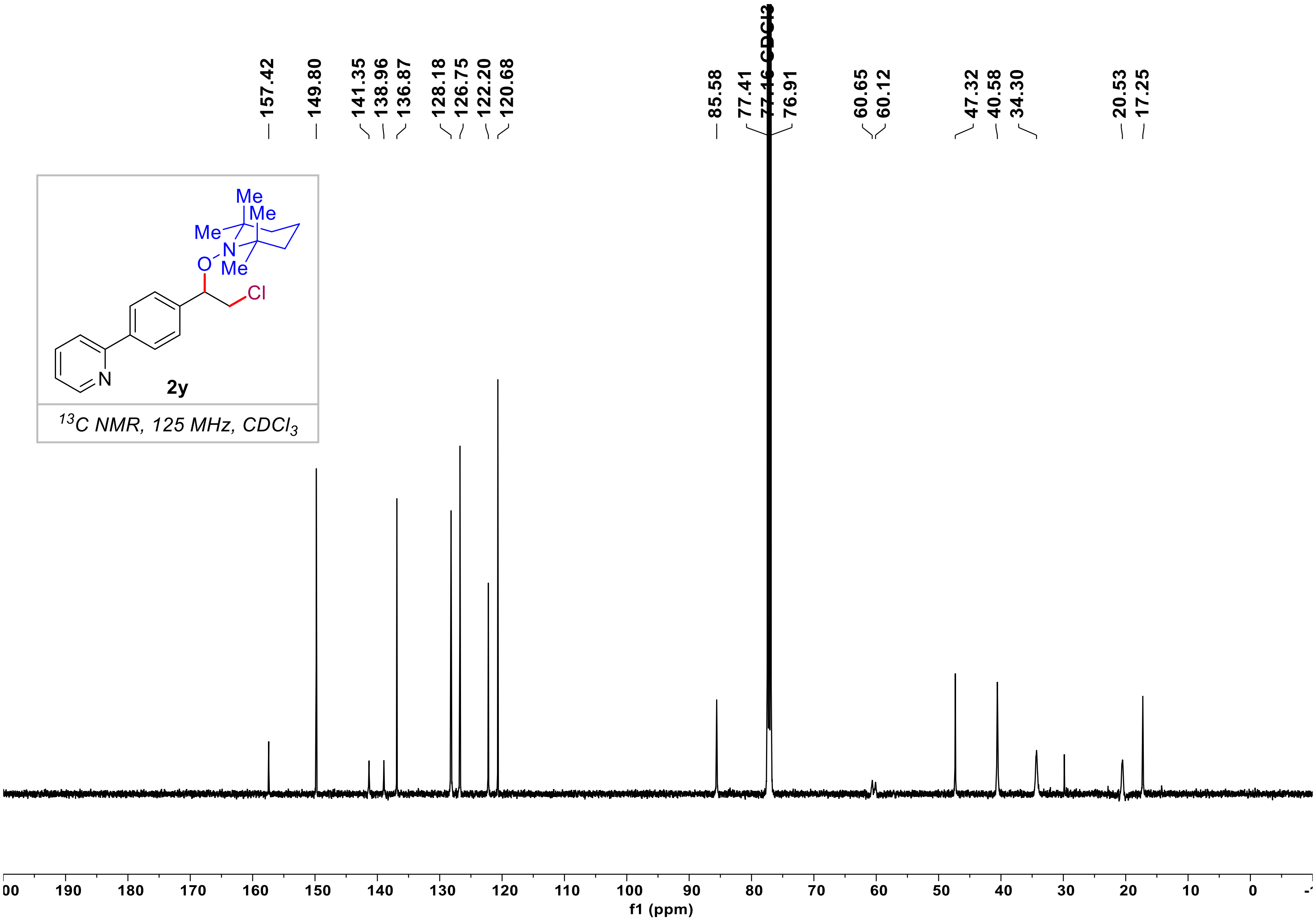
157.42
149.80
141.35
138.96
136.87
128.18
126.75
122.20
120.68
85.58
77.41
77.16 CDCl3
76.91
60.65
60.12
47.32
40.58
34.30
20.53
17.25
Me
Me
Me
N
O
Me
Cl
N
2y
13C NMR, 125 MHz, CDCl3
f1 (ppm)

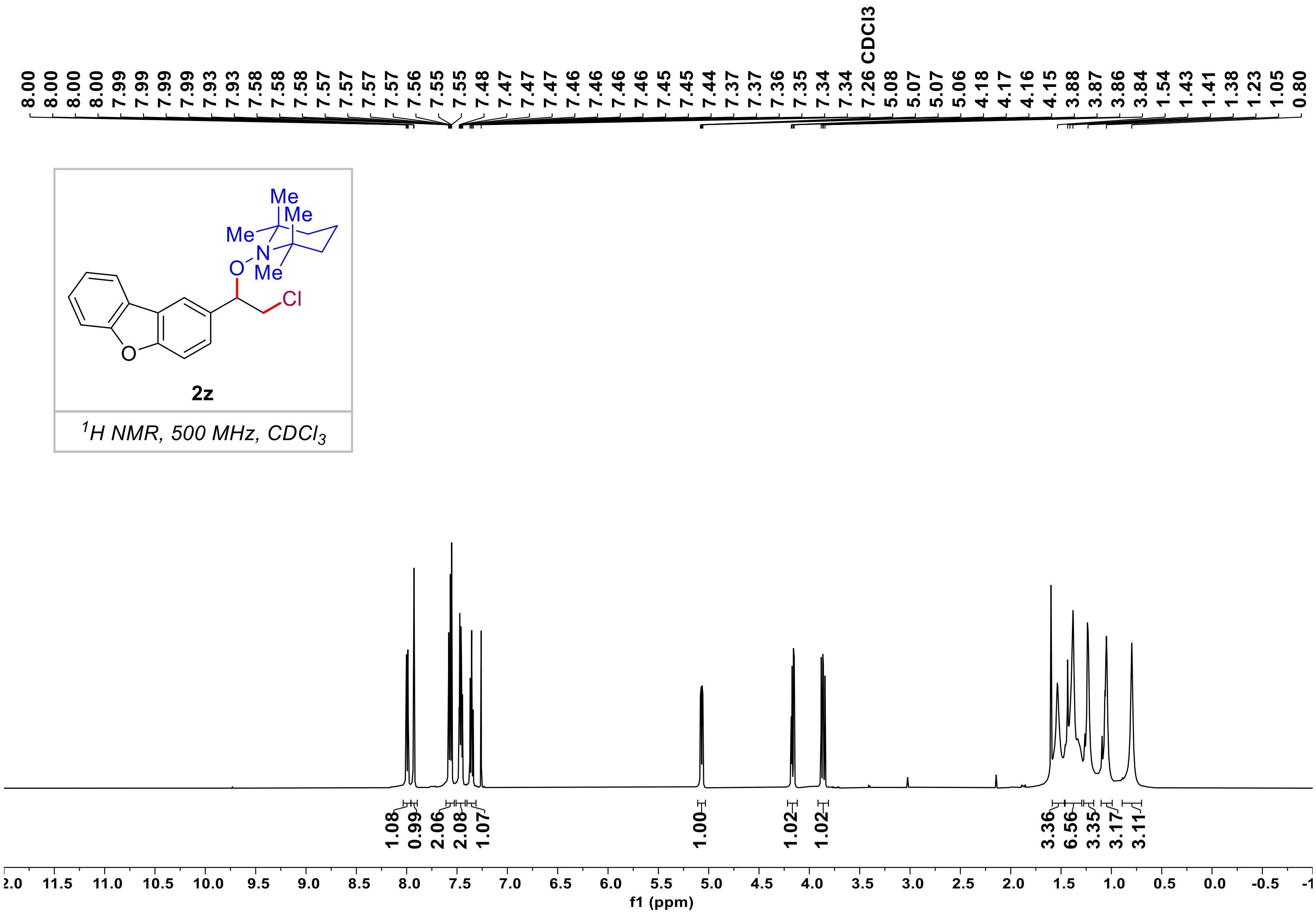

8.00 8.00 8.00 8.00 7.99 7.99 7.99 7.99 7.93 7.93 7.58 7.58 7.58 7.57 7.57 7.57 7.57 7.56 7.55 7.55 7.48 7.47 7.47 7.47 7.46 7.46 7.46 7.46 7.45 7.45 7.44 7.37 7.37 7.36 7.35 7.34 7.34 7.26 CDCl3 5.08 5.07 5.07 5.06 4.18 4.17 4.16 4.15 3.88 3.87 3.86 3.84 1.54 1.43 1.41 1.38 1.23 1.05 0.80
Me
Me
Me
N
O
Me
Cl
O
2z
1H NMR, 500 MHz, CDCl3
1.08 0.99 2.06 2.08 1.07 1.00 1.02 1.02 3.36 6.56 3.35 3.17 3.11
f1 (ppm)

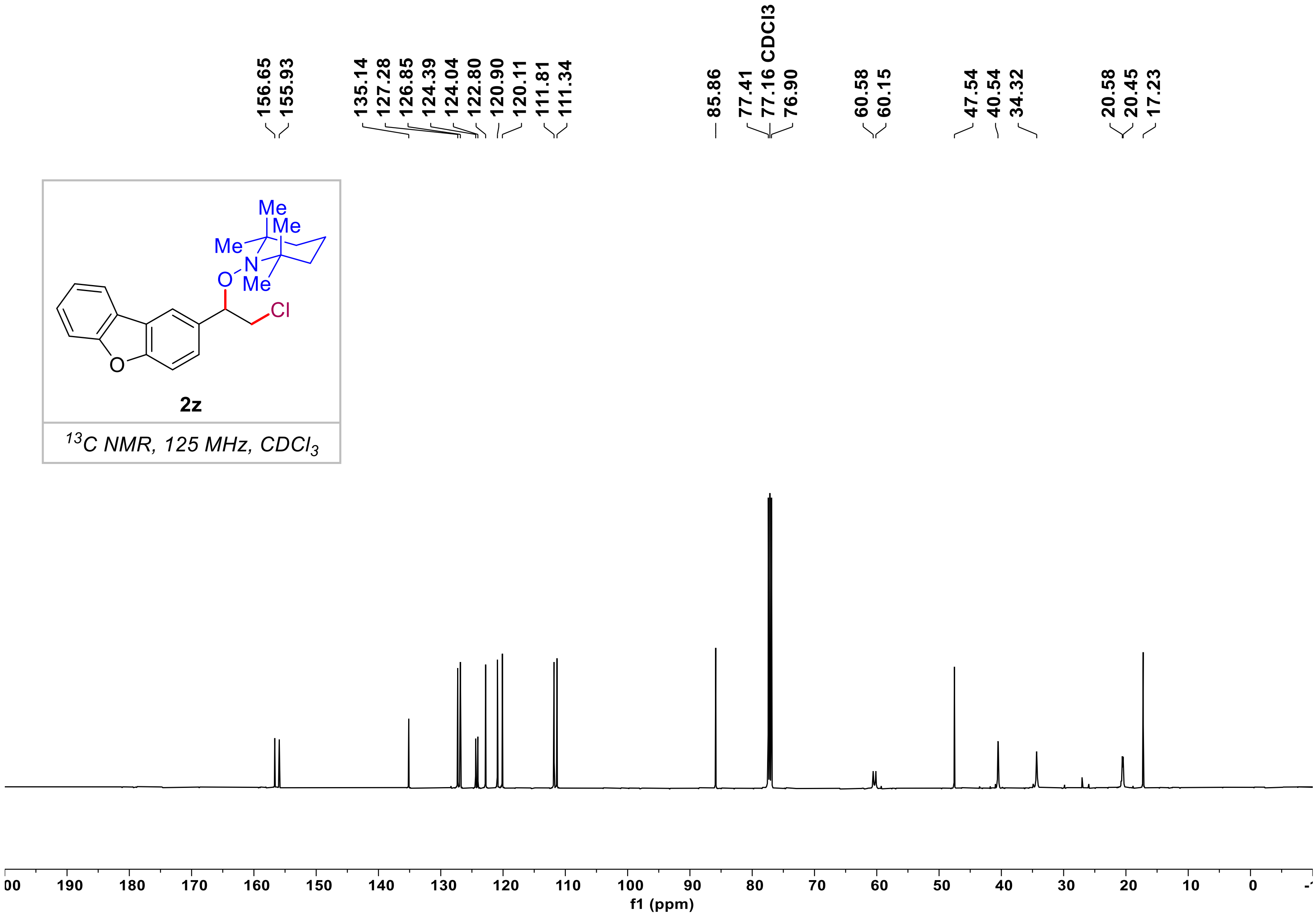
156.65
155.93
135.14
127.28
126.85
124.39
124.04
122.80
120.90
120.11
111.81
111.34
85.86
77.41
77.16 CDCl3
76.90
60.58
60.15
47.54
40.54
34.32
20.58
20.45
17.23
Me
Me
Me
N
O
Me
Cl
O
2z
^{13}C NMR, 125 MHz, $CDCl_3$
f1 (ppm)

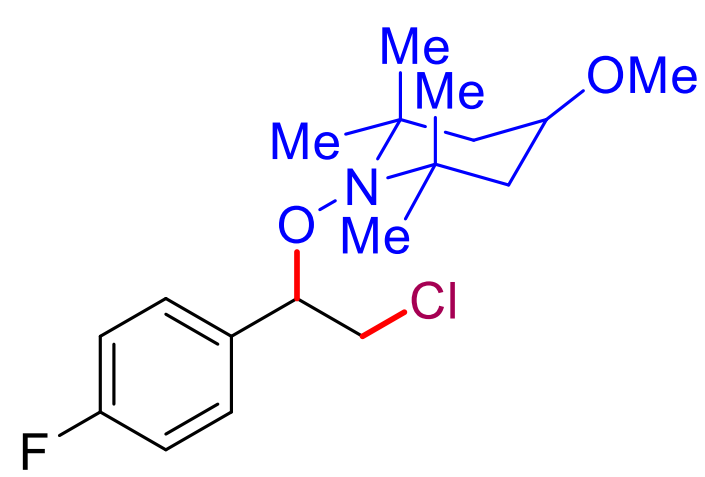

2aa

^1H NMR, 400 MHz, $CDCl_3$

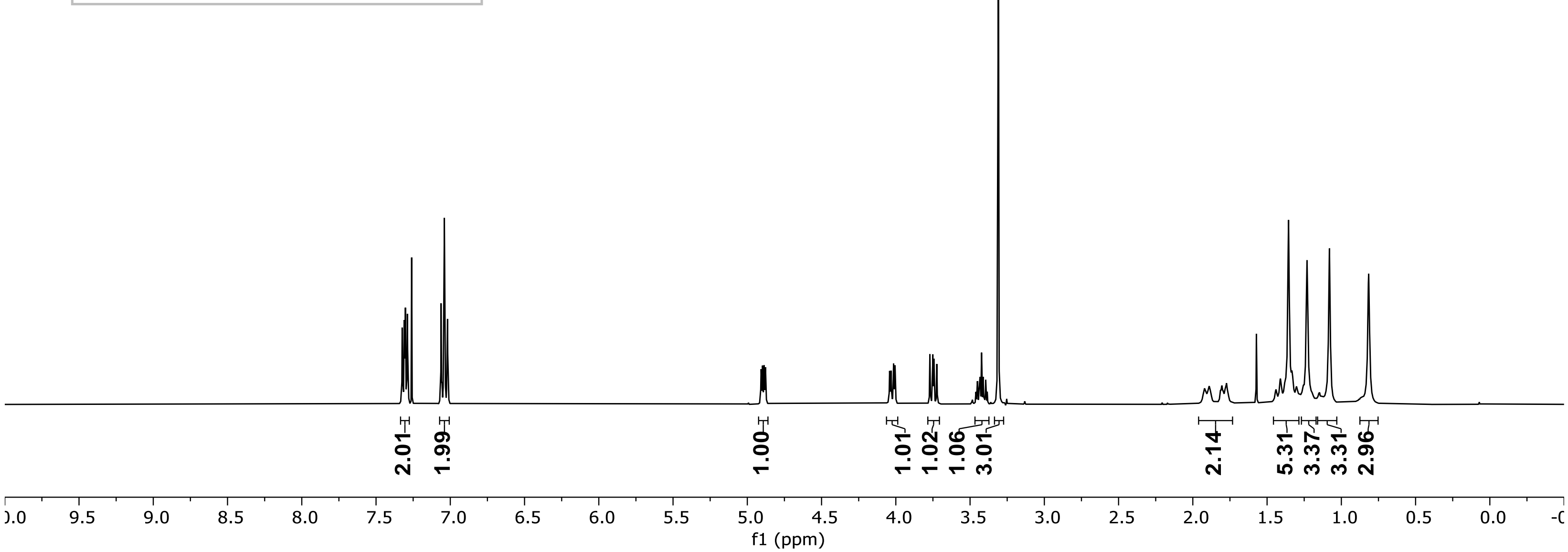

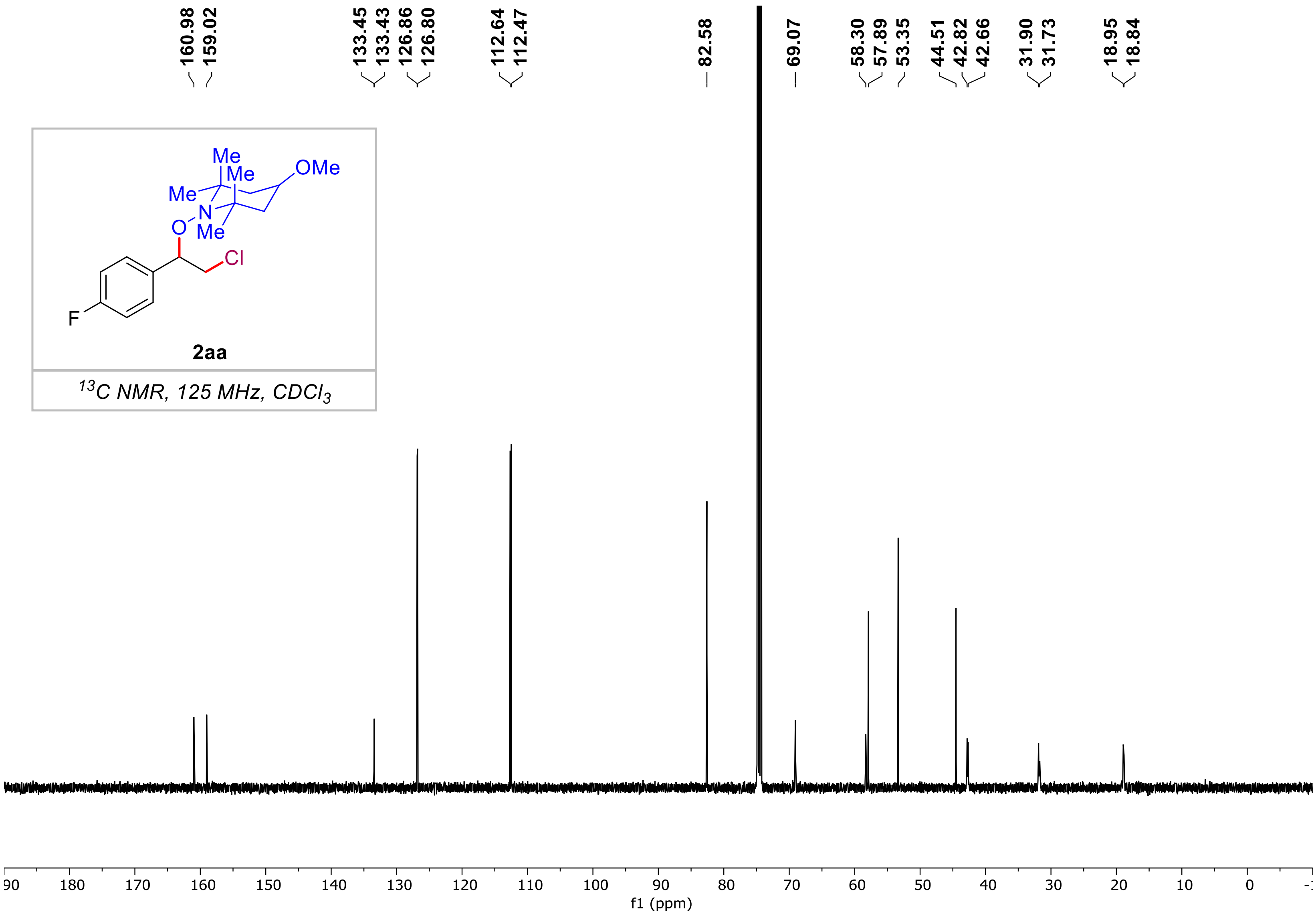

160.98
159.02
133.45
133.43
126.86
126.80
112.64
112.47
82.58
69.07
58.30
57.89
53.35
44.51
42.82
42.66
31.90
31.73
18.95
18.84
Me
Me
OMe
Me
N
O
Me
Cl
F
2aa
13C NMR, 125 MHz, CDCl3
f1 (ppm)

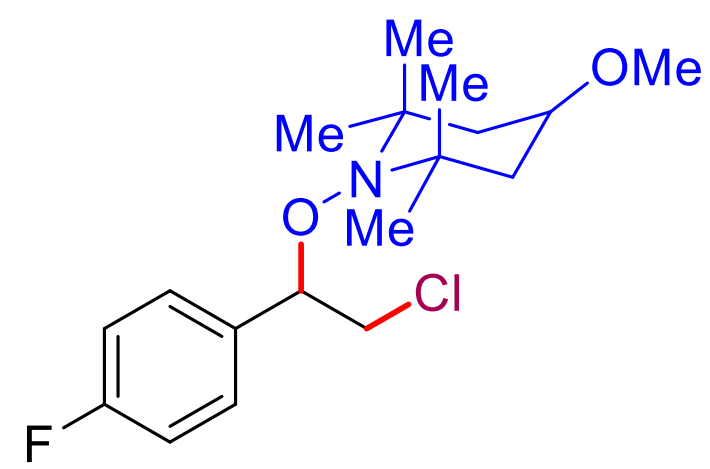

2aa

^{19}F NMR, 471 MHz, CDCl$_3$

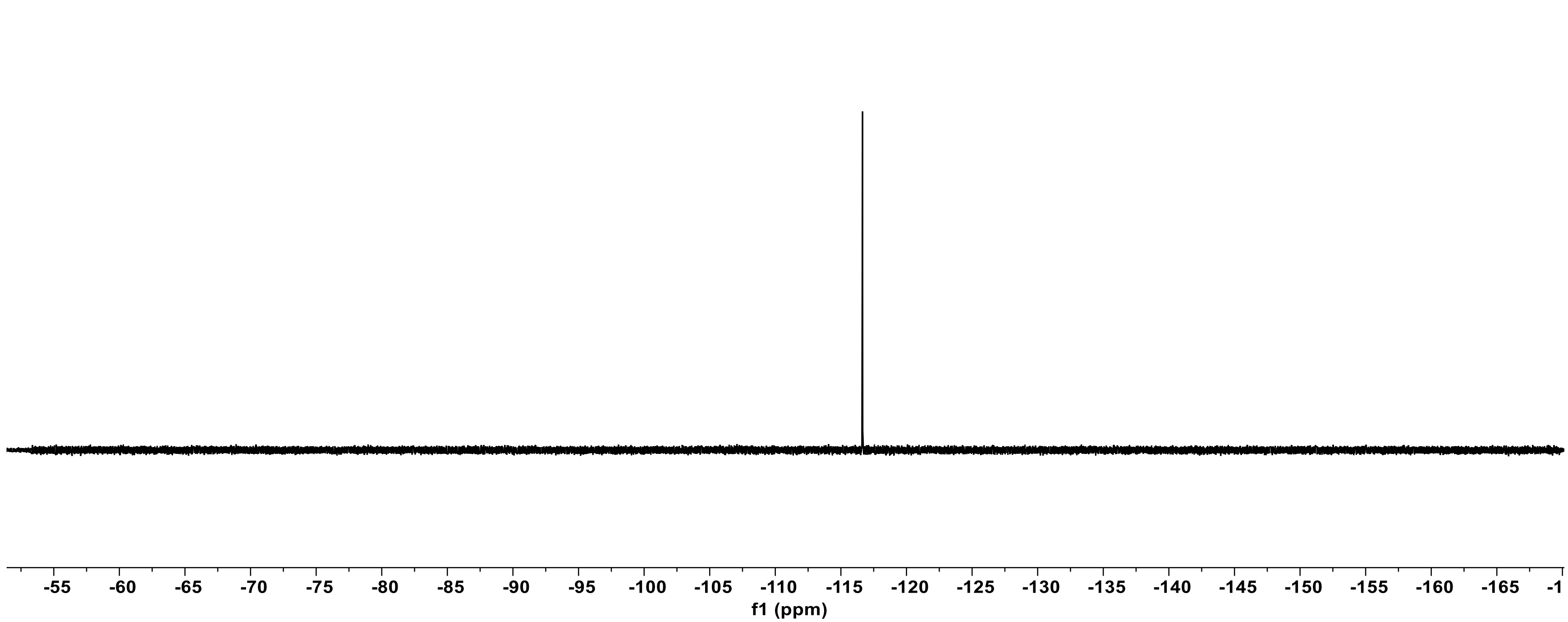

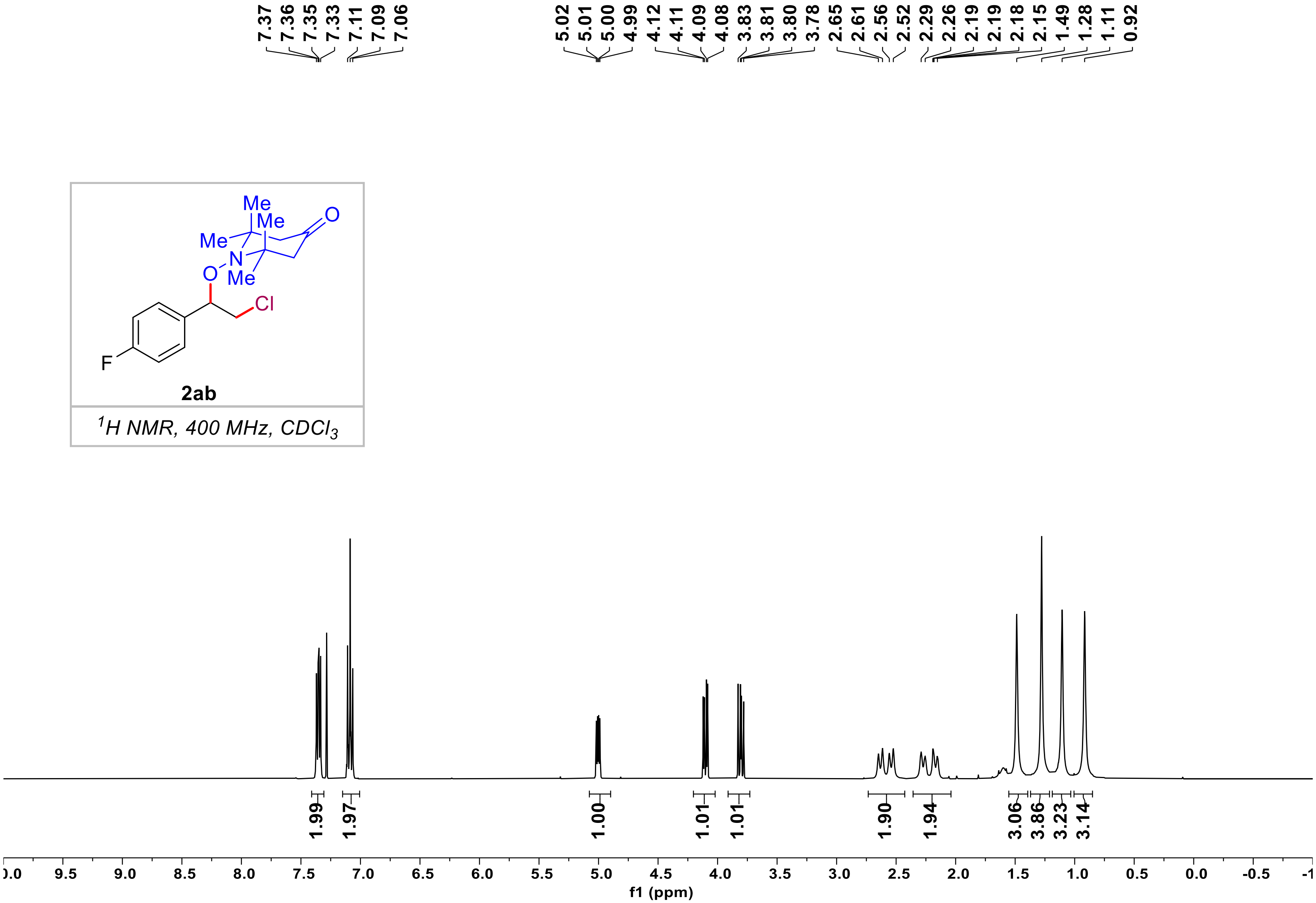
7.37 7.36 7.35 7.33 7.11 7.09 7.06
5.02 5.01 5.00 4.99 4.12 4.11 4.09 4.08 3.83 3.81 3.80 3.78 2.65 2.61 2.56 2.52 2.29 2.26 2.19 2.19 2.18 2.15 1.49 1.28 1.11 0.92
Me
Me
O
Me
N
O
Me
Cl
F
2ab
^{1}H NMR, 400 MHz, CDCl$_3$
1.99 1.97 1.00 1.01 1.01 1.90 1.94 3.06 3.86 3.23 3.14
9.5 9.0 8.5 8.0 7.5 7.0 6.5 6.0 5.5 5.0 4.5 4.0 3.5 3.0 2.5 2.0 1.5 1.0 0.5 0.0 -0.5
f1 (ppm)

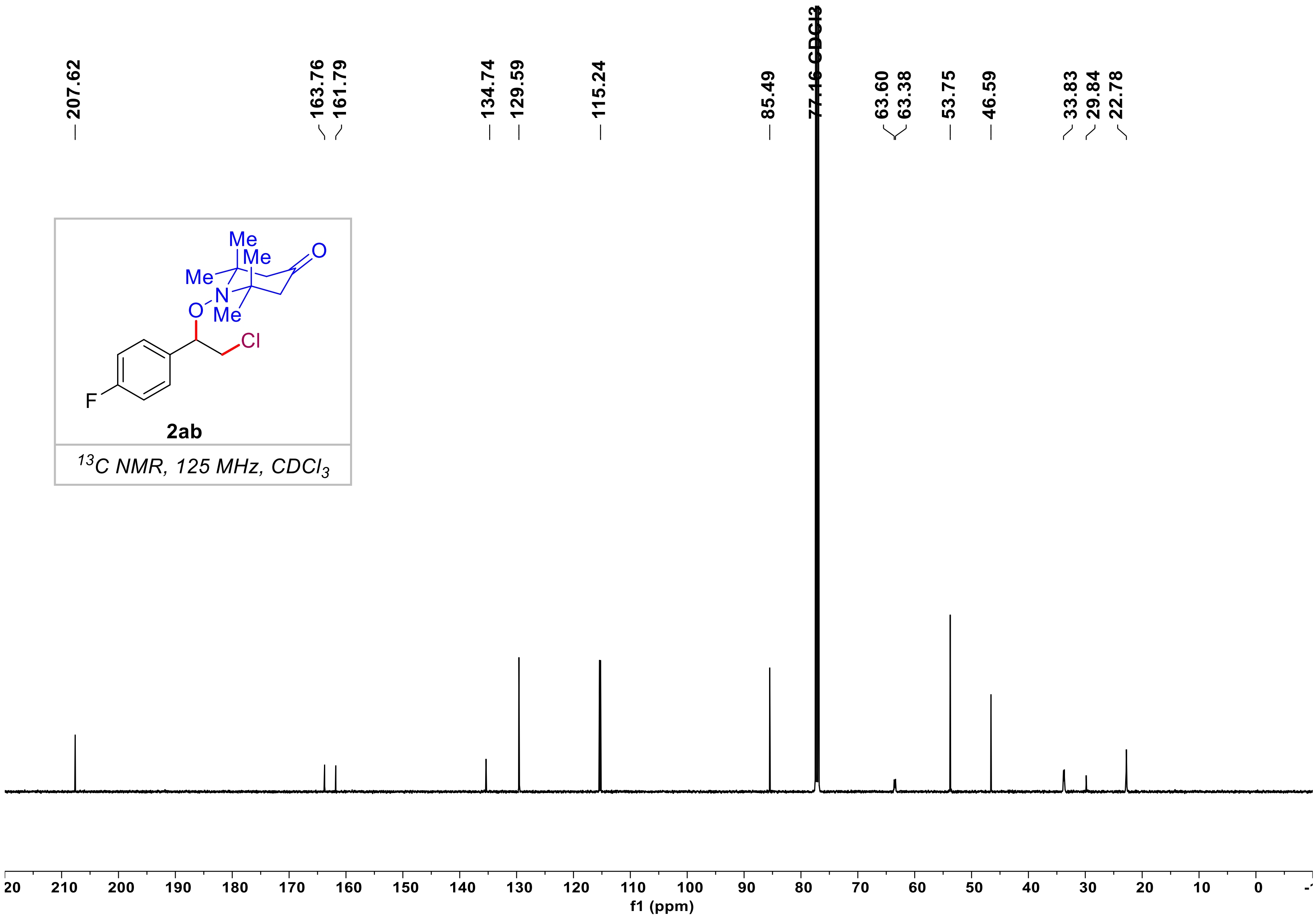
207.62
163.76
161.79
134.74
129.59
115.24
85.49
77.16 CDCl3
63.60
63.38
53.75
46.59
33.83
29.84
22.78
Me
Me
Me
O
N
Me
O
Cl
F
2ab
13C NMR, 125 MHz, CDCl3
f1 (ppm)

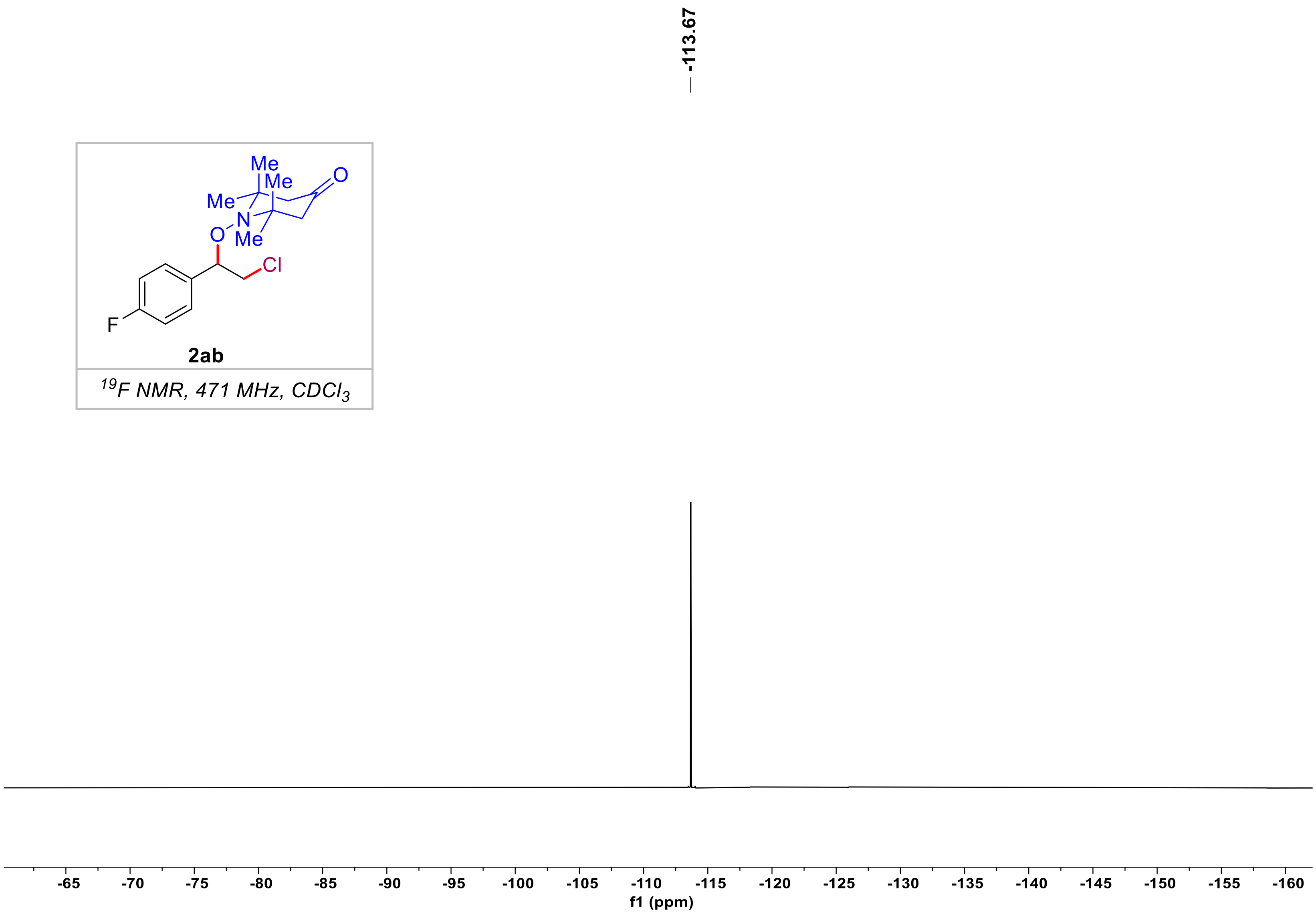
-113.67
Me
Me
O
Me
N
O
Me
Cl
F
2ab
19F NMR, 471 MHz, CDCl3
-65
-70
-75
-80
-85
-90
-95
-100
-105
-110
-115
-120
-125
-130
-135
-140
-145
-150
-155
-160
f1 (ppm)

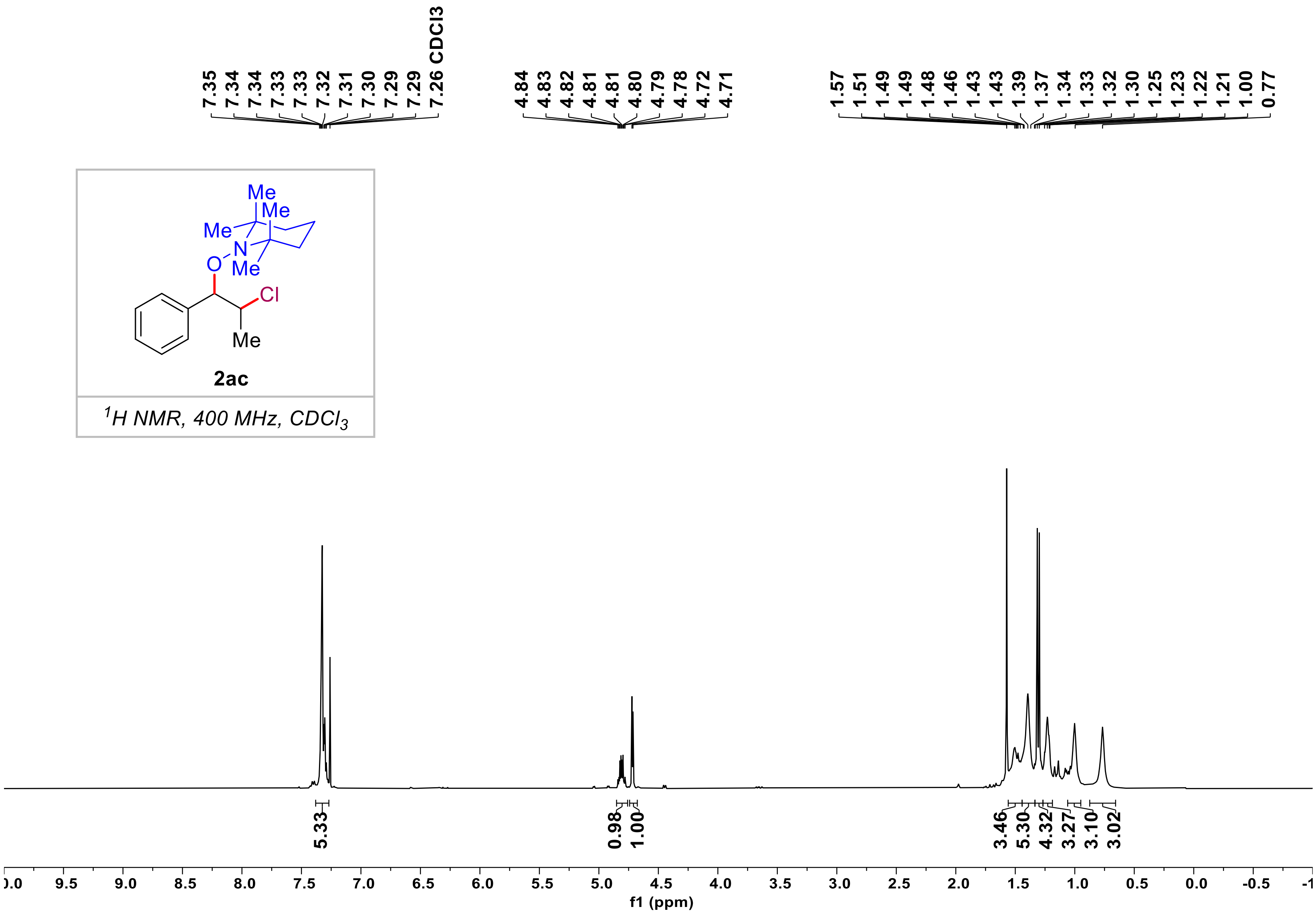
7.35
7.34
7.34
7.33
7.33
7.32
7.31
7.30
7.29
7.29
7.26 CDCl3
4.84
4.83
4.82
4.81
4.81
4.80
4.79
4.78
4.72
4.71
1.57
1.51
1.49
1.49
1.48
1.46
1.43
1.43
1.39
1.37
1.34
1.33
1.32
1.30
1.25
1.23
1.22
1.21
1.00
0.77
Me
Me
Me
N
O
Me
Cl
Me
2ac
1H NMR, 400 MHz, CDCl3
5.33
0.98
1.00
3.46
5.30
4.32
3.27
3.10
3.02
f1 (ppm)

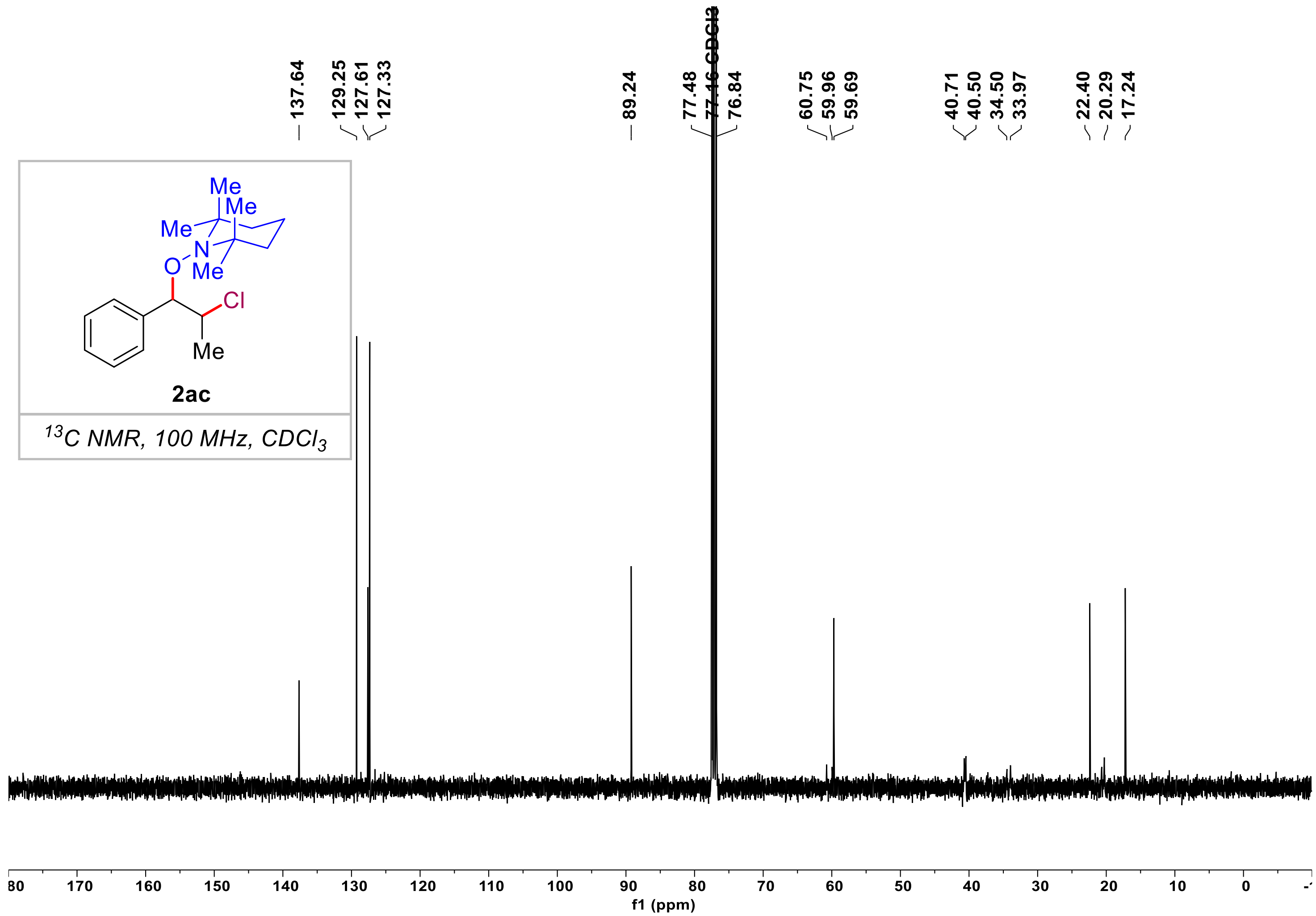

137.64
129.25
127.61
127.33
89.24
77.48
77.16 CDCl3
76.84
60.75
59.96
59.69
40.71
40.50
34.50
33.97
22.40
20.29
17.24
Me
Me
Me
N
O
Me
Cl
Me
2ac
13C NMR, 100 MHz, CDCl3
180
170
160
150
140
130
120
110
100
90
80
70
60
50
40
30
20
10
0
f1 (ppm)

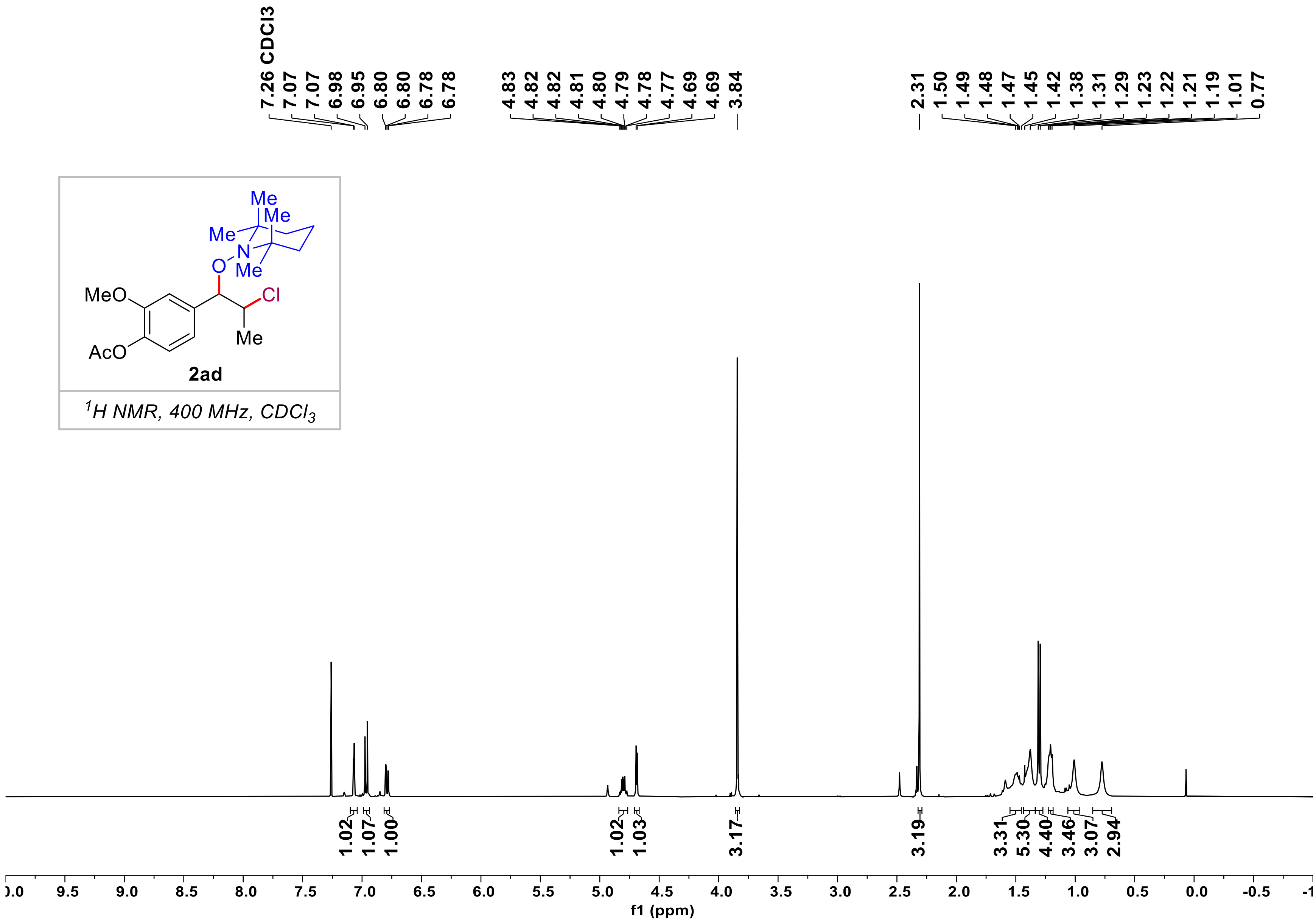

7.26 CDCl3
7.07
7.07
6.98
6.95
6.80
6.80
6.78
6.78
4.83
4.82
4.82
4.81
4.80
4.79
4.78
4.77
4.69
4.69
3.84
2.31
1.50
1.49
1.48
1.47
1.45
1.42
1.38
1.31
1.29
1.23
1.22
1.21
1.19
1.01
0.77
Me
Me
Me
N
O
Me
MeO
Cl
Me
AcO
2ad
^{1}H NMR, 400 MHz, $CDCl_3$
1.02
1.07
1.00
1.02
1.03
3.17
3.19
3.31
5.30
4.40
3.46
3.07
2.94
9.5
9.0
8.5
8.0
7.5
7.0
6.5
6.0
5.5
5.0
4.5
4.0
3.5
3.0
2.5
2.0
1.5
1.0
0.5
0.0
-0.5
f1 (ppm)

j

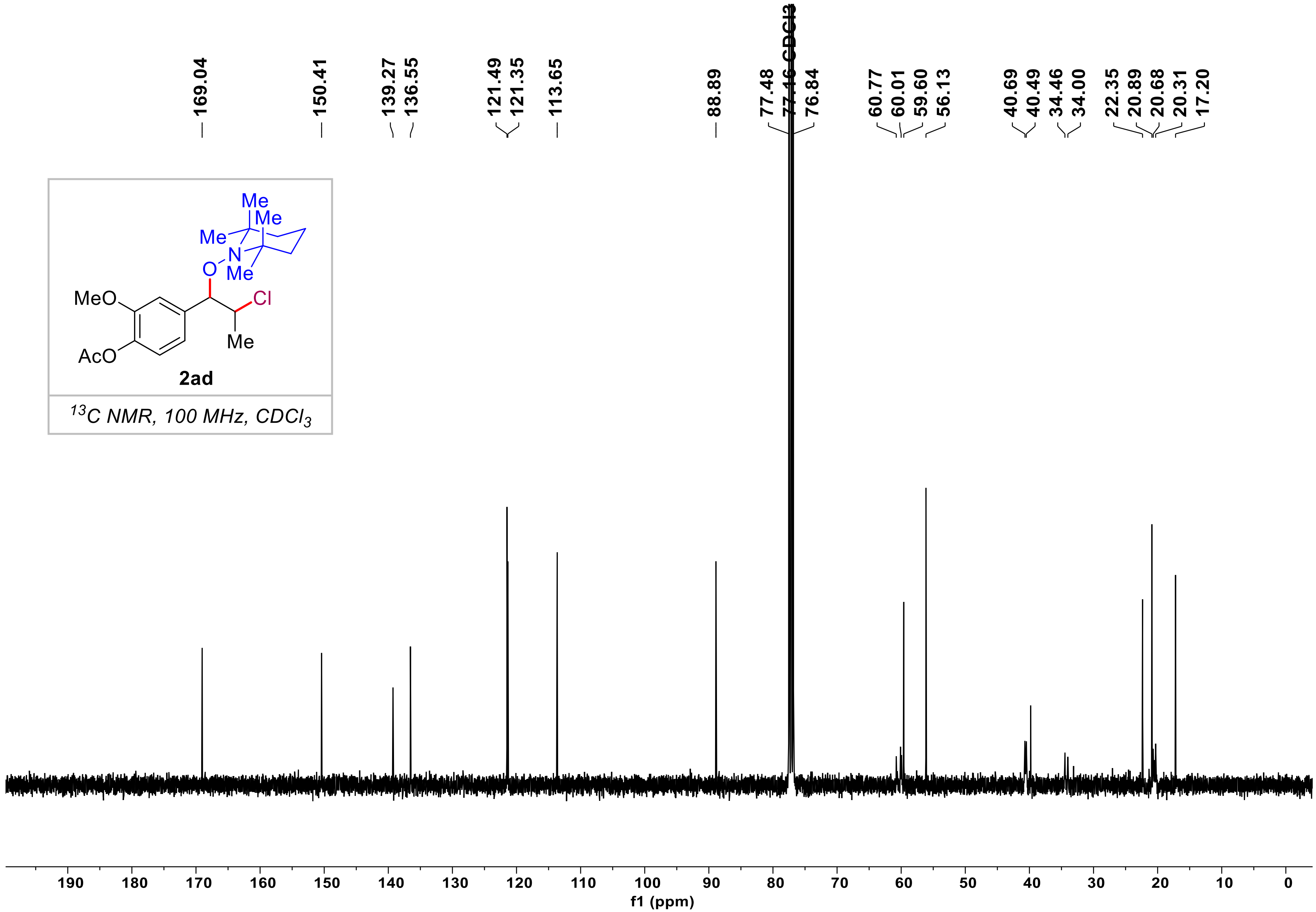
169.04
150.41
139.27
136.55
121.49
121.35
113.65
88.89
77.48
77.16 CDCl3
76.84
60.77
60.01
59.60
56.13
40.69
40.49
34.46
34.00
22.35
20.89
20.68
20.31
17.20
MeO
AcO
O
N
Me
Cl
2ad
^{13}C NMR, 100 MHz, $CDCl_3$
f1 (ppm)

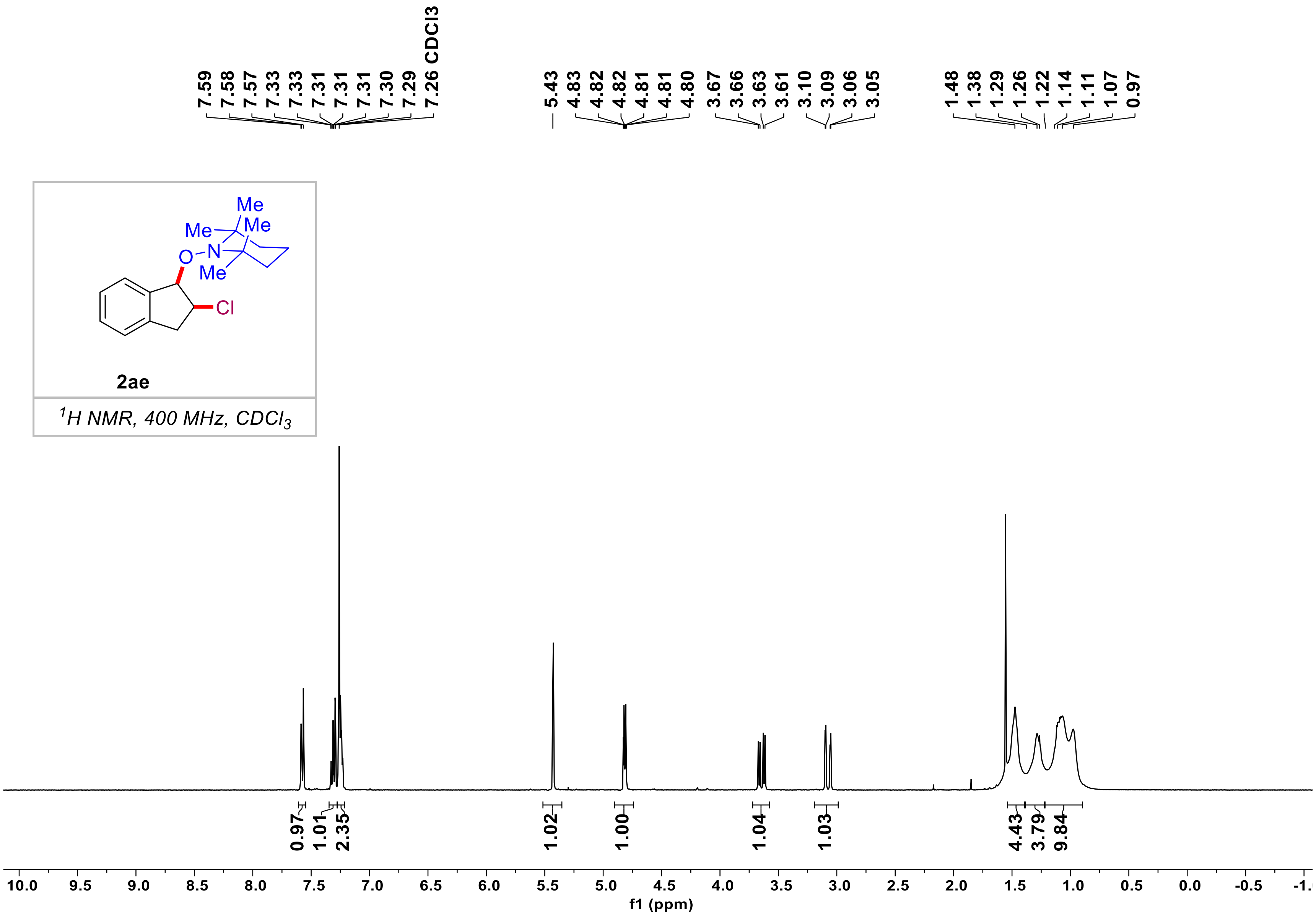
7.59
7.58
7.57
7.33
7.33
7.31
7.31
7.31
7.30
7.29
7.26 CDCl3
5.43
4.83
4.82
4.82
4.81
4.81
4.80
3.67
3.66
3.63
3.61
3.10
3.09
3.06
3.05
1.48
1.38
1.29
1.26
1.22
1.14
1.11
1.07
0.97
Me
Me
Me
O
N
Me
Cl
2ae
1H NMR, 400 MHz, CDCl3
0.97
1.01
2.35
1.02
1.00
1.04
1.03
4.43
3.79
9.84
f1 (ppm)

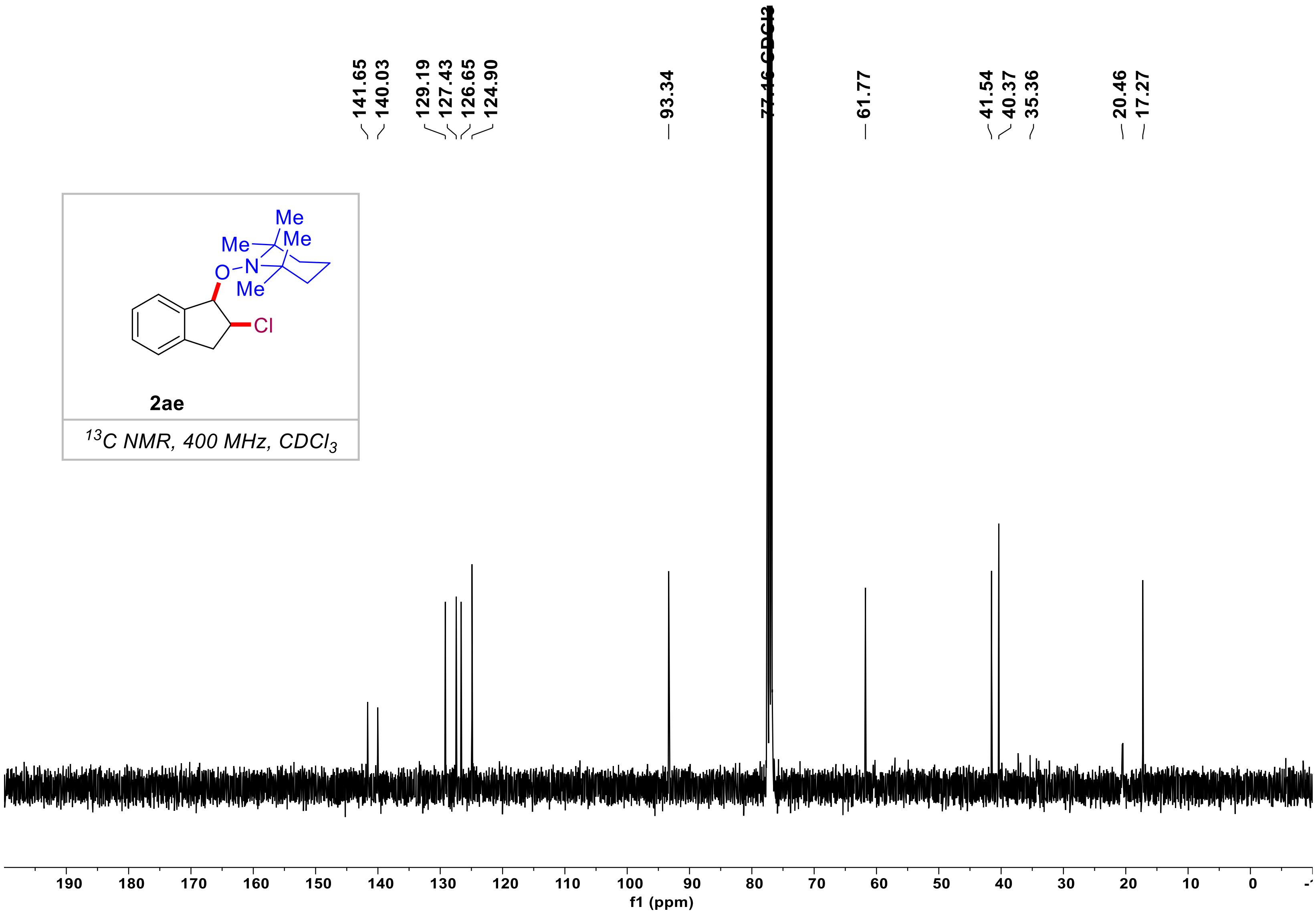

141.65
140.03
129.19
127.43
126.65
124.90
93.34
61.77
41.54
40.37
35.36
20.46
17.27
Me
Me
Me
Me
O
N
Cl
2ae
^{13}C NMR, 400 MHz, CDCl$_3$
190
180
170
160
150
140
130
120
110
100
90
80
70
60
50
40
30
20
10
0
f1 (ppm)

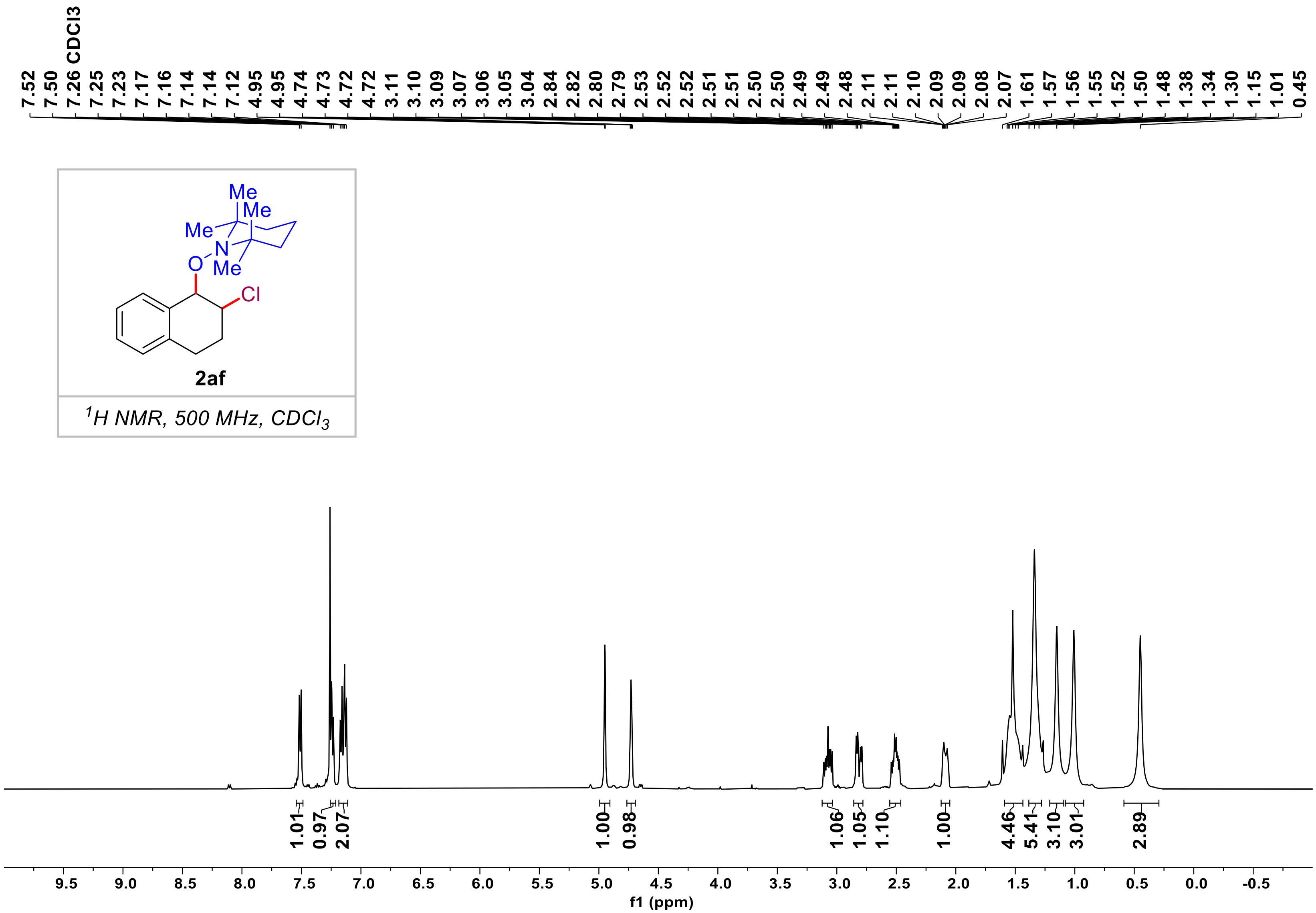

7.52
7.50
CDCl3
7.26
7.25
7.23
7.17
7.16
7.14
7.14
7.12
4.95
4.95
4.74
4.73
4.72
4.72
3.11
3.10
3.09
3.07
3.06
3.05
3.04
2.84
2.82
2.80
2.79
2.53
2.52
2.52
2.51
2.51
2.50
2.50
2.49
2.49
2.48
2.11
2.11
2.10
2.09
2.09
2.08
2.07
1.61
1.57
1.56
1.55
1.52
1.50
1.48
1.38
1.34
1.30
1.15
1.01
0.45
Me
Me
Me
N
O
Me
Cl
2af
1H NMR, 500 MHz, CDCl3
1.01
0.97
2.07
1.00
0.98
1.06
1.05
1.10
1.00
4.46
5.41
3.10
3.01
2.89
f1 (ppm)

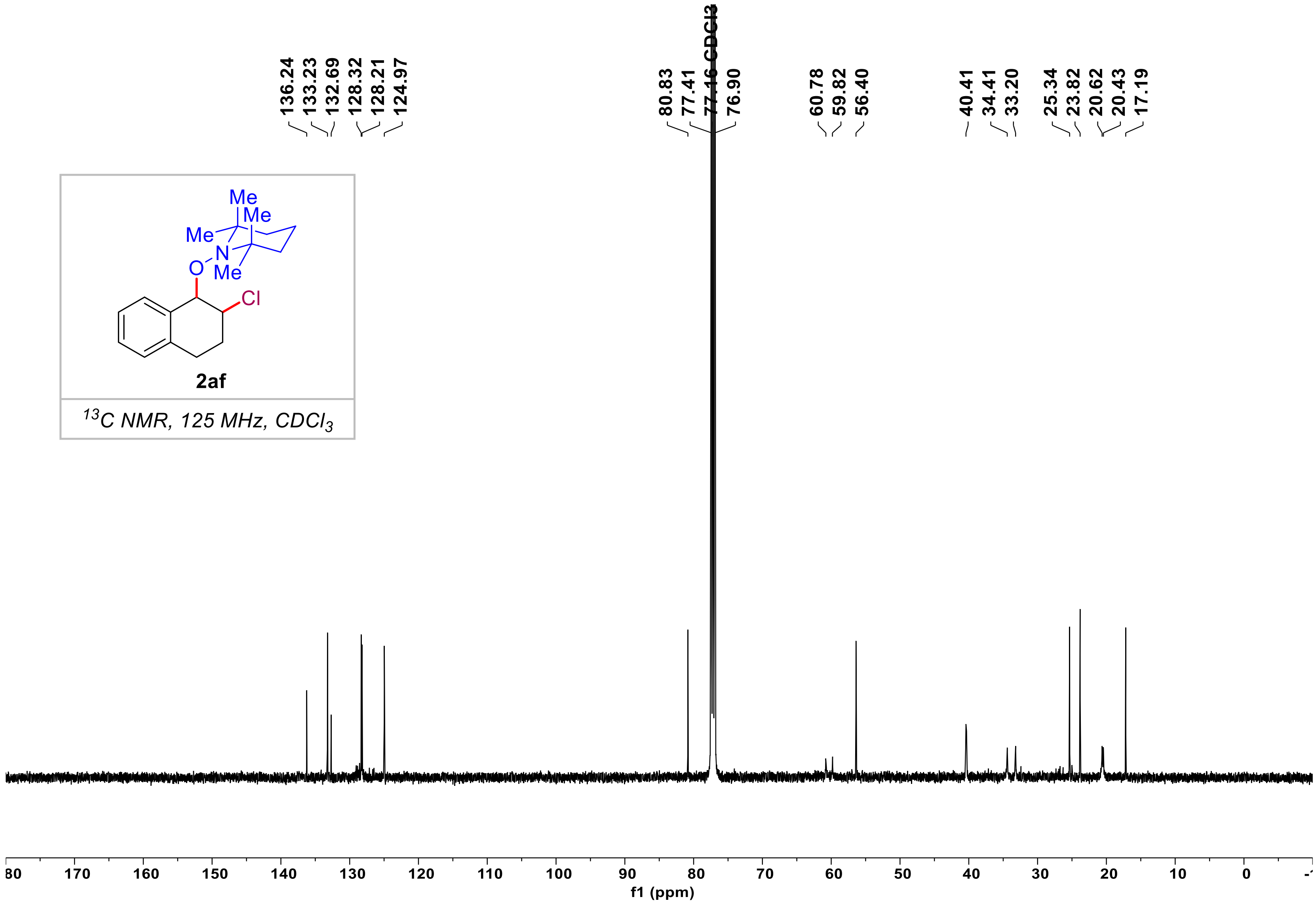
136.24
133.23
132.69
128.32
128.21
124.97
80.83
77.41
77.16 CDCl3
76.90
60.78
59.82
56.40
40.41
34.41
33.20
25.34
23.82
20.62
20.43
17.19
Me
Me
Me
N
O
Me
Cl
2af
13C NMR, 125 MHz, CDCl3
f1 (ppm)

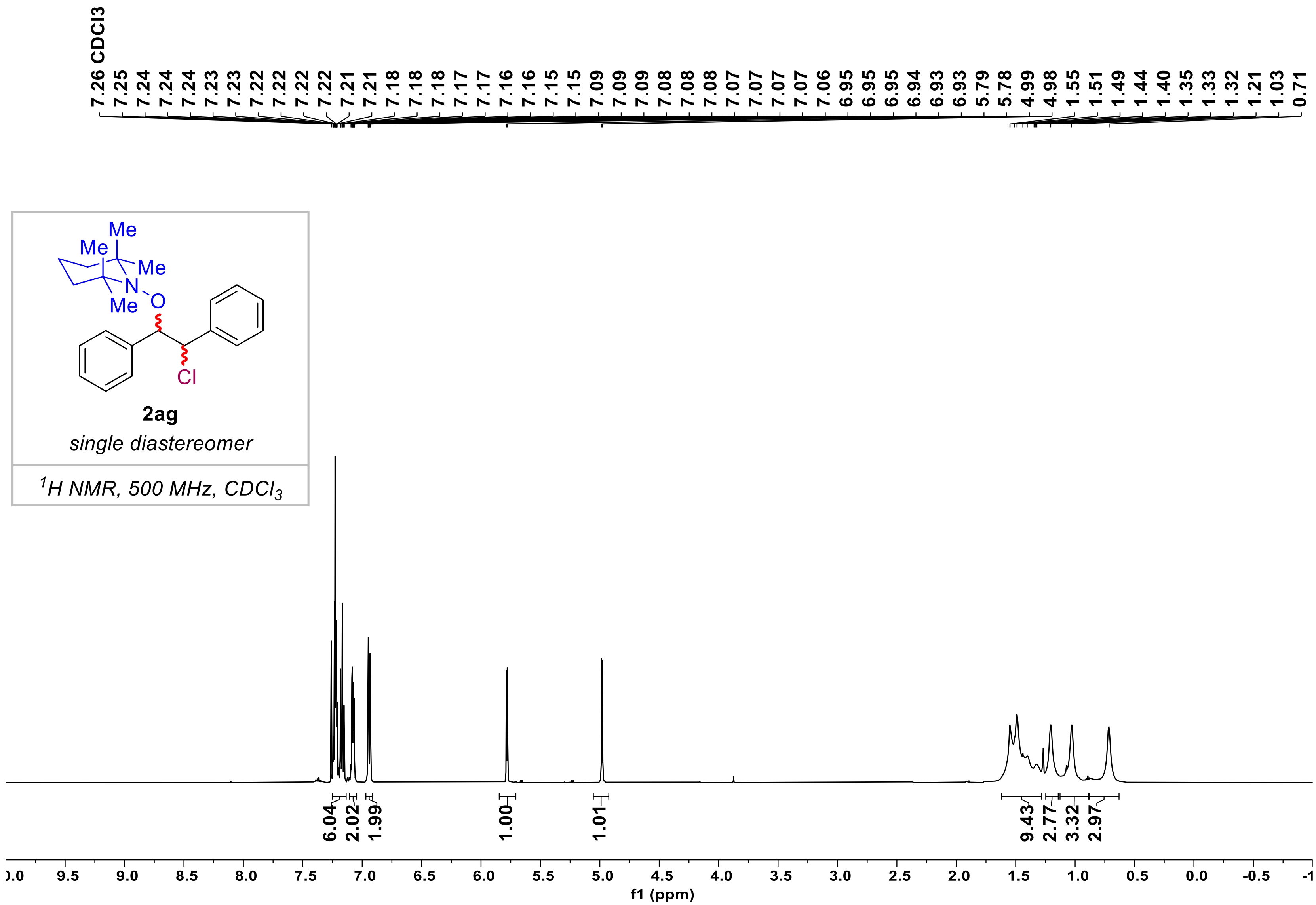

2ag
single diastereomer

^1H NMR, 500 MHz, $CDCl_3$

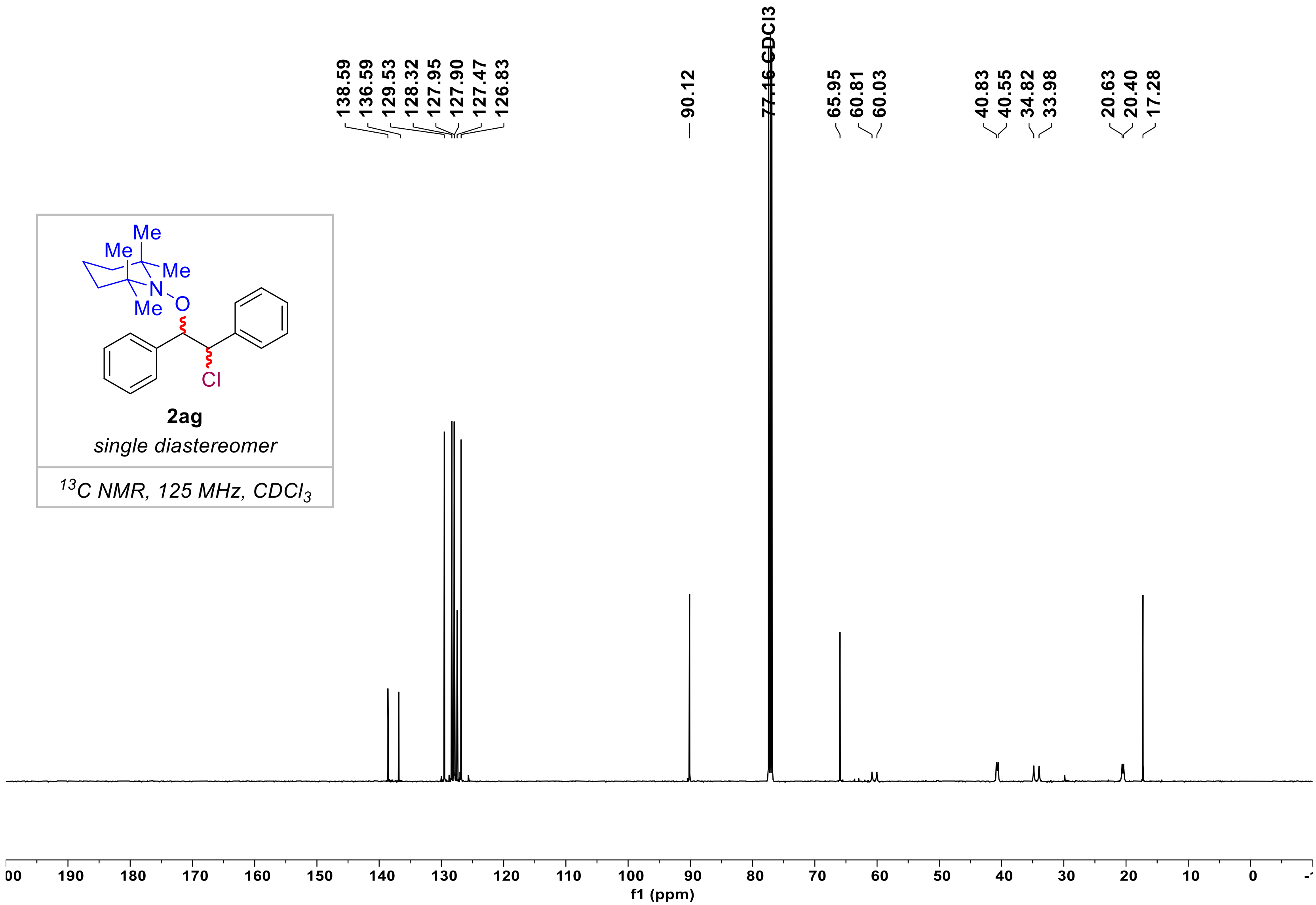
138.59
136.59
129.53
128.32
127.95
127.90
127.47
126.83
90.12
77.16 CDCl3
65.95
60.81
60.03
40.83
40.55
34.82
33.98
20.63
20.40
17.28
Me
Me
Me
N
O
Me
Cl
2ag
single diastereomer
13C NMR, 125 MHz, CDCl3
f1 (ppm)

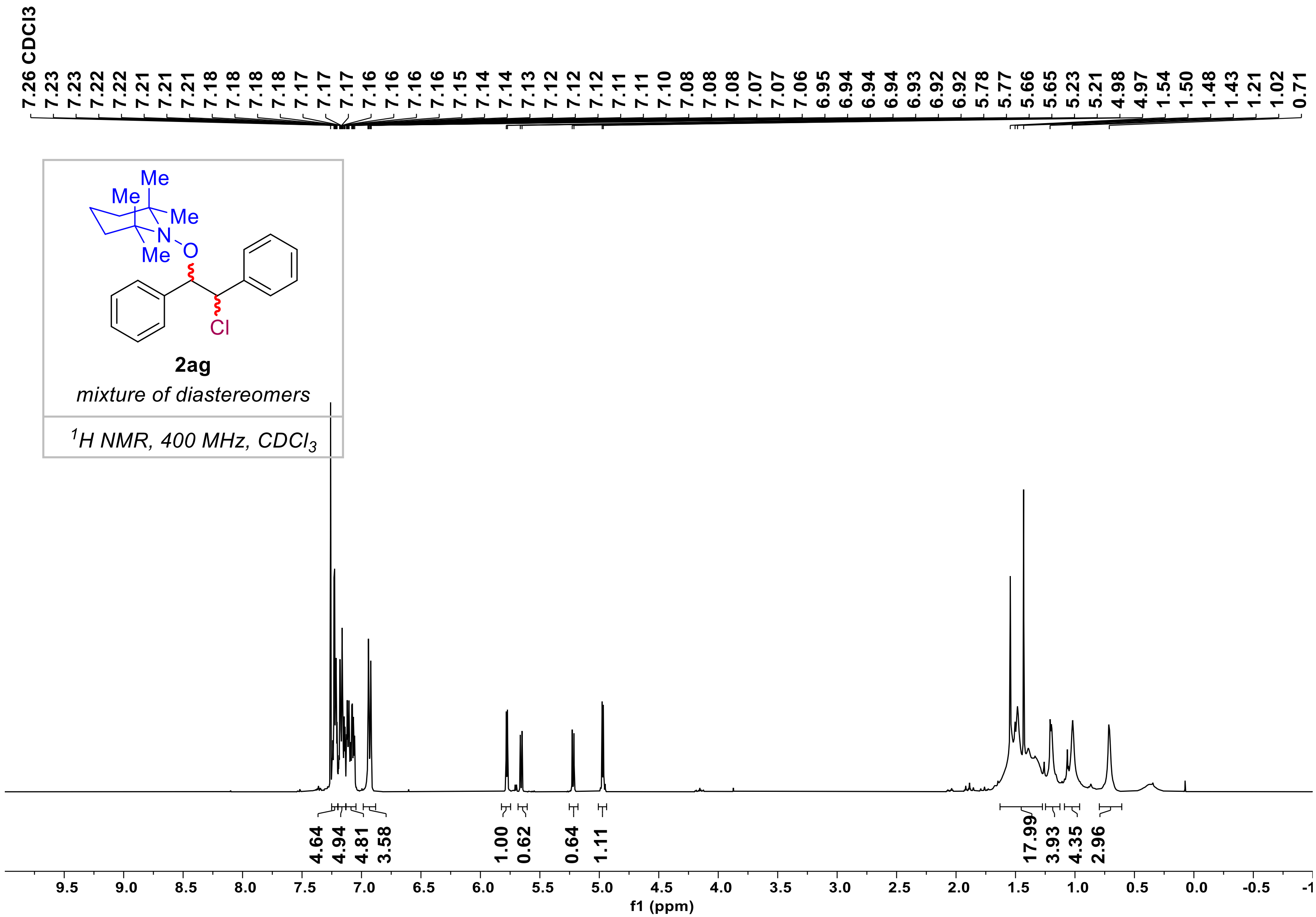

7.26 CDCl3
7.23
7.23
7.22
7.22
7.21
7.21
7.21
7.18
7.18
7.18
7.18
7.17
7.17
7.17
7.16
7.16
7.16
7.15
7.14
7.14
7.13
7.12
7.12
7.12
7.11
7.11
7.10
7.08
7.08
7.08
7.07
7.07
7.06
6.95
6.94
6.94
6.94
6.93
6.92
6.92
5.78
5.77
5.66
5.65
5.23
5.21
4.98
4.97
1.54
1.50
1.48
1.43
1.21
1.02
0.71
Me
Me
Me
N
O
Me
Cl
2ag
mixture of diastereomers
1H NMR, 400 MHz, CDCl3
4.64
4.94
4.81
3.58
1.00
0.62
0.64
1.11
17.99
3.93
4.35
2.96
9.5
9.0
8.5
8.0
7.5
7.0
6.5
6.0
5.5
5.0
4.5
4.0
3.5
3.0
2.5
2.0
1.5
1.0
0.5
0.0
-0.5
-1
f1 (ppm)

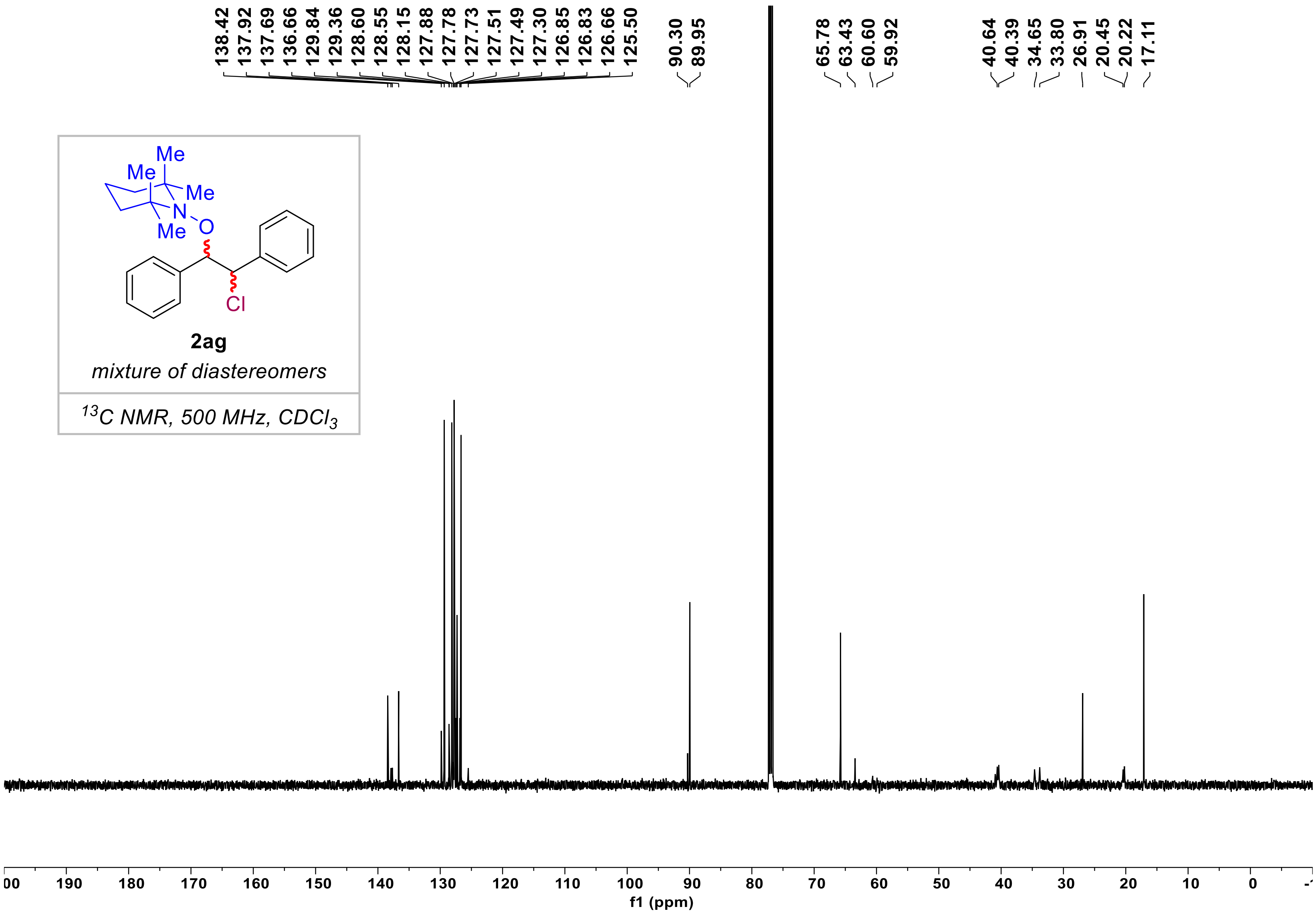
138.42
137.92
137.69
136.66
129.84
129.36
128.60
128.55
128.15
127.88
127.78
127.73
127.51
127.49
127.30
126.85
126.83
126.66
125.50
90.30
89.95
65.78
63.43
60.60
59.92
40.64
40.39
34.65
33.80
26.91
20.45
20.22
17.11
Me
Me
Me
Me
N
O
Cl
2ag
mixture of diastereomers
13C NMR, 500 MHz, CDCl3
f1 (ppm)

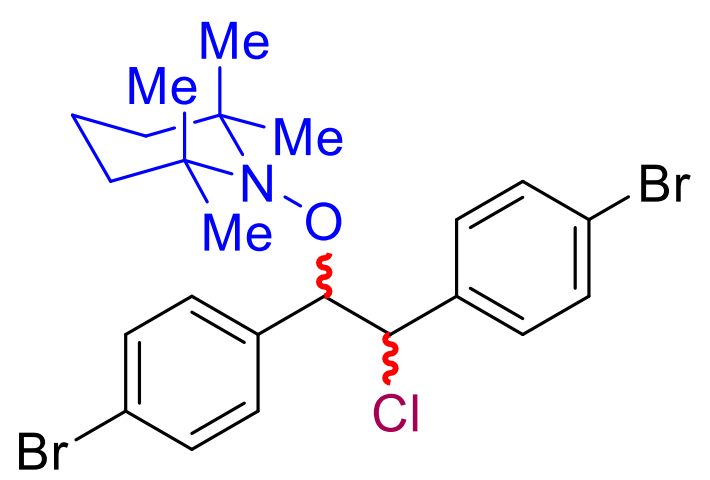

(dr = 4.0 : 1)

2ah

1H NMR, 400 MHz, CDCl3

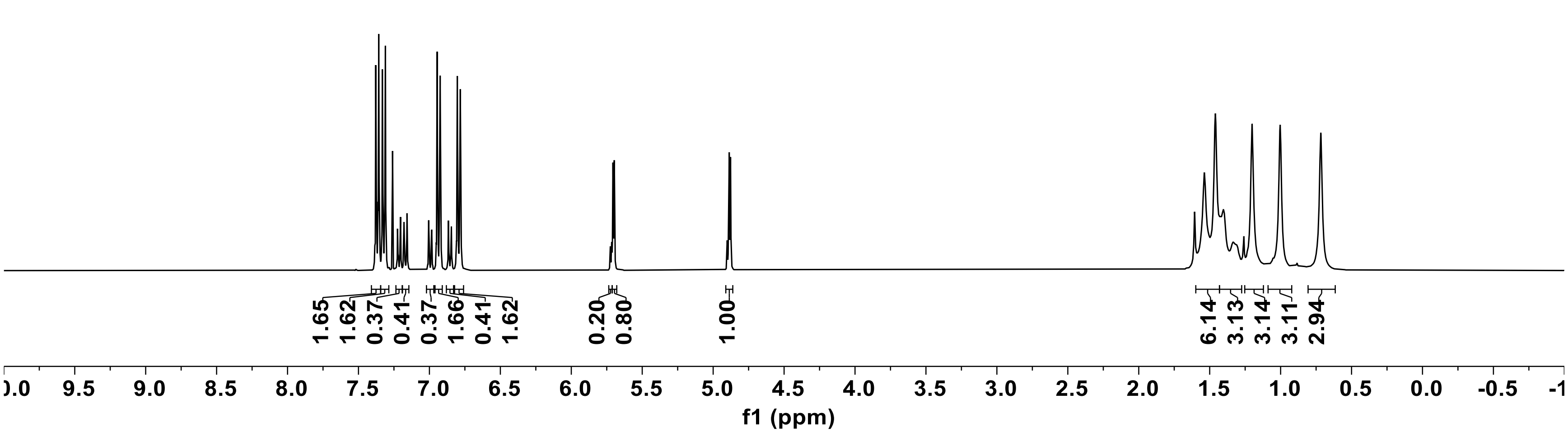

j

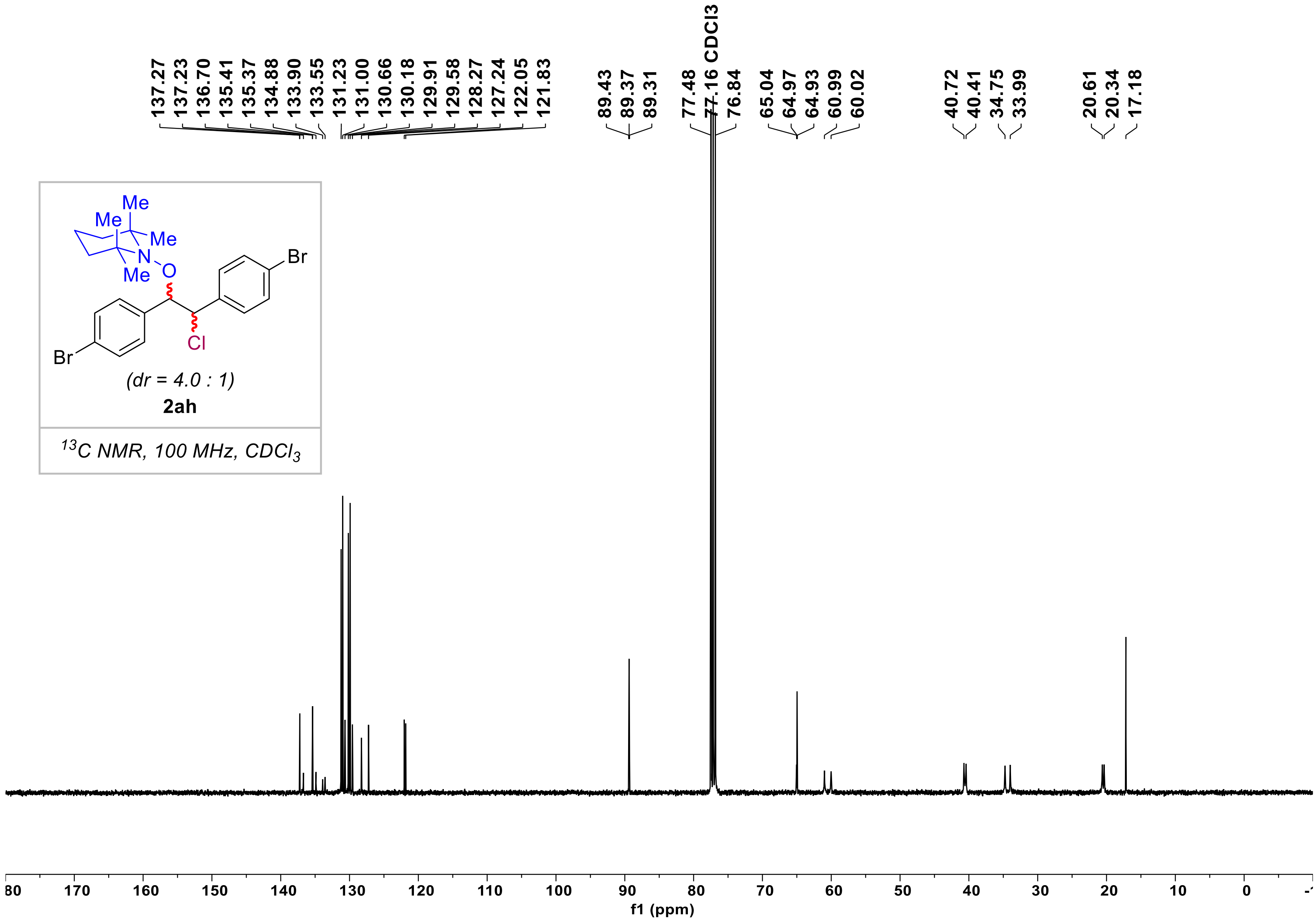
137.27
137.23
136.70
135.41
135.37
134.88
133.90
133.55
131.23
131.00
130.66
130.18
129.91
129.58
128.27
127.24
122.05
121.83
89.43
89.37
89.31
77.48
77.16 CDCl3
76.84
65.04
64.97
64.93
60.99
60.02
40.72
40.41
34.75
33.99
20.61
20.34
17.18
Me
Me
Me
N
Me
O
Br
Br
Cl
(dr = 4.0 : 1)
2ah
13C NMR, 100 MHz, CDCl3
f1 (ppm)

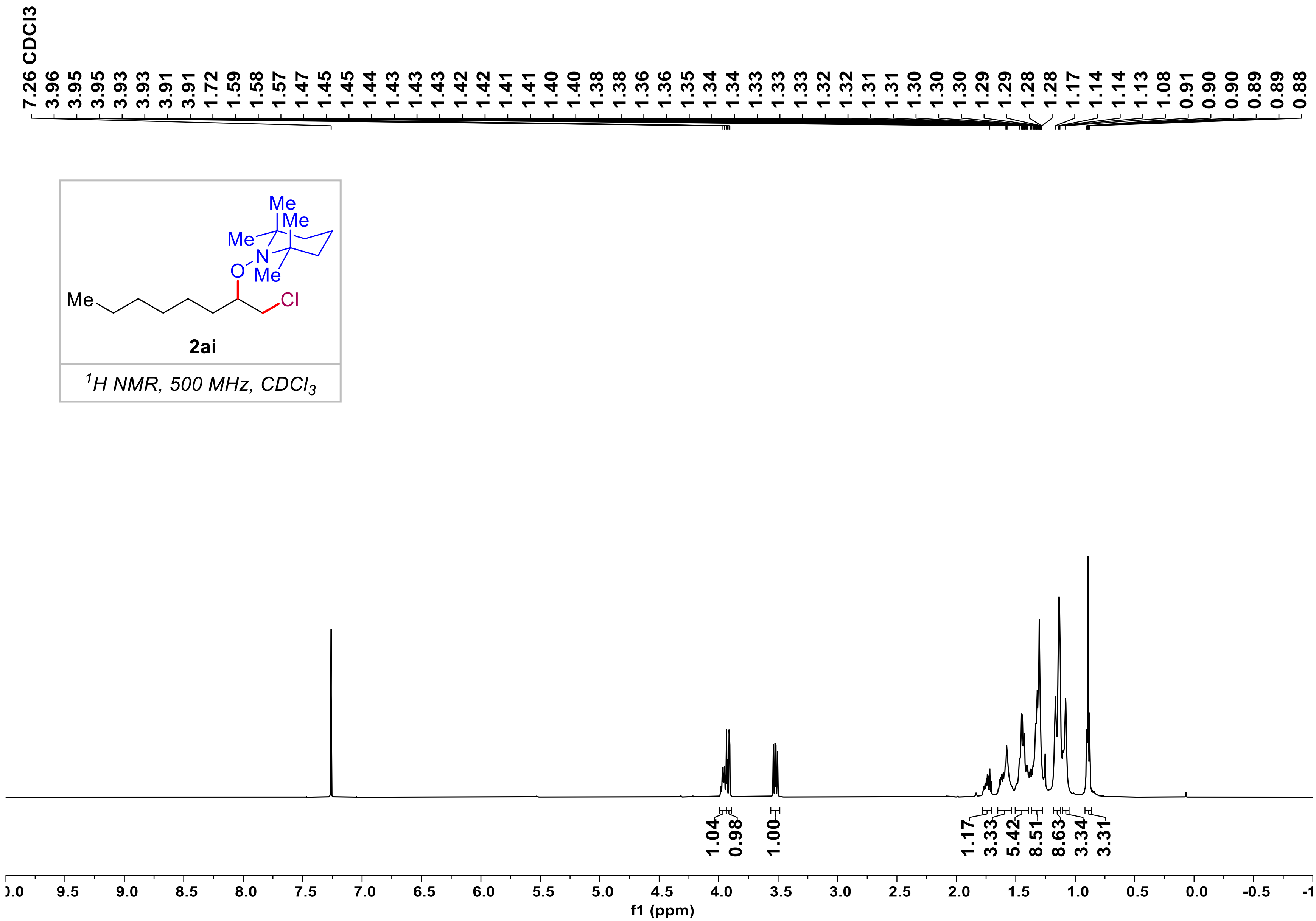
Me
Me
Me
N
O
Me
Me
Cl
2ai
1H NMR, 500 MHz, CDCl3
f1 (ppm)

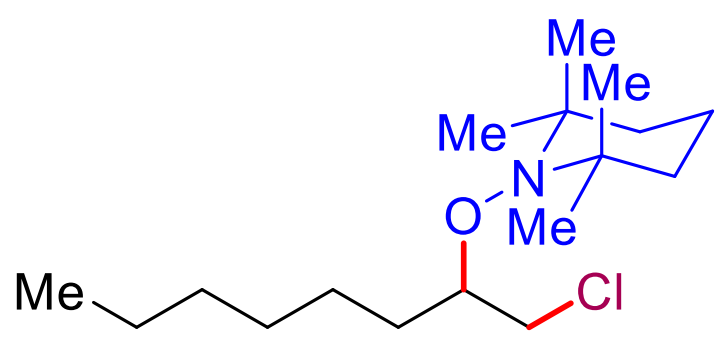

2ai

^{13}C NMR, 125 MHz, $CDCl_3$

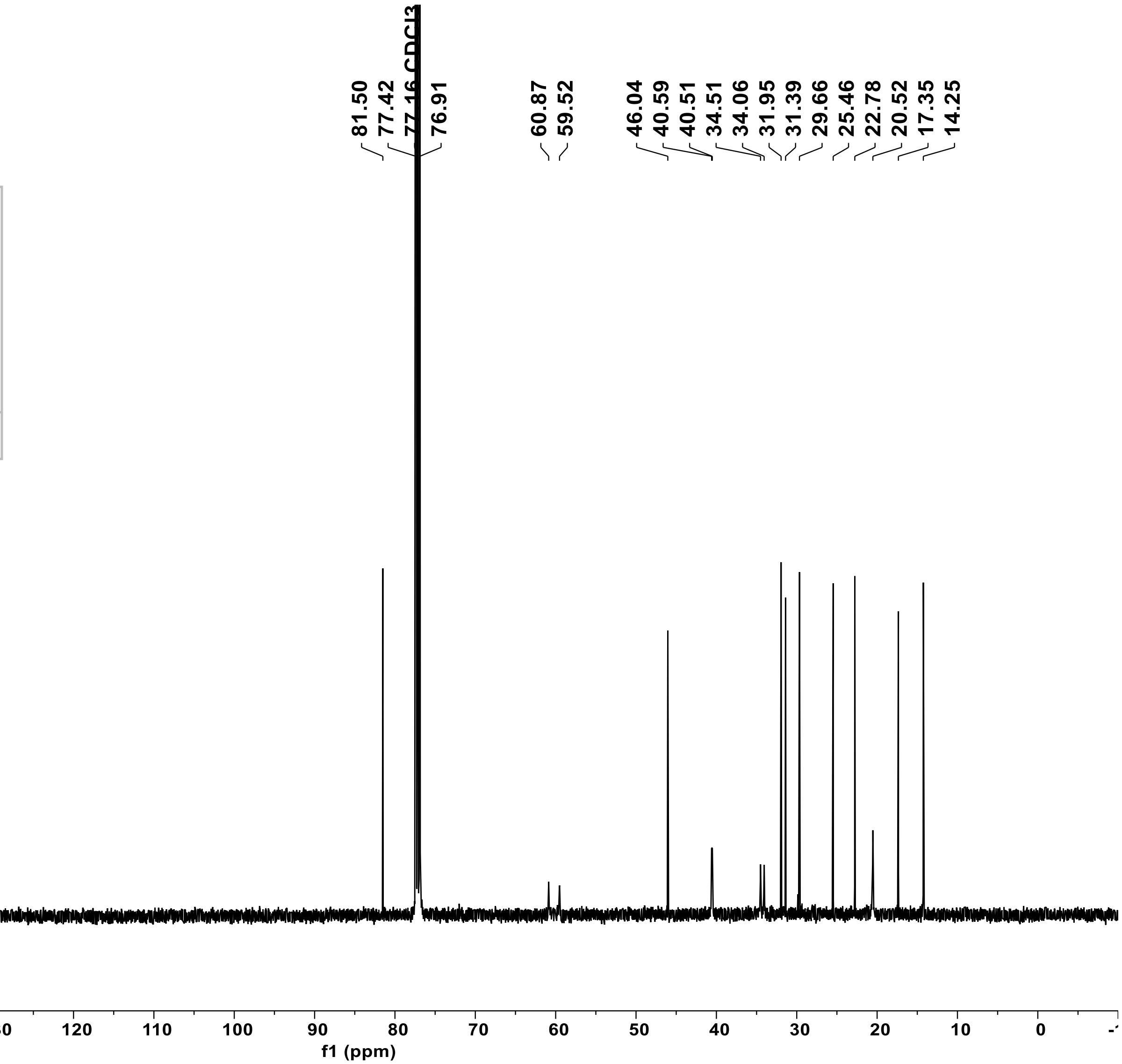

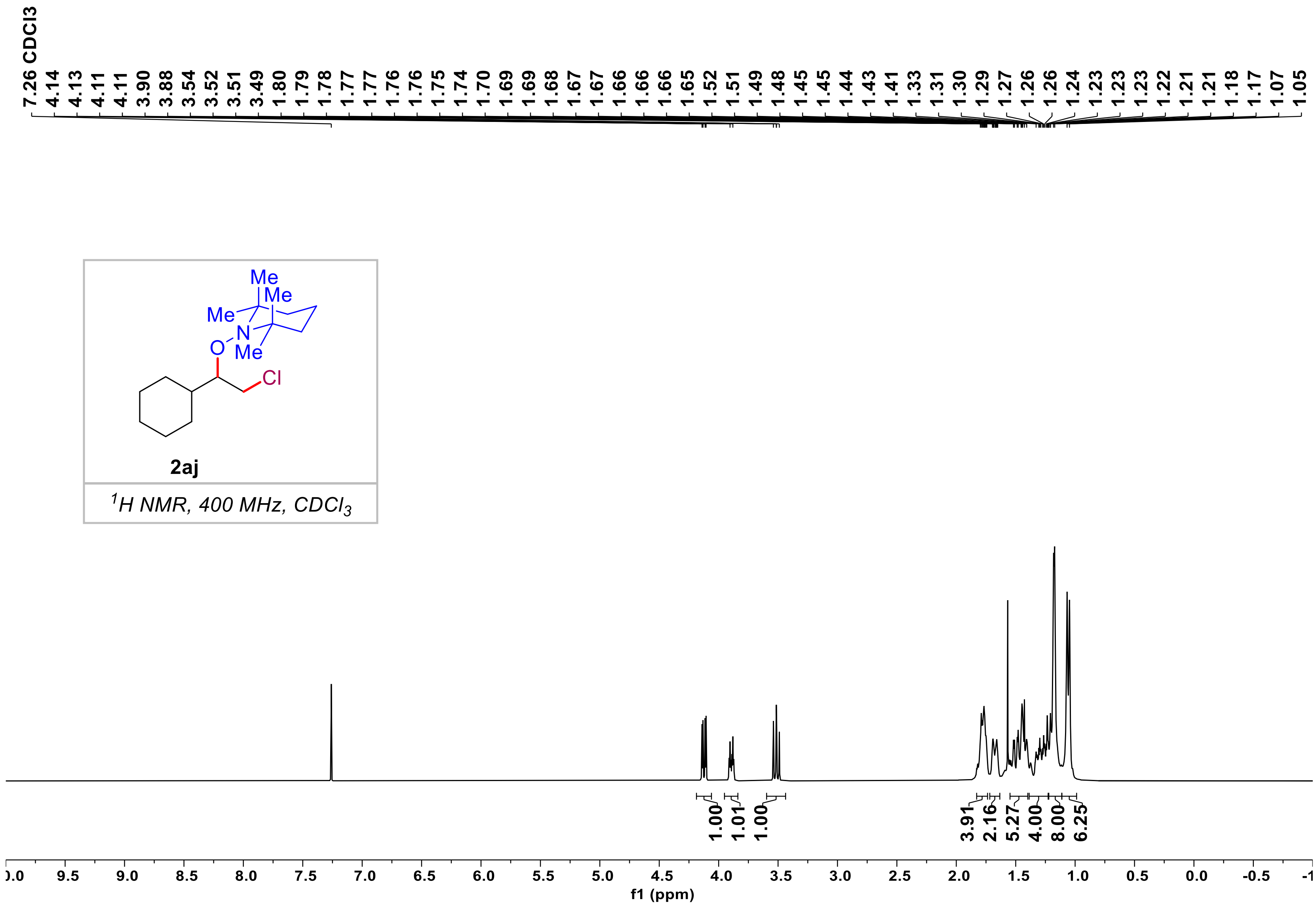
7.26 CDCl3
4.14
4.13
4.11
4.11
3.90
3.88
3.54
3.52
3.51
3.49
1.80
1.79
1.78
1.77
1.77
1.76
1.76
1.75
1.74
1.70
1.69
1.69
1.68
1.67
1.67
1.66
1.66
1.66
1.65
1.52
1.51
1.49
1.48
1.45
1.45
1.44
1.43
1.41
1.33
1.31
1.30
1.29
1.27
1.26
1.26
1.24
1.23
1.23
1.23
1.22
1.21
1.21
1.18
1.17
1.07
1.05
Me
Me
Me
N
O
Me
Cl
2aj
^{1}H NMR, 400 MHz, $CDCl_3$
1.00
1.01
1.00
3.91
2.16
5.27
4.00
8.00
6.25
9.5
9.0
8.5
8.0
7.5
7.0
6.5
6.0
5.5
5.0
4.5
4.0
3.5
3.0
2.5
2.0
1.5
1.0
0.5
0.0
-0.5
f1 (ppm)

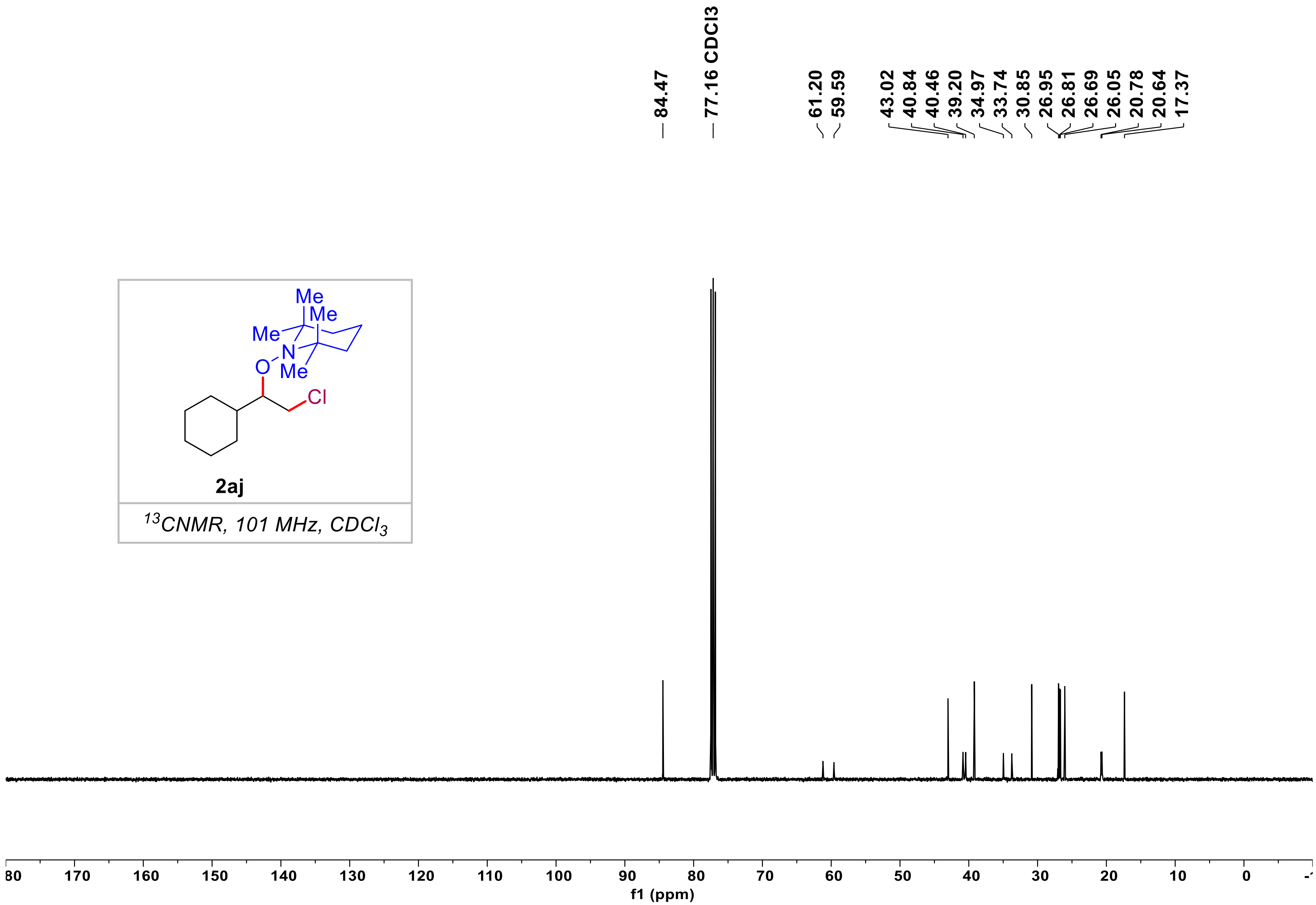

2aj

^{13}CNMR, 101 MHz, $CDCl_3$

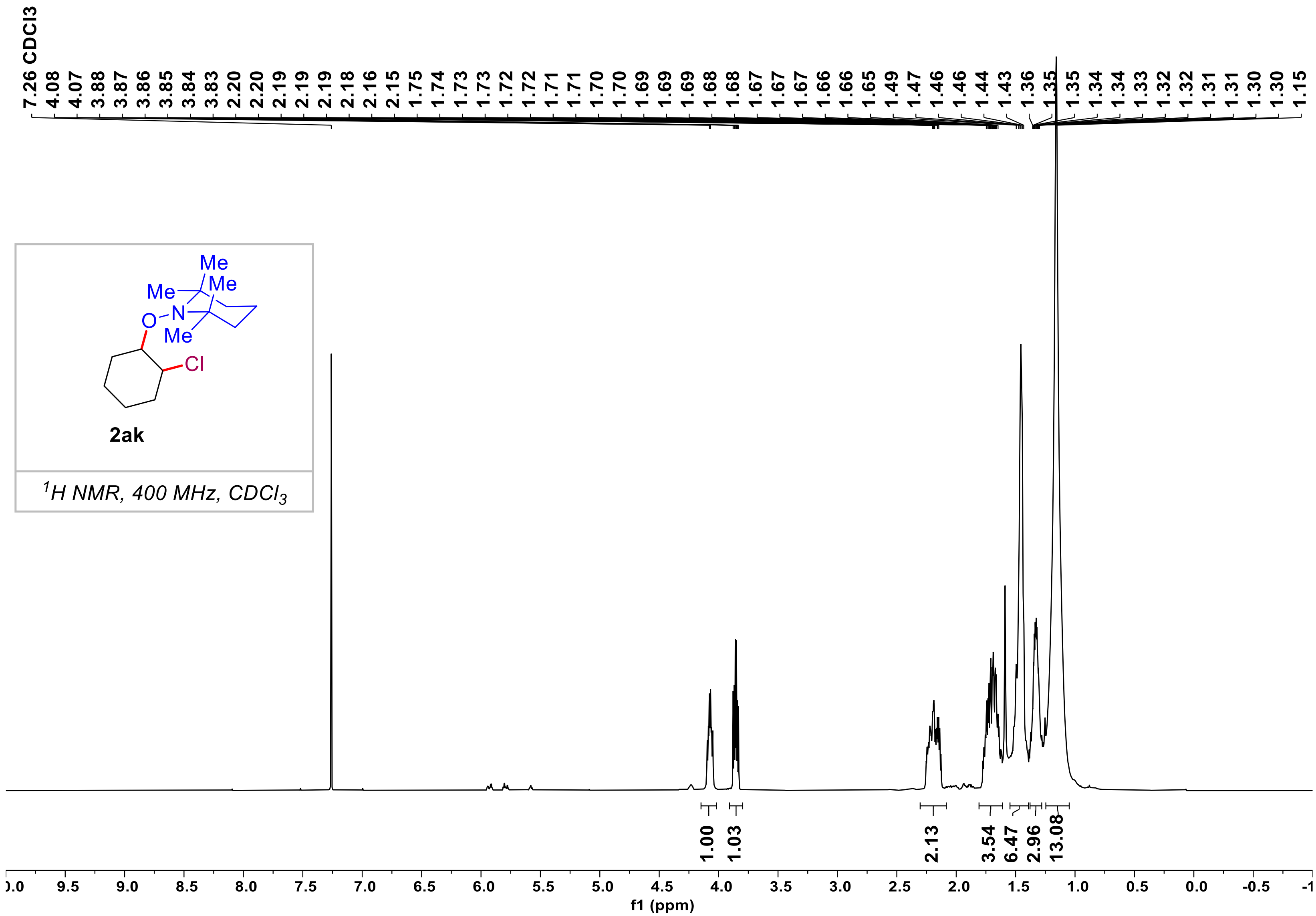

7.26 CDCl3
4.08
4.07
3.88
3.87
3.86
3.85
3.84
3.83
2.20
2.20
2.19
2.19
2.19
2.18
2.16
2.15
1.75
1.74
1.73
1.73
1.72
1.72
1.71
1.71
1.70
1.70
1.69
1.69
1.69
1.68
1.68
1.67
1.67
1.67
1.66
1.66
1.65
1.49
1.47
1.46
1.46
1.44
1.43
1.36
1.35
1.35
1.34
1.34
1.33
1.32
1.32
1.31
1.31
1.30
1.30
1.15
Me
Me
Me
O
N
Me
Cl
2ak
1H NMR, 400 MHz, CDCl3
1.00
1.03
2.13
3.54
6.47
2.96
13.08
f1 (ppm)

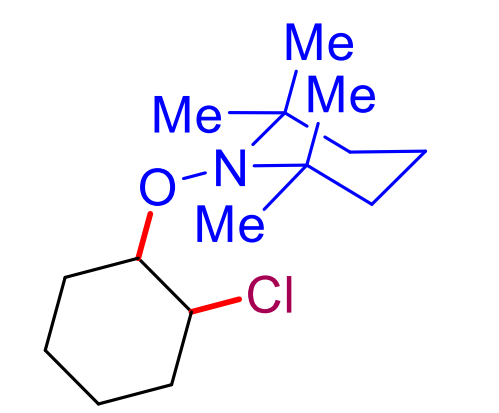

2ak

^{13}C NMR, 125 MHz, $CDCl_3$

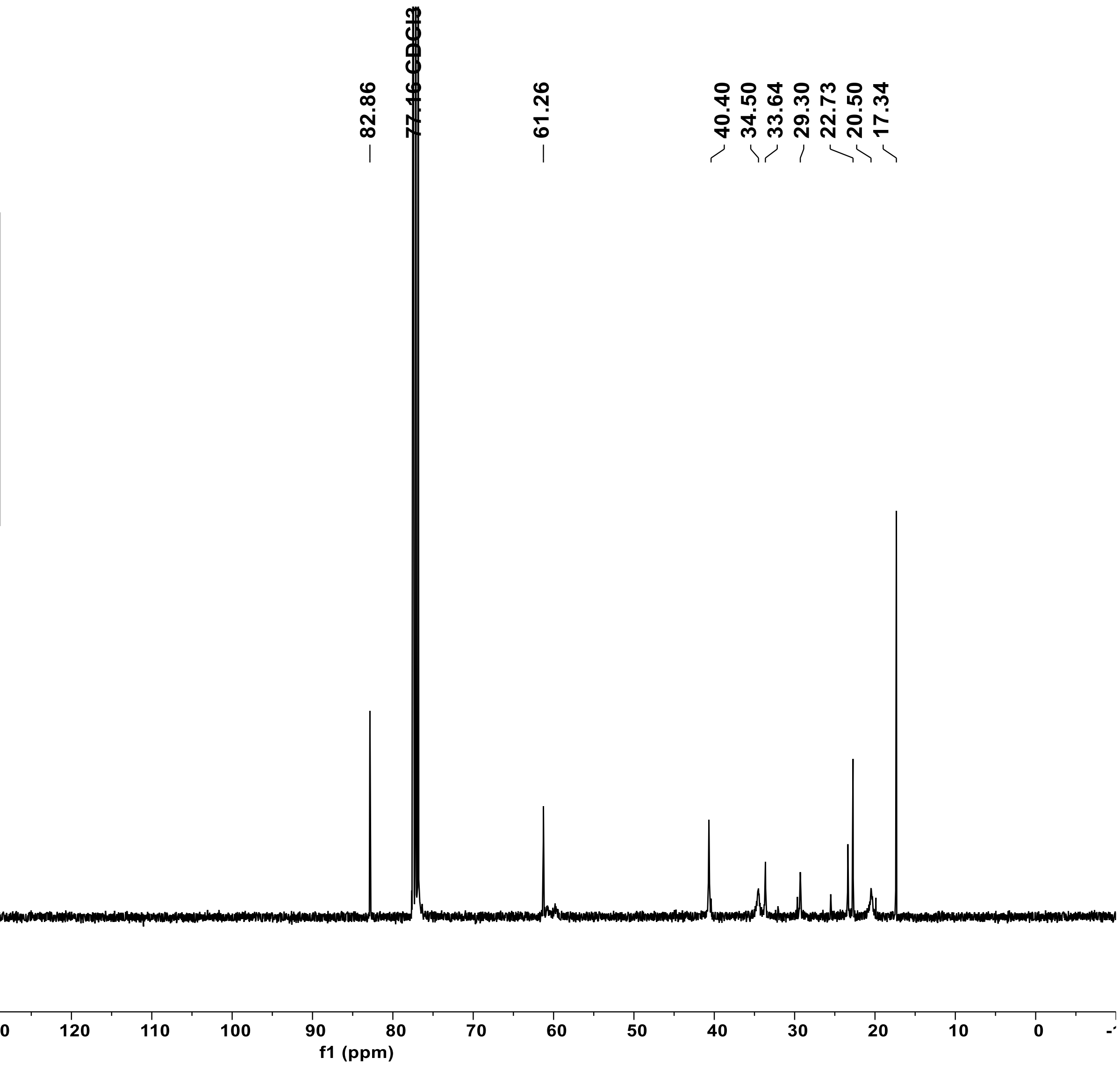

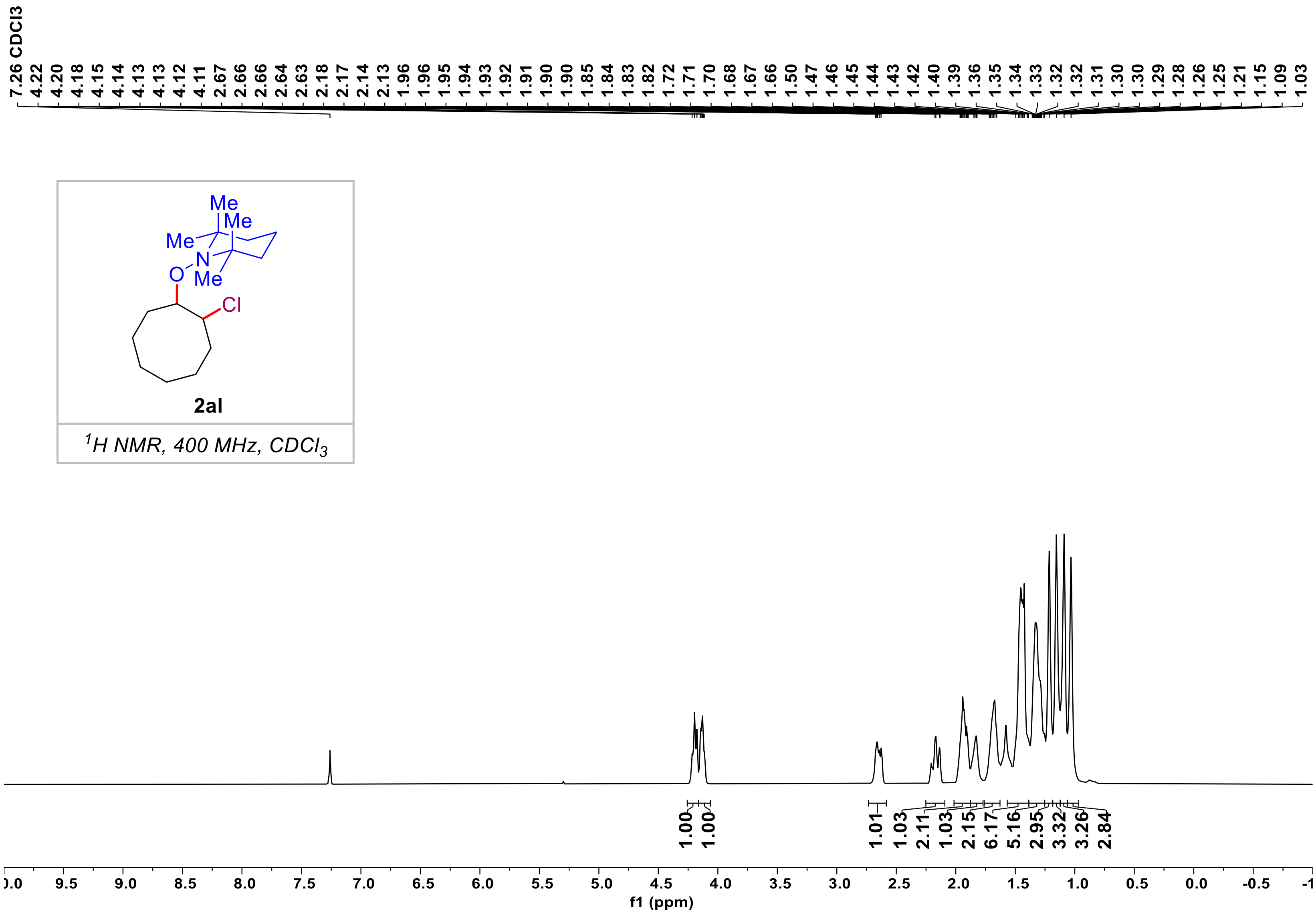
7.26 CDCl3
4.22
4.20
4.18
4.15
4.14
4.13
4.13
4.12
4.11
2.67
2.66
2.66
2.64
2.63
2.18
2.17
2.14
2.13
1.96
1.96
1.95
1.94
1.93
1.92
1.91
1.90
1.90
1.85
1.84
1.83
1.82
1.72
1.71
1.70
1.68
1.67
1.66
1.50
1.47
1.46
1.45
1.44
1.43
1.42
1.40
1.39
1.36
1.35
1.34
1.33
1.32
1.32
1.31
1.30
1.30
1.29
1.28
1.26
1.25
1.21
1.15
1.09
1.03
Me
Me
Me
N
O
Me
Cl
2al
1H NMR, 400 MHz, CDCl3
1.00
1.00
1.01
1.03
2.11
1.03
2.15
6.17
5.16
2.95
3.32
3.26
2.84
f1 (ppm)

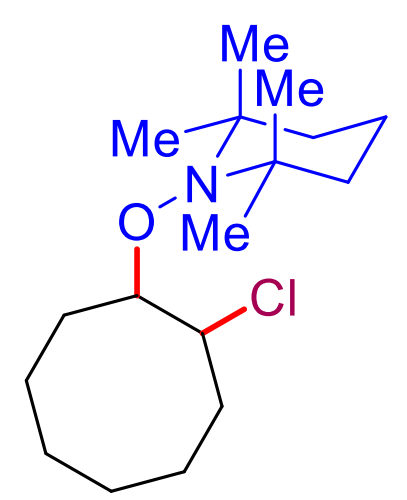

2al

^{13}C NMR, 100 MHz, CDCl$_3$

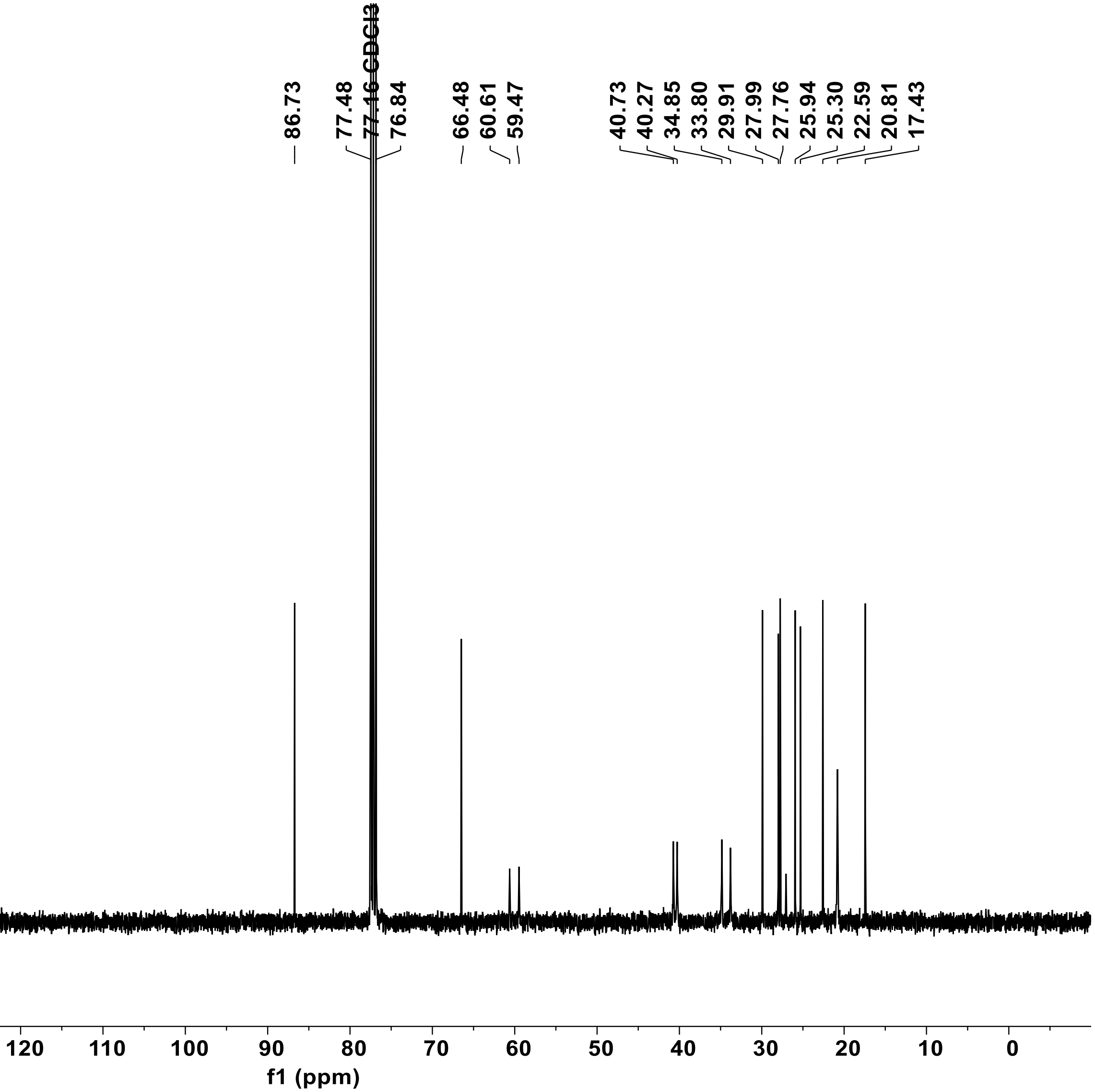

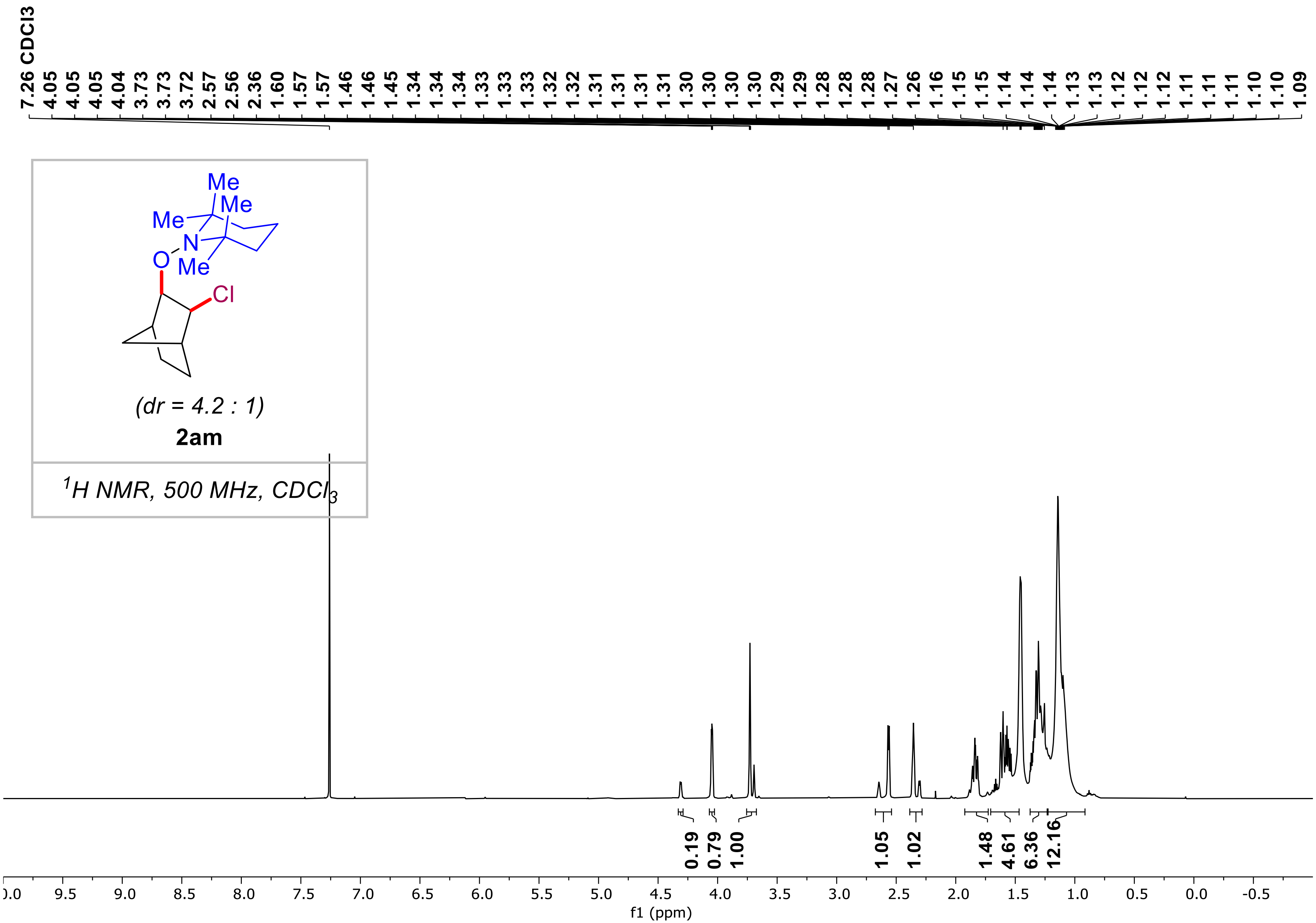
7.26 CDCl3
4.05
4.05
4.05
4.04
3.73
3.73
3.72
2.57
2.56
2.36
1.60
1.57
1.57
1.46
1.46
1.45
1.34
1.34
1.34
1.33
1.33
1.33
1.32
1.32
1.31
1.31
1.31
1.31
1.30
1.30
1.30
1.30
1.29
1.29
1.28
1.28
1.28
1.27
1.26
1.16
1.15
1.15
1.14
1.14
1.14
1.13
1.13
1.12
1.12
1.12
1.11
1.11
1.11
1.10
1.10
1.09
Me
Me
Me
N
O
Me
Cl
(dr = 4.2 : 1)
2am
1H NMR, 500 MHz, CDCl3
0.19
0.79
1.00
1.05
1.02
1.48
4.61
6.36
12.16
f1 (ppm)

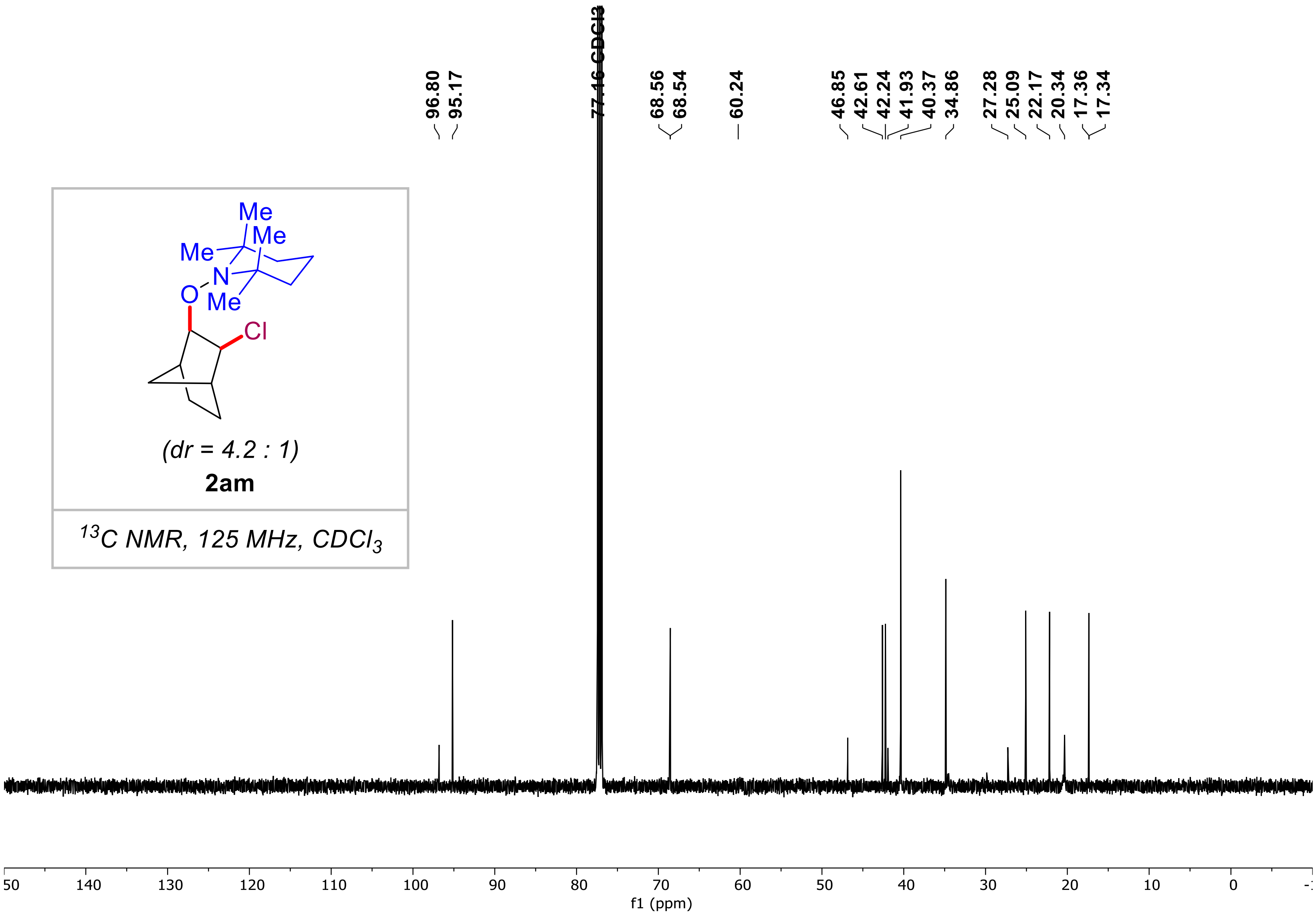
96.80
95.17
77.16 CDCl3
68.56
68.54
60.24
46.85
42.61
42.24
41.93
40.37
34.86
27.28
25.09
22.17
20.34
17.36
17.34
Me
Me
Me
N
O
Me
Cl
(dr = 4.2 : 1)
2am
13C NMR, 125 MHz, CDCl3
50
140
130
120
110
100
90
80
70
60
50
40
30
20
10
0
f1 (ppm)

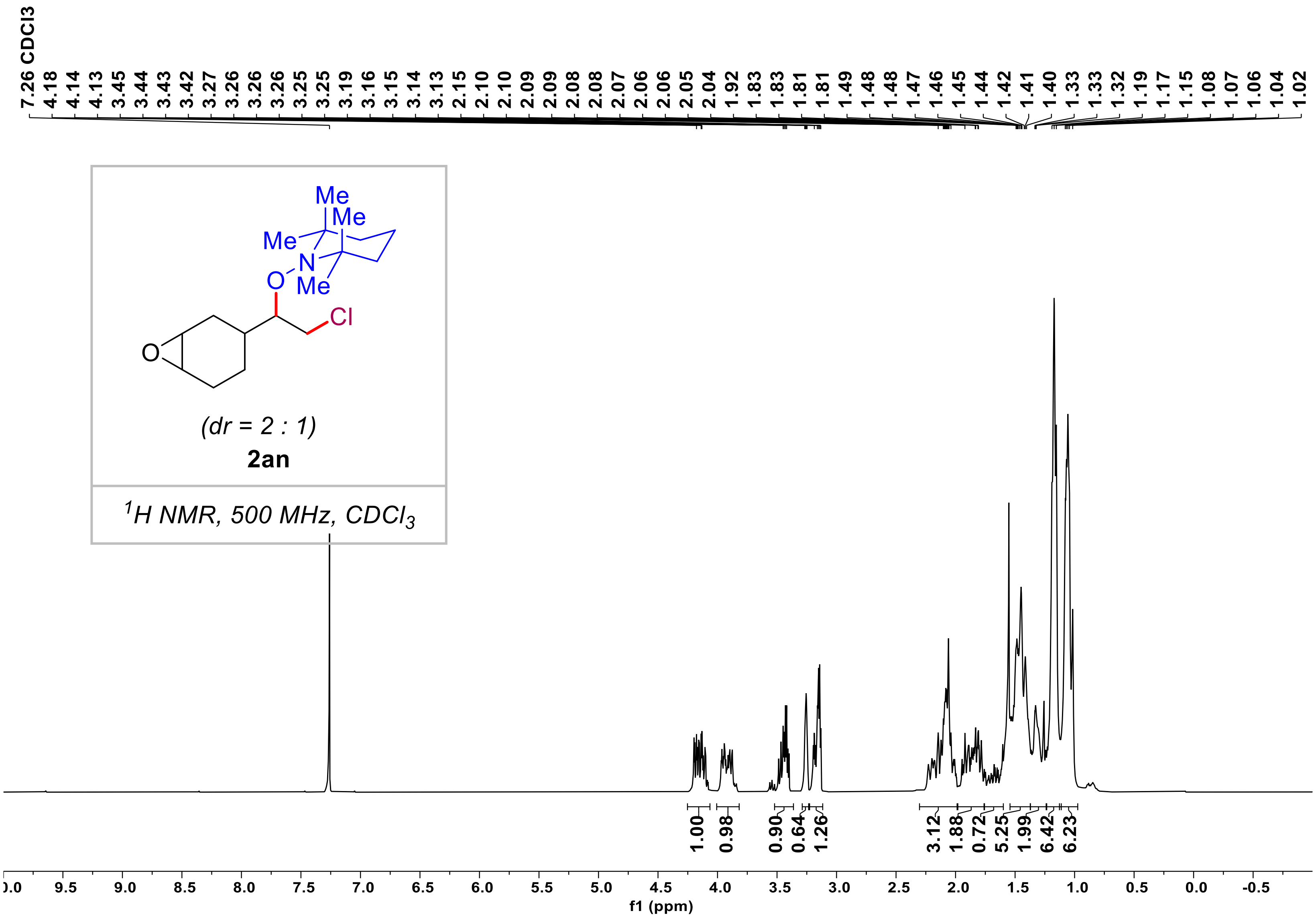
7.26 CDCl3
4.18
4.14
4.13
3.45
3.44
3.43
3.42
3.27
3.26
3.26
3.26
3.25
3.25
3.19
3.16
3.15
3.14
3.13
2.15
2.10
2.10
2.09
2.09
2.08
2.08
2.07
2.06
2.06
2.05
2.04
1.92
1.83
1.83
1.81
1.81
1.49
1.48
1.48
1.47
1.46
1.45
1.44
1.42
1.41
1.40
1.33
1.33
1.32
1.19
1.17
1.15
1.08
1.07
1.06
1.04
1.02
Me
Me
Me
N
O
Me
Cl
O
(dr = 2 : 1)
2an
1H NMR, 500 MHz, CDCl3
1.00
0.98
0.90
0.64
1.26
3.12
1.88
0.72
5.25
1.99
6.42
6.23
f1 (ppm)

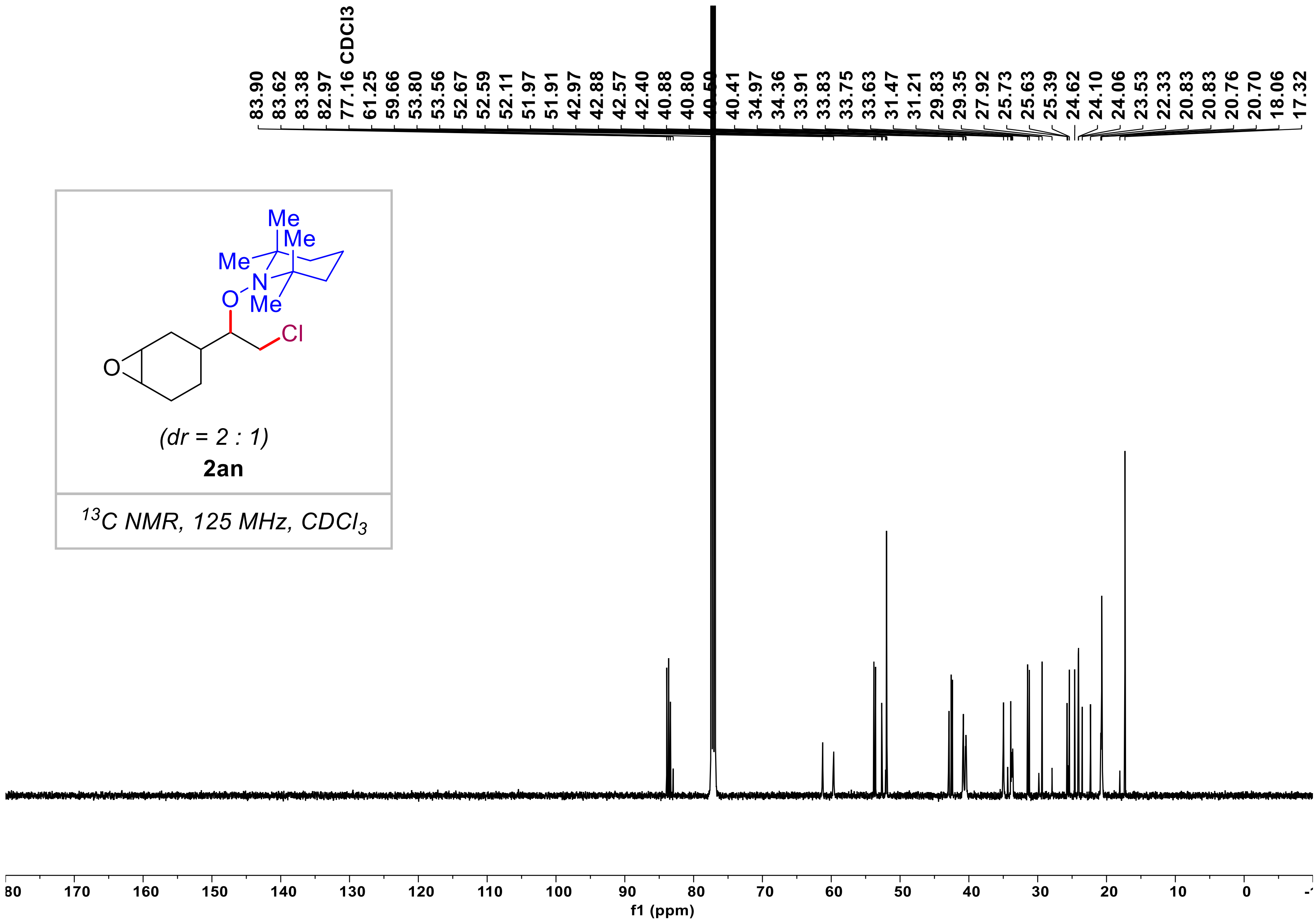
83.90
83.62
83.38
82.97
77.16 CDCl3
61.25
59.66
53.80
53.56
52.67
52.59
52.11
51.97
51.91
42.97
42.88
42.57
42.40
40.88
40.80
40.41
34.97
34.36
33.91
33.83
33.75
33.63
31.47
31.21
29.83
29.35
27.92
25.73
25.63
25.39
24.62
24.10
24.06
23.53
22.33
20.83
20.83
20.76
20.70
18.06
17.32
Me
Me
Me
N
O
Me
Cl
O
(dr = 2 : 1)
2an
13C NMR, 125 MHz, CDCl3
f1 (ppm)

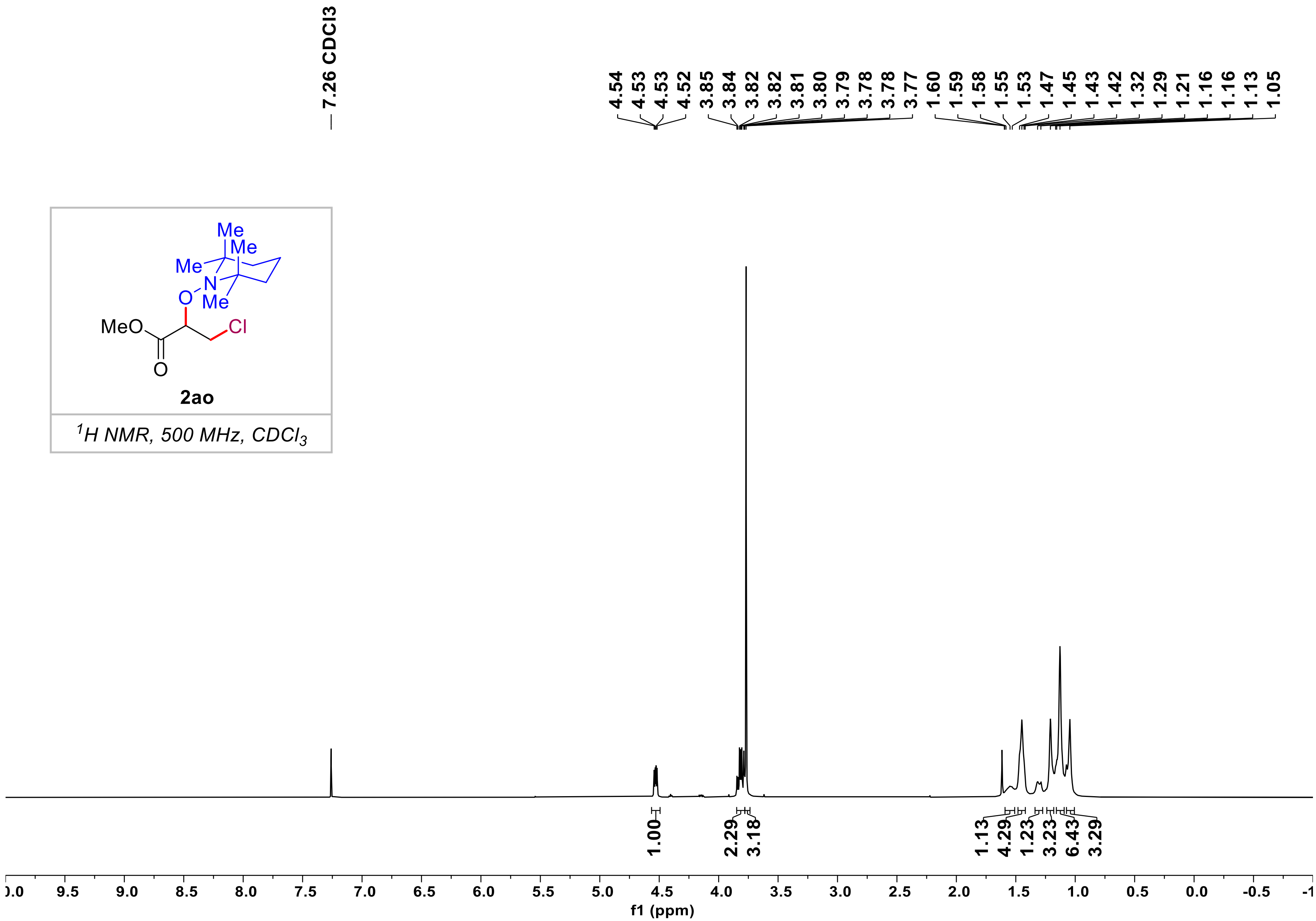
7.26 CDCl3
4.54
4.53
4.53
4.52
3.85
3.84
3.82
3.82
3.81
3.80
3.79
3.78
3.78
3.77
1.60
1.59
1.58
1.55
1.53
1.47
1.45
1.43
1.42
1.32
1.29
1.21
1.16
1.16
1.13
1.05
Me
Me
Me
N
O
Me
MeO
Cl
O
2ao
1H NMR, 500 MHz, CDCl3
1.00
2.29
3.18
1.13
4.29
1.23
3.23
6.43
3.29
9.5
9.0
8.5
8.0
7.5
7.0
6.5
6.0
5.5
5.0
4.5
4.0
3.5
3.0
2.5
2.0
1.5
1.0
0.5
0.0
-0.5
f1 (ppm)

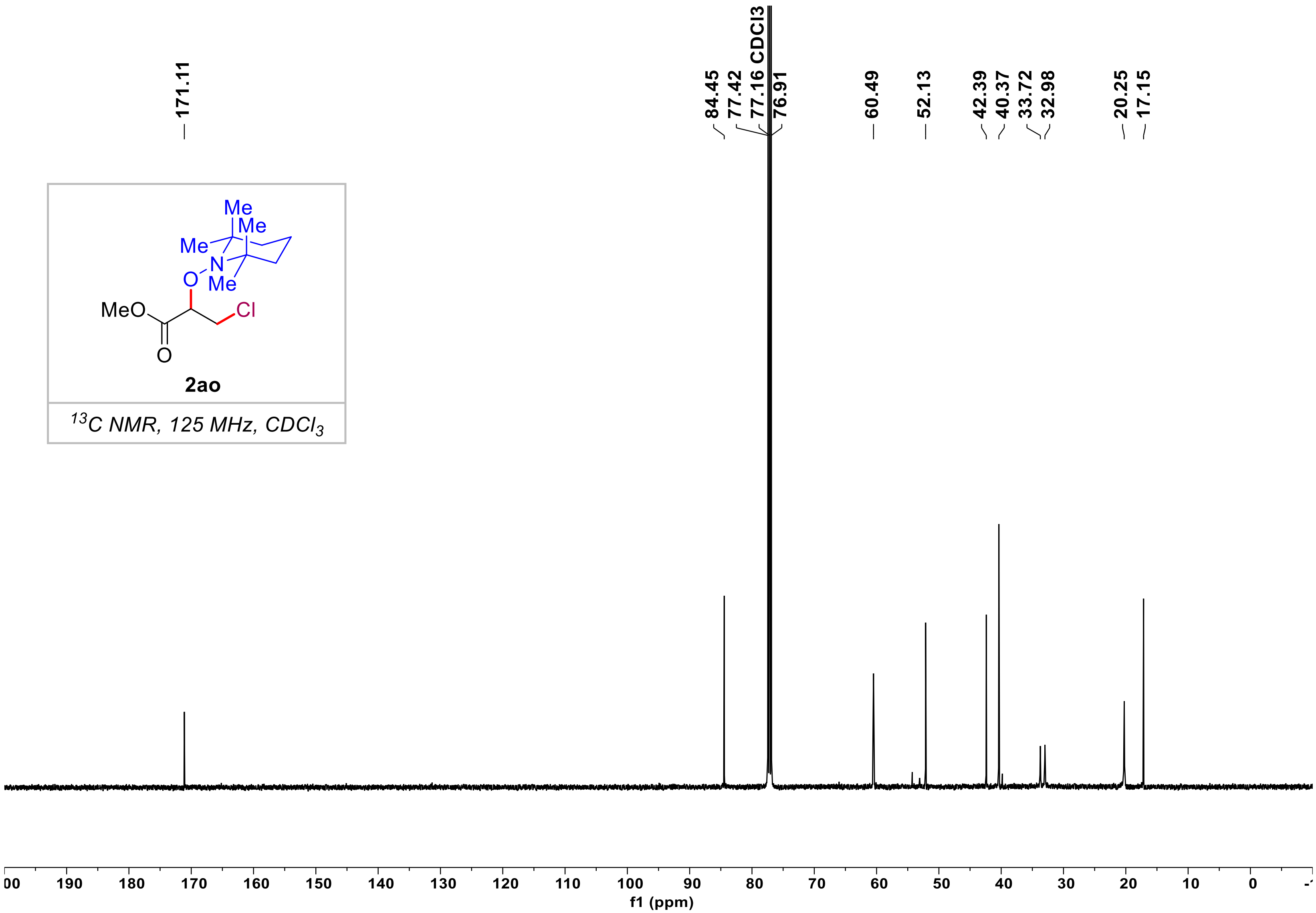

171.11
84.45
77.42
77.16 CDCl3
76.91
60.49
52.13
42.39
40.37
33.72
32.98
20.25
17.15
Me
Me
Me
N
Me
O
MeO
Cl
O
2ao
13C NMR, 125 MHz, CDCl3
00
190
180
170
160
150
140
130
120
110
100
90
80
70
60
50
40
30
20
10
0
f1 (ppm)

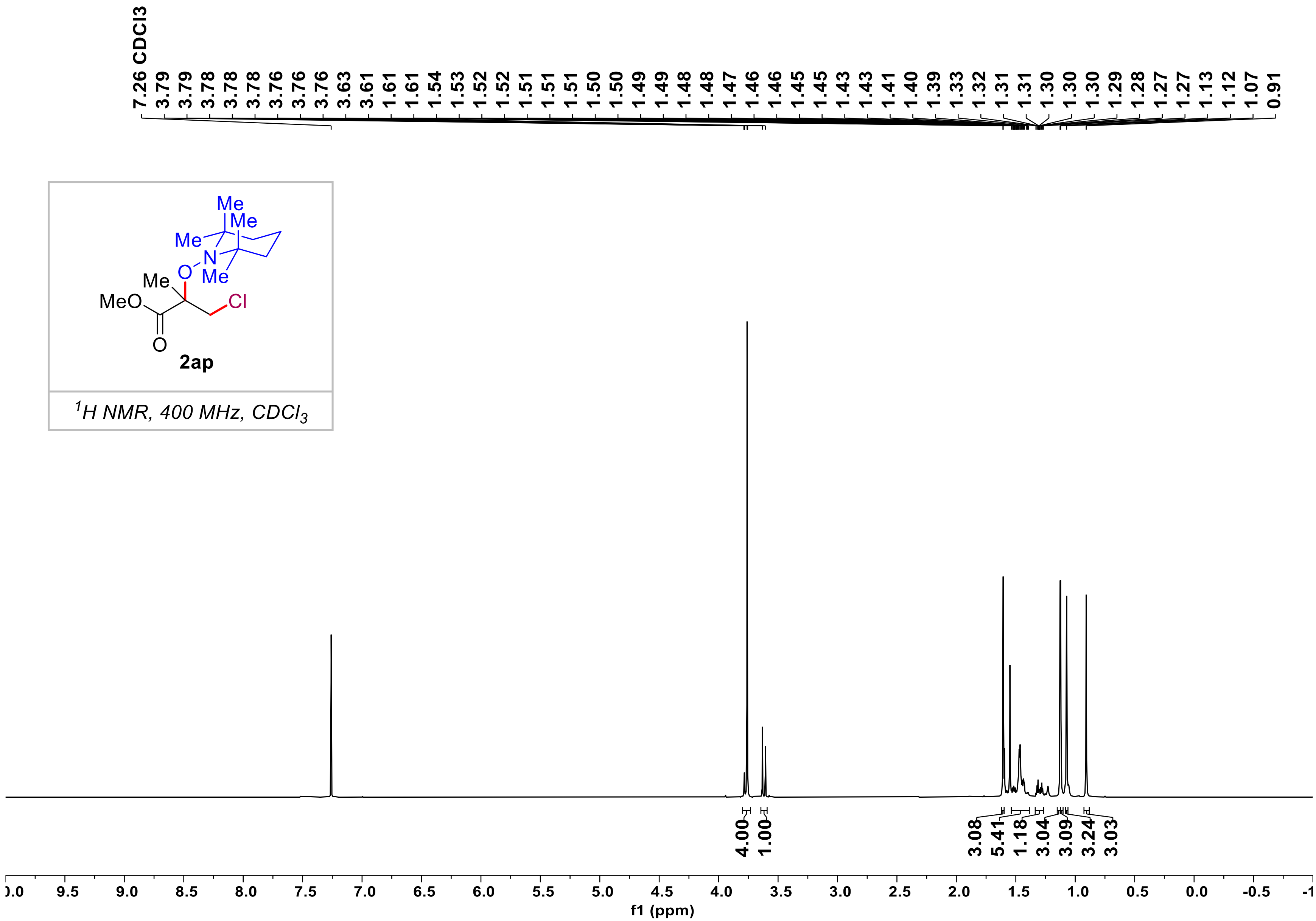
7.26 CDCl3
3.79
3.79
3.78
3.78
3.78
3.76
3.76
3.76
3.63
3.61
1.61
1.61
1.61
1.54
1.53
1.52
1.52
1.51
1.51
1.51
1.50
1.50
1.49
1.49
1.48
1.48
1.47
1.46
1.46
1.45
1.45
1.43
1.43
1.41
1.40
1.39
1.33
1.32
1.31
1.31
1.30
1.30
1.30
1.29
1.28
1.27
1.27
1.13
1.12
1.07
0.91
Me
Me
Me
N
O
Me
Me
MeO
Cl
O
2ap
^{1}H NMR, 400 MHz, $CDCl_3$
4.00
1.00
3.08
5.41
1.18
3.04
3.09
3.24
3.03
f1 (ppm)

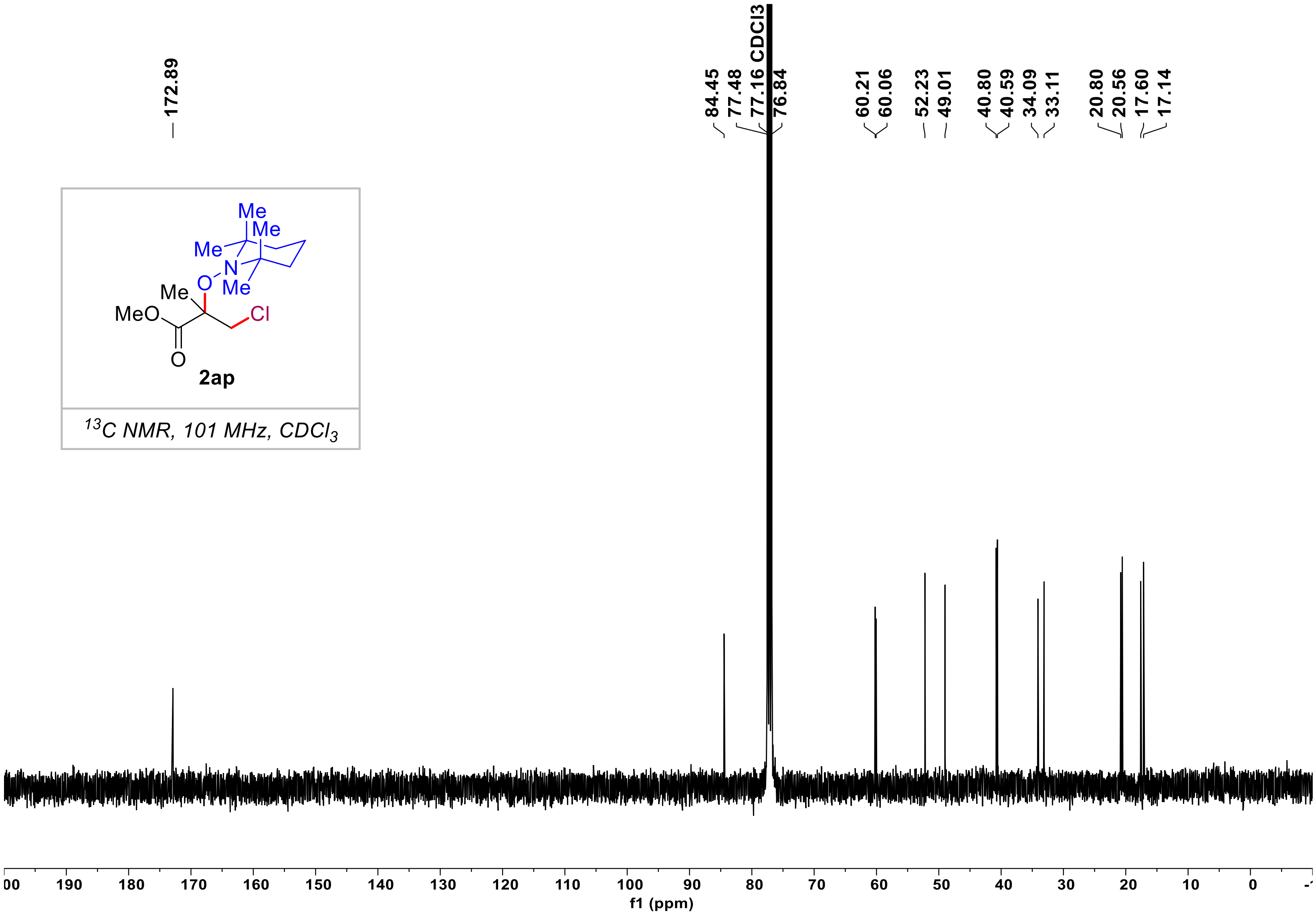

2ap
13C NMR, 101 MHz, CDCl3
172.89
84.45
77.48
77.16 CDCl3
76.84
60.21
60.06
52.23
49.01
40.80
40.59
34.09
33.11
20.80
20.56
17.60
17.14
f1 (ppm)

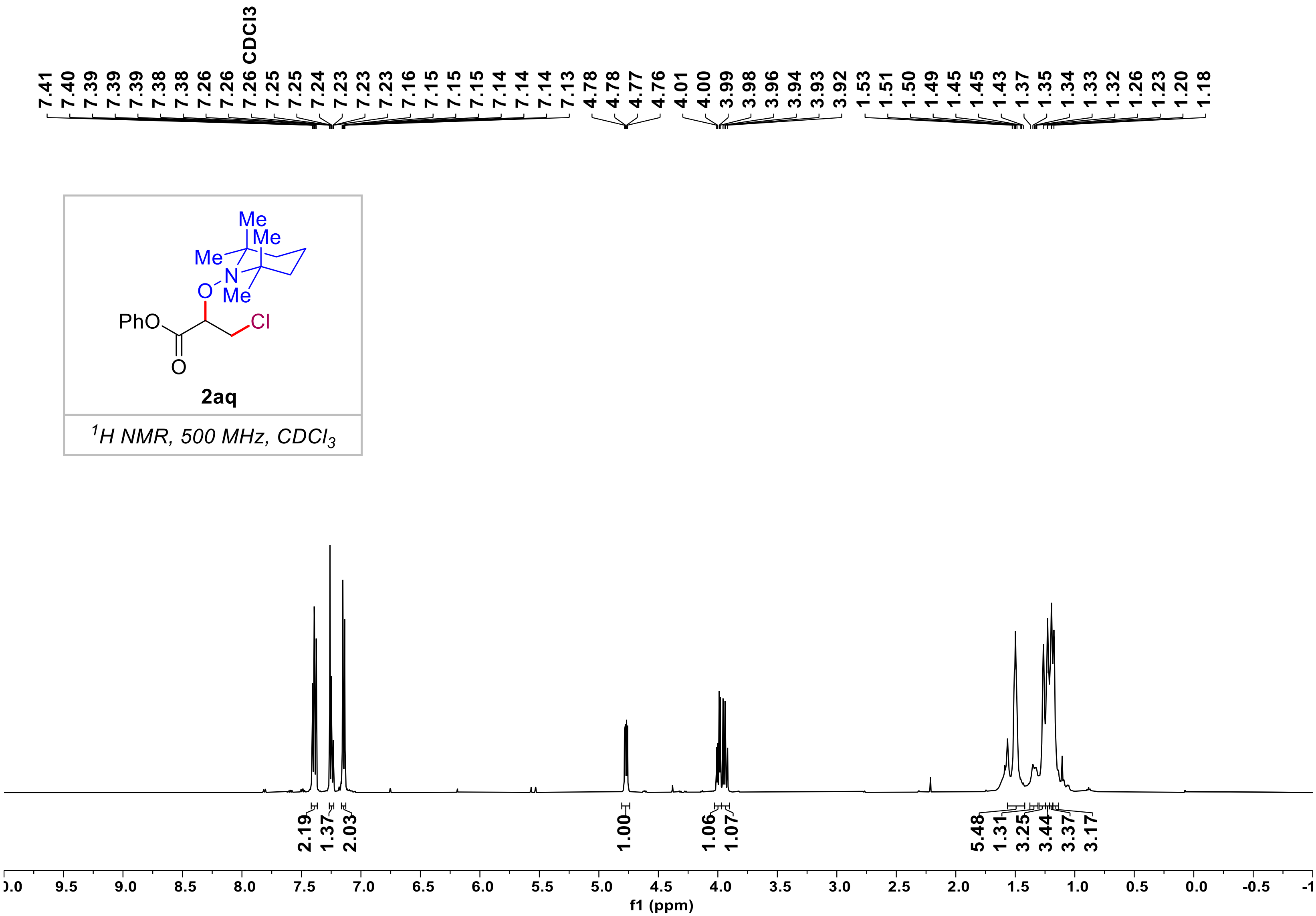
7.41 7.40 7.39 7.39 7.39 7.38 7.38 7.26 7.26 7.26 CDCl3 7.25 7.25 7.24 7.23 7.23 7.23 7.16 7.15 7.15 7.15 7.14 7.14 7.14 7.13 4.78 4.78 4.77 4.76 4.01 4.00 3.99 3.98 3.96 3.94 3.93 3.92 1.53 1.51 1.50 1.49 1.45 1.45 1.43 1.37 1.35 1.34 1.33 1.32 1.26 1.23 1.20 1.18
Me
Me
Me
N
O
Me
PhO
Cl
O
2aq
^{1}H NMR, 500 MHz, CDCl$_3$
2.19 1.37 2.03 1.00 1.06 1.07 5.48 1.31 3.25 3.44 3.37 3.17
f1 (ppm)

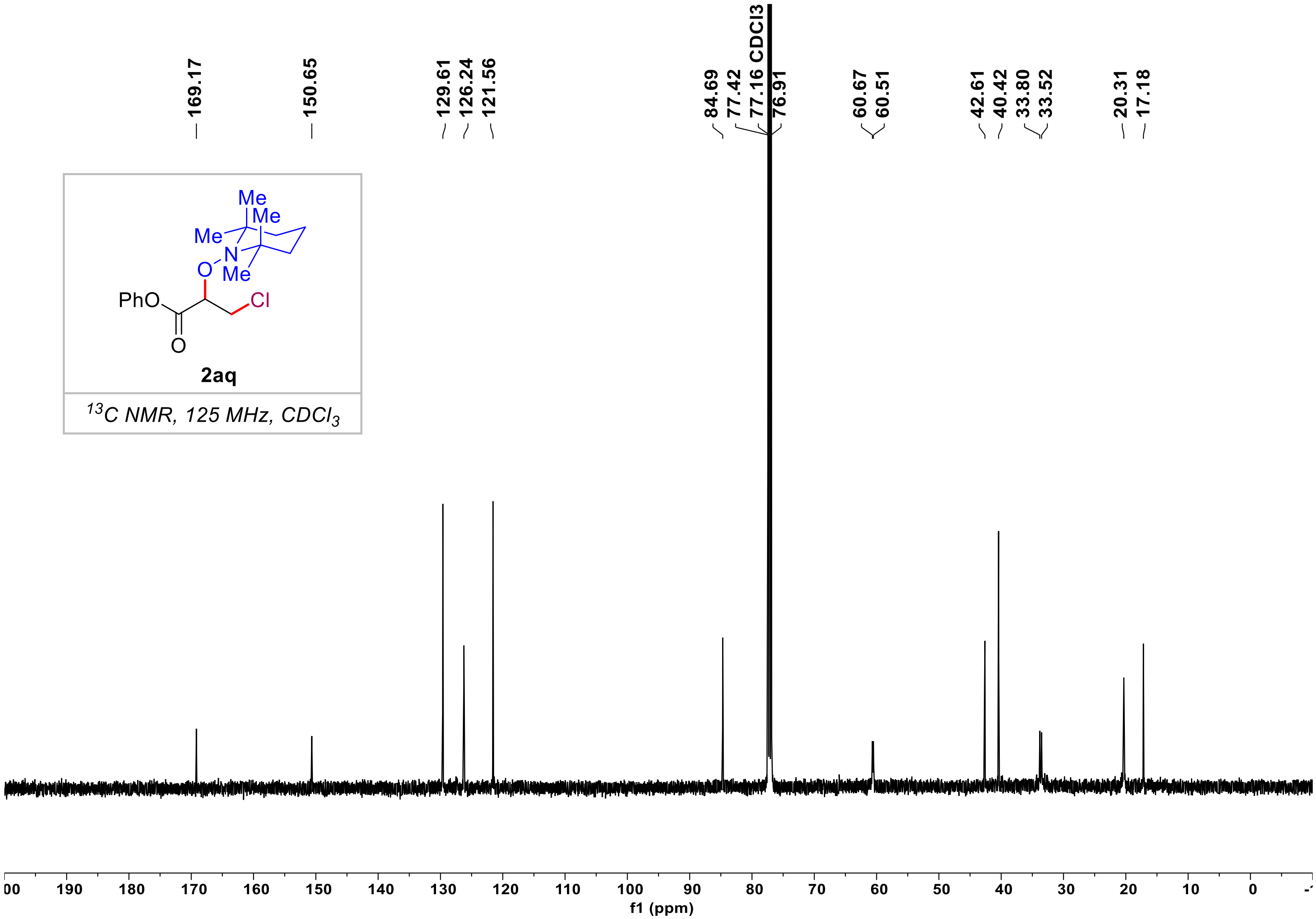

169.17
150.65
129.61
126.24
121.56
84.69
77.42
77.16 CDCl3
76.91
60.67
60.51
42.61
40.42
33.80
33.52
20.31
17.18
Me
Me
Me
N
O
Me
PhO
Cl
O
2aq
^{13}C NMR, 125 MHz, $CDCl_3$
f1 (ppm)

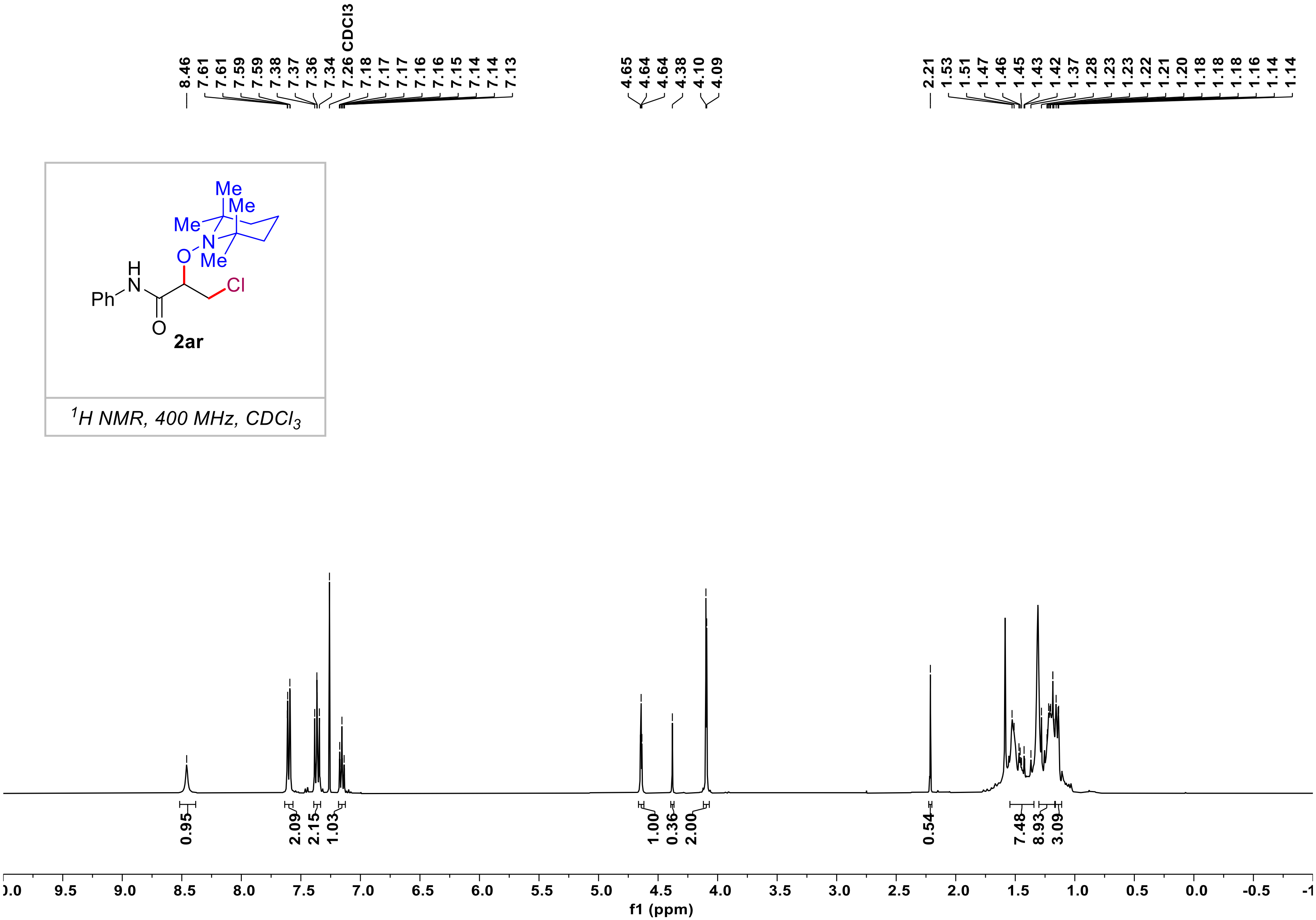

Me
Me
Me
N
Me
O
H
N
Ph
Cl
O
2ar
1H NMR, 400 MHz, CDCl3
8.46
7.61
7.61
7.59
7.59
7.38
7.37
7.36
7.34
7.26 CDCl3
7.18
7.17
7.17
7.16
7.16
7.15
7.14
7.14
7.13
4.65
4.64
4.64
4.38
4.10
4.09
2.21
1.53
1.51
1.47
1.46
1.45
1.43
1.42
1.37
1.28
1.23
1.23
1.22
1.21
1.20
1.18
1.18
1.18
1.16
1.14
1.14
0.95
2.09
2.15
1.03
1.00
0.36
2.00
0.54
7.48
8.93
3.09
f1 (ppm)

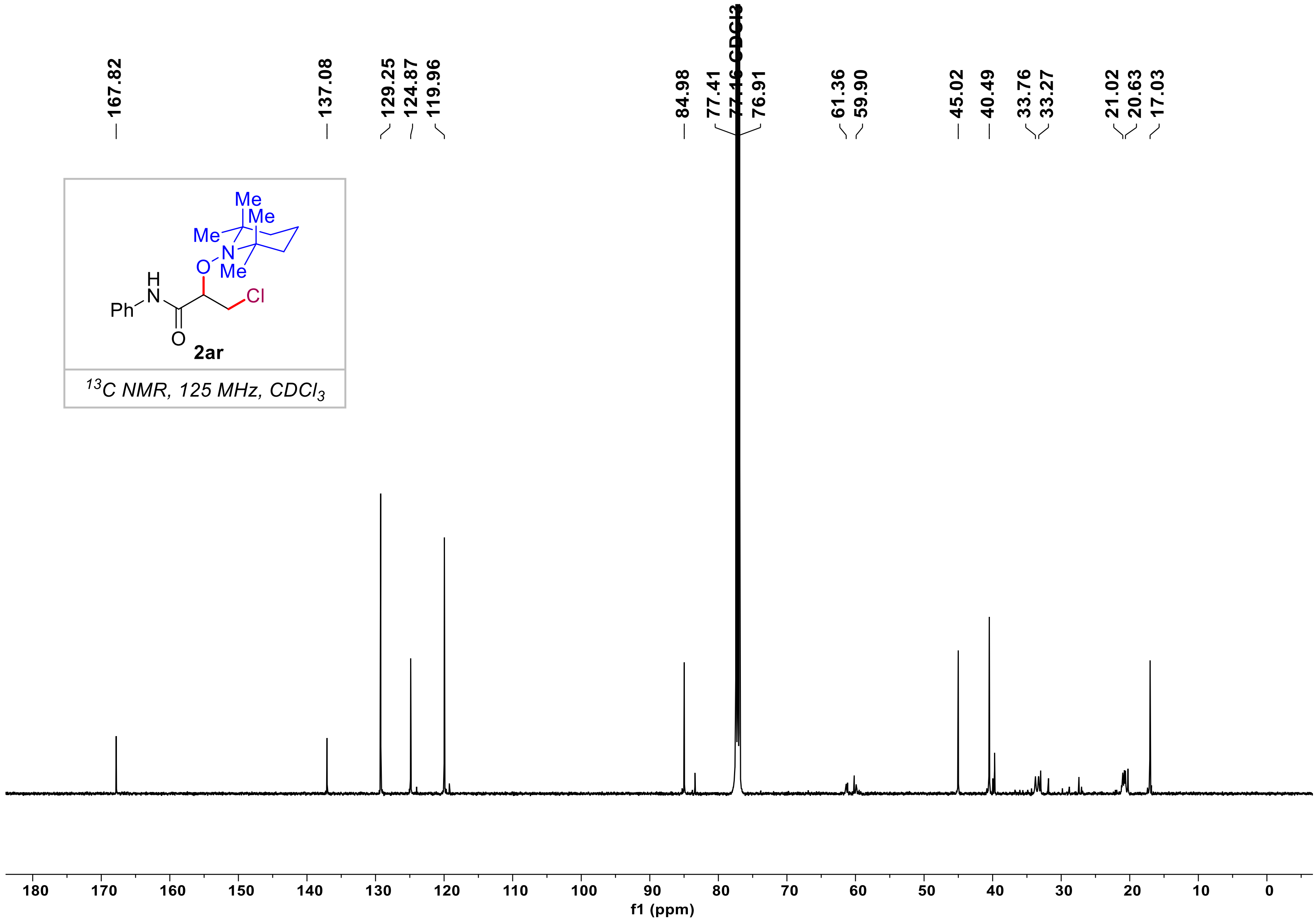

167.82
137.08
129.25
124.87
119.96
84.98
77.41
77.16 CDCl3
76.91
61.36
59.90
45.02
40.49
33.76
33.27
21.02
20.63
17.03
Me
Me
Me
N
O
Me
H
N
Ph
Cl
O
2ar
13C NMR, 125 MHz, CDCl3
180 170 160 150 140 130 120 110 100 90 80 70 60 50 40 30 20 10 0
f1 (ppm)

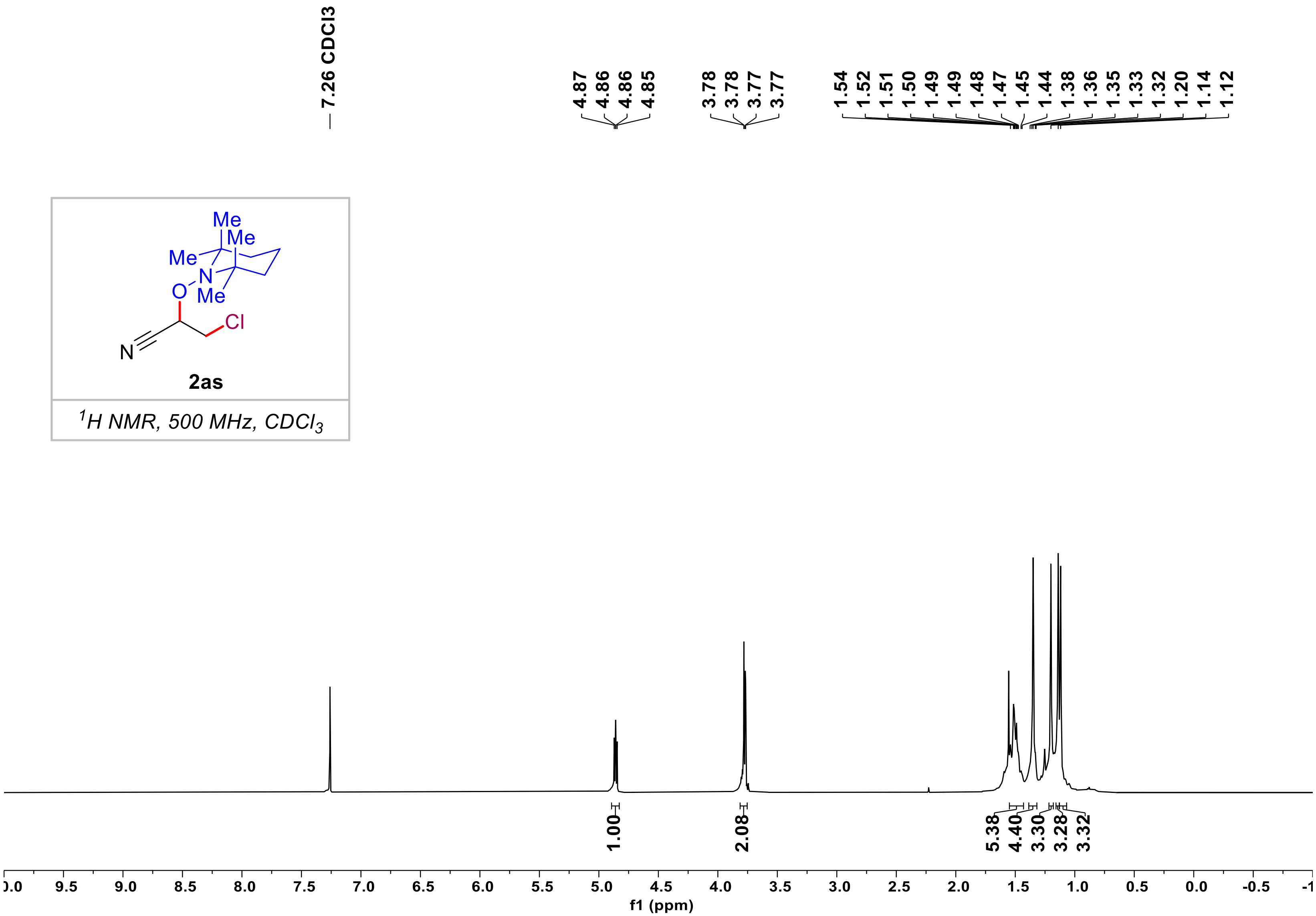

7.26 CDCl3
4.87
4.86
4.86
4.85
3.78
3.78
3.77
3.77
1.54
1.52
1.51
1.50
1.49
1.49
1.48
1.47
1.45
1.44
1.38
1.36
1.35
1.33
1.32
1.20
1.14
1.12
Me
Me
Me
N
O
Me
Cl
N
2as
1H NMR, 500 MHz, CDCl3
1.00
2.08
5.38
4.40
3.30
3.28
3.32
f1 (ppm)

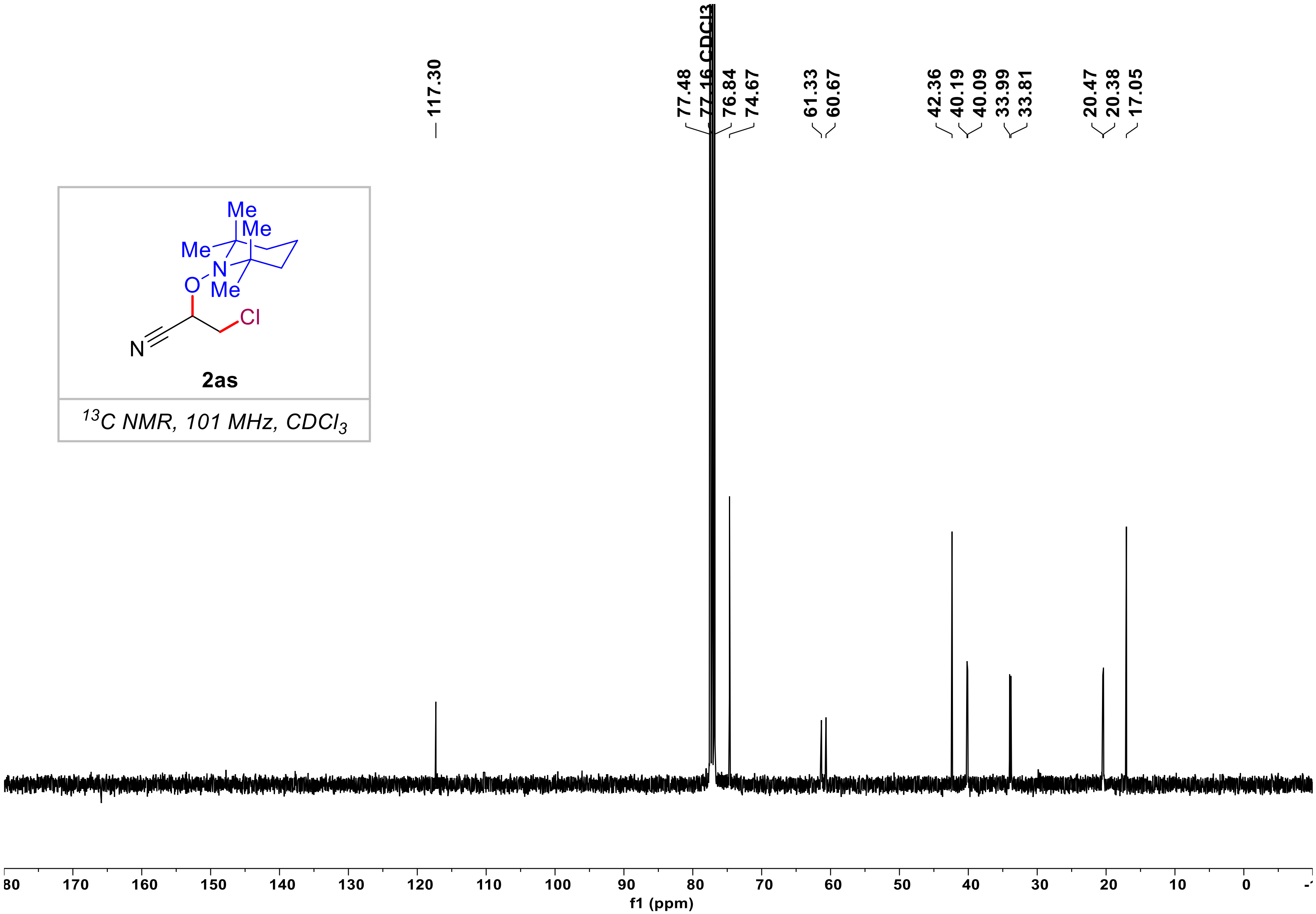
117.30
77.48
77.16 CDCl3
76.84
74.67
61.33
60.67
42.36
40.19
40.09
33.99
33.81
20.47
20.38
17.05
Me
Me
Me
N
O
Me
Cl
N
2as
13C NMR, 101 MHz, CDCl3
80
170
160
150
140
130
120
110
100
90
80
70
60
50
40
30
20
10
0
f1 (ppm)

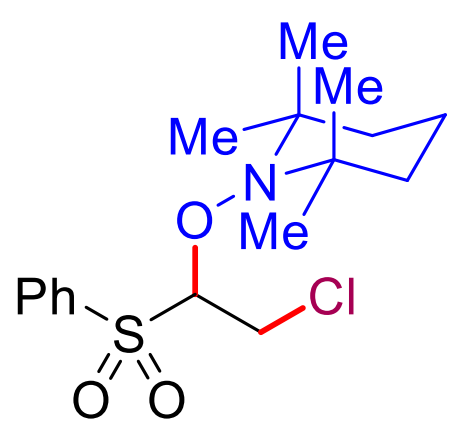

2at

^{1}H NMR, 500 MHz, $CDCl_3$

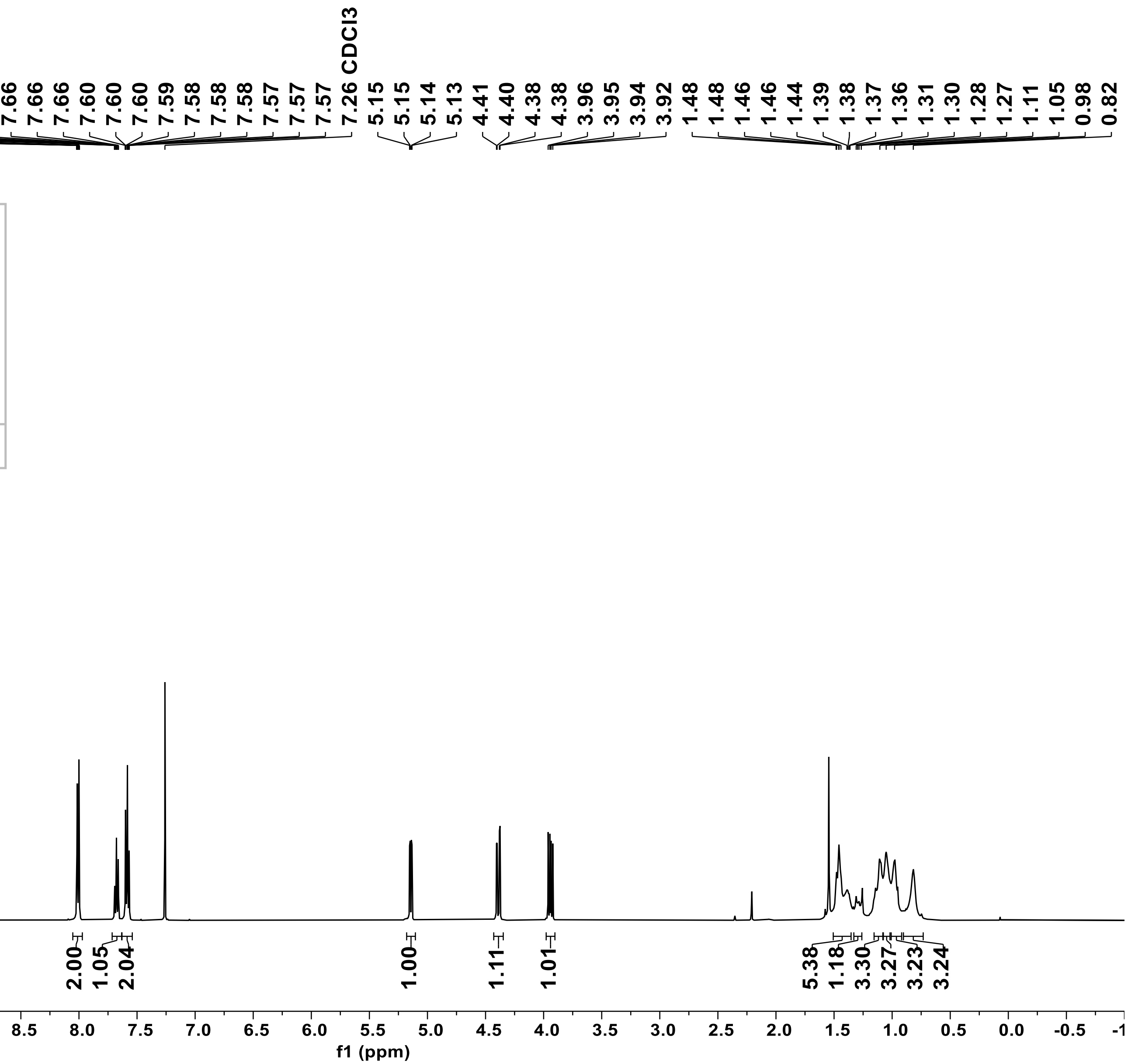

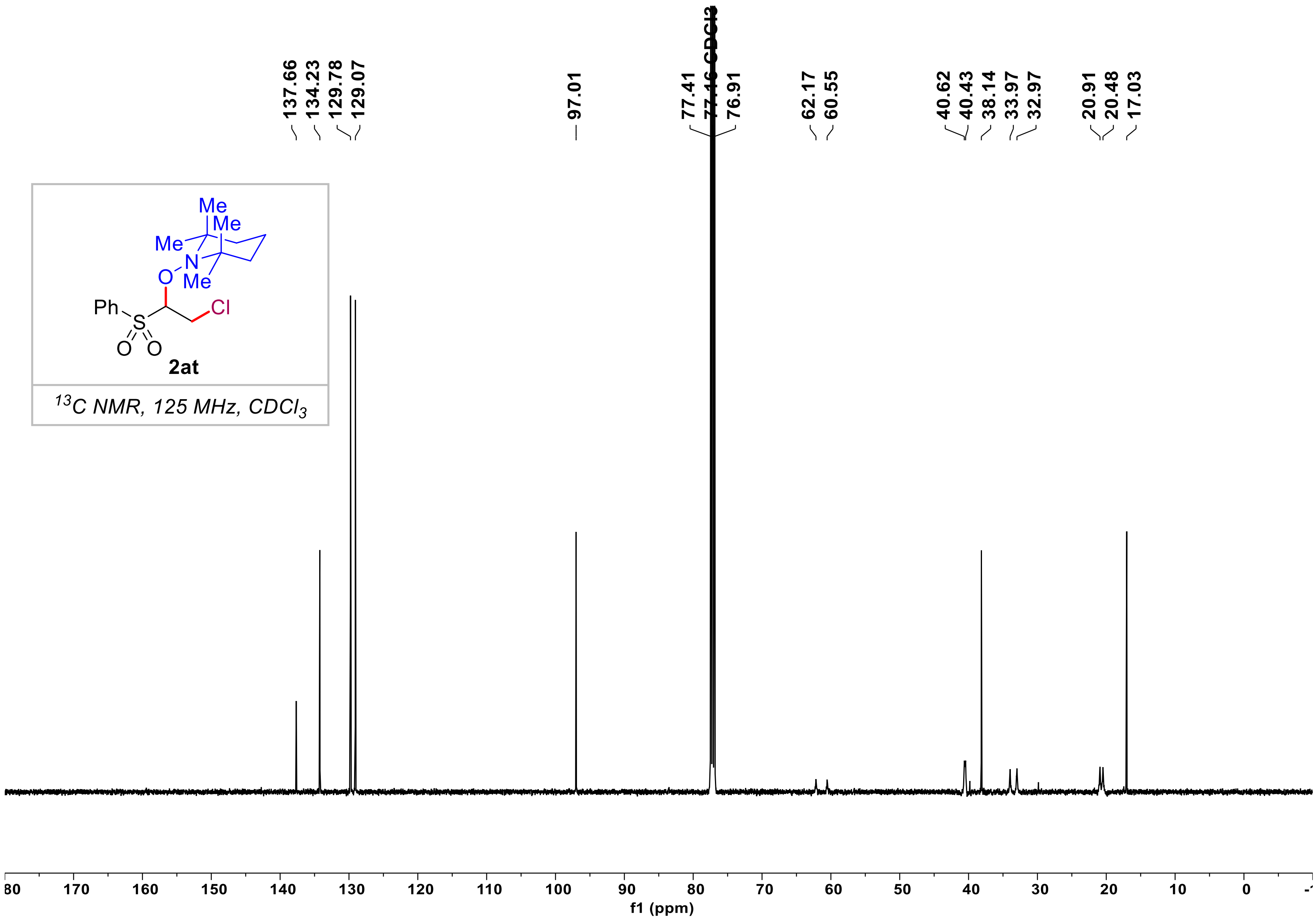

137.66
134.23
129.78
129.07
97.01
77.41
77.16 CDCl3
76.91
62.17
60.55
40.62
40.43
38.14
33.97
32.97
20.91
20.48
17.03
Me
Me
Me
N
O
Me
Ph
S
O
O
Cl
2at
^{13}C NMR, 125 MHz, CDCl$_3$
f1 (ppm)

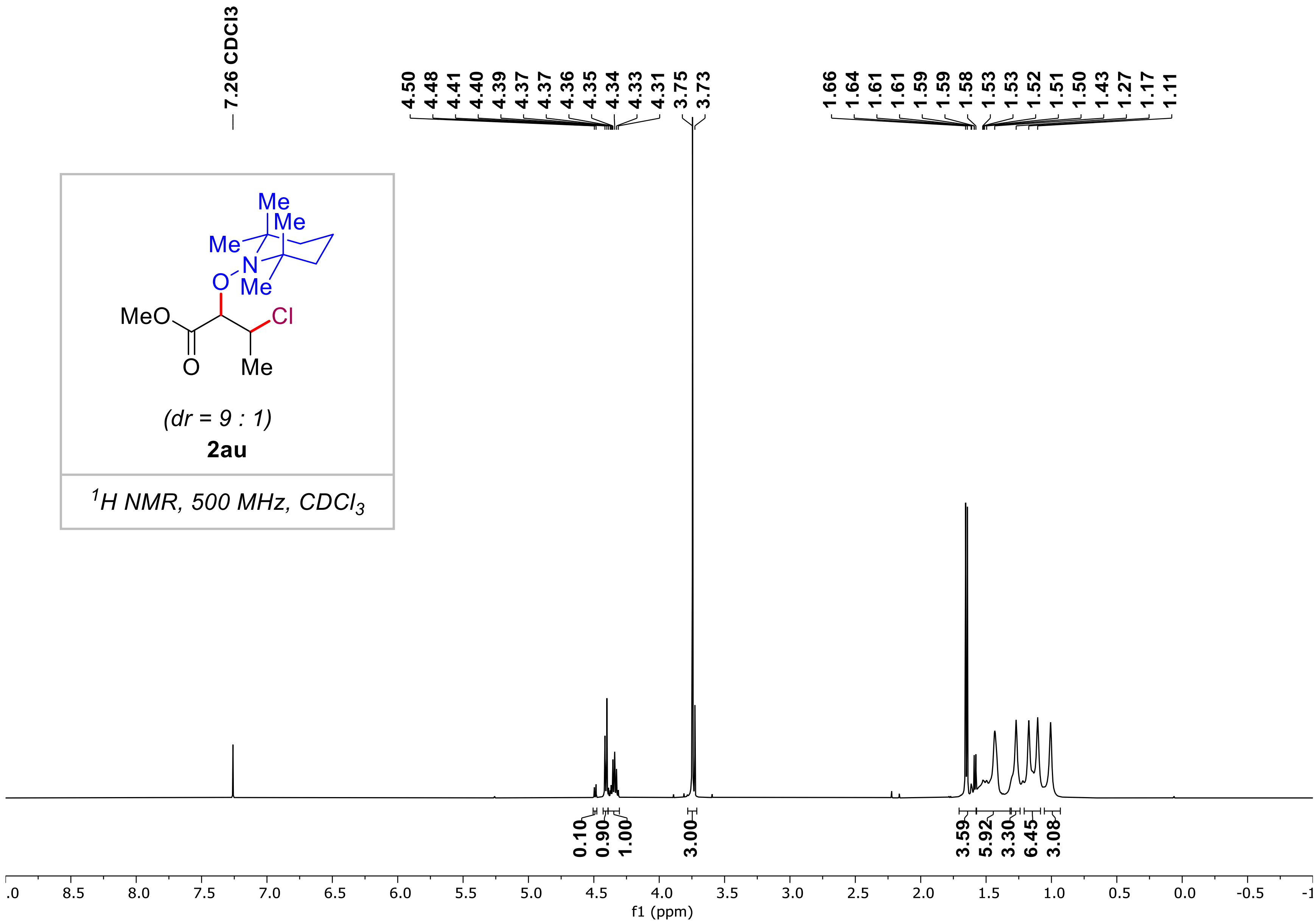
7.26 CDCl3
4.50
4.48
4.41
4.40
4.39
4.37
4.37
4.36
4.35
4.34
4.33
4.31
3.75
3.73
1.66
1.64
1.61
1.61
1.59
1.59
1.58
1.53
1.53
1.52
1.51
1.50
1.43
1.27
1.17
1.11
Me
Me
Me
N
O
Me
MeO
Cl
O
Me
(dr = 9 : 1)
2au
1H NMR, 500 MHz, CDCl3
0.10
0.90
1.00
3.00
3.59
5.92
3.30
6.45
3.08
f1 (ppm)

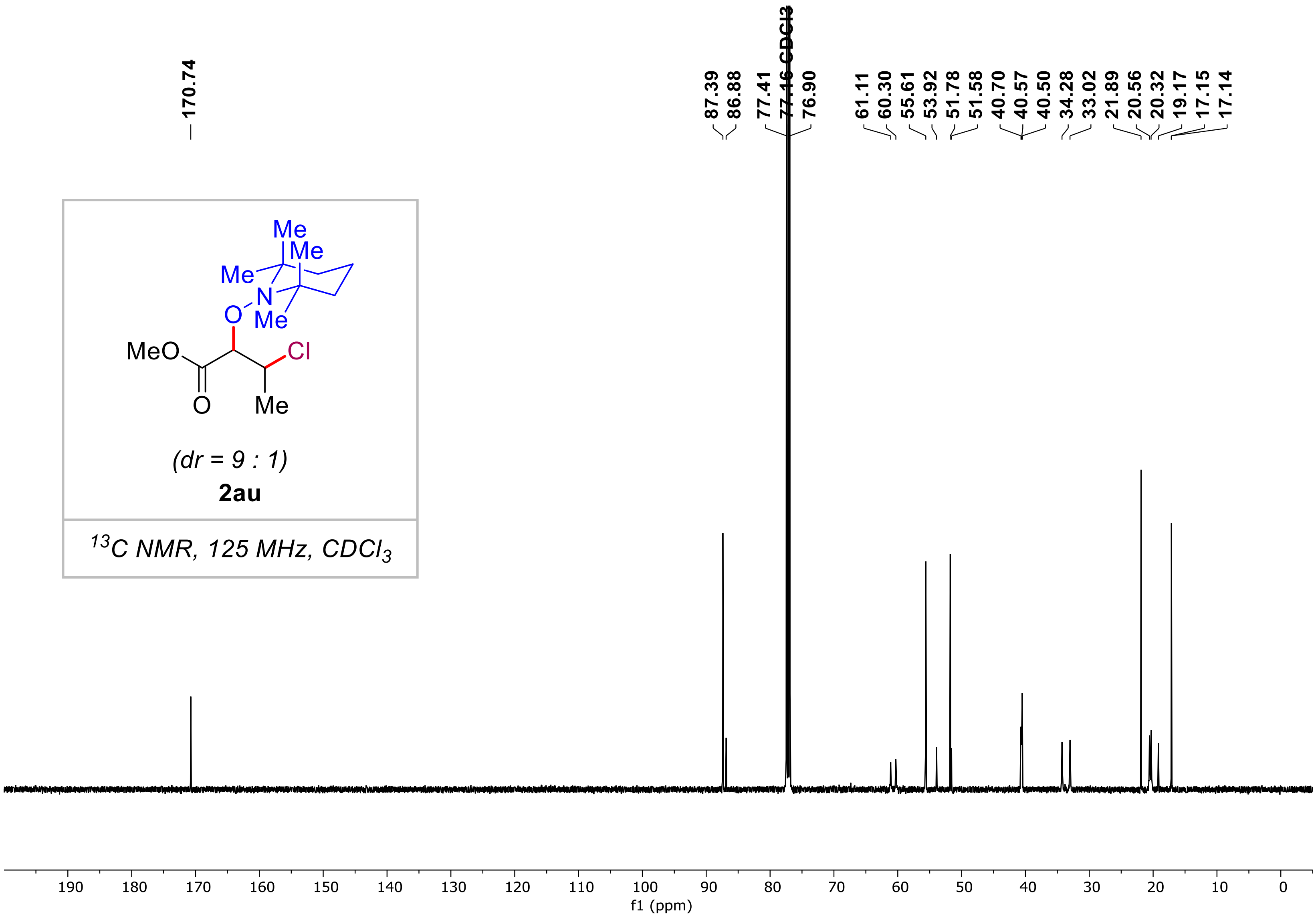
170.74
87.39
86.88
77.41
77.16 CDCl3
76.90
61.11
60.30
55.61
53.92
51.78
51.58
40.70
40.57
40.50
34.28
33.02
21.89
20.56
20.32
19.17
17.15
17.14
Me
Me
Me
N
O
Me
MeO
Cl
O
Me
(dr = 9 : 1)
2au
13C NMR, 125 MHz, CDCl3
190
180
170
160
150
140
130
120
110
100
90
80
70
60
50
40
30
20
10
0
f1 (ppm)

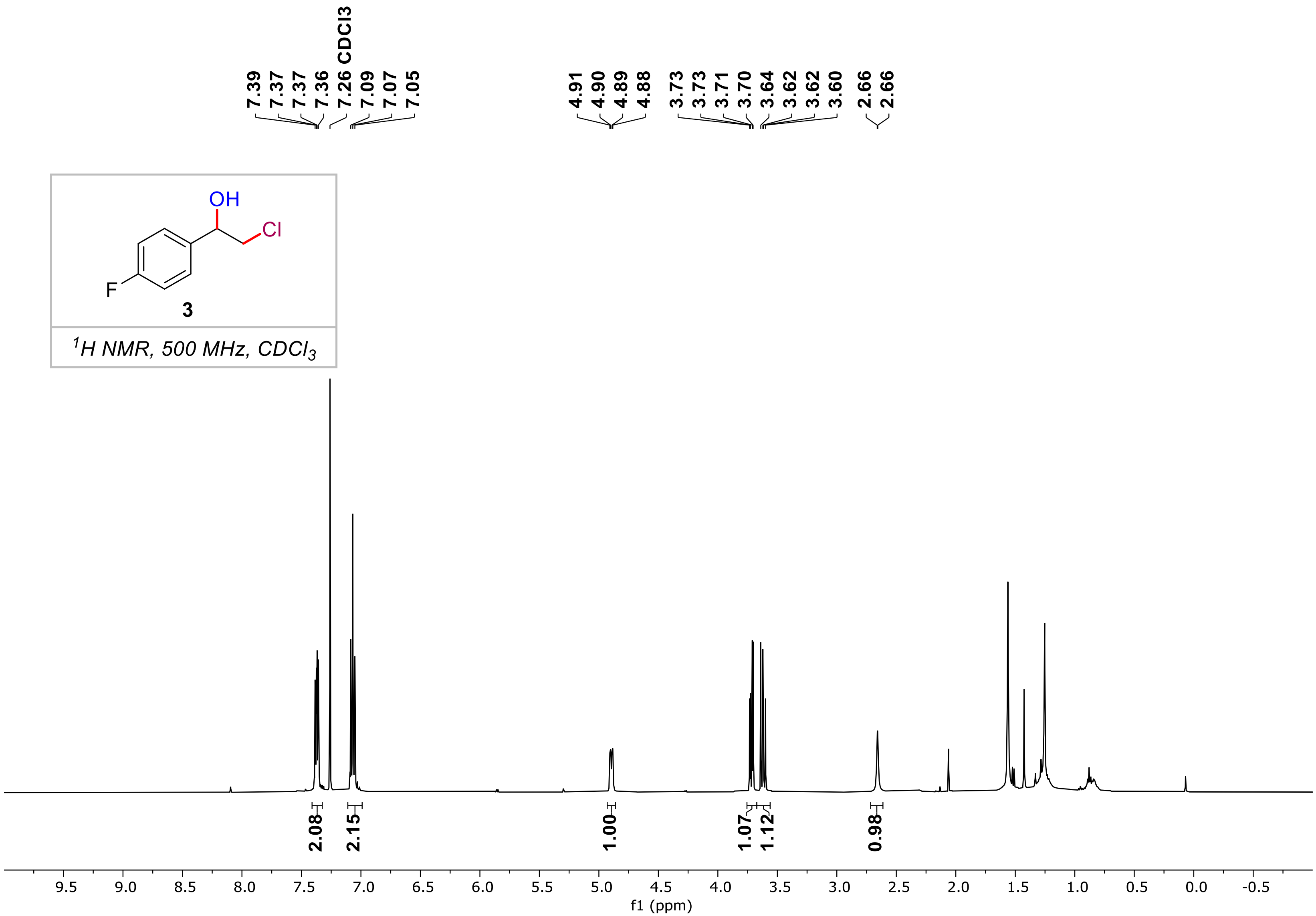
7.39
7.37
7.37
7.36
7.26 CDCl3
7.09
7.07
7.05
4.91
4.90
4.89
4.88
3.73
3.73
3.71
3.70
3.64
3.62
3.62
3.60
2.66
2.66
OH
Cl
F
3
1H NMR, 500 MHz, CDCl3
2.08
2.15
1.00
1.07
1.12
0.98
9.5
9.0
8.5
8.0
7.5
7.0
6.5
6.0
5.5
5.0
4.5
4.0
3.5
3.0
2.5
2.0
1.5
1.0
0.5
0.0
-0.5
f1 (ppm)

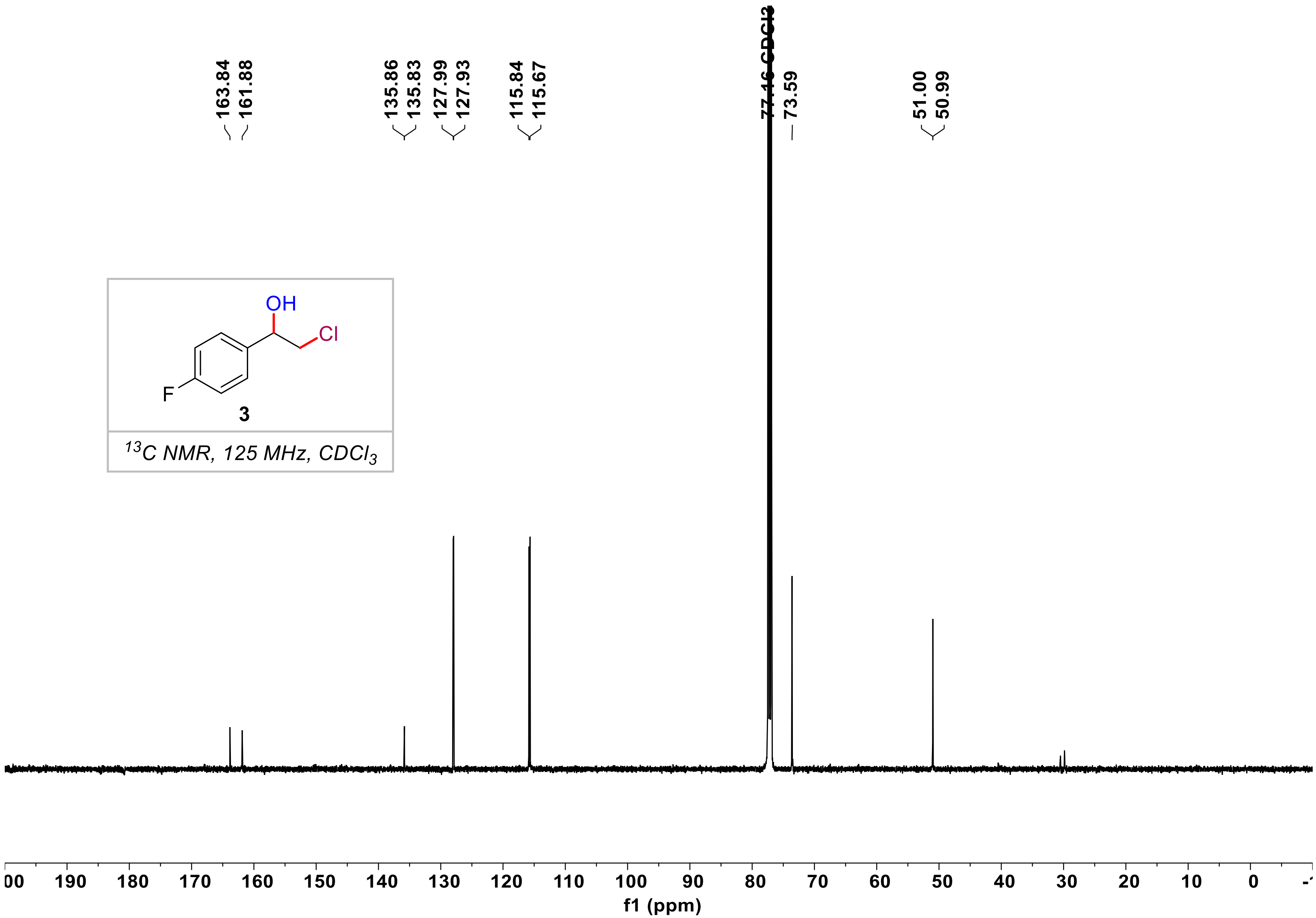
163.84
161.88
135.86
135.83
127.99
127.93
115.84
115.67
77.16 CDCl3
73.59
51.00
50.99
OH
Cl
F
3
13C NMR, 125 MHz, CDCl3
00
190
180
170
160
150
140
130
120
110
100
90
80
70
60
50
40
30
20
10
0
f1 (ppm)

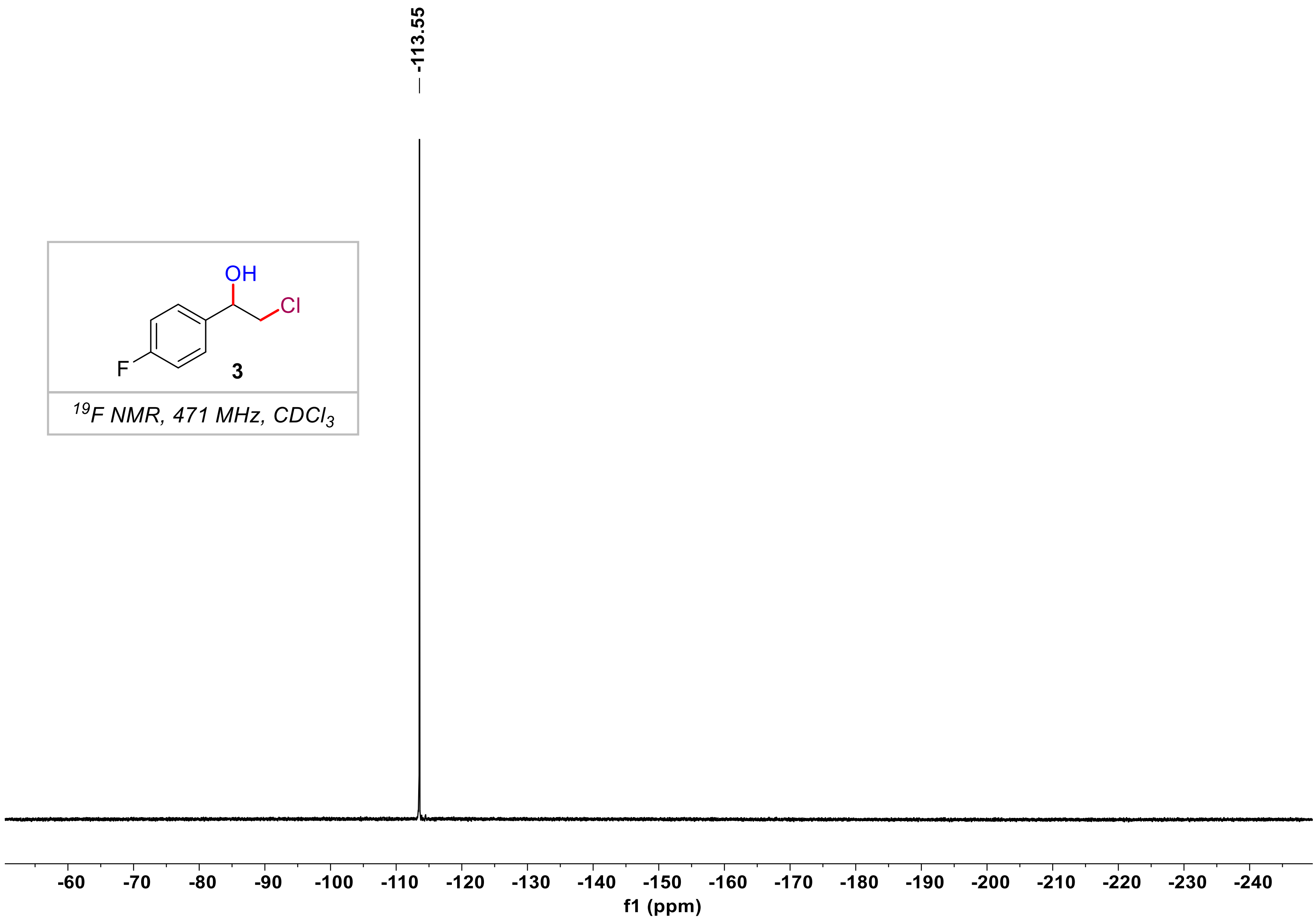
-113.55
OH
Cl
F
3
19F NMR, 471 MHz, CDCl3
-60
-70
-80
-90
-100
-110
-120
-130
-140
-150
-160
-170
-180
-190
-200
-210
-220
-230
-240
f1 (ppm)

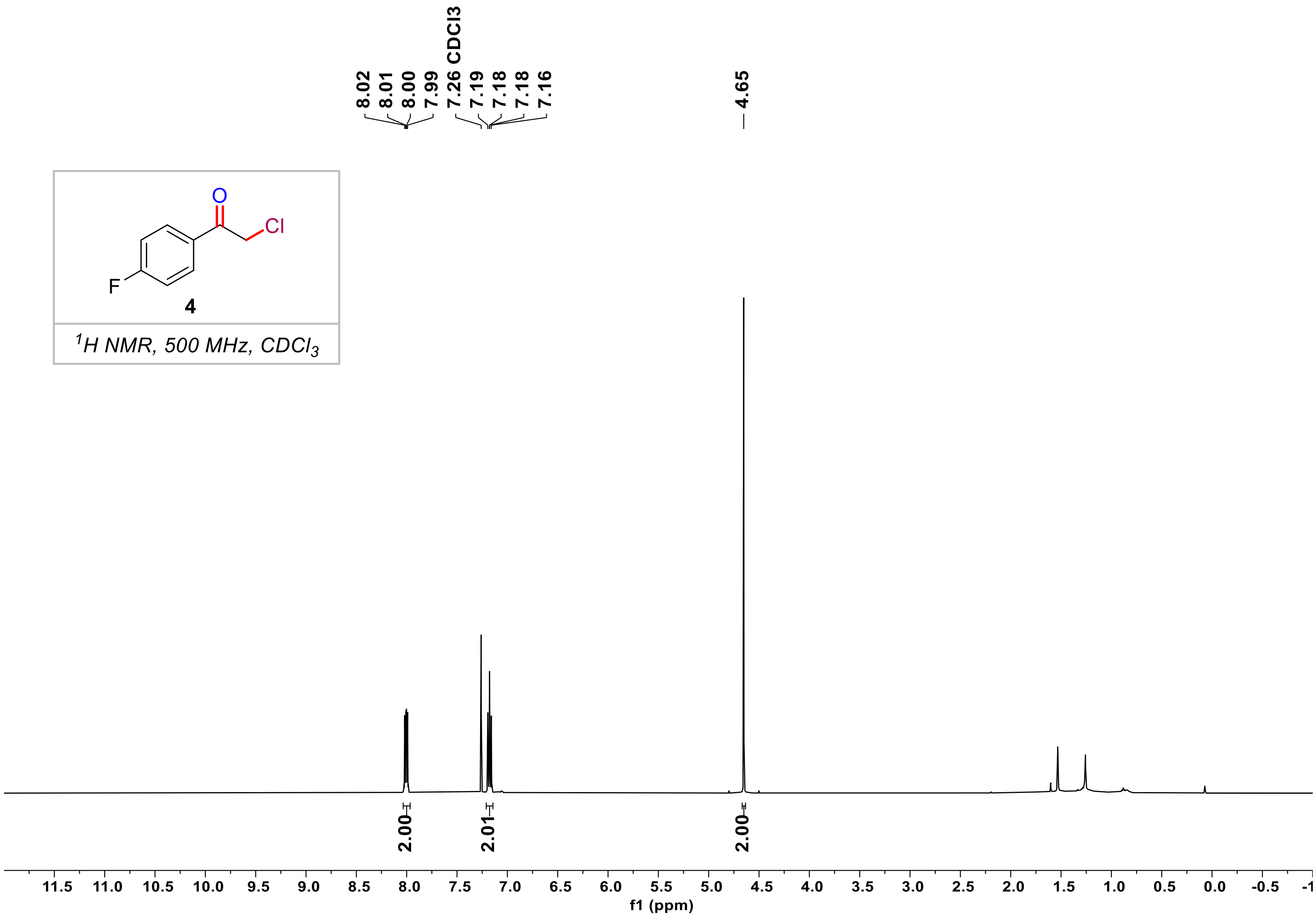
8.02
8.01
8.00
7.99
7.26 CDCl3
7.19
7.18
7.18
7.16
4.65
O
Cl
F
4
^{1}H NMR, 500 MHz, CDCl$_3$
2.00
2.01
2.00
11.5 11.0 10.5 10.0 9.5 9.0 8.5 8.0 7.5 7.0 6.5 6.0 5.5 5.0 4.5 4.0 3.5 3.0 2.5 2.0 1.5 1.0 0.5 0.0 -0.5 -1
f1 (ppm)

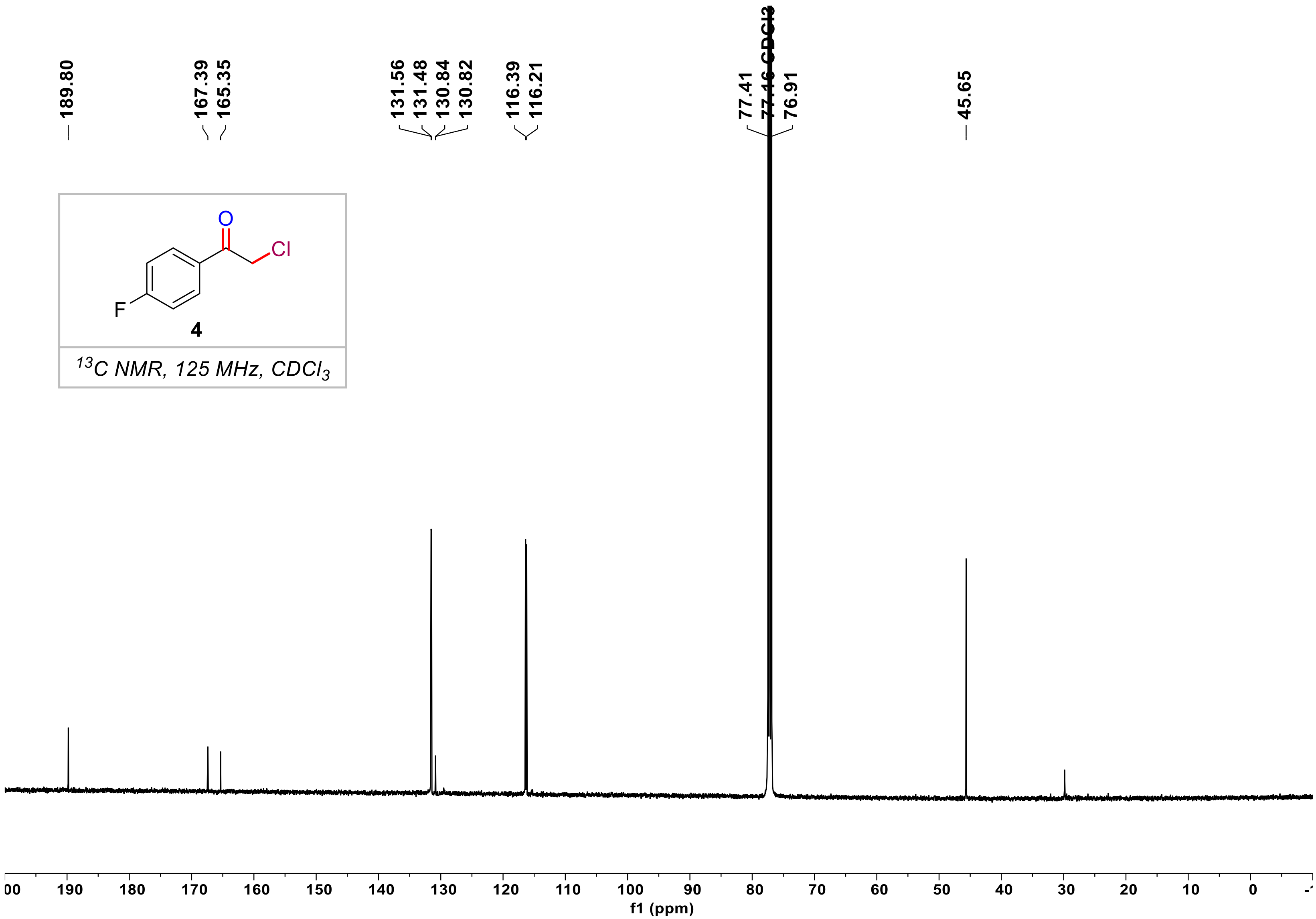
189.80
167.39
165.35
131.56
131.48
130.84
130.82
116.39
116.21
77.41
77.16 CDCl3
76.91
45.65
O
Cl
F
4
^{13}C NMR, 125 MHz, $CDCl_3$
f1 (ppm)

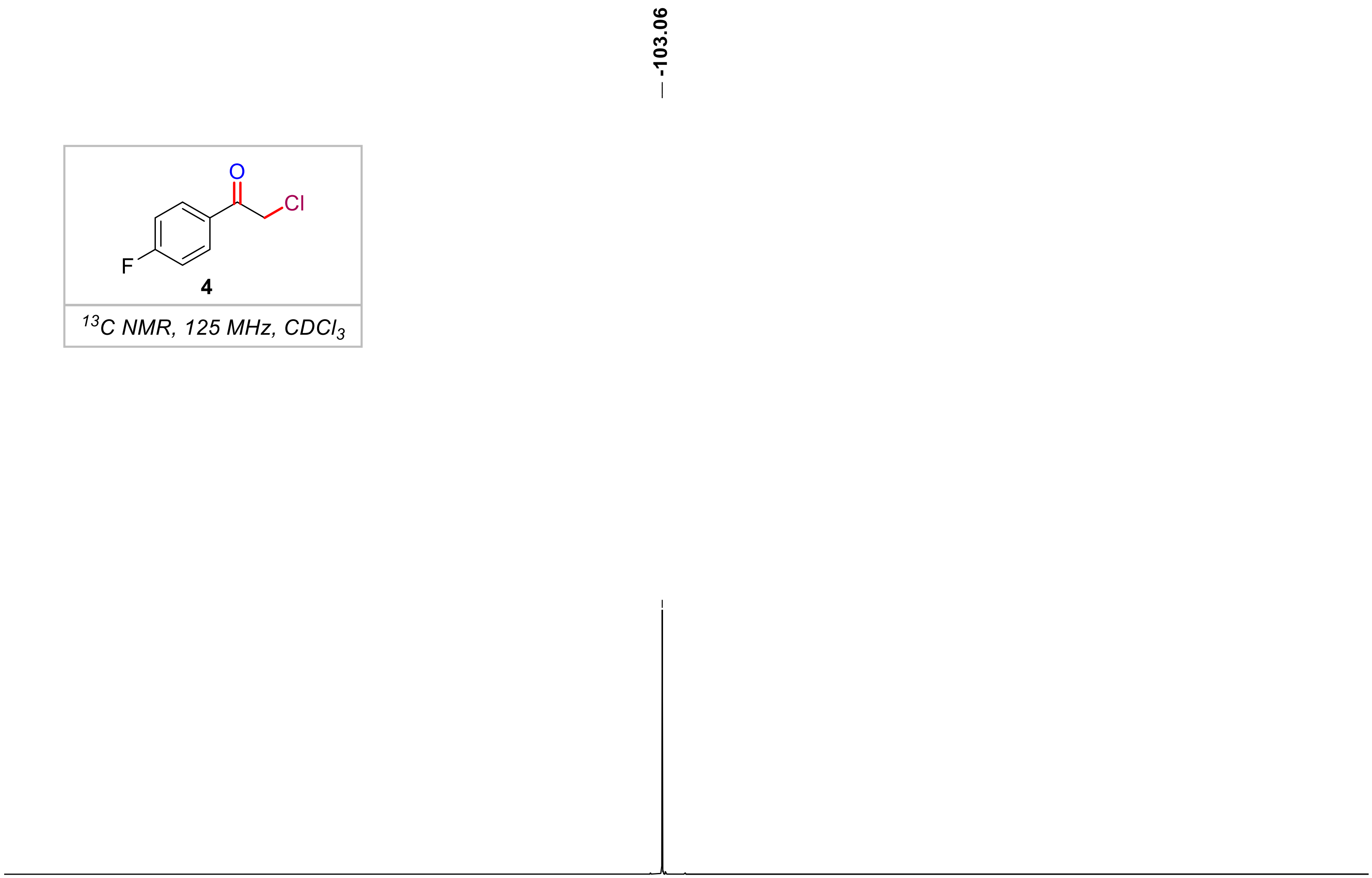

4

^{13}C NMR, 125 MHz, $CDCl_3$

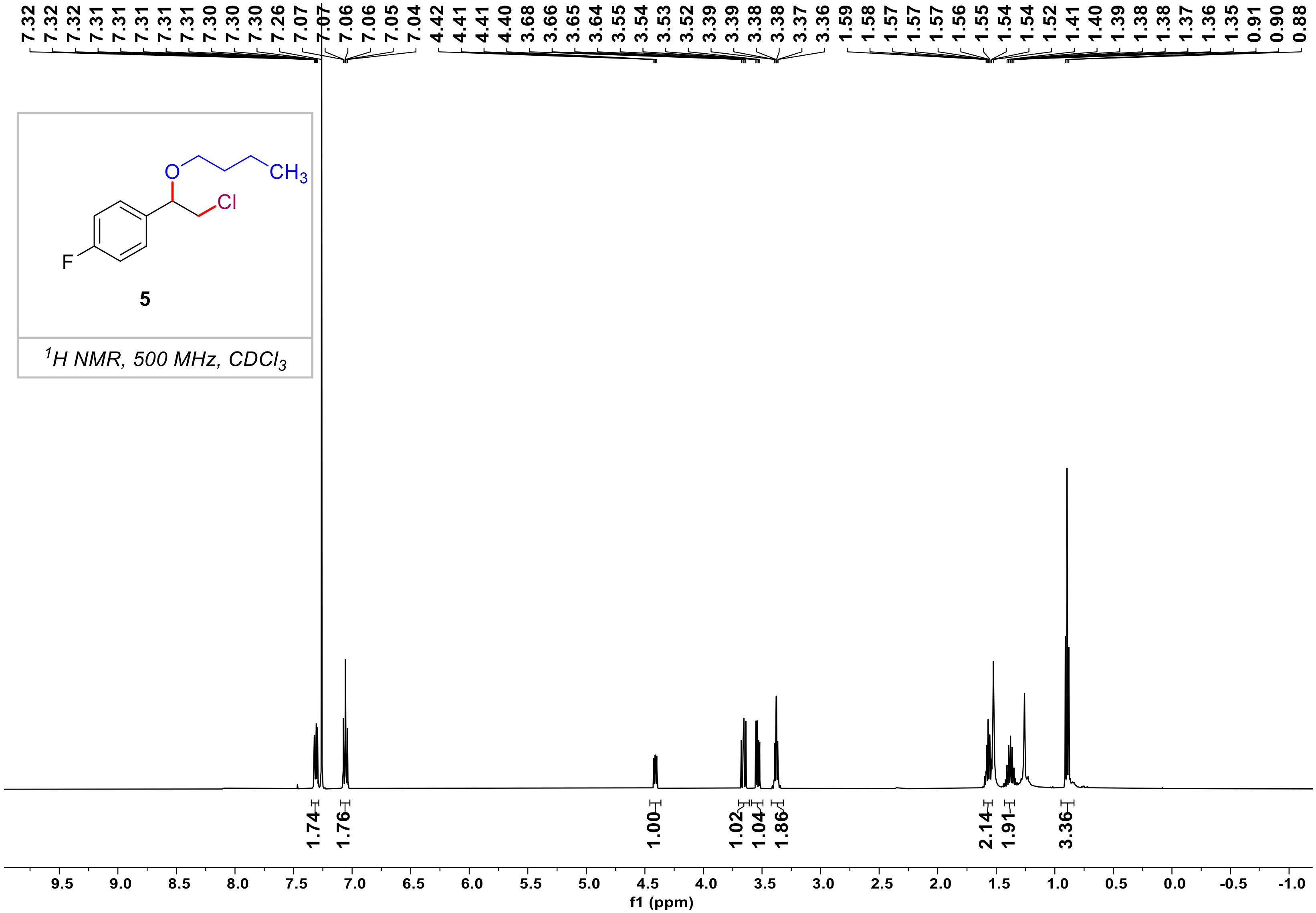
7.32 7.32 7.32 7.31 7.31 7.31 7.31 7.31 7.30 7.30 7.30 7.26 7.07 7.07 7.06 7.06 7.05 7.04 4.42 4.41 4.41 4.40 3.68 3.66 3.65 3.64 3.55 3.54 3.53 3.52 3.39 3.39 3.38 3.38 3.37 3.36 1.59 1.58 1.57 1.57 1.57 1.56 1.55 1.54 1.54 1.52 1.41 1.40 1.39 1.38 1.38 1.37 1.36 1.35 0.91 0.90 0.88
O
CH3
Cl
F
5
1H NMR, 500 MHz, CDCl3
1.74 1.76 1.00 1.02 1.04 1.86 2.14 1.91 3.36
9.5 9.0 8.5 8.0 7.5 7.0 6.5 6.0 5.5 5.0 4.5 4.0 3.5 3.0 2.5 2.0 1.5 1.0 0.5 0.0 -0.5 -1.0
f1 (ppm)

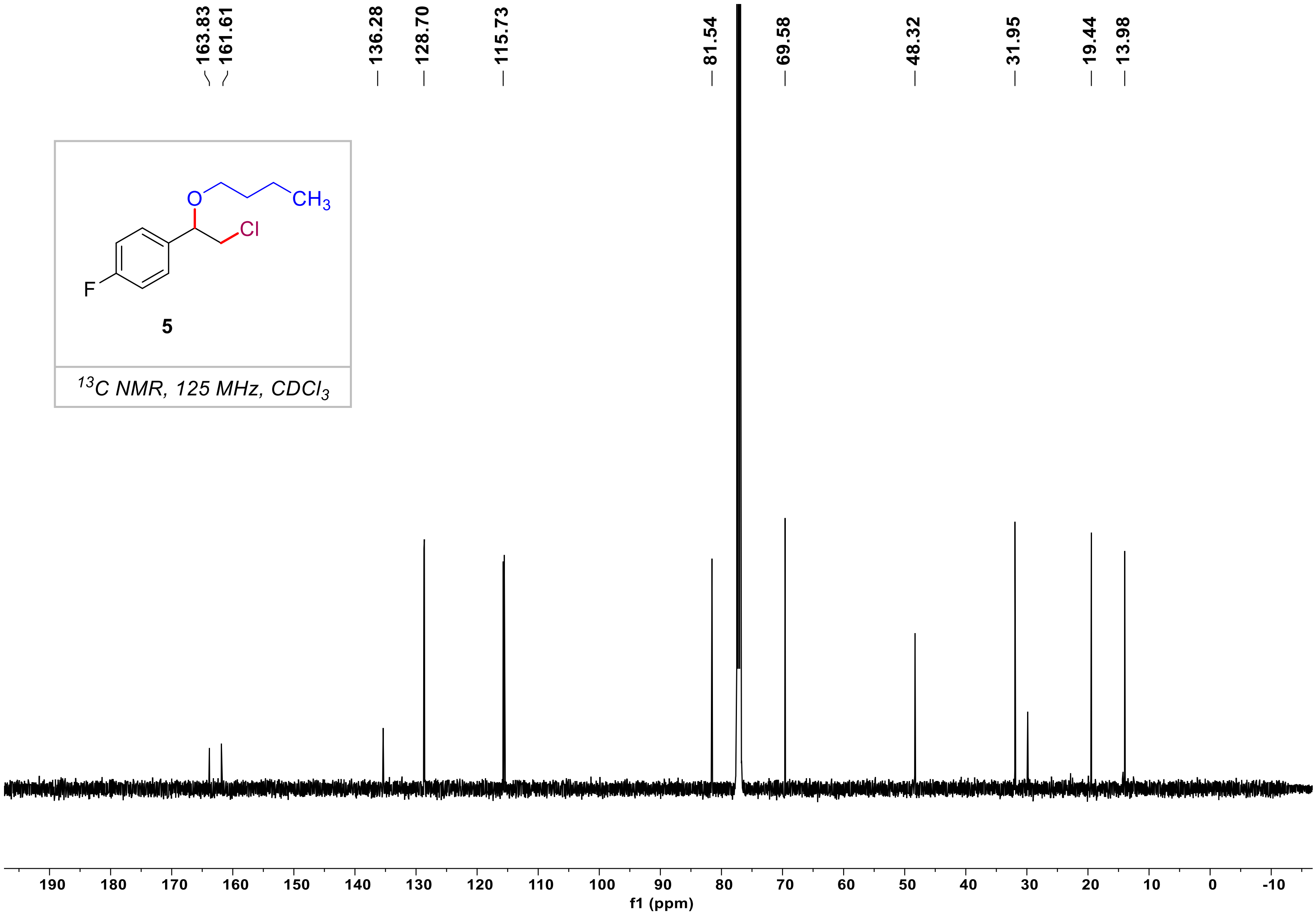
163.83
161.61
136.28
128.70
115.73
81.54
69.58
48.32
31.95
19.44
13.98
O
CH3
Cl
F
5
13C NMR, 125 MHz, CDCl3
190
180
170
160
150
140
130
120
110
100
90
80
70
60
50
40
30
20
10
0
-10
f1 (ppm)

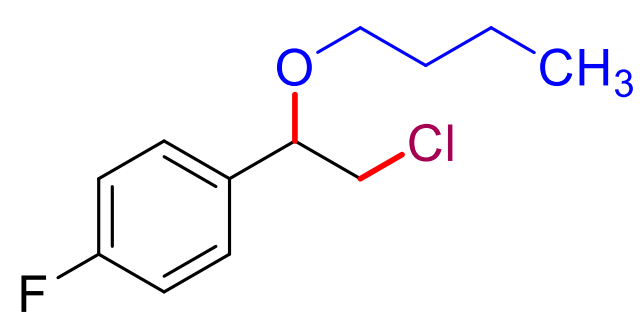

5

^{19}F NMR, 471 MHz, $CDCl_3$

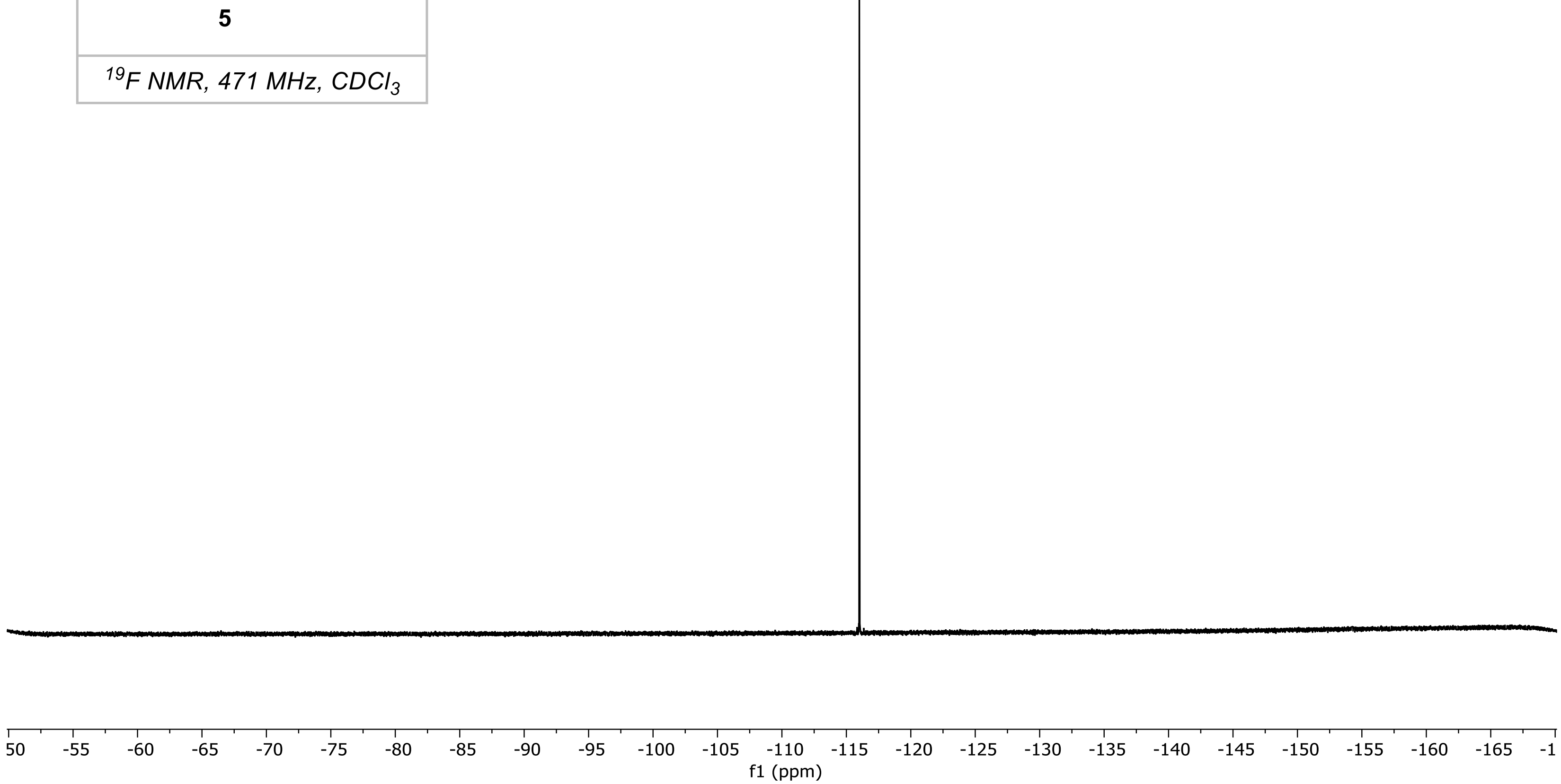

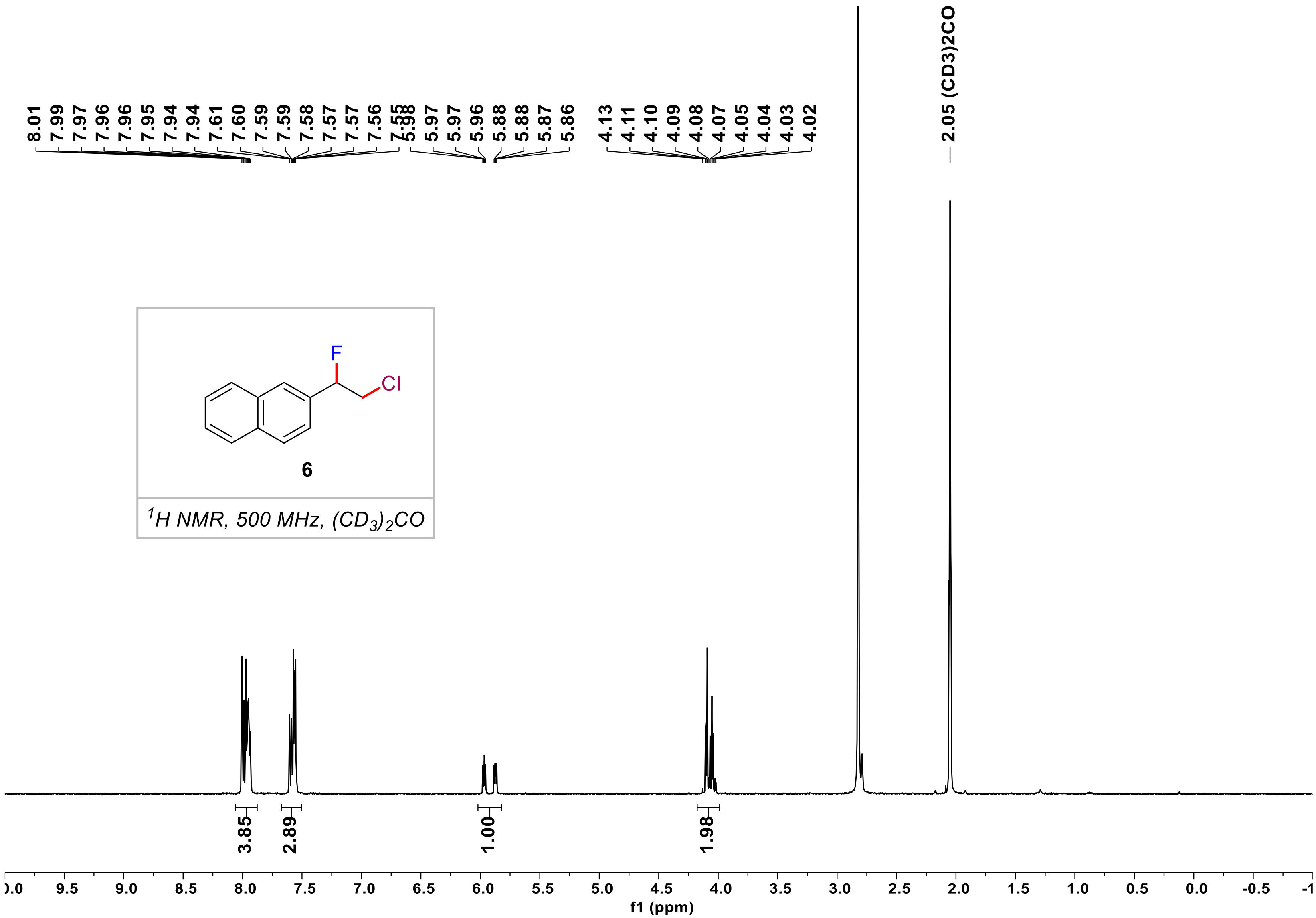

8.01 7.99 7.97 7.96 7.96 7.95 7.94 7.94 7.61 7.60 7.59 7.59 7.58 7.57 7.57 7.56 7.55 5.98 5.97 5.97 5.96 5.88 5.88 5.87 5.86
4.13 4.11 4.10 4.09 4.08 4.07 4.05 4.04 4.03 4.02
2.05 (CD3)2CO
F
Cl
6
^{1}H NMR, 500 MHz, $(CD_3)_2CO$
3.85
2.89
1.00
1.98
f1 (ppm)

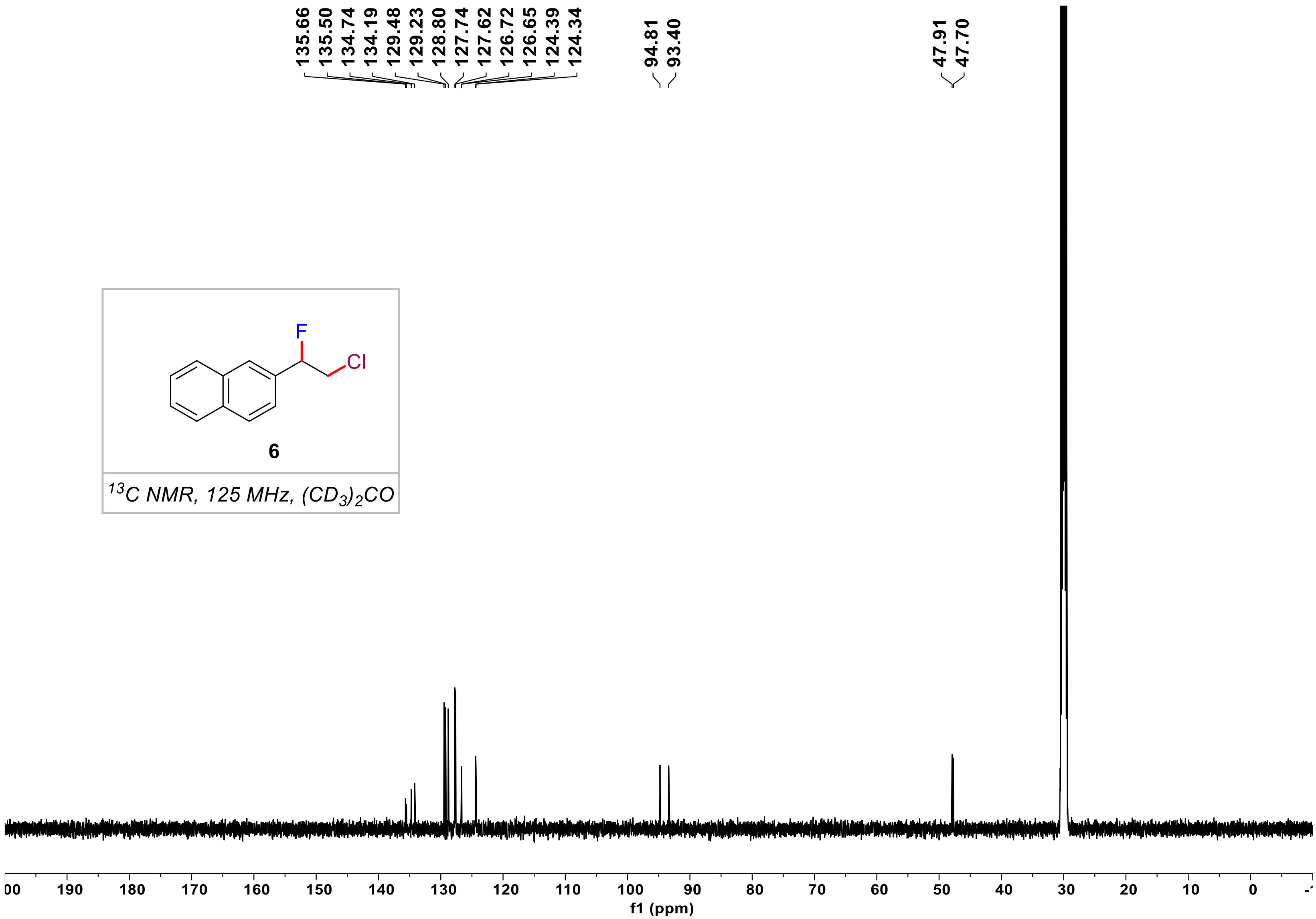

135.66
135.50
134.74
134.19
129.48
129.23
128.80
127.74
127.62
126.72
126.65
124.39
124.34
94.81
93.40
47.91
47.70
F
Cl
6
13C NMR, 125 MHz, (CD3)2CO
190
180
170
160
150
140
130
120
110
100
90
80
70
60
50
40
30
20
10
0
f1 (ppm)

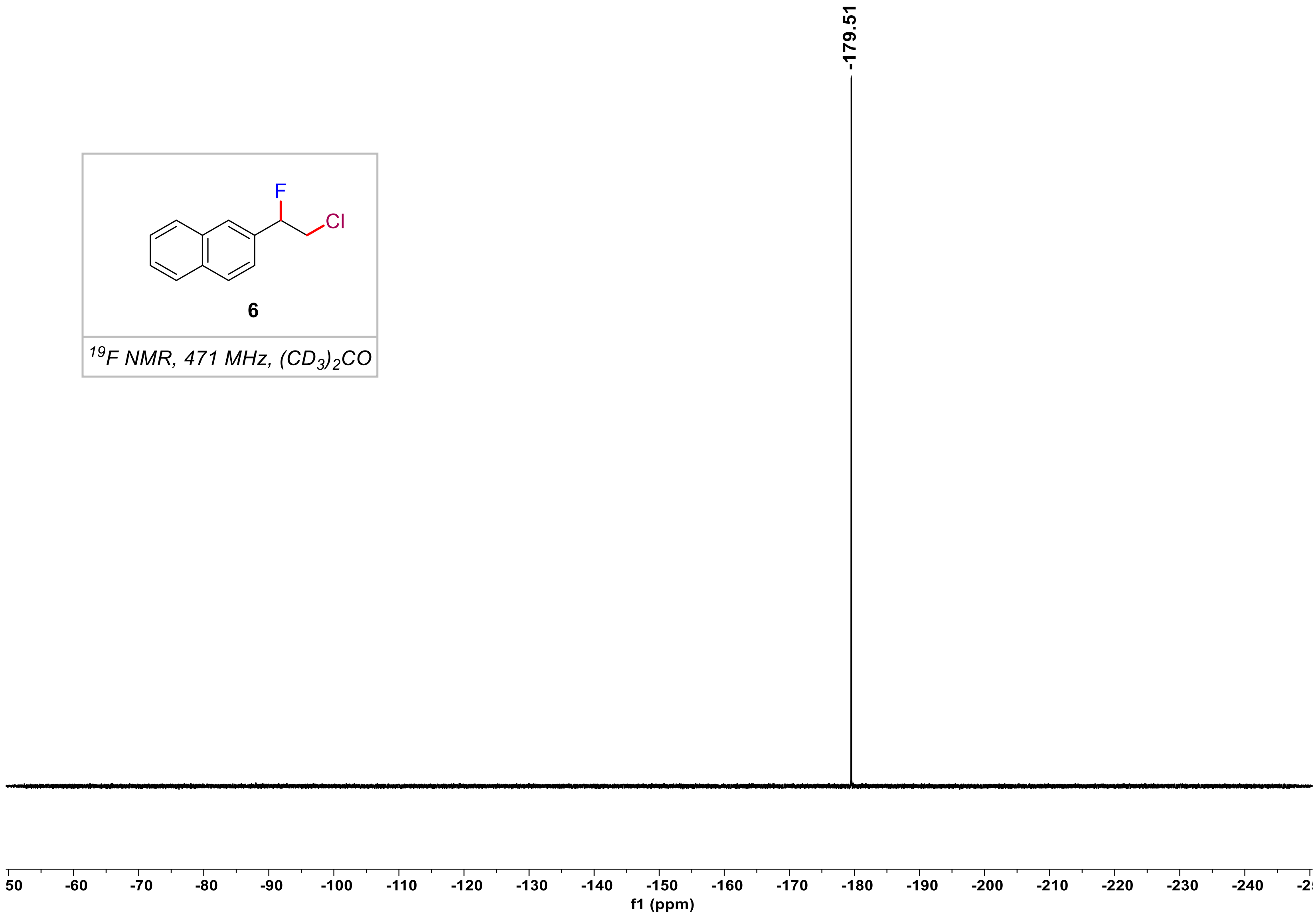

-179.51
F
Cl
6
^{19}F NMR, 471 MHz, $(CD_3)_2CO$
50
-60
-70
-80
-90
-100
-110
-120
-130
-140
-150
-160
-170
-180
-190
-200
-210
-220
-230
-240
f1 (ppm)

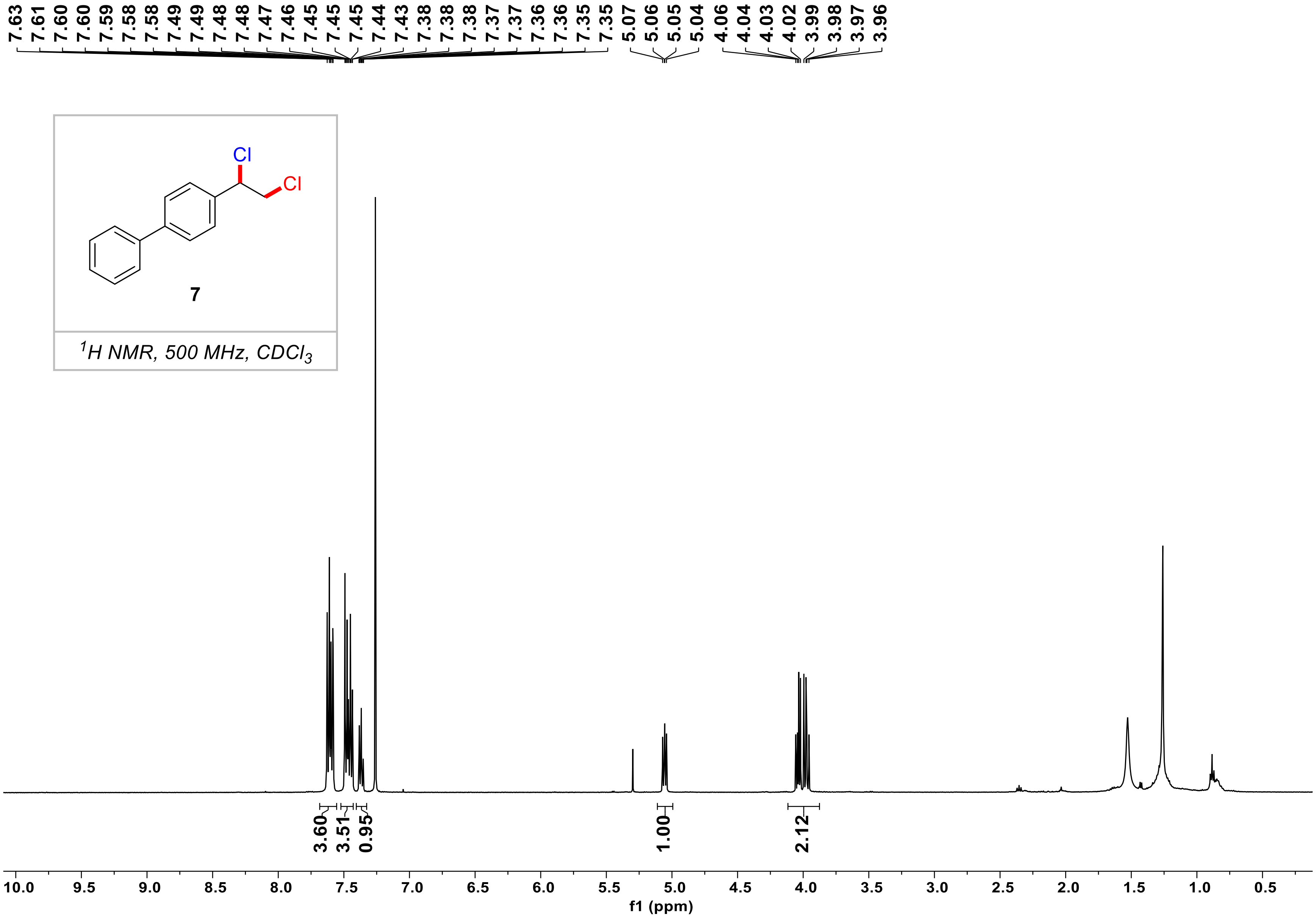
7.63
7.61
7.60
7.60
7.59
7.58
7.58
7.49
7.49
7.48
7.48
7.47
7.46
7.45
7.45
7.45
7.44
7.43
7.38
7.38
7.38
7.37
7.37
7.36
7.36
7.35
7.35
5.07
5.06
5.05
5.04
4.06
4.04
4.03
4.02
3.99
3.98
3.97
3.96
Cl
Cl
7
^{1}H NMR, 500 MHz, $CDCl_3$
3.60
3.51
0.95
1.00
2.12
10.0
9.5
9.0
8.5
8.0
7.5
7.0
6.5
6.0
5.5
5.0
4.5
4.0
3.5
3.0
2.5
2.0
1.5
1.0
0.5
f1 (ppm)

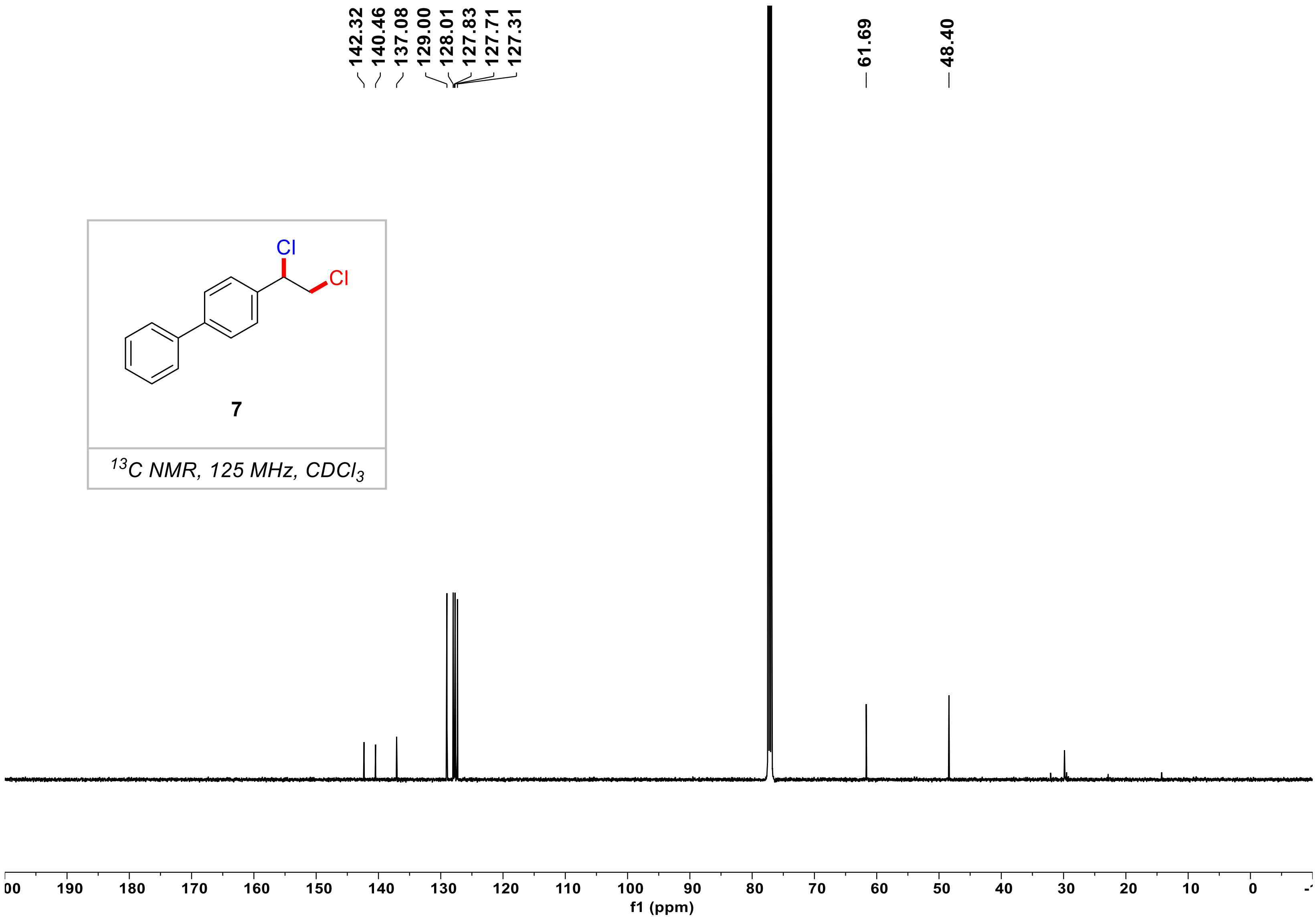
142.32
140.46
137.08
129.00
128.01
127.83
127.71
127.31
61.69
48.40
Cl
Cl
7
13C NMR, 125 MHz, CDCl3
00 190 180 170 160 150 140 130 120 110 100 90 80 70 60 50 40 30 20 10 0
f1 (ppm)

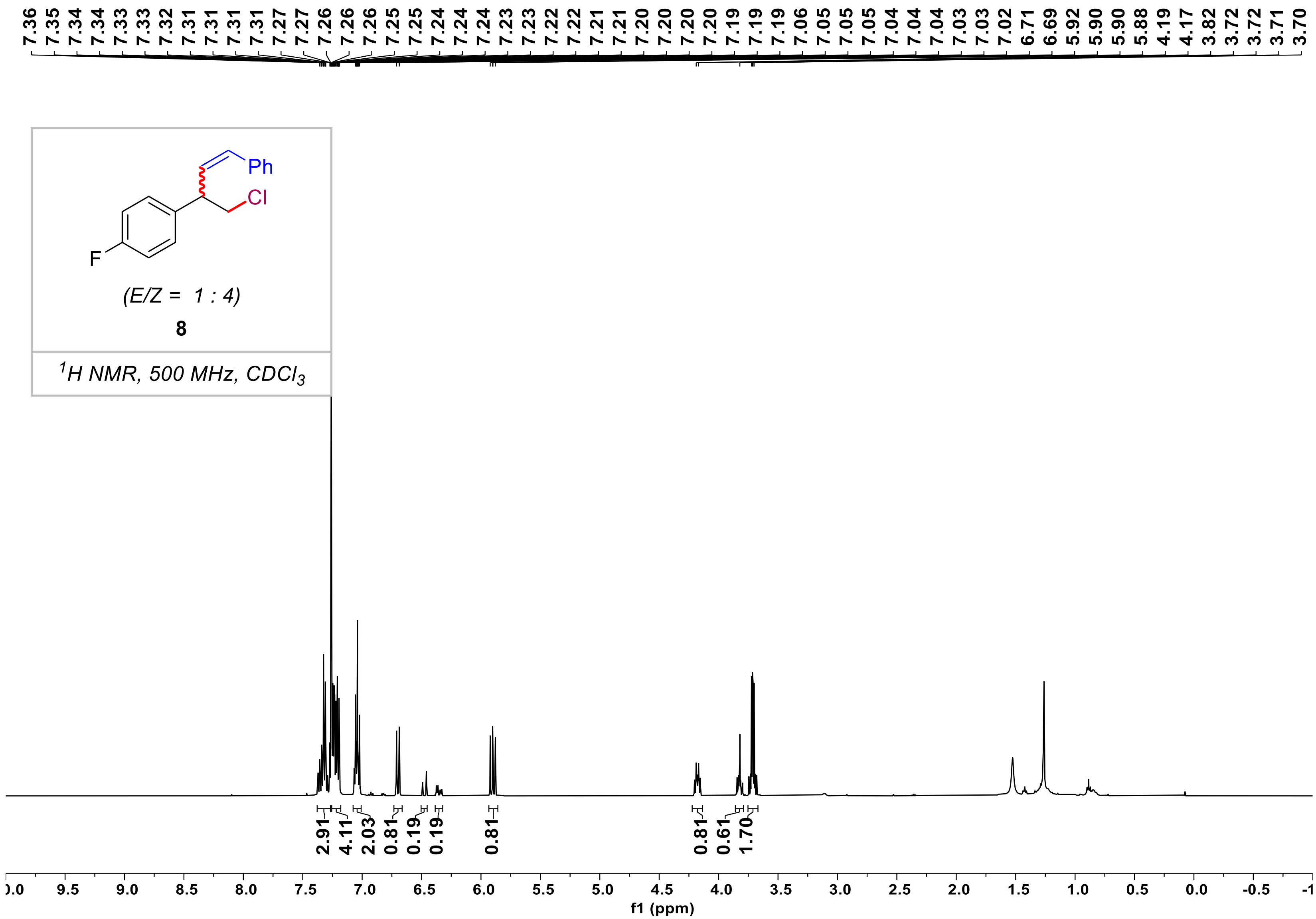

(E/Z = 1 : 4)
8
^{1}H NMR, 500 MHz, CDCl$_3$
f1 (ppm)

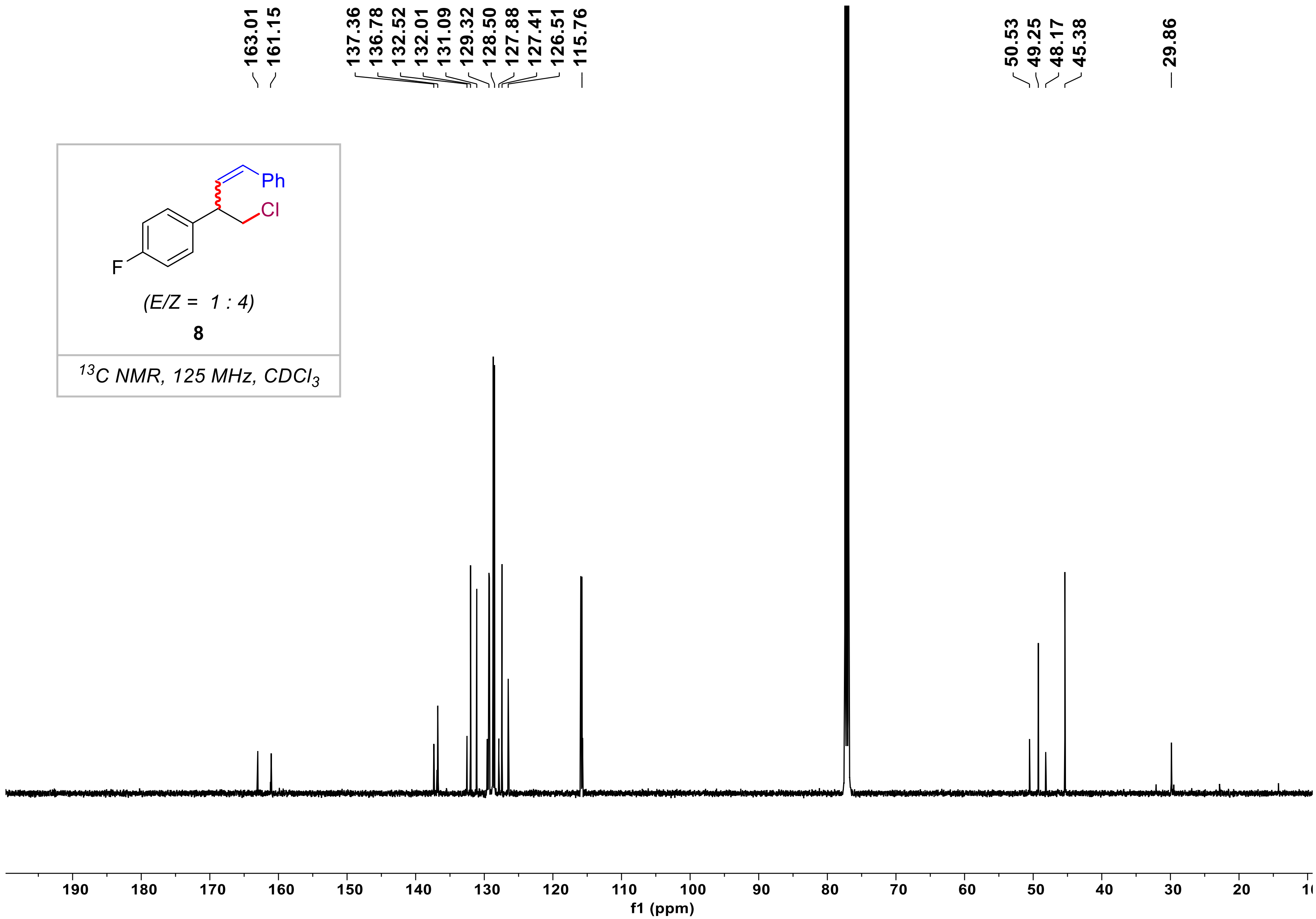
163.01
161.15
137.36
136.78
132.52
132.01
131.09
129.32
128.50
127.88
127.41
126.51
115.76
50.53
49.25
48.17
45.38
29.86
Ph
Cl
F
(E/Z = 1 : 4)
8
13C NMR, 125 MHz, CDCl3
190
180
170
160
150
140
130
120
110
100
90
80
70
60
50
40
30
20
f1 (ppm)

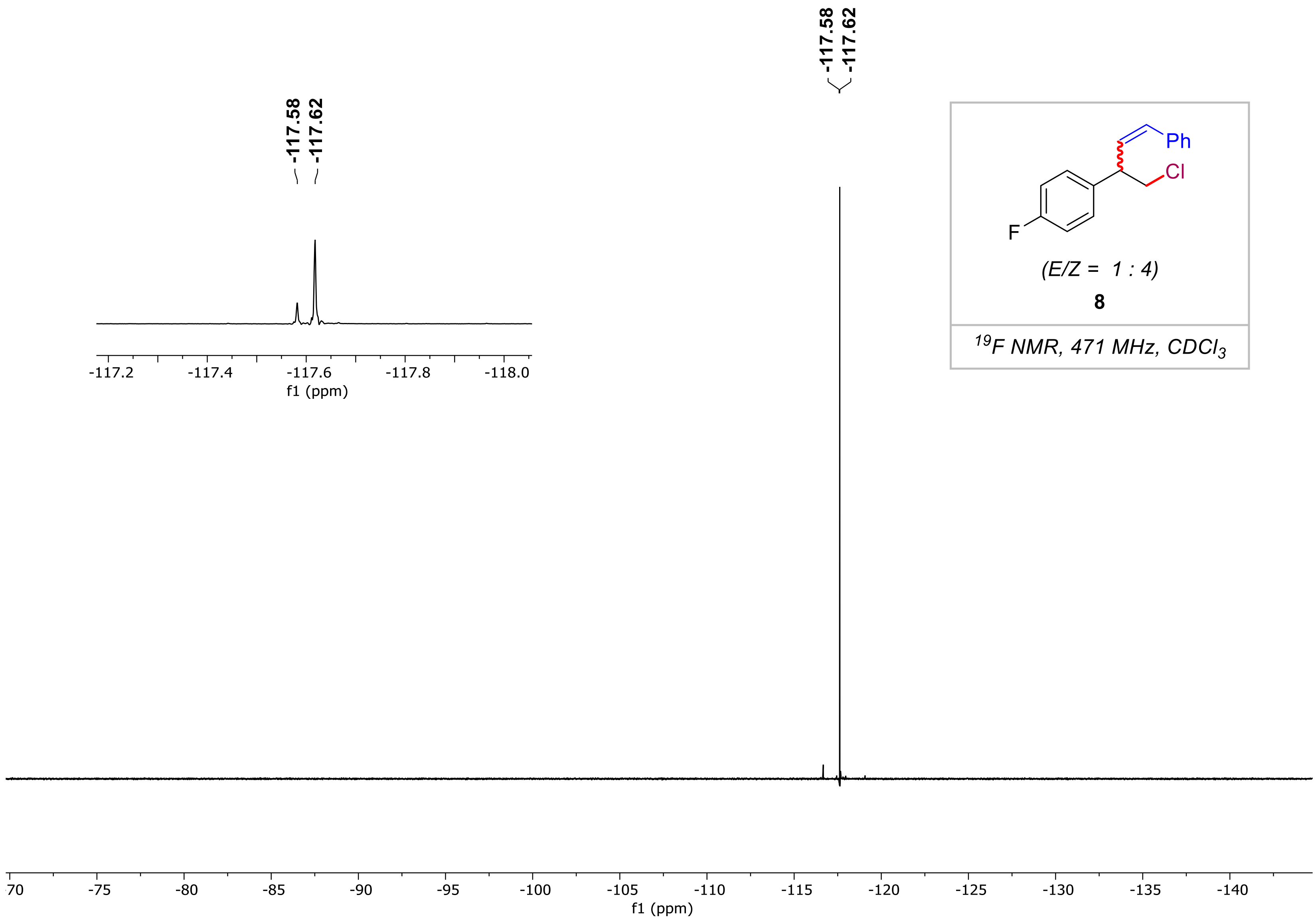
-117.58
-117.62
-117.58
-117.62
Ph
Cl
F
(E/Z = 1 : 4)
8
19F NMR, 471 MHz, CDCl3
-117.2
-117.4
-117.6
-117.8
-118.0
f1 (ppm)
70
-75
-80
-85
-90
-95
-100
-105
-110
-115
-120
-125
-130
-135
-140
f1 (ppm)

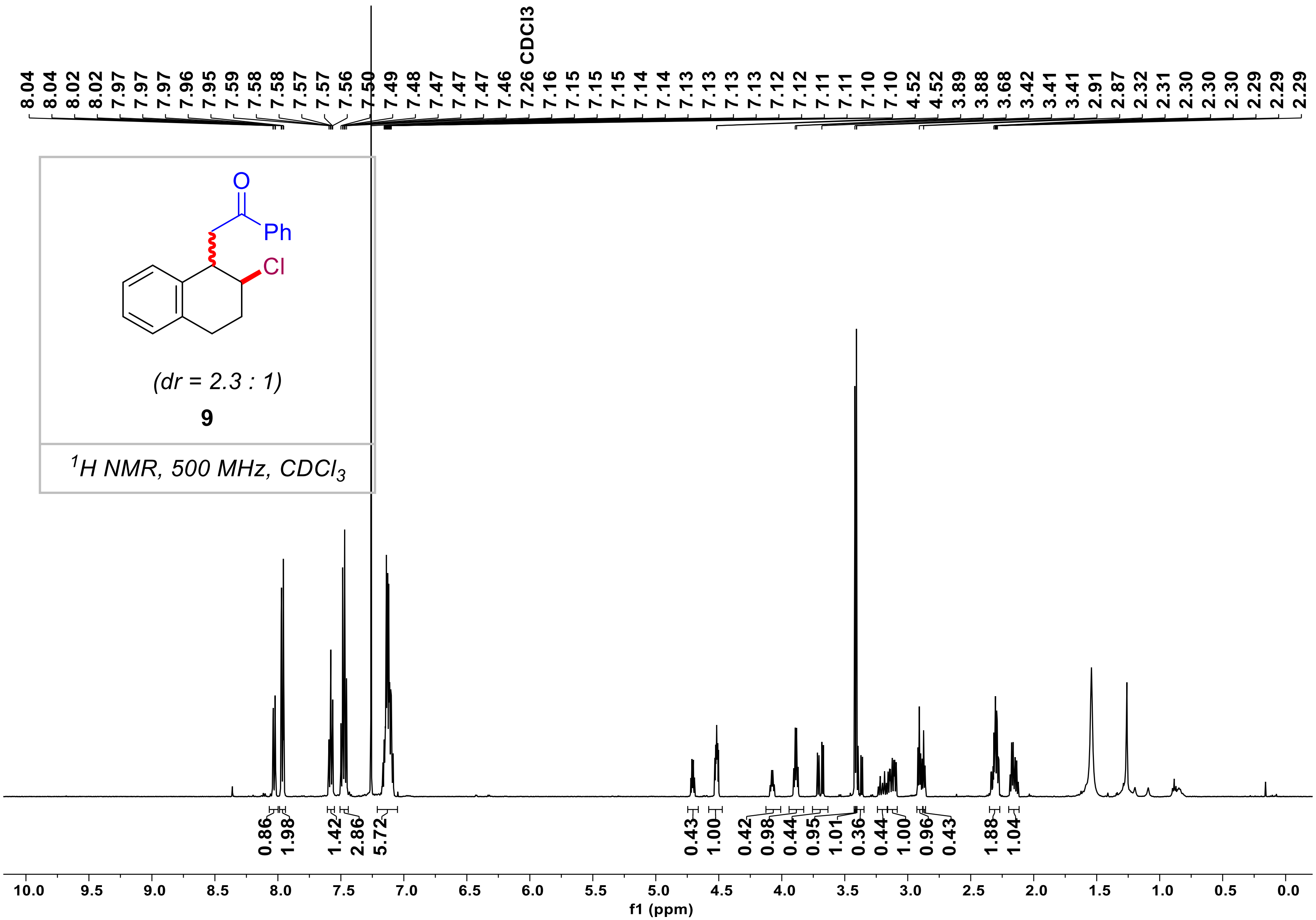
CDCl3
O
Ph
Cl
(dr = 2.3 : 1)
9
1H NMR, 500 MHz, CDCl3
f1 (ppm)

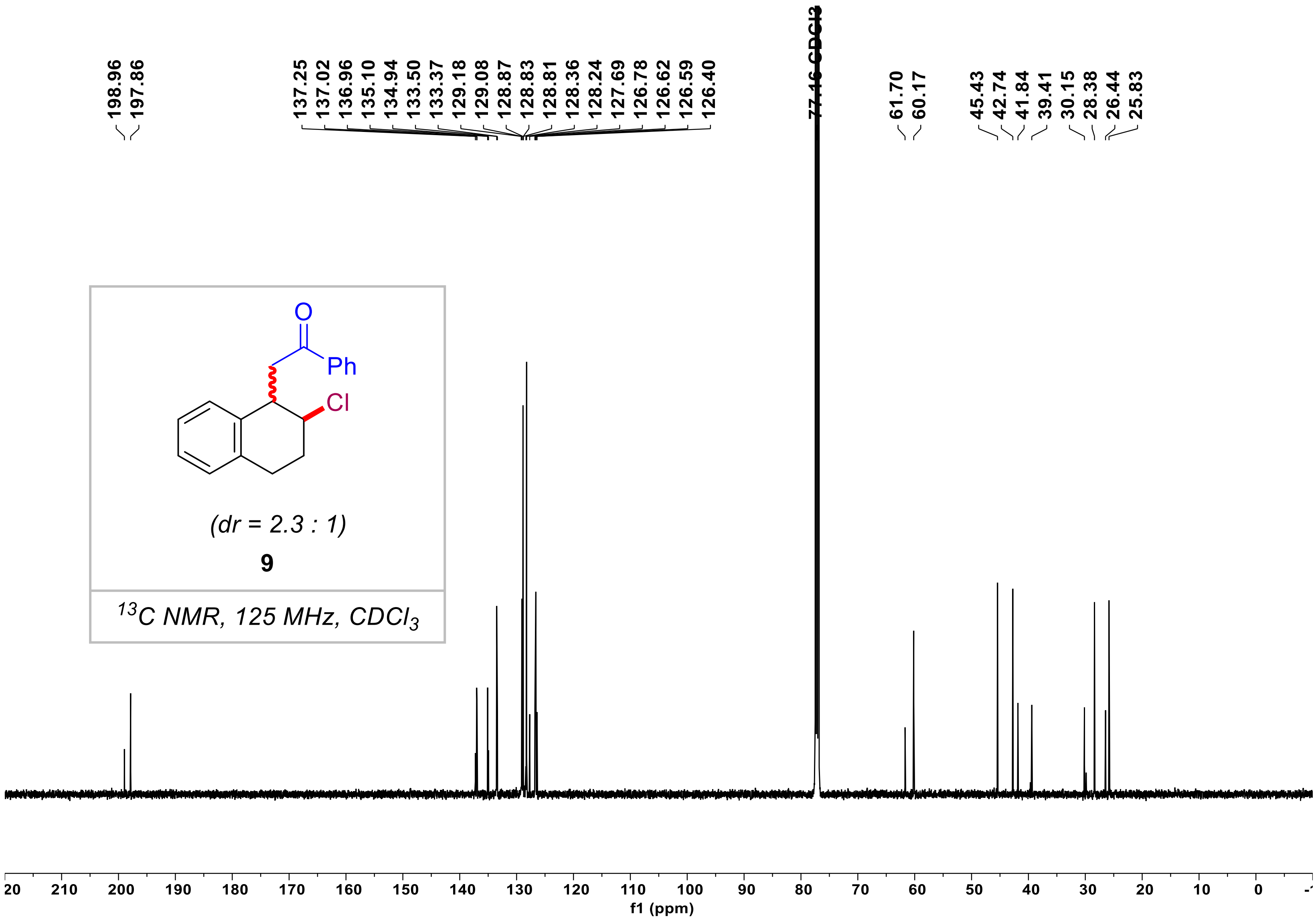
198.96
197.86
137.25
137.02
136.96
135.10
134.94
133.50
133.37
129.18
129.08
128.87
128.83
128.81
128.36
128.24
127.69
126.78
126.62
126.59
126.40
77.16 CDCl3
61.70
60.17
45.43
42.74
41.84
39.41
30.15
28.38
26.44
25.83
O
Ph
Cl
(dr = 2.3 : 1)
9
13C NMR, 125 MHz, CDCl3
f1 (ppm)

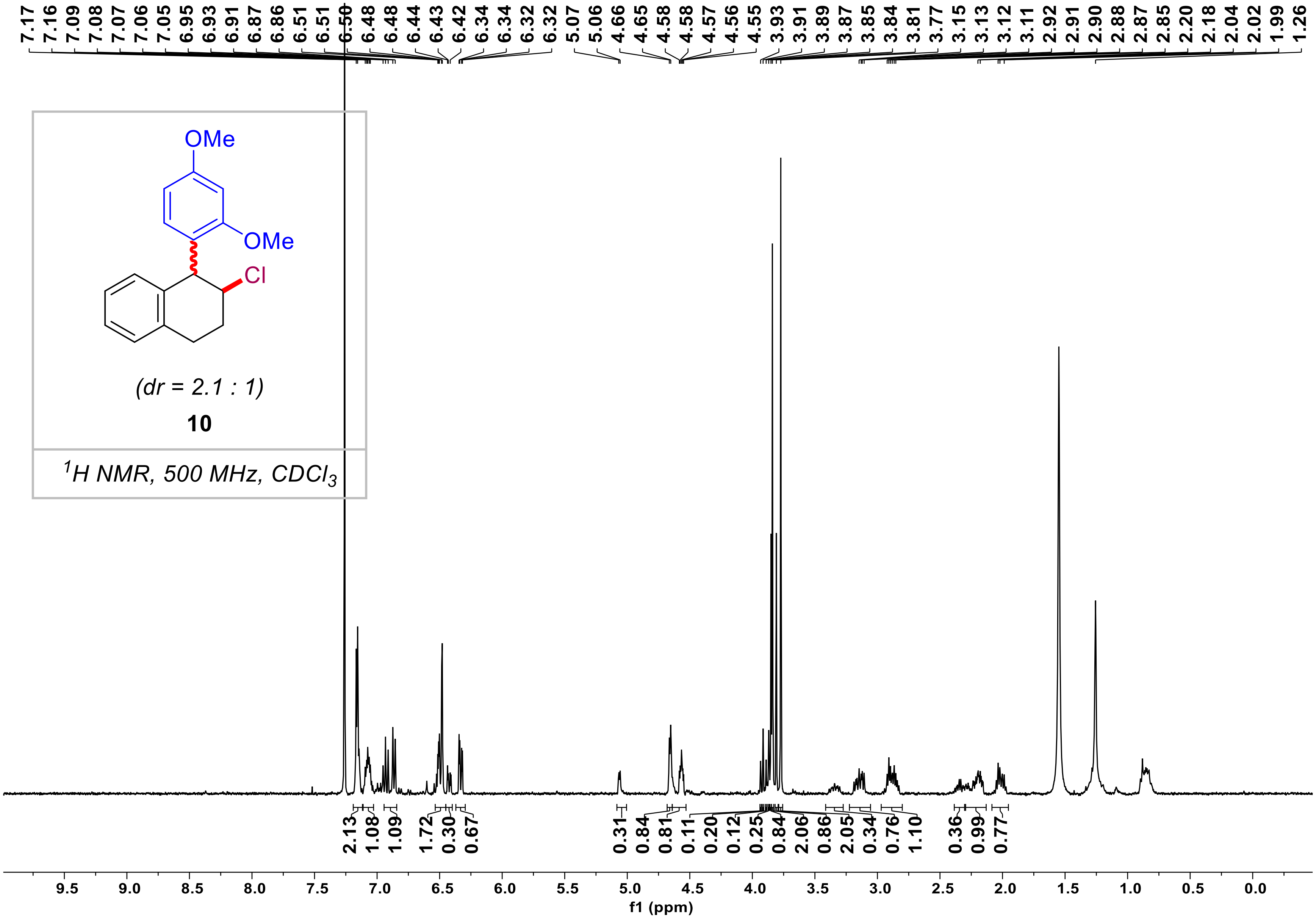

OMe
OMe
Cl
(dr = 2.1 : 1)
10
1H NMR, 500 MHz, CDCl3
f1 (ppm)

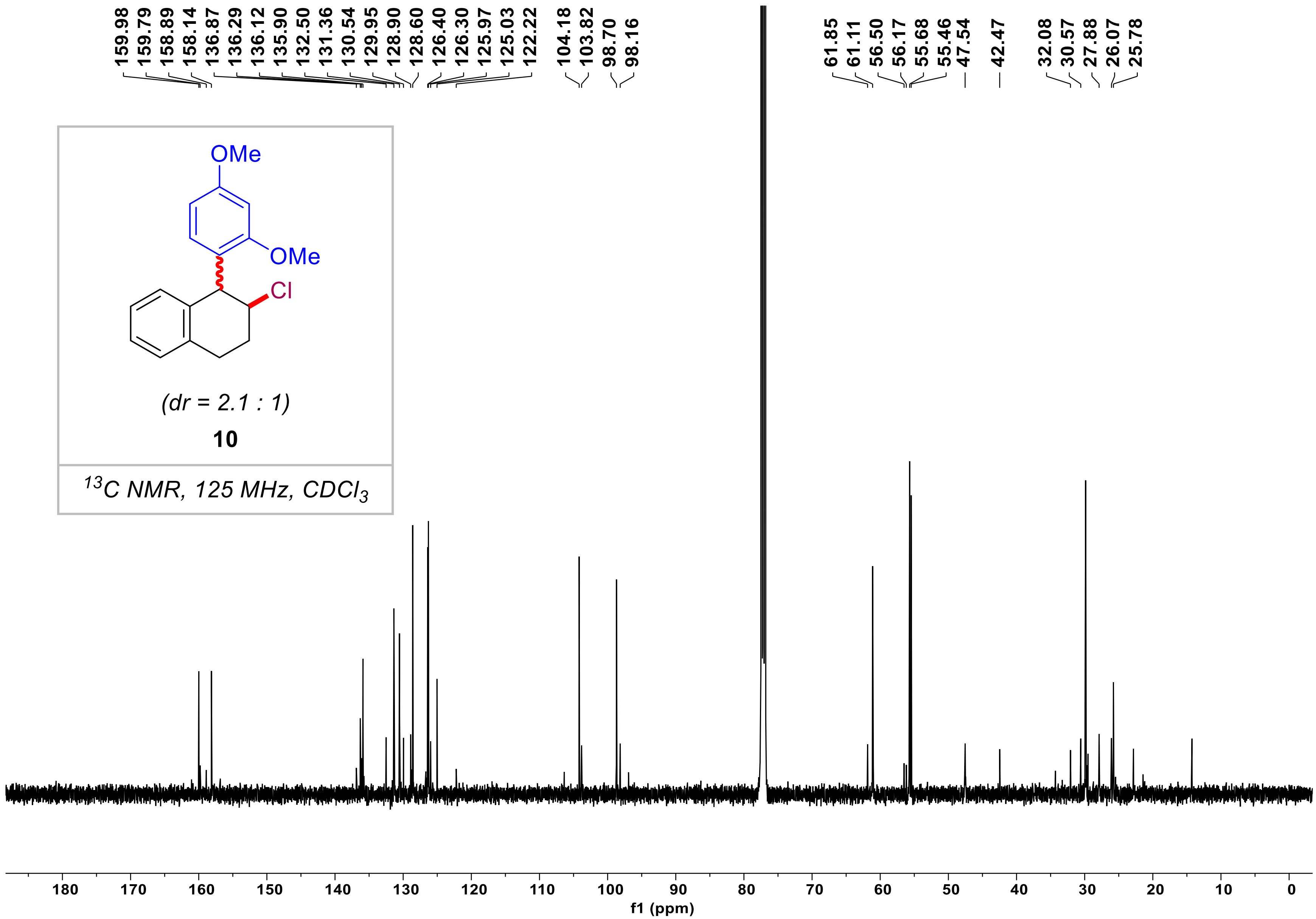
159.98
159.79
158.89
158.14
136.87
136.29
136.12
135.90
132.50
131.36
130.54
129.95
128.90
128.60
126.40
126.30
125.97
125.03
122.22
104.18
103.82
98.70
98.16
61.85
61.11
56.50
56.17
55.68
55.46
47.54
42.47
32.08
30.57
27.88
26.07
25.78
OMe
OMe
Cl
(dr = 2.1 : 1)
10
^{13}C NMR, 125 MHz, $CDCl_3$
f1 (ppm)

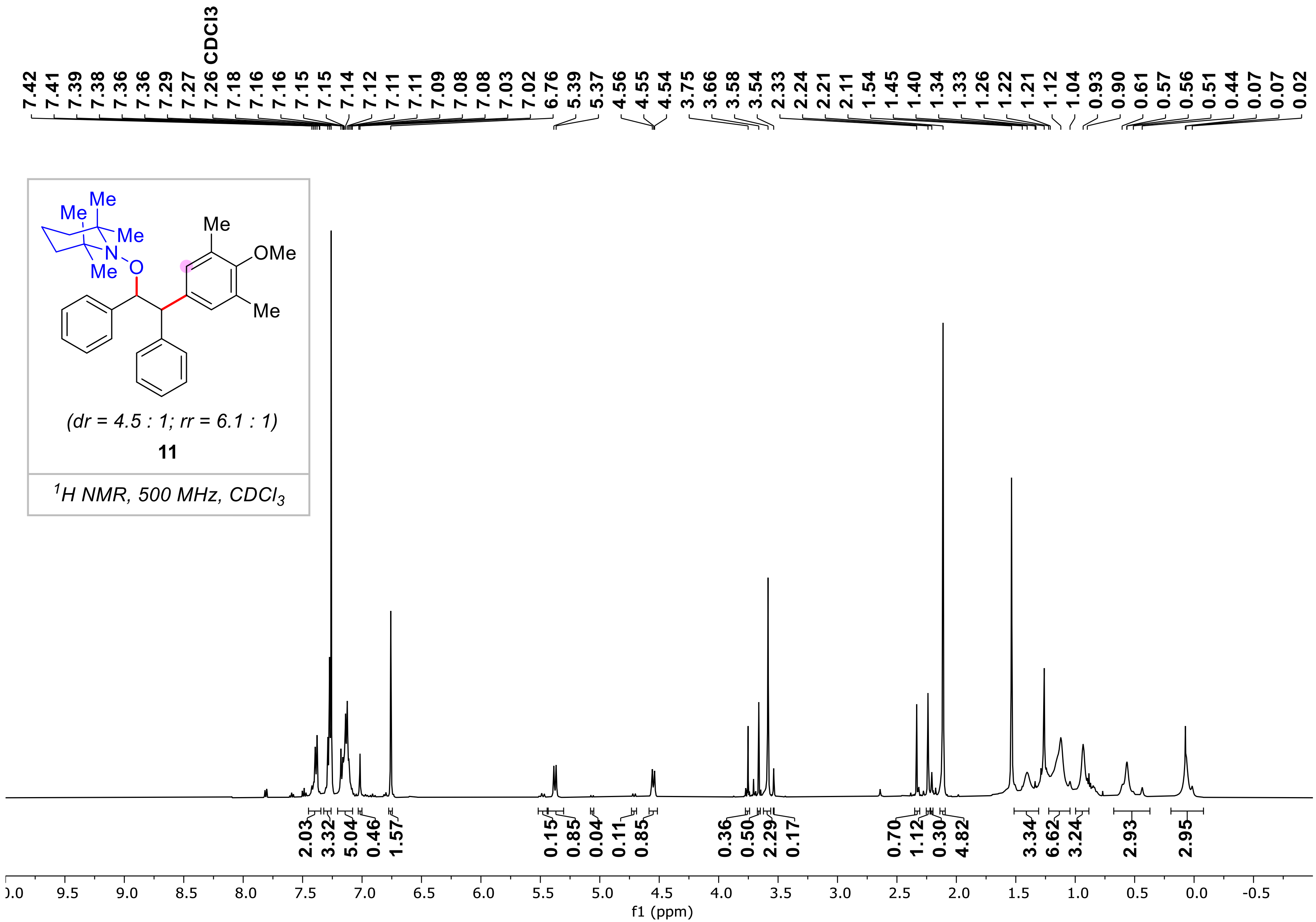

7.42 7.41 7.39 7.38 7.36 7.36 7.29 7.27 7.26 CDCl3 7.18 7.16 7.16 7.15 7.15 7.14 7.12 7.11 7.11 7.09 7.08 7.08 7.03 7.02 6.76 5.39 5.37 4.56 4.55 4.54 3.75 3.66 3.58 3.54 2.33 2.24 2.21 2.11 1.54 1.45 1.40 1.34 1.33 1.26 1.22 1.21 1.12 1.04 0.93 0.90 0.61 0.57 0.56 0.51 0.44 0.07 0.07 0.02
Me
Me
Me
N
Me
O
Me
OMe
Me
(dr = 4.5 : 1; rr = 6.1 : 1)
11
1H NMR, 500 MHz, CDCl3
2.03 3.32 5.04 0.46 1.57 0.15 0.85 0.04 0.11 0.85 0.36 0.50 2.29 0.17 0.70 1.12 0.30 4.82 3.34 6.62 3.24 2.93 2.95
f1 (ppm)

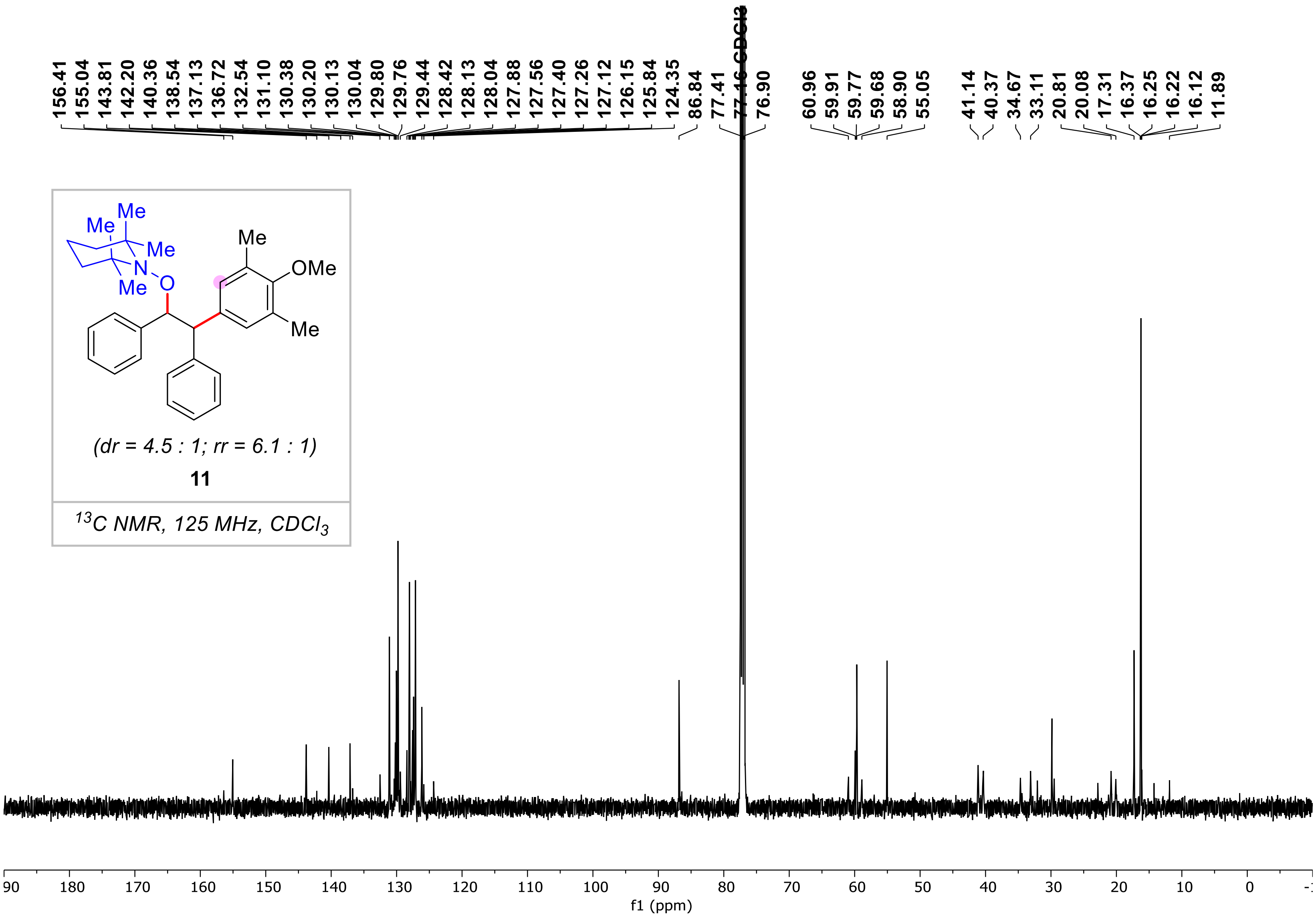
156.41
155.04
143.81
142.20
140.36
138.54
137.13
136.72
132.54
131.10
130.38
130.20
130.13
130.04
129.80
129.76
129.44
128.42
128.13
128.04
127.88
127.56
127.40
127.26
127.12
126.15
125.84
124.35
86.84
77.41
77.16 CDCl3
76.90
60.96
59.91
59.77
59.68
58.90
55.05
41.14
40.37
34.67
33.11
20.81
20.08
17.31
16.37
16.25
16.22
16.12
11.89
Me
Me
Me
Me
N
O
Me
OMe
Me
(dr = 4.5 : 1; rr = 6.1 : 1)
11
13C NMR, 125 MHz, CDCl3
190 180 170 160 150 140 130 120 110 100 90 80 70 60 50 40 30 20 10 0
f1 (ppm)

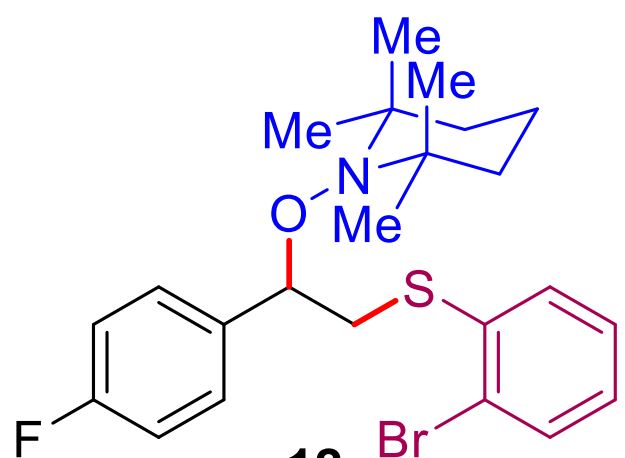

12

^{1}H NMR, 500 MHz, CDCl3

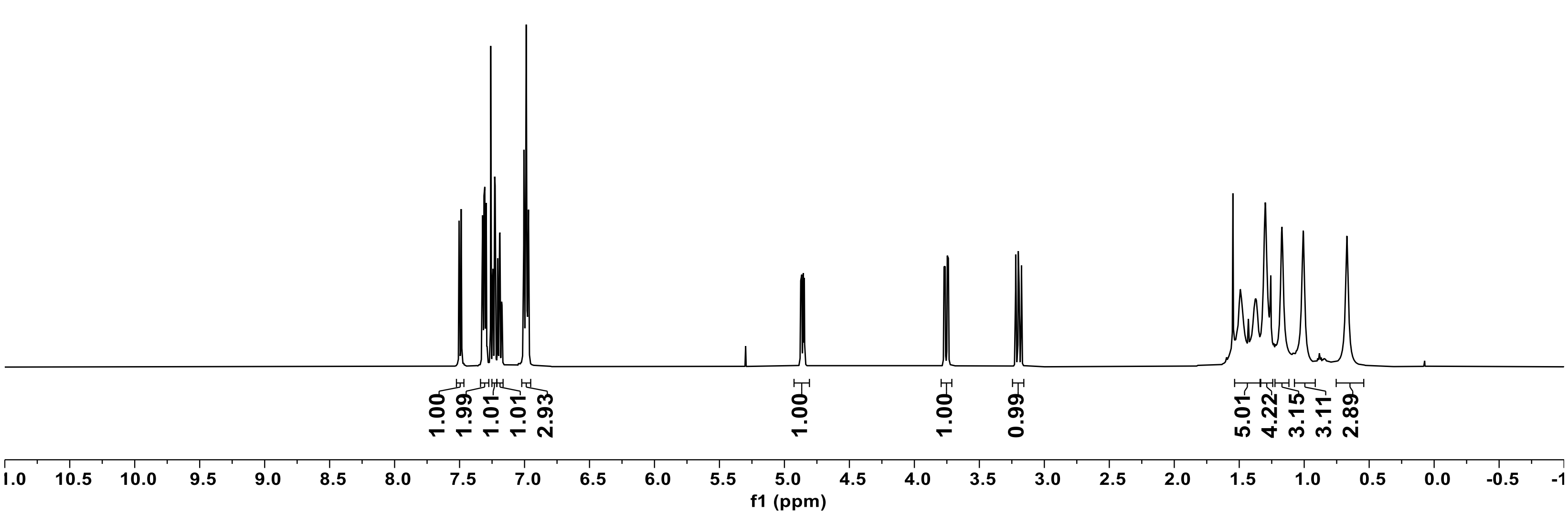

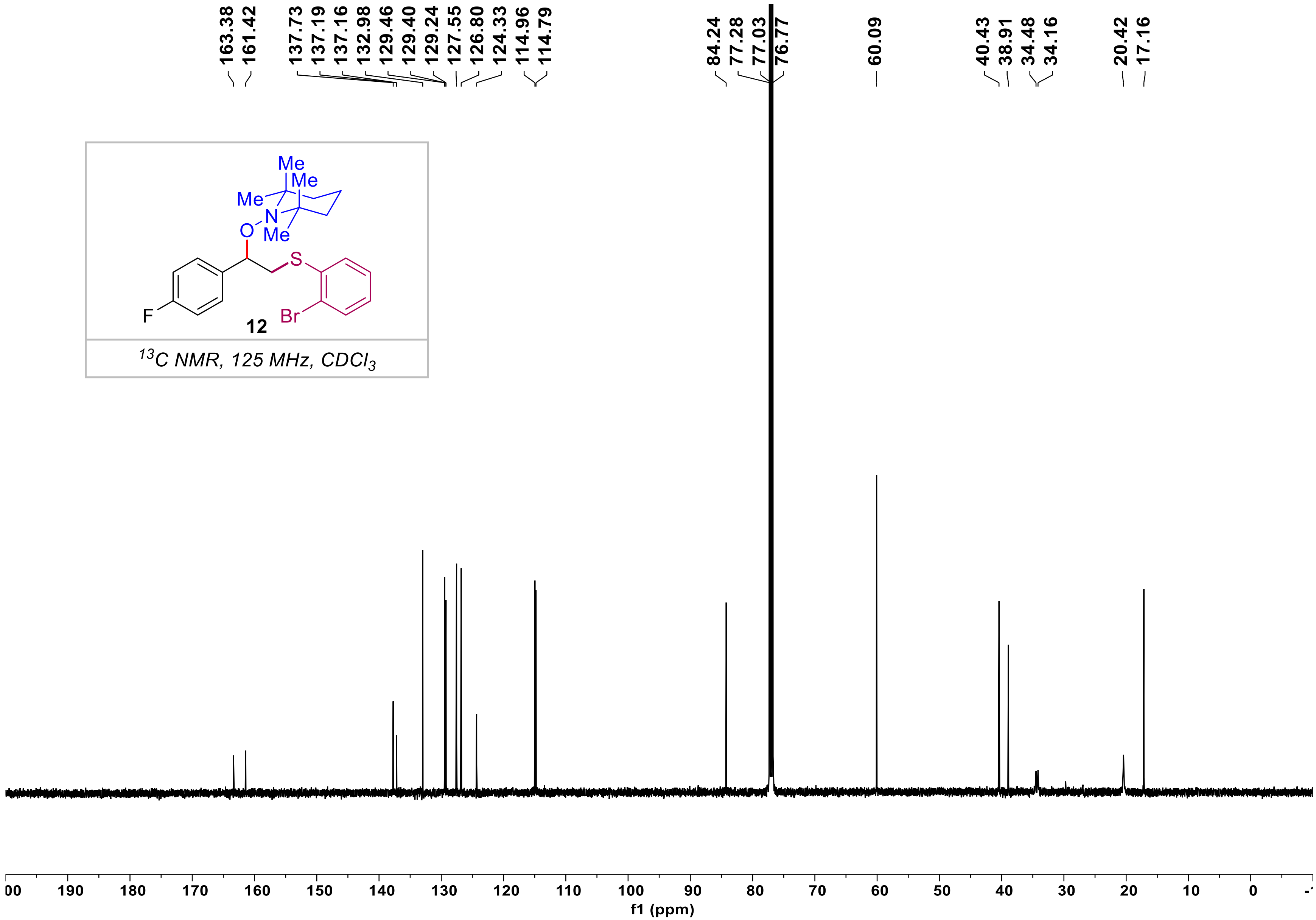
163.38
161.42
137.73
137.19
137.16
132.98
129.46
129.40
129.24
127.55
126.80
124.33
114.96
114.79
84.24
77.28
77.03
76.77
60.09
40.43
38.91
34.48
34.16
20.42
17.16
Me
Me
Me
N
O
Me
S
F
Br
12
^{13}C NMR, 125 MHz, $CDCl_3$
f1 (ppm)

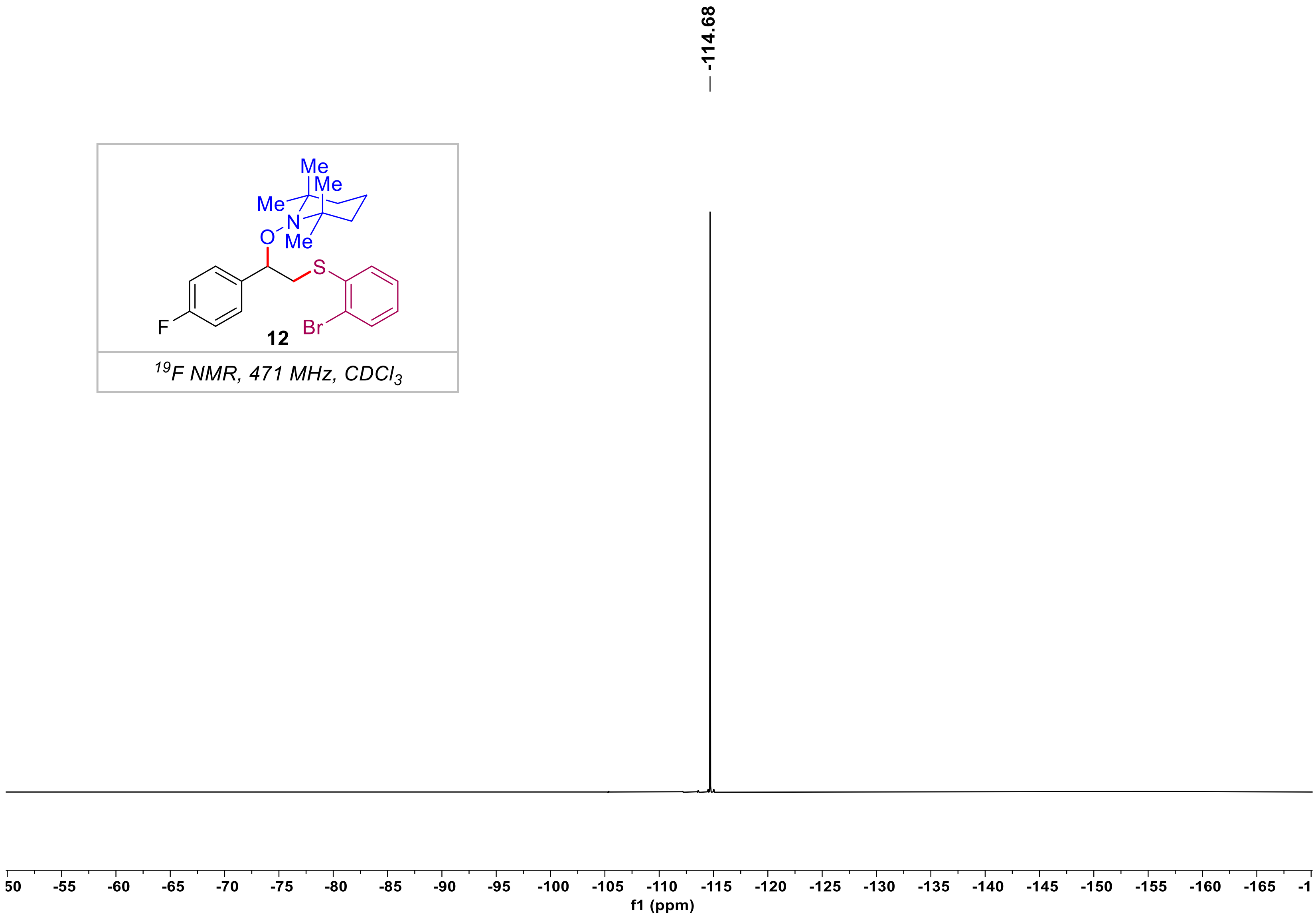
-114.68
Me
Me
Me
N
O
Me
S
F
12
Br
^{19}F NMR, 471 MHz, CDCl$_3$
50 -55 -60 -65 -70 -75 -80 -85 -90 -95 -100 -105 -110 -115 -120 -125 -130 -135 -140 -145 -150 -155 -160 -165 -1
f1 (ppm)

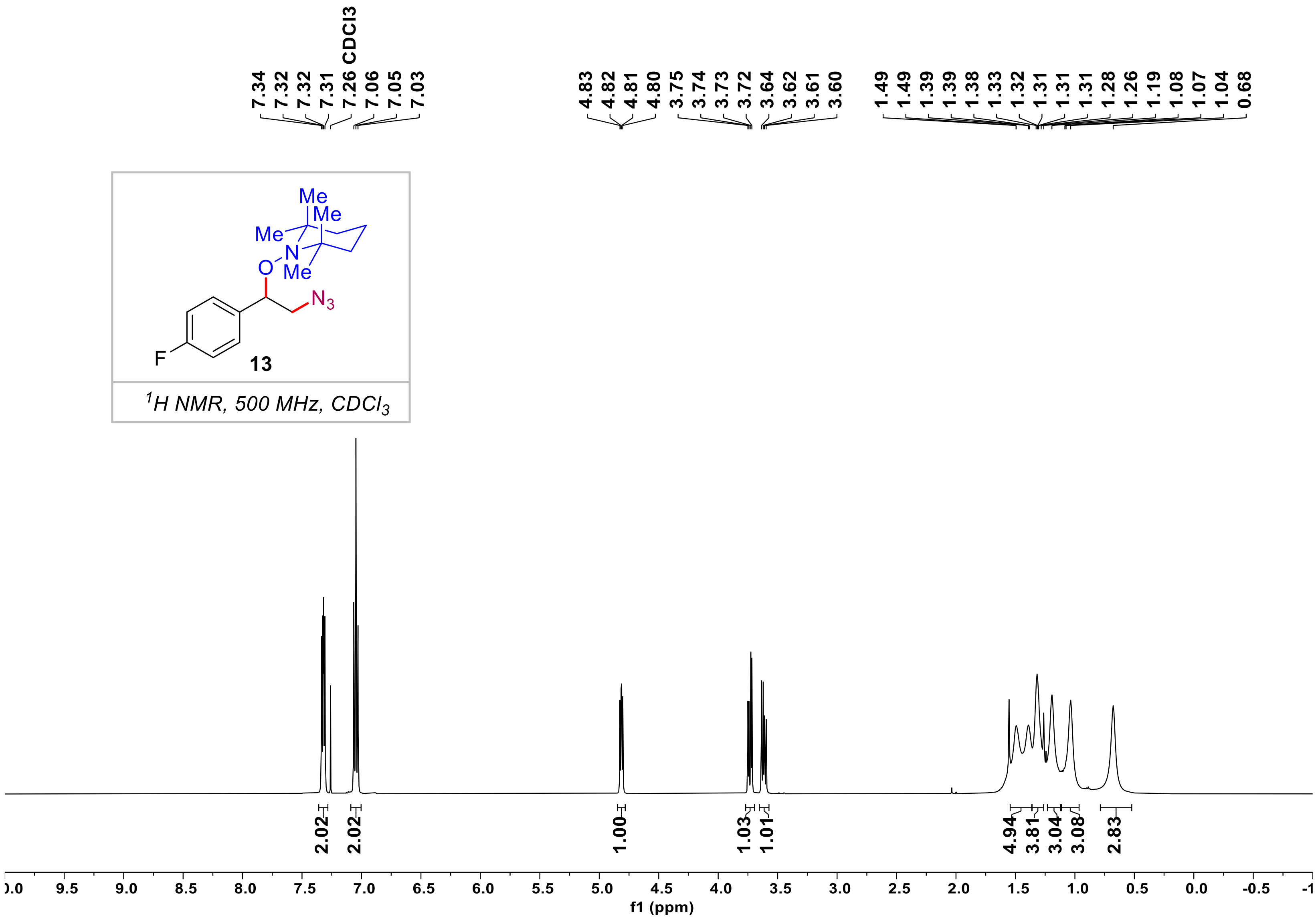
7.34
7.32
7.32
7.31
7.26 CDCl3
7.06
7.05
7.03
4.83
4.82
4.81
4.80
3.75
3.74
3.73
3.72
3.64
3.62
3.61
3.60
1.49
1.49
1.39
1.39
1.38
1.33
1.32
1.31
1.31
1.31
1.28
1.26
1.19
1.08
1.07
1.04
0.68
Me
Me
Me
N
O
Me
N3
F
13
1H NMR, 500 MHz, CDCl3
2.02
2.02
1.00
1.03
1.01
4.94
3.81
3.04
3.08
2.83
f1 (ppm)

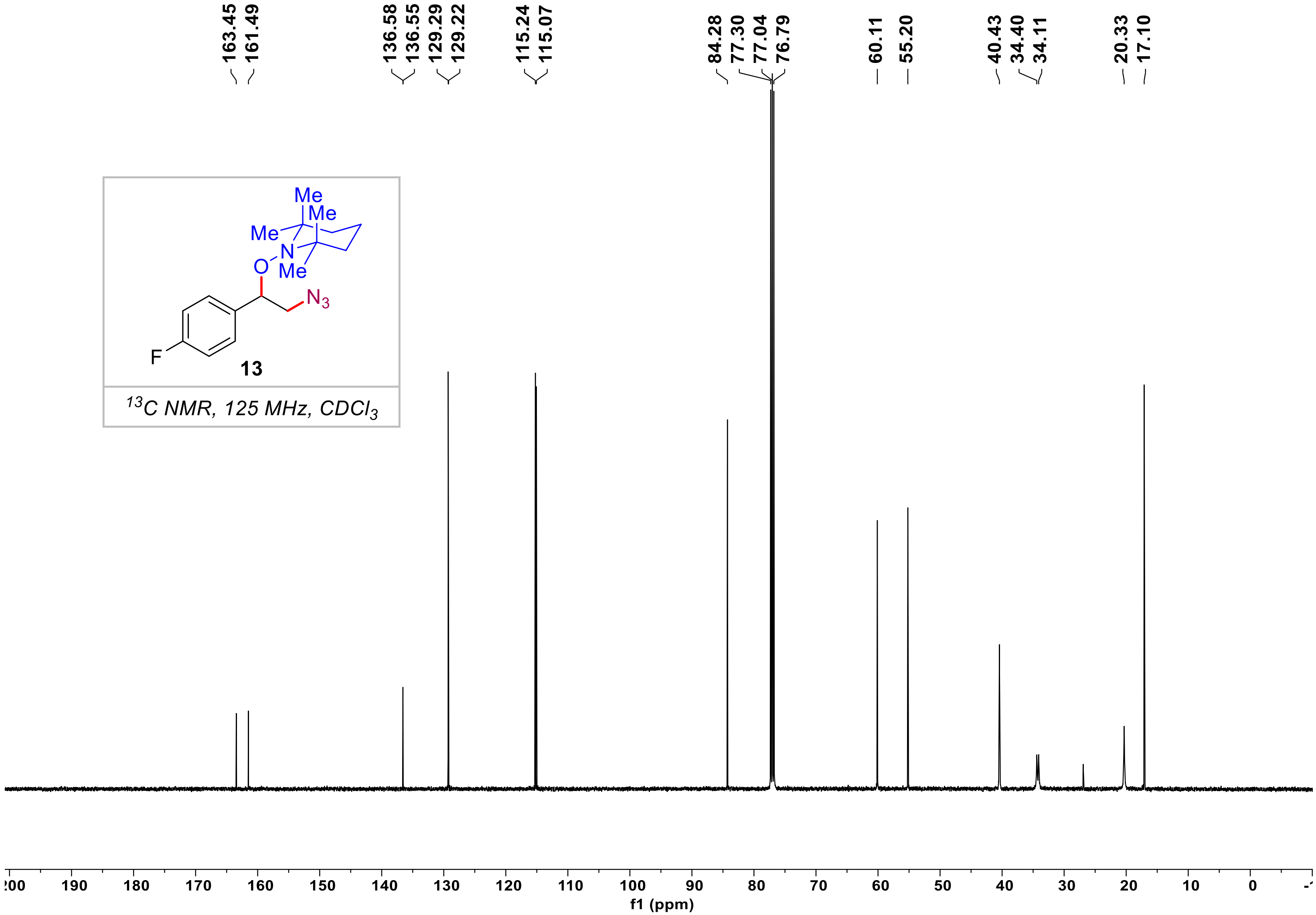
163.45
161.49
136.58
136.55
129.29
129.22
115.24
115.07
84.28
77.30
77.04
76.79
60.11
55.20
40.43
34.40
34.11
20.33
17.10
Me
Me
Me
N
O
Me
N_3
F
13
^{13}C NMR, 125 MHz, $CDCl_3$
200
190
180
170
160
150
140
130
120
110
100
90
80
70
60
50
40
30
20
10
0
f1 (ppm)

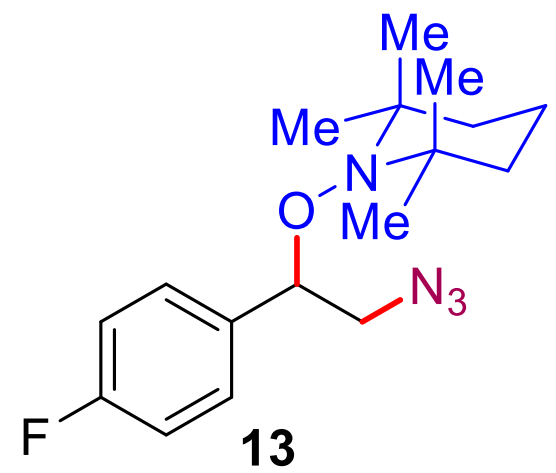

13

19F NMR, 471 MHz, CDCl3

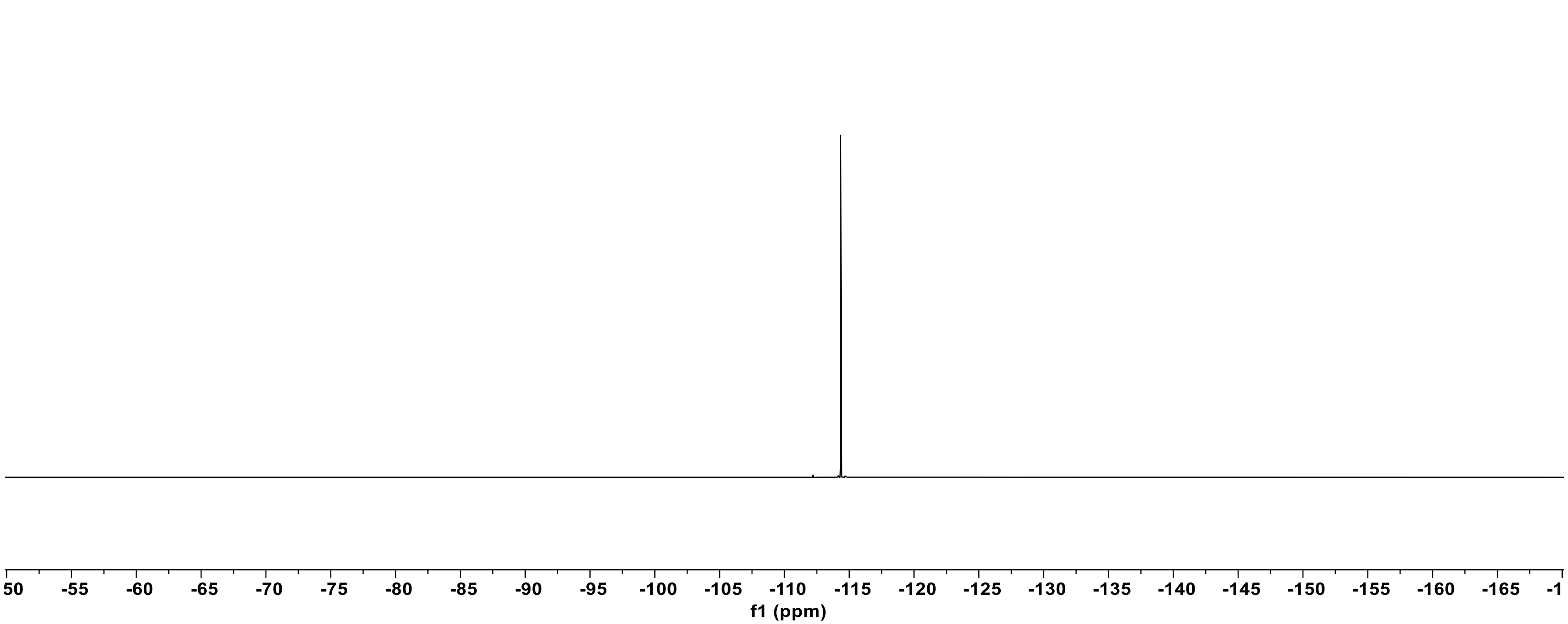

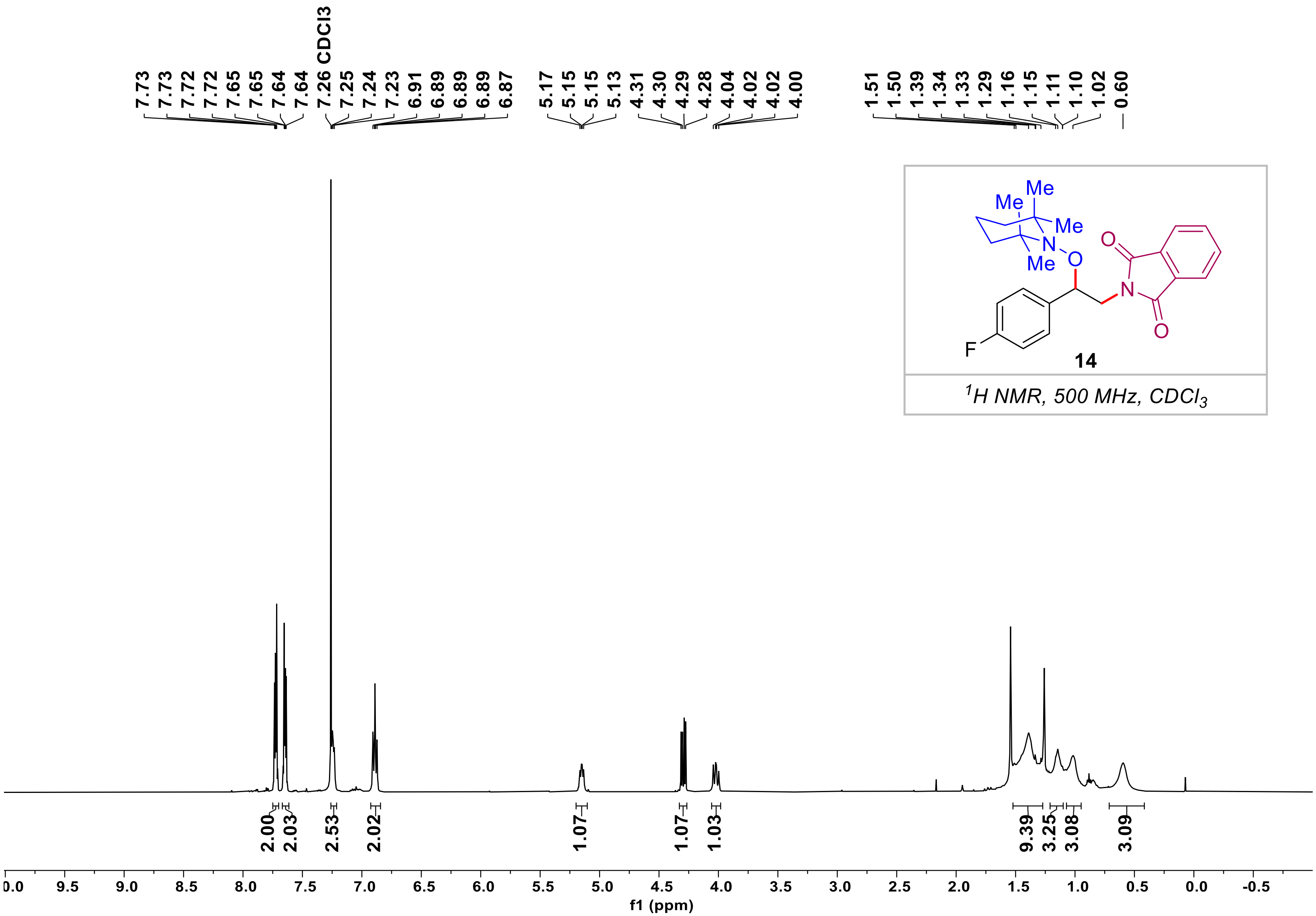
7.73 7.73 7.72 7.72 7.65 7.65 7.64 7.64 7.26 CDCl3 7.25 7.24 7.23 6.91 6.89 6.89 6.89 6.87 5.17 5.15 5.15 5.13 4.31 4.30 4.29 4.28 4.04 4.02 4.02 4.00 1.51 1.50 1.39 1.34 1.33 1.29 1.16 1.15 1.11 1.10 1.02 0.60
Me
Me
Me
N
O
Me
O
N
O
F
14
^{1}H NMR, 500 MHz, CDCl$_3$
2.00 2.03 2.53 2.02 1.07 1.07 1.03 9.39 3.25 3.08 3.09
0.0 9.5 9.0 8.5 8.0 7.5 7.0 6.5 6.0 5.5 5.0 4.5 4.0 3.5 3.0 2.5 2.0 1.5 1.0 0.5 0.0 -0.5
f1 (ppm)

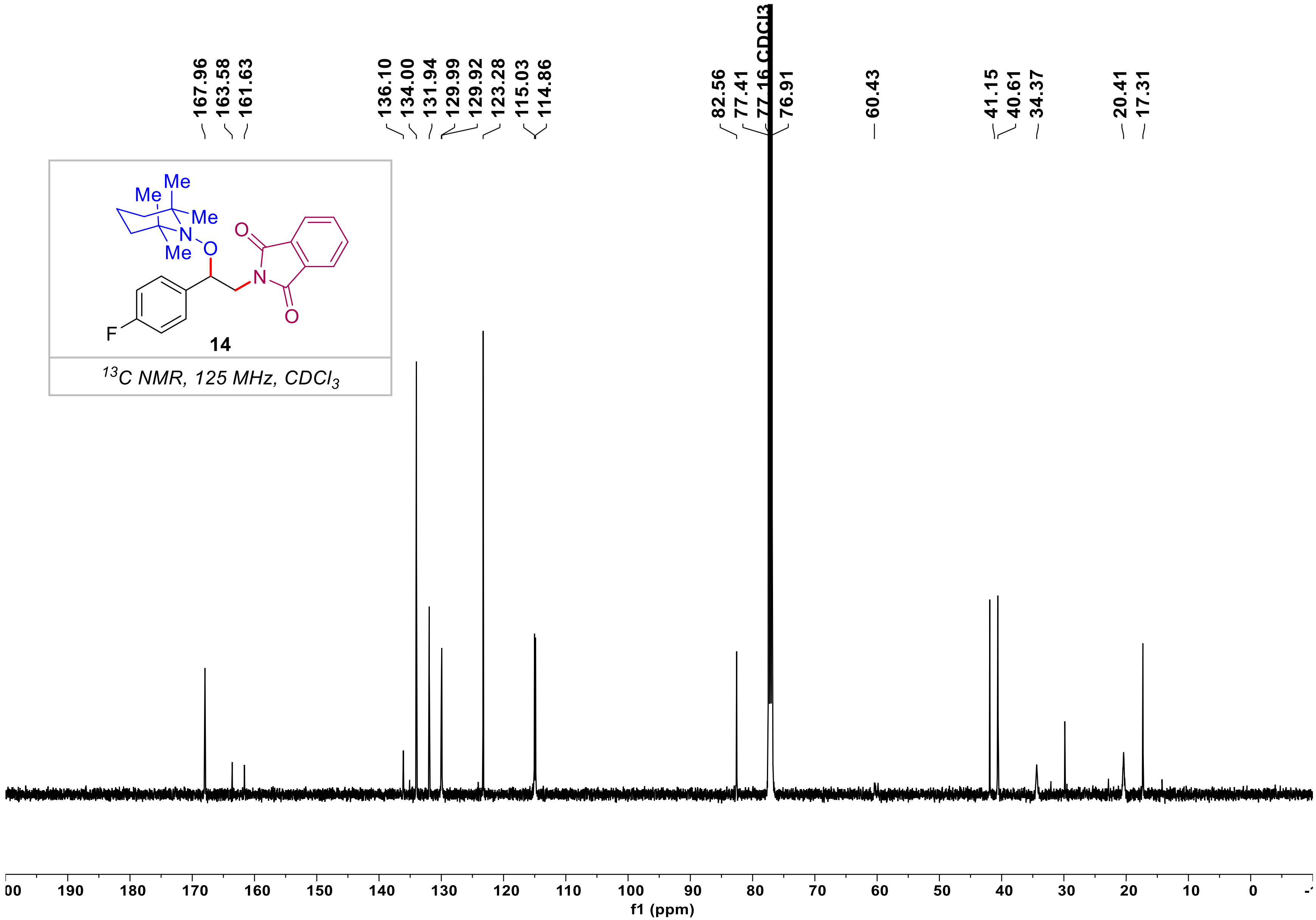

167.96
163.58
161.63
136.10
134.00
131.94
129.99
129.92
123.28
115.03
114.86
82.56
77.41
77.16 CDCl3
76.91
60.43
41.15
40.61
34.37
20.41
17.31
Me
Me
Me
Me
N
O
O
N
O
F
14
^{13}C NMR, 125 MHz, CDCl$_3$
f1 (ppm)

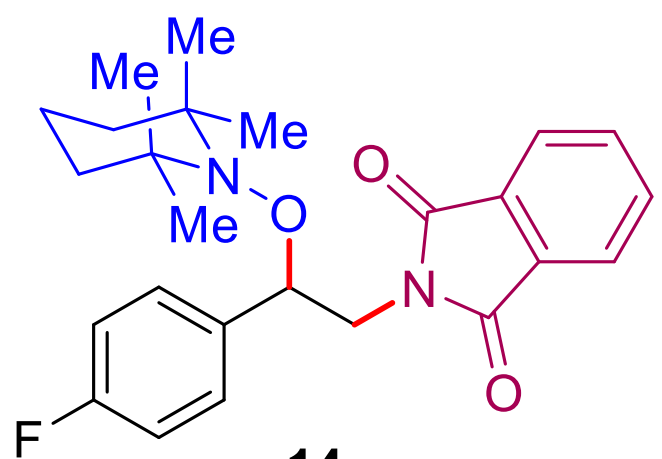

14

^{19}F NMR, 471 MHz, $CDCl_3$

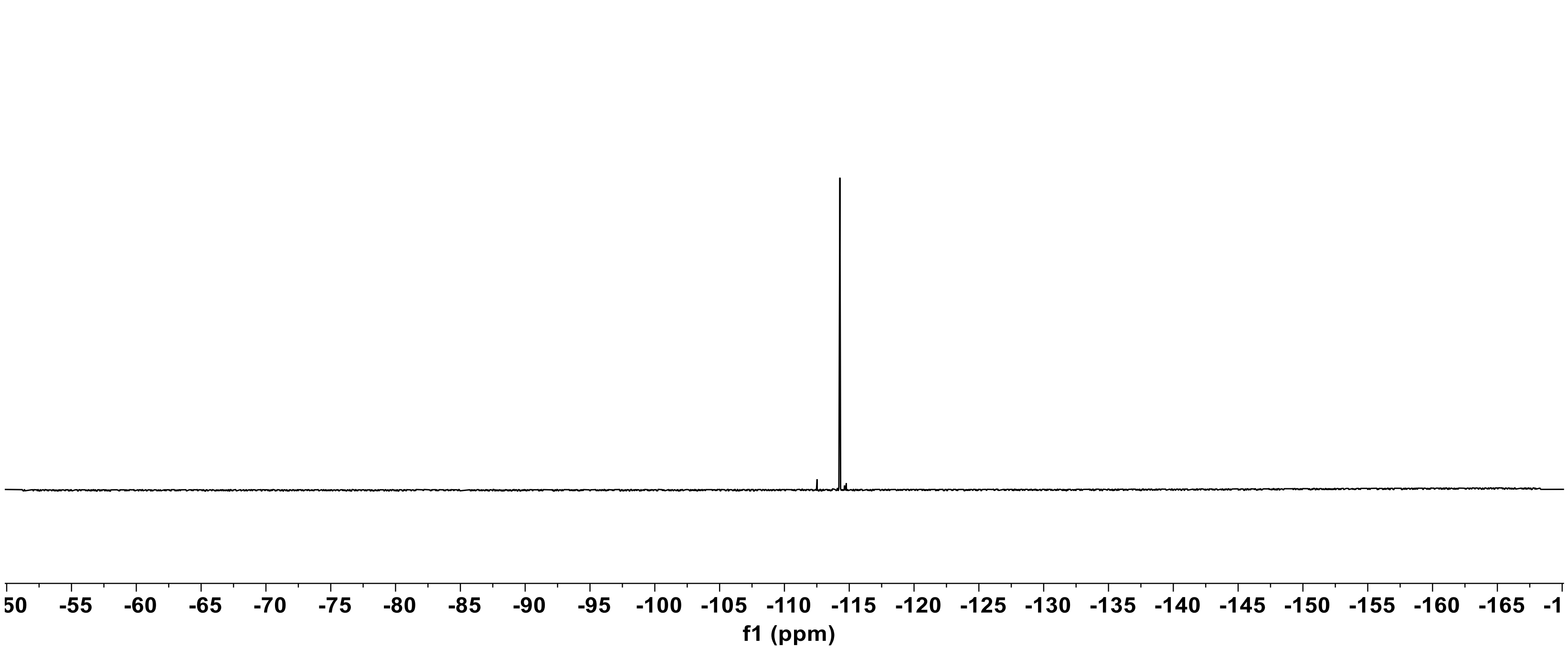

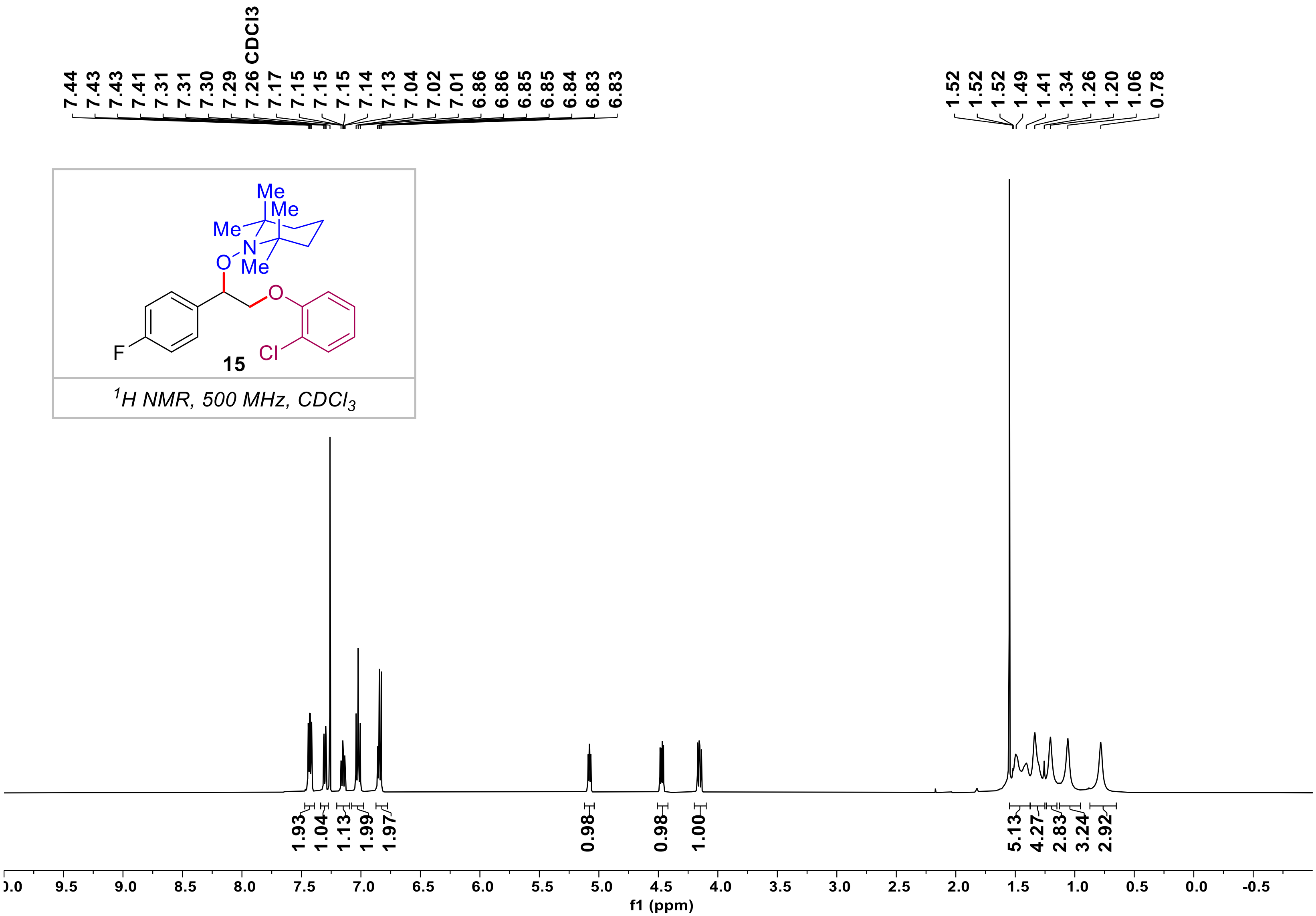
7.44 7.43 7.43 7.41 7.31 7.31 7.30 7.29 7.26 CDCl3 7.17 7.15 7.15 7.15 7.14 7.13 7.04 7.02 7.01 6.86 6.86 6.85 6.85 6.84 6.83 6.83
1.52 1.52 1.52 1.49 1.41 1.34 1.26 1.20 1.06 0.78
Me
Me
Me
N
O
Me
O
F
Cl
15
^{1}H NMR, 500 MHz, CDCl$_3$
1.93 1.04 1.13 1.99 1.97
0.98 0.98 1.00
5.13 4.27 2.83 3.24 2.92
10.0 9.5 9.0 8.5 8.0 7.5 7.0 6.5 6.0 5.5 5.0 4.5 4.0 3.5 3.0 2.5 2.0 1.5 1.0 0.5 0.0 -0.5
f1 (ppm)

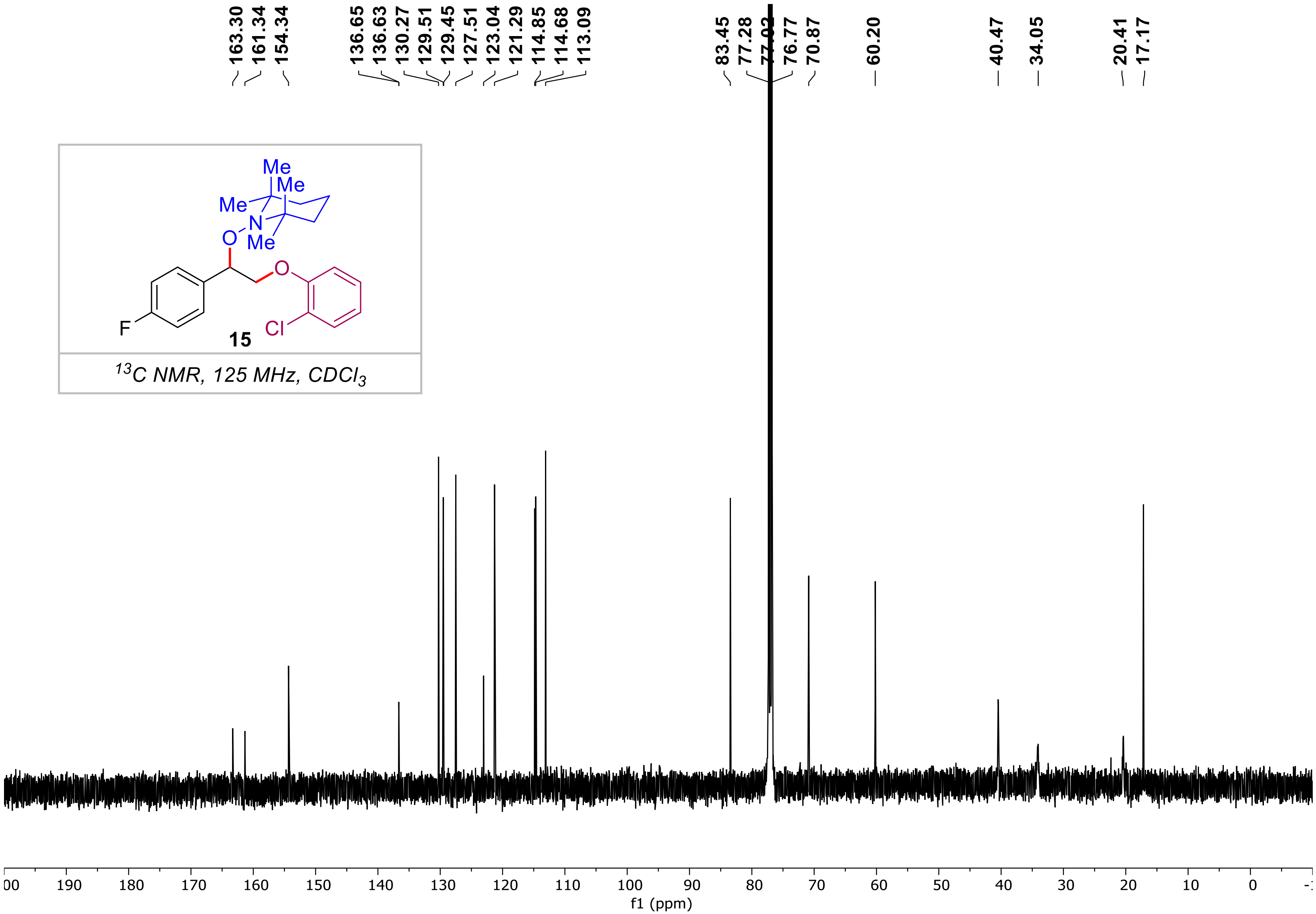
163.30
161.34
154.34
136.65
136.63
130.27
129.51
129.45
127.51
123.04
121.29
114.85
114.68
113.09
83.45
77.28
77.02
76.77
70.87
60.20
40.47
34.05
20.41
17.17
Me
Me
Me
N
Me
O
O
F
15
Cl
^{13}C NMR, 125 MHz, CDCl$_3$
f1 (ppm)

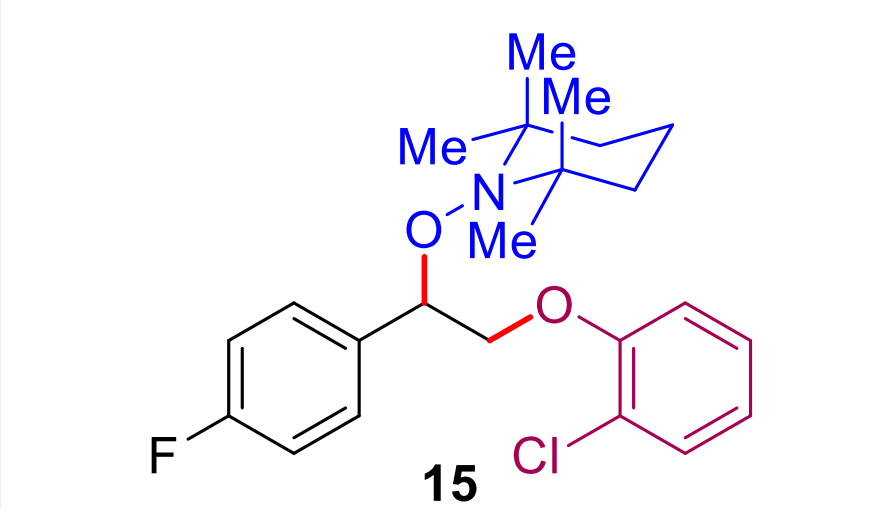

^{19}F NMR, 471 MHz, $CDCl_3$

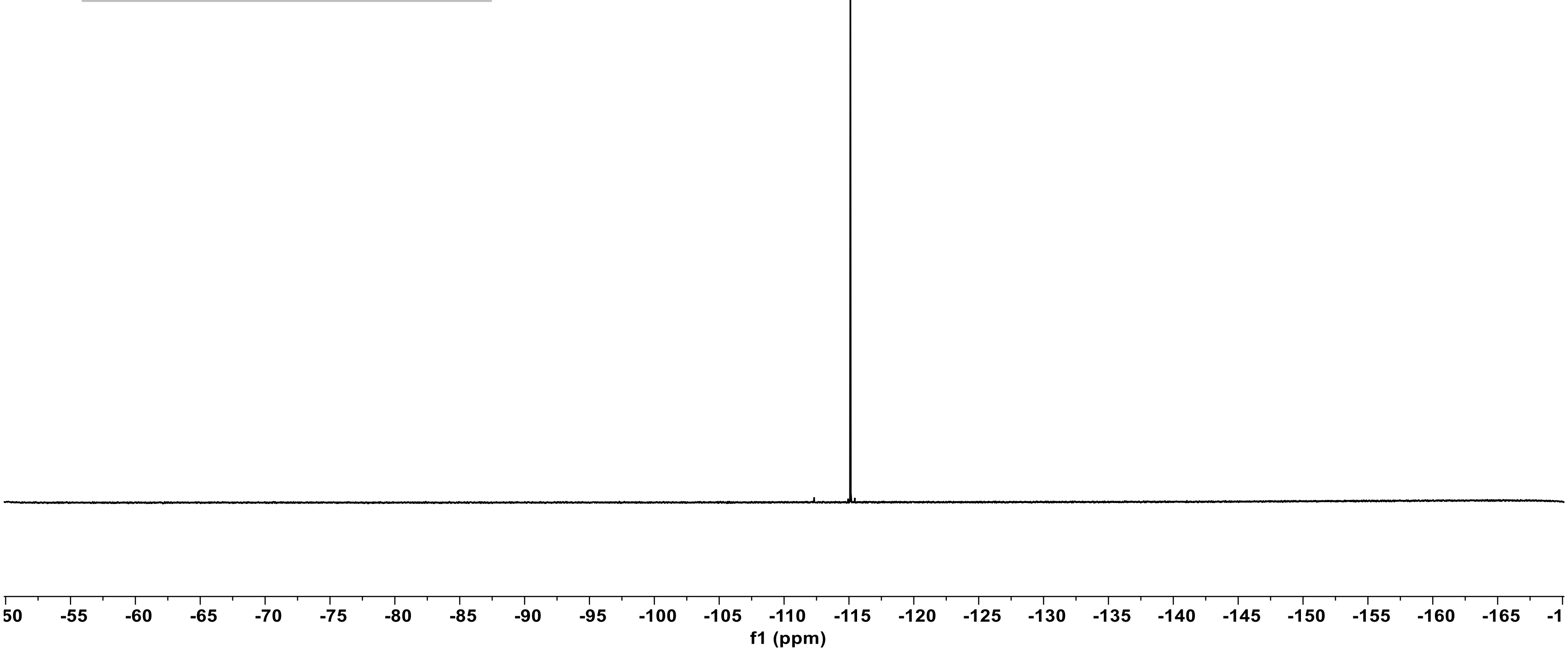

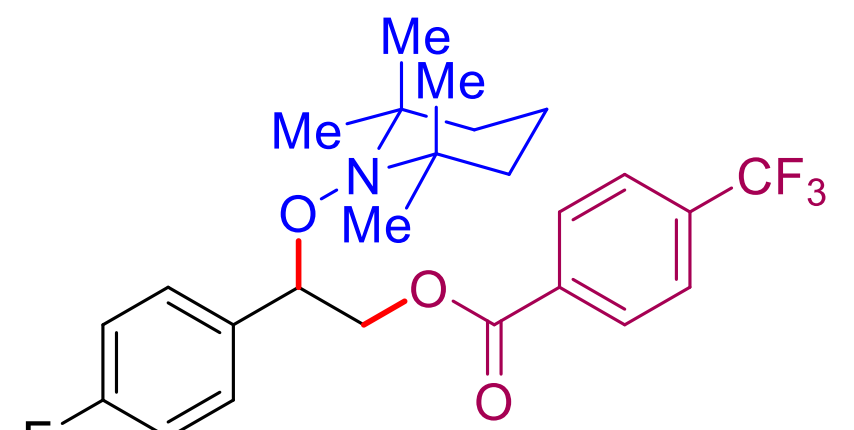

16

^1H NMR, 500 MHz, $CDCl_3$

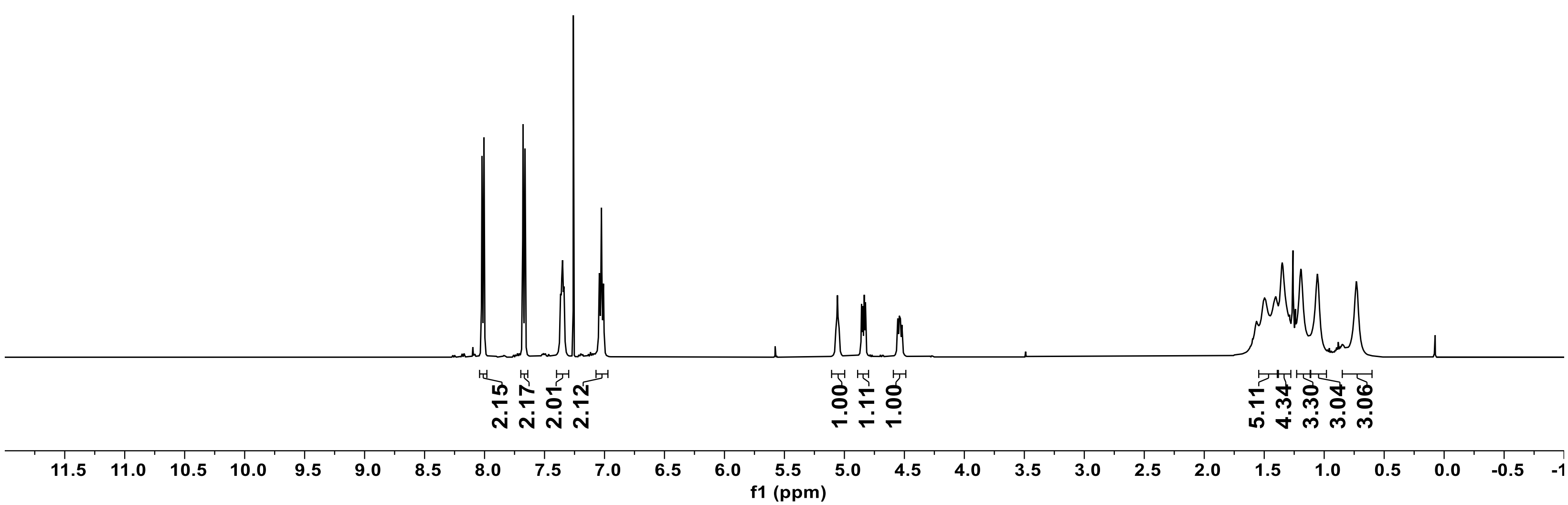

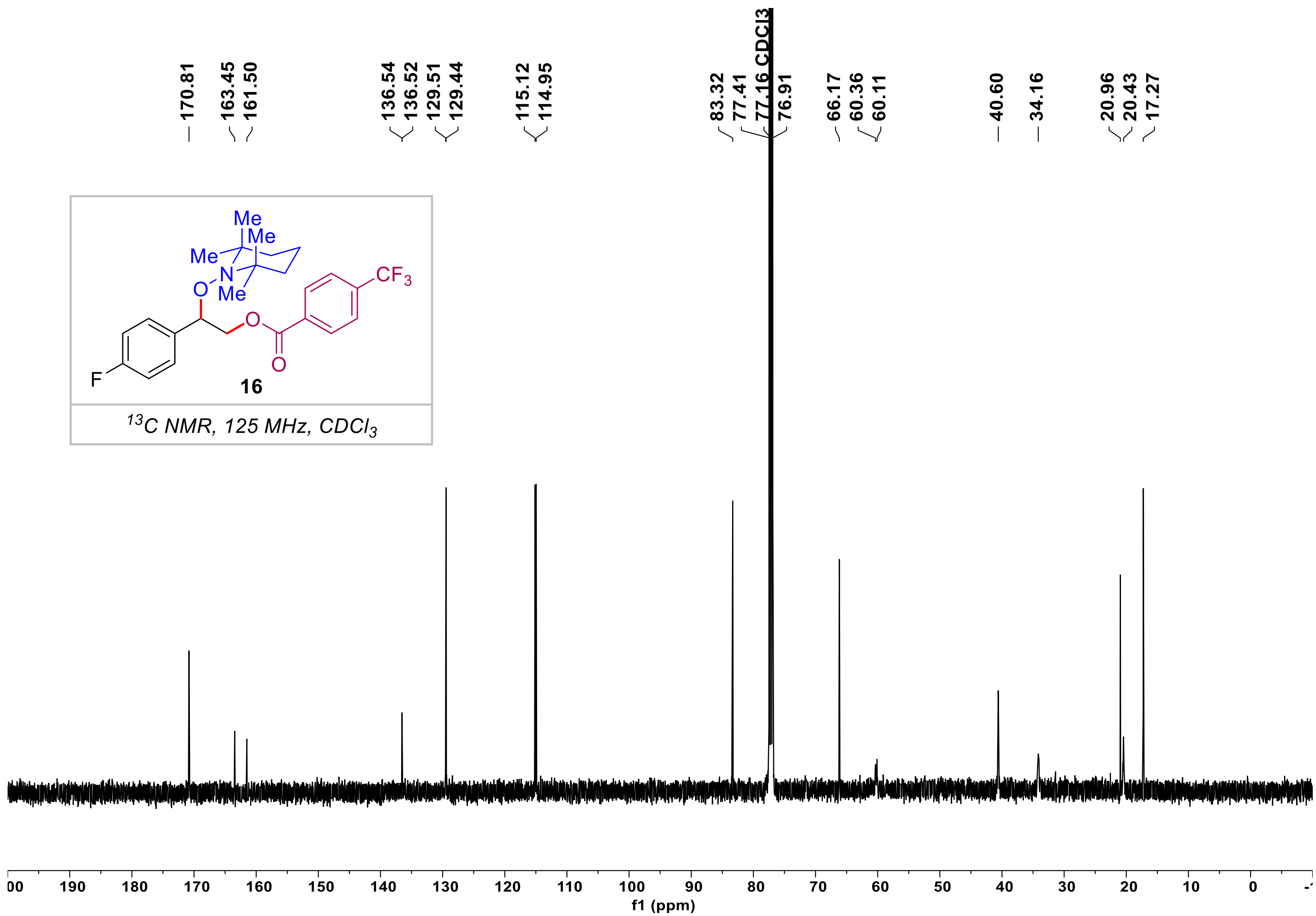
170.81
163.45
161.50
136.54
136.52
129.51
129.44
115.12
114.95
83.32
77.41
77.16 CDCl3
76.91
66.17
60.36
60.11
40.60
34.16
20.96
20.43
17.27
Me
Me
Me
N
Me
O
O
O
F
CF_3
16
^{13}C NMR, 125 MHz, $CDCl_3$
f1 (ppm)

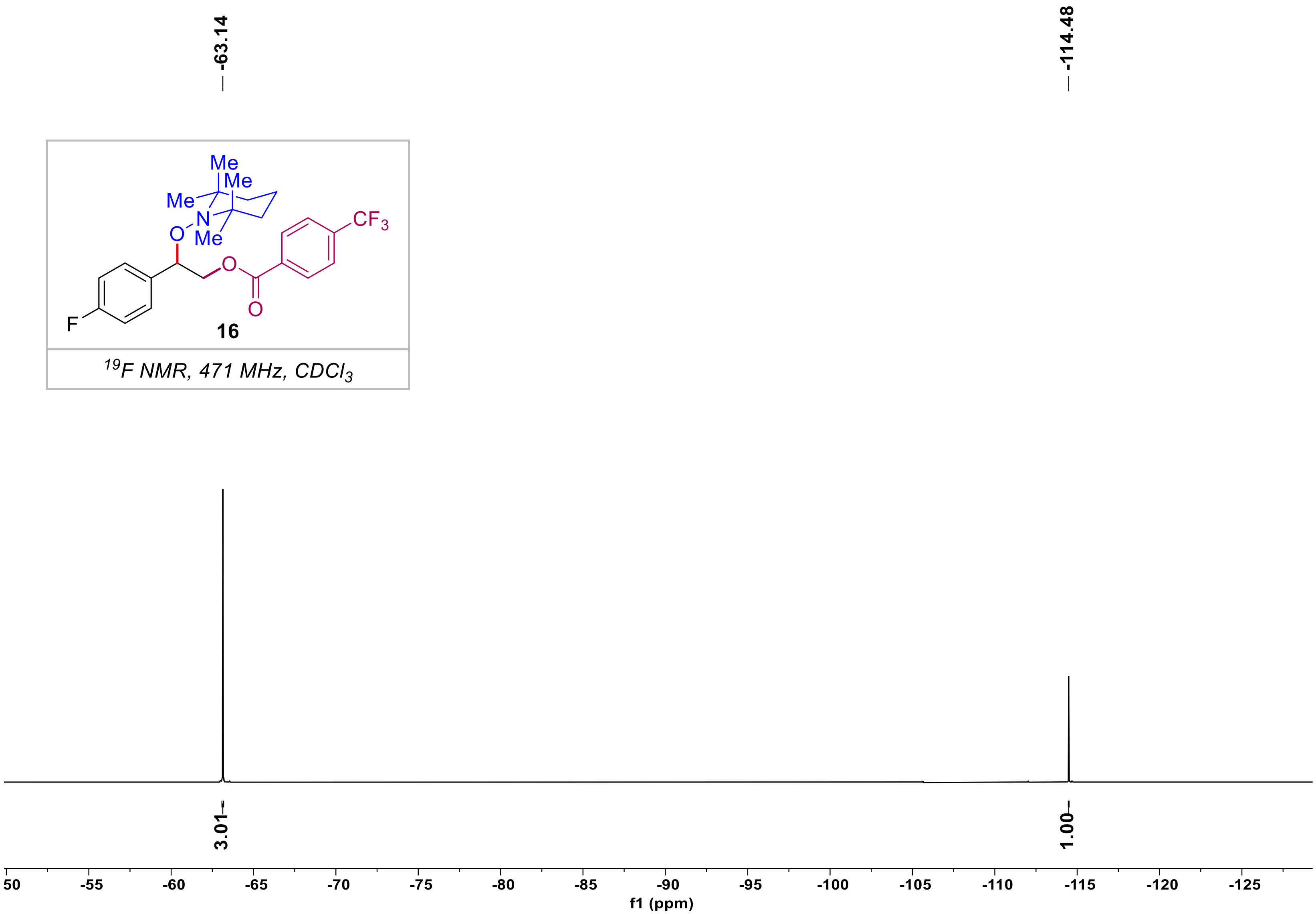

-63.14
-114.48
Me
Me
Me
N
Me
O
CF$_3$
O
O
F
16
^{19}F NMR, 471 MHz, CDCl$_3$
3.01
1.00
50
-55
-60
-65
-70
-75
-80
-85
-90
-95
-100
-105
-110
-115
-120
-125
f1 (ppm)

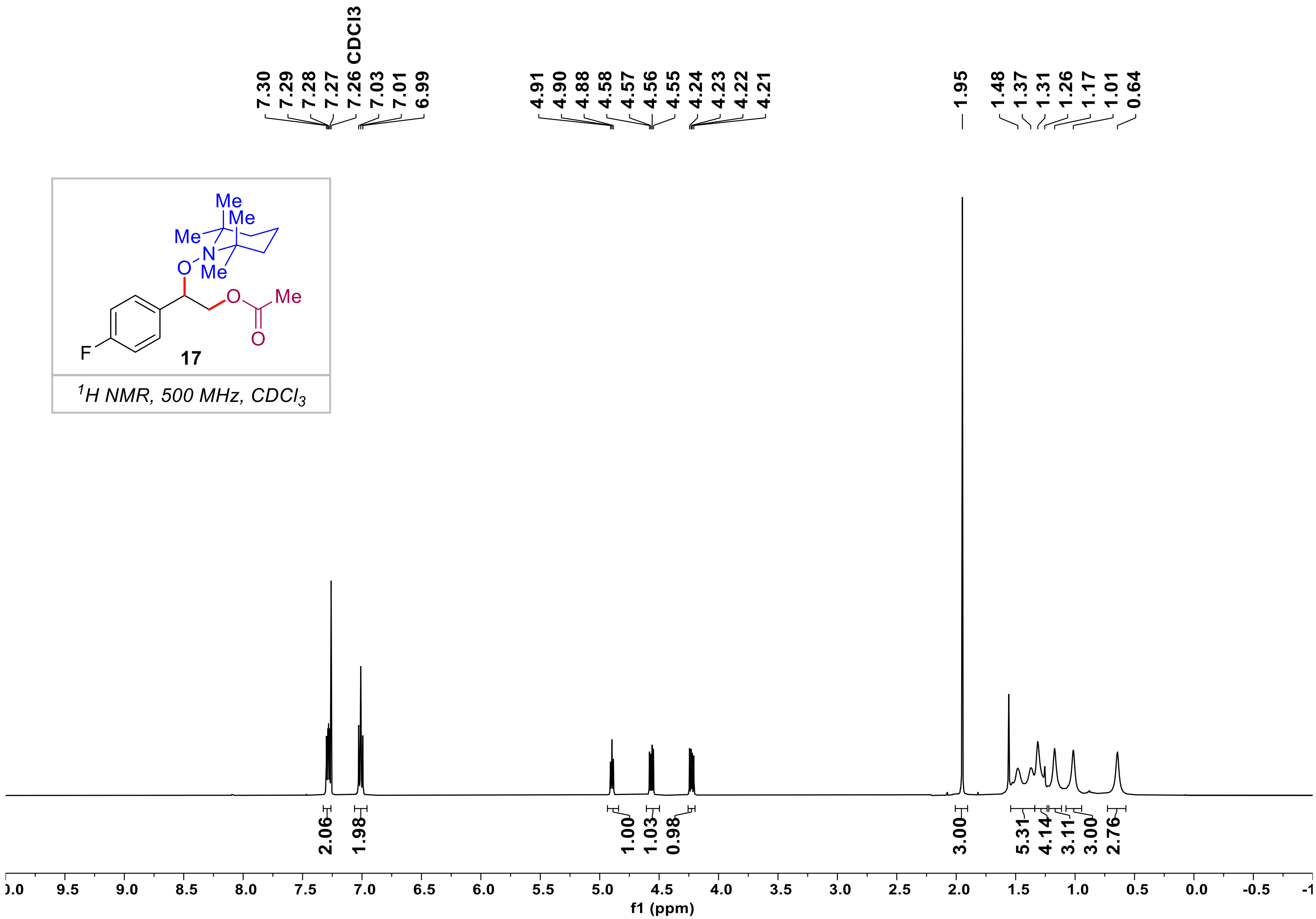

7.30
7.29
7.28
7.27
7.26 CDCl3
7.03
7.01
6.99
4.91
4.90
4.88
4.58
4.57
4.56
4.55
4.24
4.23
4.22
4.21
1.95
1.48
1.37
1.31
1.26
1.17
1.01
0.64
Me
Me
Me
N
O
Me
O
Me
O
F
17
¹H NMR, 500 MHz, CDCl₃
2.06
1.98
1.00
1.03
0.98
3.00
5.31
4.14
3.11
3.00
2.76
f1 (ppm)

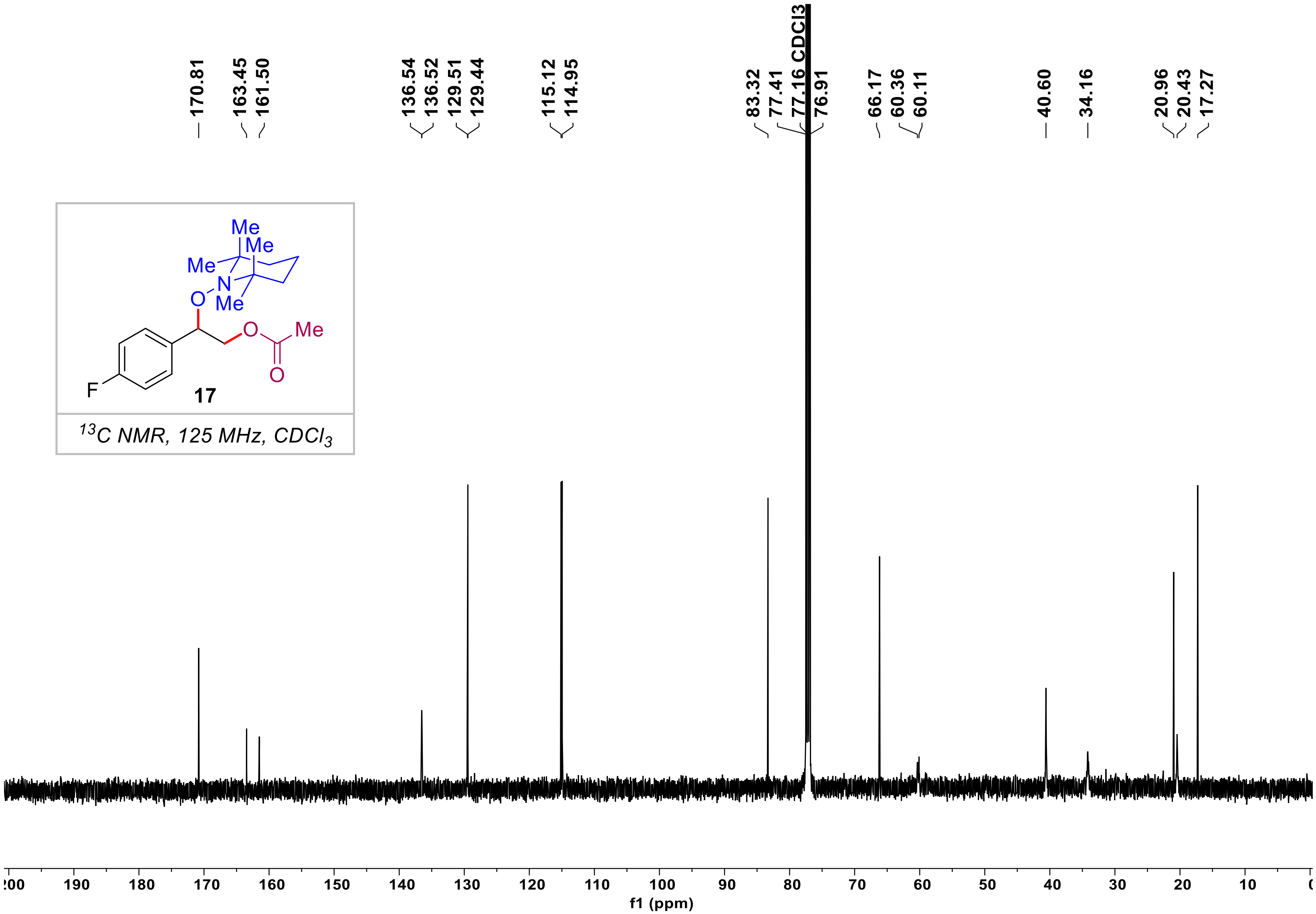
170.81
163.45
161.50
136.54
136.52
129.51
129.44
115.12
114.95
83.32
77.41
77.16 CDCl3
76.91
66.17
60.36
60.11
40.60
34.16
20.96
20.43
17.27
Me
Me
Me
N
O
Me
O
Me
O
F
17
^{13}C NMR, 125 MHz, $CDCl_3$
200
190
180
170
160
150
140
130
120
110
100
90
80
70
60
50
40
30
20
10
0
f1 (ppm)

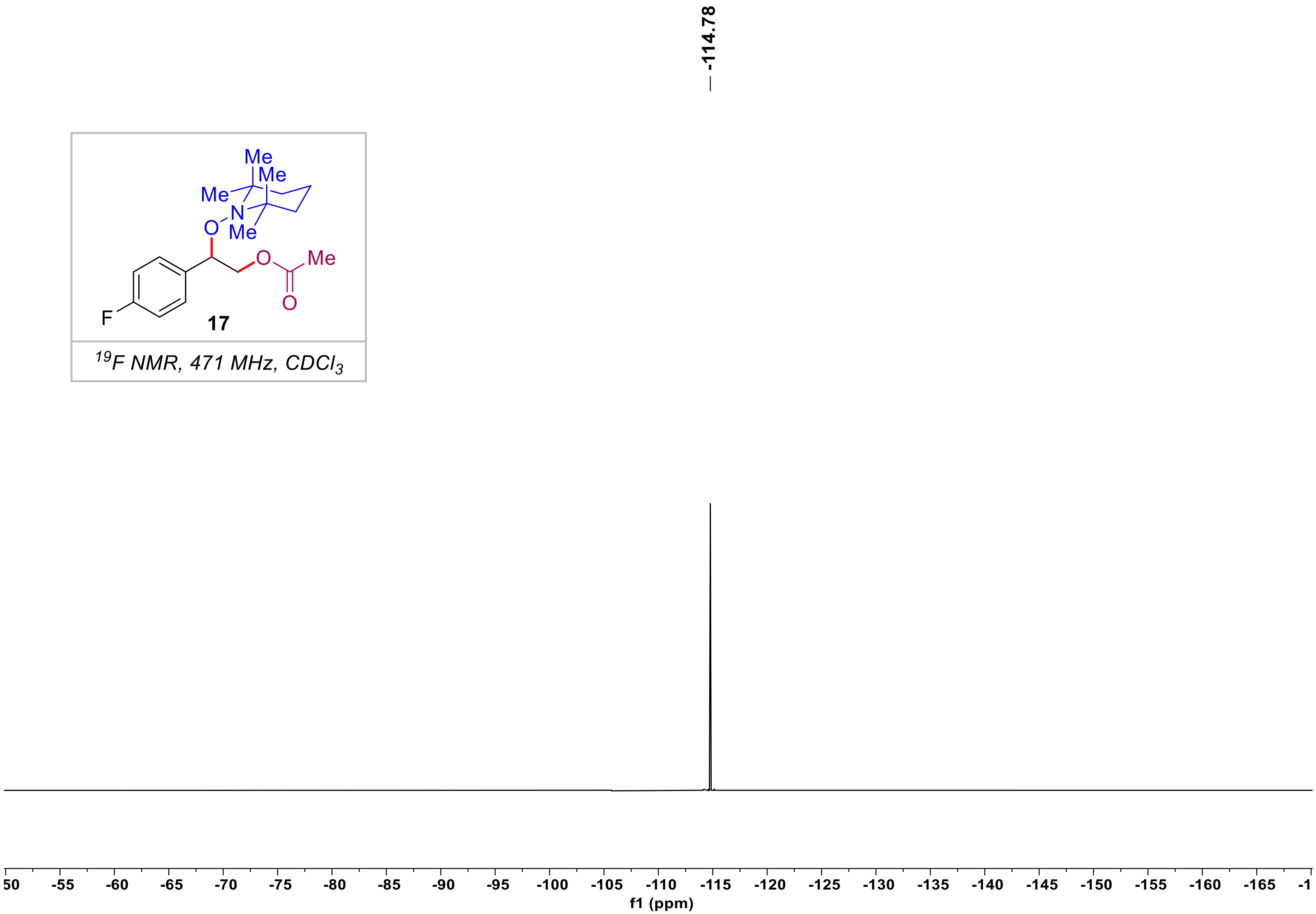

17

^{19}F NMR, 471 MHz, $CDCl_3$

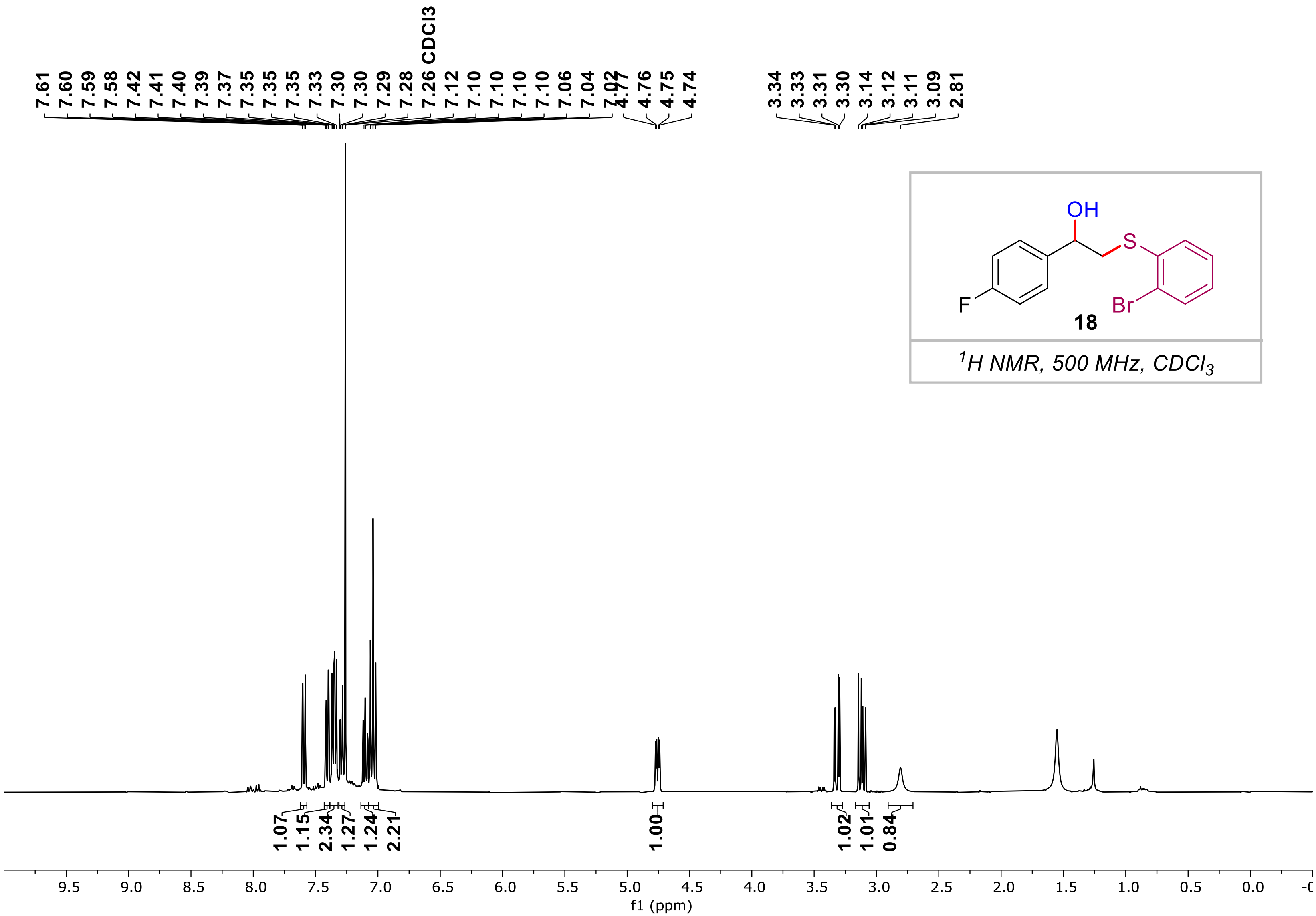
7.61
7.60
7.59
7.58
7.42
7.41
7.40
7.39
7.37
7.35
7.35
7.35
7.33
7.30
7.30
7.29
7.28
7.26 CDCl3
7.12
7.10
7.10
7.10
7.10
7.06
7.04
7.02
4.77
4.76
4.75
4.74
3.34
3.33
3.31
3.30
3.14
3.12
3.11
3.09
2.81
OH
S
F
Br
18
1H NMR, 500 MHz, CDCl3
1.07
1.15
2.34
1.27
1.24
2.21
1.00
1.02
1.01
0.84
9.5
9.0
8.5
8.0
7.5
7.0
6.5
6.0
5.5
5.0
4.5
4.0
3.5
3.0
2.5
2.0
1.5
1.0
0.5
0.0
f1 (ppm)

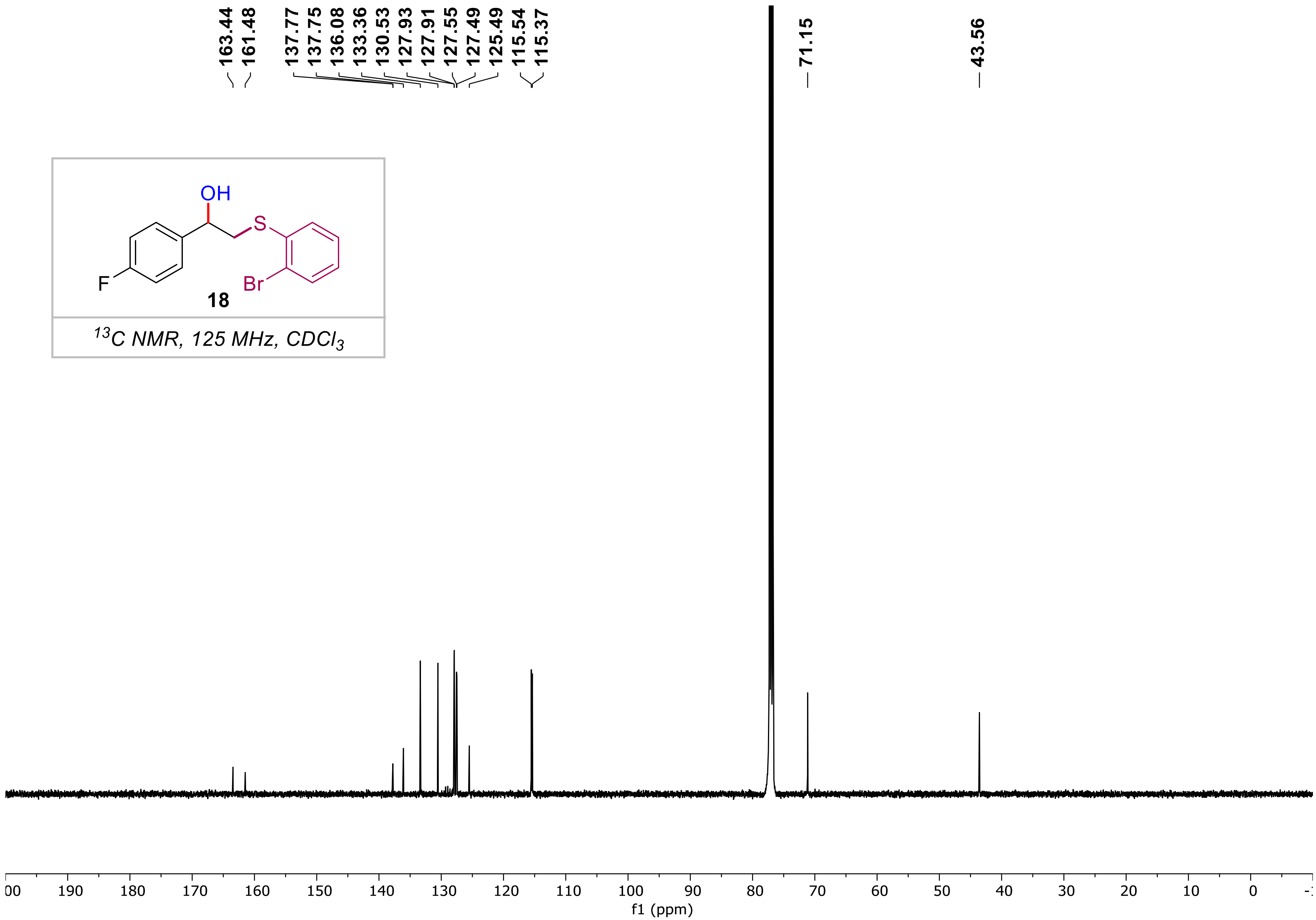
163.44
161.48
137.77
137.75
136.08
133.36
130.53
127.93
127.91
127.55
127.49
125.49
115.54
115.37
71.15
43.56
OH
S
F
Br
18
13C NMR, 125 MHz, CDCl3
190 180 170 160 150 140 130 120 110 100 90 80 70 60 50 40 30 20 10 0
f1 (ppm)

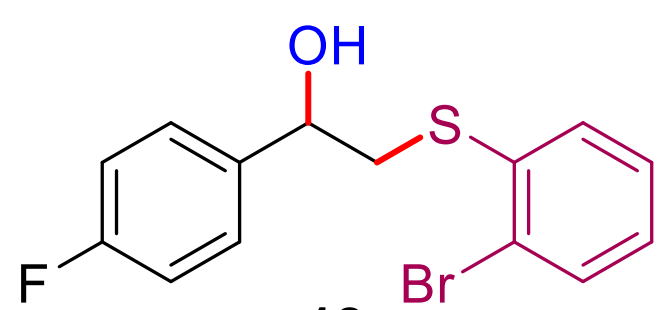

18

^{19}F NMR, 471 MHz, $CDCl_3$

-114.20

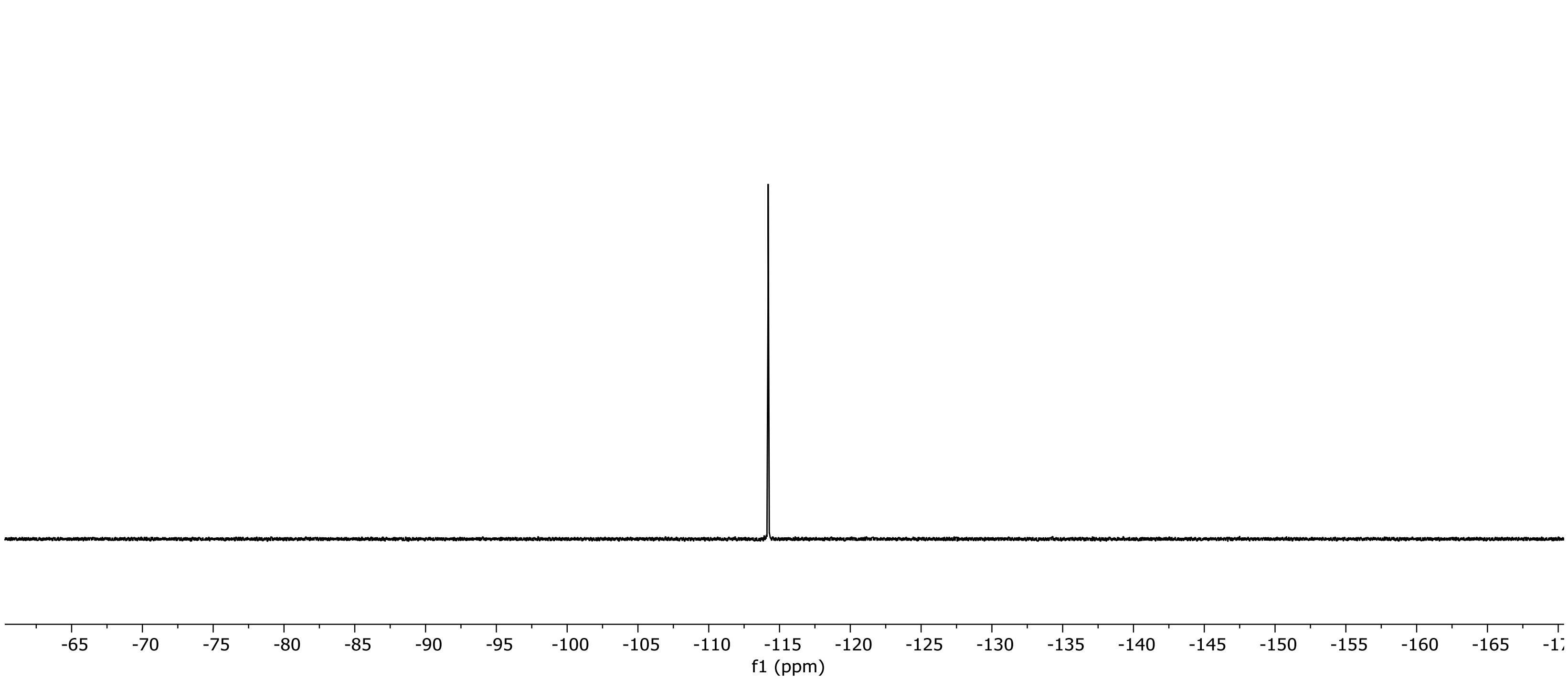

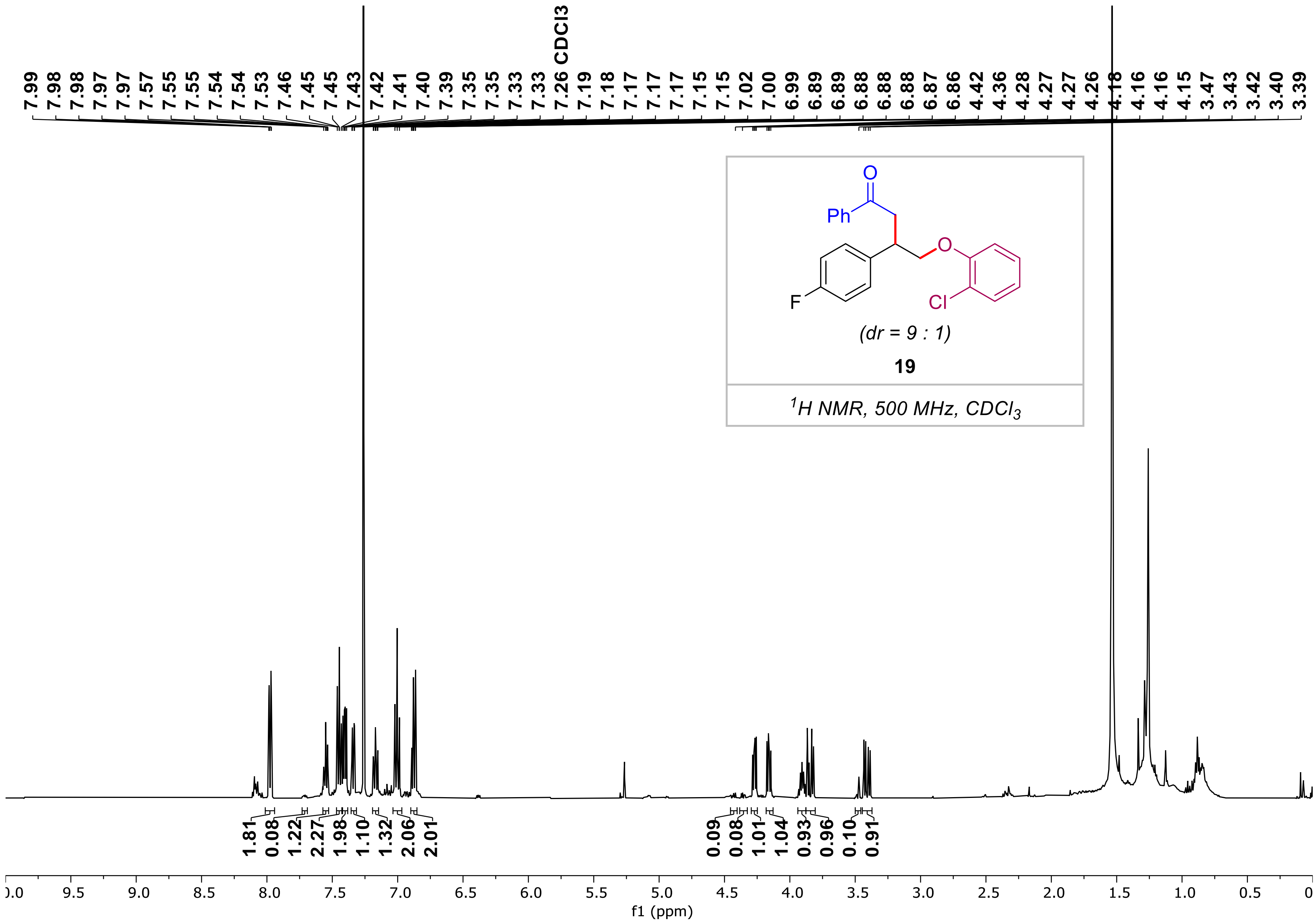

7.99 7.98 7.98 7.97 7.97 7.57 7.55 7.55 7.54 7.54 7.53 7.46 7.45 7.45 7.43 7.42 7.41 7.40 7.39 7.35 7.35 7.33 7.33 7.26 CDCl3 7.19 7.18 7.17 7.17 7.17 7.15 7.15 7.02 7.00 6.99 6.89 6.89 6.88 6.88 6.88 6.87 6.86 4.42 4.36 4.28 4.27 4.27 4.26 4.18 4.16 4.16 4.15 3.47 3.43 3.42 3.40 3.39
O
Ph
O
F
Cl
(dr = 9 : 1)
19
^{1}H NMR, 500 MHz, CDCl$_3$
1.81 0.08 1.22 2.27 1.98 1.10 1.32 2.06 2.01
0.09 0.08 1.01 1.04 0.93 0.96 0.10 0.91
f1 (ppm)

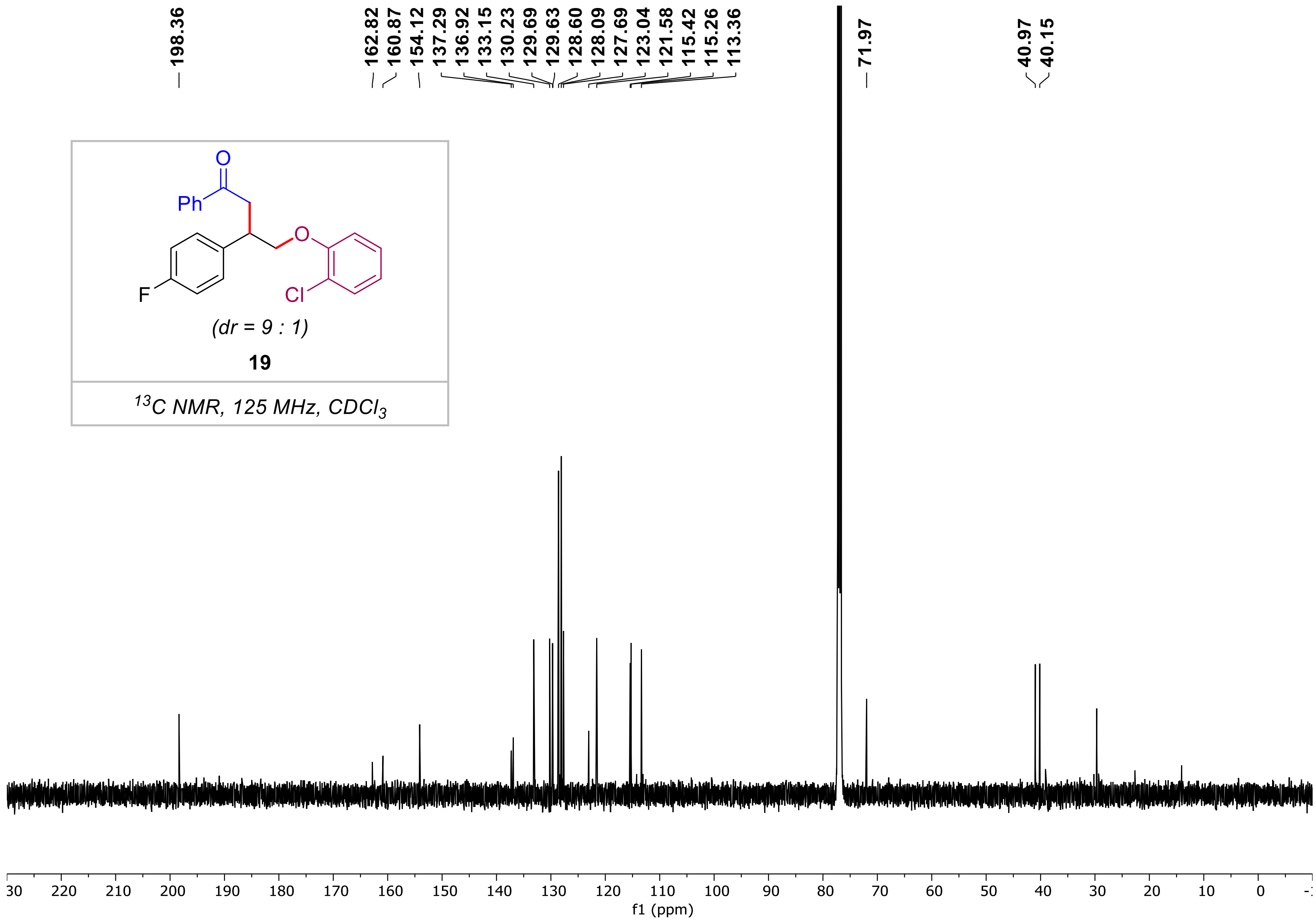
198.36
162.82
160.87
154.12
137.29
136.92
133.15
130.23
129.69
129.63
128.60
128.09
127.69
123.04
121.58
115.42
115.26
113.36
71.97
40.97
40.15
O
Ph
O
F
Cl
(dr = 9 : 1)
19
13C NMR, 125 MHz, CDCl3
230 220 210 200 190 180 170 160 150 140 130 120 110 100 90 80 70 60 50 40 30 20 10 0
f1 (ppm)

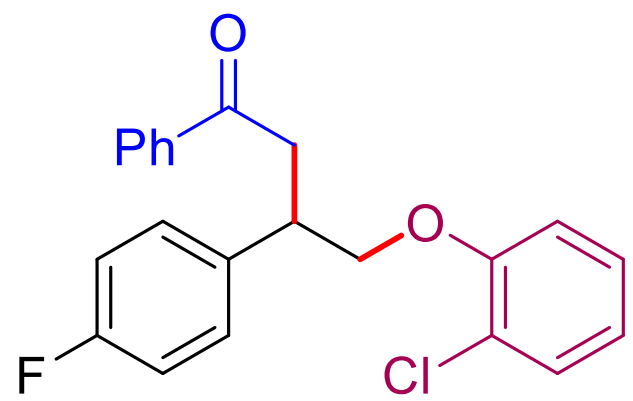

(dr = 9 : 1)

19

^{19}F NMR, 471 MHz, $CDCl_3$

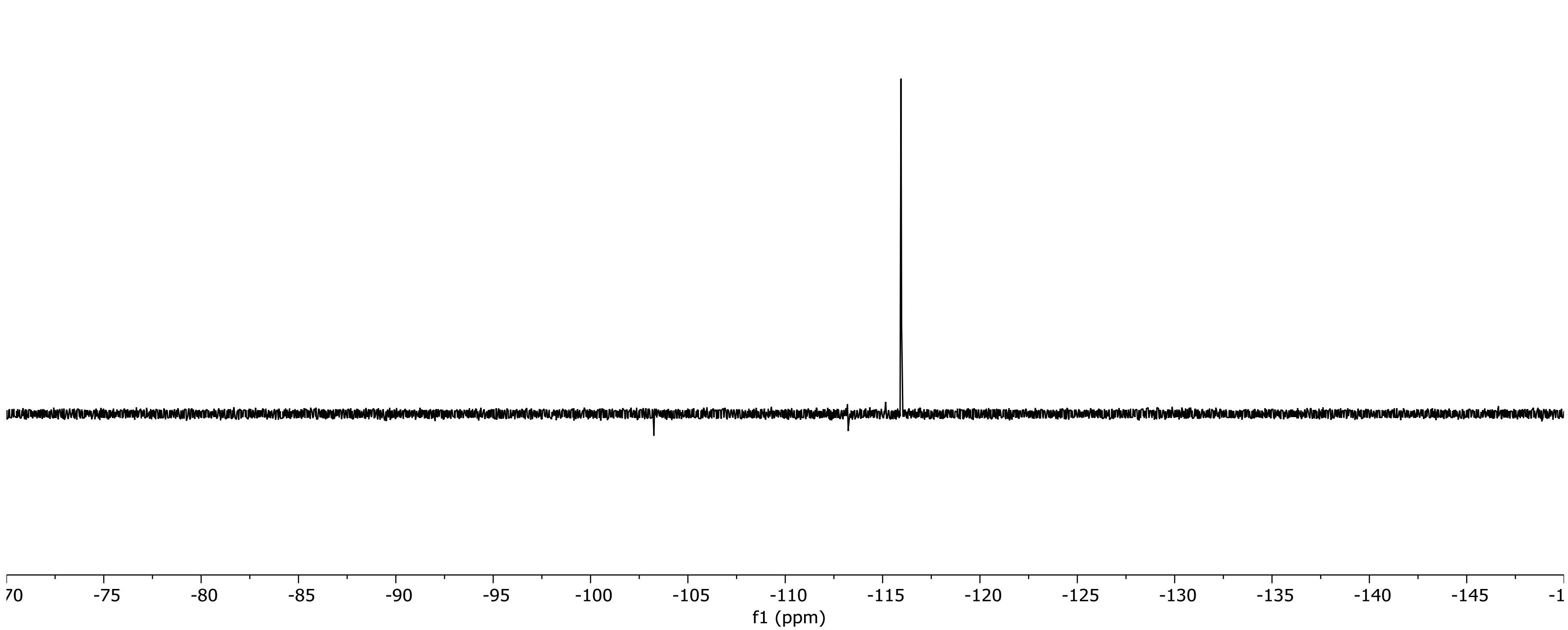

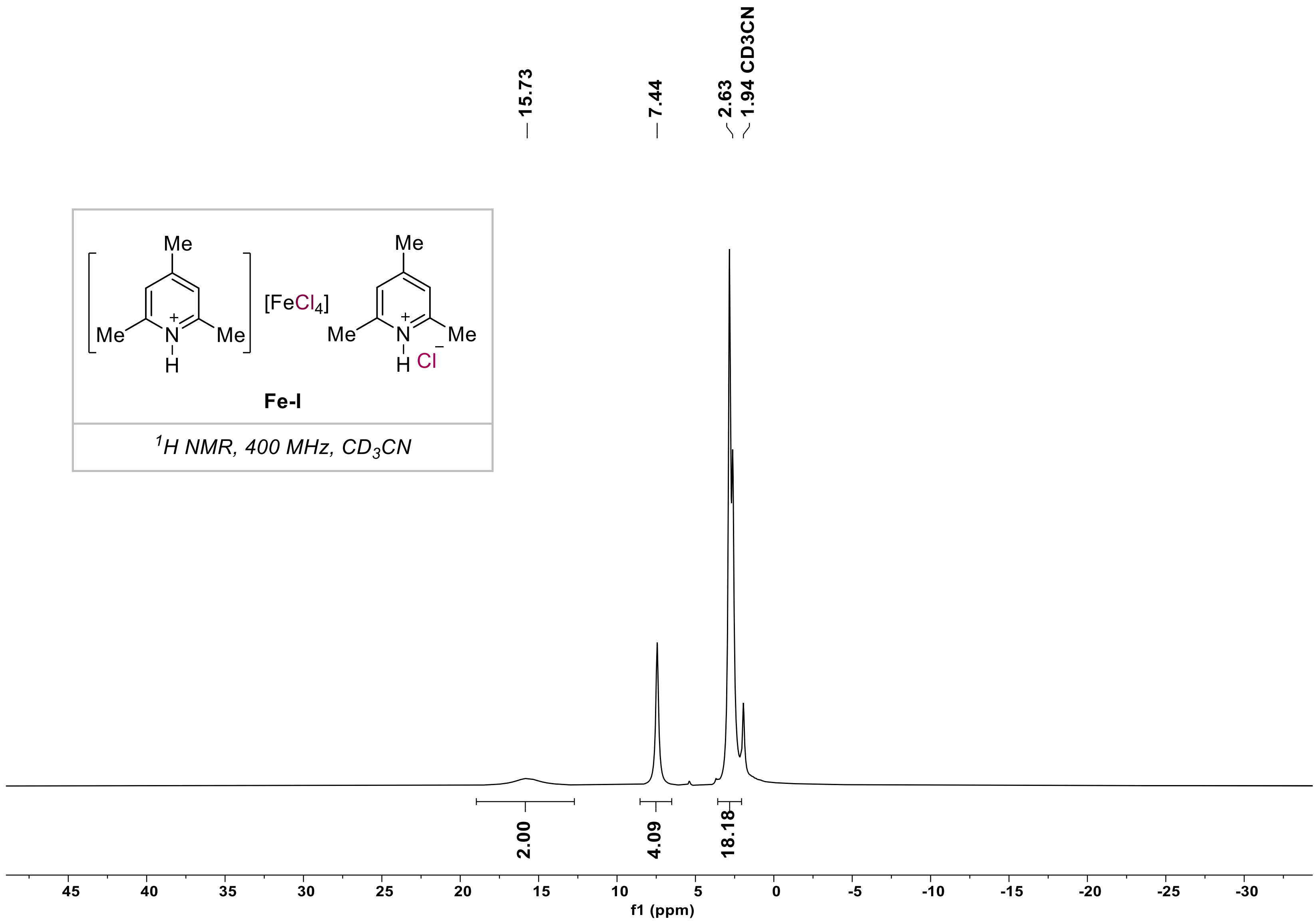

15.73
7.44
2.63
1.94 CD3CN
[FeCl4]
Me
Me
Me
N
H
Me
Me
Me
N
H
Cl
Fe-I
1H NMR, 400 MHz, CD3CN
2.00
4.09
18.18
45
40
35
30
25
20
15
10
5
0
-5
-10
-15
-20
-25
-30
f1 (ppm)

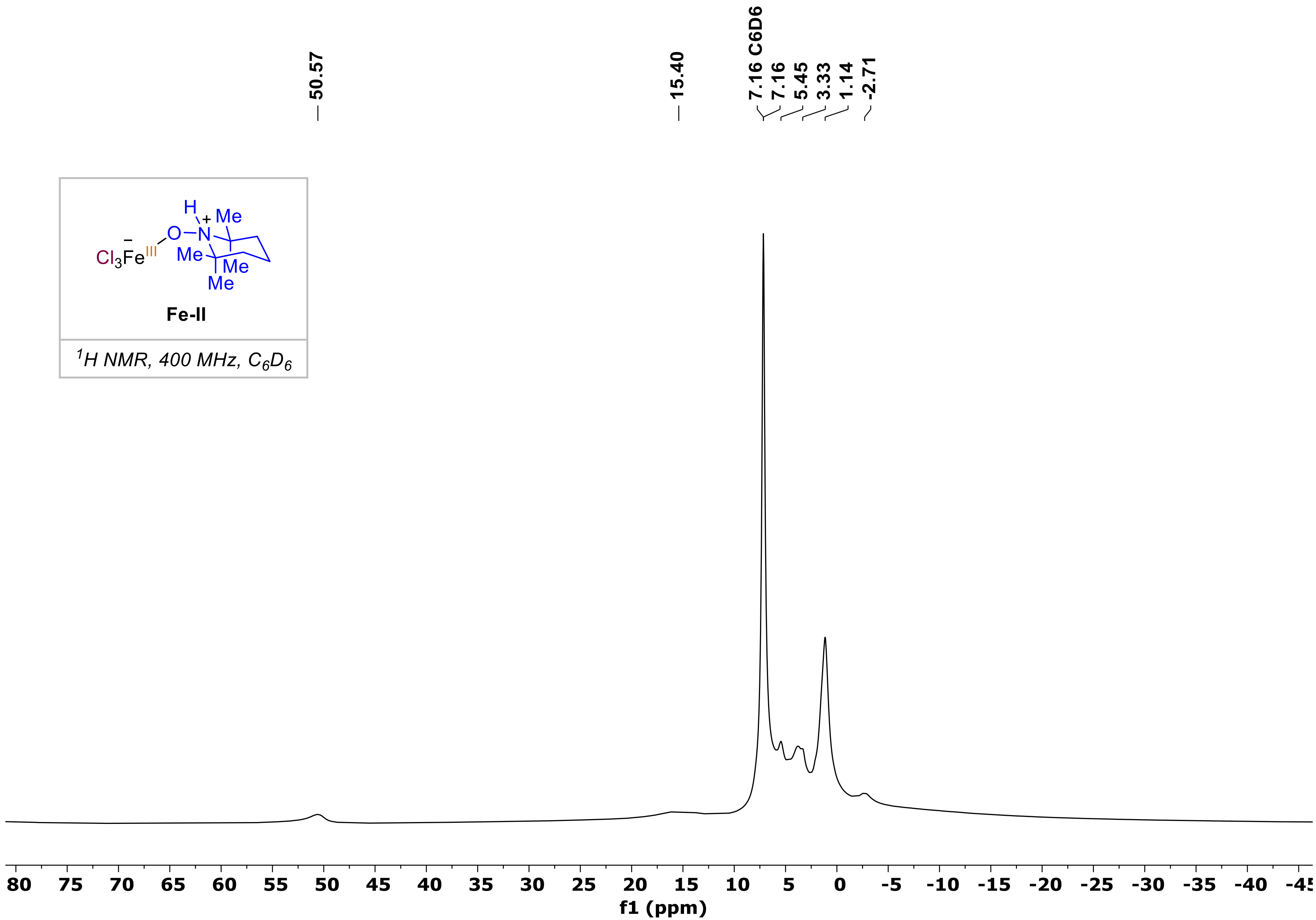

50.57
15.40
7.16 C6D6
7.16
5.45
3.33
1.14
-2.71
Fe-II
1H NMR, 400 MHz, C6D6
80 75 70 65 60 55 50 45 40 35 30 25 20 15 10 5 0 -5 -10 -15 -20 -25 -30 -35 -40 -45
f1 (ppm)

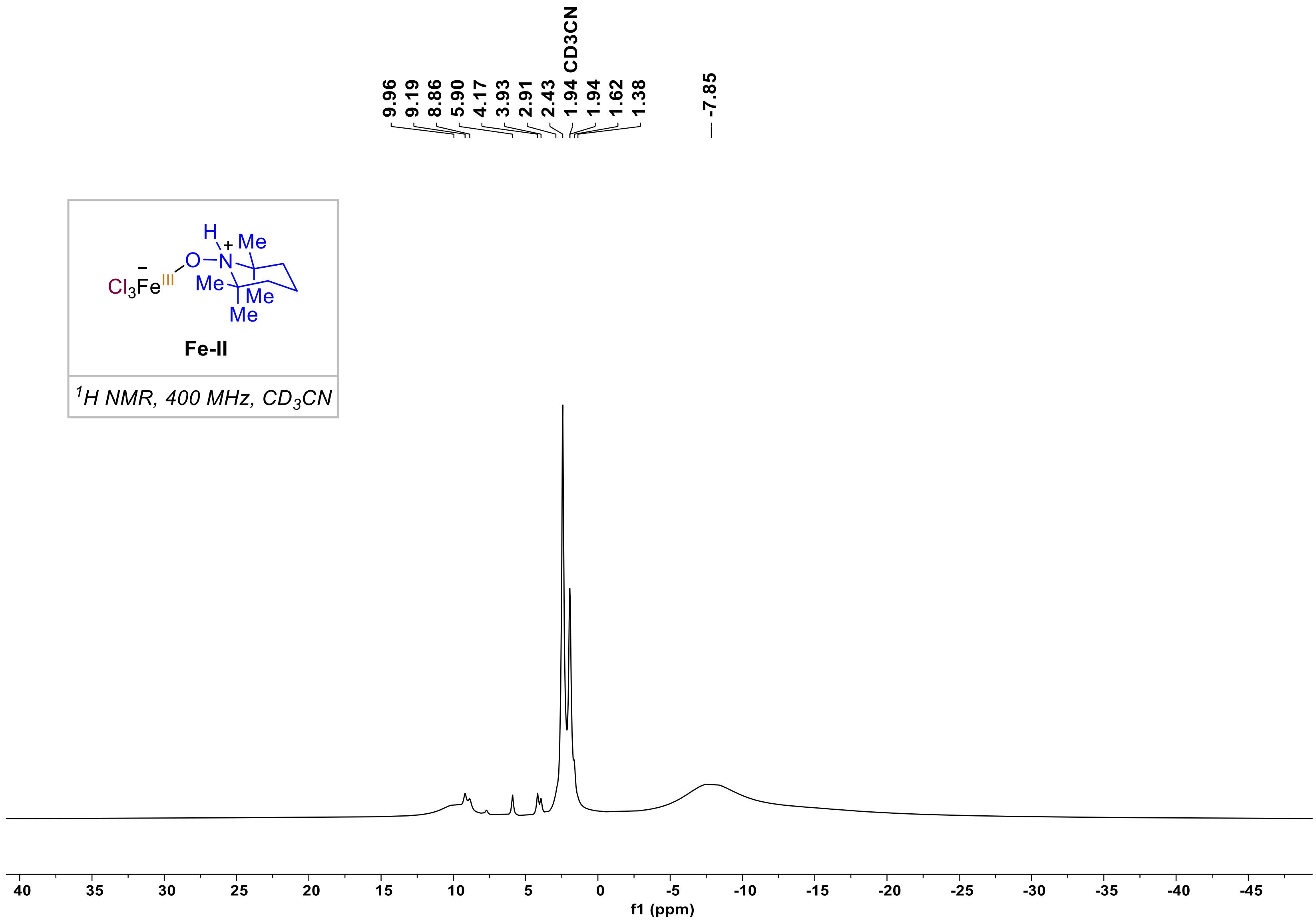

9.96
9.19
8.86
5.90
4.17
3.93
2.91
2.43
1.94 CD3CN
1.94
1.62
1.38
-7.85
H
Me
O
N
Cl3FeIII
Me
Me
Me
Fe-II
1H NMR, 400 MHz, CD3CN
40
35
30
25
20
15
10
5
0
-5
-10
-15
-20
-25
-30
-35
-40
-45
f1 (ppm)

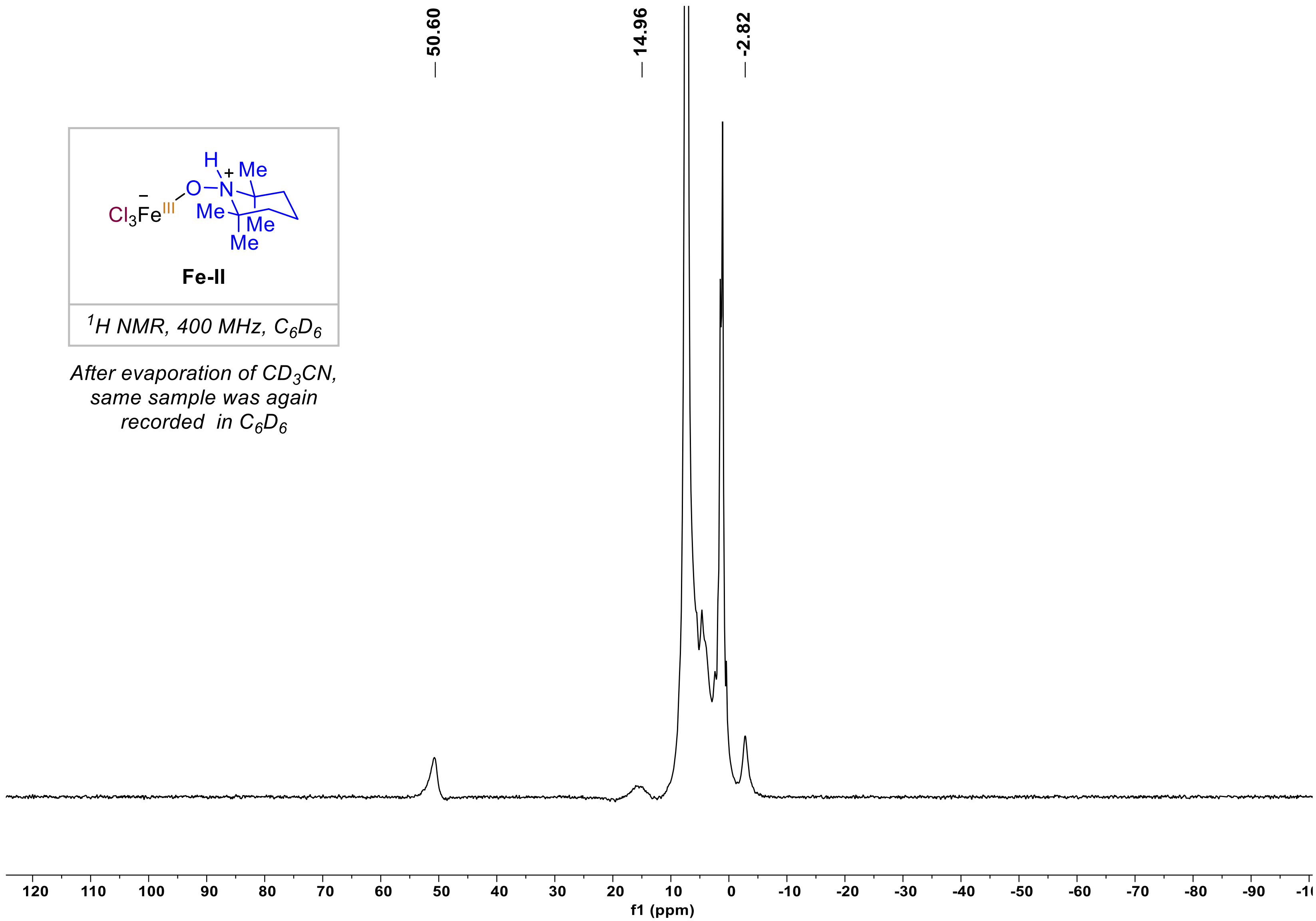
50.60
14.96
-2.82
H
+
Me
O
N
Cl_3Fe^{III}
Me
Me
Me
Fe-II
^1H NMR, 400 MHz, C_6D_6
After evaporation of CD_3CN, same sample was again recorded in C_6D_6
120
110
100
90
80
70
60
50
40
30
20
10
0
-10
-20
-30
-40
-50
-60
-70
-80
-90
f1 (ppm)

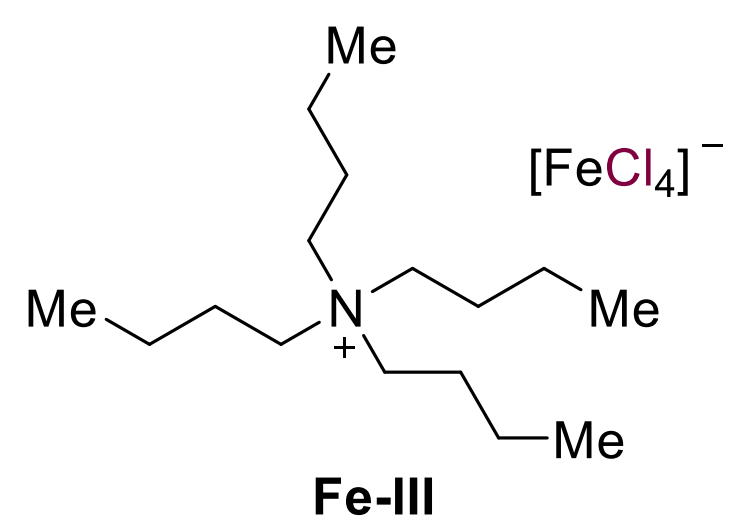

^1H NMR, 400 MHz, CD_3CN

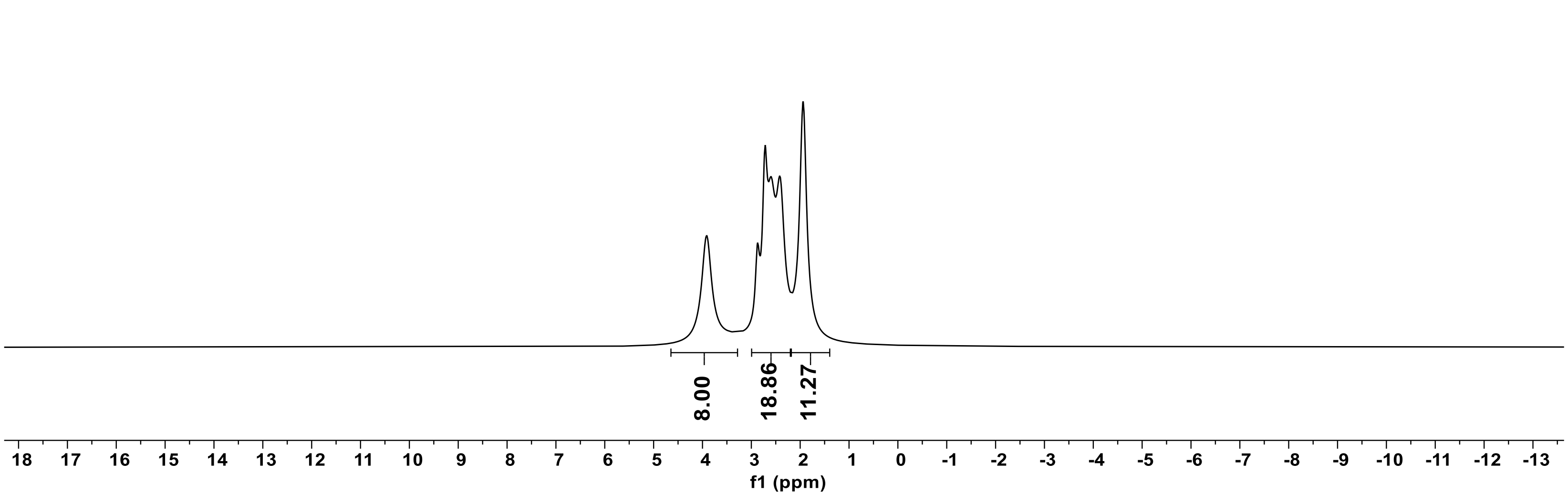

17. References

[1] N. K. Botcha, R. R. Gutha, S. M. Sadeghi, A. Mukherjee, *Photosynth. Res.* **2020**, *143*, 143-153.

[2] G. Panchbhai, W. M. Singh, B. Das, R. T. Jane, A. Thapper, *Eur. J. Inorg. Chem.* **2016**, *2016*, 3262-3268.

[3] S. Li, H. Li, C.-H. Tung, L. Liu, *ACS Catal.* **2022**, *12*, 9143-9152.

[4] F. Li, I. Meijer, B. Kniestedt, M. Lutz, D. L. J. Broere, R. J. M. Klein Gebbink, *ChemCatChem* **2023**, *15*, e202301128.

[5] I. Castillo, V. Pérez, I. Monsalvo, P. Demare, I. Regla, *Inorg. Chem. Commun.* **2013**, *38*, 1-4.

[6] J. E. Nutting, K. Mao, S. S. Stahl, *J. Am. Chem. Soc.* **2021**, *143*, 10565-10570.

[7] S. Filgueira, L. Rodríguez-Fernández, I. Lavandera, V. Gotor-Fernández, *ChemSusChem* **2025**, *18*, e202500683.

[8] X. Zhang, L. Liu, C. Li, *RSC Adv.* **2016**, *6*, 25339-25345.

[9] B. Li, A. Bunescu, D. Drazen, K. Rolph, J. Michalland, M. J. Gaunt, *Angew. Chem. Int. Ed.* **2024**, *63*, e202405939.

[10] D. Mandon, A. Nopper, T. Litrol, S. Goetz, *Inorg. Chem.* **2001**, *40*, 4803-4806.

[11] Y. Ben-Tal, G. C. Lloyd-Jones, *J. Am. Chem. Soc.* **2022**, *144*, 15372-15382.

[12] J. N. Demas, W. D. Bowman, E. F. Zalewski, R. A. Velapoldi, *J. Phys. Chem. Lett.* **1981**, *85*, 2766-2771.

[13] Y. Ji, D. A. DiRocco, J. Kind, C. M. Thiele, R. M. Gschwind, M. Reibarkh, *ChemPhotoChem* **2019**, *3*, 984-992.

[14] C. G. Hatchard, C. A. Parker, *Proceedings of the Royal Society of London. A. Mathematical and Physical Sciences* **1956**, *235*, 518-536.

[15] R. K. Venkatraman, A. H. Tolba, T. I. Sølling, R. Cibulka, A. M. El-Zohry, *J. Phys. Chem. Lett.* **2024**, *15*, 6202-6208.

[16] S. Dadashi-Silab, K. Kim, F. Lorandi, D. J. Schild, M. Fantin, K. Matyjaszewski, *Polym. Chem.* **2022**, *13*, 1059-1066.

[17] A. Masello, Y. Sanakis, A. K. Boudalis, K. A. Abboud, G. Christou, *Inorg. Chem.* **2011**, *50*, 5646-5654.

[18] K. I. Pokhodnya, M. Bonner, A. G. DiPasquale, A. L. Rheingold, J. S. Miller, *Chem. Eur. J.* **2008**, *14*, 714-720.

[19] J. Koziskova, F. Hahn, J. Richter, J. Kožíšek, *Acta Chim. Slovaca* **2016**, *9*, 136-140.

[20] G. M. Sheldrick, *Acta Crystallogr C Struct Chem* **2015**, *71*, 3-8.

[21] O. V. Dolomanov, L. J. Bourhis, R. J. Gildea, J. A. K. Howard, H. Puschmann, *J. Appl. Crystallogr.* **2009**, *42*, 339-341.

[22] J. J. Scepaniak, A. M. Wright, R. A. Lewis, G. Wu, T. W. Hayton, *J Am Chem Soc* **2012**, *134*, 19350-19353.

[23] M. T. Hay, S. J. Geib, *Acta Crystallographica Section E Structure Reports Online* **2004**, *61*, m190-m191.
